# Ciliary extracellular vesicles are distinct from the cytosolic extracellular vesicles

**DOI:** 10.1002/jev2.12086

**Published:** 2021-04-27

**Authors:** Ashraf M. Mohieldin, Rajasekharreddy Pala, Richard Beuttler, James J. Moresco, John R. Yates, Surya M. Nauli

**Affiliations:** ^1^ Department of Biomedical & Pharmaceutical Sciences Chapman University Irvine California USA; ^2^ Department of Molecular Medicine The Scripps Research Institute La Jolla California USA; ^3^ Department of Medicine University of California Irvine Irvine California USA

**Keywords:** bioinformatics, ciliary ectosomes, ciliary exosomes, ciliary extracellular vesicles, ciliary protein classes, ciliary vesicle proteome, cytosolic extracellular vesicles, gene ontology, primary cilia, signalling pathways

## Abstract

Extracellular vesicles (EVs) are cell‐derived membrane vesicles that are released into the extracellular space. EVs encapsulate key proteins and mediate intercellular signalling pathways. Recently, primary cilia have been shown to release EVs under fluid‐shear flow, but many proteins encapsulated in these vesicles have never been identified. Primary cilia are ubiquitous mechanosensory organelles that protrude from the apical surface of almost all human cells. Primary cilia also serve as compartments for signalling pathways, and their defects have been associated with a wide range of human genetic diseases called ciliopathies. To better understand the mechanism of ciliopathies, it is imperative to know the distinctive protein profiles of the differently sourced EVs (cilia vs cytosol). Here, we isolated EVs from ciliated wild‐type (WT) and non‐ciliated *IFT88* knockout (KO) mouse endothelial cells using fluid‐shear flow followed by a conventional method of EV isolation. EVs isolated from WT and KO exhibited distinctive sizes. Differences in EV protein contents were studied using liquid chromatography with tandem mass spectrometry (LC‐MS‐MS) and proteomic comparative analysis, which allowed us to classify proteins between ciliary EVs and cytosolic EVs derived from WT and KO, respectively. A total of 79 proteins were exclusively expressed in WT EVs, 145 solely in KO EVs, and 524 in both EVs. Our bioinformatics analyses revealed 29% distinct protein classes and 75% distinct signalling pathways between WT and KO EVs. Based on our statistical analyses and in vitro studies, we identified NADPH‐cytochrome P450 reductase (POR), and CD166 antigen (CD166) as potential biomarkers for ciliary and cytosolic EVs, respectively. Our protein‐protein interaction network analysis revealed that POR, but not CD166, interacted with either established or strong ciliopathy gene candidates. This report shows the unique differences between EVs secreted from cilia and the cytosol. These results will be important in advancing our understanding of human genetic diseases.

## 1 INTRODUCTION

Cell‐derived extracellular vesicles (EVs) were initially deemed as a selective process to remove unwanted cellular components and proteins. Since then, EVs have been associated with various physiological and pathological processes (Aboualaiwi et al., 2009; Arasu et al., 2020). EVs are composed of lipid bilayer that encapsulates proteins and nucleic acids. EVs play important roles in many cellular biological processes, including intercellular communication, making them ideal biomarkers for different signalling pathways (Aspera‐Werz et al., 2019; Atay et al., 2011). EVs are categorized mainly based on their size, function, and protein content. With different morphology, size, protein composition, biogenesis pathway and release mechanism, the heterogeneity of EVs is still loosely defined. However, the present study classifies EVs based on the cellular types or origins, i.e., ciliated (WT) and non‐ciliated (KO) endothelial cells.

Primary cilia protrude upward from almost all mammalian cell types (Bazzi & Anderson, 2014; Becker et al., 2016). Cilia play central roles in human health, coordinating signal transduction and development (Bellmunt et al., 2019; Bonser et al., 2015). Cilia house many receptors, ion channels, transporters, and other sensory proteins critical to their function. Since disruption of ciliary function causes a wide range of diseases, called ciliopathies, many studies have focused on investigating the complexity of ciliary proteomes (Boulanger et al., 2017; Carnino et al., 2019). The trafficking of these functional proteins is regulated by the trafficking adaptor and transition zone barrier mechanisms, which prevent the passage of all non‐ciliary proteins into the cilioplasm during ciliogenesis (Chen et al., 2018, 2019). The machinery that regulates or maintains ciliogenesis is called intraflagellar transport (IFT), which has bidirectional motility along the ciliary shaft. IFT functions as a cargo to transport materials to maintain and support primary cilia formation. In Chlamydomonas, some IFTs have functions particular to the cilia and do not significantly affect cell growth or division, indicating that IFT proteins are not involved in any essential processes other than cilia formation and maintenance (Colombo et al., 2014; Das & Storey, 2014). Importantly, the *IFT88* mutants completely fail to assemble cilia, whereas some other IFT mutants assemble very short cilia. Similarly, a mutation in *IFT88* or its homologue completely ablates primary cilia formation in worms (Deane et al., 2001), mouse kidneys (Das & Storey, 2014), and mouse vascular endothelia (Delling et al., 2013; Edvardson et al., 2010). Compared to control cells bearing primary cilia, these *IFT88* knockout cells have an impaired ciliary assembly (Greening et al., 2015; Haycraft et al., 2007) and potentially lack ciliary vesicles. Therefore, the *IFT88* knockout cells are an important tool in differentiating EV types and to analyze the physiological relevance of primary cilia in mammalian cells.

Structurally, primary cilia consist of multiple subdomains, including the appendages, centrioles, transition zones, and axoneme compartments. Each subdomain is associated with distinctive functions and unique ciliary proteins. Recent studies have emerged associating primary cilia with cilia‐derived vesicle proteins. Interestingly, EVs released from cilia have been suggested to regulate ciliogenesis (Farrer et al., 2009). In addition, the bioactive cilia‐derived vesicles released from the flagella of Chlamydomonas have been associated with proteolytic enzymes that degrade the mother cell wall to release daughter cells (Ferland et al., 2004). Some EVs released from cilia have been suggested to reduce and transport back to the photoreceptor to regenerate functional opsins (Galdzicka et al., 2002). Another proteomic study has associated EVs, isolated from urine, with vital ciliary proteins that have been implicated in human cystic disease and Bardet‐Biedl syndrome (Garcia‐Gonzalo et al., 2011). Other studies have examined the mechanism of ciliary vesicle release and compared the protein compositions between ciliary vesicles and cellular membranes (Gencer et al., 2017; Gould & Raposo, 2013). Despite the significance of these studies, the proteome within this shedding vesicle remains largely unidentified. To better understand the properties and functions of EVs and cilia in human genetic diseases, we investigated the proteome of EVs isolated from ciliated wild‐type (WT) and non‐ciliated *IFT88* knockout (KO) mice endothelial cells. Our study showed that ciliary‐derived EVs are distinct from cytosolic EVs. This distinction is highly significant and will advance our knowledge of the biology of EVs, cilia, and human genetic diseases.

## 2 RESULTS

**FIGURE 1:**
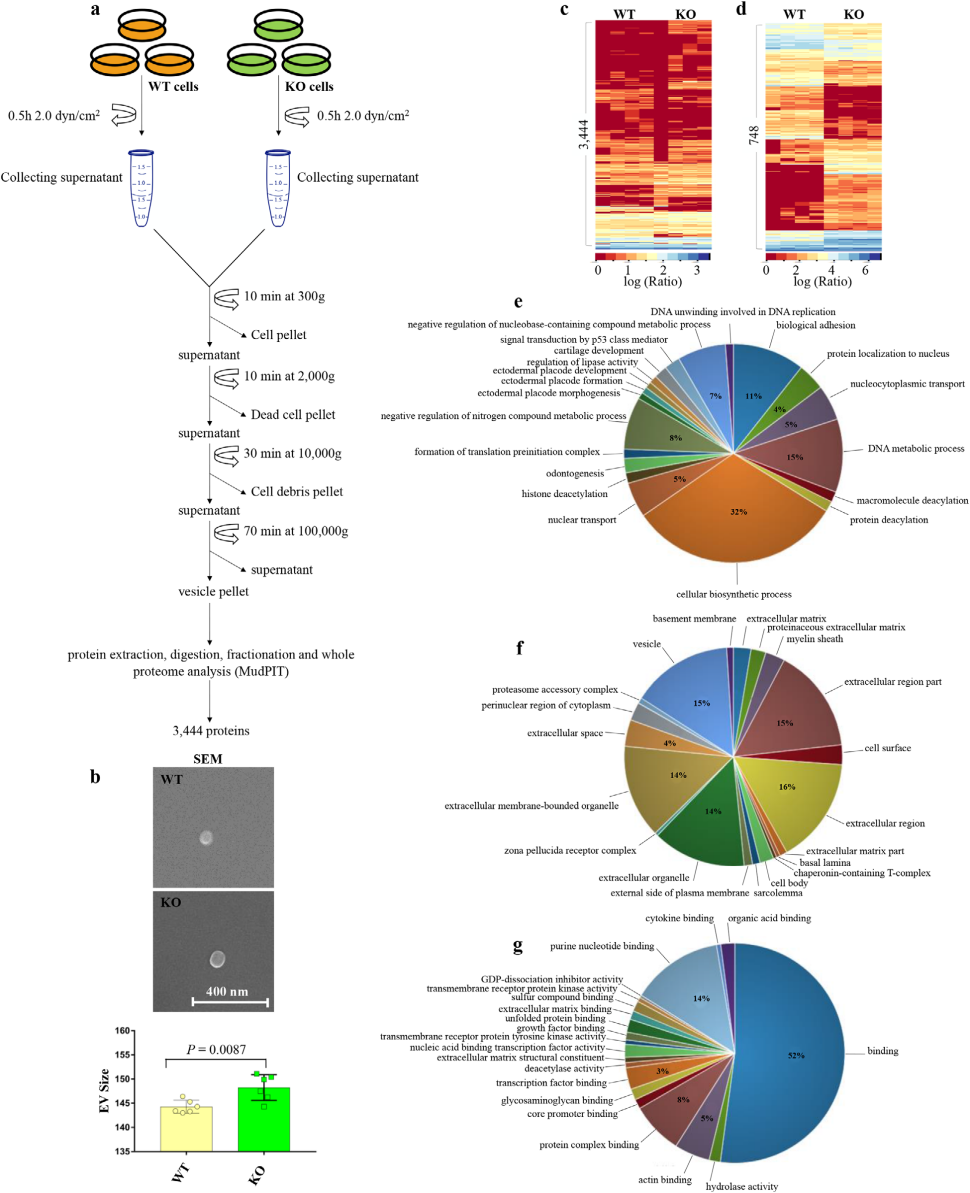
Proteomic analysis of extracellular vesicles (EVs). (A) Overview scheme of EV isolation from ciliated (wild‐type; WT) and non‐ciliated (*IFT88*; KO) cells.(B) Scanning electron microscope (SEM) analysis and quantification of isolated EV size (Figure S1). N = 6 in each group. (C) Cluster analysis of total protein expressions of 3444 proteins between WT and KO cell‐derived EVs. (Table S1).(D) Cluster analysis of differential expressions of 748 proteins between WT and KO cell‐derived EVs (*P* ≤ 0.05) (Table S2). Dataset for cluster analysis were normalized via log transformation. Scale bar indicate protein abundance (0 = low, red; 3 or 6 = high, blue). (E‐G) Three different Gene Ontology (GO) analyses describe the enrichment of the isolated EVs. The ratios of each gene expression within three different GO are described elsewhere (Figure S2). (E) The biological process pie chart describes the biological objectives to which the gene product contributes. (F) The cellular component pie chart describes the localization in the cell where the gene exerts its activity. (G) The molecular function pie chart describes the biochemical activity of each gene product

To examine the size and protein profiles of ciliary and cytosolic EVs, we isolated EVs from WT and KO endothelial cells. EVs were isolated using a standard conventional method using a fluid‐shear flow to induce ciliary vesicle release in control cells, as previously described (Farrer et al., 2009) (See Method; Figure 1a). The size of the isolated EVs was examined using a scanning electron microscope (Figure 1b). We observed that EVs isolated from WT ranged between 30 and 164 nm, and KO between 30 and 175 nm, consistent with previous reports (Hogan et al., 2009; Jana et al., 2018). For more robust quantitative analyses, we studied the mean sizes of WT and KO EVs using dynamic light scattering (Figure S1a,b). There was a significant difference in the mean sizes between WT (144.3 ± 0.5 nm) and KO EVs (148.2 ± 1.1 nm). The number of EVs was significantly decreased in KO compared to WT during shear flow (Figure S1c).

### 2.1 Proteomic analysis of ciliated WT and non‐ciliated KO EVs

Proteins extracted from EVs were further analyzed using tandem LC‐MS‐MS. Comparative proteomics analysis was used to differentiate the WT EV proteome from KO EV proteome. Thus, differences in protein expression could potentially be denoted as ciliary EVs derived from ciliated cells (WT) or cytosolic EVs derived from non‐ciliated cells (KO). The analysis of protein fractions detected a total of 3444 proteins, and only 748 proteins had a significant protein abundance (or spectral count) (Figure 1c,d; Table S1 and S2). Clustering analysis revealed the relative protein abundance of each protein group. The clustered representative proteins exhibited differences in protein expression between WT and KO EVs. To further understand the overall complex relationship between these large datasets of EVs and cellular biology, gene ontology (GO) analysis was used to illustrate the biological processes, cellular components, and molecular functions of the proteomes (Figure 1e‐g, S2) (Jones et al., 2012). Notably, the cellular biosynthetic process, which is defined as any process that modulates the frequency, rate or extent of the chemical reactions and pathways resulting in the formation of substances, comprised the highest percentage among all the biological processes. Not surprisingly, most of the cellular components are part of the EV organelles.

### 2.2 Comparative analysis of proteomes between ciliated WT and non‐ciliated KO EVs

**FIGURE 2:**
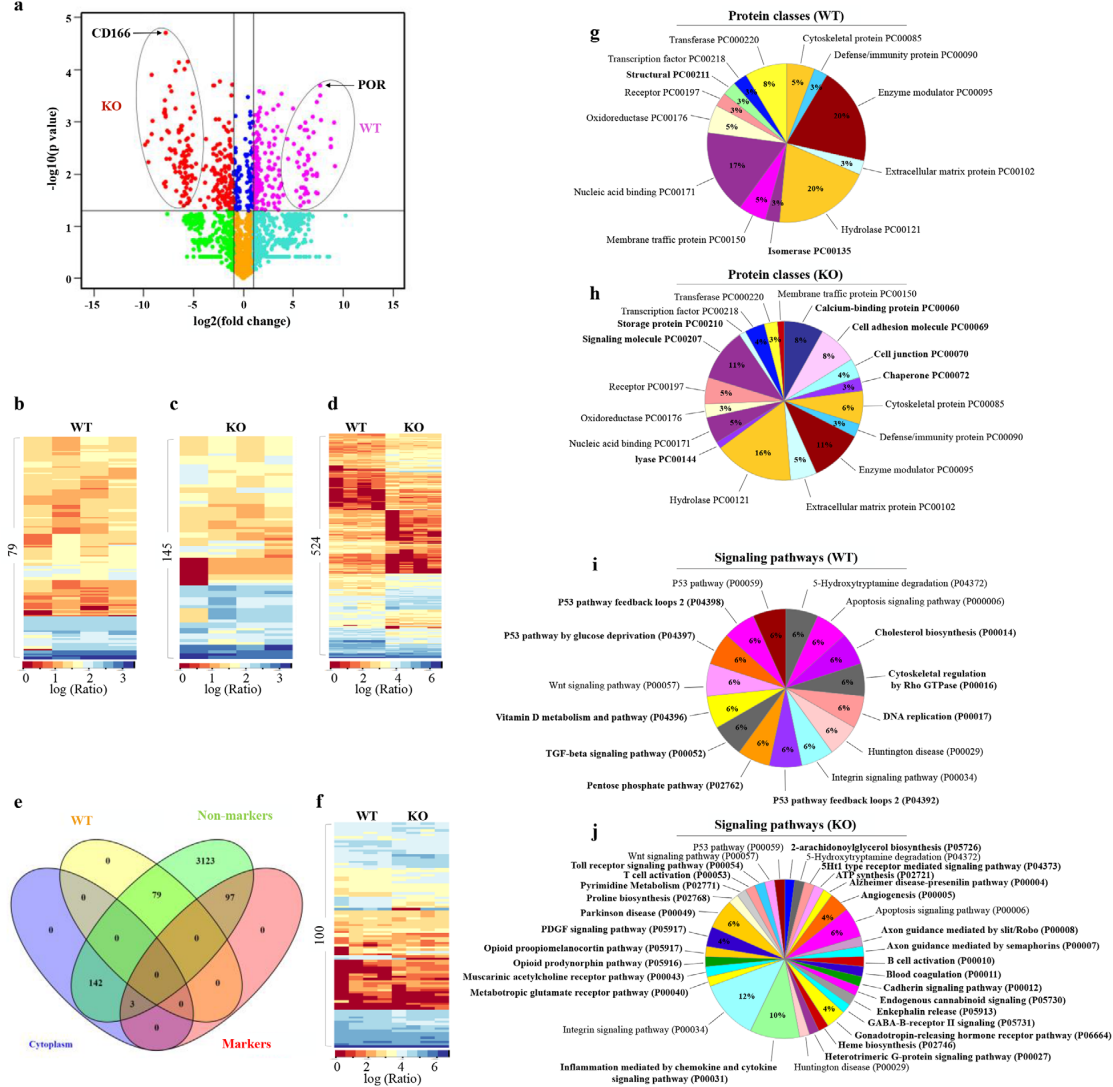
Comparative analyses of ciliated WT and non‐ciliated KO vesicles. (A) A volcano plot of WT (pink) and KO (red) vesicles. Thresholds are indicated by lines and proteins selected as significantly different are highlighted in red and pink dots. Those showing within the oval circles are exclusively expressed in WT or KO vesicles as labeled. The arrows indicate the proteins with the highest *P*‐value in each group. (B) Cluster analysis of 79 proteins exclusively expressed in WT vesicles (Table S3). (C) Cluster analysis of 145 proteins exclusively expressed in KO vesicles (Table S4). (D) Cluster analysis of 524 proteins differentially expressed in wild‐type (WT) and *IFT88* EVs (Table S5). Dataset for cluster analysis were normalized via log transformation. Scale bar indicates protein abundance (0 = low, red; 3 or 6 = high, blue). (E) Venn diagram showing the relationship between proteins identified exclusively in WT or KO vesicles and their relationship as potential EV biomarkers. (F) Cluster analysis of the top 100 EV biomarkers (Table S6). (G‐J) Gene Ontologies analyses describe the enrichment differences between WT and KO cell‐derived vesicles. (G and H) The protein class pie charts describe the function of a gene product (protein class). The percentages show distributions of proteins involved in each class; bolded classes indicate the differences in expressions between WT and KO cell‐derived vesicles; non‐bolded classes indicate the expression observed in both WT and KO cell‐derived vesicles. (I and J) The signalling pathway pie charts describe the genes involved in a coordinated effort to produce cellular responses (signalling pathways). The percentages show the protein distributions involved in each signalling pathway; bolded pathways indicate the difference of expressions between WT and KO cell‐derived vesicles; non‐bolded pathways indicate the shared expressions between WT and KO cell derived vesicles. A complete Gene Ontologies on the biological process, cellular component and molecular function between WT and KO cell‐derived vesicles is shown elsewhere (Figure S3)

Because of the differences in protein abundance, we further examined whether proteins were exclusively expressed in WT and KO EVs. Among the significant abundant proteins in WT and KO EVs, we identified 79 proteins exclusively expressed in WT EVs, 145 proteins exclusively expressed in KO EVs, and 524 proteins found in both EVs (Figure 2a‐c, Table S3‐S5). Although some proteins were found in both EVs, the difference in abundance was very noticeable, revealing a unique distinction between the two EV types (Figure 2d). To evaluate these findings with already known EV biomarkers, we compared the top hundred known EV biomarkers with our dataset (Table S6). Ninety‐seven of the known EV biomarkers were matched with proteins expressed in both WT and KO EVs, confirming the EV presence in our analyzed proteomes. The remaining three known EV biomarkers were found among proteins that expressed exclusively in KO EVs (Figure 2e,f). Most EVs have been suggested to play a specific role in a signalling pathways (Kowal et al., 2016‐Lötvall et al., 2014). Therefore, we designed GO analyses more targeted towards protein function (protein classes) and signalling pathways that were exclusively involved in EVs (Figure 2g‐j). Within the protein classes, 29% of the proteins were differentially expressed in WT and KO EVs. Within the signalling pathway, 75% of the distinct pathways were identified between the WT and KO EVs. Additional GO analyses were also performed to show that the identified KO EVs had unique biological processes, cellular components, and molecular functions (Figure S3).

### 2.3 Targeting ciliated WT and non‐ciliated KO vesicle biomarkers

**FIGURE 3:**
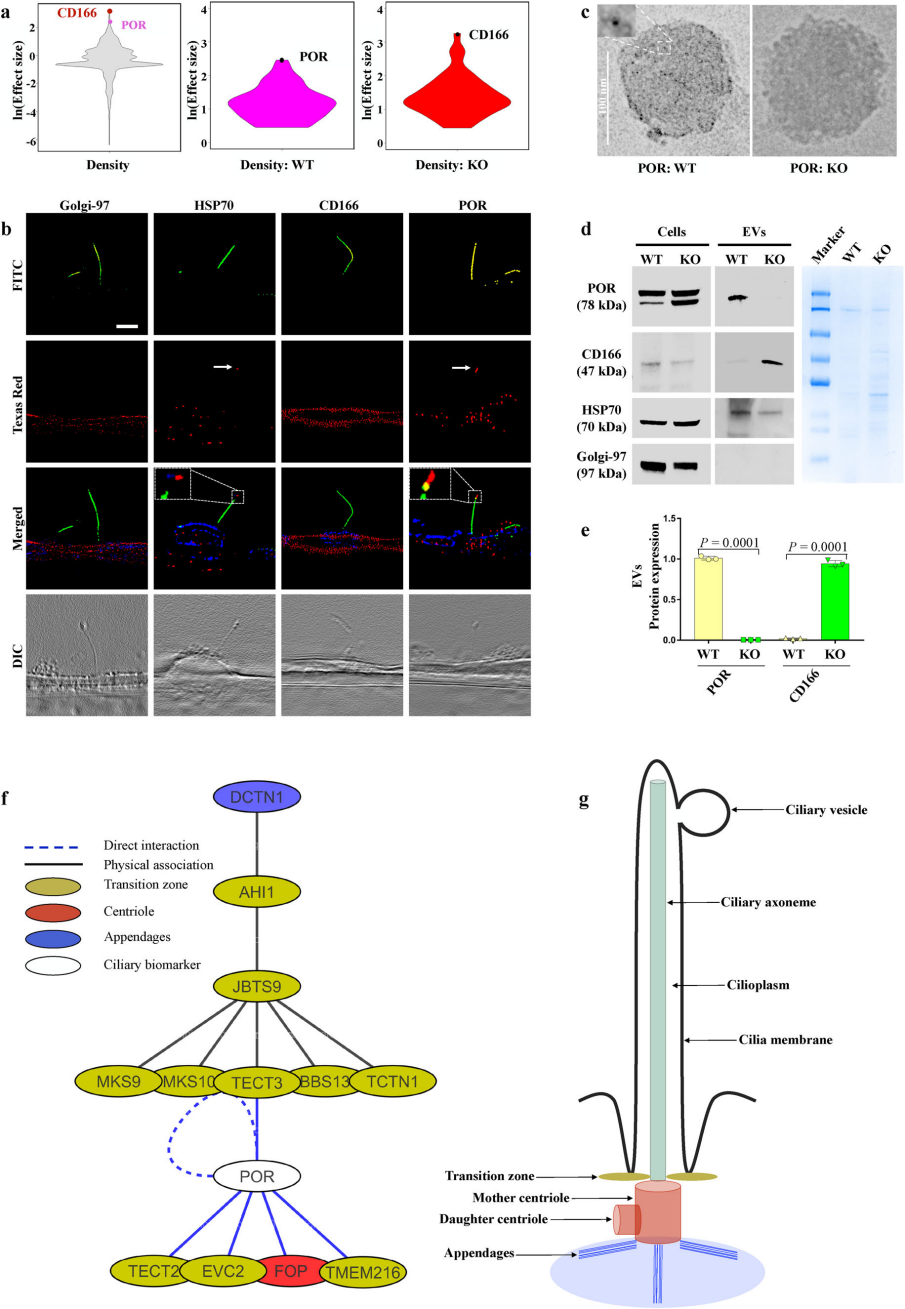
Targeting ciliated WT and non‐ciliated KO vesicles biomarkers. (A) Violin plots showing the distributions of both biomarkers in the entire proteome, in protein expressed exclusively in WT and KO cell‐derived vesicles. CD166 and POR biomarker proteins are indicated in all plots. (B) POR and Hsp70 (red) localized to primary cilia (green). CD166 and Golgi‐97 (red) localized only to cytosol. Hsp70 and Golgi‐97 were used as positive and negative controls for EV, respectively. Acetylated‐α‐tubulin (green) and dapi (blue) were used as ciliary and nuclear markers. High‐resolution differential interference contrast images are shown to confirm the presence of primary cilia and ciliary vesicles. N≥10 for each group. (C) TEM images show numerous anti‐POR immunogold‐nanoparticles (5 nm) localization in WT but not in KO vesicles. (D) A western blot analysis shows POR and CD166 protein expressions in cell lysate and isolated EVs. Hsp70 and Golgi‐97 were used as positive and negative controls for EVs, respectively. The coomassie blue staining shows equal loading of EV lysate. (E) Bar graph shows quantitation of POR and CD166 in WT and KO. N = 4 in each group. (F) A network interaction analysis showing POR interacts with sub‐ciliary compartments proteins (beige, transition zone; red, centriole; blue, appendages; white, POR). (G) A primary cilium sketch showing each sub‐ciliary compartment with its given colour in the network interaction. A complete network interaction analysis showing the interactions between POR and CD166 and other sub‐ciliary compartments proteins is shown in (Figure S5)

Based on the statistical analyses of proteins expressed exclusively in WT and KO EVs, we selected POR and CD166 as potential biomarkers for WT and KO EVs, respectively (Figure 3a, S4). To validate the specificity of these biomarkers for WT and KO EVs, we performed immunofluorescence studies (Figure 3b). We confirmed the localization of the POR biomarker in WT EVs. On the other hand, CD166 was only localized in the cytosol and not in the cilia. Hsp70 and Golgi‐97 were used as positive and negative controls for EV biomarkers (Luxmi et al., 2019; Marino et al., 2019). Because POR as a WT vesicle biomarker was also seen in the cytosol, we directly examined POR from WT and KO EV pools for further confirmation (Figure 3c‐e). Transmission electron microscopy (TEM) also confirmed the localization of anti‐POR immunogold‐nanoparticles in EVs isolated from WT; EVs isolated from KO cells were used as a negative control. Furthermore, the immunoblot analysis validated POR expression only in WT vesicle lysate and not in KO vesicle lysate. CD166 was exclusively expressed in KO vesicle lysates but not in WT vesicle lysates. This confirmed the hypotheses that WT cells predominantly released ciliary EVs, while KO cells released only cytosolic EVs. Because this was the first time that POR was reported as a ciliary protein, we examined a protein‐protein interaction network to understand the interaction between POR and known ciliary proteins (Figure 3f,g and S5). When we extended the network interaction studies to include CD166, we found that only POR, but not CD166, interacted with the ciliary transition zone, centriole, and appendage proteins. This further indicates the relevance of isolated WT EVs to primary cilia.

## 3 DISCUSSION

Proteomic comparative analyses revealed two distinct EV pools with significantly different sizes and biomarkers. We identified 748 EV proteins, of which 79 were exclusively expressed in WT EVs, 145 were exclusively expressed in KO EVs, and 524 were shared between both EVs. Even for the proteins shared between WT and KO EVs, we noticed a clear shift in protein abundance between the two EV types (Figure 2d, S4d). Previously, the focus on EV proteins revealed massive information about the protein composition and crucial roles of EVs (Masyuk et al., 2010‐Mohieldin et al., 2015). However, the overlapping size distributions, protein compositions and structural morphologies have challenged all efforts to pinpoint the differences in signalling pathways, physiological functions, and nomenclatures for EVs (Mohieldin et al., 2016, 2020). In addition, all these efforts are merely based on the conventional method of isolating cytosolic EVs (Nauli et al., 2008). Here, we have shown a new classification of EVs as cytosolic and ciliary EVs.

Although ciliary EVs have been previously observed along the ciliary shaft, no comparative analysis has been performed to examine biomarkers specific to these EVs (Ferland et al., 2004; Nozaki et al., 2018; Pazour et al., 2000). The GO comparative analysis revealed the unique expression of protein classes and signalling pathways in each EV type (Figure 2g‐j). Even though the GO categories are too broad to indicate the source of EVs, a more involved analyses might provide insight into their functions. Comparing the differences between the two EV types, we found unique protein classes and signalling pathways in each of them. This may indicate the unique physiological role of each EV type. For example, an isomerase protein class is present in WT but not in KO EVs (Figure 2g,h). This exclusive presence of the isomerase class may be significant because isomerases regulate ciliary beat frequency in airway cells (Phua et al., 2017). Given the diverse roles of cilia, many of the signalling pathways from GO seem to fit with their physiological roles (Figure 2i). For example, the signalling pathways of cholesterol biosynthesis, vitamin D metabolism, TGF‐β, Rho GTPase, and P53 that appear only in WT EVs have all been reported to be associated with primary cilia functions (Popovici et al., 1999; Shaheen et al., 2011). However, the observed associations of DNA replication and pentose phosphate signalling pathways with ciliary function are yet to be investigated.

We selected two biomarkers out of 79 or 145 proteins that were exclusively expressed in WT or KO EVs based on their high abundance and statistical significance (Figure 2a, 3a). Thus, POR and CD166 biomarkers were selected for WT and KO EVs, respectively. Because the immunofluorescence analysis showed the localization of POR on the ciliated WT EVs, as well as in the cytosol of the cell, we further examined its expression by TEM and immunoblotting (Figure 2b‐e). These analyses showed a high specificity of each biomarker for the two EV types, independently. Advantageously, using these specific biomarkers independently would for the first time allow investigators to differentiate between ciliary and cytosolic EVs. More importantly, these biomarkers might help in closing the knowledge gap regarding the difference in cytosolic EV protein abundance and exclusively expressed proteins between WT and KO EVs. EV formation, trafficking and secretion are likely regulated through different pathways, especially in the absence of primary cilia.

The WT vesicle biomarker network analysis revealed POR interactions with the ciliary transition zone, centriole, and appendage proteins. Noticeably, all ciliary genes described in the network are either established candidates or strong ciliopathy candidate genes. The established ciliopathy genes are AHI1, JBTS9, MKS9, MKS10, TECT3, TECT2, BBS13, EVC2, TMEM216 and TCTN1, while genes that are candidates for ciliopathy include DCTN1 and FOP (Stewart et al., 2016; Young & Bok, 1969). These findings further substantiate the association of the WT EV biomarker (POR) with primary cilia proteins. Further, comprehensive network analyses revealed a cross‐talk among proteins that were exclusively expressed in ciliary WT EVs (Figure S6a), as well as with several shared proteins between WT and KO EVs (Figure S6b). This might further suggest a strong relationship between these proteins and their involvement in more synchronized biological processes.

We have extensively crosschecked our results with earlier studies investigating the link between primary cilia and EVs. Recently, exocyst‐containing vesicles, which localize at the tip and sides of primary cilia (Zuo et al., 2009), have been shown to be associated with the biogenesis of EVs in mammalian renal cells (Zuo et al., 2019). We found that an extended list of EV proteins in our proteomic data overlaps with the earlier proteomic data of these exocyst‐containing vesicles. A list of 106 EV proteins matched our data (Table S7). Because the EV proteome isolated from cilia has been shown to exhibit ciliary membrane protein markers (Boulanger et al., 2017), we further investigated these bona fide cilia proteins in our proteome. Not surprisingly, we found that 37.9 % of the exclusively expressed EV proteomes in ciliated WT EVs were previously reported as bona fide ciliary proteins (Table S8). This high turnout of bona fide ciliary proteins in our proteome further supports our studies on isolating ciliary EVs.

In summary, we have shown in this report two distinctive EV types with unique biomarkers. Overall, we reported 79 proteins exclusively expressed in WT EVs, 145 proteins exclusively expressed in KO EVs, and 524 proteins in both EVs. The GO analysis suggests that each vesicle has its unique protein classes and signalling pathways. The results of this study will serve as the fundamentals on which our understanding of the biology of EVs, especially ciliary EVs, will advance.

## CONFLICTS OF INTEREST

No competing interests declared.

## AUTHOR CONTRIBUTIONS

Ashraf M. Mohieldin designed research, collected data, analyzed data, drafted the manuscript and oversaw the entire progress of the project. Rajasekharreddy Pala assisted in proteomic analysis (double‐blind). Richard Beuttler assisted with R project for statistical computing software. James J. Moresco and John R. Yates supported proteomic studies and provided all relevant materials. Surya M. Nauli conceived the idea, designed and oversaw the experimental progress. All authors were participating in finalizing the draft of the manuscript.

## Supporting information

Supporting Information
