# Ciliary extracellular vesicles are distinct from the cytosolic extracellular vesicles

**DOI:** 10.1002/jev2.12119

**Published:** 2021-07-22

Ashraf M.Mohieldin, Rajasekharreddy Pala, Richard Beuttler, James J. Moresco, John R. Yates III, SuryaM. Nauli ‘Ciliary extracellular vesicles are distinct from the cytosolic extracellular vesicles’, *Journal of Extracellular Vesicle*, 2021, **10,** (6), e12086.

In this article, the in‐text citations for the references were presented in the incorrect order. We have corrected the in‐text citations for the references in the published article so that they now appear in the correct order. The publisher regrets the error.

