# Supplementary material for: Ciliary extracellular vesicles are distinct from the cytosolic extracellular vesicles
Source: J Extracell Vesicles. 2021 Apr 27;10(6):e12086. doi: 10.1002/jev2.12086 (PMC8077156; doi:10.1002/jev2.12086)
Supplement: Supplementary file 1 — Supporting Information [file JEV2-10-e12086-s001.pdf]

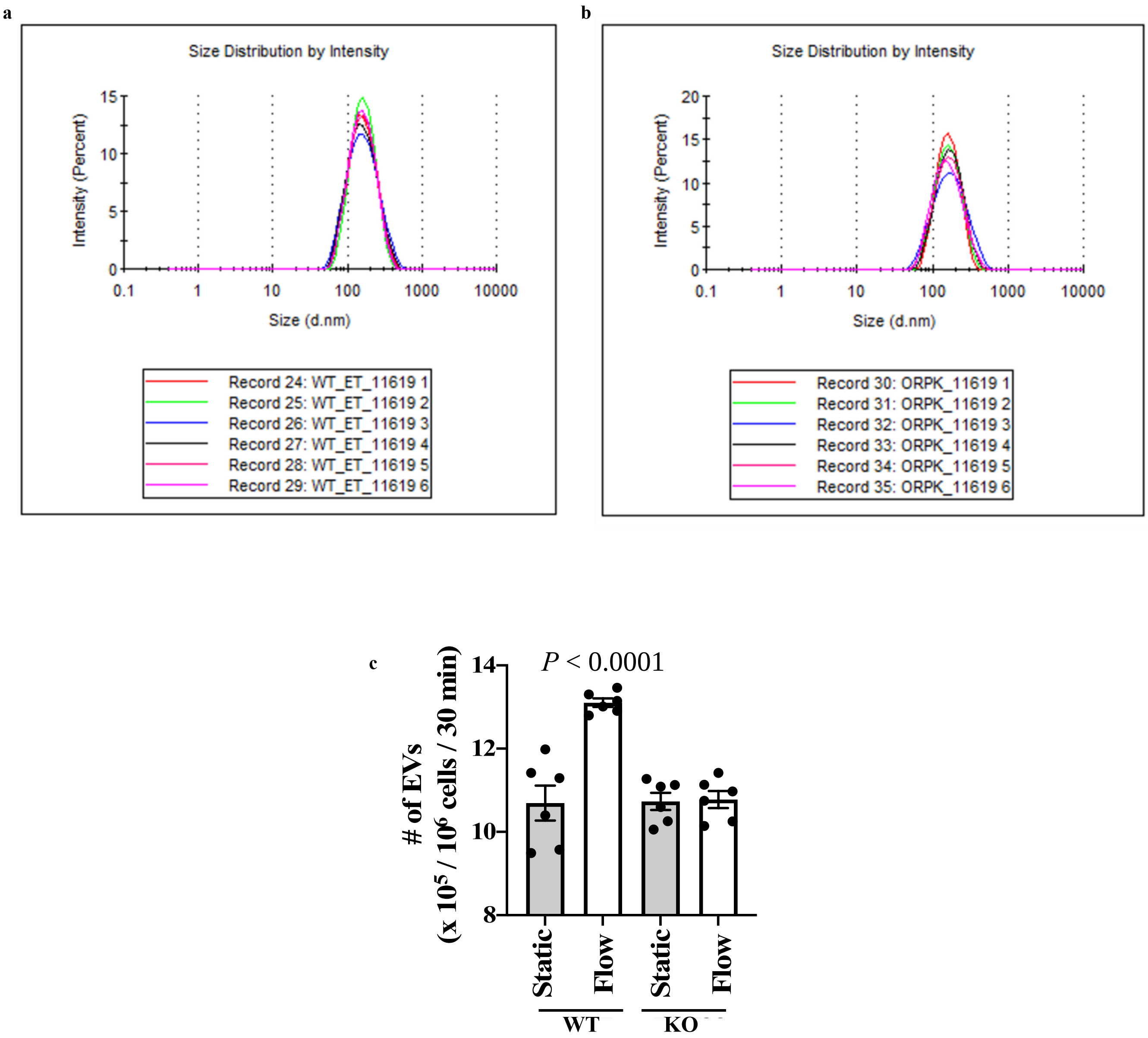

Figure S1. Size and number of EV quantifications

(A-B) The diameters of both WT (A) and KO (B) vesicles were measured using the dynamic light scattering.

(C) The number of EVs were significantly decreased during shear-flow in KO cells compared to control WT.

N=6 in each group.

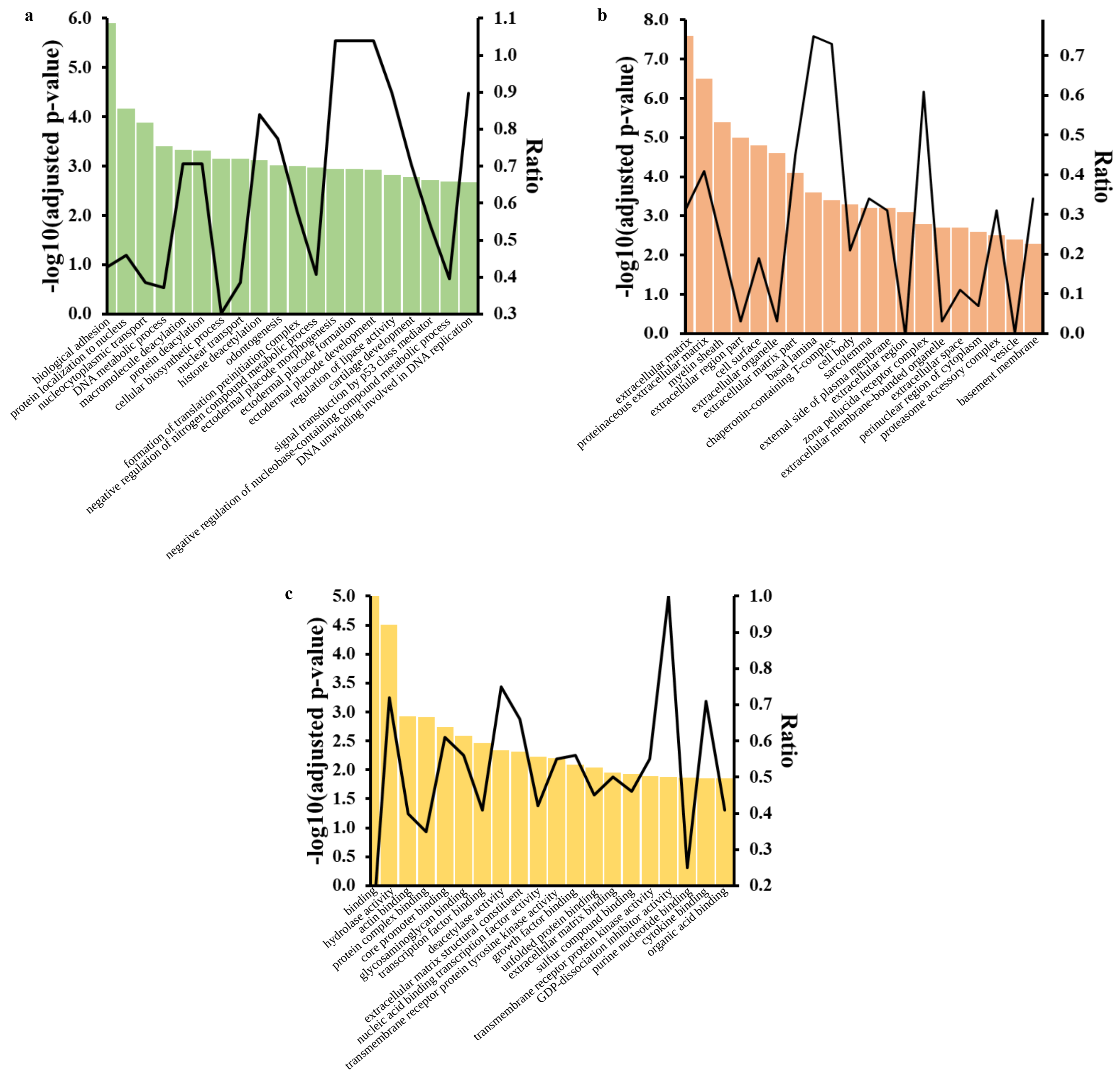

Figure S2. Gene Ontology (GO) analysis of proteomic data

(A-C) The ratio (line graph) and the adjusted p-value (bar graph) show results of the three different GO analyses. The ratio represents the significantly expressed genes involved over the total gene in each category of the (A) biological processes, (B) cellular component and (C) molecular function, respectively.

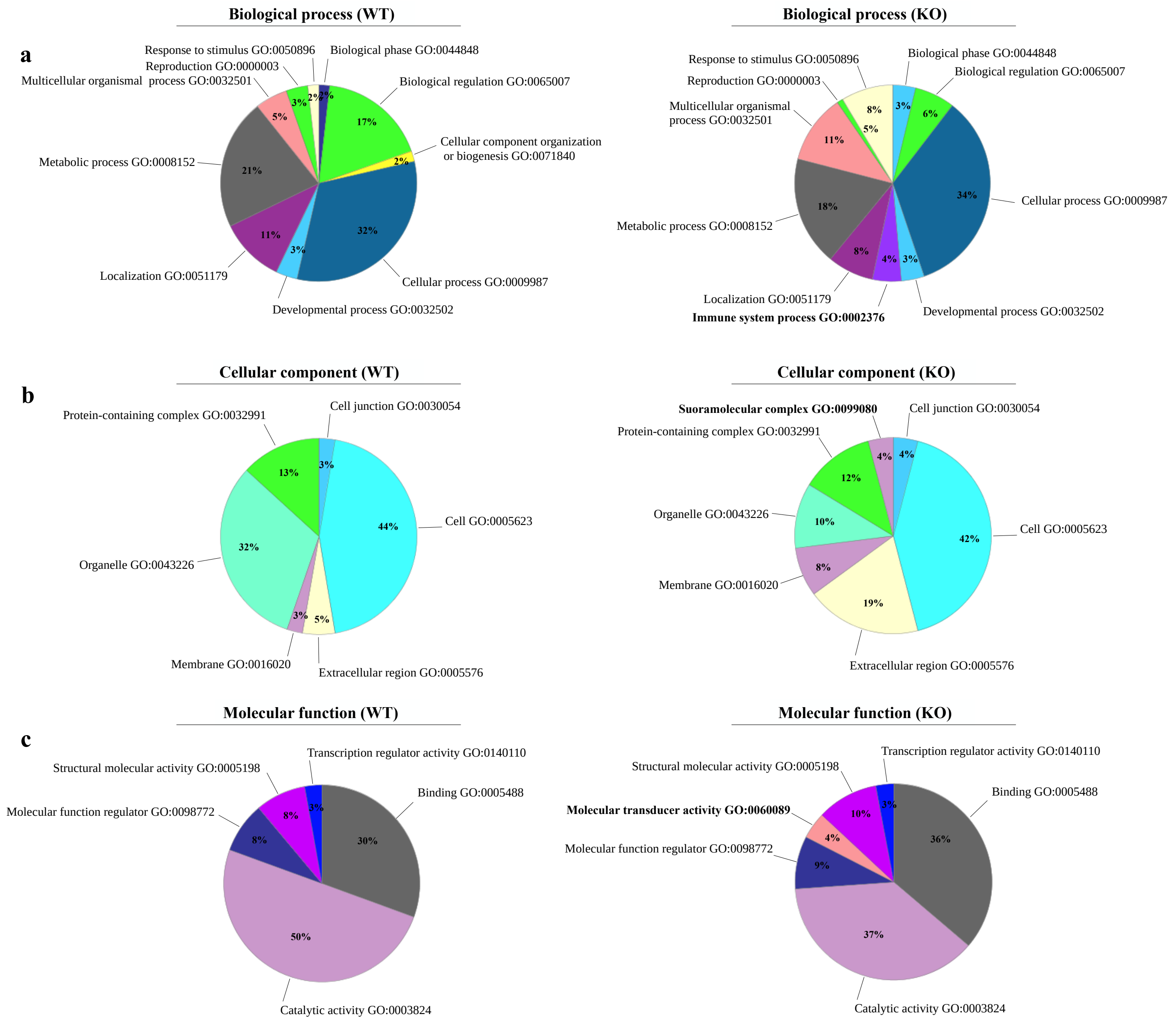

Figure S3. Comparative GO analyses between ciliated WT and non-ciliated KO vesicles

(A) the biological process pie chart describes the biological objective to which the gene product contributes.

(B) the cellular component pie chart describes the localization of the proteins in the cell where the gene exerts its activity.

(C) the molecular function pie chart describes the biochemical activity of a gene product. The percentages show the total protein involved in each process; bolded process indicates the difference of expression between WT and KO cell-derived vesicles; non-bolded process indicates the expression observed in both EVs.

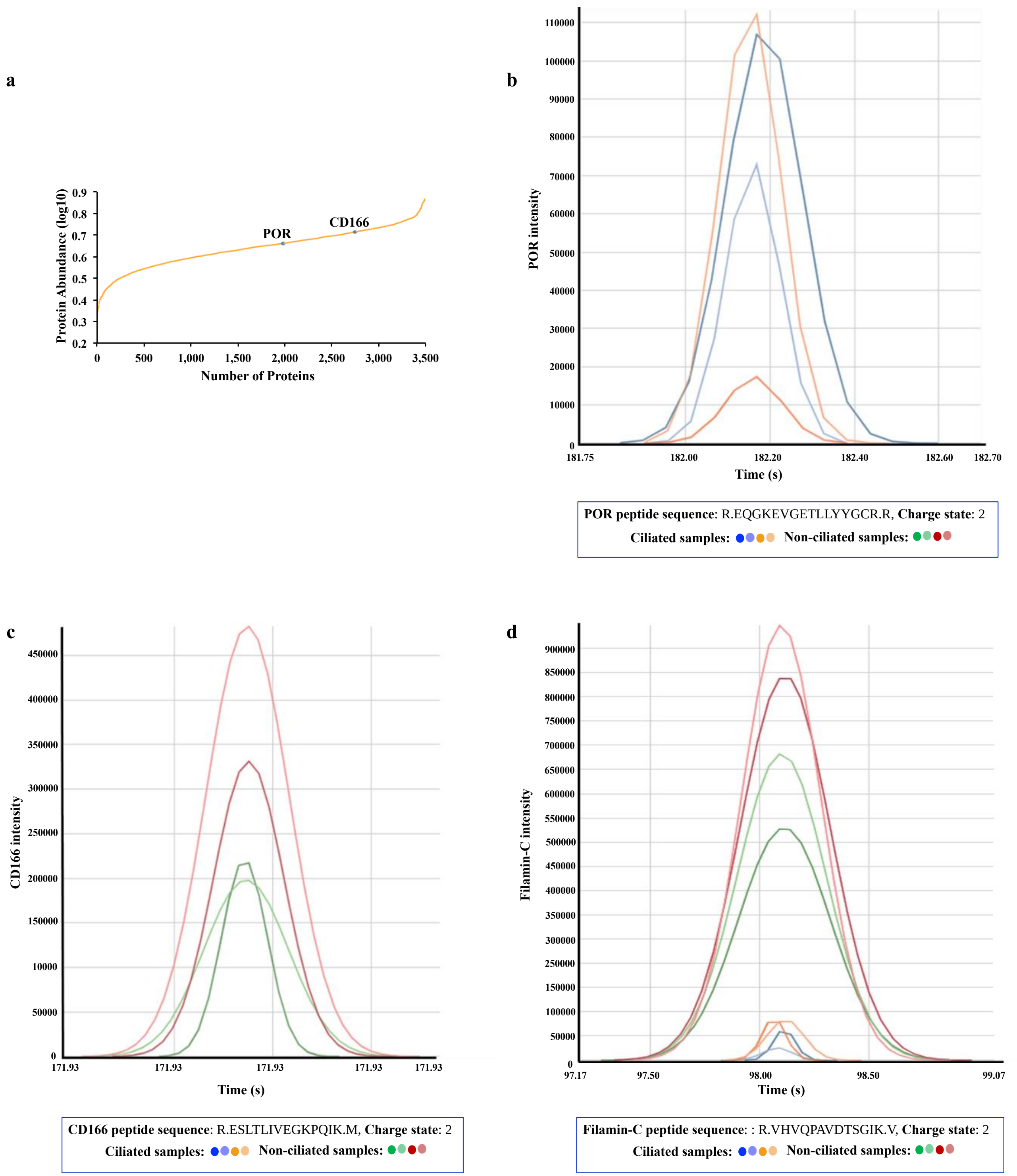

Figure S4. Analysis of relative expressions of ciliated WT and non-ciliated KO vesicle biomarkers

(A) line graph showing protein abundance of POR and CD166 biomarkers exclusively expressed in WT and KO cell-derived vesicles, respectively.

(B-D) chromatographs indicating the detection of POR , CD166 and filamin-C.

(B) POR peptide was detected at 182 second in the four independent samples from ciliated WT cells (blue, light blue, orange and light orange).

(C) CD166 peptide was detected at 171 second in the four independent samples from non-ciliated KO cells (green, light green, red and light red).

(D) Differentially expressed filamin-C peptide between WT and KO cell-derived vesicles was detected at 98 second (blue, light blue, orange, light orange, green, light green, red and light red).

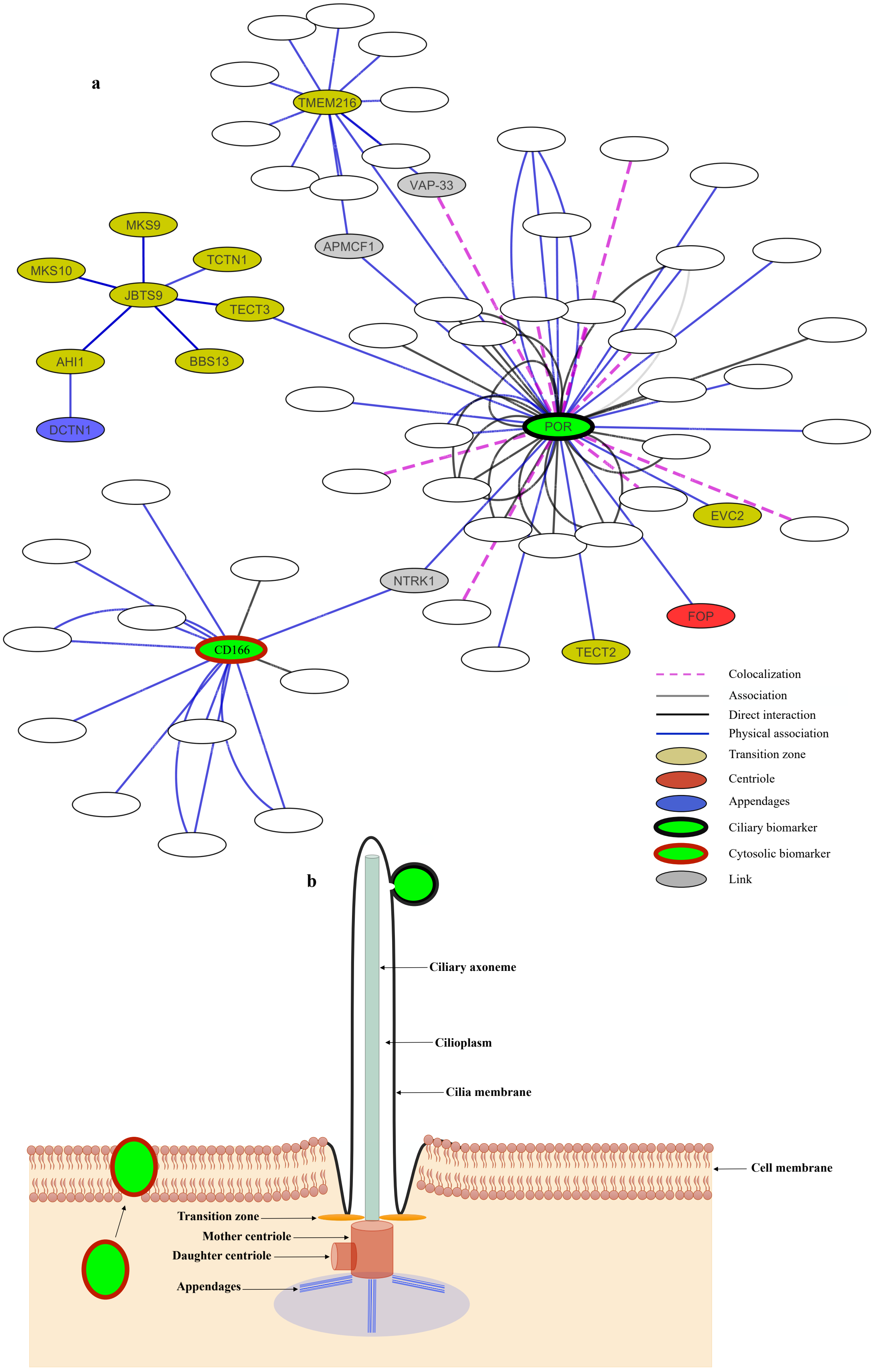
a
TMEM216
VAP-33
APMCF1
MKS9
MKS10
TCTN1
JBTS9
TECT3
AHI1
BBS13
DCTN1
POR
EVC2
NTRK1
FOP
TECT2
CD166
Colocalization
Association
Direct interaction
Physical association
Transition zone
Centriole
Appendages
Ciliary biomarker
Cytosolic biomarker
Link
b
Ciliary axoneme
Cilioplasm
Cilia membrane
Cell membrane
Transition zone
Mother centriole
Daughter centriole
Appendages

Figure S5. Network interaction among POR, CD166 and other ciliary proteins

(A) a network interaction analysis shows POR and CD166 interaction with other sub-ciliary compartments proteins (orange, transition zone; red, centriole; blue, appendages).

(B) a primary cilia sketch indicates each sub-ciliary compartment with its given color and localization of ciliary and cytosolic vesicles within the cell.

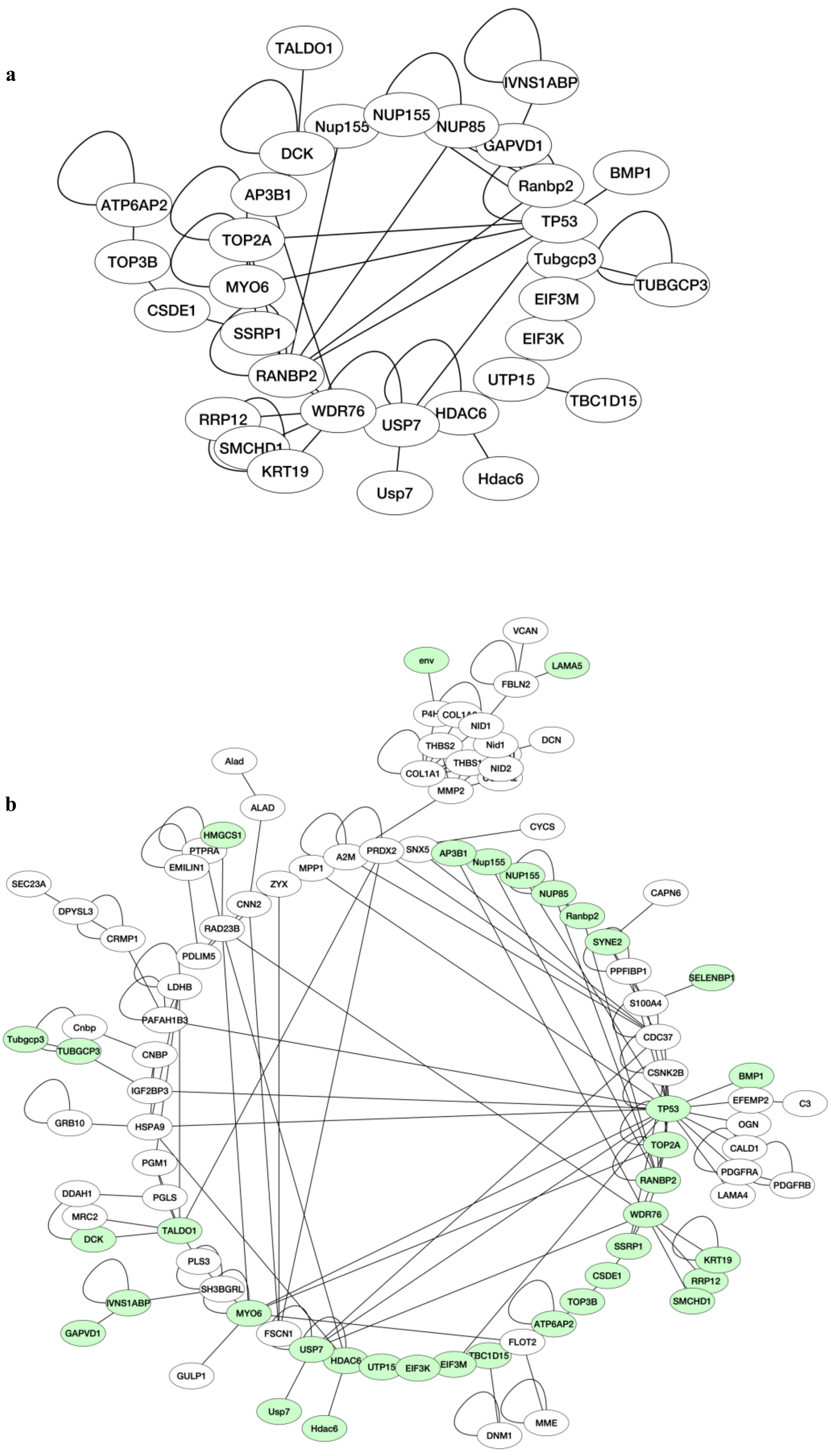
a
TALDO1
IVNS1ABP
NUP155
Nup155
NUP85
GAPVD1
DCK
Ranbp2
BMP1
ATP6AP2
AP3B1
TP53
TOP2A
Tubgcp3
TOP3B
MYO6
TUBGCP3
EIF3M
CSDE1
SSRP1
EIF3K
RANBP2
UTP15
TBC1D15
WDR76
HDAC6
RRP12
USP7
SMCHD1
KRT19
Hdac6
Usp7
b
VCAN
env
LAMA5
FBLN2
NID1
Nid1
DCN
THBS2
NID2
COL1A1
MMP2
Alad
ALAD
HMGCS1
PTPRA
EMILIN1
A2M
PRDX2
SNX5
AP3B1
Nup155
CYCS
NUP155
MPP1
ZYX
NUP85
SEC23A
CNN2
DPYSL3
Ranbp2
CAPN6
RAD23B
CRMP1
SYNE2
PDLIM5
PPFIBP1
SELENBP1
LDHB
S100A4
PAFAH1B3
CDC37
Cnbp
Tubgcp3
TUBGCP3
CNBP
CSNK2B
BMP1
IGF2BP3
TP53
EFEMP2
C3
GRB10
HSPA9
OGN
TOP2A
CALD1
PGM1
PDGFRA
PDGFRB
RANBP2
LAMA4
DDAH1
PGLS
WDR76
MRC2
TALDO1
DCK
SSRP1
KRT19
PLS3
CSDE1
RRP12
SH3BGRL
SMCHD1
TOP3B
IVNS1ABP
MYO6
ATP6AP2
GAPVD1
FSCN1
FLOT2
USP7
HDAC6
UTP15
EIF3K
EIF3M
TBC1D15
GULP1
Usp7
Hdac6
MME
DNM1

Figure S6. Network interaction among proteins expressed in ciliated WT vesicles

(A) a network interaction analysis shows interaction among proteins that are exclusively expressed in WT (Table S3).

(B) a network interaction analysis shows interaction among proteins that are exclusively expressed in ciliated WT vesicles (color light green) and proteins that are expressed in both ciliated WT and non-ciliated KO vesicles (color white).

Table S1. Analysis of protein compositions from isolated WT and KO cell-derived vesicles has revealed 3,444 protein fractions.

Table S2. Only 748 proteins fractions with significance P-value ≤ 0.05 are further analyzed.

Table S3. Shown here are 79 protein fractions expressed exclusively in isolated EVs from WT (ciliated cells) after inducing the release of ciliary vesicle by fluid-shear flow.

Table S4. Shown here are 145 protein fractions expressed exclusively in isolated EVs from KO (non-ciliated cells) after inducing the release of ciliary vesicle by fluid-shear flow.

Table S5. Shown here are 524 proteins expressed in both ciliated WT and non-ciliated KO cell-derived vesicles.

Table S6. Shown here are top 100 extracellular vesicle biomarkers matched to the proteomic dataset (Figure 2 e,f).

Table S7. A comparative analysis with primary cilia targeted vesicle study (exocyst-containing vesicles) shows the overlap with an extended list of our significantly expressed EV proteome.

Table S8. Shown here are the list of 30 exclusively expressed EV proteins isolated from ciliary WT (Table S3) that matched with known cilia specific markers.

Supplemental Information

Ciliary extracellular vesicles are distinct from the cytosolic extracellular vesicles.

Authors: Ashraf M. Mohieldin[1,3], Rajasekharreddy Pala[1], Richard Beuttler[1], James J. Moresco[2], John R. Yates, III[2], Surya M. Nauli[1,3]

Cell culture

Ciliated (control) and non-ciliated (*IFT88*) mouse knockout endothelial cells were cultured in Dulbecco's Modified Eagle Medium (DMEM) (*Corning Cellgro*), 10% fetal bovine serum (FBS) (*HyClone*) and 1% penicillin/streptomycin (*Corning Cellgro*) at 37°C in a 5% CO_2 incubator. Prior to all experiments, cells at 70-80% confluence were differentiated for 24-48 hours in serum-free and EV-free media (*Thermofisher).*

Isolation of WT and KO cell-derived vesicles

To isolate ciliary vesicles from WT and KO endothelial cells, cells were grown in a 150-mm culture dish in quadruplicate and special vesicle-depleted media was used (*Thermofisher)*. At 70-80% confluence, cell culture dishes were placed on an orbital shaker (*Cole-Parmer*) for 30 minutes at 350 rotations per minute (2.0 dyn/cm^2). Growth media was then collected respectively and spun at four different speeds: 300×*g* for 10 minutes, 2,000×*g* for 10 minutes (*Thermofisher*; Heraeus Megafuge 8R) 10,000×*g* for 30 minutes and 100,000×*g* for 70 minutes (*Thermofisher*; Sorvall wx+ ultra series centrifuge; Rotor: AH-629). The supernatants were collected in all first three rounds of centrifugation. In the fourth round of centrifugation, the supernatants were discarded, and the vesicle pellets were re-suspended in cold-PBS for another centrifugation at 100,000×*g* for 70 minutes to ensure purity of the isolated vesicles. Vesicle pellets were

then re-suspended in either PBS, Radioimmunoprecipitation assay buffer (RIPA buffer), or 2.5% glutaraldehyde for different analyses.

Scanning electron microscopy

Re-suspended vesicle pellets were fixed in 2.5% glutaraldehyde and kept for overnight at 4°C. Vesicles were then washed three times with PBS, dehydrated through a graded series of ethanol for 10 minutes each, and incubated in hexamethyldisilazane for 1 hour on ice. Samples were then mounted, air dried, sputter-coated with gold, and examined under Zeiss SEM microscope (ZEISS; Sigma 300).

Transmission electron microscopy

Isolated vesicles from control and *IFT88* knockout endothelial cells were re-suspended in PBS at room temperature. Carbon/formvar copper grids (*Electron Microscopy Sciences*) were glow discharged under ultraviolet light (UV) for one hour. Diluted vesicles were loaded into the grids for 30 minutes. Vesicles were then blocked with 1% BSA, washed with PBS, incubated with anti-rabbit POR antibody (*Abcam*), washed with PBS, incubated with 5 nm rabbit IgG gold conjugate nanoparticle (*Nanocs*), washed with PBS, and negatively stained with 2% uranyless acetate and 3% lead citrate (*Electron Microscopy Sciences*). The vesicles were examined under a JEOL JEM-2100F microscope operated at 200kV with a Field Emission electron source. Images were recorded on a Gatan OneView camera at magnification of 40,000x, corresponding to 2.8 angstrom per pixel at specimen space.

Immunocytochemistry

Cells were stained following a standard protocol, as described previously [1]. Briefly, cells were washed with sodium cacodylate buffer, incubated in 3% glutaraldehyde for 10 minutes, incubated in 1% Triton-

X for 5 minutes, and all primary antibodies were diluted in a 10% FBS solution. Primary antibodies for golgi-97 (1:500, *cell signaling*), HSP70 (1:100, *Abcam*), CD166 (1:500, *Abcam*), POR (1:500, *Abcam*), and acetylated-α-tubulin (1:1000, *Sigma*), were incubated for overnight at 4°C. The secondary antibodies FITC fluorescence (1:500, *Pierce*) and Texas-Red fluorescence (1:500, *Pierce*) were diluted in a 10% FBS solution, and incubated for 1 hour at room temperature. Cells were then rinsed with cacodylate buffer, mounted with DAPI (*Vector laboratories*), and images were taken with Nikon Ti-E using Nikon Element software. To take advantage of the naturally rigid cilium, we used Tungsten microwire to enable imaging single cells from the side as discussed previously [2, 3]. This strategy would overcome the issues with optical distortions of traditional 2-dimensional cell imaging. For our studies, we previously ordered the precision microwire from H.P. Reid, Inc. in Palm Coast, FL. Because the company has ceased to exist, a replacement of the Tungsten microwire can be obtained through Luma Metall AB, Sweden (Wire quality #823; Surface finish #42). The precision microwire was prepared by coating the wire with type I collagen (50 μg/mL in 0.02N acetic acid) to provide a conducive surface for cell attachment and growth. The microwires were then UV-sterilized for 30 min and mounted on the imaging chamber before seeding the cells. If needed the microwire can be gently rotated to observe the confluency of the cells around the microwire.

Immunoblotting

Re-suspended vesicle pellets in RIPA buffer containing protease inhibitor (*Roche*) were vortexed for 30 minutes and centrifuged for 15 minutes at 10,000×*g* (accuSpin Micro 17, *Fisher scientific*). Concentration of protein lysates were then determined using Pierce BCA Protein Assay Kit (*Thermo Fisher Scientific),* and protein lysates were then analyzed in a standard 10% gradient sodium dodecyl sulfate-polyacrylamide gel electrophoresis (SDS-PAGE). To confirm equal loading, gels were stained with Coomassie blue dye

(*Bio-Rad*, blue R-250), washed with de-staining solution (*Bio-Rad*), and imaged with the ChemiDoc XRS+ system (*Bio-Rad*). Using the dry method, proteins were transferred to nitrocellulose membrane (*Thermo Fisher Scientific*) and blocked with 5% milk. Membranes were then incubated with primary antibodies (1:500, POR; 1:500, CD166; 1: 500, golgi-97; and 1:100, HSP70) for overnight at 4°C and secondary antibodies (1:1000 for both anti-mouse and anti-rabbit) for 1 hour at room temperature. Membrane was then rinsed and imaged with the ChemiDoc XRS+ system (*Bio-Rad*).

Proteomic analyses

Samples preparation: Vesicle pellets from four independent samples of control wild-type and *IFT88* (total of 8 samples) were re-suspended in RIPA buffer. Samples were next reduced with 5 mM TCEP (*Thermofisher*) and alkylated in the dark for 15 minutes with 10 mM iodoacetamide (*Thermofisher*). Protein samples were then digested for 18 hours at 37°C in 2 M urea, 100 mM Tris, 1 mM calcium chloride and 2 µg of trypsin (*Promega*), followed by 5% formic acid neutralization.

Liquid chromatography with tandem mass spectrometry (LC-MS-MS): The digested protein samples were run through a strong cation exchange column coupled by reverse phase separation to enhance the separation power and dynamic range of analysis [4]. The column was next washed with 95% water, 5% acetonitrile, and 10% formic acid. In series of steps, the column was next attached and analyzed with an Agilent 1100 quaternary pump and a LTQ Orbitrap Velos (*Thermofisher*), with an in-house built electrospray stage, as described previously [5]. Proteins identification and quantitation were analyzed with the Integrated Proteomics Applications (San Diego, CA.). Tandem mass spectra were extracted from raw files using RawExtract (Version 1.9.9) [6], and were searched against Uniprot mus musculus database with reversed sequences using ProLuCID [7]. Peptide candidates were filtered using DTASelect program

(version 2.0). Each separation was performed and clustered independently for all eight samples. Proteins obtained from the different separations were categorized in terms of types and percentage of enrichment.

Protein data profiling: The proteomic data were analyzed based on the spectral count abundance. The p-value for each protein entry was calculated using Microsoft Excel (version 15.4; Table S1). Protein fractions with P value ≤ 0.05 were further analyzed (Table S2). Based on our proteomic comparative analysis, all highly significant protein candidates that were not present (zero spectral count) in non-ciliated *IFT88* knockout cells were categorized as WT vesicles exclusive proteins (Table 3). Similarly, highly significant protein candidates that appeared only in non-ciliated *IFT88*, and not in ciliated control cells, were categorized as proteins exclusively expressed in KO vesicles (Table 4). Proteins that were highly significant and present in both vesicles were tabulated separately and categorized as proteins shared between WT and KO cell-derived vesicles (Table 5). To calculate the fold changes in protein expressions, the averaged spectrum count of each protein in wild-type control samples was divided by the averaged spectrum count of each protein in *IFT88* samples.

Bioinformatics analyses

Gene ontology (GO) analyses: The dataset was analyzed with Ontologizer application (version 2.0) [8]. This application generated a set of interlinked HTML pages that presented three different categories: biological processes, cellular component and molecular function. For more targeted GO analyses for protein function (protein classes), signaling pathways, as well as biological processes, cellular component and molecular function, Panther classification system was used (version 14.0) [9, 10].

Volcano, clustering, Venn diagram and violin plots: When a dataset was not normally distributed or heterogeneous variance was detected, the distributions were normalized via log transformation. Plots

were made using the R project for statistical computing software, (version 3.5.3). Effect sizes included in violin plots were calculated using a Hedge's g with correction for small sample sizes [11].

Network analyses: The protein-protein interaction network was analyzed with Cytoscape software (version 3.3.0). The network interaction was simplified to examine only interaction between POR and CD166 biomarkers. Interaction with both biomarker with ciliary genes was also analyzed. Types of interactions (direct, physical, association and colocalization) were all described in the network. All ciliary genes that interacted with vesicle biomarkers were distinctively highlighted, according to their ciliary sub-compartment localization.

Statistical analyses

All quantifiable data are reported as the mean±standard error of the mean. The homogeneity of variance (homoscedasticity) was verified within each data set. Statistical analysis was performed using ANOVA (analysis of variance) followed by a Bonferroni post hoc test. Power analysis was determined from the coefficient variant. Most of our statistical analyses were performed with GraphPad Prism software (version 7.0). The probability levels *P* values, represent the significant differences, is indicated in each calculated graph. Numbers of experimental replicates and sample sizes are indicated in the figure legends.

Supplemental References

1. Mohieldin, A.M., Haymour, H.S., Lo, S.T., AbouAlaiwi, W.A., Atkinson, K.F., Ward, C.J., Gao, M., Wessely, O., and Nauli, S.M. (2015). Protein composition and movements of membrane swellings associated with primary cilia. Cellular and molecular life sciences : CMLS *72*, 2415-2429.
2. Sherpa, R.T., Pala, R., Mohieldin, A.M., and Nauli, S.M. (2019). Measurement of cytoplasmic and cilioplasmic calcium in a single living cell. Methods Cell Biol *153*, 25-42.
3. Jin, X., Mohieldin, A.M., Muntean, B.S., Green, J.A., Shah, J.V., Mykytyn, K., and Nauli, S.M. (2014). Cilioplasm is a cellular compartment for calcium signaling in response to mechanical and chemical stimuli. Cell Mol Life Sci *71*, 2165-2178.
4. Motoyama, A., Xu, T., Ruse, C.I., Wohlschlegel, J.A., and Yates, J.R., 3rd (2007). Anion and cation mixed-bed ion exchange for enhanced multidimensional separations of peptides and phosphopeptides. Analytical chemistry *79*, 3623-3634.
5. Wolters, D.A., Washburn, M.P., and Yates, J.R., 3rd (2001). An automated multidimensional protein identification technology for shotgun proteomics. Analytical chemistry *73*, 5683-5690.
6. McDonald, W.H., Tabb, D.L., Sadygov, R.G., MacCoss, M.J., Venable, J., Graumann, J., Johnson, J.R., Cociorva, D., and Yates, J.R., 3rd (2004). MS1, MS2, and SQT-three unified, compact, and easily parsed file formats for the storage of shotgun proteomic spectra and identifications. Rapid communications in mass spectrometry : RCM *18*, 2162-2168.
7. Xu, T., Park, S.K., Venable, J.D., Wohlschlegel, J.A., Diedrich, J.K., Cociorva, D., Lu, B., Liao, L., Hewel, J., Han, X., et al. (2015). ProLuCID: An improved SEQUEST-like algorithm with enhanced sensitivity and specificity. Journal of proteomics *129*, 16-24.
8. Robinson, P.N., Wollstein, A., Bohme, U., and Beattie, B. (2004). Ontologizing gene-expression microarray data: characterizing clusters with Gene Ontology. Bioinformatics *20*, 979-981.
9. Mi, H., Muruganujan, A., Huang, X., Ebert, D., Mills, C., Guo, X., and Thomas, P.D. (2019). Protocol Update for large-scale genome and gene function analysis with the PANTHER classification system (v.14.0). Nature protocols *14*, 703-721.
10. Thomas, P.D., Campbell, M.J., Kejariwal, A., Mi, H., Karlak, B., Daverman, R., Diemer, K., Muruganujan, A., and Narechania, A. (2003). PANTHER: a library of protein families and subfamilies indexed by function. Genome research *13*, 2129-2141.
11. Durlak, J.A. (2009). How to select, calculate, and interpret effect sizes. Journal of pediatric psychology *34*, 917-928.

Table S1 Click here to access/download;Table;Table S1.pdf

Table S1. Analysis of protein compositions from isolated WT and KO cell-derived vesicles has revealed 3,444 protein fractions.

Accession	T-test p-value	Spectral count for ciliated EV (WT)				Spectral count for non-ciliated EV (KO)				Description
		Sample1	Sample2	Sample3	Sample4	Sample1	Sample2	Sample3	Sample4	
P16125	0.003457809	0	0	0	0	11	13	12	13	L-lactate dehydrogenase B chain OS=Mus musculus GN=Ldhb PE=1 SV=2
P35441	0.00699971	0	0	0	0	4	7	4	6	Thrombospondin-1 OS=Mus musculus GN=Thbs1 PE=1 SV=1
Q6GQT1	0.011732945	0	0	0	0	4	8	4	6	Alpha-2-macroglobulin-P OS=Mus musculus GN=A2m PE=2 SV=2
Q61171	0.035908159	7	2	8	0	32	28	9	28	Peroxiredoxin-2 OS=Mus musculus GN=Prdx2 PE=1 SV=3
P60766	0.001039897	26	22	22	7	82	63	48	57	Cell division control protein 42 homolog OS=Mus musculus GN=Cdc42 PE=1 SV=2
Q61598	0.017221951	18	14	14	2	55	29	21	39	Rab GDP dissociation inhibitor beta OS=Mus musculus GN=Gdi2 PE=1 SV=1
P26041	0.005194877	111	85	83	28	266	206	188	192	Moesin OS=Mus musculus GN=Msn PE=1 SV=3
Q01853	0.034474749	45	26	44	11	120	98	42	89	Transitional endoplasmic reticulum ATPase OS=Mus musculus GN=Vcp PE=1 SV=4
P10107	0.001412006	90	73	70	26	229	152	129	205	Annexin A1 OS=Mus musculus GN=Anxa1 PE=1 SV=2
Q07797	0.02567394	14	15	14	0	42	38	17	37	Galectin-3-binding protein OS=Mus musculus GN=Lgals3bp PE=1 SV=1
F8WIT2	0.003200513	132	80	104	32	219	180	123	210	Annexin OS=Mus musculus GN=Anxa6 PE=1 SV=1
P18760	0.003724147	67	60	60	17	134	96	86	108	Cofilin-1 OS=Mus musculus GN=Cfl1 PE=1 SV=3
A0A1B0GSX0	0.043669846	28	20	20	14	52	36	31	51	L-lactate dehydrogenase OS=Mus musculus GN=Ldha PE=1 SV=1
P07356	0.015573479	123	89	75	15	208	135	97	156	Annexin A2 OS=Mus musculus GN=Anxa2 PE=1 SV=2
P62259	0.009276622	34	34	35	14	68	54	35	71	14-3-3 protein epsilon OS=Mus musculus GN=Ywhae PE=1 SV=1
A6ZI44	0.02587277	66	75	60	26	133	113	84	106	Fructose-bisphosphate aldolase OS=Mus musculus GN=Aldoa PE=1 SV=1
P57780	0.043192861	46	42	31	7	76	53	40	66	Alpha-actinin-4 OS=Mus musculus GN=Actn4 PE=1 SV=1
P62962	0.018547269	53	57	56	18	73	104	70	89	Profilin-1 OS=Mus musculus GN=Pfn1 PE=1 SV=2
P48036	0.012725186	73	41	42	16	95	67	59	84	Annexin A5 OS=Mus musculus GN=Anxa5 PE=1 SV=1
P17742	0.053917159	52	27	25	0	77	59	30	62	Peptidyl-prolyl cis-trans isomerase A OS=Mus musculus GN=Ppia PE=1 SV=2
Q8VDD5	0.033862598	272	183	227	83	409	303	230	305	Myosin-9 OS=Mus musculus GN=Myh9 PE=1 SV=4
Q9WU78	0.041529698	88	65	57	17	107	83	68	95	Programmed cell death 6-interacting protein OS=Mus musculus GN=Pdcd6ip PE=1 SV=3
P70168	0.01050399	51	38	49	14	63	52	40	58	Importin subunit beta-1 OS=Mus musculus GN=Kpnb1 PE=1 SV=2
Q6R0H7	0.042889642	30	19	25	7	20	17	10	21	Guanine nucleotide-binding protein G(s) subunit alpha isoforms XLas OS=Mus musculus GN=Gnas PE=1 SV=1
Q6PHC1	0.009664254	147	127	136	34	108	84	64	90	Alpha-enolase OS=Mus musculus GN=Eno1 PE=1 SV=1
P80318	0.039641284	161	123	144	68	99	88	82	103	T-complex protein 1 subunit gamma OS=Mus musculus GN=Cct3 PE=1 SV=1
P63101	0.019829529	78	75	78	35	54	31	42	70	14-3-3 protein zeta/delta OS=Mus musculus GN=Ywhaz PE=1 SV=1
Q5SXR6	0.003343502	414	294	302	96	189	174	129	202	Clathrin heavy chain OS=Mus musculus GN=Cltc PE=1 SV=1
P40142	0.006584647	63	52	60	16	38	39	17	23	Transketolase OS=Mus musculus GN=Tkt PE=1 SV=1
Q9CQV8	0.006230055	73	54	58	34	29	21	24	58	14-3-3 protein beta/alpha OS=Mus musculus GN=Ywhab PE=1 SV=3
F6YY69	0.003182217	89	81	76	40	23	26	29	67	14-3-3 protein theta (Fragment) OS=Mus musculus GN=Ywhaq PE=1 SV=1
P80314	0.001582106	217	145	230	79	76	70	68	120	T-complex protein 1 subunit beta OS=Mus musculus GN=Cct2 PE=1 SV=4
P62874	0.00790914	108	89	75	36	45	32	32	44	Guanine nucleotide-binding protein G(I)/G(S)/G(T) subunit beta-1 OS=Mus musculus GN=Gnb1 PE=1 SV=3
Q61739	0.001399553	36	26	26	9	6	6	7	5	Integrin alpha-6 OS=Mus musculus GN=Itga6 PE=1 SV=3
Q62351	0.000290724	106	72	82	24	5	5	4	10	Transferrin receptor protein 1 OS=Mus musculus GN=Tfrc PE=1 SV=1
P62821	0.07497188	0	0	0	0	2	3	0	3	Ras-related protein Rab-1A OS=Mus musculus GN=Rab1A PE=1 SV=3
O08917	0.189284287	0	0	0	0	5	0	0	6	Flotillin-1 OS=Mus musculus GN=Flot1 PE=1 SV=1
H3BKG0	0.391002219	0	0	0	0	0	0	0	4	Caveolin-1 OS=Mus musculus GN=Cav1 PE=1 SV=1
B7FAV1	0.004455195	99	73	99	20	303	195	138	211	Filamin, alpha (Fragment) OS=Mus musculus GN=Flna PE=1 SV=1
D3Z3I8	0.36236249	225	178	220	75	367	0	0	244	Elongation factor 1-alpha 1 (Fragment) OS=Mus musculus GN=Eef1a1 PE=1 SV=1
P84078	0.066086698	20	12	9	4	22	18	13	25	ADP-ribosylation factor 1 OS=Mus musculus GN=Arf1 PE=1 SV=2
Q7TT50	0.122946822	6	3	0	0	6	6	8	11	Serine/threonine-protein kinase MRCK beta OS=Mus musculus GN=Cdc42bpb PE=1 SV=2
P63001	0.102102581	49	28	42	10	47	53	58	60	Ras-related C3 botulinum toxin substrate 1 OS=Mus musculus GN=Rac1 PE=1 SV=1
P35700	0.190724786	53	33	51	7	96	50	31	60	Peroxiredoxin-1 OS=Mus musculus GN=Prdx1 PE=1 SV=1
O08992	0.172667576	17	14	14	4	24	16	12	28	Syntenin-1 OS=Mus musculus GN=Sdcbp PE=1 SV=1
Q9WV91	0.079384173	34	29	28	6	38	43	30	47	Prostaglandin F2 receptor negative regulator OS=Mus musculus GN=Ptgfrn PE=1 SV=2
P52480	0.267546327	301	216	239	80	488	246	166	352	Pyruvate kinase PKM OS=Mus musculus GN=Pkm PE=1 SV=4
P10852	0.302064676	35	26	19	4	33	27	25	38	4F2 cell-surface antigen heavy chain OS=Mus musculus GN=Slc3a2 PE=1 SV=1
P09411	0.344982189	184	141	179	76	210	223	191	223	Phosphoglycerate kinase 1 OS=Mus musculus GN=Pgk1 PE=1 SV=4
P41731	0.121361253	0	0	3	0	3	6	4	0	CD63 antigen OS=Mus musculus GN=Cd63 PE=1 SV=2
Q02053	0.120439965	120	86	102	22	169	118	72	116	Ubiquitin-like modifier-activating enzyme 1 OS=Mus musculus GN=Uba1 PE=1 SV=1
A0A140LJL0	0.717034625	7	9	7	0	12	8	0	13	CD81 antigen (Fragment) OS=Mus musculus GN=Cd81 PE=1 SV=1
Q01279	0.127624755	3	4	0	0	4	5	5	6	Epidermal growth factor receptor OS=Mus musculus GN=Egfr PE=1 SV=1
P62806	0.226080574	288	226	234	72	327	315	284	232	Histone H4 OS=Mus musculus GN=Hist1h4a PE=1 SV=2
Q99JI6	0.146372189	21	14	16	5	23	20	18	18	Ras-related protein Rap-1b OS=Mus musculus GN=Rap1b PE=1 SV=2
Q3V117	0.603663424	77	46	67	26	112	66	40	85	ATP-citrate synthase OS=Mus musculus GN=Acly PE=1 SV=1
A0A0A6YXF6	0.579607824	86	71	58	21	52	67	83	119	Transforming protein RhoA (Fragment) OS=Mus musculus GN=Rhoa PE=1 SV=1
Q9D091	0.140626898	10	4	0	0	10	7	10	11	GTPase NRas OS=Mus musculus GN=Nras PE=1 SV=1
Q61696	0.199114225	87	76	81	39	0	0	95	0	Heat shock 70 kDa protein 1A OS=Mus musculus GN=Hspa1a PE=1 SV=2
P07724	0.785465479	0	4	4	13	7	8	11	11	Serum albumin OS=Mus musculus GN=Alb PE=1 SV=3
P54116	0.081656295	3	9	10	2	5	11	8	7	Erythrocyte band 7 integral membrane protein OS=Mus musculus GN=Stom PE=1 SV=3
E9Q1F2	0.528034416	473	343	350	95	543	327	269	465	Actin, cytoplasmic 1 OS=Mus musculus GN=Actb PE=1 SV=1
P97429	0.47432758	68	43	52	14	76	42	43	57	Annexin A4 OS=Mus musculus GN=Anxa4 PE=1 SV=4
E9Q3X0	0.735196524	13	9	15	2	8	15	8	17	Major vault protein OS=Mus musculus GN=Mvp PE=1 SV=1
P26040	0.599019696	70	61	65	18	63	68	64	66	Ezrin OS=Mus musculus GN=Ezr PE=1 SV=3
A0A0U1RNJ1	0.832870111	304	204	263	79	315	246	178	280	Fatty acid synthase OS=Mus musculus GN=Fasn PE=1 SV=1
P63017	0.504313118	348	311	313	148	418	338	237	324	Heat shock cognate 71 kDa protein OS=Mus musculus GN=Hspa8 PE=1 SV=1
A0A0A0MQF6	0.508713099	137	109	132	59	182	134	74	112	Glyceraldehyde-3-phosphate dehydrogenase OS=Mus musculus GN=Gapdh PE=1 SV=1
P17751	0.918861635	122	96	106	20	84	92	98	108	Triosephosphate isomerase OS=Mus musculus GN=Tpi1 PE=1 SV=4
P21956	0.315848493	230	171	193	80	218	198	145	187	Lactadherin OS=Mus musculus GN=Mfge8 PE=1 SV=3
P80316	0.761804557	129	77	99	22	115	85	80	81	T-complex protein 1 subunit epsilon OS=Mus musculus GN=Cct5 PE=1 SV=1
P63260	0.305366673	1196	955	993	449	1055	887	881	1094	Actin, cytoplasmic 2 OS=Mus musculus GN=Actg1 PE=1 SV=1
O35598	0.430451625	4	5	7	0	8	7	3	5	Disintegrin and metalloproteinase domain-containing protein 10 OS=Mus musculus GN=Adam10 PE=1 SV=2
P08752	0.420378363	56	39	40	11	52	33	25	47	Guanine nucleotide-binding protein G(i) subunit alpha-2 OS=Mus musculus GN=Gnai2 PE=1 SV=5
Q9Z1Q5	0.266327101	47	32	37	8	43	31	27	30	Chloride intracellular channel protein 1 OS=Mus musculus GN=Clic1 PE=1 SV=3
P09055	0.144682836	94	85	90	29	89	73	71	78	Integrin beta-1 OS=Mus musculus GN=Itgb1 PE=1 SV=1
P68369	0.458594742	223	150	153	34	188	143	77	165	Tubulin alpha-1A chain OS=Mus musculus GN=Tuba1a PE=1 SV=1
P05213	0.460832697	225	151	152	34	190	143	76	166	Tubulin alpha-1B chain OS=Mus musculus GN=Tuba1b PE=1 SV=2
P20029	0.37501981	34	20	26	8	31	28	8	23	78 kDa glucose-regulated protein OS=Mus musculus GN=Hspa5 PE=1 SV=3
P68373	0.393710732	220	149	149	34	186	135	77	160	Tubulin alpha-1C chain OS=Mus musculus GN=Tuba1c PE=1 SV=1
D3Z1M1	0.70254034	59	43	49	9	35	38	36	52	Guanine nucleotide-binding protein G(I)/G(S)/G(T) subunit beta-2 (Fragment) OS=Mus musculus GN=Gnb2 PE=1 SV=1
P68510	0.230994514	22	18	26	14	16	18	20	26	14-3-3 protein eta OS=Mus musculus GN=Ywhah PE=1 SV=2
Q61187	0.604299512	4	4	0	0	0	0	0	4	Tumor susceptibility gene 101 protein OS=Mus musculus GN=Tsg101 PE=1 SV=2
E9Q0C3	0.1010874	94	93	104	43	98	82	59	88	Heat shock protein HSP 90-beta (Fragment) OS=Mus musculus GN=Hsp90ab1 PE=1 SV=1
P07901	0.088575903	233	174	225	50	194	159	124	177	Heat shock protein HSP 90-alpha OS=Mus musculus GN=Hsp90aa1 PE=1 SV=4
J3QP71	0.341094864	22	20	17	5	12	17	15	17	Basigin (Fragment) OS=Mus musculus GN=Bsg PE=1 SV=1
F2Z483	0.273409548	30	17	23	7	13	20	17	19	T-complex protein 1 subunit alpha OS=Mus musculus GN=Tcp1 PE=1 SV=1
Q9EQP2	0.623047595	30	13	18	2	18	10	8	20	EH domain-containing protein 4 OS=Mus musculus GN=Ehd4 PE=1 SV=1
P17047	0.72245146	9	0	4	0	7	5	0	5	Lysosome-associated membrane glycoprotein 2 OS=Mus musculus GN=Lamp2 PE=1 SV=2
Q8VDN2	0.114091839	125	108	113	23	98	74	50	88	Sodium/potassium-transporting ATPase subunit alpha-1 OS=Mus musculus GN=Atp1a1 PE=1 SV=1
P55258	0.267496862	7	0	7	0	4	5	7	7	Ras-related protein Rab-8A OS=Mus musculus GN=Rab8a PE=1 SV=2
P51150	0.941688958	32	19	18	0	16	14	10	26	Ras-related protein Rab-7a OS=Mus musculus GN=Rab7a PE=1 SV=2
Q91V41	0.791702711	16	11	9	0	11	9	5	8	Ras-related protein Rab-14 OS=Mus musculus GN=Rab14 PE=1 SV=3
P53986	0.107024019	16	12	16	10	8	7	8	6	Monocarboxylate transporter 1 OS=Mus musculus GN=Slc16a1 PE=1 SV=1
P35278	0.409685858	8	8	13	0	5	3	0	7	Ras-related protein Rab-5C OS=Mus musculus GN=Rab5c PE=1 SV=2
Q9QZF2	0.103596017	13	15	11	0	3	3	0	2	Glypican-1 OS=Mus musculus GN=Gpc1 PE=1 SV=1
A0A0U1RQ49	0.065348584	5	4	3	0	0	0	0	0	Hsp70-binding protein 1 (Fragment) OS=Mus musculus GN=Hspbp1 PE=1 SV=1
P40240	0.207614406	4	4	0	6	0	0	0	0	CD9 antigen OS=Mus musculus GN=Cd9 PE=1 SV=2
Reverse_sp|P58252|EF2_MOUSE	0.391002219	0	2	0	0	0	0	0	0	Elongation factor 2 OS=Mus musculus GN=Eef2 PE=1 SV=2
Q9WVF5	0.391002219	0	4	0	0	0	0	0	0	Epidermal growth factor receptor OS=Mus musculus GN=Egfr PE=1 SV=1
Q61490	1.9445E-05	0	0	0	0	29	24	17	27	CD166 antigen OS=Mus musculus GN=Alcam PE=1 SV=3
E9Q3Q6	1.9445E-05	0	0	0	0	29	24	17	27	CD166 antigen OS=Mus musculus GN=Alcam PE=1 SV=1
E9Q4G8	1.9445E-05	0	0	0	0	29	24	17	27	CD166 antigen OS=Mus musculus GN=Alcam PE=1 SV=1
Q5SUW3	7.11741E-05	0	0	0	0	6	5	4	5	Growth factor receptor-bound protein 10 OS=Mus musculus GN=Grb10 PE=1 SV=1
Q60760	7.11741E-05	0	0	0	0	6	5	4	5	Growth factor receptor-bound protein 10 OS=Mus musculus GN=Grb10 PE=1 SV=2
P26618	7.38176E-05	0	0	0	0	13	10	7	10	Platelet-derived growth factor receptor alpha OS=Mus musculus GN=Pdgfra PE=1 SV=3
O35295	9.88794E-05	0	0	0	0	7	6	5	7	Transcriptional activator protein Pur-beta OS=Mus musculus GN=Purb PE=1 SV=3
O35474	0.000105736	0	0	0	0	15	11	10	13	EGF-like repeat and discoidin I-like domain-containing protein 3 OS=Mus musculus GN=Edil3 PE=1 SV=2
Q8C4U8	0.000105736	0	0	0	0	15	11	10	13	EGF-like repeat and discoidin I-like domain-containing protein 3 OS=Mus musculus GN=Edil3 PE=1 SV=1
Q80YX1	0.00012499	0	0	0	0	71	62	49	75	Tenascin OS=Mus musculus GN=Tnc PE=1 SV=1
Q04857	0.000207749	0	0	0	0	23	19	14	17	Collagen alpha-1(VI) chain OS=Mus musculus GN=Col6a1 PE=1 SV=1
Q6P1J1	0.000257504	0	0	0	0	19	18	12	16	Crmp1 protein OS=Mus musculus GN=Crmp1 PE=1 SV=1
P51655	0.000392305	0	0	0	0	35	22	20	25	Glypican-4 OS=Mus musculus GN=Gpc4 PE=1 SV=2
D3Z1J5	0.000508638	0	0	0	0	4	3	3	4	Palladin (Fragment) OS=Mus musculus GN=Palld PE=1 SV=1
P62897	0.000546062	0	0	0	0	17	13	8	12	Cytochrome c, somatic OS=Mus musculus GN=Cycs PE=1 SV=2
P28653	0.000574134	0	0	0	0	35	21	19	31	Biglycan OS=Mus musculus GN=Bgn PE=1 SV=1
Q9WTK5	0.000637921	0	0	0	0	4	3	2	4	Nuclear factor NF-kappa-B p100 subunit OS=Mus musculus GN=Nfkb2 PE=1 SV=1
Q62009	0.000644387	0	0	0	0	37	24	17	25	Periostin OS=Mus musculus GN=Postn PE=1 SV=2
P97927	0.000654838	0	0	0	0	34	29	17	34	Laminin subunit alpha-4 OS=Mus musculus GN=Lama4 PE=1 SV=2
E9QA16	0.000749939	0	0	0	0	29	24	22	23	Protein Cald1 OS=Mus musculus GN=Cald1 PE=1 SV=1
Q8VCQ8	0.000808196	0	0	0	0	29	23	22	23	Caldesmon 1 OS=Mus musculus GN=Cald1 PE=1 SV=1
A0A0G2JGD2	0.000844875	0	0	0	0	5	5	3	4	Protein S100-A4 (Fragment) OS=Mus musculus GN=S100a4 PE=1 SV=1
P07091	0.000844875	0	0	0	0	5	5	3	4	Protein S100-A4 OS=Mus musculus GN=S100a4 PE=1 SV=1
P37889	0.000948621	0	0	0	0	86	61	36	62	Fibulin-2 OS=Mus musculus GN=Fbln2 PE=1 SV=2
A0A0J9YUE9	0.001171617	0	0	0	0	10	6	6	10	Dynamin-1 OS=Mus musculus GN=Dnm1 PE=1 SV=1
P54728	0.001176835	0	0	0	0	8	7	7	8	UV excision repair protein RAD23 homolog B OS=Mus musculus GN=Rad23b PE=1 SV=2
G3XA35	0.001386952	0	0	0	0	12	9	6	7	MCG116562, isoform CRA_a OS=Mus musculus GN=Vcan PE=1 SV=1
E9PYH0	0.001386952	0	0	0	0	12	9	6	7	Versican core protein OS=Mus musculus GN=Vcan PE=1 SV=1
P10518	0.001406176	0	0	0	0	8	9	7	8	Delta-aminolevulinic acid dehydratase OS=Mus musculus GN=Alad PE=1 SV=1
Q6P069	0.00143027	0	0	0	0	12	11	8	8	Sorcin OS=Mus musculus GN=Sri PE=1 SV=1
P18872	0.001480543	0	0	0	0	26	22	13	29	Guanine nucleotide-binding protein G(o) subunit alpha OS=Mus musculus GN=Gnao1 PE=1 SV=3
Q8CG14	0.001603835	0	0	0	0	12	12	6	10	Complement C1s-A subcomponent OS=Mus musculus GN=C1sa PE=2 SV=2
E9Q6C2	0.001603835	0	0	0	0	12	12	6	10	Protein C1s1 OS=Mus musculus GN=C1s1 PE=1 SV=1
D3Z2M7	0.001614518	0	0	0	0	26	19	11	25	Guanine nucleotide-binding protein G(o) subunit alpha (Fragment) OS=Mus musculus GN=Gnao1 PE=1 SV=1
O88322	0.001712844	0	0	0	0	92	66	35	71	Nidogen-2 OS=Mus musculus GN=Nid2 PE=1 SV=2
O70326	0.001787627	0	0	0	0	5	3	3	3	Gremlin-1 OS=Mus musculus GN=Grem1 PE=2 SV=1
Q62523	0.002084986	0	0	0	0	6	5	3	7	Zyxin OS=Mus musculus GN=Zyx PE=1 SV=2
Q7TQE2	0.002084986	0	0	0	0	6	5	3	7	Zyx protein OS=Mus musculus GN=Zyx PE=1 SV=1
Q01149	0.002120259	0	0	0	0	42	39	25	26	Collagen alpha-2(I) chain OS=Mus musculus GN=Col1a2 PE=1 SV=2
H3BL26	0.002249225	0	0	0	0	6	5	4	8	Tetraspanin-6 (Fragment) OS=Mus musculus GN=Tspan6 PE=1 SV=8
O70401	0.002249225	0	0	0	0	6	5	4	8	Tetraspanin-6 OS=Mus musculus GN=Tspan6 PE=1 SV=1
Q99L96	0.002249225	0	0	0	0	6	5	4	8	Tetraspanin OS=Mus musculus GN=Tspan6 PE=1 SV=1
P28654	0.002367549	0	0	0	0	77	101	72	91	Decorin OS=Mus musculus GN=Dcn PE=1 SV=1
Q62000	0.002404536	0	0	0	0	14	9	5	10	Mimecan OS=Mus musculus GN=Ogn PE=1 SV=1
Q61553	0.002753759	0	0	0	0	85	107	89	111	Fascin OS=Mus musculus GN=Fscn1 PE=1 SV=4
G3UXG7	0.002886723	0	0	0	0	5	5	5	5	Casein kinase II subunit beta OS=Mus musculus GN=Csnk2b PE=1 SV=1
P67871	0.002886723	0	0	0	0	5	5	5	5	Casein kinase II subunit beta OS=Mus musculus GN=Csnk2b PE=1 SV=1
D3Z4A4	0.003269405	0	0	0	0	27	18	9	20	Peroxiredoxin-2 (Fragment) OS=Mus musculus GN=Prdx2 PE=1 SV=8
D3YX76	0.003339675	0	0	0	0	4	5	3	6	Glutathione S-transferase OS=Mus musculus GN=Gstm2 PE=1 SV=1
P15626	0.003339675	0	0	0	0	4	5	3	6	Glutathione S-transferase Mu 2 OS=Mus musculus GN=Gstm2 PE=1 SV=2
A0A087WS16	0.003504367	0	0	0	0	25	18	9	15	Protein Col6a3 OS=Mus musculus GN=Col6a3 PE=1 SV=1
Q9DCC4	0.003573486	0	0	0	0	5	3	4	4	Pyrroline-5-carboxylate reductase 3 OS=Mus musculus GN=Pycrl PE=1 SV=2
Q5SS83	0.003604865	0	0	0	0	8	4	5	5	Flotillin 2, isoform CRA_a OS=Mus musculus GN=Flot2 PE=1 SV=1
Q60634	0.003604865	0	0	0	0	8	4	5	5	Flotillin-2 OS=Mus musculus GN=Flot2 PE=1 SV=2
A0A067XG53	0.003768895	0	0	0	0	8	8	6	5	Peripheral plasma membrane protein CASK (Fragment) OS=Mus musculus GN=Cask PE=1 SV=1
O70589	0.003768895	0	0	0	0	8	8	6	5	Peripheral plasma membrane protein CASK OS=Mus musculus GN=Cask PE=1 SV=2
P11152	0.004017591	0	0	0	0	19	8	10	13	Lipoprotein lipase OS=Mus musculus GN=Lpl PE=1 SV=3
B1B1E2	0.004292091	0	0	0	0	4	6	4	5	Latent-transforming growth factor beta-binding protein 1 (Fragment) OS=Mus musculus GN=Ltbp1 PE=1 SV=8
Q8CG19	0.004292091	0	0	0	0	4	6	4	5	Latent-transforming growth factor beta-binding protein 1 OS=Mus musculus GN=Ltbp1 PE=1 SV=2
Q9ET54	0.00437601	0	0	0	0	6	8	7	8	Palladin OS=Mus musculus GN=Palld PE=1 SV=2

P48428	0.004524911	0	0	0	0	11	5	5	6	Tubulin-specific chaperone A OS=Mus musculus GN=Tbca PE=1 SV=3
E9Q2A0	0.004609696	0	0	0	0	8	7	5	4	EGF-containing fibulin-like extracellular matrix protein 2 (Fragment) OS=Mus musculus GN=Efemp2 PE=1 SV=1
Q99K41	0.005109234	0	0	0	0	26	32	15	21	EMILIN-1 OS=Mus musculus GN=Emilin1 PE=1 SV=1
E9PWQ3	0.005148804	0	0	0	0	29	22	11	15	Protein Col6a3 OS=Mus musculus GN=Col6a3 PE=1 SV=2
O70433	0.005361581	0	0	0	0	5	7	5	9	Four and a half LIM domains protein 2 OS=Mus musculus GN=Fhl2 PE=1 SV=1
E9PYB0	0.005637731	0	0	0	0	7	4	6	6	Protein Ahnak2 (Fragment) OS=Mus musculus GN=Ahnak2 PE=1 SV=8
Q9WVJ9	0.005876575	0	0	0	0	12	10	5	6	EGF-containing fibulin-like extracellular matrix protein 2 OS=Mus musculus GN=Efemp2 PE=1 SV=1
G5E8D6	0.005876575	0	0	0	0	12	10	5	6	EGF-containing fibulin-like extracellular matrix protein 2 OS=Mus musculus GN=Efemp2 PE=1 SV=1
P10493	0.005996932	0	0	0	0	126	87	36	89	Nidogen-1 OS=Mus musculus GN=Nid1 PE=1 SV=2
Q9JJU8	0.006089453	0	0	0	0	8	8	9	8	SH3 domain-binding glutamic acid-rich-like protein OS=Mus musculus GN=Sh3bgrl PE=1 SV=1
Q62059	0.006315549	0	0	0	0	11	9	5	5	Versican core protein OS=Mus musculus GN=Vcan PE=1 SV=2
Q61081	0.006333512	0	0	0	0	5	2	2	3	Hsp90 co-chaperone Cdc37 OS=Mus musculus GN=Cdc37 PE=1 SV=1
O08603	0.006606666	0	0	0	0	8	7	6	4	Retinoic acid early-inducible protein 1-beta OS=Mus musculus GN=Raet1b PE=1 SV=1
P53996	0.006827793	0	0	0	0	22	13	12	9	Cellular nucleic acid-binding protein OS=Mus musculus GN=Cnbp PE=1 SV=2
Q60866	0.006963962	0	0	0	0	3	4	4	5	Phosphotriesterase-related protein OS=Mus musculus GN=Pter PE=1 SV=1
A2AUR3	0.006963962	0	0	0	0	3	4	4	5	Phosphotriesterase-related protein (Fragment) OS=Mus musculus GN=Pter PE=1 SV=2
Q80YQ1	0.00699971	0	0	0	0	4	7	4	6	Thrombospondin 1 OS=Mus musculus GN=Thbs1 PE=1 SV=1
D3YTP0	0.007130451	0	0	0	0	5	7	4	9	Metalloreductase STEAP3 (Fragment) OS=Mus musculus GN=Steap3 PE=1 SV=8
E9QN92	0.007130451	0	0	0	0	5	7	4	9	Metalloreductase STEAP3 OS=Mus musculus GN=Steap3 PE=1 SV=1
Q8CI59	0.007130451	0	0	0	0	5	7	4	9	Metalloreductase STEAP3 OS=Mus musculus GN=Steap3 PE=1 SV=1
A0A0R4J1G9	0.007130451	0	0	0	0	5	7	4	9	Metalloreductase STEAP3 OS=Mus musculus GN=Steap3 PE=1 SV=1
Q9CWS0	0.007276445	0	0	0	0	5	3	4	3	N(G),N(G)-dimethylarginine dimethylaminohydrolase 1 OS=Mus musculus GN=Ddah1 PE=1 SV=3
Q64314	0.007325832	0	0	0	0	8	10	5	13	Hematopoietic progenitor cell antigen CD34 OS=Mus musculus GN=Cd34 PE=1 SV=1
P01027	0.007522037	0	0	0	0	12	17	11	10	Complement C3 OS=Mus musculus GN=C3 PE=1 SV=3
Q3U687	0.007620967	0	0	0	0	5	8	6	6	Protein Ifit1bl2 OS=Mus musculus GN=Ifit1bl2 PE=1 SV=1
P70290	0.009163409	0	0	0	0	13	18	7	12	55 kDa erythrocyte membrane protein OS=Mus musculus GN=Mpp1 PE=1 SV=1
A2AN84	0.009163409	0	0	0	0	13	18	7	12	55 kDa erythrocyte membrane protein OS=Mus musculus GN=Mpp1 PE=1 SV=1
Q03350	0.009228339	0	0	0	0	14	12	6	6	Thrombospondin-2 OS=Mus musculus GN=Thbs2 PE=1 SV=2
Q64449	0.009564109	0	0	0	0	7	9	5	13	C-type mannose receptor 2 OS=Mus musculus GN=Mrc2 PE=1 SV=3
P14428	0.009610074	0	0	0	0	6	5	4	10	H-2 class I histocompatibility antigen, K-Q alpha chain (Fragment) OS=Mus musculus GN=H2-K1 PE=1 SV=1
G3UXU2	0.010593758	0	0	0	0	3	3	4	5	Casein kinase II subunit beta (Fragment) OS=Mus musculus GN=Csnk2b PE=1 SV=2
P61622	0.010593758	0	0	0	0	3	3	4	5	Integrin alpha-11 OS=Mus musculus GN=Itga11 PE=1 SV=1
A0A0B4J1F0	0.010593758	0	0	0	0	3	3	4	5	Integrin alpha-11 OS=Mus musculus GN=Itga11 PE=1 SV=1
Q8K2A1	0.010609968	0	0	0	0	6	4	3	2	PTB domain-containing engulfment adapter protein 1 OS=Mus musculus GN=Gulp1 PE=1 SV=1
E0CXC5	0.010609968	0	0	0	0	6	4	3	2	PTB domain-containing engulfment adapter protein 1 OS=Mus musculus GN=Gulp1 PE=1 SV=1
O35646	0.01093572	0	0	0	0	3	5	4	7	Calpain-6 OS=Mus musculus GN=Capn6 PE=1 SV=2
Q9D0F9	0.011234897	0	0	0	0	8	6	5	13	Phosphoglucomutase-1 OS=Mus musculus GN=Pgm1 PE=1 SV=4
E9QN12	0.011495961	0	0	0	0	8	3	6	6	Platelet-derived growth factor receptor beta OS=Mus musculus GN=Pdgfrb PE=1 SV=1
P05622	0.011495961	0	0	0	0	8	3	6	6	Platelet-derived growth factor receptor beta OS=Mus musculus GN=Pdgfrb PE=1 SV=1
E9QPE2	0.011495961	0	0	0	0	8	3	6	6	Platelet-derived growth factor receptor beta OS=Mus musculus GN=Pdgfrb PE=1 SV=1
B2RUE8	0.011944388	0	0	0	0	3	5	5	6	Map4k4 protein OS=Mus musculus GN=Map4k4 PE=1 SV=1
A0A0A6YWM8	0.011944388	0	0	0	0	3	5	5	6	Mitogen-activated protein kinase kinase kinase kinase 4 OS=Mus musculus GN=Map4k4 PE=1 SV=1
P97820	0.011961137	0	0	0	0	3	4	4	3	Mitogen-activated protein kinase kinase kinase kinase 4 OS=Mus musculus GN=Map4k4 PE=1 SV=1
F7DBB3	0.011973526	0	0	0	0	7	4	7	6	Protein Ahnak2 (Fragment) OS=Mus musculus GN=Ahnak2 PE=1 SV=1
P24549	0.01207989	0	0	0	0	2	4	2	4	Retinal dehydrogenase 1 OS=Mus musculus GN=Aldh1a1 PE=1 SV=5
P62965	0.013542122	0	0	0	0	4	9	5	7	Cellular retinoic acid-binding protein 1 OS=Mus musculus GN=Crabp1 PE=1 SV=2
P38647	0.013733466	0	0	0	0	7	5	2	3	Stress-70 protein, mitochondrial OS=Mus musculus GN=Hspa9 PE=1 SV=3
E9PWE8	0.013936138	0	0	0	0	39	29	8	29	Dihydropyrimidinase-related protein 3 OS=Mus musculus GN=Dpysl3 PE=1 SV=1
Q9D281	0.014866485	0	0	0	0	8	10	5	4	Protein Noxp20 OS=Mus musculus GN=Fam114a1 PE=1 SV=1
P11087	0.015241163	0	0	0	0	43	36	13	19	Collagen alpha-1(I) chain OS=Mus musculus GN=Col1a1 PE=1 SV=4
Q08093	0.015427758	0	0	0	0	7	5	6	3	Calponin-2 OS=Mus musculus GN=Cnn2 PE=1 SV=1
Q62188	0.017362145	0	0	0	0	39	29	7	29	Dihydropyrimidinase-related protein 3 OS=Mus musculus GN=Dpysl3 PE=1 SV=1
Q99K51	0.018263529	0	0	0	0	3	7	6	6	Plastin-3 OS=Mus musculus GN=Pls3 PE=1 SV=3
B1AX58	0.018263529	0	0	0	0	3	7	6	6	Plastin-3 OS=Mus musculus GN=Pls3 PE=1 SV=1
A0A1C7CYV0	0.018263529	0	0	0	0	3	7	6	6	Plastin-3 (Fragment) OS=Mus musculus GN=Pls3 PE=1 SV=1
D3Z7D5	0.019535993	0	0	0	0	8	5	2	3	Collagen alpha-2(VI) chain OS=Mus musculus GN=Col6a2 PE=1 SV=1
Q8C8U0	0.021148077	0	0	0	0	22	10	4	14	Liprin-beta-1 OS=Mus musculus GN=Ppfibp1 PE=1 SV=3
A0A0G2JGZ5	0.021633448	0	0	0	0	12	6	2	8	PDZ and LIM domain protein 5 (Fragment) OS=Mus musculus GN=Pdlim5 PE=1 SV=1
Q8BH35	0.024283172	0	0	0	0	4	5	7	5	Complement component C8 beta chain OS=Mus musculus GN=C8b PE=1 SV=1
D9J2Z9	0.026008571	0	0	0	0	13	6	2	8	ENH isoform 1b OS=Mus musculus GN=Pdlim5 PE=1 SV=1
Q8CI51	0.026008571	0	0	0	0	13	6	2	8	PDZ and LIM domain protein 5 OS=Mus musculus GN=Pdlim5 PE=1 SV=4
D9J301	0.026008571	0	0	0	0	13	6	2	8	ENH isoform 1d OS=Mus musculus GN=Pdlim5 PE=1 SV=1
D9J300	0.026008571	0	0	0	0	13	6	2	8	ENH isoform 1c OS=Mus musculus GN=Pdlim5 PE=1 SV=1
D9J302	0.026008571	0	0	0	0	13	6	2	8	ENH isoform 1e OS=Mus musculus GN=Pdlim5 PE=1 SV=1
E9Q1S3	0.026048044	0	0	0	0	5	12	4	9	Protein transport protein Sec23A OS=Mus musculus GN=Sec23a PE=1 SV=1
Q01405	0.026048044	0	0	0	0	5	12	4	9	Protein transport protein Sec23A OS=Mus musculus GN=Sec23a PE=1 SV=2
P01942	0.028797929	0	0	0	0	3	8	4	4	Hemoglobin subunit alpha OS=Mus musculus GN=Hba PE=1 SV=2
Q91VB8	0.028797929	0	0	0	0	3	8	4	4	Alpha globin 1 OS=Mus musculus GN=haemaglobin alpha 2 PE=1 SV=1
Q61205	0.032542586	0	0	0	0	2	7	4	4	Platelet-activating factor acetylhydrolase IB subunit gamma OS=Mus musculus GN=Pafah1b3 PE=1 SV=1
D3Z7E6	0.032542586	0	0	0	0	2	7	4	4	Platelet-activating factor acetylhydrolase IB subunit gamma OS=Mus musculus GN=Pafah1b3 PE=1 SV=1
Q9CPN8	0.035556315	0	0	0	0	13	14	3	6	Insulin-like growth factor 2 mRNA-binding protein 3 OS=Mus musculus GN=Igf2bp3 PE=1 SV=1
A0A0G2JEJ0	0.036259822	0	0	0	0	12	4	2	6	PDZ and LIM domain protein 5 (Fragment) OS=Mus musculus GN=Pdlim5 PE=1 SV=1
P33434	0.036569605	0	0	0	0	4	9	3	4	72 kDa type IV collagenase OS=Mus musculus GN=Mmp2 PE=1 SV=1
Q9CQ60	0.040112916	0	0	0	0	14	9	2	5	6-phosphogluconolactonase OS=Mus musculus GN=Pgls PE=1 SV=1
O08604	0.058169473	0	0	0	0	7	5	4	0	Retinoic acid early-inducible protein 1-gamma OS=Mus musculus GN=Raet1c PE=1 SV=1
Q91V35	0.058492489	0	0	0	0	3	0	2	3	Receptor-type tyrosine-protein phosphatase OS=Mus musculus GN=Ptpra PE=1 SV=1
Q9D8U8	0.058801998	0	0	0	0	4	3	0	3	Sorting nexin-5 OS=Mus musculus GN=Snx5 PE=1 SV=1
Q3TT92	0.058919844	0	0	0	0	39	29	0	29	Dihydropyrimidinase-related protein 3 OS=Mus musculus GN=Dpysl3 PE=1 SV=1
Q60716	0.059371676	0	0	0	0	4	3	2	0	Prolyl 4-hydroxylase subunit alpha-2 OS=Mus musculus GN=P4ha2 PE=1 SV=1
Q5SX75	0.059371676	0	0	0	0	4	3	2	0	Procollagen-proline, 2-oxoglutarate 4-dioxygenase (Proline 4-hydroxylase), alpha II polypeptide, isoform CRA_f OS=Mus musculus GN=P4ha2 PE=1 SV=1
Q61391	0.059404406	0	0	0	0	4	3	0	4	Neprilysin OS=Mus musculus GN=Mme PE=1 SV=3
F6X8L5	0.059605792	0	0	0	0	7	5	0	5	6-phosphogluconolactonase (Fragment) OS=Mus musculus GN=Pgls PE=1 SV=1
H3BJQ7	0.060121148	0	0	0	0	6	6	0	6	Peroxiredoxin-5, mitochondrial OS=Mus musculus GN=Prdx5 PE=1 SV=1
P99029	0.060121148	0	0	0	0	6	6	0	6	Peroxiredoxin-5, mitochondrial OS=Mus musculus GN=Prdx5 PE=1 SV=2
O70493	0.060121148	0	0	0	0	4	4	0	4	Sorting nexin-12 OS=Mus musculus GN=Snx12 PE=1 SV=1
Q3TGS7	0.060121148	0	0	0	0	4	4	0	4	Putative uncharacterized protein OS=Mus musculus GN=Snx12 PE=1 SV=1
Q6ZWQ5	0.060121148	0	0	0	0	4	4	0	4	Putative uncharacterized protein OS=Mus musculus GN=Snx12 PE=1 SV=1
Q3V2H3	0.060121148	0	0	0	0	4	4	0	4	Putative uncharacterized protein OS=Mus musculus GN=Snx12 PE=1 SV=1
O54724	0.061611669	0	0	0	0	7	7	0	6	Polymerase I and transcript release factor OS=Mus musculus GN=Ptrf PE=1 SV=1
A2AF67	0.061827257	0	0	0	0	3	2	0	3	Dedicator of cytokinesis protein 11 OS=Mus musculus GN=Dock11 PE=1 SV=1
Q99K01	0.061829808	0	0	0	0	5	3	3	0	Pyridoxal-dependent decarboxylase domain-containing protein 1 OS=Mus musculus GN=Pdxdc1 PE=1 SV=2
A0A0R4J034	0.061829808	0	0	0	0	5	3	3	0	MCG129810, isoform CRA_c OS=Mus musculus GN=Pdxdc1 PE=1 SV=1
O54988	0.062594515	0	0	0	0	9	2	6	2	STE20-like serine/threonine-protein kinase OS=Mus musculus GN=Slk PE=1 SV=2
P06684	0.063102753	0	0	0	0	5	5	0	4	Complement C5 OS=Mus musculus GN=C5 PE=1 SV=2
F7CVJ5	0.06311774	0	0	0	0	7	4	0	6	Protein Ahnak2 (Fragment) OS=Mus musculus GN=Ahnak2 PE=1 SV=1
Q561M1	0.063850636	0	0	0	0	11	9	0	7	Acp1 protein OS=Mus musculus GN=Acp1 PE=1 SV=1
Q923D2	0.064237986	0	0	0	0	0	4	3	6	Flavin reductase (NADPH) OS=Mus musculus GN=Blvrb PE=1 SV=3
E9PZC3	0.064237986	0	0	0	0	0	4	3	6	Flavin reductase (NADPH) OS=Mus musculus GN=Blvrb PE=1 SV=1
Q61166	0.064901439	0	0	0	0	4	4	0	3	Microtubule-associated protein RP/EB family member 1 OS=Mus musculus GN=Mapre1 PE=1 SV=3
A0A0J9YUN4	0.065061703	0	0	0	0	10	6	0	10	Dynamin-1 OS=Mus musculus GN=Dnm1 PE=1 SV=1
A2AF47	0.06691555	0	0	0	0	4	2	0	3	Dedicator of cytokinesis protein 11 OS=Mus musculus GN=Dock11 PE=1 SV=1
Q5SVJ1	0.067234945	0	0	0	0	6	0	4	8	Calcium/calmodulin-dependent protein kinase type II subunit beta OS=Mus musculus GN=Camk2b PE=1 SV=1
Q5SVJ0	0.067234945	0	0	0	0	6	0	4	8	Calcium/calmodulin-dependent protein kinase II, beta, isoform CRA_b OS=Mus musculus GN=Camk2b PE=1 SV=1
Q5SVI0	0.067234945	0	0	0	0	6	0	4	8	Calcium/calmodulin-dependent protein kinase type II subunit beta OS=Mus musculus GN=Camk2b PE=1 SV=1
Q5SVI1	0.067234945	0	0	0	0	6	0	4	8	Calcium/calmodulin-dependent protein kinase type II subunit beta OS=Mus musculus GN=Camk2b PE=1 SV=1
Q5SVI2	0.067234945	0	0	0	0	6	0	4	8	Calcium/calmodulin-dependent protein kinase type II subunit beta OS=Mus musculus GN=Camk2b PE=1 SV=1
Q5SVI3	0.067234945	0	0	0	0	6	0	4	8	Calcium/calmodulin-dependent protein kinase type II subunit beta OS=Mus musculus GN=Camk2b PE=1 SV=1
Q5SVI9	0.067234945	0	0	0	0	6	0	4	8	Calcium/calmodulin-dependent protein kinase type II subunit beta OS=Mus musculus GN=Camk2b PE=1 SV=1
P28652	0.067234945	0	0	0	0	6	0	4	8	Calcium/calmodulin-dependent protein kinase type II subunit beta OS=Mus musculus GN=Camk2b PE=1 SV=2
Q923T9	0.067234945	0	0	0	0	6	0	4	8	Calcium/calmodulin-dependent protein kinase type II subunit gamma OS=Mus musculus GN=Camk2g PE=1 SV=1
E9Q1V9	0.067234945	0	0	0	0	6	0	4	8	Calcium/calmodulin-dependent protein kinase type II subunit delta OS=Mus musculus GN=Camk2d PE=1 SV=2
E9Q1W0	0.067234945	0	0	0	0	6	0	4	8	Calcium/calmodulin-dependent protein kinase type II subunit delta OS=Mus musculus GN=Camk2d PE=1 SV=2
E9Q1T1	0.067234945	0	0	0	0	6	0	4	8	Calcium/calmodulin-dependent protein kinase type II subunit delta OS=Mus musculus GN=Camk2d PE=1 SV=1
Q68EG2	0.067234945	0	0	0	0	6	0	4	8	Calcium/calmodulin-dependent protein kinase type II subunit beta OS=Mus musculus GN=Camk2b PE=1 SV=1
A0A0G2JGS4	0.067234945	0	0	0	0	6	0	4	8	Calcium/calmodulin-dependent protein kinase type II subunit delta OS=Mus musculus GN=Camk2d PE=1 SV=1
Q6PHZ2	0.067234945	0	0	0	0	6	0	4	8	Calcium/calmodulin-dependent protein kinase type II subunit delta OS=Mus musculus GN=Camk2d PE=1 SV=1
P24472	0.067808674	0	0	0	0	6	3	0	4	Glutathione S-transferase A4 OS=Mus musculus GN=Gsta4 PE=1 SV=3
Q9DAW9	0.068505488	0	0	0	0	4	5	0	4	Calponin-3 OS=Mus musculus GN=Cnn3 PE=1 SV=1
A0A0G2JDV8	0.068505488	0	0	0	0	4	5	0	4	Calponin OS=Mus musculus GN=Cnn3 PE=1 SV=1
Q02788	0.068559656	0	0	0	0	10	12	2	3	Collagen alpha-2(VI) chain OS=Mus musculus GN=Col6a2 PE=1 SV=3
P24527	0.069060706	0	0	0	0	3	3	0	2	Leukotriene A-4 hydrolase OS=Mus musculus GN=Lta4h PE=1 SV=4
A0A0R4J086	0.07046827	0	0	0	0	13	6	0	9	Olfactomedin-like protein 3 OS=Mus musculus GN=Olfml3 PE=1 SV=1
Q00915	0.071091559	0	0	0	0	6	4	0	3	Retinol-binding protein 1 OS=Mus musculus GN=Rbp1 PE=1 SV=2
A0A0R4J0S4	0.071228456	0	0	0	0	10	7	0	5	Lethal(2) giant larvae protein homolog 1 OS=Mus musculus GN=Llgl1 PE=1 SV=1
Q80Y17	0.071228456	0	0	0	0	10	7	0	5	Lethal(2) giant larvae protein homolog 1 OS=Mus musculus GN=Llgl1 PE=1 SV=1
Q8CBG6	0.071715916	0	0	0	0	9	8	0	5	6-phosphogluconolactonase OS=Mus musculus GN=Pgls PE=1 SV=1
Q9R257	0.071854304	0	0	0	0	5	7	0	6	Heme-binding protein 1 OS=Mus musculus GN=Hebp1 PE=1 SV=2
A0A140T8J4	0.071854304	0	0	0	0	5	7	0	6	Heme binding protein 1 OS=Mus musculus GN=Hebp1 PE=1 SV=1
Q9CZ44	0.072476027	0	0	0	0	4	0	2	5	NSFL1 cofactor p47 OS=Mus musculus GN=Nsfl1c PE=1 SV=1
Q9CR86	0.076053456	0	0	0	0	3	2	0	4	Calcium-regulated heat stable protein 1 OS=Mus musculus GN=Carhsp1 PE=1 SV=1
E9Q9E4	0.076469602	0	0	0	0	0	7	4	4	Cyclin-dependent kinase 4 (Fragment) OS=Mus musculus GN=Cdk4 PE=1 SV=1
P61226	0.078037625	0	0	0	0	3	3	0	5	Ras-related protein Rap-2b OS=Mus musculus GN=Rap2b PE=1 SV=1
P00493	0.078037625	0	0	0	0	3	3	0	5	Hypoxanthine-guanine phosphoribosyltransferase OS=Mus musculus GN=Hprt1 PE=1 SV=3
Q8BK62	0.079270066	0	0	0	0	15	6	0	9	Olfactomedin-like protein 3 OS=Mus musculus GN=Olfml3 PE=2 SV=2
P70206	0.080022834	0	0	0	0	4	3	0	6	Plexin-A1 OS=Mus musculus GN=Plxna1 PE=1 SV=1
Q9DB34	0.080972725	0	0	0	0	2	3	0	2	Charged multivesicular body protein 2a OS=Mus musculus GN=Chmp2a PE=1 SV=1
D3Z4X1	0.081499815	0	0	0	0	7	4	2	0	6-phosphogluconolactonase OS=Mus musculus GN=Pgls PE=1 SV=1
Reverse_sp|Q9QY33|TSN3_MOUSE	0.082732269	0	0	0	0	3	4	4	0	Tetraspanin-3 OS=Mus musculus GN=Tspan3 PE=1 SV=1
Q8K482	0.08335286	0	0	0	0	7	6	0	3	EMILIN-2 OS=Mus musculus GN=Emilin2 PE=1 SV=1
Q8C3V4	0.084103981	0	0	0	0	2	3	0	4	Signal transducer and activator of transcription OS=Mus musculus GN=Stat1 PE=1 SV=1
A0A087WSP5	0.084103981	0	0	0	0	2	3	0	4	Signal transducer and activator of transcription OS=Mus musculus GN=Stat1 PE=1 SV=1
P42225	0.084103981	0	0	0	0	2	3	0	4	Signal transducer and activator of transcription 1 OS=Mus musculus GN=Stat1 PE=1 SV=1
G3X8Q5	0.084410115	0	0	0	0	5	7	0	10	Ceruloplasmin OS=Mus musculus GN=Cp PE=1 SV=1
Q61147	0.084410115	0	0	0	0	5	7	0	10	Ceruloplasmin OS=Mus musculus GN=Cp PE=1 SV=2
E9PZD8	0.084410115	0	0	0	0	5	7	0	10	Ceruloplasmin OS=Mus musculus GN=Cp PE=1 SV=1
G3X9T8	0.084410115	0	0	0	0	5	7	0	10	Ceruloplasmin OS=Mus musculus GN=Cp PE=1 SV=1
Q8CG16	0.084437203	0	0	0	0	6	10	0	7	Complement C1r-A subcomponent OS=Mus musculus GN=C1ra PE=1 SV=1
G5E8G0	0.085270199	0	0	0	0	10	4	0	5	Heterogeneous nuclear ribonucleoprotein D, isoform CRA_b OS=Mus musculus GN=Hnrnpd PE=1 SV=1
G3X9W0	0.085270199	0	0	0	0	10	4	0	5	Heterogeneous nuclear ribonucleoprotein D, isoform CRA_a OS=Mus musculus GN=Hnrnpd PE=1 SV=1
P70202	0.087887774	0	0	0	0	5	3	5	0	Latexin OS=Mus musculus GN=Lxn PE=1 SV=2
P08121	0.088633399	0	0	0	0	12	9	3	0	Collagen alpha-1(III) chain OS=Mus musculus GN=Col3a1 PE=1 SV=4
Q8BFW7	0.089152769	0	0	0	0	7	3	0	3	Lipoma-preferred partner homolog OS=Mus musculus GN=Lpp PE=1 SV=1
Q8BK64	0.08930278	0	0	0	0	3	4	0	2	Activator of 90 kDa heat shock protein ATPase homolog 1 OS=Mus musculus GN=Ahsa1 PE=1 SV=2
B1AQD3	0.090893068	0	0	0	0	4	0	6	6	Ras-related protein Rab-34 OS=Mus musculus GN=Rab34 PE=1 SV=1
Q64008	0.090893068	0	0	0	0	4	0	6	6	Ras-related protein Rab-34 OS=Mus musculus GN=Rab34 PE=1 SV=2
Q0PD20	0.090893068	0	0	0	0	4	0	6	6	Rab34 OS=Mus musculus GN=Rab34 PE=1 SV=1
Q99K94	0.093851909	0	0	0	0	2	2	0	4	Signal transducer and activator of transcription OS=Mus musculus GN=Stat1 PE=1 SV=1
G3X9H8	0.093851909	0	0	0	0	4	4	0	8	E3 ubiquitin-protein ligase NEDD4-like OS=Mus musculus GN=Nedd4l PE=1 SV=1
F6VLT8	0.094007766	0	0	0	0	0	3	6	6	tRNA (guanine-N(7)-)-methyltransferase non-catalytic subunit WDR4 (Fragment) OS=Mus musculus GN=Wdr4 PE=1 SV=1
Q99KC8	0.094303581	0	0	0	0	4	3	0	7	von Willebrand factor A domain-containing protein 5A OS=Mus musculus GN=Vwa5a PE=1 SV=2
P82198	0.095192261	0	0	0	0	0	2	3	2	Transforming growth factor-beta-induced protein ig-h3 OS=Mus musculus GN=Tgfbi PE=1 SV=1
P53690	0.10475924	0	0	0	0	2	0	2	5	Matrix metalloproteinase-14 OS=Mus musculus GN=Mmp14 PE=2 SV=3
Q8CFI0	0.106450822	0	0	0	0	4	4	0	9	E3 ubiquitin-protein ligase NEDD4-like OS=Mus musculus GN=Nedd4l PE=1 SV=2
B1AQD4	0.107380535	0	0	0	0	3	0	6	6	Ras-related protein Rab-34 (Fragment) OS=Mus musculus GN=Rab34 PE=1 SV=1
D6RHA2	0.108131208	0	0	0	0	2	0	3	2	N-acylglucosamine 2-epimerase OS=Mus musculus GN=Renbp PE=1 SV=2

P82343	0.108131208	0	0	0	0	2	0	3	2	N-acylglucosamine 2-epimerase OS=Mus musculus GN=Renbp PE=1 SV=3
P70193	0.110262818	0	0	0	0	0	6	7	3	Leucine-rich repeats and immunoglobulin-like domains protein 1 OS=Mus musculus GN=Lrig1 PE=1 SV=2
E9Q1X8	0.110788078	0	0	0	0	0	2	2	6	Voltage-dependent calcium channel subunit alpha-2/delta-1 OS=Mus musculus GN=Cacna2d1 PE=1 SV=2
O08532	0.110788078	0	0	0	0	0	2	2	6	Voltage-dependent calcium channel subunit alpha-2/delta-1 OS=Mus musculus GN=Cacna2d1 PE=1 SV=1
Q6NXL1	0.116682183	0	0	0	0	2	6	3	0	Protein Sec24d OS=Mus musculus GN=Sec24d PE=1 SV=1
O89086	0.11928607	0	0	0	0	2	0	4	3	RNA-binding protein 3 OS=Mus musculus GN=Rbm3 PE=1 SV=1
Q8BG13	0.11928607	0	0	0	0	2	0	4	3	Putative uncharacterized protein OS=Mus musculus GN=Rbm3 PE=1 SV=1
Q9QZM0	0.138979589	0	0	0	0	2	5	0	2	Ubiquilin-2 OS=Mus musculus GN=Ubqln2 PE=1 SV=2
Reverse_tr|E0CXL4|E0CXL4_MOUSE	0.181697101	0	0	0	0	5	4	0	0	Partitioning defective 3 homolog OS=Mus musculus GN=Pard3 PE=1 SV=1
Reverse_sp|Q99NH2|PARD3_MOUSE	0.181697101	0	0	0	0	5	4	0	0	Partitioning defective 3 homolog OS=Mus musculus GN=Pard3 PE=1 SV=2
P25446	0.181697101	0	0	0	0	5	4	0	0	Tumor necrosis factor receptor superfamily member 6 OS=Mus musculus GN=Fas PE=1 SV=2
Q01721	0.181697101	0	0	0	0	5	4	0	0	Growth arrest-specific protein 1 OS=Mus musculus GN=Gas1 PE=2 SV=2
Reverse_tr|E0CZE2|E0CZE2_MOUSE	0.181697101	0	0	0	0	5	4	0	0	Partitioning defective 3 homolog (Fragment) OS=Mus musculus GN=Pard3 PE=1 SV=1
Reverse_tr|B7ZNY3|B7ZNY3_MOUSE	0.181697101	0	0	0	0	5	4	0	0	Pard3 protein OS=Mus musculus GN=Pard3 PE=1 SV=1
Reverse_tr|A0A0R4J1Y4|A0A0R4J1Y4_MOUSE	0.181697101	0	0	0	0	5	4	0	0	Partitioning defective 3 homolog OS=Mus musculus GN=Pard3 PE=1 SV=1
Reverse_tr|E0CX45|E0CX45_MOUSE	0.181697101	0	0	0	0	5	4	0	0	Partitioning defective 3 homolog (Fragment) OS=Mus musculus GN=Pard3 PE=1 SV=1
Reverse_tr|F6UGU7|F6UGU7_MOUSE	0.181697101	0	0	0	0	5	4	0	0	Partitioning defective 3 homolog (Fragment) OS=Mus musculus GN=Pard3 PE=1 SV=1
A0A0R4J0T7	0.181697101	0	0	0	0	5	4	0	0	Growth arrest-specific protein 1 OS=Mus musculus GN=Gas1 PE=1 SV=1
Reverse_tr|G3XA13|G3XA13_MOUSE	0.181697101	0	0	0	0	5	4	0	0	Par-3 (Partitioning defective 3) homolog (C. elegans) OS=Mus musculus GN=Pard3 PE=1 SV=1
Reverse_tr|A5D6P2|A5D6P2_MOUSE	0.181697101	0	0	0	0	5	4	0	0	Par-3 (Partitioning defective 3) homolog (C. elegans) OS=Mus musculus GN=Pard3 PE=1 SV=1
D3Z765	0.181783016	0	0	0	0	0	0	2	3	Putative RNA-binding protein Luc7-like 1 (Fragment) OS=Mus musculus GN=Luc7l PE=1 SV=1
Reverse_sp|Q99JR8|SMRD2_MOUSE	0.181783016	0	0	0	0	0	0	2	3	SWI/SNF-related matrix-associated actin-dependent regulator of chromatin subfamily D member 2 OS=Mus musculus GN=Smarcd2 PE=1 SV=2
D3YZ19	0.181783016	0	0	0	0	0	0	2	3	DNA-directed RNA polymerase II subunit RPB11 OS=Mus musculus GN=Polr2j PE=1 SV=1
A0A0R4J047	0.181783016	0	0	0	0	0	0	2	3	Luc7 homolog (S. cerevisiae)-like, isoform CRA_c OS=Mus musculus GN=Luc7l PE=1 SV=1
Q3TIR6	0.181932095	0	0	0	0	4	3	0	0	Prefoldin subunit 3 OS=Mus musculus GN=Vbp1 PE=1 SV=1
Q921I9	0.181932095	0	0	0	0	4	3	0	0	Exosome complex component RRP41 OS=Mus musculus GN=Exosc4 PE=1 SV=3
Q6VGS5	0.182507666	0	0	0	0	0	3	0	3	Protein Daple OS=Mus musculus GN=Ccdc88c PE=1 SV=1
Q8VD75	0.182507666	0	0	0	0	0	2	0	2	Huntingtin-interacting protein 1 OS=Mus musculus GN=Hip1 PE=1 SV=2
Q9ESN9	0.182507666	0	0	0	0	0	2	0	2	C-Jun-amino-terminal kinase-interacting protein 3 OS=Mus musculus GN=Mapk8ip3 PE=1 SV=1
J3QNR6	0.182507666	0	0	0	0	0	2	0	2	C-Jun-amino-terminal kinase-interacting protein 3 OS=Mus musculus GN=Mapk8ip3 PE=1 SV=1
Q8R3B1	0.182507666	0	0	0	0	0	4	0	4	1-phosphatidylinositol 4,5-bisphosphate phosphodiesterase delta-1 OS=Mus musculus GN=Plcd1 PE=1 SV=2
E9Q6B6	0.182507666	0	0	0	0	0	2	0	2	C-Jun-amino-terminal kinase-interacting protein 3 OS=Mus musculus GN=Mapk8ip3 PE=1 SV=1
E9Q6E0	0.182507666	0	0	0	0	0	2	0	2	C-Jun-amino-terminal kinase-interacting protein 3 OS=Mus musculus GN=Mapk8ip3 PE=1 SV=1
Q5SW88	0.182507666	0	0	0	0	0	3	0	3	Protein Rab1a OS=Mus musculus GN=Rab1a PE=1 SV=1
Q5SW87	0.182507666	0	0	0	0	0	3	0	3	Protein Rab1a OS=Mus musculus GN=Rab1a PE=1 SV=1
K3W4S4	0.182507666	0	0	0	0	0	2	0	2	C-Jun-amino-terminal kinase-interacting protein 3 OS=Mus musculus GN=Mapk8ip3 PE=1 SV=1
Q60817	0.182862354	0	0	0	0	0	4	0	5	Nascent polypeptide-associated complex subunit alpha OS=Mus musculus GN=Naca PE=1 SV=1
P50518	0.183048992	0	0	0	0	2	0	0	2	V-type proton ATPase subunit E 1 OS=Mus musculus GN=Atp6v1e1 PE=1 SV=2
P47791	0.183048992	0	0	0	0	4	0	0	4	Glutathione reductase, mitochondrial OS=Mus musculus GN=Gsr PE=1 SV=3
Q9JHI0	0.183048992	0	0	0	0	4	0	0	4	Matrix metalloproteinase-19 OS=Mus musculus GN=Mmp19 PE=2 SV=1
A0A1B0GRG1	0.183048992	0	0	0	0	4	0	0	4	Ras-related protein R-Ras (Fragment) OS=Mus musculus GN=Rras PE=1 SV=1
A0A0N4SW07	0.183048992	0	0	0	0	2	0	0	2	V-type proton ATPase subunit E 1 (Fragment) OS=Mus musculus GN=Atp6v1e1 PE=1 SV=1
A0A0N4SW34	0.183048992	0	0	0	0	2	0	0	2	V-type proton ATPase subunit E 1 (Fragment) OS=Mus musculus GN=Atp6v1e1 PE=1 SV=1
P62743	0.18314734	0	0	0	0	0	3	2	0	AP-2 complex subunit sigma OS=Mus musculus GN=Ap2s1 PE=1 SV=1
Q91YR9	0.183629719	0	0	0	0	4	0	0	3	Prostaglandin reductase 1 OS=Mus musculus GN=Ptgr1 PE=1 SV=2
Q9Z2H5	0.183629719	0	0	0	0	4	0	0	3	Band 4.1-like protein 1 OS=Mus musculus GN=Epb41l1 PE=1 SV=2
E9PV14	0.183629719	0	0	0	0	4	0	0	3	Band 4.1-like protein 1 (Fragment) OS=Mus musculus GN=Epb41l1 PE=1 SV=1
Q9CPU0	0.183629719	0	0	0	0	4	0	0	3	Lactoylglutathione lyase OS=Mus musculus GN=Glo1 PE=1 SV=3
A2AUK5	0.183629719	0	0	0	0	4	0	0	3	Band 4.1-like protein 1 OS=Mus musculus GN=Epb41l1 PE=1 SV=1
A2AUK8	0.183629719	0	0	0	0	4	0	0	3	Band 4.1-like protein 1 OS=Mus musculus GN=Epb41l1 PE=1 SV=1
A2AUK6	0.183629719	0	0	0	0	4	0	0	3	Band 4.1-like protein 1 OS=Mus musculus GN=Epb41l1 PE=1 SV=1
A2AUK7	0.183629719	0	0	0	0	4	0	0	3	Band 4.1-like protein 1 OS=Mus musculus GN=Epb41l1 PE=1 SV=1
Q922R8	0.184049837	0	0	0	0	6	4	0	0	Protein disulfide-isomerase A6 OS=Mus musculus GN=Pdia6 PE=1 SV=3
F7CJN9	0.185306662	0	0	0	0	4	0	3	0	Serotransferrin (Fragment) OS=Mus musculus GN=Trf PE=4 SV=1
Q91ZJ5	0.185306662	0	0	0	0	4	0	3	0	UTP--glucose-1-phosphate uridylyltransferase OS=Mus musculus GN=Ugp2 PE=1 SV=3
Q9JLB0	0.185925393	0	0	0	0	3	3	0	0	MAGUK p55 subfamily member 6 OS=Mus musculus GN=Mpp6 PE=1 SV=1
P48774	0.185925393	0	0	0	0	4	4	0	0	Glutathione S-transferase Mu 5 OS=Mus musculus GN=Gstm5 PE=1 SV=1
Q9ERG0	0.185925393	0	0	0	0	2	2	0	0	LIM domain and actin-binding protein 1 OS=Mus musculus GN=Lima1 PE=1 SV=3
Q5NCU4	0.185925393	0	0	0	0	2	2	0	0	SPARC OS=Mus musculus GN=Sparc PE=1 SV=1
P07214	0.185925393	0	0	0	0	2	2	0	0	SPARC OS=Mus musculus GN=Sparc PE=1 SV=1
Q58A65	0.185925393	0	0	0	0	2	2	0	0	C-Jun-amino-terminal kinase-interacting protein 4 OS=Mus musculus GN=Spag9 PE=1 SV=2
B2RQG2	0.185925393	0	0	0	0	3	3	0	0	PHD finger protein 3 OS=Mus musculus GN=Phf3 PE=1 SV=1
E9Q512	0.187361206	0	0	0	0	0	3	3	0	Protein Trip11 OS=Mus musculus GN=Trip11 PE=1 SV=1
E9Q0U3	0.187361206	0	0	0	0	0	2	2	0	BRCA1-A complex subunit BRE OS=Mus musculus GN=Bre PE=1 SV=1
Q8BVJ9	0.187361206	0	0	0	0	0	3	3	0	Protein Trip11 (Fragment) OS=Mus musculus GN=Trip11 PE=1 SV=1
Q9CQX6	0.187361206	0	0	0	0	0	6	6	0	MCG141091, isoform CRA_a OS=Mus musculus GN=Gm16286 PE=1 SV=1
Q9R059	0.187555485	0	0	0	0	9	0	0	6	Four and a half LIM domains protein 3 OS=Mus musculus GN=Fhl3 PE=1 SV=2
P06837	0.187555485	0	0	0	0	3	0	0	2	Neuromodulin OS=Mus musculus GN=Gap43 PE=1 SV=1
Q1MX43	0.187555485	0	0	0	0	3	0	0	2	Protein kinase C OS=Mus musculus GN=Prkcd PE=1 SV=1
Q1MX42	0.187555485	0	0	0	0	3	0	0	2	Protein kinase C OS=Mus musculus GN=Prkcd PE=1 SV=1
Q1MX41	0.187555485	0	0	0	0	3	0	0	2	Protein kinase C delta type OS=Mus musculus GN=Prkcd PE=1 SV=1
Q1MX40	0.187555485	0	0	0	0	3	0	0	2	Protein kinase C OS=Mus musculus GN=Prkcd PE=1 SV=1
P28867	0.187555485	0	0	0	0	3	0	0	2	Protein kinase C delta type OS=Mus musculus GN=Prkcd PE=1 SV=3
Q9D997	0.187717479	0	0	0	0	5	3	0	0	N-acetyl-D-glucosamine kinase OS=Mus musculus GN=Nagk PE=1 SV=1
Q9QZ08	0.187717479	0	0	0	0	5	3	0	0	N-acetyl-D-glucosamine kinase OS=Mus musculus GN=Nagk PE=1 SV=3
G3UYU4	0.189284287	0	0	0	0	5	0	0	6	Flotillin-1 OS=Mus musculus GN=Flot1 PE=1 SV=1
Q60631	0.189826693	0	0	0	0	0	5	0	4	Growth factor receptor-bound protein 2 OS=Mus musculus GN=Grb2 PE=1 SV=1
B1AT92	0.189826693	0	0	0	0	0	5	0	4	Growth factor receptor-bound protein 2 OS=Mus musculus GN=Grb2 PE=1 SV=1
O08795	0.192234684	0	0	0	0	0	0	2	2	Glucosidase 2 subunit beta OS=Mus musculus GN=Prkcsh PE=1 SV=1
Q3UDM0	0.192234684	0	0	0	0	0	0	3	3	MOB kinase activator 1B OS=Mus musculus GN=Mob1b PE=1 SV=1
Q921Y0	0.192234684	0	0	0	0	0	0	3	3	MOB kinase activator 1A OS=Mus musculus GN=Mob1a PE=2 SV=3
Q8BPB0	0.192234684	0	0	0	0	0	0	3	3	MOB kinase activator 1B OS=Mus musculus GN=Mob1b PE=1 SV=3
Q64339	0.194957854	0	0	0	0	3	0	0	4	Ubiquitin-like protein ISG15 OS=Mus musculus GN=Isg15 PE=1 SV=4
D3Z4U3	0.1952413	0	0	0	0	0	4	2	0	Ubiquitin-conjugating enzyme E2 K OS=Mus musculus GN=Ube2k PE=1 SV=1
Q921M7	0.197263378	0	0	0	0	4	5	0	0	Protein FAM49B OS=Mus musculus GN=Fam49b PE=1 SV=1
Q61792	0.19758519	0	0	0	0	6	3	0	0	LIM and SH3 domain protein 1 OS=Mus musculus GN=Lasp1 PE=1 SV=1
B7ZCP6	0.200381786	0	0	0	0	3	0	3	0	Copine-1 (Fragment) OS=Mus musculus GN=Cpne1 PE=1 SV=2
P06795	0.200381786	0	0	0	0	2	0	2	0	Multidrug resistance protein 1B OS=Mus musculus GN=Abcb1b PE=1 SV=1
A0A1B0GR63	0.200707146	0	0	0	0	0	3	0	2	Charged multivesicular body protein 2a OS=Mus musculus GN=Chmp2a PE=1 SV=1
A0A1B0GT75	0.200707146	0	0	0	0	0	3	0	2	Charged multivesicular body protein 2a OS=Mus musculus GN=Chmp2a PE=1 SV=1
A0A1B0GSH4	0.200707146	0	0	0	0	0	3	0	2	Charged multivesicular body protein 2a OS=Mus musculus GN=Chmp2a PE=1 SV=1
Q08879	0.200707146	0	0	0	0	0	3	0	2	Fibulin-1 OS=Mus musculus GN=Fbln1 PE=1 SV=2
P31230	0.201637289	0	0	0	0	3	4	0	0	Aminoacyl tRNA synthase complex-interacting multifunctional protein 1 OS=Mus musculus GN=Aimp1 PE=1 SV=2
Q3UZG4	0.201637289	0	0	0	0	3	4	0	0	Aminoacyl tRNA synthase complex-interacting multifunctional protein 1 OS=Mus musculus GN=Aimp1 PE=1 SV=1
G5E8J0	0.202805091	0	0	0	0	2	0	0	3	Neurogenic locus notch homolog protein 2 OS=Mus musculus GN=Notch2 PE=1 SV=1
F7BE32	0.204062024	0	0	0	0	0	0	6	5	Ras-related protein Rab-34 (Fragment) OS=Mus musculus GN=Rab34 PE=1 SV=1
P12032	0.204347839	0	0	0	0	0	3	4	0	Metalloproteinase inhibitor 1 OS=Mus musculus GN=Timp1 PE=1 SV=2
Q61703	0.20469728	0	0	0	0	6	0	0	3	Inter-alpha-trypsin inhibitor heavy chain H2 OS=Mus musculus GN=Itih2 PE=1 SV=1
G3X977	0.20469728	0	0	0	0	6	0	0	3	Inter-alpha trypsin inhibitor, heavy chain 2 OS=Mus musculus GN=Itih2 PE=1 SV=1
Q80X81	0.20469728	0	0	0	0	6	0	0	3	Acetyl-Coenzyme A acetyltransferase 3 OS=Mus musculus GN=Acat3 PE=1 SV=1
P28301	0.205926322	0	0	0	0	9	4	0	0	Protein-lysine 6-oxidase OS=Mus musculus GN=Lox PE=1 SV=1
O88428	0.210607504	0	0	0	0	2	3	0	0	Bifunctional 3'-phosphoadenosine 5'-phosphosulfate synthase 2 OS=Mus musculus GN=Papss2 PE=1 SV=2
A2AT02	0.210919196	0	0	0	0	3	0	0	5	NSFL1 cofactor p47 OS=Mus musculus GN=Nsfl1c PE=1 SV=1
Q8R550	0.213667784	0	0	0	0	0	2	3	0	SH3 domain-containing kinase-binding protein 1 OS=Mus musculus GN=Sh3kbp1 PE=1 SV=1
B0R0Y8	0.213667784	0	0	0	0	0	2	3	0	SH3 domain-containing kinase-binding protein 1 (Fragment) OS=Mus musculus GN=Sh3kbp1 PE=1 SV=1
P35288	0.222420871	0	0	0	0	0	0	3	2	Ras-related protein Rab-23 OS=Mus musculus GN=Rab23 PE=1 SV=2
Q9D4I9	0.222420871	0	0	0	0	0	0	3	2	Putative uncharacterized protein OS=Mus musculus GN=Rab23 PE=1 SV=1
Q61035	0.224016507	0	0	0	0	0	4	0	2	Histidine--tRNA ligase, cytoplasmic OS=Mus musculus GN=Hars PE=1 SV=2
Q99JY8	0.226672289	0	0	0	0	2	0	0	4	Phospholipid phosphatase 3 OS=Mus musculus GN=Plpp3 PE=1 SV=1
O35516	0.226672289	0	0	0	0	2	0	0	4	Neurogenic locus notch homolog protein 2 OS=Mus musculus GN=Notch2 PE=1 SV=1
G3XA25	0.229008823	0	0	0	0	8	0	0	3	Acetyl-CoA acetyltransferase, cytosolic OS=Mus musculus GN=Acat2 PE=1 SV=1
Q8CAY6	0.229008823	0	0	0	0	8	0	0	3	Acetyl-CoA acetyltransferase, cytosolic OS=Mus musculus GN=Acat2 PE=1 SV=2
Q6NZD2	0.234346693	0	0	0	0	2	0	3	0	Putative uncharacterized protein OS=Mus musculus GN=Snx1 PE=1 SV=1
contaminant_UBIQUITIN09	0.234346693	0	0	0	0	2	0	3	0	no description
Q9WV80	0.234346693	0	0	0	0	2	0	3	0	Sorting nexin-1 OS=Mus musculus GN=Snx1 PE=1 SV=1
Q9ER72	0.234346693	0	0	0	0	2	0	3	0	Cysteine--tRNA ligase, cytoplasmic OS=Mus musculus GN=Cars PE=1 SV=2
Q8BND5	0.250454941	0	0	0	0	3	7	0	0	Sulfhydryl oxidase 1 OS=Mus musculus GN=Qsox1 PE=1 SV=1
P26883	0.27385809	0	0	0	0	13	0	0	3	Peptidyl-prolyl cis-trans isomerase FKBP1A OS=Mus musculus GN=Fkbp1a PE=1 SV=2
P25799	0.286716188	0	0	0	0	2	7	0	0	Nuclear factor NF-kappa-B p105 subunit OS=Mus musculus GN=Nfkb1 PE=1 SV=2
D6RFU9	0.391002219	0	0	0	0	0	0	0	5	Synaptophysin-like protein 1 OS=Mus musculus GN=Sypl PE=1 SV=1
Reverse_tr|A2A7T3|A2A7T3_MOUSE	0.391002219	0	0	0	0	0	0	0	4	Arginine-glutamic acid dipeptide repeats protein OS=Mus musculus GN=Rere PE=1 SV=1
Q9R1L5	0.391002219	0	0	0	0	2	0	0	0	Microtubule-associated serine/threonine-protein kinase 1 OS=Mus musculus GN=Mast1 PE=1 SV=3
O55091	0.391002219	0	0	0	0	0	0	0	6	Protein IMPACT OS=Mus musculus GN=Impact PE=1 SV=2
E9Q3L4	0.391002219	0	0	0	0	2	0	0	0	Protein Ifi207 OS=Mus musculus GN=Ifi207 PE=1 SV=2
E9Q3L2	0.391002219	0	0	0	0	0	0	0	2	Phosphatidylinositol 4-kinase alpha OS=Mus musculus GN=Pi4ka PE=1 SV=2
Q5D0E0	0.391002219	0	0	0	0	2	0	0	0	Ikbkb protein OS=Mus musculus GN=Ikbkb PE=1 SV=1
Q9CXD9	0.391002219	0	0	0	0	0	0	2	0	Leucine-rich repeat-containing protein 17 OS=Mus musculus GN=Lrrc17 PE=2 SV=1
D3Z794	0.391002219	0	0	0	0	2	0	0	0	Small ubiquitin-related modifier 2 OS=Mus musculus GN=Sumo2 PE=1 SV=1
P58389	0.391002219	0	0	0	0	0	0	0	2	Serine/threonine-protein phosphatase 2A activator OS=Mus musculus GN=Ppp2r4 PE=1 SV=1
G3UYF9	0.391002219	0	0	0	0	2	0	0	0	Prefoldin subunit 6 OS=Mus musculus GN=Pfdn6 PE=1 SV=1
Q99J93	0.391002219	0	0	0	0	0	0	0	7	Interferon-induced transmembrane protein 2 OS=Mus musculus GN=Ifitm2 PE=1 SV=1
Q9QY06	0.391002219	0	0	0	0	0	0	0	4	Unconventional myosin-IXb OS=Mus musculus GN=Myo9b PE=1 SV=2
Q9D0K2	0.391002219	0	0	0	0	4	0	0	0	Succinyl-CoA:3-ketoacid coenzyme A transferase 1, mitochondrial OS=Mus musculus GN=Oxct1 PE=1 SV=1
B1AW94	0.391002219	0	0	0	0	3	0	0	0	Protein kinase C and casein kinase II substrate protein 3 (Fragment) OS=Mus musculus GN=Pacsin3 PE=1 SV=1
O09117	0.391002219	0	0	0	0	0	0	0	5	Synaptophysin-like protein 1 OS=Mus musculus GN=Sypl1 PE=1 SV=2
P61957	0.391002219	0	0	0	0	2	0	0	0	Small ubiquitin-related modifier 2 OS=Mus musculus GN=Sumo2 PE=1 SV=1
Reverse_tr|F8WH29|F8WH29_MOUSE	0.391002219	0	0	0	0	0	2	0	0	Fibrocystin-L OS=Mus musculus GN=Pkhd1l1 PE=1 SV=1
Q9DBC3	0.391002219	0	0	0	0	0	0	0	2	Cap-specific mRNA (nucleoside-2'-O-)-methyltransferase 1 OS=Mus musculus GN=Cmtr1 PE=1 SV=1
I7HFT9	0.391002219	0	0	0	0	2	0	0	0	Histone H1t OS=Mus musculus GN=Hist1h1t PE=1 SV=1
Q9CXP8	0.391002219	0	0	0	0	0	0	2	0	Guanine nucleotide-binding protein G(I)/G(S)/G(O) subunit gamma-10 OS=Mus musculus GN=Gng10 PE=3 SV=1
Q9Z1E4	0.391002219	0	0	0	0	0	0	2	0	Glycogen [starch] synthase, muscle OS=Mus musculus GN=Gys1 PE=1 SV=2
Q9QZ06	0.391002219	0	0	0	0	0	2	0	0	Toll-interacting protein OS=Mus musculus GN=Tollip PE=1 SV=1
Reverse_sp|Q6NZC7|S23IP_MOUSE	0.391002219	0	0	0	0	2	0	0	0	SEC23-interacting protein OS=Mus musculus GN=Sec23ip PE=1 SV=2
P14733	0.391002219	0	0	0	0	0	0	0	3	Lamin-B1 OS=Mus musculus GN=Lmnb1 PE=1 SV=3
E0CZE0	0.391002219	0	0	0	0	0	3	0	0	NEDD8-activating enzyme E1 regulatory subunit OS=Mus musculus GN=Nae1 PE=1 SV=1
G3UYU6	0.391002219	0	0	0	0	0	0	3	0	Receptor-type tyrosine-protein phosphatase delta OS=Mus musculus GN=Ptprd PE=1 SV=1
Q8JZZ7	0.391002219	0	0	0	0	0	0	0	2	Adhesion G protein-coupled receptor L2 OS=Mus musculus GN=Adgrl2 PE=1 SV=3
Reverse_sp|Q5ND29|RILP_MOUSE	0.391002219	0	0	0	0	0	0	2	0	Rab-interacting lysosomal protein OS=Mus musculus GN=Rilp PE=1 SV=1
G3X922	0.391002219	0	0	0	0	0	0	2	0	MCG115602 OS=Mus musculus GN=Dnajc13 PE=1 SV=1
Q6VYI4	0.391002219	0	0	0	0	0	0	4	0	TAR DNA-binding protein 43 OS=Mus musculus GN=Tardbp PE=1 SV=1
Q6VYI5	0.391002219	0	0	0	0	0	0	4	0	TAR DNA-binding protein 43 OS=Mus musculus GN=Tardbp PE=1 SV=1
B9EJA3	0.391002219	0	0	0	0	0	0	3	0	Protein tyrosine phosphatase, receptor type, D OS=Mus musculus GN=Ptprd PE=1 SV=1
Q9R1Q6	0.391002219	0	0	0	0	2	0	0	0	Transmembrane protein 176B OS=Mus musculus GN=Tmem176b PE=1 SV=1
Q8CEB6	0.391002219	0	0	0	0	0	0	0	2	1,5-anhydro-D-fructose reductase OS=Mus musculus GN=Akr1e1 PE=1 SV=1
D3YU15	0.391002219	0	0	0	0	5	0	0	0	Dimethylarginine dimethylaminohydrolase 1, isoform CRA_a OS=Mus musculus GN=Ddah1 PE=1 SV=1
Reverse_tr|E9Q8F8|E9Q8F8_MOUSE	0.391002219	0	0	0	0	4	0	0	0	Protein Abca14 OS=Mus musculus GN=Abca14 PE=4 SV=1
Q5FWK3	0.391002219	0	0	0	0	0	3	0	0	Rho GTPase-activating protein 1 OS=Mus musculus GN=Arhgap1 PE=1 SV=1
Q9JK53	0.391002219	0	0	0	0	2	0	0	0	Prolargin OS=Mus musculus GN=Prelp PE=1 SV=2
Q6A0D4	0.391002219	0	0	0	0	0	0	0	2	Raftlin OS=Mus musculus GN=Rftn1 PE=1 SV=4
Reverse_tr|Z4YL78|Z4YL78_MOUSE	0.391002219	0	0	0	0	0	0	3	0	Cytoskeleton-associated protein 5 OS=Mus musculus GN=Ckap5 PE=1 SV=1
E9QMV2	0.391002219	0	0	0	0	3	0	0	0	Costars family protein ABRACL OS=Mus musculus GN=Abracl PE=1 SV=1
G3UXH4	0.391002219	0	0	0	0	0	0	0	4	Serine/threonine-protein kinase N2 OS=Mus musculus GN=Pkn2 PE=1 SV=1
Q8VBW6	0.391002219	0	0	0	0	0	3	0	0	NEDD8-activating enzyme E1 regulatory subunit OS=Mus musculus GN=Nae1 PE=1 SV=1
Reverse_sp|Q8BW94|DYH3_MOUSE	0.391002219	0	0	0	0	0	0	0	2	Dynein heavy chain 3, axonemal OS=Mus musculus GN=Dnah3 PE=1 SV=2
P70122	0.391002219	0	0	0	0	2	0	0	0	Ribosome maturation protein SBDS OS=Mus musculus GN=Sbds PE=1 SV=4

Reverse_tr|E9Q9X4|E9Q9X4_MOUSE	0.391002219	0	0	0	0	3	0	0	0	Nitric oxide synthase OS=Mus musculus GN=Nos3 PE=1 SV=1
Q9D892	0.391002219	0	0	0	0	0	2	0	0	Inosine triphosphate pyrophosphatase OS=Mus musculus GN=Itpa PE=1 SV=2
Reverse_sp|C8YR32|LOXH1_MOUSE	0.391002219	0	0	0	0	0	0	2	0	Lipoxygenase homology domain-containing protein 1 OS=Mus musculus GN=Loxhd1 PE=2 SV=1
Q9R0Y5	0.391002219	0	0	0	0	4	0	0	0	Adenylate kinase isoenzyme 1 OS=Mus musculus GN=Ak1 PE=1 SV=1
Q8K2J0	0.391002219	0	0	0	0	2	0	0	0	1-phosphatidylinositol 4,5-bisphosphate phosphodiesterase delta-3 OS=Mus musculus GN=Plcd3 PE=1 SV=2
Reverse_sp|D3YXG0|HMCN1_MOUSE	0.391002219	0	0	0	0	0	0	3	0	Hemicentin-1 OS=Mus musculus GN=Hmcn1 PE=1 SV=1
Q6P572	0.391002219	0	0	0	0	0	0	2	0	Mtmr2 protein OS=Mus musculus GN=Mtmr2 PE=1 SV=1
Q70KF4	0.391002219	0	0	0	0	2	0	0	0	Cardiomyopathy-associated protein 5 OS=Mus musculus GN=Cmya5 PE=1 SV=2
F6VXK7	0.391002219	0	0	0	0	0	0	0	2	Unconventional myosin-XV (Fragment) OS=Mus musculus GN=Myo15 PE=4 SV=1
Reverse_sp|Q9Z329|ITPR2_MOUSE	0.391002219	0	0	0	0	2	0	0	0	Inositol 1,4,5-trisphosphate receptor type 2 OS=Mus musculus GN=Itpr2 PE=1 SV=4
E9Q4S3	0.391002219	0	0	0	0	3	0	0	0	Ninein OS=Mus musculus GN=Nin PE=1 SV=1
Q60872	0.391002219	0	0	0	0	2	0	0	0	Eukaryotic translation initiation factor 1A OS=Mus musculus GN=Eif1a PE=2 SV=3
Reverse_sp|P13541|MYH3_MOUSE	0.391002219	0	0	0	0	0	2	0	0	Myosin-3 OS=Mus musculus GN=Myh3 PE=2 SV=2
B7ZCP7	0.391002219	0	0	0	0	0	0	3	0	Copine-1 (Fragment) OS=Mus musculus GN=Cpne1 PE=1 SV=1
Q3UNH9	0.391002219	0	0	0	0	0	0	2	0	Putative uncharacterized protein OS=Mus musculus GN=Tarbp2 PE=1 SV=1
H3BJH8	0.391002219	0	0	0	0	0	0	3	0	Protein Trip11 (Fragment) OS=Mus musculus GN=Trip11 PE=1 SV=1
A0A1B0GT92	0.391002219	0	0	0	0	0	0	2	0	Glycogen [starch] synthase OS=Mus musculus GN=Gys1 PE=1 SV=1
Q9ET30	0.391002219	0	0	0	0	0	0	0	2	Transmembrane 9 superfamily member 3 OS=Mus musculus GN=Tm9sf3 PE=1 SV=1
Q5SSZ5	0.391002219	0	0	0	0	2	0	0	0	Tensin-3 OS=Mus musculus GN=Tns3 PE=1 SV=1
E9PXV7	0.391002219	0	0	0	0	0	0	4	0	Protein diaphanous homolog 1 OS=Mus musculus GN=Diaph1 PE=1 SV=1
Q6PDL0	0.391002219	0	0	0	0	0	0	0	2	Cytoplasmic dynein 1 light intermediate chain 2 OS=Mus musculus GN=Dync1li2 PE=1 SV=2
F7AAP4	0.391002219	0	0	0	0	5	0	0	0	Calcium-transporting ATPase OS=Mus musculus GN=Atp2b4 PE=1 SV=1
O70439	0.391002219	0	0	0	0	2	0	0	0	Syntaxin-7 OS=Mus musculus GN=Stx7 PE=1 SV=3
Reverse_sp|Q8CJ40|CROCC_MOUSE	0.391002219	0	0	0	0	2	0	0	0	Rootletin OS=Mus musculus GN=Crocc PE=1 SV=2
Q91YR1	0.391002219	0	0	0	0	3	0	0	0	Twinfilin-1 OS=Mus musculus GN=Twf1 PE=1 SV=2
O70400	0.391002219	0	0	0	0	3	0	0	0	PDZ and LIM domain protein 1 OS=Mus musculus GN=Pdlim1 PE=1 SV=4
P23492	0.391002219	0	0	0	0	2	0	0	0	Purine nucleoside phosphorylase OS=Mus musculus GN=Pnp PE=1 SV=2
Q99PL5	0.391002219	0	0	0	0	0	2	0	0	Ribosome-binding protein 1 OS=Mus musculus GN=Rrbp1 PE=1 SV=2
A0A1B0GT68	0.391002219	0	0	0	0	0	0	0	7	Uncharacterized protein OS=Mus musculus PE=4 SV=1
F6XC54	0.391002219	0	0	0	0	0	0	4	0	Protein diaphanous homolog 1 OS=Mus musculus GN=Diaph1 PE=1 SV=1
A2AH25	0.391002219	0	0	0	0	0	3	0	0	Rho GTPase-activating protein 1 OS=Mus musculus GN=Arhgap1 PE=1 SV=1
Q9CW03	0.391002219	0	0	0	0	2	0	0	0	Structural maintenance of chromosomes protein 3 OS=Mus musculus GN=Smc3 PE=1 SV=2
G3UWK6	0.391002219	0	0	0	0	0	0	3	0	Receptor-type tyrosine-protein phosphatase delta OS=Mus musculus GN=Ptprd PE=1 SV=1
F7AAE0	0.391002219	0	0	0	0	2	0	0	0	Phosphoinositide phospholipase C (Fragment) OS=Mus musculus GN=Plcd3 PE=1 SV=1
Q922X9	0.391002219	0	0	0	0	0	2	0	0	Protein arginine N-methyltransferase 7 OS=Mus musculus GN=Prmt7 PE=1 SV=1
D3Z074	0.391002219	0	0	0	0	0	0	4	0	Protein diaphanous homolog 1 OS=Mus musculus GN=Diaph1 PE=1 SV=2
E9QKL6	0.391002219	0	0	0	0	2	0	0	0	Interferon-activable protein 204 OS=Mus musculus GN=Ifi204 PE=1 SV=1
Q9R045	0.391002219	0	0	0	0	0	0	0	3	Angiopoietin-related protein 2 OS=Mus musculus GN=Angptl2 PE=2 SV=2
E9PYL1	0.391002219	0	0	0	0	0	0	0	2	Chromodomain-helicase-DNA-binding protein 5 OS=Mus musculus GN=Chd5 PE=1 SV=1
O08539	0.391002219	0	0	0	0	5	0	0	0	Myc box-dependent-interacting protein 1 OS=Mus musculus GN=Bin1 PE=1 SV=1
Reverse_sp|Q9JLQ0|CD2AP_MOUSE	0.391002219	0	0	0	0	0	0	0	2	CD2-associated protein OS=Mus musculus GN=Cd2ap PE=1 SV=3
Q80U72	0.391002219	0	0	0	0	0	4	0	0	Protein scribble homolog OS=Mus musculus GN=Scrib PE=1 SV=2
Q04690	0.391002219	0	0	0	0	3	0	0	0	Neurofibromin OS=Mus musculus GN=Nf1 PE=1 SV=1
G3UX60	0.391002219	0	0	0	0	0	0	3	0	Receptor-type tyrosine-protein phosphatase delta OS=Mus musculus GN=Ptprd PE=1 SV=1
Reverse_tr|A2ANY6|A2ANY6_MOUSE	0.391002219	0	0	0	0	0	0	0	2	Protein Mdn1 OS=Mus musculus GN=Mdn1 PE=1 SV=1
P20352	0.391002219	0	0	0	0	0	3	0	0	Tissue factor OS=Mus musculus GN=F3 PE=1 SV=2
Q60795	0.391002219	0	0	0	0	8	0	0	0	Nuclear factor erythroid 2-related factor 2 OS=Mus musculus GN=Nfe2l2 PE=1 SV=2
P18052	0.391002219	0	0	0	0	3	0	0	0	Receptor-type tyrosine-protein phosphatase alpha OS=Mus musculus GN=Ptpra PE=1 SV=3
E9QKE4	0.391002219	0	0	0	0	2	0	0	0	Rab3 GTPase-activating protein non-catalytic subunit OS=Mus musculus GN=Rab3gap2 PE=1 SV=1
A0A1B0GSF0	0.391002219	0	0	0	0	0	0	2	0	60S ribosomal protein L13a OS=Mus musculus GN=Rpl13a PE=4 SV=1
O70591	0.391002219	0	0	0	0	0	0	0	2	Prefoldin subunit 2 OS=Mus musculus GN=Pfdn2 PE=1 SV=2
Reverse_tr|Q6P264|Q6P264_MOUSE	0.391002219	0	0	0	0	0	0	2	0	Fn3k protein OS=Mus musculus GN=Fn3k PE=1 SV=1
Q921E2	0.391002219	0	0	0	0	3	0	0	0	Ras-related protein Rab-31 OS=Mus musculus GN=Rab31 PE=1 SV=1
Reverse_sp|P51830|ADCY9_MOUSE	0.391002219	0	0	0	0	0	0	0	2	Adenylate cyclase type 9 OS=Mus musculus GN=Adcy9 PE=1 SV=1
F7AT44	0.391002219	0	0	0	0	0	2	0	0	Toll-interacting protein OS=Mus musculus GN=Tollip PE=1 SV=2
B0V2N1	0.391002219	0	0	0	0	0	2	0	0	Receptor-type tyrosine-protein phosphatase S OS=Mus musculus GN=Ptprs PE=1 SV=1
Q8BG79	0.391002219	0	0	0	0	3	0	0	0	CWF19-like protein 2 OS=Mus musculus GN=Cwf19l2 PE=1 SV=1
Q61043	0.391002219	0	0	0	0	3	0	0	0	Ninein OS=Mus musculus GN=Nin PE=1 SV=3
Q8BWW9	0.391002219	0	0	0	0	0	0	0	4	Serine/threonine-protein kinase N2 OS=Mus musculus GN=Pkn2 PE=1 SV=3
Q8BWW3	0.391002219	0	0	0	0	3	0	0	0	Phosphoacetylglucosamine mutase OS=Mus musculus GN=Pgm3 PE=1 SV=1
A0A1B0GRP7	0.391002219	0	0	0	0	0	0	0	6	Proline synthase co-transcribed bacterial homolog protein (Fragment) OS=Mus musculus GN=Prosc PE=1 SV=1
Q08024	0.391002219	0	0	0	0	0	0	0	2	Core-binding factor subunit beta OS=Mus musculus GN=Cbfb PE=1 SV=1
D3YVN1	0.391002219	0	0	0	0	9	0	0	0	Actin-like protein 6A (Fragment) OS=Mus musculus GN=Actl6a PE=1 SV=1
O08599	0.391002219	0	0	0	0	4	0	0	0	Syntaxin-binding protein 1 OS=Mus musculus GN=Stxbp1 PE=1 SV=2
B7ZNR9	0.391002219	0	0	0	0	3	0	0	0	Map4k4 protein OS=Mus musculus GN=Map4k4 PE=1 SV=1
E9PYU4	0.391002219	0	0	0	0	0	0	0	2	Chromodomain-helicase-DNA-binding protein 5 OS=Mus musculus GN=Chd5 PE=1 SV=1
Reverse_sp|Q6ZPS2|CRNS1_MOUSE	0.391002219	0	0	0	0	0	0	0	2	Carnosine synthase 1 OS=Mus musculus GN=Carns1 PE=1 SV=2
E9QLJ0	0.391002219	0	0	0	0	2	0	0	0	Cardiomyopathy-associated protein 5 OS=Mus musculus GN=Cmya5 PE=1 SV=1
Q62446	0.391002219	0	0	0	0	0	0	0	3	Peptidyl-prolyl cis-trans isomerase FKBP3 OS=Mus musculus GN=Fkbp3 PE=1 SV=2
Q68FG2	0.391002219	0	0	0	0	0	0	2	0	Protein Sptbn2 OS=Mus musculus GN=Sptbn2 PE=1 SV=1
Reverse_sp|A2AGT5|CKAP5_MOUSE	0.391002219	0	0	0	0	0	0	3	0	Cytoskeleton-associated protein 5 OS=Mus musculus GN=Ckap5 PE=1 SV=1
Q8BH58	0.391002219	0	0	0	0	0	2	0	0	TIP41-like protein OS=Mus musculus GN=Tiprl PE=1 SV=1
Reverse_tr|A0A140LIN9|A0A140LIN9_MOUSE	0.391002219	0	0	0	0	0	0	0	2	Dynein heavy chain 3, axonemal OS=Mus musculus GN=Dnah3 PE=1 SV=1
Q8BH40	0.391002219	0	0	0	0	2	0	0	0	Putative uncharacterized protein OS=Mus musculus GN=Stx7 PE=1 SV=1
Q4VA93	0.391002219	0	0	0	0	2	0	0	0	Protein kinase C OS=Mus musculus GN=Prkca PE=1 SV=1
P57787	0.391002219	0	0	0	0	0	3	0	0	Monocarboxylate transporter 4 OS=Mus musculus GN=Slc16a3 PE=1 SV=1
Q68FE6	0.391002219	0	0	0	0	2	0	0	0	Protein FAM65A OS=Mus musculus GN=Fam65a PE=1 SV=2
F6W7I2	0.391002219	0	0	0	0	2	0	0	0	Deoxyhypusine synthase (Fragment) OS=Mus musculus GN=Dhps PE=1 SV=1
Q62432	0.391002219	0	0	0	0	0	0	0	2	Mothers against decapentaplegic homolog 2 OS=Mus musculus GN=Smad2 PE=1 SV=2
P20444	0.391002219	0	0	0	0	2	0	0	0	Protein kinase C alpha type OS=Mus musculus GN=Prkca PE=1 SV=3
E0CYI9	0.391002219	0	0	0	0	0	2	0	0	GRIP and coiled-coil domain-containing protein 2 OS=Mus musculus GN=Gcc2 PE=1 SV=1
Q9JHZ2	0.391002219	0	0	0	0	0	2	0	0	Progressive ankylosis protein OS=Mus musculus GN=Ankh PE=2 SV=1
Q5NCU3	0.391002219	0	0	0	0	2	0	0	0	SPARC (Fragment) OS=Mus musculus GN=Sparc PE=1 SV=1
E9Q2T8	0.391002219	0	0	0	0	0	0	5	0	EGF-containing fibulin-like extracellular matrix protein 2 (Fragment) OS=Mus musculus GN=Efemp2 PE=1 SV=1
Q5H8C4	0.391002219	0	0	0	0	2	0	0	0	Vacuolar protein sorting-associated protein 13A OS=Mus musculus GN=Vps13a PE=1 SV=1
Q3TXV4	0.391002219	0	0	0	0	3	0	0	0	Putative uncharacterized protein OS=Mus musculus GN=Rab31 PE=1 SV=1
Q6P1B9	0.391002219	0	0	0	0	5	0	0	0	Bin1 protein OS=Mus musculus GN=Bin1 PE=1 SV=1
Reverse_sp|Q14AT2|TEX11_MOUSE	0.391002219	0	0	0	0	2	0	0	0	Testis-expressed sequence 11 protein OS=Mus musculus GN=Tex11 PE=1 SV=1
E9Q828	0.391002219	0	0	0	0	5	0	0	0	Calcium-transporting ATPase OS=Mus musculus GN=Atp2b4 PE=1 SV=1
Q3TXU5	0.391002219	0	0	0	0	2	0	0	0	Deoxyhypusine synthase OS=Mus musculus GN=Dhps PE=1 SV=2
Reverse_tr|E9PZF0|E9PZF0_MOUSE	0.391002219	0	0	0	0	0	0	0	2	Nucleoside diphosphate kinase OS=Mus musculus GN=Gm20390 PE=3 SV=1
Q7TPW1	0.391002219	0	0	0	0	0	4	0	0	Nexilin OS=Mus musculus GN=Nexn PE=1 SV=3
E9PVW8	0.391002219	0	0	0	0	0	0	3	0	Receptor-type tyrosine-protein phosphatase delta OS=Mus musculus GN=Ptprd PE=1 SV=2
A0A0G2JFV3	0.391002219	0	0	0	0	0	0	0	2	Adhesion G protein-coupled receptor L2 OS=Mus musculus GN=Adgrl2 PE=1 SV=1
P30412	0.391002219	0	0	0	0	2	0	0	0	Peptidyl-prolyl cis-trans isomerase C OS=Mus musculus GN=Ppic PE=1 SV=1
E9Q7I7	0.391002219	0	0	0	0	0	0	3	0	Receptor-type tyrosine-protein phosphatase delta OS=Mus musculus GN=Ptprd PE=1 SV=2
P35235	0.391002219	0	0	0	0	2	0	0	0	Tyrosine-protein phosphatase non-receptor type 11 OS=Mus musculus GN=Ptpn11 PE=1 SV=2
A0A0A6YW53	0.391002219	0	0	0	0	3	0	0	0	Mitogen-activated protein kinase kinase kinase kinase 4 OS=Mus musculus GN=Map4k4 PE=1 SV=1
D3YZ57	0.391002219	0	0	0	0	0	0	0	5	Tyrosine-protein kinase OS=Mus musculus GN=Fyn PE=1 SV=1
Q3UJQ9	0.391002219	0	0	0	0	4	0	0	0	Succinyl-CoA:3-ketoacid-coenzyme A transferase OS=Mus musculus GN=Oxct1 PE=1 SV=1
A0A1D5RM94	0.391002219	0	0	0	0	0	0	0	2	Cytoplasmic dynein 1 light intermediate chain 2 OS=Mus musculus GN=Dync1li2 PE=1 SV=1
D3YYU8	0.391002219	0	0	0	0	0	0	0	4	Obscurin-like protein 1 OS=Mus musculus GN=Obsl1 PE=1 SV=1
E9PVM7	0.391002219	0	0	0	0	0	4	0	0	Glutathione S-transferase (Fragment) OS=Mus musculus GN=Gstm5 PE=1 SV=1
Reverse_sp|Q9R207|NBN_MOUSE	0.391002219	0	0	0	0	0	2	0	0	Nibrin OS=Mus musculus GN=Nbn PE=1 SV=1
D3Z2F8	0.391002219	0	0	0	0	0	8	0	0	Actin-related protein 2/3 complex subunit 3 OS=Mus musculus GN=Arpc3 PE=1 SV=1
Reverse_tr|E9PUU8|E9PUU8_MOUSE	0.391002219	0	0	0	0	0	2	0	0	Fibrocystin-L OS=Mus musculus GN=Pkhd1l1 PE=1 SV=1
O88351	0.391002219	0	0	0	0	2	0	0	0	Inhibitor of nuclear factor kappa-B kinase subunit beta OS=Mus musculus GN=Ikbkb PE=1 SV=1
E9Q806	0.391002219	0	0	0	0	0	0	3	0	Copine-1 OS=Mus musculus GN=Cpne1 PE=1 SV=1
D3Z282	0.391002219	0	0	0	0	0	0	2	0	RISC-loading complex subunit TARBP2 (Fragment) OS=Mus musculus GN=Tarbp2 PE=1 SV=1
P49817	0.391002219	0	0	0	0	0	0	0	4	Caveolin-1 OS=Mus musculus GN=Cav1 PE=1 SV=1
O88447	0.391002219	0	0	0	0	3	0	0	0	Kinesin light chain 1 OS=Mus musculus GN=Klc1 PE=1 SV=3
Q6ZQ12	0.391002219	0	0	0	0	0	0	2	0	Ninein-like protein OS=Mus musculus GN=Ninl PE=1 SV=3
P01887	0.391002219	0	0	0	0	0	4	0	0	Beta-2-microglobulin OS=Mus musculus GN=B2m PE=1 SV=2
A0A0A6YVY7	0.391002219	0	0	0	0	0	0	0	2	Ubiquitin carboxyl-terminal hydrolase 4 OS=Mus musculus GN=Usp4 PE=1 SV=1
A0A0G2JFF5	0.391002219	0	0	0	0	0	0	0	2	Adhesion G protein-coupled receptor L2 OS=Mus musculus GN=Adgrl2 PE=1 SV=1
Reverse_sp|P00520|ABL1_MOUSE	0.391002219	0	0	0	0	0	2	0	0	Tyrosine-protein kinase ABL1 OS=Mus musculus GN=Abl1 PE=1 SV=3
E9PWI6	0.391002219	0	0	0	0	0	2	0	0	GRIP and coiled-coil domain-containing protein 2 OS=Mus musculus GN=Gcc2 PE=1 SV=1
Q03958	0.391002219	0	0	0	0	2	0	0	0	Prefoldin subunit 6 OS=Mus musculus GN=Pfdn6 PE=1 SV=1
A0A0G2JG59	0.391002219	0	0	0	0	0	4	0	0	Nexilin (Fragment) OS=Mus musculus GN=Nexn PE=1 SV=1
O35083	0.391002219	0	0	0	0	0	0	0	3	1-acyl-sn-glycerol-3-phosphate acyltransferase alpha OS=Mus musculus GN=Agpat1 PE=1 SV=1
A0A0G2JG66	0.391002219	0	0	0	0	0	0	0	2	Adhesion G protein-coupled receptor L2 OS=Mus musculus GN=Adgrl2 PE=1 SV=1
Reverse_tr|E9Q9E7|E9Q9E7_MOUSE	0.391002219	0	0	0	0	0	2	0	0	Centriolin OS=Mus musculus GN=Cntrl PE=1 SV=2
Reverse_sp|P70227|ITPR3_MOUSE	0.391002219	0	0	0	0	0	0	0	2	Inositol 1,4,5-trisphosphate receptor type 3 OS=Mus musculus GN=Itpr3 PE=1 SV=3
Reverse_tr|R4GML3|R4GML3_MOUSE	0.391002219	0	0	0	0	0	2	0	0	Centriolin OS=Mus musculus GN=Cntrl PE=1 SV=2
Q3TQ39	0.391002219	0	0	0	0	2	0	0	0	Protein kinase C alpha type OS=Mus musculus GN=Prkca PE=1 SV=1
P21447	0.391002219	0	0	0	0	2	0	0	0	Multidrug resistance protein 1A OS=Mus musculus GN=Abcb1a PE=1 SV=3
Reverse_tr|H3BJG4|H3BJG4_MOUSE	0.391002219	0	0	0	0	0	0	0	3	Protein Trip11 (Fragment) OS=Mus musculus GN=Trip11 PE=1 SV=1
Q3U1U4	0.391002219	0	0	0	0	2	0	0	0	Integrin alpha-M OS=Mus musculus GN=Itgam PE=1 SV=1
A0A0A6YWM5	0.391002219	0	0	0	0	2	0	0	0	Rab3 GTPase-activating protein non-catalytic subunit OS=Mus musculus GN=Rab3gap2 PE=1 SV=1
Q9EPK2	0.391002219	0	0	0	0	0	0	6	0	Protein XRP2 OS=Mus musculus GN=Rp2 PE=1 SV=3
Q8BMJ3	0.391002219	0	0	0	0	2	0	0	0	Eukaryotic translation initiation factor 1A, X-chromosomal OS=Mus musculus GN=Eif1ax PE=2 SV=3
Reverse_sp|P70313|NOS3_MOUSE	0.391002219	0	0	0	0	3	0	0	0	Nitric oxide synthase, endothelial OS=Mus musculus GN=Nos3 PE=1 SV=4
P05555	0.391002219	0	0	0	0	2	0	0	0	Integrin alpha-M OS=Mus musculus GN=Itgam PE=1 SV=2
P52760	0.391002219	0	0	0	0	2	0	0	0	Ribonuclease UK114 OS=Mus musculus GN=Hrsp12 PE=1 SV=3
P51432	0.391002219	0	0	0	0	0	2	0	0	1-phosphatidylinositol 4,5-bisphosphate phosphodiesterase beta-3 OS=Mus musculus GN=Plcb3 PE=1 SV=2
B1ASP2	0.391002219	0	0	0	0	0	0	3	0	Tyrosine-protein kinase OS=Mus musculus GN=Jak1 PE=1 SV=1
H7BWX9	0.391002219	0	0	0	0	2	0	0	0	Small ubiquitin-related modifier 2 OS=Mus musculus GN=Sumo2 PE=1 SV=1
J3QQ16	0.391002219	0	0	0	0	0	0	0	15	Protein Col6a3 OS=Mus musculus GN=Col6a3 PE=1 SV=1
A0A0A6YWR8	0.391002219	0	0	0	0	3	0	0	0	Mitogen-activated protein kinase kinase kinase kinase 4 OS=Mus musculus GN=Map4k4 PE=1 SV=1
Reverse_tr|K3W4R0|K3W4R0_MOUSE	0.391002219	0	0	0	0	4	0	0	0	Dynein heavy chain 17, axonemal OS=Mus musculus GN=Dnah17 PE=1 SV=1
Reverse_tr|E9Q706|E9Q706_MOUSE	0.391002219	0	0	0	0	0	0	0	2	Adenylate cyclase type 9 OS=Mus musculus GN=Adcy9 PE=1 SV=1
A0A1D5RMC1	0.391002219	0	0	0	0	0	0	0	2	Cytoplasmic dynein 1 light intermediate chain 2 (Fragment) OS=Mus musculus GN=Dync1li2 PE=1 SV=1
A0A0G2JGN6	0.391002219	0	0	0	0	2	0	0	0	26S proteasome non-ATPase regulatory subunit 9 (Fragment) OS=Mus musculus GN=Psmd9 PE=1 SV=1
D3YXG0	0.391002219	0	0	0	0	0	4	0	0	Hemicentin-1 OS=Mus musculus GN=Hmcn1 PE=1 SV=1
Reverse_sp|A2AL36|CNTRL_MOUSE	0.391002219	0	0	0	0	0	2	0	0	Centriolin OS=Mus musculus GN=Cntrl PE=1 SV=2
A0A0G2JGM8	0.391002219	0	0	0	0	0	0	0	2	Adhesion G protein-coupled receptor L2 OS=Mus musculus GN=Adgrl2 PE=1 SV=1
G5E8F4	0.391002219	0	0	0	0	7	0	0	0	Fucose-1-phosphate guanylyltransferase OS=Mus musculus GN=Fpgt PE=1 SV=1
G5E8F1	0.391002219	0	0	0	0	2	0	0	0	Integrin alpha-M OS=Mus musculus GN=Itgam PE=1 SV=2
Reverse_tr|G3X928|G3X928_MOUSE	0.391002219	0	0	0	0	2	0	0	0	SEC23-interacting protein OS=Mus musculus GN=Sec23ip PE=1 SV=1
Q91WG4	0.391002219	0	0	0	0	0	3	0	0	Elongator complex protein 2 OS=Mus musculus GN=Elp2 PE=1 SV=1
A0A0G2JGI1	0.391002219	0	0	0	0	0	4	0	0	Nexilin OS=Mus musculus GN=Nexn PE=1 SV=1
Q8CHG3	0.391002219	0	0	0	0	0	2	0	0	GRIP and coiled-coil domain-containing protein 2 OS=Mus musculus GN=Gcc2 PE=1 SV=2
A0A140T8I9	0.391002219	0	0	0	0	0	0	0	2	Phosphatidylinositol 4-kinase alpha OS=Mus musculus GN=Pi4ka PE=1 SV=1
E9Q0M9	0.391002219	0	0	0	0	0	0	0	23	Protein Cald1 OS=Mus musculus GN=Cald1 PE=1 SV=1
Q61735	0.391002219	0	0	0	0	4	0	0	0	Leukocyte surface antigen CD47 OS=Mus musculus GN=Cd47 PE=1 SV=2
G5E8T0	0.391002219	0	0	0	0	3	0	0	0	DnaJ (Hsp40) homolog, subfamily C, member 5, isoform CRA_b OS=Mus musculus GN=Dnajc5 PE=1 SV=1
A0A0G2JH16	0.391002219	0	0	0	0	0	0	0	2	Adhesion G protein-coupled receptor L2 OS=Mus musculus GN=Adgrl2 PE=1 SV=1
Q3U141	0.391002219	0	0	0	0	2	0	0	0	Inhibitor of nuclear factor kappa-B kinase subunit beta OS=Mus musculus GN=Ikbkb PE=1 SV=1
E9PXB7	0.391002219	0	0	0	0	0	0	0	9	E3 ubiquitin-protein ligase NEDD4-like OS=Mus musculus GN=Nedd4l PE=1 SV=1
F8VPL5	0.391002219	0	0	0	0	3	0	0	0	Mitogen-activated protein kinase kinase kinase kinase 4 OS=Mus musculus GN=Map4k4 PE=1 SV=2
Reverse_sp|Q3UHU5|MTCL1_MOUSE	0.391002219	0	0	0	0	2	0	0	0	Microtubule cross-linking factor 1 OS=Mus musculus GN=Mtcl1 PE=1 SV=1
Q07235	0.391002219	0	0	0	0	5	0	0	0	Glia-derived nexin OS=Mus musculus GN=Serpine2 PE=1 SV=2
A0A0R4J196	0.391002219	0	0	0	0	0	2	0	0	C-Jun-amino-terminal kinase-interacting protein 4 OS=Mus musculus GN=Spag9 PE=1 SV=1
Reverse_sp|Q69Z23|DYH17_MOUSE	0.391002219	0	0	0	0	4	0	0	0	Dynein heavy chain 17, axonemal OS=Mus musculus GN=Dnah17 PE=1 SV=2
E9Q604	0.391002219	0	0	0	0	2	0	0	0	Integrin alpha M, isoform CRA_a OS=Mus musculus GN=Itgam PE=1 SV=2

Q9DCT1	0.391002219	0	0	0	0	0	0	0	2	1,5-anhydro-D-fructose reductase OS=Mus musculus GN=Akr1e2 PE=1 SV=1
Q62077	0.391002219	0	0	0	0	0	0	0	2	1-phosphatidylinositol 4,5-bisphosphate phosphodiesterase gamma-1 OS=Mus musculus GN=Plcg1 PE=1 SV=2
Q9QZZ4	0.391002219	0	0	0	0	0	0	0	2	Unconventional myosin-XV OS=Mus musculus GN=Myo15a PE=1 SV=2
P97314	0.391002219	0	0	0	0	0	0	0	4	Cysteine and glycine-rich protein 2 OS=Mus musculus GN=Csrp2 PE=1 SV=3
Q91V64	0.391002219	0	0	0	0	2	0	0	0	Isochorismatase domain-containing protein 1 OS=Mus musculus GN=Isoc1 PE=1 SV=1
A0A0R4J0T4	0.391002219	0	0	0	0	2	0	0	0	Inhibitor of nuclear factor kappa-B kinase subunit beta OS=Mus musculus GN=Ikbkb PE=1 SV=1
Q5SXG1	0.391002219	0	0	0	0	0	3	0	0	Misshapen-like kinase 1 OS=Mus musculus GN=Mink1 PE=1 SV=1
Q5SXG3	0.391002219	0	0	0	0	0	3	0	0	Misshapen-like kinase 1 OS=Mus musculus GN=Mink1 PE=1 SV=1
Q543K9	0.391002219	0	0	0	0	2	0	0	0	Purine nucleoside phosphorylase OS=Mus musculus GN=Pnp PE=1 SV=1
Reverse_tr|A0A0R4J0K2|A0A0R4J0K2_MOUSE	0.391002219	0	0	0	0	0	0	3	0	Cytoskeleton-associated protein 5 OS=Mus musculus GN=Ckap5 PE=1 SV=1
Reverse_tr|J3QMC5|J3QMC5_MOUSE	0.391002219	0	0	0	0	0	0	0	2	Protein Mdn1 OS=Mus musculus GN=Mdn1 PE=1 SV=1
Q8BLD4	0.391002219	0	0	0	0	0	0	4	0	MCG16669, isoform CRA_f OS=Mus musculus GN=Tardbp PE=1 SV=1
A9JEI5	0.391002219	0	0	0	0	0	2	0	0	Toll interacting protein, isoform CRA_b OS=Mus musculus GN=Tollip PE=1 SV=1
Q9CR00	0.391002219	0	0	0	0	2	0	0	0	26S proteasome non-ATPase regulatory subunit 9 OS=Mus musculus GN=Psmd9 PE=1 SV=1
E9Q5K8	0.391002219	0	0	0	0	2	0	0	0	Integrin alpha-M OS=Mus musculus GN=Itgam PE=1 SV=1
Reverse_sp|Q8BL66|EEA1_MOUSE	0.391002219	0	0	0	0	0	3	0	0	Early endosome antigen 1 OS=Mus musculus GN=Eea1 PE=1 SV=2
F8WH31	0.391002219	0	0	0	0	0	2	0	0	C-Jun-amino-terminal kinase-interacting protein 4 OS=Mus musculus GN=Spag9 PE=1 SV=2
F6SH61	0.391002219	0	0	0	0	0	2	0	0	C-Jun-amino-terminal kinase-interacting protein 4 (Fragment) OS=Mus musculus GN=Spag9 PE=1 SV=1
S4R1V0	0.391002219	0	0	0	0	3	0	0	0	PDZ and LIM domain protein 1 (Fragment) OS=Mus musculus GN=Pdlim1 PE=1 SV=1
S4R1T7	0.391002219	0	0	0	0	0	0	0	23	Protein Cald1 OS=Mus musculus GN=Cald1 PE=1 SV=1
A0A0G2JDF4	0.391002219	0	0	0	0	0	0	0	2	Adhesion G protein-coupled receptor L2 OS=Mus musculus GN=Adgrl2 PE=1 SV=1
Reverse_tr|Q91XZ9|Q91XZ9_MOUSE	0.391002219	0	0	0	0	0	0	3	0	Protein Pcdhb20 OS=Mus musculus GN=Pcdhb20 PE=1 SV=1
A0A0A0MQ87	0.391002219	0	0	0	0	2	0	0	0	Son of sevenless homolog 2 OS=Mus musculus GN=Sos2 PE=1 SV=1
A2AUE1	0.391002219	0	0	0	0	3	0	0	0	DnaJ homolog subfamily C member 5 (Fragment) OS=Mus musculus GN=Dnajc5 PE=1 SV=1
A0A0G2JDE3	0.391002219	0	0	0	0	0	0	0	2	Adhesion G protein-coupled receptor L2 OS=Mus musculus GN=Adgrl2 PE=1 SV=1
E9PUD1	0.391002219	0	0	0	0	0	2	0	0	C-Jun-amino-terminal kinase-interacting protein 4 OS=Mus musculus GN=Spag9 PE=1 SV=1
Reverse_sp|A2AAE1|K1109_MOUSE	0.391002219	0	0	0	0	2	0	0	0	Uncharacterized protein KIAA1109 OS=Mus musculus GN=Kiaa1109 PE=1 SV=4
Q9CZX0	0.391002219	0	0	0	0	4	0	0	0	Elongator complex protein 3 OS=Mus musculus GN=Elp3 PE=1 SV=1
A0A0A0MQG1	0.391002219	0	0	0	0	0	0	0	4	Obscurin-like protein 1 (Fragment) OS=Mus musculus GN=Obsl1 PE=1 SV=1
D3Z3V2	0.391002219	0	0	0	0	2	0	0	0	CMP-N-acetylneuraminate-beta-galactosamide-alpha-2,3-sialyltransferase 2 OS=Mus musculus GN=St3gal2 PE=1 SV=1
Q9Z2Y8	0.391002219	0	0	0	0	0	0	0	6	Proline synthase co-transcribed bacterial homolog protein OS=Mus musculus GN=Prosc PE=1 SV=1
O08915	0.391002219	0	0	0	0	0	2	0	0	AH receptor-interacting protein OS=Mus musculus GN=Aip PE=1 SV=1
Q9EP71	0.391002219	0	0	0	0	0	0	0	2	Ankycorbin OS=Mus musculus GN=Rai14 PE=1 SV=1
A0A0G2JDK6	0.391002219	0	0	0	0	0	0	0	2	Adhesion G protein-coupled receptor L2 OS=Mus musculus GN=Adgrl2 PE=1 SV=1
A0A0R4J088	0.391002219	0	0	0	0	0	3	0	0	Tissue factor OS=Mus musculus GN=F3 PE=1 SV=1
Q9JLV1	0.391002219	0	0	0	0	2	0	0	0	BAG family molecular chaperone regulator 3 OS=Mus musculus GN=Bag3 PE=1 SV=2
P97473	0.391002219	0	0	0	0	0	0	2	0	RISC-loading complex subunit TARBP2 OS=Mus musculus GN=Tarbp2 PE=1 SV=2
Q8R0B4	0.391002219	0	0	0	0	0	0	4	0	MCG16669, isoform CRA_a OS=Mus musculus GN=Tardbp PE=1 SV=1
P63213	0.391002219	0	0	0	0	0	0	0	3	Guanine nucleotide-binding protein G(I)/G(S)/G(O) subunit gamma-2 OS=Mus musculus GN=Gng2 PE=1 SV=2
Q9JM52	0.391002219	0	0	0	0	0	3	0	0	Misshapen-like kinase 1 OS=Mus musculus GN=Mink1 PE=1 SV=3
Q3V1G4	0.391002219	0	0	0	0	0	3	0	0	Olfactomedin-like protein 2B OS=Mus musculus GN=Olfml2b PE=1 SV=2
E9PV41	0.391002219	0	0	0	0	0	0	4	0	Protein diaphanous homolog 1 OS=Mus musculus GN=Diaph1 PE=1 SV=1
A0A0G2JEX1	0.391002219	0	0	0	0	0	4	0	0	Nexilin OS=Mus musculus GN=Nexn PE=1 SV=1
Reverse_sp|Q80TZ9|RERE_MOUSE	0.391002219	0	0	0	0	0	0	0	4	Arginine-glutamic acid dipeptide repeats protein OS=Mus musculus GN=Rere PE=1 SV=3
E9PV63	0.391002219	0	0	0	0	4	0	0	0	Glutathione S-transferase Mu 5 OS=Mus musculus GN=Gstm5 PE=1 SV=1
Q11204	0.391002219	0	0	0	0	2	0	0	0	CMP-N-acetylneuraminate-beta-galactosamide-alpha-2,3-sialyltransferase 2 OS=Mus musculus GN=St3gal2 PE=2 SV=2
A0A0G2JFD8	0.391002219	0	0	0	0	0	4	0	0	Nexilin OS=Mus musculus GN=Nexn PE=1 SV=1
P0DOV2	0.391002219	0	0	0	0	2	0	0	0	Interferon-activable protein 204 OS=Mus musculus GN=Ifi204 PE=1 SV=1
D3YZS5	0.391002219	0	0	0	0	0	0	2	0	RISC-loading complex subunit TARBP2 (Fragment) OS=Mus musculus GN=Tarbp2 PE=1 SV=1
G3UZK1	0.391002219	0	0	0	0	0	0	2	0	Guanine nucleotide-binding protein subunit gamma OS=Mus musculus GN=Gm20503 PE=3 SV=1
Reverse_sp|Q80ZA4|PKHL1_MOUSE	0.391002219	0	0	0	0	0	2	0	0	Fibrocystin-L OS=Mus musculus GN=Pkhd1l1 PE=1 SV=1
E9Q6J5	0.391002219	0	0	0	0	0	0	2	0	Biorientation of chromosomes in cell division protein 1-like 1 OS=Mus musculus GN=Bod1l PE=1 SV=1
Q00519	0.391002219	0	0	0	0	0	2	0	0	Xanthine dehydrogenase/oxidase OS=Mus musculus GN=Xdh PE=1 SV=5
Reverse_tr|E9PX09|E9PX09_MOUSE	0.391002219	0	0	0	0	0	0	0	2	Carnosine synthase 1 OS=Mus musculus GN=Carns1 PE=1 SV=1
G3UZM9	0.391002219	0	0	0	0	0	0	0	4	Serine/threonine-protein kinase N2 OS=Mus musculus GN=Pkn2 PE=1 SV=1
Q8VE98	0.391002219	0	0	0	0	0	0	0	2	CD276 antigen OS=Mus musculus GN=Cd276 PE=1 SV=1
Q9Z2D1	0.391002219	0	0	0	0	0	0	2	0	Myotubularin-related protein 2 OS=Mus musculus GN=Mtmr2 PE=1 SV=3
G3X9G2	0.391002219	0	0	0	0	0	3	0	0	Misshapen-like kinase 1 OS=Mus musculus GN=Mink1 PE=1 SV=1
Q3B7Z2	0.391002219	0	0	0	0	0	0	0	2	Oxysterol-binding protein 1 OS=Mus musculus GN=Osbp PE=1 SV=3
Q8C5G6	0.391002219	0	0	0	0	0	2	0	0	Putative uncharacterized protein OS=Mus musculus GN=Tollip PE=1 SV=1
Q9CYR6	0.391002219	0	0	0	0	3	0	0	0	Phosphoacetylglucosamine mutase OS=Mus musculus GN=Pgm3 PE=1 SV=1
J3QJX0	0.391002219	0	0	0	0	0	0	0	4	Cullin-4B OS=Mus musculus GN=Cul4b PE=1 SV=1
A2AVJ7	0.391002219	0	0	0	0	0	2	0	0	Ribosome-binding protein 1 OS=Mus musculus GN=Rrbp1 PE=1 SV=1
F8WI14	0.391002219	0	0	0	0	0	4	0	0	Extracellular matrix protein 1 OS=Mus musculus GN=Ecm1 PE=1 SV=1
Q8R180	0.391002219	0	0	0	0	0	0	0	2	ERO1-like protein alpha OS=Mus musculus GN=Ero1a PE=1 SV=2
A2A4E2	0.391002219	0	0	0	0	2	0	0	0	Protein Fam90a1a OS=Mus musculus GN=Fam90a1a PE=4 SV=1
Reverse_tr|E9Q7P0|E9Q7P0_MOUSE	0.391002219	0	0	0	0	4	0	0	0	Dynein heavy chain 17, axonemal OS=Mus musculus GN=Dnah17 PE=1 SV=1
S4R2T6	0.391002219	0	0	0	0	2	0	0	0	SH3 domain-binding protein 1 OS=Mus musculus GN=Sh3bp1 PE=1 SV=1
E9PVG7	0.391002219	0	0	0	0	3	0	0	0	Mitogen-activated protein kinase kinase kinase kinase 4 OS=Mus musculus GN=Map4k4 PE=1 SV=2
P60904	0.391002219	0	0	0	0	3	0	0	0	DnaJ homolog subfamily C member 5 OS=Mus musculus GN=Dnajc5 PE=1 SV=1
Reverse_tr|K3W4R5|K3W4R5_MOUSE	0.391002219	0	0	0	0	0	0	3	0	Cytoskeleton-associated protein 5 OS=Mus musculus GN=Ckap5 PE=1 SV=1
A2A4A6	0.391002219	0	0	0	0	0	0	0	2	Phosphoinositide phospholipase C OS=Mus musculus GN=Plcg1 PE=1 SV=1
Q9CQ92	0.391002219	0	0	0	0	0	3	0	0	Mitochondrial fission 1 protein OS=Mus musculus GN=Fis1 PE=1 SV=1
A0A0G2JEQ8	0.391002219	0	0	0	0	0	0	0	2	Adhesion G protein-coupled receptor L2 OS=Mus musculus GN=Adgrl2 PE=1 SV=1
Q02384	0.391002219	0	0	0	0	2	0	0	0	Son of sevenless homolog 2 OS=Mus musculus GN=Sos2 PE=1 SV=2
A0A0B4J1L4	0.391002219	0	0	0	0	0	0	0	4	Obscurin-like protein 1 OS=Mus musculus GN=Obsl1 PE=1 SV=1
Q9Z1Y4	0.391002219	0	0	0	0	3	0	0	0	Thyroid receptor-interacting protein 6 OS=Mus musculus GN=Trip6 PE=1 SV=1
G3X9U9	0.391002219	0	0	0	0	0	3	0	0	Fission 1 (Mitochondrial outer membrane) homolog (Yeast), isoform CRA_c OS=Mus musculus GN=Fis1 PE=1 SV=1
Q4KML4	0.391002219	0	0	0	0	3	0	0	0	Costars family protein ABRACL OS=Mus musculus GN=Abracl PE=1 SV=1
Q61508	0.391002219	0	0	0	0	0	4	0	0	Extracellular matrix protein 1 OS=Mus musculus GN=Ecm1 PE=1 SV=2
Q91ZX7	0.002094732	0	3	0	2	50	35	23	34	Prolow-density lipoprotein receptor-related protein 1 OS=Mus musculus GN=Lrp1 PE=1 SV=1
A0A0R4J0I9	0.002094732	0	3	0	2	50	35	23	34	Low density lipoprotein receptor-related protein 1 OS=Mus musculus GN=Lrp1 PE=1 SV=1
B9EHT6	0.099812452	0	35	47	14	501	362	0	316	Fibronectin OS=Mus musculus GN=Fn1 PE=1 SV=1
Q9WV92	0.00028565	0	4	0	0	48	51	29	48	Band 4.1-like protein 3 OS=Mus musculus GN=Epb41l3 PE=1 SV=1
A0A087WS56	0.007546296	48	34	45	13	518	384	169	326	Fibronectin OS=Mus musculus GN=Fn1 PE=1 SV=1
A0A087WR50	0.008514515	49	35	47	14	537	393	169	338	Fibronectin OS=Mus musculus GN=Fn1 PE=1 SV=1
P11276	0.008592762	49	35	47	14	537	394	169	337	Fibronectin OS=Mus musculus GN=Fn1 PE=1 SV=4
Q3UHL6	0.008242822	49	35	47	14	533	385	169	334	Fibronectin OS=Mus musculus GN=Fn1 PE=1 SV=1
E9Q3W4	0.000203089	21	15	20	5	191	148	108	138	Plectin OS=Mus musculus GN=Plec PE=1 SV=1
A0A087WSN6	0.009265727	47	35	47	14	505	370	157	318	Fibronectin OS=Mus musculus GN=Fn1 PE=1 SV=1
Q9QXS1	0.00020287	21	16	21	5	192	148	109	138	Plectin OS=Mus musculus GN=Plec PE=1 SV=3
O70318	0.000903752	7	6	3	2	41	37	27	49	Band 4.1-like protein 2 OS=Mus musculus GN=Epb41l2 PE=1 SV=2
P46935	0.000882051	7	4	4	2	36	23	23	35	E3 ubiquitin-protein ligase NEDD4 OS=Mus musculus GN=Nedd4 PE=1 SV=3
A2AKI5	0.036732805	4	2	0	3	28	19	20	15	Integrin alpha-V OS=Mus musculus GN=Itgav PE=1 SV=1
P43406	0.036732805	4	2	0	3	28	19	20	15	Integrin alpha-V OS=Mus musculus GN=Itgav PE=1 SV=2
Q05816	0.030910442	5	4	4	0	48	34	8	25	Fatty acid-binding protein, epidermal OS=Mus musculus GN=Fabp5 PE=1 SV=3
E9Q8Z9	0.128160884	0	5	4	2	27	0	17	27	Catenin delta-1 OS=Mus musculus GN=Ctnnd1 PE=1 SV=1
E9Q986	0.130281299	0	5	4	2	27	0	17	26	Catenin delta-1 OS=Mus musculus GN=Ctnnd1 PE=1 SV=1
E9Q8Z6	0.130281299	0	5	4	2	27	0	17	26	Catenin delta-1 OS=Mus musculus GN=Ctnnd1 PE=1 SV=1
E9Q901	0.130281299	0	5	4	2	27	0	17	26	Catenin delta-1 OS=Mus musculus GN=Ctnnd1 PE=1 SV=1
E9Q907	0.130281299	0	5	4	2	27	0	17	26	Catenin delta-1 OS=Mus musculus GN=Ctnnd1 PE=1 SV=1
G3X9V2	0.130281299	0	5	4	2	27	0	17	26	Catenin (Cadherin associated protein), delta 1, isoform CRA_a OS=Mus musculus GN=Ctnnd1 PE=1 SV=1
contaminant_KERATIN15	0.201776586	0	0	0	2	14	11	12	0	no description
Q8VHX6	3.99755E-05	33	28	33	10	199	155	116	162	Filamin-C OS=Mus musculus GN=Flnc PE=1 SV=3
D3Z2H2	0.002623066	0	5	4	2	27	18	17	26	Catenin delta-1 OS=Mus musculus GN=Ctnnd1 PE=1 SV=1
E9Q8Z8	0.002623066	0	5	4	2	27	18	17	26	Catenin delta-1 OS=Mus musculus GN=Ctnnd1 PE=1 SV=1
P30999	0.003751875	0	5	4	2	27	17	17	27	Catenin delta-1 OS=Mus musculus GN=Ctnnd1 PE=1 SV=2
E9Q905	0.003751875	0	5	4	2	27	17	17	27	Catenin delta-1 OS=Mus musculus GN=Ctnnd1 PE=1 SV=1
E9Q904	0.003804625	0	5	4	2	27	17	17	26	Catenin delta-1 OS=Mus musculus GN=Ctnnd1 PE=1 SV=1
D3Z7H6	0.00531301	0	5	4	2	27	16	17	27	Catenin delta-1 OS=Mus musculus GN=Ctnnd1 PE=1 SV=1
E9Q8Z4	0.00531301	0	5	4	2	27	16	17	27	Catenin delta-1 OS=Mus musculus GN=Ctnnd1 PE=1 SV=1
P11688	0.028181962	5	0	3	0	37	24	6	27	Integrin alpha-5 OS=Mus musculus GN=Itga5 PE=1 SV=3
E9Q903	0.309385994	0	5	4	2	0	16	0	25	Catenin delta-1 OS=Mus musculus GN=Ctnnd1 PE=1 SV=1
Q99K85	0.018362065	8	2	8	0	30	41	19	38	Phosphoserine aminotransferase OS=Mus musculus GN=Psat1 PE=1 SV=1
Q8BKG3	0.010103084	6	2	0	2	23	13	16	19	Inactive tyrosine-protein kinase 7 OS=Mus musculus GN=Ptk7 PE=1 SV=1
D3Z2H7	0.008558374	0	5	4	2	25	14	14	25	Catenin delta-1 OS=Mus musculus GN=Ctnnd1 PE=1 SV=1
E9Q8Z5	0.012336252	0	5	4	2	26	13	15	24	Catenin delta-1 OS=Mus musculus GN=Ctnnd1 PE=1 SV=1
Q64282	0.031492629	2	0	0	0	8	10	13	9	Interferon-induced protein with tetratricopeptide repeats 1 OS=Mus musculus GN=Ifit1 PE=1 SV=2
D3Z1V4	0.135841933	4	0	2	0	20	6	0	19	Phosphatidylethanolamine-binding protein 1 OS=Mus musculus GN=Pebp1 PE=1 SV=1
Q3U6K9	0.017099648	8	2	6	0	24	33	16	28	Phosphoserine aminotransferase OS=Mus musculus GN=Psat1 PE=1 SV=1
Q9DBC7	0.005440575	4	3	5	0	16	22	17	20	cAMP-dependent protein kinase type I-alpha regulatory subunit OS=Mus musculus GN=Prkar1a PE=1 SV=3
Q9D6X6	0.009669237	5	4	4	0	29	18	10	23	Serine protease 23 OS=Mus musculus GN=Prss23 PE=2 SV=2
P70296	0.119878119	6	0	2	0	23	9	0	23	Phosphatidylethanolamine-binding protein 1 OS=Mus musculus GN=Pebp1 PE=1 SV=3
Q9R0H5	0.277156706	0	0	0	3	15	11	14	0	Keratin, type II cytoskeletal 71 OS=Mus musculus GN=Krt71 PE=1 SV=1
H7BX99	0.03476965	4	2	3	0	11	16	7	19	Prothrombin OS=Mus musculus GN=F2 PE=1 SV=1
P19221	0.03476965	4	2	3	0	11	16	7	19	Prothrombin OS=Mus musculus GN=F2 PE=1 SV=1
A0A0N4SVS6	0.004042493	4	4	0	0	26	19	14	11	Cellular nucleic acid-binding protein OS=Mus musculus GN=Cnbp PE=1 SV=1
Q80X90	0.001244779	51	26	45	7	163	151	101	135	Filamin-B OS=Mus musculus GN=Flnb PE=1 SV=3
A2A816	0.248203635	2	0	0	0	0	0	7	10	Protein deglycase DJ-1 (Fragment) OS=Mus musculus GN=Park7 PE=1 SV=1
P09103	0.020890872	3	0	4	0	14	20	9	16	Protein disulfide-isomerase OS=Mus musculus GN=P4hb PE=1 SV=2
Q640N1	0.034718994	15	11	16	2	59	60	18	44	Adipocyte enhancer-binding protein 1 OS=Mus musculus GN=Aebp1 PE=1 SV=1
P27601	0.00323207	2	2	0	0	8	10	5	9	Guanine nucleotide-binding protein subunit alpha-13 OS=Mus musculus GN=Gna13 PE=1 SV=1
Q99LX0	0.039921807	2	0	0	0	4	8	7	13	Protein deglycase DJ-1 OS=Mus musculus GN=Park7 PE=1 SV=1
Reverse_tr|G5E866|G5E866_MOUSE	0.939635772	0	12	4	5	0	0	28	0	Splicing factor 3B subunit 1 OS=Mus musculus GN=Sf3b1 PE=1 SV=1
Reverse_sp|Q99NB9|SF3B1_MOUSE	0.939635772	0	12	4	5	0	0	28	0	Splicing factor 3B subunit 1 OS=Mus musculus GN=Sf3b1 PE=1 SV=1
A0A087WQK0	0.205896685	0	0	0	7	38	0	21	22	60S ribosomal protein L3 (Fragment) OS=Mus musculus GN=Rpl3 PE=1 SV=1
E9QN70	0.004383447	10	9	10	4	45	26	22	33	Laminin subunit beta-1 OS=Mus musculus GN=Lamb1 PE=1 SV=1
P02469	0.004383447	10	9	10	4	45	26	22	33	Laminin subunit beta-1 OS=Mus musculus GN=Lamb1 PE=1 SV=3
P50396	0.027087657	8	5	4	2	18	19	19	16	Rab GDP dissociation inhibitor alpha OS=Mus musculus GN=Gdi1 PE=1 SV=3
Q8K354	0.008318163	4	0	0	0	15	14	15	16	Carbonyl reductase [NADPH] 3 OS=Mus musculus GN=Cbr3 PE=1 SV=1
Q02248	0.001557465	12	11	13	4	50	35	25	38	Catenin beta-1 OS=Mus musculus GN=Ctnnb1 PE=1 SV=1
A2A813	0.044255444	2	0	0	0	4	6	7	12	Protein deglycase DJ-1 OS=Mus musculus GN=Park7 PE=1 SV=1
Q99LD8	0.015789468	0	5	4	0	24	15	9	17	N(G),N(G)-dimethylarginine dimethylaminohydrolase 2 OS=Mus musculus GN=Ddah2 PE=1 SV=1
Q99PT1	0.013658744	19	11	8	0	42	46	40	53	Rho GDP-dissociation inhibitor 1 OS=Mus musculus GN=Arhgdia PE=1 SV=3
Q5F2E8	0.007103036	2	0	0	0	8	7	7	6	Serine/threonine-protein kinase TAO1 OS=Mus musculus GN=Taok1 PE=1 SV=1
A2A815	0.16017177	2	0	0	0	3	0	7	11	Protein deglycase DJ-1 (Fragment) OS=Mus musculus GN=Park7 PE=1 SV=8
A2A817	0.502500613	2	0	0	0	0	0	7	0	Protein deglycase DJ-1 (Fragment) OS=Mus musculus GN=Park7 PE=1 SV=1
A0A0R4J1D0	0.753637865	2	0	2	0	0	0	0	7	Copine-2 OS=Mus musculus GN=Cpne2 PE=1 SV=1
P26645	0.152265174	0	0	3	0	20	3	8	0	Myristoylated alanine-rich C-kinase substrate OS=Mus musculus GN=Marcks PE=1 SV=2
Q07113	0.014478008	4	0	0	0	11	15	11	18	Cation-independent mannose-6-phosphate receptor OS=Mus musculus GN=Igf2r PE=1 SV=1
Q9CZN7	0.020165652	0	3	5	0	19	14	7	15	Serine hydroxymethyltransferase OS=Mus musculus GN=Shmt2 PE=1 SV=1
Q64727	0.019078436	23	14	17	7	85	46	28	48	Vinculin OS=Mus musculus GN=Vcl PE=1 SV=4
Q8VDJ3	0.012054101	3	4	3	0	13	9	10	13	Vigilin OS=Mus musculus GN=Hdlbp PE=1 SV=1
Q7TPR4	0.002739464	28	26	25	10	94	79	42	84	Alpha-actinin-1 OS=Mus musculus GN=Actn1 PE=1 SV=1
P20152	0.004728754	46	37	39	14	157	96	66	127	Vimentin OS=Mus musculus GN=Vim PE=1 SV=3
Q61206	0.362897083	2	0	2	0	7	0	0	6	Platelet-activating factor acetylhydrolase IB subunit beta OS=Mus musculus GN=Pafah1b2 PE=1 SV=2
Q6ZPJ3	0.676447881	0	0	0	2	7	6	0	0	(E3-independent) E2 ubiquitin-conjugating enzyme UBE2O OS=Mus musculus GN=Ube2o PE=1 SV=3
A0A087WP83	0.013247168	3	4	3	0	12	9	9	13	Vigilin OS=Mus musculus GN=Hdlbp PE=1 SV=1
F8VQJ3	0.002102524	36	33	36	9	112	105	61	84	Laminin subunit gamma-1 OS=Mus musculus GN=Lamc1 PE=1 SV=1
Q9D034	0.065982792	2	2	0	0	7	6	0	6	Guanine nucleotide-binding protein subunit alpha-13 OS=Mus musculus GN=Gna13 PE=1 SV=1
P26231	0.011904908	11	9	11	0	45	31	18	36	Catenin alpha-1 OS=Mus musculus GN=Ctnna1 PE=1 SV=1
A1BN54	0.004246363	28	26	24	10	89	72	37	78	Alpha actinin 1a OS=Mus musculus GN=Actn1 PE=1 SV=1
P15532	0.007111787	23	26	22	4	68	54	56	52	Nucleoside diphosphate kinase A OS=Mus musculus GN=Nme1 PE=1 SV=1
Q5NC80	0.007773212	22	26	22	4	65	54	56	51	Nucleoside diphosphate kinase (Fragment) OS=Mus musculus GN=Nme1 PE=1 SV=1
P10639	0.002332472	0	8	11	6	16	25	18	42	Thioredoxin OS=Mus musculus GN=Txn PE=1 SV=3

P02468	0.002943711	35	32	33	9	103	96	53	77 Laminin subunit gamma-1 OS=Mus musculus GN=Lamc1 PE=1 SV=2
A2A7S7	0.002754969	3	0	4	0	11	9	10	12 Tyrosine--tRNA ligase OS=Mus musculus GN=Yars PE=1 SV=1
Q91WQ3	0.002754969	3	0	4	0	11	9	10	12 Tyrosine--tRNA ligase, cytoplasmic OS=Mus musculus GN=Yars PE=1 SV=3
Q61072	0.044523102	0	3	3	0	5	7	12	12 Disintegrin and metalloproteinase domain-containing protein 9 OS=Mus musculus GN=Adam9 PE=1 SV=2
E9Q638	0.044523102	0	3	3	0	5	7	12	12 Disintegrin and metalloproteinase domain-containing protein 9 OS=Mus musculus GN=Adam9 PE=1 SV=1
A0A140LHU0	0.044523102	0	3	3	0	5	7	12	12 Disintegrin and metalloproteinase domain-containing protein 9 OS=Mus musculus GN=Adam9 PE=1 SV=1
Q9JJI8	0.063550309	0	2	0	0	6	6	0	6 60S ribosomal protein L38 OS=Mus musculus GN=Rpl38 PE=1 SV=3
H3BK84	0.212731185	2	2	2	0	7	5	0	6 cAMP-dependent protein kinase type II-beta regulatory subunit OS=Mus musculus GN=Prkar2b PE=1 SV=1
P48678	0.012733216	20	12	18	7	52	46	33	37 Prelamin-A/C OS=Mus musculus GN=Lmna PE=1 SV=2
Q6IRU2	0.005968047	17	18	18	9	51	44	38	48 Tropomyosin alpha-4 chain OS=Mus musculus GN=Tpm4 PE=1 SV=3
B7FAU9	0.004572122	99	73	99	20	304	195	138	212 Filamin, alpha OS=Mus musculus GN=Flna PE=1 SV=1
Q8BTM8	0.004572122	99	73	99	20	304	195	138	212 Filamin-A OS=Mus musculus GN=Flna PE=1 SV=5
B1AZ46	0.022450116	3	2	0	0	10	5	4	10 Brain-specific angiogenesis inhibitor 1-associated protein 2 OS=Mus musculus GN=Baiap2 PE=1 SV=1
Q8BKX1	0.022450116	3	2	0	0	10	5	4	10 Brain-specific angiogenesis inhibitor 1-associated protein 2 OS=Mus musculus GN=Baiap2 PE=1 SV=2
P0CG49	0.861490741	0	0	0	51	147	0	0	0 Polyubiquitin-B OS=Mus musculus GN=Ubb PE=2 SV=1
P06745	0.007105037	6	8	7	0	19	21	18	22 Glucose-6-phosphate isomerase OS=Mus musculus GN=Gpi PE=1 SV=4
Q5SX22	0.856428706	0	0	0	46	131	0	0	0 Polyubiquitin-B (Fragment) OS=Mus musculus GN=Ubb PE=4 SV=2
O09061	0.082808176	2	0	0	0	8	6	0	3 Proteasome subunit beta type-1 OS=Mus musculus GN=Psmb1 PE=1 SV=1
D3YVL0	0.092955025	0	0	2	0	0	5	8	4 Putative helicase MOV-10 OS=Mus musculus GN=Mov10 PE=1 SV=2
E9PW39	0.092955025	0	0	2	0	0	5	8	4 Putative helicase MOV-10 OS=Mus musculus GN=Mov10 PE=1 SV=1
P23249	0.092955025	0	0	2	0	0	5	8	4 Putative helicase MOV-10 OS=Mus musculus GN=Mov10 PE=1 SV=2
D3Z3E8	0.092955025	0	0	2	0	0	5	8	4 Putative helicase MOV-10 OS=Mus musculus GN=Mov10 PE=1 SV=1
Q80T06	0.039440948	2	5	5	0	16	7	10	12 Elongation factor 1-delta OS=Mus musculus GN=Eef1d PE=1 SV=1
P57776	0.039440948	2	5	5	0	16	7	10	12 Elongation factor 1-delta OS=Mus musculus GN=Eef1d PE=1 SV=3
A0A0R4J1E2	0.039440948	2	5	5	0	16	7	10	12 Elongation factor 1-delta OS=Mus musculus GN=Eef1d PE=1 SV=1
Q8VHM5	0.682030757	0	0	5	0	14	0	0	0 Heterogeneous nuclear ribonucleoprotein R OS=Mus musculus GN=Hnrnpr PE=1 SV=1
G3UXK0	0.794207453	3	0	2	0	0	7	0	0 Dystrobrevin OS=Mus musculus GN=Dtnb PE=1 SV=1
G3UY34	0.794207453	3	0	2	0	0	7	0	0 Dystrobrevin OS=Mus musculus GN=Dtnb PE=1 SV=1
G3UZJ3	0.794207453	3	0	2	0	0	7	0	0 Dystrobrevin beta OS=Mus musculus GN=Dtnb PE=1 SV=1
O88477	0.003378692	5	2	3	0	15	10	7	5 Insulin-like growth factor 2 mRNA-binding protein 1 OS=Mus musculus GN=Igf2bp1 PE=1 SV=1
P31324	0.233134288	4	3	2	0	9	6	0	10 cAMP-dependent protein kinase type II-beta regulatory subunit OS=Mus musculus GN=Prkar2b PE=1 SV=3
P19324	0.011396807	13	6	10	0	33	30	16	28 Serpin H1 OS=Mus musculus GN=Serpinh1 PE=1 SV=3
Q8BY89	0.017839346	11	8	8	2	18	20	17	25 Choline transporter-like protein 2 OS=Mus musculus GN=Slc44a2 PE=1 SV=2
Q920E5	0.008539605	0	0	2	0	8	5	6	3 Farnesyl pyrophosphate synthase OS=Mus musculus GN=Fdps PE=1 SV=1
E9QN08	0.038124294	2	5	5	0	15	7	10	12 Elongation factor 1-delta (Fragment) OS=Mus musculus GN=Eef1d PE=1 SV=1
P61028	0.307377623	2	0	0	0	0	5	0	6 Ras-related protein Rab-8B OS=Mus musculus GN=Rab8b PE=1 SV=1
P16045	0.016281761	62	21	51	5	100	84	83	111 Galectin-1 OS=Mus musculus GN=Lgals1 PE=1 SV=3
Q924C6	0.358213982	2	2	3	0	8	3	0	8 Lysyl oxidase homolog 4 OS=Mus musculus GN=Loxl4 PE=2 SV=2
E9Q600	0.358213982	2	2	3	0	8	3	0	8 Lysyl oxidase homolog 4 OS=Mus musculus GN=Loxl4 PE=1 SV=1
P05132	0.033724025	3	2	5	0	9	13	6	8 cAMP-dependent protein kinase catalytic subunit alpha OS=Mus musculus GN=Prkaca PE=1 SV=3
E9QNP0	0.945834642	0	0	0	46	0	0	124	0 KxDL motif-containing protein 1 OS=Mus musculus GN=Kxd1 PE=1 SV=1
E9Q616	0.014783589	29	25	21	14	54	53	51	81 Protein Ahnak OS=Mus musculus GN=Ahnak PE=1 SV=1
P62746	0.81142792	4	3	2	4	9	7	0	10 Rho-related GTP-binding protein RhoB OS=Mus musculus GN=Rhob PE=1 SV=1
Q60790	0.594717038	0	3	0	0	0	0	8	0 Ras GTPase-activating protein 3 OS=Mus musculus GN=Rasa3 PE=1 SV=2
P0C0A3	0.061974367	0	2	0	0	6	6	0	4 Charged multivesicular body protein 6 OS=Mus musculus GN=Chmp6 PE=1 SV=2
B1AZ42	0.061974367	0	2	0	0	6	6	0	4 Charged multivesicular body protein 6 (Fragment) OS=Mus musculus GN=Chmp6 PE=1 SV=1
Q6PI17	0.175724708	0	0	2	0	7	5	0	4 Metalloproteinase inhibitor 2 OS=Mus musculus GN=Timp2 PE=1 SV=1
P25785	0.175724708	0	0	2	0	7	5	0	4 Metalloproteinase inhibitor 2 OS=Mus musculus GN=Timp2 PE=1 SV=2
Q9D2R0	0.004781852	8	2	5	0	16	12	12	13 Acetoacetyl-CoA synthetase OS=Mus musculus GN=Aacs PE=1 SV=1
P13020	0.000191089	21	15	18	5	46	38	29	43 Gelsolin OS=Mus musculus GN=Gsn PE=1 SV=3
D3YUQ9	0.000167805	2	0	5	0	12	7	9	9 Elongation factor 1-delta (Fragment) OS=Mus musculus GN=Eef1d PE=1 SV=8
Q01768	0.014502381	22	20	22	2	48	38	39	49 Nucleoside diphosphate kinase B OS=Mus musculus GN=Nme2 PE=1 SV=1
Q922H4	0.877937905	0	4	2	2	0	7	0	7 Mannose-1-phosphate guanyltransferase alpha OS=Mus musculus GN=Gmppa PE=1 SV=1
E9PZF0	0.022298686	22	25	23	3	52	40	44	55 Nucleoside diphosphate kinase OS=Mus musculus GN=Gm20390 PE=3 SV=1
P97427	0.00916954	8	3	8	0	19	18	12	16 Dihydropyrimidinase-related protein 1 OS=Mus musculus GN=Crmp1 PE=1 SV=1
Z4YK85	0.426526658	41	31	43	5	94	72	0	62 Agrin OS=Mus musculus GN=Agrn PE=1 SV=1
E9Q6R7	0.003243697	0	0	2	0	6	3	5	6 Protein Utrn OS=Mus musculus GN=Utrn PE=1 SV=1
P68037	0.004129713	2	3	0	0	8	8	5	4 Ubiquitin-conjugating enzyme E2 L3 OS=Mus musculus GN=Ube2l3 PE=1 SV=1
Q6P5F7	0.014585274	2	3	0	0	7	6	5	7 Protein tweety homolog 3 OS=Mus musculus GN=Ttyh3 PE=1 SV=1
E9QNH6	0.025887518	0	2	2	0	4	6	3	7 Unconventional myosin-Ib OS=Mus musculus GN=Myo1b PE=1 SV=1
E9Q580	0.025887518	0	2	2	0	4	6	3	7 Unconventional myosin-Ib OS=Mus musculus GN=Myo1b PE=1 SV=1
P46735	0.025887518	0	2	2	0	4	6	3	7 Unconventional myosin-Ib OS=Mus musculus GN=Myo1b PE=1 SV=3
Q7TQD7	0.025887518	0	2	2	0	4	6	3	7 Myo1b protein OS=Mus musculus GN=Myo1b PE=1 SV=1
Q9CR16	0.038302929	12	8	8	6	31	16	14	24 Peptidyl-prolyl cis-trans isomerase D OS=Mus musculus GN=Ppid PE=1 SV=3
Q8BFY6	0.18372863	2	0	0	0	6	0	0	4 Peflin OS=Mus musculus GN=Pef1 PE=1 SV=1
P47199	0.718096995	0	0	2	0	5	0	0	0 Quinone oxidoreductase OS=Mus musculus GN=Cryz PE=1 SV=1
P50543	0.895562203	2	0	2	0	0	0	0	5 Protein S100-A11 OS=Mus musculus GN=S100a11 PE=1 SV=1
Q05793	0.009005813	400	325	327	143	983	731	531	695 Basement membrane-specific heparan sulfate proteoglycan core protein OS=Mus musculus GN=Hspg2 PE=1 SV=1
E9Q7Q3	0.084423853	0	0	27	13	61	51	41	43 Tropomyosin alpha-3 chain OS=Mus musculus GN=Tpm3 PE=1 SV=1
A2ASQ1	1.29728E-05	40	30	42	5	91	71	63	61 Agrin OS=Mus musculus GN=Agrn PE=1 SV=1
P61079	0.193881582	0	0	3	0	7	9	0	6 Ubiquitin-conjugating enzyme E2 D3 OS=Mus musculus GN=Ube2d3 PE=1 SV=1
A0A0G2JGL0	0.193881582	0	0	3	0	7	9	0	6 Ubiquitin-conjugating enzyme E2 D3 OS=Mus musculus GN=Ube2d3 PE=1 SV=1
A0A0G2JE32	0.193881582	0	0	3	0	7	9	0	6 Ubiquitin-conjugating enzyme E2 D3 (Fragment) OS=Mus musculus GN=Ube2d3 PE=1 SV=1
M0QWP1	0.000307749	44	32	44	5	98	72	66	69 Agrin OS=Mus musculus GN=Agrn PE=1 SV=1
O08553	0.044363487	45	34	36	4	91	81	34	83 Dihydropyrimidinase-related protein 2 OS=Mus musculus GN=Dpysl2 PE=1 SV=2
Q6NXH9	0.842948403	0	0	0	7	22	12	0	0 Keratin, type II cytoskeletal 73 OS=Mus musculus GN=Krt73 PE=1 SV=1
B1B0C7	0.005774716	470	369	367	154	1090	802	577	780 Basement membrane-specific heparan sulfate proteoglycan core protein OS=Mus musculus GN=Hspg2 PE=1 SV=1
Q9DBJ1	0.005286234	50	40	30	14	88	78	49	105 Phosphoglycerate mutase 1 OS=Mus musculus GN=Pgam1 PE=1 SV=3
Q5SF07	0.023864489	6	2	4	0	10	12	7	9 Insulin-like growth factor 2 mRNA-binding protein 2 OS=Mus musculus GN=Igf2bp2 PE=1 SV=1
E9PZ16	0.006178147	480	373	373	159	1100	808	579	792 Basement membrane-specific heparan sulfate proteoglycan core protein OS=Mus musculus GN=Hspg2 PE=1 SV=1
P27773	0.01867859	6	5	8	2	14	14	8	13 Protein disulfide-isomerase A3 OS=Mus musculus GN=Pdia3 PE=1 SV=2
P59108	0.648561657	3	0	0	0	0	0	0	7 Copine-2 OS=Mus musculus GN=Cpne2 PE=1 SV=1
contaminant_INT-STD1	0.071376351	397	290	364	270	775	728	643	932 BSA
P11679	0.241430789	20	14	20	10	37	0	0	0 Keratin, type II cytoskeletal 8 OS=Mus musculus GN=Krt8 PE=1 SV=4
Q99LB4	0.668788747	7	4	4	3	13	8	0	10 Capping protein (Actin filament), gelsolin-like OS=Mus musculus GN=Capg PE=1 SV=1
P40124	0.044783459	28	20	21	9	38	46	44	50 Adenylyl cyclase-associated protein 1 OS=Mus musculus GN=Cap1 PE=1 SV=4
Q8BU31	0.271381953	3	0	2	0	4	6	0	7 Ras-related protein Rap-2c OS=Mus musculus GN=Rap2c PE=1 SV=1
A6X8Z3	0.024269418	6	2	4	0	10	12	7	7 Insulin-like growth factor 2 mRNA-binding protein 2 OS=Mus musculus GN=Igf2bp2 PE=1 SV=1
P70349	0.027930054	4	6	8	0	22	12	10	10 Histidine triad nucleotide-binding protein 1 OS=Mus musculus GN=Hint1 PE=1 SV=3
Q62159	0.009530461	23	14	15	6	49	29	19	32 Rho-related GTP-binding protein RhoC OS=Mus musculus GN=Rhoc PE=1 SV=2
E9Q0P9	0.68610885	7	0	0	2	13	7	0	0 Serpin B6 (Fragment) OS=Mus musculus GN=Serpinb6a PE=1 SV=1
P26043	0.01477506	51	41	38	10	86	78	74	72 Radixin OS=Mus musculus GN=Rdx PE=1 SV=3
F7B5B5	0.335266818	6	3	5	0	14	7	0	10 Protein Hnrnpr OS=Mus musculus GN=Hnrnpr PE=1 SV=1
P26039	0.017462765	27	23	28	7	67	41	30	50 Talin-1 OS=Mus musculus GN=Tln1 PE=1 SV=2
K3W4R2	0.860016103	0	9	6	4	20	8	0	0 Myosin-14 OS=Mus musculus GN=Myh14 PE=1 SV=1
Q9R0P5	0.022736054	14	11	10	3	29	23	18	14 Destrin OS=Mus musculus GN=Dstn PE=1 SV=3
K7E6F1	0.65198429	8	0	0	2	14	8	0	0 Serpin B6 (Fragment) OS=Mus musculus GN=Serpinb6a PE=1 SV=1
Q9WUM3	0.050922558	13	6	10	0	16	27	18	24 Coronin-1B OS=Mus musculus GN=Coro1b PE=1 SV=1
Q924B0	0.064348852	4	3	5	0	14	8	4	9 Inositol (Myo)-1(Or 4)-monophosphatase 1 OS=Mus musculus GN=Impa1 PE=1 SV=1
Q80ZJ2	0.064348852	4	3	5	0	14	8	4	9 Impa1 protein OS=Mus musculus GN=Impa1 PE=1 SV=1
Q99L47	0.016949415	12	14	6	3	23	19	13	21 Hsc70-interacting protein OS=Mus musculus GN=St13 PE=1 SV=1
Q8R3C7	0.112300242	0	4	0	0	9	8	9	0 DEAD (Asp-Glu-Ala-Asp) box polypeptide 19b OS=Mus musculus GN=Ddx19b PE=1 SV=1
D3Z3B8	0.206145048	3	0	0	0	7	0	0	6 Disks large homolog 1 OS=Mus musculus GN=Dlg1 PE=1 SV=1
P14824	0.000780295	123	76	95	31	212	169	122	199 Annexin A6 OS=Mus musculus GN=Anxa6 PE=1 SV=3
D3Z6I8	0.07307509	26	25	27	13	61	51	41	43 Tropomyosin alpha-3 chain OS=Mus musculus GN=Tpm3 PE=1 SV=1
Q8C253	0.057682834	26	13	17	3	32	41	21	33 Galectin OS=Mus musculus GN=Lgals3 PE=1 SV=1
G3UYZ1	0.056359463	0	5	0	0	14	6	11	12 Immunoglobulin superfamily member 8 OS=Mus musculus GN=Igsf8 PE=1 SV=1
Q8R366	0.056359463	0	5	0	0	14	6	11	12 Immunoglobulin superfamily member 8 OS=Mus musculus GN=Igsf8 PE=1 SV=2
A0A0R4J117	0.056359463	0	5	0	0	14	6	11	12 Immunoglobulin superfamily member 8 OS=Mus musculus GN=Igsf8 PE=1 SV=1
Q792Z1	0.484429974	3	2	2	0	5	0	0	0 MCG140784 OS=Mus musculus GN=Try10 PE=1 SV=1
Z4YJS5	0.010353682	31	23	27	5	57	46	46	35 Agrin OS=Mus musculus GN=Agrn PE=1 SV=1
Q8CI94	0.162992508	11	8	7	4	25	12	4	23 Glycogen phosphorylase, brain form OS=Mus musculus GN=Pygb PE=1 SV=3
D3Z2H9	0.058939483	27	26	25	13	57	53	39	45 Protein Tpm3-rs7 OS=Mus musculus GN=Tpm3-rs7 PE=3 SV=1
Q9JJ00	0.010001185	0	0	2	0	3	4	4	6 Phospholipid scramblase 1 OS=Mus musculus GN=Plscr1 PE=1 SV=1
P62071	0.026161193	9	0	3	0	13	13	12	13 Ras-related protein R-Ras2 OS=Mus musculus GN=Rras2 PE=1 SV=1
A2AMW0	0.021410143	26	23	26	9	49	55	29	45 Capping protein (Actin filament) muscle Z-line, beta, isoform CRA_a OS=Mus musculus GN=Capzb PE=1 SV=1
A0A1B0GSR9	0.048280948	28	20	18	14	51	36	31	51 L-lactate dehydrogenase OS=Mus musculus GN=Ldha PE=1 SV=1
F8WJK8	0.02031791	12	14	6	4	23	19	13	21 Hsc70-interacting protein OS=Mus musculus GN=St13 PE=1 SV=1
P12367	0.399523715	2	4	3	0	8	5	0	6 cAMP-dependent protein kinase type II-alpha regulatory subunit OS=Mus musculus GN=Prkar2a PE=1 SV=2
A0A0J9YUL3	0.014622645	18	18	17	5	39	26	26	31 Septin 11, isoform CRA_b OS=Mus musculus GN=Sept11 PE=1 SV=1
Q8C1B7	0.014622645	18	18	17	5	39	26	26	31 Septin-11 OS=Mus musculus GN=Sept11 PE=1 SV=4
A0A0J9YTY0	0.014622645	18	18	17	5	39	26	26	31 Septin-11 OS=Mus musculus GN=Sept11 PE=1 SV=1
P16110	0.070641882	27	13	17	3	32	41	20	33 Galectin-3 OS=Mus musculus GN=Lgals3 PE=1 SV=3
P06151	0.045821003	28	20	19	14	51	36	31	51 L-lactate dehydrogenase A chain OS=Mus musculus GN=Ldha PE=1 SV=3
P24452	0.314916214	6	3	4	0	11	8	0	8 Macrophage-capping protein OS=Mus musculus GN=Capg PE=1 SV=2
Q564E2	0.043669846	28	20	20	14	52	36	31	51 L-lactate dehydrogenase OS=Mus musculus GN=Ldha PE=1 SV=1
Q60864	0.014640323	22	16	25	7	43	40	28	34 Stress-induced-phosphoprotein 1 OS=Mus musculus GN=Stip1 PE=1 SV=1
P15379	0.011007158	5	5	6	0	14	11	8	11 CD44 antigen OS=Mus musculus GN=Cd44 PE=1 SV=3
Q8R1F1	0.034373567	22	11	13	4	27	24	19	33 Niban-like protein 1 OS=Mus musculus GN=Fam129b PE=1 SV=2
contaminant_KERATIN04	0.66660403	3	10	0	4	7	19	9	0 no description
F8WGL3	0.003869876	67	60	61	17	132	95	86	108 Cofilin-1 OS=Mus musculus GN=Cfl1 PE=1 SV=1
contaminant_UBIQUITIN02	0.008719991	50	42	42	10	107	68	50	70 no description
F7AV51	0.708567719	0	9	8	7	4	20	25	0 Protein DBF4 homolog A (Fragment) OS=Mus musculus GN=Dbf4 PE=4 SV=1
Q9QZ41	0.708567719	0	9	8	7	4	20	25	0 Protein DBF4 homolog A OS=Mus musculus GN=Dbf4 PE=1 SV=1
D3YUG6	0.064141238	10	6	8	0	11	21	14	19 Coronin (Fragment) OS=Mus musculus GN=Coro1b PE=1 SV=1
P70303	0.276635638	10	4	0	3	6	10	15	15 CTP synthase 2 OS=Mus musculus GN=Ctps2 PE=1 SV=1
E9Q390	0.028963772	104	81	83	20	149	139	110	184 Myoferlin OS=Mus musculus GN=Myof PE=1 SV=2
A2A9M4	0.039854574	6	2	5	0	8	9	8	10 Dedicator of cytokinesis protein 7 OS=Mus musculus GN=Dock7 PE=1 SV=2
A2A9M5	0.039854574	6	2	5	0	8	9	8	10 Dedicator of cytokinesis protein 7 OS=Mus musculus GN=Dock7 PE=1 SV=1
A0A0U1RNK7	0.039854574	6	2	5	0	8	9	8	10 Dedicator of cytokinesis protein 7 OS=Mus musculus GN=Dock7 PE=1 SV=1
Q8R1A4	0.039854574	6	2	5	0	8	9	8	10 Dedicator of cytokinesis protein 7 OS=Mus musculus GN=Dock7 PE=1 SV=3
Q69ZN7	0.029905108	105	81	83	20	149	139	110	184 Myoferlin OS=Mus musculus GN=Myof PE=1 SV=2
P47757	0.017694696	26	23	26	9	46	51	29	43 F-actin-capping protein subunit beta OS=Mus musculus GN=Capzb PE=1 SV=3
Q9CQW9	0.007599624	0	5	8	0	16	13	11	12 Interferon-induced transmembrane protein 3 OS=Mus musculus GN=Ifitm3 PE=1 SV=1
D3YV80	0.014229372	0	2	2	0	3	5	3	5 Unconventional myosin-Ib (Fragment) OS=Mus musculus GN=Myo1b PE=1 SV=1
P13595	0.030679052	0	4	0	0	10	6	6	10 Neural cell adhesion molecule 1 OS=Mus musculus GN=Ncam1 PE=1 SV=3
E9QB01	0.030679052	0	4	0	0	10	6	6	10 Neural cell adhesion molecule 1 OS=Mus musculus GN=Ncam1 PE=1 SV=2
A0A0A6YY47	0.030679052	0	4	0	0	10	6	6	10 Neural cell adhesion molecule 1 OS=Mus musculus GN=Ncam1 PE=1 SV=1
Q61024	0.045048444	54	48	50	8	82	79	59	100 Asparagine synthetase [glutamine-hydrolyzing] OS=Mus musculus GN=Asns PE=1 SV=3
O55023	0.056506669	4	3	5	0	13	8	4	7 Inositol monophosphatase 1 OS=Mus musculus GN=Impa1 PE=1 SV=1
A0A0G2JDJ5	0.061609588	0	0	2	0	5	3	4	0 Farnesyl pyrophosphate synthase (Fragment) OS=Mus musculus GN=Fdps PE=4 SV=1
D3YX87	0.066898112	2	0	0	0	5	3	0	4 Protein misato homolog 1 OS=Mus musculus GN=Msto1 PE=1 SV=1
Q8C0M0	0.080821734	0	0	2	0	5	4	3	0 WD repeat-containing protein 59 OS=Mus musculus GN=Wdr59 PE=1 SV=2
A0A1D5RLJ8	0.080821734	0	0	2	0	5	4	3	0 WD repeat domain 59, isoform CRA_b OS=Mus musculus GN=Wdr59 PE=1 SV=1
D3Z1F9	0.080821734	0	0	2	0	5	4	3	0 WD repeat-containing protein 59 OS=Mus musculus GN=Wdr59 PE=1 SV=2
P62838	0.238130329	0	0	3	0	5	8	0	5 Ubiquitin-conjugating enzyme E2 D2 OS=Mus musculus GN=Ube2d2 PE=1 SV=1
O88738	0.391002219	0	0	2	0	0	0	4	0 Baculoviral IAP repeat-containing protein 6 OS=Mus musculus GN=Birc6 PE=1 SV=2
S4R1L5	0.391002219	0	0	2	0	0	0	4	0 Baculoviral IAP repeat-containing protein 6 OS=Mus musculus GN=Birc6 PE=1 SV=1
S4R2P8	0.391002219	0	0	2	0	0	0	4	0 Baculoviral IAP repeat-containing protein 6 OS=Mus musculus GN=Birc6 PE=1 SV=1
A0A0A0MQN8	0.798892371	0	0	3	0	6	0	0	0 Neuroplastin OS=Mus musculus GN=Nptn PE=1 SV=1
P62774	0.848114912	6	4	2	0	9	0	0	7 Myotrophin OS=Mus musculus GN=Mtpn PE=1 SV=2
A0A0J9YUD8	0.017141741	5	7	5	0	9	15	11	10 High mobility group protein B1 OS=Mus musculus GN=Hmgb1 PE=1 SV=1
A2APM5	0.023080029	5	5	7	0	14	11	8	12 CD44 antigen OS=Mus musculus GN=Cd44 PE=1 SV=1
Q80X37	0.023080029	5	5	7	0	14	11	8	12 CD44 antigen OS=Mus musculus GN=Cd44 PE=1 SV=1
P08730	0.217824834	10	8	11	4	19	23	14	9 Keratin, type I cytoskeletal 13 OS=Mus musculus GN=Krt13 PE=1 SV=2

O55131	0.01071421	28	22	34	7	63	39	36	41	Septin-7 OS=Mus musculus GN=Sept7 PE=1 SV=1
E9Q1G8	0.01071421	28	22	34	7	63	39	36	41	Septin-7 OS=Mus musculus GN=Sept7 PE=1 SV=2
E9Q9F5	0.01071421	28	22	34	7	63	39	36	41	Septin-7 OS=Mus musculus GN=Sept7 PE=1 SV=2
E9PX48	0.034680111	6	2	5	0	8	9	8	9	Dedicator of cytokinesis protein 7 OS=Mus musculus GN=Dock7 PE=1 SV=2
Q9CWK8	0.160833804	4	6	6	3	12	9	6	10	Sorting nexin-2 OS=Mus musculus GN=Snx2 PE=1 SV=2
D3Z5M7	0.769789584	5	6	3	5	17	7	5	8	Peroxidasin homolog OS=Mus musculus GN=Pxdn PE=1 SV=1
F7CAZ6	0.05592503	21	20	25	9	40	45	24	37	F-actin-capping protein subunit beta (Fragment) OS=Mus musculus GN=Capzb PE=1 SV=1
Q9JKF1	0.075501963	52	22	29	5	63	54	30	63	Ras GTPase-activating-like protein IQGAP1 OS=Mus musculus GN=Iqgap1 PE=1 SV=2
A2APM3	0.029385921	5	5	7	0	14	10	8	12	CD44 antigen OS=Mus musculus GN=Cd44 PE=1 SV=1
A2APM4	0.029385921	5	5	7	0	14	10	8	12	CD44 antigen OS=Mus musculus GN=Cd44 PE=1 SV=1
A2APM2	0.029385921	5	5	7	0	14	10	8	12	CD44 antigen OS=Mus musculus GN=Cd44 PE=1 SV=1
E9QKM8	0.029385921	5	5	7	0	14	10	8	12	CD44 antigen OS=Mus musculus GN=Cd44 PE=1 SV=1
Q3U8S1	0.029385921	5	5	7	0	14	10	8	12	CD44 antigen OS=Mus musculus GN=Cd44 PE=1 SV=1
Q9CXW3	0.399917211	7	3	5	0	9	12	0	8	Calcyclin-binding protein OS=Mus musculus GN=Cacybp PE=1 SV=1
P05064	0.028302261	65	75	60	26	133	113	84	105	Fructose-bisphosphate aldolase A OS=Mus musculus GN=Aldoa PE=1 SV=2
A2AMH3	0.296909448	4	0	4	0	8	8	0	7	Choline transporter-like protein 1 OS=Mus musculus GN=Slc44a1 PE=1 SV=1
A2AMH4	0.296909448	4	0	4	0	8	8	0	7	Choline transporter-like protein 1 OS=Mus musculus GN=Slc44a1 PE=1 SV=1
Q6X893	0.385119773	4	4	4	0	8	8	0	7	Choline transporter-like protein 1 OS=Mus musculus GN=Slc44a1 PE=1 SV=3
Q9CR57	0.010399706	9	5	6	0	15	13	11	12	60S ribosomal protein L14 OS=Mus musculus GN=Rpl14 PE=1 SV=3
G3V018	0.061536962	7	5	8	0	15	12	8	16	Heterogeneous nuclear ribonucleoprotein Q OS=Mus musculus GN=Syncrip PE=1 SV=1
A0A0R4J259	0.061536962	7	5	8	0	15	12	8	16	Heterogeneous nuclear ribonucleoprotein Q OS=Mus musculus GN=Syncrip PE=1 SV=1
G3UZI2	0.061536962	7	5	8	0	15	12	8	16	Heterogeneous nuclear ribonucleoprotein Q OS=Mus musculus GN=Syncrip PE=1 SV=1
Q7TMK9	0.061536962	7	5	8	0	15	12	8	16	Heterogeneous nuclear ribonucleoprotein Q OS=Mus musculus GN=Syncrip PE=1 SV=2
O09131	0.274581224	3	3	5	0	8	10	3	0	Glutathione S-transferase omega-1 OS=Mus musculus GN=Gsto1 PE=1 SV=2
Q8K1M3	0.428124176	3	4	3	0	8	5	0	6	Protein kinase, cAMP dependent regulatory, type II alpha OS=Mus musculus GN=Prkar2a PE=1 SV=1
A0A0A6YX73	0.428124176	3	4	3	0	8	5	0	6	cAMP-dependent protein kinase type II-alpha regulatory subunit OS=Mus musculus GN=Prkar2a PE=1 SV=1
A2APM1	0.022903385	5	5	7	0	14	10	8	11	CD44 antigen OS=Mus musculus GN=Cd44 PE=1 SV=1
Q07076	0.095121234	18	16	16	4	35	22	13	32	Annexin A7 OS=Mus musculus GN=Anxa7 PE=1 SV=2
Q9CQ65	0.057505843	26	17	24	3	43	37	19	33	S-methyl-5'-thioadenosine phosphorylase OS=Mus musculus GN=Mtap PE=1 SV=1
E9PYY0	0.973780872	6	5	4	2	10	5	0	9	Serpin B6 (Fragment) OS=Mus musculus GN=Serpinb6a PE=1 SV=1
P45377	0.096253019	19	11	16	0	26	28	20	41	Aldose reductase-related protein 2 OS=Mus musculus GN=Akr1b8 PE=1 SV=2
G3UZ48	0.082836467	7	5	8	0	15	12	7	16	Heterogeneous nuclear ribonucleoprotein Q OS=Mus musculus GN=Syncrip PE=1 SV=1
H3BKD0	0.738681855	0	0	28	3	0	30	0	28	Heterogeneous nuclear ribonucleoprotein K (Fragment) OS=Mus musculus GN=Hnrnpk PE=1 SV=1
P16546	0.021822929	21	12	18	3	29	26	19	27	Spectrin alpha chain, non-erythrocytic 1 OS=Mus musculus GN=Sptan1 PE=1 SV=4
contaminant_KERATIN08	0.159079157	21	12	31	4	46	41	24	16	no description
P14211	0.046847509	9	7	10	4	18	13	10	15	Calreticulin OS=Mus musculus GN=Calr PE=1 SV=1
E9Q5J9	0.206151137	20	24	24	13	44	43	28	36	Tropomyosin alpha-3 chain OS=Mus musculus GN=Tpm3 PE=1 SV=1
P14069	0.00893839	31	24	30	6	56	41	40	32	Protein S100-A6 OS=Mus musculus GN=S100a6 PE=1 SV=3
O54962	0.316121859	6	5	6	3	14	10	5	8	Barrier-to-autointegration factor OS=Mus musculus GN=Banf1 PE=1 SV=1
Q9R0P9	0.078833087	0	0	2	0	4	4	3	0	Ubiquitin carboxyl-terminal hydrolase isozyme L1 OS=Mus musculus GN=Uchl1 PE=1 SV=1
P61759	0.087386179	2	0	0	0	4	3	0	4	Prefoldin subunit 3 OS=Mus musculus GN=Vbp1 PE=1 SV=2
Q5XJY5	0.569012531	0	0	0	2	4	3	0	4	Coatomer subunit delta OS=Mus musculus GN=Arcn1 PE=1 SV=2
Q8JZV7	0.028675804	2	0	4	0	7	6	4	5	N-acetylglucosamine-6-phosphate deacetylase OS=Mus musculus GN=Amdhd2 PE=1 SV=1
Q9CX00	0.301214949	9	0	2	3	9	10	7	8	IST1 homolog OS=Mus musculus GN=Ist1 PE=1 SV=1
A0A0A6YY91	0.02760317	0	4	0	0	9	6	6	8	Neural cell adhesion molecule 1 (Fragment) OS=Mus musculus GN=Ncam1 PE=1 SV=1
P21107	0.300128128	19	23	23	13	42	40	26	33	Tropomyosin alpha-3 chain OS=Mus musculus GN=Tpm3 PE=1 SV=3
Q62261	0.077187758	28	26	28	14	43	47	35	48	Spectrin beta chain, non-erythrocytic 1 OS=Mus musculus GN=Sptbn1 PE=1 SV=2
Q02013	0.480200386	4	3	3	0	7	4	0	7	Aquaporin-1 OS=Mus musculus GN=Aqp1 PE=1 SV=3
G3UZW7	0.645309458	3	4	3	0	0	0	0	6	Protein mago nashi homolog 2 OS=Mus musculus GN=Magohb PE=4 SV=1
Q9Z2K1	0.27757977	9	7	17	2	23	22	10	8	Keratin, type I cytoskeletal 16 OS=Mus musculus GN=Krt16 PE=1 SV=3
B7ZCP4	0.516085314	3	2	0	0	6	0	3	0	Copine-1 OS=Mus musculus GN=Cpne1 PE=1 SV=1
Q99M73	0.890996938	0	5	0	0	9	0	0	0	Keratin, type II cuticular Hb4 OS=Mus musculus GN=Krt84 PE=2 SV=2
Q5SVG4	0.154426357	8	5	0	0	10	13	0	12	AP-1 complex subunit beta-1 OS=Mus musculus GN=Ap1b1 PE=1 SV=1
Q5SVG5	0.154426357	8	5	0	0	10	13	0	12	AP-1 complex subunit beta-1 OS=Mus musculus GN=Ap1b1 PE=1 SV=1
Q3U4W8	0.196794507	9	6	15	3	17	12	9	21	Ubiquitinyl hydrolase 1 OS=Mus musculus GN=Usp5 PE=1 SV=1
P56399	0.196794507	9	6	15	3	17	12	9	21	Ubiquitin carboxyl-terminal hydrolase 5 OS=Mus musculus GN=Usp5 PE=1 SV=1
Q3TTY5	0.596515309	12	5	21	9	32	20	18	14	Keratin, type II cytoskeletal 2 epidermal OS=Mus musculus GN=Krt2 PE=1 SV=1
Q8VIJ6	0.035439802	11	7	12	2	13	15	15	14	Splicing factor, proline- and glutamine-rich OS=Mus musculus GN=Sfpq PE=1 SV=1
A2AMH5	0.433224961	4	5	4	0	8	8	0	7	Choline transporter-like protein 1 OS=Mus musculus GN=Slc44a1 PE=1 SV=1
P56480	0.501156992	5	3	5	0	12	5	0	6	ATP synthase subunit beta, mitochondrial OS=Mus musculus GN=Atp5b PE=1 SV=2
P68181	0.249893822	3	2	6	0	2	11	4	9	cAMP-dependent protein kinase catalytic subunit beta OS=Mus musculus GN=Prkacb PE=1 SV=2
Q3TMX0	0.089179587	16	14	0	4	24	16	12	28	MCG4375, isoform CRA_b OS=Mus musculus GN=Sdcbp PE=1 SV=1
F6RV17	0.978609728	6	4	7	0	11	0	0	9	Serine/threonine-protein phosphatase 2A 55 kDa regulatory subunit B OS=Mus musculus GN=Ppp2r2d PE=1 SV=2
contaminant_KERATIN20	0.529319647	14	15	19	10	38	23	24	17	no description
B7ZCU0	0.001425764	0	2	4	0	4	7	6	4	Abl interactor 1 OS=Mus musculus GN=Abi1 PE=1 SV=1
B7ZCU2	0.001425764	0	2	4	0	4	7	6	4	Abl interactor 1 OS=Mus musculus GN=Abi1 PE=1 SV=1
B7ZCU3	0.001425764	0	2	4	0	4	7	6	4	Abl interactor 1 OS=Mus musculus GN=Abi1 PE=1 SV=1
B7ZCU4	0.001425764	0	2	4	0	4	7	6	4	Abl interactor 1 OS=Mus musculus GN=Abi1 PE=1 SV=1
B7ZCU5	0.001425764	0	2	4	0	4	7	6	4	Abl interactor 1 OS=Mus musculus GN=Abi1 PE=1 SV=1
Q8CBW3	0.001425764	0	2	4	0	4	7	6	4	Abl interactor 1 OS=Mus musculus GN=Abi1 PE=1 SV=3
J3QNK8	0.001425764	0	2	4	0	4	7	6	4	Abl interactor 1 OS=Mus musculus GN=Abi1 PE=1 SV=1
O88783	0.217537216	0	0	2	0	0	4	3	0	Coagulation factor V OS=Mus musculus GN=F5 PE=1 SV=1
A0A0A0MQG2	0.115025548	28	26	27	14	41	44	35	46	Spectrin beta chain, non-erythrocytic 1 (Fragment) OS=Mus musculus GN=Sptbn1 PE=1 SV=1
contaminant_KERATIN02	0.840154152	43	51	96	45	210	104	39	56	no description
Q9DAS9	0.105946732	4	4	8	0	6	10	8	13	Guanine nucleotide-binding protein G(I)/G(S)/G(O) subunit gamma-12 OS=Mus musculus GN=Gng12 PE=1 SV=3
P61205	0.066086698	20	12	9	4	22	18	13	25	ADP-ribosylation factor 3 OS=Mus musculus GN=Arf3 PE=2 SV=2
Q6ZWZ7	0.090491295	18	10	9	4	23	14	16	18	60S ribosomal protein L17 OS=Mus musculus GN=Rpl17 PE=1 SV=1
contaminant_KERATIN14	0.734712602	7	4	12	6	20	12	10	8	no description
Q6ZWV3	0.418762282	13	14	10	6	30	15	14	15	60S ribosomal protein L10 OS=Mus musculus GN=Rpl10 PE=1 SV=3
Q9WUD1	0.112135376	2	0	5	0	5	9	4	6	STIP1 homology and U box-containing protein 1 OS=Mus musculus GN=Stub1 PE=1 SV=1
P15331	0.34114082	0	4	3	0	7	6	0	5	Peripherin OS=Mus musculus GN=Prph PE=1 SV=2
S4R1C4	0.831957707	0	4	3	0	0	0	0	6	Calcium-transporting ATPase OS=Mus musculus GN=Atp2b2 PE=1 SV=1
Q9ESX5	0.032023431	11	10	11	0	22	15	18	18	H/ACA ribonucleoprotein complex subunit 4 OS=Mus musculus GN=Dkc1 PE=1 SV=4
Q9CPR4	0.072673605	18	10	9	4	23	14	15	18	60S ribosomal protein L17 OS=Mus musculus GN=Rpl17 PE=1 SV=3
Q8C052	0.021685248	12	12	6	4	16	16	9	17	Microtubule-associated protein 1S OS=Mus musculus GN=Map1s PE=1 SV=2
P03991	0.177085052	4	3	4	0	6	5	4	10	H-2 class I histocompatibility antigen, K-W28 alpha chain OS=Mus musculus GN=H2-K1 PE=1 SV=2
P86048	0.527034526	11	11	9	6	24	13	14	12	60S ribosomal protein L10-like OS=Mus musculus GN=Rpl10l PE=2 SV=1
Q9EST5	0.212081797	0	3	2	0	4	3	2	8	Acidic leucine-rich nuclear phosphoprotein 32 family member B OS=Mus musculus GN=Anp32b PE=1 SV=1
Q9D8B3	0.216208886	15	14	9	5	15	20	18	20	Charged multivesicular body protein 4b OS=Mus musculus GN=Chmp4b PE=1 SV=2
contaminant_KERATIN13	0.344621769	79	73	133	34	223	121	99	97	no description
P60335	0.177535438	22	11	17	5	34	27	12	20	Poly(rC)-binding protein 1 OS=Mus musculus GN=Pcbp1 PE=1 SV=1
Q61414	0.099515381	16	8	18	3	24	23	17	12	Keratin, type I cytoskeletal 15 OS=Mus musculus GN=Krt15 PE=1 SV=2
A0A0N4SVT3	0.117647009	4	4	8	0	6	9	8	13	Guanine nucleotide-binding protein subunit gamma (Fragment) OS=Mus musculus GN=Gng12 PE=1 SV=1
A0A0N4SW28	0.117647009	4	4	8	0	6	9	8	13	Guanine nucleotide-binding protein subunit gamma OS=Mus musculus GN=Gng12 PE=1 SV=1
P53994	0.530502691	9	12	7	6	15	5	0	23	Ras-related protein Rab-2A OS=Mus musculus GN=Rab2a PE=1 SV=1
Q9JMH6	0.689804096	0	3	2	3	5	0	4	0	Thioredoxin reductase 1, cytoplasmic OS=Mus musculus GN=Txnrd1 PE=1 SV=3
Q61239	0.889616466	3	3	2	0	0	6	0	3	Protein farnesyltransferase/geranylgeranyltransferase type-1 subunit alpha OS=Mus musculus GN=Fnta PE=1 SV=1
Q3TLP8	0.103777105	49	28	42	10	47	53	58	59	Putative uncharacterized protein OS=Mus musculus GN=Rac1 PE=1 SV=1
P29341	0.096063561	32	20	25	4	41	44	20	31	Polyadenylate-binding protein 1 OS=Mus musculus GN=Pabpc1 PE=1 SV=2
Q99MN1	0.147289486	38	18	25	3	43	35	21	42	Lysine--tRNA ligase OS=Mus musculus GN=Kars PE=1 SV=1
P62631	0.028515588	144	109	133	38	193	198	158	162	Elongation factor 1-alpha 2 OS=Mus musculus GN=Eef1a2 PE=1 SV=1
contaminant_KERATIN17	0.825334686	27	21	41	24	77	52	35	25	no description
Q3UPL0	0.214235166	19	13	22	7	35	25	14	28	Protein transport protein Sec31A OS=Mus musculus GN=Sec31a PE=1 SV=2
H7BWY4	0.088242453	3	0	0	0	7	0	2	6	Disks large homolog 1 OS=Mus musculus GN=Dlg1 PE=1 SV=1
E9Q9H0	0.088242453	3	0	0	0	7	0	2	6	Disks large homolog 1 OS=Mus musculus GN=Dlg1 PE=1 SV=1
Q811D0	0.088242453	3	0	0	0	7	0	2	6	Disks large homolog 1 OS=Mus musculus GN=Dlg1 PE=1 SV=1
P10833	0.160783849	3	0	0	0	4	4	0	7	Ras-related protein R-Ras OS=Mus musculus GN=Rras PE=1 SV=1
P42227	0.580447993	6	3	8	4	5	12	9	9	Signal transducer and activator of transcription 3 OS=Mus musculus GN=Stat3 PE=1 SV=2
P39688	0.819180041	0	0	3	0	0	0	0	5	Tyrosine-protein kinase Fyn OS=Mus musculus GN=Fyn PE=1 SV=4
A0A087WNM1	0.07797806	2	0	0	0	4	3	0	3	SLIT-ROBO Rho GTPase-activating protein 2 OS=Mus musculus GN=Srgap2 PE=1 SV=1
Q9R1P0	0.128356855	0	2	0	0	3	3	0	4	Proteasome subunit alpha type-4 OS=Mus musculus GN=Psma4 PE=1 SV=1
E9PW69	0.128356855	0	2	0	0	3	3	0	4	Proteasome subunit alpha type (Fragment) OS=Mus musculus GN=Psma4 PE=1 SV=1
Q91Z67	0.876907505	2	0	0	2	4	3	0	3	SLIT-ROBO Rho GTPase-activating protein 2 OS=Mus musculus GN=Srgap2 PE=1 SV=2
Q8BP43	0.634292927	28	30	26	15	50	39	0	34	Putative uncharacterized protein OS=Mus musculus GN=Tpm1 PE=1 SV=1
Q8R2P8	0.147745807	38	18	25	3	43	34	21	41	Lysine--tRNA ligase OS=Mus musculus GN=Kars PE=1 SV=1
P10126	0.012180774	291	199	255	75	449	342	250	315	Elongation factor 1-alpha 1 OS=Mus musculus GN=Eef1a1 PE=1 SV=3
Q9D0E1	0.288404106	10	4	6	3	11	11	7	9	Heterogeneous nuclear ribonucleoprotein M OS=Mus musculus GN=Hnrnpm PE=1 SV=3
I7HLV2	0.444468961	13	14	10	6	27	15	14	15	60S ribosomal protein L10 (Fragment) OS=Mus musculus GN=Rpl10 PE=1 SV=1
Q9DBG3	0.136313481	8	5	7	0	10	14	6	14	AP-2 complex subunit beta OS=Mus musculus GN=Ap2b1 PE=1 SV=1
Q60854	0.165646251	12	7	13	2	20	13	8	15	Serpin B6 OS=Mus musculus GN=Serpinb6 PE=1 SV=1
F8WIV2	0.165646251	12	7	13	2	20	13	8	15	Serpin B6 OS=Mus musculus GN=Serpinb6a PE=1 SV=1
Q922U2	0.744961278	30	26	38	21	76	52	40	21	Keratin, type II cytoskeletal 5 OS=Mus musculus GN=Krt5 PE=1 SV=1
P97372	0.076879192	4	6	6	0	5	10	10	10	Proteasome activator complex subunit 2 OS=Mus musculus GN=Psme2 PE=1 SV=4
Q9QUM9	0.698214632	7	5	8	5	13	10	10	8	Proteasome subunit alpha type-6 OS=Mus musculus GN=Psma6 PE=1 SV=1
Q9CQL1	0.591069678	3	4	4	0	0	0	0	6	Protein mago nashi homolog 2 OS=Mus musculus GN=Magohb PE=2 SV=1
P61327	0.591069678	3	4	4	0	0	0	0	6	Protein mago nashi homolog OS=Mus musculus GN=Magoh PE=2 SV=1
A0A023T778	0.591069678	3	4	4	0	0	0	0	6	Mago nashi protein OS=Mus musculus GN=Magohb PE=1 SV=1
P17426	0.030555305	8	5	4	0	13	10	5	9	AP-2 complex subunit alpha-1 OS=Mus musculus GN=Ap2a1 PE=1 SV=1
P01897	0.132116947	6	6	5	0	12	8	5	12	H-2 class I histocompatibility antigen, L-D alpha chain OS=Mus musculus GN=H2-L PE=1 SV=2
O88342	0.007333964	27	20	21	5	39	30	20	30	WD repeat-containing protein 1 OS=Mus musculus GN=Wdr1 PE=1 SV=3
Q91XH5	0.16058759	10	9	10	3	18	9	9	16	Sepiapterin reductase OS=Mus musculus GN=Spr PE=1 SV=1
Q6IFZ6	0.363773647	22	21	25	10	45	28	29	24	Keratin, type II cytoskeletal 1b OS=Mus musculus GN=Krt77 PE=1 SV=1
E9Q3M3	0.12449409	0	4	5	0	9	6	4	10	Dynactin subunit 1 OS=Mus musculus GN=Dctn1 PE=1 SV=1
O08788	0.12449409	0	4	5	0	9	6	4	10	Dynactin subunit 1 OS=Mus musculus GN=Dctn1 PE=1 SV=3
E9Q586	0.12449409	0	4	5	0	9	6	4	10	Dynactin subunit 1 OS=Mus musculus GN=Dctn1 PE=1 SV=1
D3YX34	0.12449409	0	4	5	0	9	6	4	10	Dynactin subunit 1 OS=Mus musculus GN=Dctn1 PE=1 SV=1
Q9D8E6	0.084987879	33	25	27	7	50	34	21	43	60S ribosomal protein L4 OS=Mus musculus GN=Rpl4 PE=1 SV=3
Q99J77	0.1846073	22	13	14	2	23	22	12	25	Sialic acid synthase OS=Mus musculus GN=Nans PE=1 SV=1
Q6IFX2	0.538846248	7	6	12	0	21	16	3	0	Keratin, type I cytoskeletal 42 OS=Mus musculus GN=Krt42 PE=1 SV=1
Q8C166	0.139233085	3	2	0	0	6	3	3	0	Copine-1 OS=Mus musculus GN=Cpne1 PE=1 SV=1
P60605	0.778359223	0	2	3	0	5	3	0	0	Ubiquitin-conjugating enzyme E2 G2 OS=Mus musculus GN=Ube2g2 PE=1 SV=1
G3UZX2	0.778359223	0	2	3	0	5	3	0	0	Ubiquitin-conjugating enzyme E2 G2 (Fragment) OS=Mus musculus GN=Ube2g2 PE=1 SV=1
P14115	0.605812595	8	7	5	0	16	5	0	11	60S ribosomal protein L27a OS=Mus musculus GN=Rpl27a PE=1 SV=5
P58771	0.508733656	28	30	24	15	50	39	32	34	Tropomyosin alpha-1 chain OS=Mus musculus GN=Tpm1 PE=1 SV=1
Q64105	0.159161762	10	9	10	3	17	9	9	16	Sepiapterin reductase OS=Mus musculus GN=Spr PE=1 SV=1
Q8BSH3	0.399219925	28	30	0	15	50	39	32	34	Putative uncharacterized protein OS=Mus musculus GN=Tpm1 PE=1 SV=1
E9Q454	0.399219925	28	30	0	15	50	39	32	34	Tropomyosin alpha-1 chain OS=Mus musculus GN=Tpm1 PE=1 SV=1
E9Q452	0.399219925	28	30	0	15	50	39	32	34	Tropomyosin alpha-1 chain OS=Mus musculus GN=Tpm1 PE=1 SV=1
A2A5N1	0.044372222	21	8	17	5	25	18	16	22	14-3-3 protein beta/alpha (Fragment) OS=Mus musculus GN=Ywhab PE=1 SV=1
B8JK32	0.078576623	10	3	4	0	10	11	7	8	Heterogeneous nuclear ribonucleoprotein M OS=Mus musculus GN=Hnrnpm PE=1 SV=1
Q7TSG6	0.033598986	50	39	38	0	69	72	65	62	Radixin OS=Mus musculus GN=Rdx PE=1 SV=1
O35643	0.13599819	8	5	6	0	10	13	5	12	AP-1 complex subunit beta-1 OS=Mus musculus GN=Ap1b1 PE=1 SV=2
O35988	0.448050982	7	0	0	0	0	7	0	15	Syndecan-4 OS=Mus musculus GN=Sdc4 PE=1 SV=1
Q9QYB1	0.56883375	28	23	26	14	51	37	23	32	Chloride intracellular channel protein 4 OS=Mus musculus GN=Clic4 PE=1 SV=3
P67984	0.612119828	4	3	0	0	7	0	4	0	60S ribosomal protein L22 OS=Mus musculus GN=Rpl22 PE=1 SV=2
Q64514	0.371376664	11	12	12	2	13	13	9	23	Tripeptidyl-peptidase 2 OS=Mus musculus GN=Tpp2 PE=1 SV=3
Q9Z1Z0	0.541286062	5	0	3	3	7	6	4	6	General vesicular transport factor p115 OS=Mus musculus GN=Uso1 PE=1 SV=2
O35639	0.088480247	43	30	41	8	57	43	34	57	Annexin A3 OS=Mus musculus GN=Anxa3 PE=1 SV=4
B7ZNU9	0.004471069	4	0	0	0	10	6	5	4	Beta-2-syntrophin OS=Mus musculus GN=Sntb2 PE=2 SV=1
Q9D4E6	0.12169071	22	13	16	3	27	26	12	19	Polyadenylate-binding protein OS=Mus musculus GN=Pabpc6 PE=1 SV=1
E9Q450	0.546811877	28	30	26	15	50	39	31	34	Tropomyosin alpha-1 chain OS=Mus musculus GN=Tpm1 PE=1 SV=1
E9Q4P0	0.224024195	112	0	97	46	132	0	0	0	KxDL motif-containing protein 1 (Fragment) OS=Mus musculus GN=Kxd1 PE=1 SV=1
E9PWZ3	0.396364891	13	12	8	5	16	17	13	13	Protein Rpl3l OS=Mus musculus GN=Rpl3l PE=1 SV=1
Q3UQ28	0.820735289	11	10	11	6	25	16	5	13	Peroxidasin homolog OS=Mus musculus GN=Pxdn PE=1 SV=2
B2RY53	0.417949923	13	7	5	4	12	10	10	13	EG620155 protein OS=Mus musculus GN=Gm6133 PE=2 SV=1

Q61655	0.23493532	7	4	7	2	10	8	8	5	ATP-dependent RNA helicase DDX19A OS=Mus musculus GN=Ddx19a PE=1 SV=2
A0A1B0GSF7	0.178247069	18	0	0	3	15	19	16	15	60S ribosomal protein L18 OS=Mus musculus GN=Rpl18 PE=1 SV=1
H3BIY9	0.127010146	8	5	7	0	10	13	6	12	AP complex subunit beta OS=Mus musculus GN=Ap2b1 PE=1 SV=1
P46638	0.455458301	8	7	8	5	10	9	9	15	Ras-related protein Rab-11B OS=Mus musculus GN=Rab11b PE=1 SV=3
P21279	0.548696184	7	4	4	0	8	7	0	8	Guanine nucleotide-binding protein G(q) subunit alpha OS=Mus musculus GN=Gnaq PE=1 SV=4
G3UXX3	0.160975935	10	9	10	3	16	9	9	15	Sepiapterin reductase OS=Mus musculus GN=Spr PE=1 SV=1
P99024	0.019141659	327	210	262	46	440	317	232	304	Tubulin beta-5 chain OS=Mus musculus GN=Tubb5 PE=1 SV=1
Q922F4	0.01999493	209	150	180	38	311	233	152	186	Tubulin beta-6 chain OS=Mus musculus GN=Tubb6 PE=1 SV=1
contaminant_KERATIN05	0.59701727	29	22	46	11	67	52	27	19	no description
P68372	0.007931338	311	205	249	46	416	310	228	281	Tubulin beta-4B chain OS=Mus musculus GN=Tubb4b PE=1 SV=1
Q9WUM4	0.152650374	12	6	7	2	14	10	5	12	Coronin-1C OS=Mus musculus GN=Coro1c PE=1 SV=2
Q7TMM9	0.008265494	304	194	239	44	402	298	216	263	Tubulin beta-2A chain OS=Mus musculus GN=Tubb2a PE=1 SV=1
Q91VH6	0.602245994	5	5	6	0	9	12	0	3	Protein MEMO1 OS=Mus musculus GN=Memo1 PE=1 SV=1
Q8VE47	0.2351008	3	0	0	0	5	0	0	4	Ubiquitin-like modifier-activating enzyme 5 OS=Mus musculus GN=Uba5 PE=1 SV=2
Q8R574	0.312430585	2	2	2	0	3	0	0	0	Phosphoribosyl pyrophosphate synthase-associated protein 2 OS=Mus musculus GN=Prpsap2 PE=1 SV=1
P84084	0.321663467	5	3	4	0	5	7	2	10	ADP-ribosylation factor 5 OS=Mus musculus GN=Arf5 PE=1 SV=2
H7BX01	0.391002219	0	2	0	0	0	3	0	0	Dynamin-like 120 kDa protein, mitochondrial OS=Mus musculus GN=Opa1 PE=1 SV=1
J3QNY1	0.552976076	14	0	16	9	22	22	7	27	Protein Gm9242 OS=Mus musculus GN=Gm9242 PE=4 SV=1
Q3UGB5	0.625755727	4	4	4	0	4	7	0	7	DAZ associated protein 1, isoform CRA_b OS=Mus musculus GN=Dazap1 PE=1 SV=1
Q9JII5	0.625755727	4	4	4	0	4	7	0	7	DAZ-associated protein 1 OS=Mus musculus GN=Dazap1 PE=1 SV=2
F8VQC1	0.703908018	0	0	0	2	0	3	0	0	Signal recognition particle subunit SRP72 OS=Mus musculus GN=Srp72 PE=1 SV=1
E9Q740	0.703908018	0	0	0	2	0	3	0	0	Protein Srp72 OS=Mus musculus GN=Srp72 PE=1 SV=1
Q9D1G1	0.731523359	0	0	0	2	3	2	0	4	Ras-related protein Rab-1B OS=Mus musculus GN=Rab1b PE=1 SV=1
A0A087WSQ1	0.756612216	2	0	0	0	0	3	0	0	SLIT-ROBO Rho GTPase-activating protein 2 (Fragment) OS=Mus musculus GN=Srgap2 PE=1 SV=1
G3UY75	0.762348101	0	2	0	0	0	0	3	0	Receptor-type tyrosine-protein phosphatase delta OS=Mus musculus GN=Ptprd PE=1 SV=1
P62313	0.794656282	4	0	0	0	0	0	0	6	U6 snRNA-associated Sm-like protein LSm6 OS=Mus musculus GN=Lsm6 PE=1 SV=1
Reverse_sp|P09838|TDT_MOUSE	0.82072506	0	0	2	0	0	3	0	0	DNA nucleotidylexotransferase OS=Mus musculus GN=Dntt PE=1 SV=3
Q3UMF0	0.913348156	0	2	0	0	0	0	0	3	Cordon-bleu protein-like 1 OS=Mus musculus GN=Cobll1 PE=1 SV=2
B1AR69	0.913348156	0	2	0	0	0	0	0	3	Protein Myh13 OS=Mus musculus GN=Myh13 PE=1 SV=1
B1AZ26	0.913348156	0	2	0	0	0	0	0	3	Cordon-bleu protein-like 1 (Fragment) OS=Mus musculus GN=Cobll1 PE=1 SV=8
B1AZ14	0.913348156	0	2	0	0	0	0	0	3	Cordon-bleu protein-like 1 OS=Mus musculus GN=Cobll1 PE=1 SV=1
B1AZ15	0.913348156	0	2	0	0	0	0	0	3	Cordon-bleu protein-like 1 OS=Mus musculus GN=Cobll1 PE=1 SV=1
Q99LS3	0.913348156	0	2	0	0	0	0	0	3	Phosphoserine phosphatase OS=Mus musculus GN=Psph PE=1 SV=1
E9QKZ2	0.91908832	0	0	2	0	3	0	0	0	Importin-9 OS=Mus musculus GN=Ipo9 PE=1 SV=1
Q91YE6	0.91908832	0	0	2	0	3	0	0	0	Importin-9 OS=Mus musculus GN=Ipo9 PE=1 SV=3
P26369	0.91908832	0	0	2	0	3	0	0	0	Splicing factor U2AF 65 kDa subunit OS=Mus musculus GN=U2af2 PE=1 SV=3
A0A0A6YXR4	0.91908832	0	0	2	0	3	0	0	0	Quinone oxidoreductase (Fragment) OS=Mus musculus GN=Cryz PE=1 SV=5
Q80XR5	0.91908832	0	0	2	0	3	0	0	0	Splicing factor U2AF 65 kDa subunit OS=Mus musculus GN=U2af2 PE=1 SV=1
Q8BXK9	0.954265367	0	0	0	3	0	5	4	0	Chloride intracellular channel protein 5 OS=Mus musculus GN=Clic5 PE=1 SV=1
Q62426	0.1481037	4	5	5	0	11	6	4	7	Cystatin-B OS=Mus musculus GN=Cstb PE=1 SV=1
P02301	0.244057952	46	34	52	13	80	56	46	35	Histone H3.3C OS=Mus musculus GN=H3f3c PE=3 SV=3
Q9CWF2	0.007218146	307	196	238	46	397	297	216	263	Tubulin beta-2B chain OS=Mus musculus GN=Tubb2b PE=1 SV=1
P45591	0.129065734	6	7	0	0	13	9	0	7	Cofilin-2 OS=Mus musculus GN=Cfl2 PE=1 SV=1
O70310	0.529793159	19	6	10	2	21	16	2	16	Glycylpeptide N-tetradecanoyltransferase 1 OS=Mus musculus GN=Nmt1 PE=1 SV=1
P68433	0.900389435	44	0	53	14	76	56	0	33	Histone H3.1 OS=Mus musculus GN=Hist1h3a PE=1 SV=2
contaminant_KERATIN22	0.809625526	66	66	91	45	135	101	92	69	no description
A2A6U3	0.418524953	15	13	16	4	29	10	13	19	Septin-9 OS=Mus musculus GN=Sept9 PE=1 SV=1
P99027	0.145734169	9	0	8	0	17	13	4	16	60S acidic ribosomal protein P2 OS=Mus musculus GN=Rplp2 PE=1 SV=3
P84091	0.584953501	6	2	7	2	10	6	5	4	AP-2 complex subunit mu OS=Mus musculus GN=Ap2m1 PE=1 SV=1
A0A0J9YUZ4	0.058985968	8	8	8	0	9	15	12	11	High mobility group protein B1 (Fragment) OS=Mus musculus GN=Hmgb1 PE=1 SV=1
P63158	0.058985968	8	8	8	0	9	15	12	11	High mobility group protein B1 OS=Mus musculus GN=Hmgb1 PE=1 SV=2
Q8K2T1	0.250590776	3	0	2	0	3	5	3	0	NmrA-like family domain-containing protein 1 OS=Mus musculus GN=Nmral1 PE=1 SV=1
D3YU12	0.250590776	3	0	2	0	3	5	3	0	NmrA-like family domain-containing protein 1 OS=Mus musculus GN=Nmral1 PE=1 SV=1
G5E8S7	0.250590776	3	0	2	0	3	5	3	0	NmrA-like family domain containing 1, isoform CRA_a OS=Mus musculus GN=Nmral1 PE=1 SV=1
H7BX22	0.183641318	17	13	11	2	22	14	10	17	Ran-specific GTPase-activating protein OS=Mus musculus GN=Ranbp1 PE=1 SV=1
Q922I7	0.054312274	8	8	5	0	13	10	9	9	MCG13402, isoform CRA_c OS=Mus musculus GN=Ptbp1 PE=1 SV=1
Q3UDE2	0.133464047	52	44	32	12	65	53	25	62	Tubulin--tyrosine ligase-like protein 12 OS=Mus musculus GN=Ttll12 PE=1 SV=1
D3Z494	0.229295285	16	11	15	0	12	21	17	32	MCG142264, isoform CRA_a OS=Mus musculus GN=Akr1b10 PE=1 SV=1
P62492	0.744191921	8	7	8	5	10	8	10	13	Ras-related protein Rab-11A OS=Mus musculus GN=Rab11a PE=1 SV=3
P08030	0.1626491	35	32	30	5	46	32	33	38	Adenine phosphoribosyltransferase OS=Mus musculus GN=Aprt PE=1 SV=2
P62141	0.383702843	45	30	27	5	49	38	17	52	Serine/threonine-protein phosphatase PP1-beta catalytic subunit OS=Mus musculus GN=Ppp1cb PE=1 SV=3
S4R2M7	0.523548104	148	119	151	70	170	186	173	180	Phosphoglycerate kinase OS=Mus musculus GN=Pgk1 PE=1 SV=1
P47226	0.716530963	0	4	4	3	6	5	0	5	Testin OS=Mus musculus GN=Tes PE=1 SV=1
Q80UG5	0.462040735	15	13	17	4	29	10	13	19	Septin-9 OS=Mus musculus GN=Sept9 PE=1 SV=1
P34884	0.836469134	12	5	12	0	18	0	0	10	Macrophage migration inhibitory factor OS=Mus musculus GN=Mif PE=1 SV=2
A2A6U5	0.368174052	14	13	15	3	25	10	13	17	Septin-9 (Fragment) OS=Mus musculus GN=Sept9 PE=1 SV=1
Q64337	0.139312504	3	3	0	0	4	6	3	0	Sequestosome-1 OS=Mus musculus GN=Sqstm1 PE=1 SV=1
D3YX79	0.843007197	0	0	4	2	7	0	2	4	Proteasome subunit alpha type OS=Mus musculus GN=Gm8394 PE=3 SV=1
Q9Z2U1	0.843007197	0	0	4	2	7	0	2	4	Proteasome subunit alpha type-5 OS=Mus musculus GN=Psma5 PE=1 SV=1
D3Z0S1	0.223753894	65	42	51	14	70	0	0	54	Annexin (Fragment) OS=Mus musculus GN=Anxa4 PE=1 SV=1
A0A140T8L3	0.332725684	34	20	27	5	32	0	0	30	Protein Rpl7a-ps5 OS=Mus musculus GN=Rpl7a-ps5 PE=4 SV=1
Q9CZD3	0.223216598	65	62	50	11	73	63	59	75	Glycine--tRNA ligase OS=Mus musculus GN=Gars PE=1 SV=1
Q8BK67	0.241864397	86	52	54	22	83	79	65	80	Protein RCC2 OS=Mus musculus GN=Rcc2 PE=1 SV=1
Q9D358	0.435033292	9	7	7	4	12	10	0	7	Low molecular weight phosphotyrosine protein phosphatase OS=Mus musculus GN=Acp1 PE=1 SV=3
F8VPU2	0.225466084	4	3	4	0	5	4	4	8	FERM, RhoGEF and pleckstrin domain-containing protein 1 OS=Mus musculus GN=Farp1 PE=1 SV=1
Q8R2Q8	0.019477191	6	0	8	0	12	11	10	7	Bone marrow stromal antigen 2 OS=Mus musculus GN=Bst2 PE=1 SV=1
Q8BGJ5	0.054782418	8	8	5	0	13	10	8	9	MCG13402, isoform CRA_a OS=Mus musculus GN=Ptbp1 PE=1 SV=1
O70435	0.099330756	3	4	0	0	9	4	4	3	Proteasome subunit alpha type-3 OS=Mus musculus GN=Psma3 PE=1 SV=3
E9Q3P9	0.116378032	8	7	8	5	10	0	0	0	Ras-related protein Rab-11A OS=Mus musculus GN=Rab11a PE=4 SV=1
G3UY29	0.116378032	8	7	8	5	10	0	0	0	MCG22989, isoform CRA_a OS=Mus musculus GN=Rab11b PE=4 SV=1
F8WGS1	0.116378032	8	7	8	5	10	0	0	0	Ras-related protein Rab-11A (Fragment) OS=Mus musculus GN=Rab11a PE=4 SV=1
P62751	0.898184857	12	11	4	8	13	15	9	13	60S ribosomal protein L23a OS=Mus musculus GN=Rpl23a PE=1 SV=1
contaminant_KERATIN06	0.029775118	12	6	13	0	19	19	13	8	no description
Q8BHN3	0.487742658	6	2	0	2	7	5	3	4	Neutral alpha-glucosidase AB OS=Mus musculus GN=Ganab PE=1 SV=1
P84244	0.073994888	45	35	0	0	80	56	0	35	Histone H3.3 OS=Mus musculus GN=H3f3a PE=1 SV=2
Q9JK81	0.080521409	16	16	9	4	20	18	8	18	UPF0160 protein MYG1, mitochondrial OS=Mus musculus GN=Myg1 PE=1 SV=1
P34022	0.360771148	20	17	11	2	24	16	10	21	Ran-specific GTPase-activating protein OS=Mus musculus GN=Ranbp1 PE=1 SV=2
Q9EPU0	0.0869985	29	25	31	8	37	36	23	36	Regulator of nonsense transcripts 1 OS=Mus musculus GN=Upf1 PE=1 SV=2
contaminant_KERATIN03	0.66998613	69	64	89	34	109	107	75	72	no description
Q9QY36	0.528928949	6	4	2	0	0	6	6	5	N-alpha-acetyltransferase 10 OS=Mus musculus GN=Naa10 PE=1 SV=1
B1AUY7	0.528928949	6	4	2	0	0	6	6	5	N-alpha-acetyltransferase 10 OS=Mus musculus GN=Naa10 PE=1 SV=1
B1AUY8	0.528928949	6	4	2	0	0	6	6	5	N-alpha-acetyltransferase 10 OS=Mus musculus GN=Naa10 PE=1 SV=1
P19246	0.886451011	0	0	0	6	10	7	0	0	Neurofilament heavy polypeptide OS=Mus musculus GN=Nefh PE=1 SV=3
Q9QUI0	0.468008444	91	72	61	21	62	73	88	124	Transforming protein RhoA OS=Mus musculus GN=Rhoa PE=1 SV=1
contaminant_KERATIN16	0.485058889	10	7	9	3	12	9	13	7	no description
G5E829	0.131694756	17	8	18	0	29	19	12	21	Plasma membrane calcium-transporting ATPase 1 OS=Mus musculus GN=Atp2b1 PE=1 SV=1
Q3TWV4	0.657673882	6	2	7	2	10	5	5	4	AP-2 complex subunit mu OS=Mus musculus GN=Ap2m1 PE=1 SV=1
Q9WVK4	0.291558912	52	30	39	8	61	40	26	55	EH domain-containing protein 1 OS=Mus musculus GN=Ehd1 PE=1 SV=1
P62983	0.545974288	117	99	101	46	141	126	126	118	Ubiquitin-40S ribosomal protein S27a OS=Mus musculus GN=Rps27a PE=1 SV=2
Q8BZY3	0.016354237	6	4	6	0	9	8	9	4	Protein Ddx19b OS=Mus musculus GN=Ddx19b PE=1 SV=1
contaminant_UBIQUITIN08	0.606601808	112	95	96	46	131	122	122	114	no description
A2AE89	0.013079041	5	0	0	0	9	8	4	7	Glutathione S-transferase Mu 1 OS=Mus musculus GN=Gstm1 PE=1 SV=2
P10649	0.013079041	5	0	0	0	9	8	4	7	Glutathione S-transferase Mu 1 OS=Mus musculus GN=Gstm1 PE=1 SV=2
A2A6F8	0.74496571	3	0	2	0	4	0	0	3	60S ribosomal protein L23 (Fragment) OS=Mus musculus GN=Rpl23 PE=1 SV=1
A0A140T8M7	0.856411676	12	11	4	8	13	14	9	13	Protein Rpl23a-ps3 OS=Mus musculus GN=Rpl23a-ps3 PE=3 SV=1
Q6URW6	0.953699919	11	9	6	4	21	8	5	8	Myosin-14 OS=Mus musculus GN=Myh14 PE=1 SV=1
A2A513	0.636387905	21	23	27	10	31	36	26	20	Keratin, type I cytoskeletal 10 OS=Mus musculus GN=Krt10 PE=1 SV=1
Q9CYL5	0.877048817	10	9	8	6	15	7	8	16	Golgi-associated plant pathogenesis-related protein 1 OS=Mus musculus GN=Glipr2 PE=1 SV=3
P16627	0.687753333	81	71	79	37	93	101	96	82	Heat shock 70 kDa protein 1-like OS=Mus musculus GN=Hspa1l PE=1 SV=4
Q9R0K7	0.28872551	4	4	5	0	10	4	3	7	Plasma membrane calcium-transporting ATPase 2 OS=Mus musculus GN=Atp2b2 PE=1 SV=2
Q9WTI7	0.452481681	46	35	44	8	65	39	25	55	Unconventional myosin-Ic OS=Mus musculus GN=Myo1c PE=1 SV=2
P62835	0.072081155	13	7	12	0	16	12	16	15	Ras-related protein Rap-1A OS=Mus musculus GN=Rap1a PE=1 SV=1
F8WGG3	0.086029878	14	12	7	4	17	14	6	14	UPF0160 protein MYG1, mitochondrial (Fragment) OS=Mus musculus GN=Myg1 PE=1 SV=1
P47911	0.210608528	39	21	25	5	36	28	30	30	60S ribosomal protein L6 OS=Mus musculus GN=Rpl6 PE=1 SV=3
O08638	0.993276623	63	52	58	37	91	79	61	58	Myosin-11 OS=Mus musculus GN=Myh11 PE=1 SV=1
Q8BGQ7	0.338125278	96	78	77	18	80	90	83	117	Alanine--tRNA ligase, cytoplasmic OS=Mus musculus GN=Aars PE=1 SV=1
Q9Z2U0	0.050970093	7	0	5	0	10	7	6	10	Proteasome subunit alpha type-7 OS=Mus musculus GN=Psma7 PE=1 SV=1
P63321	0.73804578	8	8	8	0	9	10	0	14	Ras-related protein Ral-A OS=Mus musculus GN=Rala PE=1 SV=1
B2RXM2	0.831968559	14	17	16	9	21	22	7	27	EG627828 protein OS=Mus musculus GN=Gm6793 PE=1 SV=1
F8WID5	0.059205304	27	28	0	0	49	37	32	33	Tropomyosin alpha-1 chain OS=Mus musculus GN=Tpm1 PE=1 SV=1
Q9QYJ0	0.17295565	16	9	24	2	14	18	21	17	DnaJ homolog subfamily A member 2 OS=Mus musculus GN=Dnaja2 PE=1 SV=1
P04104	0.398489498	22	19	27	8	32	23	27	22	Keratin, type II cytoskeletal 1 OS=Mus musculus GN=Krt1 PE=1 SV=4
Q8BG05	0.813609932	14	18	16	9	22	22	7	27	Heterogeneous nuclear ribonucleoprotein A3 OS=Mus musculus GN=Hnrnpa3 PE=1 SV=1
A2AL12	0.813609932	14	18	16	9	22	22	7	27	Heterogeneous nuclear ribonucleoprotein A3 OS=Mus musculus GN=Hnrnpa3 PE=1 SV=1
Q9DC51	0.345435321	23	17	25	6	26	26	16	29	Guanine nucleotide-binding protein G(k) subunit alpha OS=Mus musculus GN=Gnai3 PE=1 SV=3
P61971	0.836019833	11	3	8	0	9	0	0	11	Nuclear transport factor 2 OS=Mus musculus GN=Nutf2 PE=1 SV=1
Q6P1F6	0.239968476	29	21	31	10	28	32	26	38	Serine/threonine-protein phosphatase 2A 55 kDa regulatory subunit B alpha isoform OS=Mus musculus GN=Ppp2r2a PE=1 SV=1
Q60865	0.180631487	4	4	3	0	3	6	5	6	Caprin-1 OS=Mus musculus GN=Caprin1 PE=1 SV=2
Q8CB58	0.082528587	8	8	5	0	11	10	8	9	MCG13402, isoform CRA_d OS=Mus musculus GN=Ptbp1 PE=1 SV=1
A0A0G2JGP4	0.140626898	10	4	0	0	10	7	10	11	GTPase NRas (Fragment) OS=Mus musculus GN=Nras PE=1 SV=1
A0A0G2JE25	0.140626898	10	4	0	0	10	7	10	11	GTPase NRas (Fragment) OS=Mus musculus GN=Nras PE=1 SV=1
P97855	0.874438245	14	8	14	6	17	16	10	14	Ras GTPase-activating protein-binding protein 1 OS=Mus musculus GN=G3bp1 PE=1 SV=1
Q78PY7	0.196797092	41	41	43	10	55	48	31	49	Staphylococcal nuclease domain-containing protein 1 OS=Mus musculus GN=Snd1 PE=1 SV=1
A0A0G2JE52	0.10175105	12	7	12	0	13	12	16	15	Ras-related protein Rap-1A OS=Mus musculus GN=Rap1a PE=1 SV=1
P61222	0.429939624	37	30	26	9	37	30	24	47	ATP-binding cassette sub-family E member 1 OS=Mus musculus GN=Abce1 PE=1 SV=1
Q921W7	0.799082841	0	7	7	3	13	5	0	5	Putative uncharacterized protein OS=Mus musculus GN=Tes PE=1 SV=2
Q3ULB1	0.799082841	0	7	7	3	13	5	0	5	Putative uncharacterized protein OS=Mus musculus GN=Tes PE=1 SV=1
P50516	0.672029362	12	7	14	4	13	15	8	14	V-type proton ATPase catalytic subunit A OS=Mus musculus GN=Atp6v1a PE=1 SV=2
A0A0J9YUS5	0.609767249	12	14	10	4	13	12	15	14	Eukaryotic translation initiation factor 4 gamma 1 OS=Mus musculus GN=Eif4g1 PE=1 SV=1
Q6NZJ6	0.609767249	12	14	10	4	13	12	15	14	Eukaryotic translation initiation factor 4 gamma 1 OS=Mus musculus GN=Eif4g1 PE=1 SV=1
E9Q9E1	0.609767249	12	14	10	4	13	12	15	14	Eukaryotic translation initiation factor 4 gamma 1 OS=Mus musculus GN=Eif4g1 PE=1 SV=1
E9PVC6	0.609767249	12	14	10	4	13	12	15	14	Eukaryotic translation initiation factor 4 gamma 1 OS=Mus musculus GN=Eif4g1 PE=1 SV=1
E9PVC5	0.609767249	12	14	10	4	13	12	15	14	Eukaryotic translation initiation factor 4 gamma 1 OS=Mus musculus GN=Eif4g1 PE=1 SV=1
Q91V92	0.939769476	72	41	61	26	105	57	33	75	ATP-citrate synthase OS=Mus musculus GN=Acly PE=1 SV=1
P12970	0.299569097	39	21	31	5	36	28	27	38	60S ribosomal protein L7a OS=Mus musculus GN=Rpl7a PE=1 SV=2
Q9QXY6	0.550573006	15	8	14	4	17	13	8	17	EH domain-containing protein 3 OS=Mus musculus GN=Ehd3 PE=1 SV=2
Q9ERD7	0.376529057	144	81	97	18	131	115	74	136	Tubulin beta-3 chain OS=Mus musculus GN=Tubb3 PE=1 SV=1
P63005	0.369159251	21	15	15	2	15	20	16	20	Platelet-activating factor acetylhydrolase IB subunit alpha OS=Mus musculus GN=Pafah1b1 PE=1 SV=2
H3BLG5	0.271839866	15	13	13	0	22	13	12	26	Syntenin-1 (Fragment) OS=Mus musculus GN=Sdcbp PE=1 SV=1
P46467	0.164623313	0	0	2	0	0	4	2	2	Vacuolar protein sorting-associated protein 4B OS=Mus musculus GN=Vps4b PE=1 SV=2
Q2YDW2	0.551199045	2	0	5	0	5	5	0	4	Protein misato homolog 1 OS=Mus musculus GN=Msto1 PE=1 SV=1
E9PUB7	0.551199045	2	0	5	0	5	5	0	4	Protein misato homolog 1 OS=Mus musculus GN=Msto1 PE=1 SV=1
O88844	0.534995555	4	0	6	0	5	6	0	9	Isocitrate dehydrogenase [NADP] cytoplasmic OS=Mus musculus GN=Idh1 PE=1 SV=2
Q9D1D4	0.089128157	3	0	0	0	5	3	0	4	Transmembrane emp24 domain-containing protein 10 OS=Mus musculus GN=Tmed10 PE=1 SV=1
D3YZJ1	0.207290571	3	3	0	0	3	6	3	0	Sequestosome-1 OS=Mus musculus GN=Sqstm1 PE=1 SV=1
O70251	0.209820241	3	0	0	0	5	0	0	3	Elongation factor 1-beta OS=Mus musculus GN=Eef1b PE=1 SV=5
O35379	0.260462485	3	0	0	0	2	4	0	6	Multidrug resistance-associated protein 1 OS=Mus musculus GN=Abcc1 PE=1 SV=1
A0A1D5RM76	0.298267572	8	5	7	4	9	7	0	8	Tubulin beta-3 chain OS=Mus musculus GN=Tubb3 PE=1 SV=1
Q7TT37	0.305498977	8	0	0	4	3	0	0	13	Elongator complex protein 1 OS=Mus musculus GN=Ikbkap PE=1 SV=2
Q8VEH5	0.331780661	0	0	3	0	4	3	0	5	EPM2A-interacting protein 1 OS=Mus musculus GN=Epm2aip1 PE=1 SV=1
A0A1D5RMJ8	0.391002219	0	0	0	3	0	0	0	4	CCR4-NOT transcription complex subunit 1 OS=Mus musculus GN=Cnot1 PE=1 SV=1
Q8CG76	0.497443093	4	0	5	0	6	7	0	5	Aflatoxin B1 aldehyde reductase member 2 OS=Mus musculus GN=Akr7a2 PE=1 SV=3
Q8CGA0	0.55333092	3	3	0	0	0	4	0	0	Protein phosphatase 1F OS=Mus musculus GN=Ppm1f PE=1 SV=1
Q9D1R9	0.704565544	3	0	0	0	0	0	4	0	60S ribosomal protein L34 OS=Mus musculus GN=Rpl34 PE=1 SV=2
Q8BJU0	0.769176926	4	2	0	0	0	0	0	4	Small glutamine-rich tetratricopeptide repeat-containing protein alpha OS=Mus musculus GN=Sgta PE=1 SV=2

Q60737	0.803869701	4	0	2	0	3	0	0	5	Casein kinase II subunit alpha OS=Mus musculus GN=Csnk2a1 PE=1 SV=2
O08808	0.807167315	0	3	0	0	0	0	4	0	Protein diaphanous homolog 1 OS=Mus musculus GN=Diaph1 PE=1 SV=1
Q4U4S6	0.807167315	0	3	0	0	0	0	4	0	Xin actin-binding repeat-containing protein 2 OS=Mus musculus GN=Xirp2 PE=1 SV=1
P61967	0.826683737	2	4	0	0	0	3	0	5	AP-1 complex subunit sigma-1A OS=Mus musculus GN=Ap1s1 PE=1 SV=1
Q3UX61	0.393331313	5	3	0	0	0	6	6	4	N-alpha-acetyltransferase 11 OS=Mus musculus GN=Naa11 PE=1 SV=1
P62754	0.323533389	20	18	23	3	31	19	16	19	40S ribosomal protein S6 OS=Mus musculus GN=Rps6 PE=1 SV=1
Q9D2Y4	0.424046399	5	4	4	0	4	5	3	11	Mixed lineage kinase domain-like protein OS=Mus musculus GN=Mlkl PE=1 SV=1
E9QPE7	0.457554452	64	52	58	37	90	0	62	58	Myosin-11 OS=Mus musculus GN=Myh11 PE=1 SV=1
P62242	0.362907971	48	34	32	12	47	37	34	49	40S ribosomal protein S8 OS=Mus musculus GN=Rps8 PE=1 SV=2
B2M1R6	0.33613807	34	18	28	3	28	30	23	29	Heterogeneous nuclear ribonucleoprotein K OS=Mus musculus GN=Hnrnpk PE=1 SV=1
Q8VED5	0.19842754	15	13	19	9	0	22	0	15	Keratin, type II cytoskeletal 79 OS=Mus musculus GN=Krt79 PE=1 SV=2
P35762	0.802851382	8	10	7	0	12	8	0	13	CD81 antigen OS=Mus musculus GN=Cd81 PE=1 SV=2
P17879	0.856625887	87	76	82	39	95	99	97	83	Heat shock 70 kDa protein 1B OS=Mus musculus GN=Hspa1b PE=1 SV=3
contaminant_KERATIN07	0.103078147	14	8	19	0	27	22	13	10	no description
P61979	0.350611877	36	19	28	3	29	30	24	30	Heterogeneous nuclear ribonucleoprotein K OS=Mus musculus GN=Hnrnpk PE=1 SV=1
Q9Z1Z2	0.29290152	37	25	34	3	44	35	22	29	Serine-threonine kinase receptor-associated protein OS=Mus musculus GN=Strap PE=1 SV=2
Q6ZWV7	0.026249612	0	0	4	0	6	6	4	5	60S ribosomal protein L35 OS=Mus musculus GN=Rpl35 PE=1 SV=1
Q8BH43	0.120141757	3	5	0	0	7	5	5	4	Wiskott-Aldrich syndrome protein family member 2 OS=Mus musculus GN=Wasf2 PE=1 SV=1
D3YWH9	0.426466153	27	15	17	5	21	22	20	21	40S ribosomal protein S9 OS=Mus musculus GN=Rps9 PE=1 SV=1
Q3UV17	0.98182997	15	15	23	8	40	17	17	6	Keratin, type II cytoskeletal 2 oral OS=Mus musculus GN=Krt76 PE=1 SV=1
Q60715	0.143304561	3	3	0	2	0	3	0	4	Prolyl 4-hydroxylase subunit alpha-1 OS=Mus musculus GN=P4ha1 PE=1 SV=2
E9Q7B0	0.143304561	3	3	0	2	0	3	0	4	Prolyl 4-hydroxylase subunit alpha-1 OS=Mus musculus GN=P4ha1 PE=1 SV=1
O09172	0.269283738	3	3	0	2	0	4	0	3	Glutamate--cysteine ligase regulatory subunit OS=Mus musculus GN=Gclm PE=1 SV=1
P17156	0.933288014	117	95	101	60	129	130	110	117	Heat shock-related 70 kDa protein 2 OS=Mus musculus GN=Hspa2 PE=1 SV=2
Q8CBR6	0.528574465	2	3	5	0	0	2	6	5	Tsukushin OS=Mus musculus GN=Tsku PE=2 SV=2
Q9JM14	0.76543117	3	3	4	0	4	6	0	3	5'(3')-deoxyribonucleotidase, cytosolic type OS=Mus musculus GN=Nt5c PE=1 SV=1
A2AW41	0.059821767	5	0	5	0	10	5	4	7	Protein Hnrnpr (Fragment) OS=Mus musculus GN=Hnrnpr PE=1 SV=1
Q9QXB9	0.104210758	7	7	4	2	0	7	0	6	Developmentally-regulated GTP-binding protein 2 OS=Mus musculus GN=Drg2 PE=1 SV=1
contaminant_KERATIN12	0.960476599	22	11	28	9	35	27	18	11	no description
D3YYZ2	0.943886605	102	92	84	46	110	102	110	99	MCG1031578 OS=Mus musculus GN=Gm5239 PE=1 SV=1
E9Q0U7	0.336955817	9	11	6	0	17	10	4	14	Heat shock protein 105 kDa OS=Mus musculus GN=Hsph1 PE=1 SV=1
Q61699	0.336955817	9	11	6	0	17	10	4	14	Heat shock protein 105 kDa OS=Mus musculus GN=Hsph1 PE=1 SV=2
P11438	0.919517221	12	9	9	7	11	12	14	11	Lysosome-associated membrane glycoprotein 1 OS=Mus musculus GN=Lamp1 PE=1 SV=2
Q62465	0.770349045	47	43	40	22	64	53	27	53	Synaptic vesicle membrane protein VAT-1 homolog OS=Mus musculus GN=Vat1 PE=1 SV=3
Q925E7	0.138350725	9	4	9	0	12	7	8	11	Serine/threonine-protein phosphatase 2A 55 kDa regulatory subunit B delta isoform OS=Mus musculus GN=Ppp2r2d PE=1 SV=1
P08556	0.146673227	10	4	8	0	10	7	10	11	GTPase NRas OS=Mus musculus GN=Nras PE=1 SV=1
H3BK96	0.368493679	36	19	28	3	29	29	24	29	Heterogeneous nuclear ribonucleoprotein K (Fragment) OS=Mus musculus GN=Hnrnpk PE=1 SV=8
Q6Q477	0.044866919	3	0	4	0	5	4	4	5	Plasma membrane calcium-transporting ATPase 4 OS=Mus musculus GN=Atp2b4 PE=1 SV=1
Q9DCL9	0.574475978	29	28	21	6	36	25	17	30	Multifunctional protein ADE2 OS=Mus musculus GN=Paics PE=1 SV=4
E0CXB1	0.766558599	4	4	8	5	8	7	7	5	Proteasome endopeptidase complex OS=Mus musculus GN=Psma6 PE=1 SV=1
P70670	0.831239205	3	0	4	0	0	4	0	5	Nascent polypeptide-associated complex subunit alpha, muscle-specific form OS=Mus musculus GN=Naca PE=1 SV=2
contaminant_KERATIN21	0.894529057	36	18	33	18	55	35	30	15	no description
G5E8N7	0.964952892	5	3	4	2	6	4	5	3	Bifunctional polynucleotide phosphatase/kinase OS=Mus musculus GN=Pnkp PE=1 SV=1
E9Q9A5	0.964952892	5	3	4	2	6	4	5	3	Bifunctional polynucleotide phosphatase/kinase OS=Mus musculus GN=Pnkp PE=1 SV=1
Q9JLV6	0.964952892	5	3	4	2	6	4	5	3	Bifunctional polynucleotide phosphatase/kinase OS=Mus musculus GN=Pnkp PE=1 SV=2
P50247	0.152503527	66	48	59	13	66	60	53	60	Adenosylhomocysteinase OS=Mus musculus GN=Ahcy PE=1 SV=3
Q9Z1R9	0.214407003	71	42	39	24	56	59	0	54	MCG124046 OS=Mus musculus GN=Prss1 PE=1 SV=1
H3BKM0	0.391389055	7	3	7	0	10	8	2	9	AP-2 complex subunit beta OS=Mus musculus GN=Ap2b1 PE=1 SV=1
Q9QWL7	0.984712237	21	10	26	8	33	24	16	10	Keratin, type I cytoskeletal 17 OS=Mus musculus GN=Krt17 PE=1 SV=3
P60867	0.540669399	27	15	18	2	20	22	13	24	40S ribosomal protein S20 OS=Mus musculus GN=Rps20 PE=1 SV=1
Q3TL72	0.245593927	11	3	9	0	11	13	6	9	NEDD8-activating enzyme E1 catalytic subunit OS=Mus musculus GN=Uba3 PE=1 SV=1
Q8C878	0.245593927	11	3	9	0	11	13	6	9	NEDD8-activating enzyme E1 catalytic subunit OS=Mus musculus GN=Uba3 PE=1 SV=2
P84096	0.960219258	13	6	13	5	15	10	12	10	Rho-related GTP-binding protein RhoG OS=Mus musculus GN=Rhog PE=1 SV=1
Q9R0N0	0.830090344	19	22	13	9	23	19	16	22	Galactokinase OS=Mus musculus GN=Galk1 PE=1 SV=2
B7ZBY7	0.397788873	4	6	0	0	7	5	0	7	Ubiquitin-conjugating enzyme E2 variant 1 OS=Mus musculus GN=Ube2v1 PE=3 SV=1
E9PY39	0.397788873	4	6	0	0	7	5	0	7	Protein Gm20431 OS=Mus musculus GN=Gm20431 PE=3 SV=1
Q9CZY3	0.397788873	4	6	0	0	7	5	0	7	Ubiquitin-conjugating enzyme E2 variant 1 OS=Mus musculus GN=Ube2v1 PE=1 SV=1
P50446	0.932743768	18	0	16	8	23	15	15	0	Keratin, type II cytoskeletal 6A OS=Mus musculus GN=Krt6a PE=1 SV=3
H3BK18	0.629574176	32	0	28	3	26	0	0	27	Heterogeneous nuclear ribonucleoprotein K (Fragment) OS=Mus musculus GN=Hnrnpk PE=1 SV=1
Q6ZWN5	0.526135056	37	17	22	5	25	26	24	27	40S ribosomal protein S9 OS=Mus musculus GN=Rps9 PE=1 SV=3
A0A1B0GQU8	0.411139668	22	18	19	3	15	26	19	18	60S ribosomal protein L18 OS=Mus musculus GN=Rpl18 PE=1 SV=1
P35980	0.411139668	22	18	19	3	15	26	19	18	60S ribosomal protein L18 OS=Mus musculus GN=Rpl18 PE=1 SV=3
P61255	0.945641694	16	18	22	7	28	16	16	19	60S ribosomal protein L26 OS=Mus musculus GN=Rpl26 PE=1 SV=1
E9PWY9	0.467922964	26	18	27	8	24	23	23	29	Phenylalanine--tRNA ligase alpha subunit OS=Mus musculus GN=Farsa PE=1 SV=1
E9PXM6	0.005128225	0	6	8	0	8	10	10	7	Equilibrative nucleoside transporter 1 OS=Mus musculus GN=Slc29a1 PE=1 SV=1
Reverse_tr|E9Q8K5|E9Q8K5_MOUSE	0.565137286	0	0	2	0	0	2	0	3	Titin OS=Mus musculus GN=Ttn PE=1 SV=1
Q99JB8	0.596269088	4	0	4	0	3	3	0	9	Protein kinase C and casein kinase II substrate protein 3 OS=Mus musculus GN=Pacsin3 PE=1 SV=1
P70227	0.676874056	4	0	4	0	0	0	4	6	Inositol 1,4,5-trisphosphate receptor type 3 OS=Mus musculus GN=Itpr3 PE=1 SV=3
Q9CZH3	0.697327445	2	2	2	0	3	0	0	2	Proteasome assembly chaperone 3 OS=Mus musculus GN=Psmg3 PE=1 SV=1
Q9CYI4	0.808031223	2	2	0	0	0	0	2	3	Putative RNA-binding protein Luc7-like 1 OS=Mus musculus GN=Luc7l PE=1 SV=2
O35682	0.848831264	4	2	6	0	0	4	0	6	Myeloid-associated differentiation marker OS=Mus musculus GN=Myadm PE=1 SV=2
Q9Z329	0.897141878	0	0	4	0	0	5	0	0	Inositol 1,4,5-trisphosphate receptor type 2 OS=Mus musculus GN=Itpr2 PE=1 SV=4
Q6ZWU9	0.918030989	24	17	16	8	18	19	22	22	40S ribosomal protein S27 OS=Mus musculus GN=Rps27 PE=1 SV=3
A0A0G2JDW7	0.918030989	24	17	16	8	18	19	22	22	40S ribosomal protein S27 (Fragment) OS=Mus musculus GN=Rps27 PE=1 SV=1
Q6P9J9	0.781839724	13	7	8	5	9	7	9	16	Anoctamin-6 OS=Mus musculus GN=Ano6 PE=1 SV=1
Q6ZWY9	0.402276641	262	0	0	95	218	224	216	227	Histone H2B type 1-C/E/G OS=Mus musculus GN=Hist1h2bc PE=1 SV=3
P61750	0.282910935	9	7	7	0	9	10	6	13	ADP-ribosylation factor 4 OS=Mus musculus GN=Arf4 PE=1 SV=2
Q9DCD0	0.878234836	21	15	20	7	28	15	15	20	6-phosphogluconate dehydrogenase, decarboxylating OS=Mus musculus GN=Pgd PE=1 SV=3
B1ARA3	0.892787866	16	18	22	7	27	16	16	19	60S ribosomal protein L26 (Fragment) OS=Mus musculus GN=Rpl26 PE=1 SV=1
Q8C0C7	0.529978451	26	18	28	8	24	23	23	29	Phenylalanine--tRNA ligase alpha subunit OS=Mus musculus GN=Farsa PE=1 SV=1
O88456	0.303070343	3	0	4	0	0	3	5	5	Calpain small subunit 1 OS=Mus musculus GN=Capns1 PE=1 SV=1
A0A0R4IZW8	0.303070343	3	0	4	0	0	3	5	5	Calpain small subunit 1 OS=Mus musculus GN=Capns1 PE=1 SV=1
A0A0R4J1C2	0.303070343	3	0	4	0	0	3	5	5	Calpain small subunit 1 OS=Mus musculus GN=Capns1 PE=1 SV=1
P62331	0.577847768	0	3	4	0	5	5	0	3	ADP-ribosylation factor 6 OS=Mus musculus GN=Arf6 PE=1 SV=2
P39053	0.25945162	10	6	4	0	10	6	7	10	Dynamin-1 OS=Mus musculus GN=Dnm1 PE=1 SV=2
A0A087WRC0	0.416975625	8	11	12	0	11	11	8	21	Tripeptidyl-peptidase 2 OS=Mus musculus GN=Tpp2 PE=1 SV=1
P14152	0.527015299	11	14	13	2	16	10	0	11	Malate dehydrogenase, cytoplasmic OS=Mus musculus GN=Mdh1 PE=1 SV=3
A0A140T8L1	0.245252103	35	0	27	5	32	25	23	30	Protein Rpl7a-ps3 OS=Mus musculus GN=Rpl7a-ps3 PE=4 SV=1
Q9EQK5	0.735196524	13	9	15	2	8	15	8	17	Major vault protein OS=Mus musculus GN=Mvp PE=1 SV=4
Q64525	0.410290189	264	0	0	96	218	224	217	227	Histone H2B type 2-B OS=Mus musculus GN=Hist2h2bb PE=1 SV=3
Q61411	0.19461563	9	4	9	0	8	7	10	11	GTPase HRas OS=Mus musculus GN=Hras PE=1 SV=2
Q8BP67	0.869189662	7	10	5	0	14	9	0	4	60S ribosomal protein L24 OS=Mus musculus GN=Rpl24 PE=1 SV=2
Q64478	0.411685461	265	0	0	96	218	224	217	227	Histone H2B type 1-H OS=Mus musculus GN=Hist1h2bh PE=1 SV=3
Q00612	0.333008977	41	36	47	9	36	45	42	40	Glucose-6-phosphate 1-dehydrogenase X OS=Mus musculus GN=G6pdx PE=1 SV=3
A0A0A6YXL3	0.415098238	4	0	2	0	0	2	6	3	60S ribosomal protein L31 OS=Mus musculus GN=Rpl31 PE=1 SV=1
A6PWR1	0.586133163	3	0	3	0	3	2	0	6	Protein kinase C and casein kinase II substrate protein 3 (Fragment) OS=Mus musculus GN=Pacsin3 PE=1 SV=1
Q3UE92	0.775597727	4	3	2	0	3	0	3	5	Putative uncharacterized protein OS=Mus musculus GN=Xpnpep1 PE=1 SV=1
Q6P1B1	0.775597727	4	3	2	0	3	0	3	5	Xaa-Pro aminopeptidase 1 OS=Mus musculus GN=Xpnpep1 PE=1 SV=1
S4R1I3	0.775597727	4	3	2	0	3	0	3	5	Xaa-Pro aminopeptidase 1 OS=Mus musculus GN=Xpnpep1 PE=1 SV=1
Q8BWY3	0.495846162	9	0	3	0	0	0	8	10	Eukaryotic peptide chain release factor subunit 1 OS=Mus musculus GN=Etf1 PE=1 SV=4
Q00493	0.804384873	19	12	22	7	29	20	8	16	Carboxypeptidase E OS=Mus musculus GN=Cpe PE=1 SV=2
A2A8L5	0.767398729	34	28	28	8	32	29	22	36	Receptor-type tyrosine-protein phosphatase F OS=Mus musculus GN=Ptprf PE=1 SV=1
D3Z5G8	0.287412639	7	6	6	3	5	0	0	10	Disco-interacting protein 2 homolog B OS=Mus musculus GN=Dip2b PE=1 SV=1
contaminant_KERATIN18	0.933444688	35	12	32	11	44	30	23	12	no description
P28660	0.338225522	15	9	9	0	17	12	6	18	Nck-associated protein 1 OS=Mus musculus GN=Nckap1 PE=1 SV=2
A2AS98	0.338225522	15	9	9	0	17	12	6	18	Nck-associated protein 1 OS=Mus musculus GN=Nckap1 PE=1 SV=1
P19096	0.951521481	311	209	270	79	325	252	185	282	Fatty acid synthase OS=Mus musculus GN=Fasn PE=1 SV=2
Q9QZB7	0.164964423	4	3	3	0	4	0	0	0	Actin-related protein 10 OS=Mus musculus GN=Actr10 PE=1 SV=2
Q9JL18	0.441094296	4	3	3	0	0	0	0	4	Squamous cell carcinoma antigen recognized by T-cells 3 OS=Mus musculus GN=Sart3 PE=1 SV=1
Q8K183	0.441094296	4	3	3	0	0	0	0	4	Pyridoxal kinase OS=Mus musculus GN=Pdxk PE=1 SV=1
P62814	0.244037566	9	5	11	0	10	8	9	13	V-type proton ATPase subunit B, brain isoform OS=Mus musculus GN=Atp6v1b2 PE=1 SV=1
P27659	0.235291345	73	45	62	15	75	52	50	57	60S ribosomal protein L3 OS=Mus musculus GN=Rpl3 PE=1 SV=3
Q9JK38	0.537239355	2	0	3	0	3	0	0	0	Glucosamine 6-phosphate N-acetyltransferase OS=Mus musculus GN=Gnpnat1 PE=1 SV=1
F2Z456	0.598979449	0	2	3	0	3	0	0	0	NADH-cytochrome b5 reductase OS=Mus musculus GN=Cyb5r3 PE=1 SV=1
P62204	0.758520635	44	36	34	6	46	29	26	43	Calmodulin OS=Mus musculus GN=Calm1 PE=1 SV=2
Q3UKW2	0.758520635	44	36	34	6	46	29	26	43	Calmodulin OS=Mus musculus GN=Calm1 PE=1 SV=1
D6RCW7	0.855779197	8	4	3	0	6	4	0	8	F-actin-capping protein subunit alpha-2 OS=Mus musculus GN=Capza2 PE=1 SV=1
Q3UEB3	0.871573761	2	3	0	0	0	0	3	0	Poly(U)-binding-splicing factor PUF60 OS=Mus musculus GN=Puf60 PE=1 SV=2
D3YUP1	0.994493935	0	2	3	0	4	2	0	0	Histone-arginine methyltransferase CARM1 OS=Mus musculus GN=Carm1 PE=1 SV=1
Q9WVG6	0.994493935	0	2	3	0	4	2	0	0	Histone-arginine methyltransferase CARM1 OS=Mus musculus GN=Carm1 PE=1 SV=2
Q504P4	0.582428666	336	304	302	146	410	336	238	319	Heat shock cognate 71 kDa protein OS=Mus musculus GN=Hspa8 PE=1 SV=1
Q64674	0.894453315	17	14	15	0	23	14	0	18	Spermidine synthase OS=Mus musculus GN=Srm PE=1 SV=1
Q9WVA4	0.754575721	30	20	21	6	33	20	17	22	Transgelin-2 OS=Mus musculus GN=Tagln2 PE=1 SV=4
P35979	0.762222076	21	18	16	4	22	16	15	17	60S ribosomal protein L12 OS=Mus musculus GN=Rpl12 PE=1 SV=2
Q68FL4	0.737214614	11	12	13	2	8	17	8	12	Putative adenosylhomocysteinase 3 OS=Mus musculus GN=Ahcyl2 PE=1 SV=1
Q80SW1	0.748009508	12	11	13	2	8	17	8	12	Putative adenosylhomocysteinase 2 OS=Mus musculus GN=Ahcyl1 PE=1 SV=1
Q3U0V1	0.639196034	5	6	8	3	9	8	3	6	Far upstream element-binding protein 2 OS=Mus musculus GN=Khsrp PE=1 SV=2
A0A1D5RM85	0.198635087	13	15	12	0	12	19	15	17	60S ribosomal protein L18a (Fragment) OS=Mus musculus GN=Rpl18a PE=1 SV=1
A0A0N4SW89	0.858198721	65	43	52	12	69	39	40	54	Annexin (Fragment) OS=Mus musculus GN=Anxa4 PE=1 SV=3
D3Z5E2	0.616351879	105	95	100	43	146	102	73	81	Heat shock cognate 71 kDa protein (Fragment) OS=Mus musculus GN=Hspa8 PE=1 SV=1
P62717	0.186732377	13	15	13	0	13	19	15	17	60S ribosomal protein L18a OS=Mus musculus GN=Rpl18a PE=1 SV=1
Q61316	0.553888381	43	36	38	13	54	39	16	43	Heat shock 70 kDa protein 4 OS=Mus musculus GN=Hspa4 PE=1 SV=1
E9PUX4	0.166043238	38	17	24	0	35	28	30	30	60S ribosomal protein L6 OS=Mus musculus GN=Rpl6l PE=3 SV=1
E9QAZ2	0.630158112	42	40	39	22	39	44	41	43	Ribosomal protein L15 OS=Mus musculus GN=Gm10020 PE=3 SV=1
Q9CZM2	0.630158112	42	40	39	22	39	44	41	43	60S ribosomal protein L15 OS=Mus musculus GN=Rpl15 PE=2 SV=4
P22752	0.990054491	168	101	117	38	146	101	116	132	Histone H2A type 1 OS=Mus musculus GN=Hist1h2ab PE=1 SV=3
Q8CGP6	0.990054491	168	101	117	38	146	101	116	132	Histone H2A type 1-H OS=Mus musculus GN=Hist1h2ah PE=1 SV=3
Q8CGP5	0.990054491	168	101	117	38	146	101	116	132	Histone H2A type 1-F OS=Mus musculus GN=Hist1h2af PE=1 SV=3
V9GXB6	0.025603822	3	3	4	2	0	2	0	5	Heterogeneous nuclear ribonucleoprotein L-like OS=Mus musculus GN=Hnrnpll PE=1 SV=1
A0A0A6YX26	0.186231495	4	0	2	0	3	2	6	3	60S ribosomal protein L31 OS=Mus musculus GN=Rpl31 PE=1 SV=1
P62900	0.186231495	4	0	2	0	3	2	6	3	60S ribosomal protein L31 OS=Mus musculus GN=Rpl31 PE=1 SV=1
A0A087WS46	0.281420615	3	0	0	0	4	0	0	3	Elongation factor 1-beta OS=Mus musculus GN=Eef1b2 PE=1 SV=1
B1AW93	0.412726898	3	0	0	0	3	0	0	4	Protein kinase C and casein kinase II substrate protein 3 (Fragment) OS=Mus musculus GN=Pacsin3 PE=1 SV=1
Q9JII6	0.64395427	0	0	3	0	4	0	0	3	Alcohol dehydrogenase [NADP(+)] OS=Mus musculus GN=Akr1a1 PE=1 SV=3
Q8CIB5	0.644705852	12	13	9	8	15	14	11	9	Fermitin family homolog 2 OS=Mus musculus GN=Fermt2 PE=1 SV=1
H3BJA3	0.856948004	3	3	0	0	0	4	0	3	Glutamate--cysteine ligase regulatory subunit (Fragment) OS=Mus musculus GN=Gclm PE=1 SV=1
P47754	0.875619543	10	4	4	0	7	5	0	9	F-actin-capping protein subunit alpha-2 OS=Mus musculus GN=Capza2 PE=1 SV=3
A0A087WS59	0.097133139	2	0	0	0	3	2	0	2	SLIT-ROBO Rho GTPase-activating protein 2 (Fragment) OS=Mus musculus GN=Srgap2 PE=1 SV=1
F7APP3	0.378232827	0	0	2	0	3	2	0	2	Glycylpeptide N-tetradecanoyltransferase OS=Mus musculus GN=Nmt2 PE=1 SV=1
A2AJH3	0.378232827	0	0	2	0	3	2	0	2	Glycylpeptide N-tetradecanoyltransferase OS=Mus musculus GN=Nmt2 PE=1 SV=1
O70311	0.378232827	0	0	2	0	3	2	0	2	Glycylpeptide N-tetradecanoyltransferase 2 OS=Mus musculus GN=Nmt2 PE=1 SV=1
Q8CGP0	0.655606799	180	155	203	87	175	193	183	177	Histone H2B type 3-B OS=Mus musculus GN=Hist3h2bb PE=1 SV=3
Q8BFU2	0.974570656	168	102	117	38	146	101	116	132	Histone H2A type 3 OS=Mus musculus GN=Hist3h2a PE=1 SV=3
A0A0N4SV66	0.974570656	168	102	117	38	146	101	116	132	Histone H2A OS=Mus musculus GN=H2afj PE=1 SV=1
Q8CGP7	0.974570656	168	102	117	38	146	101	116	132	Histone H2A type 1-K OS=Mus musculus GN=Hist1h2ak PE=1 SV=3
Q61781	0.756661176	18	9	20	8	26	20	11	7	Keratin, type I cytoskeletal 14 OS=Mus musculus GN=Krt14 PE=1 SV=2
Q8R1M2	0.959315359	168	103	117	38	146	101	116	132	Histone H2A.J OS=Mus musculus GN=H2afj PE=1 SV=1
Q3U2G2	0.520271299	43	37	38	13	54	39	16	43	Heat shock 70 kDa protein 4 OS=Mus musculus GN=Hspa4 PE=1 SV=1
P62918	0.613072191	16	13	16	5	19	12	13	14	60S ribosomal protein L8 OS=Mus musculus GN=Rpl8 PE=1 SV=2
P30416	0.984380436	62	42	60	13	60	44	41	60	Peptidyl-prolyl cis-trans isomerase FKBP4 OS=Mus musculus GN=Fkbp4 PE=1 SV=5
P70677	0.888690302	7	7	5	0	6	9	0	7	Caspase-3 OS=Mus musculus GN=Casp3 PE=1 SV=1
A0A0N4SV57	0.954548427	62	43	49	12	66	37	38	51	Annexin (Fragment) OS=Mus musculus GN=Anxa4 PE=1 SV=1
P32883	0.046515762	9	0	7	0	11	7	9	10	GTPase KRas OS=Mus musculus GN=Kras PE=1 SV=1
Q60605	0.897634647	26	21	19	5	22	18	20	22	Myosin light polypeptide 6 OS=Mus musculus GN=Myl6 PE=1 SV=3
P51660	0.420754885	4	4	5	0	0	0	0	5	Peroxisomal multifunctional enzyme type 2 OS=Mus musculus GN=Hsd17b4 PE=1 SV=3

D3YU75	0.931007061	4	2	7	0	5	5	0	5	Translationally-controlled tumor protein OS=Mus musculus GN=Tpt1 PE=1 SV=1
Q9JIW9	0.8955073	12	9	5	0	7	10	0	13	Ras-related protein Ral-B OS=Mus musculus GN=Ralb PE=1 SV=1
B2RXS4	0.878854695	52	33	38	8	46	31	30	44	Plexin-B2 OS=Mus musculus GN=Plxnb2 PE=1 SV=1
Q9QZQ8	0.436999836	13	20	20	6	17	20	14	17	Core histone macro-H2A.1 OS=Mus musculus GN=H2afy PE=1 SV=3
A0A1D5RLW5	0.210136213	13	15	13	0	13	18	15	17	60S ribosomal protein L18a OS=Mus musculus GN=Rpl18a PE=1 SV=1
Q6ZWX6	0.974726714	26	15	27	5	22	20	15	27	Eukaryotic translation initiation factor 2 subunit 1 OS=Mus musculus GN=Eif2s1 PE=1 SV=3
P54754	0.029758739	0	0	5	0	5	7	5	6	Ephrin type-B receptor 3 OS=Mus musculus GN=Ephb3 PE=1 SV=2
F6WHQ7	0.043269315	5	0	0	0	6	6	4	7	Glutathione S-transferase Mu 1 (Fragment) OS=Mus musculus GN=Gstm1 PE=1 SV=1
O55029	0.757372828	24	18	26	6	27	19	13	26	Coatomer subunit beta' OS=Mus musculus GN=Copb2 PE=1 SV=2
Q99NB9	0.802221708	56	33	52	7	46	29	39	56	Splicing factor 3B subunit 1 OS=Mus musculus GN=Sf3b1 PE=1 SV=1
G5E866	0.802221708	56	33	52	7	46	29	39	56	Splicing factor 3B subunit 1 OS=Mus musculus GN=Sf3b1 PE=1 SV=1
A0A1D5RME4	0.142139849	12	11	11	0	13	14	13	12	60S ribosomal protein L18a (Fragment) OS=Mus musculus GN=Rpl18a PE=1 SV=1
P16858	0.501994244	137	109	132	59	182	133	74	112	Glyceraldehyde-3-phosphate dehydrogenase OS=Mus musculus GN=Gapdh PE=1 SV=2
P70333	0.591305889	13	9	15	4	18	8	9	12	Heterogeneous nuclear ribonucleoprotein H2 OS=Mus musculus GN=Hnrnph2 PE=1 SV=1
P11499	0.684884497	354	279	321	81	353	288	214	331	Heat shock protein HSP 90-beta OS=Mus musculus GN=Hsp90ab1 PE=1 SV=3
P17183	0.081115609	23	10	12	4	13	0	0	15	Gamma-enolase OS=Mus musculus GN=Eno2 PE=1 SV=2
P61089	0.272414781	11	5	5	0	11	5	7	9	Ubiquitin-conjugating enzyme E2 N OS=Mus musculus GN=Ube2n PE=1 SV=1
Q8CIE6	0.929327547	77	62	68	10	78	67	36	67	Coatomer subunit alpha OS=Mus musculus GN=Copa PE=1 SV=2
Q9R0Q7	0.949542469	7	6	8	0	9	7	0	8	Prostaglandin E synthase 3 OS=Mus musculus GN=Ptges3 PE=1 SV=1
D3Z7C6	0.949542469	7	6	8	0	9	7	0	8	Prostaglandin E synthase 3 OS=Mus musculus GN=Ptges3 PE=1 SV=1
Q8BFQ4	0.970024987	36	25	37	7	30	27	24	39	WD repeat-containing protein 82 OS=Mus musculus GN=Wdr82 PE=1 SV=1
H3BKR2	0.907641808	47	35	37	8	40	32	32	41	Guanine nucleotide-binding protein G(I)/G(S)/G(T) subunit beta-1 (Fragment) OS=Mus musculus GN=Gnb1 PE=1 SV=8
P27661	0.931170099	147	90	111	35	114	83	116	124	Histone H2AX OS=Mus musculus GN=H2afx PE=1 SV=2
P63087	0.921696715	41	29	32	5	47	26	6	43	Serine/threonine-protein phosphatase PP1-gamma catalytic subunit OS=Mus musculus GN=Ppp1cc PE=1 SV=1
Q5EBP8	0.406496228	20	21	22	10	25	21	16	21	Heterogeneous nuclear ribonucleoprotein A1 OS=Mus musculus GN=Hnrnpa1 PE=1 SV=1
Q9Z2X1	0.253000576	12	11	16	0	23	12	11	13	Heterogeneous nuclear ribonucleoprotein F OS=Mus musculus GN=Hnrnpf PE=1 SV=3
Q3V4D5	0.47731691	6	4	0	0	0	6	6	5	N-acetyltransferase ARD1 homolog (S. cerevisiae), isoform CRA_b OS=Mus musculus GN=Naa10 PE=1 SV=1
B1AUZ1	0.47731691	6	4	0	0	0	6	6	5	N-alpha-acetyltransferase 10 OS=Mus musculus GN=Naa10 PE=1 SV=1
B1AUY9	0.47731691	6	4	0	0	0	6	6	5	N-alpha-acetyltransferase 10 OS=Mus musculus GN=Naa10 PE=1 SV=1
P63323	0.345710184	15	15	9	6	18	15	6	12	40S ribosomal protein S12 OS=Mus musculus GN=Rps12 PE=1 SV=2
P05784	0.918769567	7	0	8	0	8	9	0	0	Keratin, type I cytoskeletal 18 OS=Mus musculus GN=Krt18 PE=1 SV=5
Q61820	0.82169606	56	50	47	5	56	41	35	47	GTP-binding nuclear protein Ran, testis-specific isoform OS=Mus musculus GN=Rasl2-9 PE=2 SV=1
A0A1D5RLD8	0.477988518	126	103	127	59	169	122	74	105	Glyceraldehyde-3-phosphate dehydrogenase OS=Mus musculus PE=1 SV=1
Q6ZWZ6	0.361038106	18	17	13	6	21	20	6	14	40S ribosomal protein S12 OS=Mus musculus GN=Rps12 PE=1 SV=1
Q3TPJ8	0.794371976	8	7	14	2	7	8	10	10	Cytoplasmic dynein 1 intermediate chain 2 OS=Mus musculus GN=Dync1i2 PE=1 SV=1
O88487	0.794371976	8	7	14	2	7	8	10	10	Cytoplasmic dynein 1 intermediate chain 2 OS=Mus musculus GN=Dync1i2 PE=1 SV=1
A2BFF9	0.794371976	8	7	14	2	7	8	10	10	Cytoplasmic dynein 1 intermediate chain 2 OS=Mus musculus GN=Dync1i2 PE=1 SV=1
A2BFF8	0.794371976	8	7	14	2	7	8	10	10	Cytoplasmic dynein 1 intermediate chain 2 OS=Mus musculus GN=Dync1i2 PE=1 SV=1
A2BFF5	0.794371976	8	7	14	2	7	8	10	10	Cytoplasmic dynein 1 intermediate chain 2 OS=Mus musculus GN=Dync1i2 PE=1 SV=1
F7C312	0.105022405	17	12	12	6	16	13	8	16	Endoplasmin (Fragment) OS=Mus musculus GN=Hsp90b1 PE=1 SV=1
P62264	0.525297953	16	11	15	5	20	13	4	16	40S ribosomal protein S14 OS=Mus musculus GN=Rps14 PE=1 SV=3
P05201	0.021068069	2	0	0	0	3	2	2	2	Aspartate aminotransferase, cytoplasmic OS=Mus musculus GN=Got1 PE=1 SV=3
P59708	0.027736255	0	6	6	0	6	8	7	6	Splicing factor 3B subunit 6 OS=Mus musculus GN=Sf3b6 PE=1 SV=1
D3YWR7	0.101888632	0	4	0	0	4	4	5	5	Dihydropteridine reductase OS=Mus musculus GN=Qdpr PE=1 SV=1
A0A0G2JGY0	0.101888632	0	4	0	0	4	4	5	5	Dihydropteridine reductase OS=Mus musculus GN=Qdpr PE=1 SV=1
A0A0G2JGJ1	0.101888632	0	4	0	0	4	4	5	5	Dihydropteridine reductase OS=Mus musculus GN=Qdpr PE=1 SV=1
Q8BVI4	0.101888632	0	4	0	0	4	4	5	5	Dihydropteridine reductase OS=Mus musculus GN=Qdpr PE=1 SV=2
P97379	0.150746139	3	0	5	0	3	7	6	2	Ras GTPase-activating protein-binding protein 2 OS=Mus musculus GN=G3bp2 PE=1 SV=2
O88952	0.244869014	4	0	4	0	3	8	4	3	Protein lin-7 homolog C OS=Mus musculus GN=Lin7c PE=1 SV=2
E9Q4T8	0.280056052	6	3	3	4	0	0	5	4	Cullin-3 OS=Mus musculus GN=Cul3 PE=1 SV=1
Q9JLV5	0.280056052	6	3	3	4	0	0	5	4	Cullin-3 OS=Mus musculus GN=Cul3 PE=1 SV=1
Q3UFY7	0.534694257	4	0	0	0	0	5	0	4	7-methylguanosine phosphate-specific 5'-nucleotidase OS=Mus musculus GN=Nt5c3b PE=1 SV=3
A0A0R4J146	0.534694257	4	0	0	0	0	5	0	4	5'-nucleotidase OS=Mus musculus GN=Nt5c3b PE=1 SV=1
O09167	0.774322215	19	12	13	4	13	17	7	17	60S ribosomal protein L21 OS=Mus musculus GN=Rpl21 PE=1 SV=3
Q9CQM8	0.774322215	19	12	13	4	13	17	7	17	60S ribosomal protein L21 OS=Mus musculus GN=Rpl21 PE=1 SV=1
P61027	0.875436276	8	0	9	7	18	5	7	6	Ras-related protein Rab-10 OS=Mus musculus GN=Rab10 PE=1 SV=1
A0A0J9YUD5	0.244318728	4	4	6	0	7	5	4	5	Protein Nup205 OS=Mus musculus GN=Nup205 PE=1 SV=1
B9EJ54	0.244318728	4	4	6	0	7	5	4	5	MCG21756, isoform CRA_b OS=Mus musculus GN=Nup205 PE=1 SV=1
Q9D0W5	0.185908554	3	3	2	0	3	0	0	0	Peptidyl-prolyl cis-trans isomerase-like 1 OS=Mus musculus GN=Ppil1 PE=1 SV=1
D3Z645	0.789474356	19	10	18	2	16	16	11	12	Vacuolar protein sorting-associated protein 29 OS=Mus musculus GN=Vps29 PE=1 SV=1
D3YYD5	0.789474356	19	10	18	2	16	16	11	12	Vacuolar protein sorting-associated protein 29 (Fragment) OS=Mus musculus GN=Vps29 PE=1 SV=1
Q9QZ88	0.789474356	19	10	18	2	16	16	11	12	Vacuolar protein sorting-associated protein 29 OS=Mus musculus GN=Vps29 PE=1 SV=1
P10853	0.544549303	264	184	253	95	220	227	219	227	Histone H2B type 1-F/J/L OS=Mus musculus GN=Hist1h2bf PE=1 SV=2
P49312	0.370329755	20	22	22	10	25	21	16	21	Heterogeneous nuclear ribonucleoprotein A1 OS=Mus musculus GN=Hnrnpa1 PE=1 SV=2
Q91V55	0.942458587	46	36	33	9	32	34	41	32	40S ribosomal protein S5 OS=Mus musculus GN=Rps5 PE=1 SV=1
Q8CGP2	0.535974232	264	184	253	95	220	227	218	227	Histone H2B type 1-P OS=Mus musculus GN=Hist1h2bp PE=1 SV=3
P10854	0.516902745	264	183	252	95	218	224	217	227	Histone H2B type 1-M OS=Mus musculus GN=Hist1h2bm PE=1 SV=2
P47856	0.315632102	14	12	11	0	12	14	12	17	Glutamine--fructose-6-phosphate aminotransferase [isomerizing] 1 OS=Mus musculus GN=Gfpt1 PE=1 SV=3
Q64475	0.515809314	265	186	254	96	220	227	219	227	Histone H2B type 1-B OS=Mus musculus GN=Hist1h2bb PE=1 SV=3
P97461	0.962796444	46	36	33	9	31	34	41	32	40S ribosomal protein S5 OS=Mus musculus GN=Rps5 PE=1 SV=3
D3YYM6	0.967681259	46	36	33	9	32	33	41	32	40S ribosomal protein S5 (Fragment) OS=Mus musculus GN=Rps5 PE=1 SV=1
D3Z1S8	0.967681259	46	36	33	9	32	33	41	32	40S ribosomal protein S5 (Fragment) OS=Mus musculus GN=Rps5 PE=1 SV=1
F8WIX8	0.707961606	133	94	115	31	106	96	98	115	Histone H2A OS=Mus musculus GN=Hist1h2al PE=3 SV=1
Q64523	0.707961606	133	94	115	31	106	96	98	115	Histone H2A type 2-C OS=Mus musculus GN=Hist2h2ac PE=1 SV=3
Q9JIF7	0.961761946	20	16	9	0	20	16	0	14	Coatomer subunit beta OS=Mus musculus GN=Copb1 PE=1 SV=1
P12815	0.004613724	20	7	12	6	18	6	8	18	Programmed cell death protein 6 OS=Mus musculus GN=Pdcd6 PE=1 SV=2
P97315	0.342825715	0	3	0	0	4	2	0	4	Cysteine and glycine-rich protein 1 OS=Mus musculus GN=Csrp1 PE=1 SV=3
Q6GSS7	0.690000049	133	95	115	31	106	96	98	115	Histone H2A type 2-A OS=Mus musculus GN=Hist2h2aa1 PE=1 SV=3
D3YVM5	0.767631323	56	46	42	13	43	38	40	53	60S acidic ribosomal protein P0 (Fragment) OS=Mus musculus GN=Rplp0 PE=1 SV=1
P62827	0.171603281	211	183	169	63	228	160	123	182	GTP-binding nuclear protein Ran OS=Mus musculus GN=Ran PE=1 SV=3
Q60770	0.329290995	9	6	4	0	7	7	6	8	Syntaxin-binding protein 3 OS=Mus musculus GN=Stxbp3 PE=1 SV=1
Q5SV64	0.264086517	127	92	101	29	125	90	74	0	Myosin-10 OS=Mus musculus GN=Myh10 PE=1 SV=1
P47963	0.223486557	15	9	10	5	12	11	7	13	60S ribosomal protein L13 OS=Mus musculus GN=Rpl13 PE=1 SV=3
A2A9X5	0.980422381	3	3	4	0	4	5	0	2	5'(3')-deoxyribonucleotidase, cytosolic type OS=Mus musculus GN=Nt5c PE=1 SV=1
P84228	0.428887145	79	58	74	25	97	56	54	53	Histone H3.2 OS=Mus musculus GN=Hist1h3b PE=1 SV=2
Q6ZQ73	0.286730464	7	4	4	0	7	4	5	6	Cullin-associated NEDD8-dissociated protein 2 OS=Mus musculus GN=Cand2 PE=1 SV=2
P45376	0.95063047	88	72	93	17	58	68	85	86	Aldose reductase OS=Mus musculus GN=Akr1b1 PE=1 SV=3
P10630	0.990512299	33	21	32	4	27	28	16	28	Eukaryotic initiation factor 4A-II OS=Mus musculus GN=Eif4a2 PE=1 SV=2
B1ARA5	0.438331886	16	17	22	7	25	13	13	17	60S ribosomal protein L26 OS=Mus musculus GN=Rpl26 PE=1 SV=1
Q8BH64	0.458382151	13	3	10	0	8	13	6	11	EH domain-containing protein 2 OS=Mus musculus GN=Ehd2 PE=1 SV=1
P70696	0.490217415	214	165	214	94	170	199	192	191	Histone H2B type 1-A OS=Mus musculus GN=Hist1h2ba PE=1 SV=3
Q9JIM1	0.017470515	0	7	9	0	8	10	10	7	Equilibrative nucleoside transporter 1 OS=Mus musculus GN=Slc29a1 PE=1 SV=3
Q60967	0.242598565	9	7	8	0	7	10	9	9	Bifunctional 3'-phosphoadenosine 5'-phosphosulfate synthase 1 OS=Mus musculus GN=Papss1 PE=1 SV=1
P60710	0.309405752	1195	956	992	449	1057	887	882	1093	Actin, cytoplasmic 1 OS=Mus musculus GN=Actb PE=1 SV=1
P57722	0.169674666	15	11	13	5	16	11	0	9	Poly(rC)-binding protein 3 OS=Mus musculus GN=Pcbp3 PE=1 SV=3
Q9JIZ9	0.998273176	4	3	4	0	4	3	0	5	Phospholipid scramblase 3 OS=Mus musculus GN=Plscr3 PE=1 SV=1
Q5F284	0.998273176	4	3	4	0	4	3	0	5	Phospholipid scramblase (Fragment) OS=Mus musculus GN=Plscr3 PE=1 SV=1
E9Q3V6	0.739069691	20	27	25	6	26	15	21	23	Septin-2 OS=Mus musculus GN=Sept2 PE=1 SV=1
A0A0G2JGC1	0.787991822	33	23	30	5	38	25	3	33	Serine/threonine-protein phosphatase OS=Mus musculus GN=Ppp1cc PE=1 SV=1
Q3UH60	0.078852338	7	6	7	3	5	5	5	10	Disco-interacting protein 2 homolog B OS=Mus musculus GN=Dip2b PE=1 SV=1
P41105	0.254672008	17	10	9	0	16	12	11	13	60S ribosomal protein L28 OS=Mus musculus GN=Rpl28 PE=1 SV=2
Q9WVA3	0.184680969	46	36	39	12	48	40	21	35	Mitotic checkpoint protein BUB3 OS=Mus musculus GN=Bub3 PE=1 SV=2
A0A140LHA2	0.184680969	46	36	39	12	48	40	21	35	Mitotic checkpoint protein BUB3 OS=Mus musculus GN=Bub3 PE=1 SV=1
E0CYQ2	0.185102563	0	0	4	0	0	5	3	5	NudC domain-containing protein 2 OS=Mus musculus GN=Nudcd2 PE=1 SV=1
Q9CQ48	0.185102563	0	0	4	0	0	5	3	5	NudC domain-containing protein 2 OS=Mus musculus GN=Nudcd2 PE=1 SV=1
H3BIX4	0.40140763	0	3	5	0	6	3	4	0	Neuroplastin (Fragment) OS=Mus musculus GN=Nptn PE=1 SV=1
Z4YLB7	0.40140763	0	3	5	0	6	3	4	0	Neuroplastin OS=Mus musculus GN=Nptn PE=1 SV=1
P97300	0.40140763	0	3	5	0	6	3	4	0	Neuroplastin OS=Mus musculus GN=Nptn PE=1 SV=3
P63037	0.306117408	31	27	20	7	28	23	15	26	DnaJ homolog subfamily A member 1 OS=Mus musculus GN=Dnaja1 PE=1 SV=1
G3UYQ2	0.360543257	5	5	9	2	4	5	0	8	Large proline-rich protein BAG6 OS=Mus musculus GN=Bag6 PE=1 SV=1
Q3UF95	0.360543257	5	5	9	2	4	5	0	8	Large proline-rich protein BAG6 OS=Mus musculus GN=Bag6 PE=1 SV=1
A0A1B0GX81	0.360543257	5	5	9	2	4	5	0	8	Large proline-rich protein BAG6 (Fragment) OS=Mus musculus GN=Bag6 PE=1 SV=1
Q9Z1R2	0.360543257	5	5	9	2	4	5	0	8	Large proline-rich protein BAG6 OS=Mus musculus GN=Bag6 PE=1 SV=1
G3UZT6	0.360543257	5	5	9	2	4	5	0	8	Large proline-rich protein BAG6 OS=Mus musculus GN=Bag6 PE=1 SV=1
E9PZB3	0.459899626	26	18	29	7	24	22	14	26	Protein Gm5093 OS=Mus musculus GN=Gm5093 PE=1 SV=1
Q9QZE5	0.261651828	25	25	24	9	32	22	14	21	Coatomer subunit gamma-1 OS=Mus musculus GN=Copg1 PE=1 SV=1
Q9Z0L8	0.59285939	6	5	3	0	5	0	0	5	Gamma-glutamyl hydrolase OS=Mus musculus GN=Ggh PE=1 SV=2
Q8R050	0.327384137	15	16	22	4	17	17	14	13	Eukaryotic peptide chain release factor GTP-binding subunit ERF3A OS=Mus musculus GN=Gspt1 PE=1 SV=2
Q60972	0.311909452	12	15	11	5	14	16	7	9	Histone-binding protein RBBP4 OS=Mus musculus GN=Rbbp4 PE=1 SV=5
Q9D6P8	0.364549945	24	16	14	0	23	16	15	23	Calmodulin-like protein 3 OS=Mus musculus GN=Calml3 PE=2 SV=1
Q6PFB2	0.31722313	9	9	15	0	19	16	8	4	Rcc1 protein OS=Mus musculus GN=Rcc1 PE=1 SV=1
Q8VE37	0.31722313	9	9	15	0	19	16	8	4	Regulator of chromosome condensation OS=Mus musculus GN=Rcc1 PE=1 SV=1
Q9CS42	0.380471177	9	5	5	0	8	9	3	7	Ribose-phosphate pyrophosphokinase 2 OS=Mus musculus GN=Prps2 PE=1 SV=4
E9Q5B6	0.309810304	6	4	0	0	8	4	0	4	Heterogeneous nuclear ribonucleoprotein D0 (Fragment) OS=Mus musculus GN=Hnrnpd PE=1 SV=1
P14148	0.677383208	37	34	29	7	33	24	22	35	60S ribosomal protein L7 OS=Mus musculus GN=Rpl7 PE=1 SV=2
Q8C2Q7	0.291501296	15	11	15	6	20	8	9	13	Heterogeneous nuclear ribonucleoprotein H OS=Mus musculus GN=Hnrnph1 PE=1 SV=1
O35737	0.291501296	15	11	15	6	20	8	9	13	Heterogeneous nuclear ribonucleoprotein H OS=Mus musculus GN=Hnrnph1 PE=1 SV=3
P63028	0.988199605	5	3	8	0	5	5	0	7	Translationally-controlled tumor protein OS=Mus musculus GN=Tpt1 PE=1 SV=1
Q62029	0.36056391	11	7	6	0	14	9	3	8	Polyadenylate-binding protein OS=Mus musculus GN=Pabpc2 PE=1 SV=1
Q99LF4	0.881929849	48	32	44	4	54	33	18	31	tRNA-splicing ligase RtcB homolog OS=Mus musculus GN=Rtcb PE=1 SV=1
Q61879	0.004263292	132	97	111	31	128	93	76	97	Myosin-10 OS=Mus musculus GN=Myh10 PE=1 SV=2
B8JKK2	0.466282528	32	32	28	22	29	32	30	30	Ribosomal protein L15 (Fragment) OS=Mus musculus GN=Rpl15 PE=3 SV=1
P19157	0.058350465	20	19	16	8	19	12	0	19	Glutathione S-transferase P 1 OS=Mus musculus GN=Gstp1 PE=1 SV=2
P21981	0.323082836	5	4	8	0	4	7	6	7	Protein-glutamine gamma-glutamyltransferase 2 OS=Mus musculus GN=Tgm2 PE=1 SV=4
E9Q3T7	0.89921017	6	8	3	0	8	5	0	5	Adenylosuccinate lyase OS=Mus musculus GN=Adsl PE=1 SV=1
P24547	0.889019676	23	17	26	3	17	14	8	34	Inosine-5'-monophosphate dehydrogenase 2 OS=Mus musculus GN=Impdh2 PE=1 SV=2
A0A0U1RQ96	0.301842243	87	62	42	17	67	60	24	69	Actin, gamma-enteric smooth muscle (Fragment) OS=Mus musculus GN=Actg2 PE=3 SV=1
P05480	0.473879705	10	7	5	0	7	7	6	11	Neuronal proto-oncogene tyrosine-protein kinase Src OS=Mus musculus GN=Src PE=1 SV=4
F8WI90	0.473879705	10	7	5	0	7	7	6	11	Tyrosine-protein kinase OS=Mus musculus GN=Src PE=1 SV=1
P60843	0.885560621	64	42	67	7	56	52	25	57	Eukaryotic initiation factor 4A-I OS=Mus musculus GN=Eif4a1 PE=1 SV=1
Q8R422	0.134394586	7	6	8	3	6	5	0	8	CD109 antigen OS=Mus musculus GN=Cd109 PE=1 SV=1
F6ZV59	0.922694978	7	5	6	0	10	4	0	5	Heterogeneous nuclear ribonucleoprotein D0 (Fragment) OS=Mus musculus GN=Hnrnpd PE=1 SV=1
Q3UH59	0.002109539	133	96	111	31	128	92	74	96	Myosin-10 OS=Mus musculus GN=Myh10 PE=1 SV=1
P62911	0.254657137	21	7	12	0	18	13	12	13	60S ribosomal protein L32 OS=Mus musculus GN=Rpl32 PE=1 SV=2
F8WI35	0.572380655	42	0	0	0	0	57	0	31	Histone H3 OS=Mus musculus GN=H3f3a PE=1 SV=1
B2RQC7	0.104195257	7	6	7	3	5	4	5	10	DIP2 disco-interacting protein 2 homolog B (Drosophila) OS=Mus musculus GN=Dip2b PE=1 SV=1
Q3THE2	0.325476089	7	4	8	5	7	5	5	8	Myosin regulatory light chain 12B OS=Mus musculus GN=Myl12b PE=1 SV=2
P50431	0.418297806	21	21	22	9	10	26	19	21	Serine hydroxymethyltransferase, cytosolic OS=Mus musculus GN=Shmt1 PE=1 SV=3
Q8CDN6	0.491769051	13	11	19	8	16	16	12	9	Thioredoxin-like protein 1 OS=Mus musculus GN=Txnl1 PE=1 SV=3
P70698	0.239209901	43	31	42	12	30	32	26	45	CTP synthase 1 OS=Mus musculus GN=Ctps1 PE=1 SV=2
P40237	0.731804255	0	0	4	5	5	0	5	4	CD82 antigen OS=Mus musculus GN=Cd82 PE=1 SV=1
G3UXY0	0.121018971	0	0	9	0	5	12	11	0	Proteasome activator complex subunit 1 OS=Mus musculus GN=Psme1 PE=1 SV=1
P35293	0.959558877	12	6	10	0	11	9	0	9	Ras-related protein Rab-18 OS=Mus musculus GN=Rab18 PE=1 SV=2
P23591	0.482226728	16	14	10	0	15	12	10	18	GDP-L-fucose synthase OS=Mus musculus GN=Tsta3 PE=1 SV=3
G3UZ26	0.371432202	21	21	23	9	10	26	19	21	Serine hydroxymethyltransferase (Fragment) OS=Mus musculus GN=Shmt1 PE=1 SV=1
A2A547	0.531974196	11	13	11	2	14	10	5	9	Ribosomal protein L19 OS=Mus musculus GN=Rpl19 PE=1 SV=1
P84099	0.531974196	11	13	11	2	14	10	5	9	60S ribosomal protein L19 OS=Mus musculus GN=Rpl19 PE=1 SV=1
F6R5R5	0.647496952	11	17	5	4	7	11	10	10	Lysosomal alpha-glucosidase (Fragment) OS=Mus musculus GN=Gaa PE=1 SV=1
Q9DBR1	0.411412456	5	6	8	0	6	6	6	8	5'-3' exoribonuclease 2 OS=Mus musculus GN=Xrn2 PE=1 SV=1
Q14AA6	0.075990944	154	134	142	50	148	120	102	122	GTP-binding nuclear protein Ran OS=Mus musculus GN=1700009N14Rik PE=2 SV=1
P61982	0.236190194	20	22	25	13	19	15	21	27	14-3-3 protein gamma OS=Mus musculus GN=Ywhag PE=1 SV=2
D3YWF6	0.021469055	14	12	12	6	9	10	8	18	Ubiquitin thioesterase OTUB1 OS=Mus musculus GN=Otub1 PE=1 SV=1
Q7TQI3	0.021469055	14	12	12	6	9	10	8	18	Ubiquitin thioesterase OTUB1 OS=Mus musculus GN=Otub1 PE=1 SV=2
contaminant_GR78_MOUSE	0.37501981	34	20	26	8	31	28	8	23	owl|P20029| 78 KD GLUCOSE REGULATED PROTEIN PRECURSOR (GRP 78) (IMMUNOGLOBULIN...
contaminant_GR78_RAT	0.37501981	34	20	26	8	31	28	8	23	owl|P06761| 78 KD GLUCOSE REGULATED PROTEIN PRECURSOR (GRP 78) (IMMUNOGLOBULIN...

F8WHM5	0.464117936	4	3	4	0	4	3	3	5 Golgi apparatus protein 1 (Fragment) OS=Mus musculus GN=Glg1 PE=1 SV=1
Q61543	0.464117936	4	3	4	0	4	3	3	5 Golgi apparatus protein 1 OS=Mus musculus GN=Glg1 PE=1 SV=1
E9Q555	0.165871116	17	17	10	5	13	13	8	16 E3 ubiquitin-protein ligase RNF213 OS=Mus musculus GN=Rnf213 PE=1 SV=2
A0A171EBL2	0.165871116	17	17	10	5	13	13	8	16 E3 ubiquitin-protein ligase RNF213 OS=Mus musculus GN=Rnf213 PE=1 SV=1
Q64737	0.234896263	40	25	29	7	36	21	18	28 Trifunctional purine biosynthetic protein adenosine-3 OS=Mus musculus GN=Gart PE=1 SV=3
D3Z0Y2	0.353945584	26	16	22	3	18	18	0	15 Peroxiredoxin-6 OS=Mus musculus GN=Prdx6 PE=1 SV=1
P62889	0.205077118	20	18	22	10	21	12	17	21 60S ribosomal protein L30 OS=Mus musculus GN=Rpl30 PE=1 SV=2
Q60668	0.182320781	8	8	7	2	10	4	0	5 Heterogeneous nuclear ribonucleoprotein D0 OS=Mus musculus GN=Hnrnpd PE=1 SV=2
P42208	0.508182543	22	30	26	7	27	15	21	23 Septin-2 OS=Mus musculus GN=Sept2 PE=1 SV=2
A3KFU5	0.376058296	10	5	11	0	15	7	6	7 Polyadenylate-binding protein OS=Mus musculus GN=Pabpc4 PE=1 SV=1
Q99JR5	0.398036093	32	25	28	0	43	26	18	27 Tubulointerstitial nephritis antigen-like OS=Mus musculus GN=Tinagl1 PE=1 SV=1
G3X9K9	0.026066285	0	0	9	0	5	12	11	8 Proteasome activator complex subunit 1 OS=Mus musculus GN=Psme1 PE=1 SV=1
O55234	0.122012939	4	0	2	3	0	0	0	3 Proteasome subunit beta type-5 OS=Mus musculus GN=Psmb5 PE=1 SV=3
P24668	0.142575347	11	8	11	0	14	9	10	7 Cation-dependent mannose-6-phosphate receptor OS=Mus musculus GN=M6pr PE=1 SV=1
A3KGK7	0.201149384	3	2	0	0	3	2	0	0 Copine-1 (Fragment) OS=Mus musculus GN=Cpne1 PE=1 SV=1
E9Q3E9	0.201826954	13	16	12	6	12	13	10	12 Staphylococcal nuclease domain-containing protein 1 OS=Mus musculus GN=Snd1 PE=1 SV=1
A0A0A6YWG8	0.236220404	10	7	12	3	9	8	0	7 Actin-like protein 6A (Fragment) OS=Mus musculus GN=Actl6a PE=1 SV=1
D3Z7P0	0.26265147	0	0	3	0	0	2	2	5 BRCA1-A complex subunit BRE OS=Mus musculus GN=Bre PE=1 SV=3
G5E896	0.284393425	3	2	4	0	0	3	0	0 Enhancer of mRNA decapping 4, isoform CRA_b OS=Mus musculus GN=Edc4 PE=1 SV=1
A0A0R4J1Q0	0.284393425	3	2	4	0	0	3	0	0 Enhancer of mRNA-decapping protein 4 OS=Mus musculus GN=Edc4 PE=1 SV=1
Q3UJB9	0.284393425	3	2	4	0	0	3	0	0 Enhancer of mRNA-decapping protein 4 OS=Mus musculus GN=Edc4 PE=1 SV=2
Q99LG2	0.311114306	7	7	4	2	6	0	4	5 Transportin-2 OS=Mus musculus GN=Tnpo2 PE=1 SV=1
A0A0G2JG29	0.328460839	18	14	12	8	11	12	13	16 40S ribosomal protein S27 OS=Mus musculus GN=Rps27 PE=1 SV=1
Q6ZWQ9	0.33079493	7	4	8	5	7	5	5	7 MCG5400 OS=Mus musculus GN=Myl12a PE=1 SV=1
Q91VR8	0.331820455	0	4	4	0	0	4	0	0 Protein BRICK1 OS=Mus musculus GN=Brk1 PE=1 SV=1
contaminant_GR78_HUMAN	0.340267783	34	20	26	8	29	28	8	23 owl|P11021| 78 KD GLUCOSE REGULATED PROTEIN PRECURSOR (GRP 78) (IMMUNOGLOBULIN...
contaminant_GR78_MESAU	0.340267783	34	20	26	8	29	28	8	23 owl|P07823| 78 KD GLUCOSE REGULATED PROTEIN PRECURSOR (GRP 78) (IMMUNOGLOBULIN...
G3XA17	0.366084105	2	0	2	0	2	0	0	0 Eukaryotic translation initiation factor 4 gamma 2 OS=Mus musculus GN=Eif4g2 PE=1 SV=1
F7CBP1	0.366084105	2	0	2	0	2	0	0	0 Eukaryotic translation initiation factor 4 gamma 2 OS=Mus musculus GN=Eif4g2 PE=1 SV=1
Q62448	0.366084105	2	0	2	0	2	0	0	0 Eukaryotic translation initiation factor 4 gamma 2 OS=Mus musculus GN=Eif4g2 PE=1 SV=2
P49722	0.368773216	2	2	0	0	2	0	0	0 Proteasome subunit alpha type-2 OS=Mus musculus GN=Psma2 PE=1 SV=3
B1AW91	0.391002219	3	0	0	0	3	0	0	0 Protein kinase C and casein kinase II substrate protein 3 (Fragment) OS=Mus musculus GN=Pacsin3 PE=1 SV=8
P58281	0.391002219	0	3	0	0	0	3	0	0 Dynamin-like 120 kDa protein, mitochondrial OS=Mus musculus GN=Opa1 PE=1 SV=1
Q8BH04	0.391002219	0	3	0	0	0	3	0	0 Phosphoenolpyruvate carboxykinase [GTP], mitochondrial OS=Mus musculus GN=Pck2 PE=1 SV=1
A0A0R4J0G0	0.391002219	0	3	0	0	0	3	0	0 Phosphoenolpyruvate carboxykinase [GTP], mitochondrial OS=Mus musculus GN=Pck2 PE=1 SV=1
A0A1B0GS09	0.604299512	4	4	0	0	0	0	0	4 Tumor susceptibility gene 101 protein (Fragment) OS=Mus musculus GN=Tsg101 PE=1 SV=1
B1AWD8	0.662691541	0	0	0	8	0	0	8	0 Clathrin light chain A OS=Mus musculus GN=Clta PE=1 SV=1
Q99K70	0.662691541	0	0	0	2	0	0	2	0 Ras-related GTP-binding protein C OS=Mus musculus GN=Rragc PE=1 SV=1
Q7TSV4	0.680880204	2	0	2	0	0	2	0	0 Phosphoglucomutase-2 OS=Mus musculus GN=Pgm2 PE=1 SV=1
P27612	0.748382103	3	0	5	0	2	3	0	7 Phospholipase A-2-activating protein OS=Mus musculus GN=Plaa PE=1 SV=4
Q8C0E3	0.790432547	0	3	7	0	6	5	0	4 Tripartite motif-containing protein 47 OS=Mus musculus GN=Trim47 PE=1 SV=2
Q9CQ09	0.808016376	13	0	0	0	0	0	13	0 Protein Rpl3l OS=Mus musculus GN=Rpl3l PE=1 SV=1
Q3TU36	0.849886839	0	2	0	0	2	0	0	0 Protein Rap1gds1 OS=Mus musculus GN=Rap1gds1 PE=1 SV=1
A0A0J9YUM4	0.849886839	0	2	0	0	2	0	0	0 C-terminal-binding protein 1 OS=Mus musculus GN=Ctbp1 PE=1 SV=1
Q9DBG5	0.849886839	0	3	0	0	3	0	0	0 Perilipin-3 OS=Mus musculus GN=Plin3 PE=1 SV=1
O88712	0.849886839	0	2	0	0	2	0	0	0 C-terminal-binding protein 1 OS=Mus musculus GN=Ctbp1 PE=1 SV=2
A0A0G2JGC8	0.849886839	0	2	0	0	2	0	0	0 Protein Rap1gds1 OS=Mus musculus GN=Rap1gds1 PE=1 SV=1
E9Q912	0.849886839	0	2	0	0	2	0	0	0 Protein Rap1gds1 OS=Mus musculus GN=Rap1gds1 PE=1 SV=1
E9Q6Q4	0.849886839	0	2	0	0	2	0	0	0 Protein Rap1gds1 OS=Mus musculus GN=Rap1gds1 PE=1 SV=1
S4R2C4	0.849886839	0	2	0	0	2	0	0	0 Son of sevenless homolog 2 OS=Mus musculus GN=Sos2 PE=1 SV=1
A0A0J9YU62	0.849886839	0	2	0	0	2	0	0	0 C-terminal-binding protein 1 OS=Mus musculus GN=Ctbp1 PE=1 SV=1
H3BIV7	0.858008374	3	5	0	0	5	0	0	3 Serine/threonine-protein phosphatase 2A 65 kDa regulatory subunit A beta isoform OS=Mus musculus GN=Ppp2r1b PE=1 SV=1
J3QQ30	0.893353577	3	0	3	0	0	0	2	4 Phosphatidylinositol transfer protein alpha isoform OS=Mus musculus GN=Pitpna PE=1 SV=1
J3QPW1	0.893353577	3	0	3	0	0	0	2	4 Phosphatidylinositol transfer protein alpha isoform OS=Mus musculus GN=Pitpna PE=1 SV=1
P53810	0.893353577	3	0	3	0	0	0	2	4 Phosphatidylinositol transfer protein alpha isoform OS=Mus musculus GN=Pitpna PE=1 SV=2
A0A0G2JF31	0.900140637	0	0	2	0	2	0	0	0 Thymidylate synthase (Fragment) OS=Mus musculus GN=Tyms PE=1 SV=1
Q8BWU5	0.903426338	0	2	2	0	0	2	0	2 Probable tRNA N6-adenosine threonylcarbamoyltransferase OS=Mus musculus GN=Osgep PE=1 SV=2
E0CYN9	0.903426338	0	2	2	0	0	2	0	2 Probable tRNA N6-adenosine threonylcarbamoyltransferase OS=Mus musculus GN=Osgep PE=1 SV=1
A0A0R4J1Y3	0.903426338	0	2	2	0	0	2	0	2 Probable tRNA N6-adenosine threonylcarbamoyltransferase OS=Mus musculus GN=Osgep PE=1 SV=1
G3UXT8	0.906431553	4	5	7	0	4	5	0	7 Large proline-rich protein BAG6 (Fragment) OS=Mus musculus GN=Bag6 PE=1 SV=1
Q9CY64	0.908765346	4	3	3	0	3	3	0	4 Biliverdin reductase A OS=Mus musculus GN=Blvra PE=1 SV=1
F6YA11	0.919995716	13	9	10	0	9	10	0	13 Exportin-1 (Fragment) OS=Mus musculus GN=Xpo1 PE=1 SV=1
Q9JJK2	0.921450104	3	0	0	0	0	3	0	0 LanC-like protein 2 OS=Mus musculus GN=Lancl2 PE=1 SV=1
Q8BP48	0.921450104	2	0	0	0	0	2	0	0 Methionine aminopeptidase 1 OS=Mus musculus GN=Metap1 PE=1 SV=1
F6RJV6	0.921450104	3	0	0	0	0	3	0	0 LanC-like protein 2 (Fragment) OS=Mus musculus GN=Lancl2 PE=1 SV=1
D6RE33	0.921450104	3	0	0	0	0	3	0	0 Enhancer of mRNA-decapping protein 4 OS=Mus musculus GN=Edc4 PE=1 SV=1
Q9CXY6	0.921450104	3	0	0	0	0	3	0	0 Interleukin enhancer-binding factor 2 OS=Mus musculus GN=Ilf2 PE=1 SV=1
Q9CT10	0.92840486	0	2	0	0	0	0	2	0 Ran-binding protein 3 OS=Mus musculus GN=Ranbp3 PE=1 SV=2
P62830	0.943602506	4	3	3	0	5	0	2	3 60S ribosomal protein L23 OS=Mus musculus GN=Rpl23 PE=1 SV=1
P61082	0.951487156	6	3	3	0	3	4	0	5 NEDD8-conjugating enzyme Ubc12 OS=Mus musculus GN=Ube2m PE=1 SV=1
F7CDT0	0.951487156	6	3	3	0	3	4	0	5 NEDD8-conjugating enzyme Ubc12 (Fragment) OS=Mus musculus GN=Ube2m PE=1 SV=1
F6WMC0	0.951765864	5	3	2	0	2	3	0	5 NEDD8-conjugating enzyme Ubc12 (Fragment) OS=Mus musculus GN=Ube2m PE=1 SV=1
Q5SPX8	0.958049947	0	0	3	0	0	0	0	3 TBC1 domain family member 10A OS=Mus musculus GN=Tbc1d10a PE=1 SV=1
F6W5Q8	0.958049947	0	0	3	0	0	0	0	3 Vacuolar protein sorting-associated protein VTA1 homolog (Fragment) OS=Mus musculus GN=Vta1 PE=1 SV=8
P58802	0.958049947	0	0	3	0	0	0	0	3 TBC1 domain family member 10A OS=Mus musculus GN=Tbc1d10a PE=1 SV=1
Q9CR26	0.958049947	0	0	3	0	0	0	0	3 Vacuolar protein sorting-associated protein VTA1 homolog OS=Mus musculus GN=Vta1 PE=1 SV=1
Q9QYJ3	0.966634205	4	0	0	0	0	0	0	4 DnaJ homolog subfamily B member 1 OS=Mus musculus GN=Dnajb1 PE=1 SV=3
P70697	0.966634205	3	0	0	0	0	0	0	3 Uroporphyrinogen decarboxylase OS=Mus musculus GN=Urod PE=1 SV=2
Q3TYL7	0.966634205	4	0	0	0	0	0	0	4 DnaJ homolog subfamily B member 1 OS=Mus musculus GN=Dnajb1 PE=1 SV=1
P62301	0.983202751	5	5	6	0	5	0	5	6 40S ribosomal protein S13 OS=Mus musculus GN=Rps13 PE=1 SV=2
Q921R2	0.983202751	5	5	6	0	5	0	5	6 40S ribosomal protein S13 OS=Mus musculus GN=Rps13 PE=1 SV=1
Q9CR51	0.996337707	0	0	3	0	0	3	0	0 V-type proton ATPase subunit G 1 OS=Mus musculus GN=Atp6v1g1 PE=1 SV=3
P68134	0.277358431	814	671	736	396	621	640	643	711 Actin, alpha skeletal muscle OS=Mus musculus GN=Acta1 PE=1 SV=1
P68033	0.271848296	823	675	742	397	629	644	644	717 Actin, alpha cardiac muscle 1 OS=Mus musculus GN=Actc1 PE=1 SV=1
O08709	0.345638779	30	18	23	3	19	18	0	18 Peroxiredoxin-6 OS=Mus musculus GN=Prdx6 PE=1 SV=3
Q6GT24	0.345638779	30	18	23	3	19	18	0	18 Peroxiredoxin 6 OS=Mus musculus GN=Prdx6 PE=1 SV=1
P08113	0.006887119	29	23	26	9	25	20	14	27 Endoplasmin OS=Mus musculus GN=Hsp90b1 PE=1 SV=2
Q9CPY7	0.110246711	48	49	49	17	53	34	25	49 Cytosol aminopeptidase OS=Mus musculus GN=Lap3 PE=1 SV=3
Q9R1P4	0.402998733	7	8	4	0	5	9	5	6 Proteasome subunit alpha type-1 OS=Mus musculus GN=Psma1 PE=1 SV=1
Q11011	0.584092951	36	20	17	4	27	14	14	21 Puromycin-sensitive aminopeptidase OS=Mus musculus GN=Npepps PE=1 SV=2
Q9D662	0.138023607	8	8	4	3	0	5	3	9 Protein transport protein Sec23B OS=Mus musculus GN=Sec23b PE=1 SV=1
P70441	0.201281242	4	6	8	5	7	0	4	6 Na(+)/H(+) exchange regulatory cofactor NHE-RF1 OS=Mus musculus GN=Slc9a3r1 PE=1 SV=3
Q8BHF5	0.271063517	16	10	15	5	11	13	10	0 Reticulon OS=Mus musculus GN=Rtn4 PE=1 SV=1
D3YW98	0.24337829	13	6	13	0	11	13	11	7 Vacuolar protein sorting-associated protein 29 OS=Mus musculus GN=Vps29 PE=1 SV=1
P62137	0.467029562	62	44	51	11	66	33	11	55 Serine/threonine-protein phosphatase PP1-alpha catalytic subunit OS=Mus musculus GN=Ppp1ca PE=1 SV=1
H3BJ97	0.438381486	32	25	28	0	40	26	18	27 Tubulointerstitial nephritis antigen-like OS=Mus musculus GN=Tinagl1 PE=1 SV=1
Q9Z0N1	0.360311356	54	43	41	8	41	39	26	37 Eukaryotic translation initiation factor 2 subunit 3, X-linked OS=Mus musculus GN=Eif2s3x PE=1 SV=2
P68368	0.284209651	212	142	144	33	168	139	71	142 Tubulin alpha-4A chain OS=Mus musculus GN=Tuba4a PE=1 SV=1
Q99P72	0.224102373	16	10	15	5	11	13	10	11 Reticulon-4 OS=Mus musculus GN=Rtn4 PE=1 SV=2
Q9JHU4	0.379211428	324	244	274	53	248	220	153	254 Cytoplasmic dynein 1 heavy chain 1 OS=Mus musculus GN=Dync1h1 PE=1 SV=2
P17427	0.196508308	10	6	11	3	7	6	0	9 AP-2 complex subunit alpha-2 OS=Mus musculus GN=Ap2a2 PE=1 SV=2
P62702	0.593826007	92	64	87	14	55	65	53	78 40S ribosomal protein S4, X isoform OS=Mus musculus GN=Rps4x PE=1 SV=2
Q6ZWY3	0.882641469	14	8	10	0	10	12	0	9 40S ribosomal protein S27-like OS=Mus musculus GN=Rps27l PE=1 SV=3
P54923	0.596199917	14	10	7	0	10	10	6	14 [Protein ADP-ribosylarginine] hydrolase OS=Mus musculus GN=Adprh PE=1 SV=1
Q9DCH4	0.642524164	14	6	18	0	12	13	7	17 Eukaryotic translation initiation factor 3 subunit F OS=Mus musculus GN=Eif3f PE=1 SV=2
D3Z795	0.016341053	13	6	7	3	6	0	0	8 Proteasome assembly chaperone 1 OS=Mus musculus GN=Psmg1 PE=1 SV=1
Q9JK23	0.016341053	13	6	7	3	6	0	0	8 Proteasome assembly chaperone 1 OS=Mus musculus GN=Psmg1 PE=1 SV=1
A0A1B0GSA8	0.930215017	19	18	19	0	0	21	16	17 60S ribosomal protein L18 (Fragment) OS=Mus musculus GN=Rpl18 PE=1 SV=1
D3YYB1	0.332587356	13	20	15	7	18	9	12	14 Septin-2 (Fragment) OS=Mus musculus GN=Sept2 PE=1 SV=1
Q9CQ19	0.356692388	5	3	6	0	4	0	5	0 Myosin regulatory light polypeptide 9 OS=Mus musculus GN=Myl9 PE=1 SV=3
P70460	0.698191964	0	5	4	0	7	3	3	0 Vasodilator-stimulated phosphoprotein OS=Mus musculus GN=Vasp PE=1 SV=4
G3X9V0	0.017822702	0	0	6	0	5	6	6	6 MCG22048, isoform CRA_a OS=Mus musculus GN=Psme2 PE=1 SV=1
Q62167	0.36984196	23	14	30	4	18	14	0	19 ATP-dependent RNA helicase DDX3X OS=Mus musculus GN=Ddx3x PE=1 SV=3
A2AGN7	0.548598187	22	20	24	3	23	16	8	19 26S protease regulatory subunit 6A OS=Mus musculus GN=Psmc3 PE=1 SV=1
Q8BFZ3	0.145751506	293	208	186	50	252	153	110	189 Beta-actin-like protein 2 OS=Mus musculus GN=Actbl2 PE=1 SV=1
Q9D0M1	0.618648267	9	5	8	0	10	5	4	9 Phosphoribosyl pyrophosphate synthase-associated protein 1 OS=Mus musculus GN=Prpsap1 PE=1 SV=1
B1AT82	0.618648267	9	5	8	0	10	5	4	9 MCG6846, isoform CRA_c OS=Mus musculus GN=Prpsap1 PE=1 SV=1
P61202	0.532304475	3	2	6	0	0	4	3	0 COP9 signalosome complex subunit 2 OS=Mus musculus GN=Cops2 PE=1 SV=1
contaminant_BASI_MOUSE	0.341094864	22	20	17	5	12	17	15	17 owl|P18572| BASIGIN PRECURSOR (BASIC IMMUNOGLOBULIN SUPERFAMILY) (MEMBRANE...
E9QN31	0.113140828	22	19	17	4	21	15	9	14 Probable 28S rRNA (cytosine-C(5))-methyltransferase OS=Mus musculus GN=Nop2 PE=1 SV=1
D3Z722	0.666612815	13	8	9	0	13	5	6	14 40S ribosomal protein S19 OS=Mus musculus GN=Rps19 PE=1 SV=1
D3YUT3	0.666612815	13	8	9	0	13	5	6	14 40S ribosomal protein S19 (Fragment) OS=Mus musculus GN=Rps19 PE=1 SV=2
D3YUG3	0.666612815	13	8	9	0	13	5	6	14 40S ribosomal protein S19 (Fragment) OS=Mus musculus GN=Rps19 PE=1 SV=8
D3Z5R8	0.666612815	13	8	9	0	13	5	6	14 40S ribosomal protein S19 (Fragment) OS=Mus musculus GN=Rps19 PE=1 SV=8
Q9CZX8	0.666612815	13	8	9	0	13	5	6	14 40S ribosomal protein S19 OS=Mus musculus GN=Rps19 PE=1 SV=3
O08573	0.908287558	4	7	9	0	8	0	6	5 Galectin-9 OS=Mus musculus GN=Lgals9 PE=1 SV=1
B1AQR8	0.908287558	4	7	9	0	8	0	6	5 Galectin OS=Mus musculus GN=Lgals9 PE=1 SV=1
G3X9T7	0.908287558	4	7	9	0	8	0	6	5 Galectin OS=Mus musculus GN=Lgals9 PE=1 SV=1
Q6NZM8	0.345659781	9	7	7	0	7	9	7	6 3'-phosphoadenosine 5'-phosphosulfate synthase 1, isoform CRA_a OS=Mus musculus GN=Papss1 PE=1 SV=1
Q62433	0.384456112	7	11	13	0	12	11	9	7 Protein NDRG1 OS=Mus musculus GN=Ndrg1 PE=1 SV=1
Q5U4C5	0.430310755	8	4	4	0	8	3	5	4 Importin-4 OS=Mus musculus GN=Ipo4 PE=1 SV=1
P54761	0.034542464	3	0	5	0	4	3	5	3 Ephrin type-B receptor 4 OS=Mus musculus GN=Ephb4 PE=1 SV=2
Q8C8K1	0.034542464	3	0	5	0	4	3	5	3 Ephrin type-B receptor 4 OS=Mus musculus GN=Ephb4 PE=1 SV=1
E9PWK7	0.034542464	3	0	5	0	4	3	5	3 Ephrin type-B receptor 4 OS=Mus musculus GN=Ephb4 PE=1 SV=1
A0A0J9YUI1	0.18107936	0	0	4	0	3	6	2	4 Ubiquitin-conjugating enzyme E2 K OS=Mus musculus GN=Ube2k PE=1 SV=1
P61087	0.18107936	0	0	4	0	3	6	2	4 Ubiquitin-conjugating enzyme E2 K OS=Mus musculus GN=Ube2k PE=1 SV=3
Reverse_sp|A2ASS6|TITIN_MOUSE	0.074215139	0	4	2	2	0	2	0	3 Titin OS=Mus musculus GN=Ttn PE=1 SV=1
Q3TW96	0.31862569	18	13	14	2	14	14	7	9 UDP-N-acetylhexosamine pyrophosphorylase-like protein 1 OS=Mus musculus GN=Uap1l1 PE=1 SV=1
Q8BH78	0.226490696	16	10	15	5	11	13	10	9 Reticulon OS=Mus musculus GN=Rtn4 PE=1 SV=1
Q76MZ3	0.058950198	80	46	74	25	69	48	34	59 Serine/threonine-protein phosphatase 2A 65 kDa regulatory subunit A alpha isoform OS=Mus musculus GN=Ppp2r1a PE=1 SV=3
D3Z5M2	0.08334386	10	0	0	0	15	0	6	7 Polyadenylate-binding protein OS=Mus musculus GN=Gm10110 PE=3 SV=1
A3KFU8	0.08334386	10	0	0	0	15	0	6	7 Polyadenylate-binding protein OS=Mus musculus GN=Pabpc4 PE=1 SV=1
G3UXZ5	0.133904801	0	0	10	0	5	12	11	0 Proteasome activator complex subunit 1 (Fragment) OS=Mus musculus GN=Psme1 PE=1 SV=1
Q8BP47	0.677654581	46	29	25	4	26	20	18	33 Asparagine--tRNA ligase, cytoplasmic OS=Mus musculus GN=Nars PE=1 SV=2
E9PYI8	0.485397729	10	4	13	2	10	6	4	7 Ubiquitin carboxyl-terminal hydrolase 14 OS=Mus musculus GN=Usp14 PE=1 SV=1
Q9JMA1	0.485397729	10	4	13	2	10	6	4	7 Ubiquitin carboxyl-terminal hydrolase 14 OS=Mus musculus GN=Usp14 PE=1 SV=3
P62245	0.210810328	49	27	28	11	27	23	22	35 40S ribosomal protein S15a OS=Mus musculus GN=Rps15a PE=1 SV=2
Q61753	0.005640006	87	66	69	18	81	57	42	43 D-3-phosphoglycerate dehydrogenase OS=Mus musculus GN=Phgdh PE=1 SV=3
Q91V89	0.27243126	19	20	13	4	14	13	10	15 Protein Ppp2r5d OS=Mus musculus GN=Ppp2r5d PE=1 SV=1
P14869	0.151537615	76	56	55	18	50	38	42	59 60S acidic ribosomal protein P0 OS=Mus musculus GN=Rplp0 PE=1 SV=3
Q6ZQ38	0.098024777	17	12	17	5	12	11	7	17 Cullin-associated NEDD8-dissociated protein 1 OS=Mus musculus GN=Cand1 PE=1 SV=2
F8WJ41	0.208063434	49	27	27	11	26	23	21	35 40S ribosomal protein S15a (Fragment) OS=Mus musculus GN=Rps15a PE=1 SV=1
P68040	0.517662236	170	91	116	19	106	102	40	116 Receptor of activated protein C kinase 1 OS=Mus musculus GN=Rack1 PE=1 SV=3
F6ZLC6	0.255452975	24	21	16	13	29	14	10	15 Histone-binding protein RBBP7 (Fragment) OS=Mus musculus GN=Rbbp7 PE=1 SV=8
S4R1N1	0.108066756	67	49	48	18	45	33	38	51 60S acidic ribosomal protein P0 (Fragment) OS=Mus musculus GN=Rplp0 PE=1 SV=1
B1AT83	0.612418766	6	4	8	0	7	5	4	6 Phosphoribosyl pyrophosphate synthase-associated protein 1 (Fragment) OS=Mus musculus GN=Prpsap1 PE=1 SV=1
P62852	0.239995612	8	0	0	0	7	7	0	8 40S ribosomal protein S25 OS=Mus musculus GN=Rps25 PE=1 SV=1
Q8R1B4	0.040790369	41	27	38	9	30	26	21	28 Eukaryotic translation initiation factor 3 subunit C OS=Mus musculus GN=Eif3c PE=1 SV=1
P31938	0.073415372	0	8	9	0	6	10	8	7 Dual specificity mitogen-activated protein kinase kinase 1 OS=Mus musculus GN=Map2k1 PE=1 SV=2
Q91WK2	0.036436971	6	6	5	2	3	5	0	5 Eukaryotic translation initiation factor 3 subunit H OS=Mus musculus GN=Eif3h PE=1 SV=1
P54822	0.704748381	6	11	5	0	10	5	0	5 Adenylosuccinate lyase OS=Mus musculus GN=Adsl PE=1 SV=2
O54984	0.818275949	7	6	9	0	5	9	0	6 ATPase Asna1 OS=Mus musculus GN=Asna1 PE=1 SV=2
P70699	0.501161657	15	18	7	4	7	11	11	11 Lysosomal alpha-glucosidase OS=Mus musculus GN=Gaa PE=1 SV=2
Q3TRH2	0.454597453	21	16	14	3	13	10	12	14 26S proteasome non-ATPase regulatory subunit 12 OS=Mus musculus GN=Psmd12 PE=1 SV=1
Q62419	0.707865198	5	4	2	0	4	3	0	3 Endophilin-A2 OS=Mus musculus GN=Sh3gl1 PE=1 SV=1

Q922K7	0.069687512	22	19	17	4	18	15	9	14	Probable 28S rRNA (cytosine-C(5))-methyltransferase OS=Mus musculus GN=Nop2 PE=1 SV=1
P61161	0.177859577	43	35	43	11	35	40	13	31	Actin-related protein 2 OS=Mus musculus GN=Actr2 PE=1 SV=1
Q8C863	0.827783213	5	3	2	0	2	3	0	4	E3 ubiquitin-protein ligase Itchy OS=Mus musculus GN=Itch PE=1 SV=2
F6YY88	0.893453785	3	4	3	0	0	5	2	2	Methylthioribose-1-phosphate isomerase (Fragment) OS=Mus musculus GN=Mri1 PE=1 SV=1
P97371	0.032810954	0	0	10	0	5	12	11	8	Proteasome activator complex subunit 1 OS=Mus musculus GN=Psme1 PE=1 SV=2
Q7TNP2	0.146299267	4	8	6	2	6	0	0	3	Serine/threonine-protein phosphatase 2A 65 kDa regulatory subunit A beta isoform OS=Mus musculus GN=Ppp2r1b PE=1 SV=2
A0A0J9YTQ8	0.361396015	0	0	5	0	0	5	4	0	Ras GTPase-activating protein-binding protein 2 (Fragment) OS=Mus musculus GN=G3bp2 PE=1 SV=1
Q8BT60	0.363210692	6	15	5	4	8	4	4	11	Copine-3 OS=Mus musculus GN=Cpne3 PE=1 SV=2
B8JK33	0.557812127	10	0	0	0	0	11	7	0	Heterogeneous nuclear ribonucleoprotein M OS=Mus musculus GN=Hnrnpm PE=1 SV=1
D3YTQ9	0.569574268	0	0	7	3	5	0	4	0	40S ribosomal protein S15 OS=Mus musculus GN=Rps15 PE=1 SV=1
P62843	0.569574268	0	0	7	3	5	0	4	0	40S ribosomal protein S15 OS=Mus musculus GN=Rps15 PE=1 SV=2
A2AFI9	0.145138141	28	24	24	13	33	17	10	20	Histone-binding protein RBBP7 OS=Mus musculus GN=Rbbp7 PE=1 SV=1
A2AFJ1	0.114177873	27	26	24	11	32	17	10	20	Histone-binding protein RBBP7 OS=Mus musculus GN=Rbbp7 PE=1 SV=1
A0A1B0GSG5	0.342161783	26	18	21	3	22	11	12	16	Ribonuclease inhibitor OS=Mus musculus GN=Rnh1 PE=1 SV=1
Q91VI7	0.342161783	26	18	21	3	22	11	12	16	Ribonuclease inhibitor OS=Mus musculus GN=Rnh1 PE=1 SV=1
P23116	0.000334455	69	48	55	19	55	36	31	49	Eukaryotic translation initiation factor 3 subunit A OS=Mus musculus GN=Eif3a PE=1 SV=5
O70475	0.109873106	32	23	24	6	31	14	11	20	UDP-glucose 6-dehydrogenase OS=Mus musculus GN=Ugdh PE=1 SV=1
G3UY38	0.10204498	15	14	14	5	13	14	0	5	Heterogeneous nuclear ribonucleoprotein L OS=Mus musculus GN=Hnrnpl PE=1 SV=1
E9QAI5	0.094462382	40	26	40	11	26	31	21	26	CAD protein OS=Mus musculus GN=Cad PE=1 SV=1
B2RQC6	0.094462382	40	26	40	11	26	31	21	26	CAD protein OS=Mus musculus GN=Cad PE=1 SV=1
P48722	0.901585849	0	4	5	0	5	3	0	4	Heat shock 70 kDa protein 4L OS=Mus musculus GN=Hspa4l PE=1 SV=2
H3BKS8	0.866523927	7	4	7	0	5	5	6	0	tRNA (guanine(26)-N(2))-dimethyltransferase OS=Mus musculus GN=Trmt1 PE=1 SV=1
E9PWD4	0.866523927	7	4	7	0	5	5	6	0	tRNA (guanine(26)-N(2))-dimethyltransferase OS=Mus musculus GN=Trmt1 PE=1 SV=1
A0A0R4IZW7	0.866523927	7	4	7	0	5	5	6	0	tRNA (guanine(26)-N(2))-dimethyltransferase OS=Mus musculus GN=Trmt1 PE=1 SV=1
Q3TX08	0.866523927	7	4	7	0	5	5	6	0	tRNA (guanine(26)-N(2))-dimethyltransferase OS=Mus musculus GN=Trmt1 PE=1 SV=2
P48758	0.581043745	11	8	14	0	12	13	7	7	Carbonyl reductase [NADPH] 1 OS=Mus musculus GN=Cbr1 PE=1 SV=3
Q3THW5	0.413592864	56	29	34	3	28	0	27	26	Histone H2A.V OS=Mus musculus GN=H2afv PE=1 SV=3
G5E924	0.153259497	15	15	18	5	14	16	0	5	Heterogeneous nuclear ribonucleoprotein L (Fragment) OS=Mus musculus GN=Hnrnpl PE=1 SV=1
Q8R081	0.153259497	15	15	18	5	14	16	0	5	Heterogeneous nuclear ribonucleoprotein L OS=Mus musculus GN=Hnrnpl PE=1 SV=2
P58252	0.004311816	399	264	287	89	315	191	161	247	Elongation factor 2 OS=Mus musculus GN=Eef2 PE=1 SV=2
A0A0G2JE29	0.200586314	22	14	17	4	21	7	6	16	Procollagen C-endopeptidase enhancer 1 (Fragment) OS=Mus musculus GN=Pcolce PE=1 SV=1
P0C0S6	0.668678516	56	29	34	3	28	26	27	26	Histone H2A.Z OS=Mus musculus GN=H2afz PE=1 SV=2
Q3UA72	0.577418527	4	0	0	0	0	0	4	3	Actin-related protein 2/3 complex subunit 5 OS=Mus musculus GN=Arpc5 PE=1 SV=1
Q9CPW4	0.577418527	4	0	0	0	0	0	4	3	Actin-related protein 2/3 complex subunit 5 OS=Mus musculus GN=Arpc5 PE=1 SV=3
Q8K0C9	0.171331292	11	14	19	3	11	14	8	8	GDP-mannose 4,6 dehydratase OS=Mus musculus GN=Gmds PE=1 SV=1
Q5SUR0	0.014697201	24	19	22	5	18	15	13	15	Phosphoribosylformylglycinamidine synthase OS=Mus musculus GN=Pfas PE=1 SV=1
A0A1B0GSE8	0.043916264	22	14	20	5	17	15	10	11	40S ribosomal protein S11 (Fragment) OS=Mus musculus GN=Rps11 PE=1 SV=1
P47753	0.336117213	12	14	4	8	11	7	7	8	F-actin-capping protein subunit alpha-1 OS=Mus musculus GN=Capza1 PE=1 SV=4
Q5RKN9	0.336117213	12	14	4	8	11	7	7	8	Capping protein (Actin filament) muscle Z-line, alpha 1 OS=Mus musculus GN=Capza1 PE=1 SV=1
A0A0G2JH04	0.059457398	17	9	18	9	0	10	0	13	Eukaryotic translation initiation factor 4E OS=Mus musculus GN=Eif4e PE=1 SV=1
Q8R5C5	0.601295471	35	21	25	2	21	16	11	24	Beta-centractin OS=Mus musculus GN=Actr1b PE=1 SV=1
E9PV58	0.868441856	6	6	3	0	4	0	4	5	Transportin-2 OS=Mus musculus GN=Tnpo2 PE=1 SV=1
Q9JMA2	0.708373477	8	8	6	0	5	7	0	7	Queuine tRNA-ribosyltransferase catalytic subunit 1 OS=Mus musculus GN=Qtrt1 PE=1 SV=2
Q60973	0.113172037	29	27	26	13	34	17	10	21	Histone-binding protein RBBP7 OS=Mus musculus GN=Rbbp7 PE=1 SV=1
O55135	0.485588393	20	12	24	2	16	14	8	12	Eukaryotic translation initiation factor 6 OS=Mus musculus GN=Eif6 PE=1 SV=2
A0A140T8K6	0.520021707	7	5	8	0	5	7	6	5	60S ribosomal protein L36 OS=Mus musculus GN=Rpl36-ps3 PE=3 SV=1
Q6ZWZ4	0.520021707	7	5	8	0	5	7	6	5	60S ribosomal protein L36 OS=Mus musculus GN=Rpl36 PE=1 SV=1
Q8C2E7	0.283999553	3	2	2	0	0	0	2	0	WASH complex subunit strumpellin OS=Mus musculus GN=Kiaa0196 PE=1 SV=2
Q9D0D4	0.316794996	2	2	3	0	0	0	0	2	Probable dimethyladenosine transferase OS=Mus musculus GN=Dimt1 PE=2 SV=1
Q00PI9	0.321351902	2	3	2	0	0	0	0	2	Heterogeneous nuclear ribonucleoprotein U-like protein 2 OS=Mus musculus GN=Hnrnpul2 PE=1 SV=2
A0A0U1RNK6	0.043733859	13	10	9	3	13	7	3	7	S-adenosylmethionine synthase OS=Mus musculus GN=Mat2a PE=1 SV=1
F8VPK0	0.305822745	7	4	10	0	5	7	10	2	Protein Ttc37 OS=Mus musculus GN=Ttc37 PE=1 SV=1
Q60749	0.467116922	0	3	4	0	3	0	0	0	KH domain-containing, RNA-binding, signal transduction-associated protein 1 OS=Mus musculus GN=Khdrbs1 PE=1 SV=2
P57716	0.570431153	4	0	3	0	0	0	3	0	Nicastrin OS=Mus musculus GN=Ncstn PE=1 SV=3
P01898	0.452158509	5	5	4	0	4	0	0	4	H-2 class I histocompatibility antigen, Q10 alpha chain OS=Mus musculus GN=H2-Q10 PE=1 SV=3
P17918	0.009483303	54	39	37	16	42	29	21	33	Proliferating cell nuclear antigen OS=Mus musculus GN=Pcna PE=1 SV=2
Q8QZY1	0.468816474	69	35	35	7	34	26	21	44	Eukaryotic translation initiation factor 3 subunit L OS=Mus musculus GN=Eif3l PE=1 SV=1
P28656	0.693617281	11	5	11	0	13	4	0	6	Nucleosome assembly protein 1-like 1 OS=Mus musculus GN=Nap1l1 PE=1 SV=2
E9PW66	0.693617281	11	5	11	0	13	4	0	6	Nucleosome assembly protein 1-like 1 OS=Mus musculus GN=Nap1l1 PE=1 SV=1
P70452	0.314036203	4	0	6	0	3	5	4	5	Syntaxin-4 OS=Mus musculus GN=Stx4 PE=1 SV=1
Q91YZ8	0.098568042	11	0	0	0	15	0	6	7	Polyadenylate-binding protein OS=Mus musculus GN=Pabpc4 PE=1 SV=1
Q6PHQ9	0.098568042	11	0	0	0	15	0	6	7	Polyadenylate-binding protein OS=Mus musculus GN=Pabpc4 PE=1 SV=1
Q91V12	0.112263304	20	15	19	5	19	11	5	15	Cytosolic acyl coenzyme A thioester hydrolase OS=Mus musculus GN=Acot7 PE=1 SV=2
P01899	0.862639909	8	7	8	0	9	6	3	8	H-2 class I histocompatibility antigen, D-B alpha chain OS=Mus musculus GN=H2-D1 PE=1 SV=2
P29387	0.76273714	28	25	24	0	19	21	18	29	Guanine nucleotide-binding protein subunit beta-4 OS=Mus musculus GN=Gnb4 PE=1 SV=4
P14206	0.454186716	63	42	59	5	51	29	21	42	40S ribosomal protein SA OS=Mus musculus GN=Rpsa PE=1 SV=4
Q9Z0N2	0.398112856	41	33	33	3	29	25	15	24	Eukaryotic translation initiation factor 2 subunit 3, Y-linked OS=Mus musculus GN=Eif2s3y PE=1 SV=2
A0A140LHF7	0.416423711	47	26	31	5	24	27	11	30	Protein arginine N-methyltransferase 1 OS=Mus musculus GN=Prmt1 PE=1 SV=1
P97351	0.091348172	62	32	39	14	38	35	24	27	40S ribosomal protein S3a OS=Mus musculus GN=Rps3a PE=1 SV=3
P63094	0.042889642	30	19	25	7	20	17	10	21	Guanine nucleotide-binding protein G(s) subunit alpha isoforms short OS=Mus musculus GN=Gnas PE=1 SV=1
Q9EPU4	0.375788781	9	12	14	2	11	6	4	10	Cleavage and polyadenylation specificity factor subunit 1 OS=Mus musculus GN=Cpsf1 PE=1 SV=1
D3YWJ3	0.791748537	0	0	0	46	0	30	47	0	40S ribosomal protein S2 OS=Mus musculus GN=Rps2 PE=1 SV=1
Q61990	0.040219956	24	17	16	4	18	12	8	13	Poly(rC)-binding protein 2 OS=Mus musculus GN=Pcbp2 PE=1 SV=1
B7ZCF1	0.085531586	38	36	33	15	26	16	25	35	26S protease regulatory subunit 6A OS=Mus musculus GN=Psmc3 PE=1 SV=1
A0A140LJF4	0.441890231	46	21	31	5	21	25	12	28	Protein arginine N-methyltransferase 1 (Fragment) OS=Mus musculus GN=Prmt1 PE=1 SV=1
E9Q8K5	0.241482363	0	8	4	6	5	0	0	0	Titin OS=Mus musculus GN=Ttn PE=1 SV=1
Q8CD76	0.413569618	3	3	0	0	3	2	2	3	Kinesin light chain 1 OS=Mus musculus GN=Klc1 PE=1 SV=1
Q5UE59	0.413569618	3	3	0	0	3	2	2	3	Kinesin light chain 1 OS=Mus musculus GN=Klc1 PE=1 SV=1
E9Q7C9	0.413569618	3	3	0	0	3	2	2	3	Kinesin light chain 1 OS=Mus musculus GN=Klc1 PE=1 SV=1
Q7TNF4	0.413569618	3	3	0	0	3	2	2	3	Kinesin light chain 1 OS=Mus musculus GN=Klc1 PE=1 SV=1
Q9CVB6	0.420989411	13	10	11	2	3	11	6	10	Actin-related protein 2/3 complex subunit 2 OS=Mus musculus GN=Arpc2 PE=1 SV=3
Q9D1M0	0.467600551	6	0	6	0	5	0	5	5	Protein SEC13 homolog OS=Mus musculus GN=Sec13 PE=1 SV=3
P97370	0.646850134	4	7	7	0	6	5	0	4	Sodium/potassium-transporting ATPase subunit beta-3 OS=Mus musculus GN=Atp1b3 PE=1 SV=1
Q8VI75	0.767354152	8	6	4	0	8	3	5	4	Importin-4 OS=Mus musculus GN=Ipo4 PE=1 SV=1
Q9D1P4	0.881492308	0	3	0	0	3	0	0	2	Cysteine and histidine-rich domain-containing protein 1 OS=Mus musculus GN=Chordc1 PE=1 SV=1
Q9EQQ9	0.183131818	3	0	5	0	4	0	4	2	Protein O-GlcNAcase OS=Mus musculus GN=Mgea5 PE=1 SV=2
Q9D0I9	0.056114253	50	39	37	10	33	27	19	34	Arginine--tRNA ligase, cytoplasmic OS=Mus musculus GN=Rars PE=1 SV=2
P17182	0.02134838	172	144	148	38	127	103	72	115	Alpha-enolase OS=Mus musculus GN=Eno1 PE=1 SV=3
Q9R0E2	0.75272492	8	9	11	0	13	7	7	4	Procollagen-lysine,2-oxoglutarate 5-dioxygenase 1 OS=Mus musculus GN=Plod1 PE=1 SV=1
Q60996	0.842688019	9	12	7	0	10	9	5	7	Serine/threonine-protein phosphatase 2A 56 kDa regulatory subunit gamma isoform OS=Mus musculus GN=Ppp2r5c PE=1 SV=2
E9PYH2	0.104166772	20	15	19	5	19	10	5	15	Cytosolic acyl coenzyme A thioester hydrolase OS=Mus musculus GN=Acot7 PE=1 SV=1
P63073	0.088762929	17	9	18	9	9	10	0	14	Eukaryotic translation initiation factor 4E OS=Mus musculus GN=Eif4e PE=1 SV=1
P62192	0.063620078	32	28	26	7	22	18	13	24	26S protease regulatory subunit 4 OS=Mus musculus GN=Psmc1 PE=1 SV=1
Q8BMJ2	0.007944488	88	67	77	23	67	51	32	61	Leucine--tRNA ligase, cytoplasmic OS=Mus musculus GN=Lars PE=1 SV=2
E9PXZ2	0.777326031	4	3	3	0	3	3	2	3	Oxysterol-binding protein OS=Mus musculus GN=Osbpl9 PE=1 SV=2
A2A8Z1	0.777326031	4	3	3	0	3	3	2	3	Oxysterol-binding protein-related protein 9 OS=Mus musculus GN=Osbpl9 PE=1 SV=1
F6UHR6	0.777326031	4	3	3	0	3	3	2	3	Oxysterol-binding protein OS=Mus musculus GN=Osbpl9 PE=1 SV=2
A0A0A6YXH3	0.777326031	4	3	3	0	3	3	2	3	Oxysterol-binding protein OS=Mus musculus GN=Osbpl9 PE=1 SV=1
Q9Z127	0.645836903	10	9	11	0	5	7	14	7	Large neutral amino acids transporter small subunit 1 OS=Mus musculus GN=Slc7a5 PE=1 SV=2
P10605	0.230204159	19	14	20	2	15	10	0	9	Cathepsin B OS=Mus musculus GN=Ctsb PE=1 SV=2
O35226	0.831786566	6	5	6	0	0	5	4	5	26S proteasome non-ATPase regulatory subunit 4 OS=Mus musculus GN=Psmd4 PE=1 SV=1
Q64522	0.122345494	68	53	62	23	32	25	51	61	Histone H2A type 2-B OS=Mus musculus GN=Hist2h2ab PE=1 SV=3
P63242	0.102850594	29	23	28	9	34	13	9	17	Eukaryotic translation initiation factor 5A-1 OS=Mus musculus GN=Eif5a PE=1 SV=2
Q9JIF0	0.49415339	73	41	52	5	36	38	21	45	Protein arginine N-methyltransferase 1 OS=Mus musculus GN=Prmt1 PE=1 SV=1
A0A171KXD3	0.49415339	73	41	52	5	36	38	21	45	Protein arginine N-methyltransferase 1 OS=Mus musculus GN=Prmt1 PE=1 SV=1
Q9ES97	0.256490042	11	4	7	0	0	6	0	0	Reticulon-3 OS=Mus musculus GN=Rtn3 PE=1 SV=2
E9Q242	0.582097945	6	11	5	0	9	5	0	4	Adenylosuccinate lyase OS=Mus musculus GN=Adsl PE=1 SV=1
E9PUD2	0.273133897	14	12	16	2	13	11	4	8	Dynamin-1-like protein OS=Mus musculus GN=Dnm1l PE=1 SV=1
P16381	0.380752838	23	13	27	3	14	13	10	17	Putative ATP-dependent RNA helicase Pl10 OS=Mus musculus GN=D1Pas1 PE=1 SV=1
P17439	0.259257581	2	0	7	2	3	0	0	3	Glucosylceramidase OS=Mus musculus GN=Gba PE=1 SV=1
P62908	0.008808373	121	77	102	25	81	60	55	69	40S ribosomal protein S3 OS=Mus musculus GN=Rps3 PE=1 SV=1
A0A0A0MQA5	0.851752884	207	134	141	0	169	139	72	143	Tubulin alpha chain (Fragment) OS=Mus musculus GN=Tuba4a PE=1 SV=1
Q61235	0.012082616	8	0	0	0	12	5	5	4	Beta-2-syntrophin OS=Mus musculus GN=Sntb2 PE=1 SV=2
D6REG4	0.646239074	6	5	5	0	5	3	0	5	Glyoxylate reductase/hydroxypyruvate reductase OS=Mus musculus GN=Grhpr PE=1 SV=1
Q91Z53	0.646239074	6	5	5	0	5	3	0	5	Glyoxylate reductase/hydroxypyruvate reductase OS=Mus musculus GN=Grhpr PE=1 SV=1
P62715	0.957578001	36	28	33	0	42	23	10	30	Serine/threonine-protein phosphatase 2A catalytic subunit beta isoform OS=Mus musculus GN=Ppp2cb PE=1 SV=1
Q9Z2N8	0.09612211	14	12	16	4	9	10	0	9	Actin-like protein 6A OS=Mus musculus GN=Actl6a PE=1 SV=2
P62270	0.072013465	64	48	58	15	28	43	33	46	40S ribosomal protein S18 OS=Mus musculus GN=Rps18 PE=1 SV=3
F6YVP7	0.072013465	64	48	58	15	28	43	33	46	Protein Gm10260 OS=Mus musculus GN=Gm10260 PE=3 SV=2
P08249	0.909679466	9	8	8	0	8	6	4	9	Malate dehydrogenase, mitochondrial OS=Mus musculus GN=Mdh2 PE=1 SV=3
O88569	0.130011678	22	17	27	13	21	14	12	17	Heterogeneous nuclear ribonucleoproteins A2/B1 OS=Mus musculus GN=Hnrnpa2b1 PE=1 SV=2
O88685	0.064935212	38	37	35	16	26	16	25	35	26S protease regulatory subunit 6A OS=Mus musculus GN=Psmc3 PE=1 SV=2
P62281	0.049546624	29	21	28	5	22	18	12	15	40S ribosomal protein S11 OS=Mus musculus GN=Rps11 PE=1 SV=3
Q9Z1Q9	0.20669009	172	116	147	21	120	78	66	104	Valine--tRNA ligase OS=Mus musculus GN=Vars PE=1 SV=1
P97310	0.011443144	88	81	92	22	64	67	40	57	DNA replication licensing factor MCM2 OS=Mus musculus GN=Mcm2 PE=1 SV=3
D3YXG6	0.366941148	13	10	11	2	3	10	6	10	Actin-related protein 2/3 complex subunit 2 OS=Mus musculus GN=Arpc2 PE=1 SV=1
Q6PE01	0.040997119	20	22	24	11	17	16	10	19	U5 small nuclear ribonucleoprotein 40 kDa protein OS=Mus musculus GN=Snrnp40 PE=1 SV=1
A0A140T8V5	0.012813235	47	36	38	16	36	26	19	29	Proliferating cell nuclear antigen OS=Mus musculus GN=Pcna-ps2 PE=3 SV=1
D3Z6N3	0.021163668	25	20	23	8	17	14	15	15	DNA replication licensing factor MCM7 OS=Mus musculus GN=Mcm7 PE=1 SV=1
Q5FWX7	0.719088166	4	3	3	0	3	0	2	3	Oxysterol-binding protein OS=Mus musculus GN=Osbpl9 PE=1 SV=1
E9Q6C1	0.719088166	4	3	3	0	3	0	2	3	Oxysterol-binding protein OS=Mus musculus GN=Osbpl9 PE=1 SV=1
G3UY93	0.201673297	169	112	146	21	117	75	64	103	Valine--tRNA ligase (Fragment) OS=Mus musculus GN=Vars PE=1 SV=1
A0A087WR97	0.072716981	12	10	8	5	8	9	4	7	TAR DNA-binding protein 43 (Fragment) OS=Mus musculus GN=Tardbp PE=1 SV=1
D3Z0F5	0.130502491	5	3	4	3	2	4	0	3	COP9 signalosome complex subunit 6 OS=Mus musculus GN=Cops6 PE=1 SV=1
O88545	0.130502491	5	3	4	3	2	4	0	3	COP9 signalosome complex subunit 6 OS=Mus musculus GN=Cops6 PE=1 SV=1
Q8BYA0	0.194543718	19	17	16	3	10	14	6	14	Tubulin-specific chaperone D OS=Mus musculus GN=Tbcd PE=1 SV=1
Q8CH72	0.220588552	3	0	2	0	2	0	0	0	E3 ubiquitin-protein ligase TRIM32 OS=Mus musculus GN=Trim32 PE=1 SV=2
Q9EST4	0.23122119	3	2	0	0	2	0	0	0	Proteasome assembly chaperone 2 OS=Mus musculus GN=Psmg2 PE=1 SV=1
O88643	0.44212375	0	5	5	0	4	0	0	0	Serine/threonine-protein kinase PAK 1 OS=Mus musculus GN=Pak1 PE=1 SV=1
Q9DCN2	0.449318376	0	2	3	0	2	0	0	0	NADH-cytochrome b5 reductase 3 OS=Mus musculus GN=Cyb5r3 PE=1 SV=3
A0A0A0MQM3	0.449318376	0	2	3	0	2	0	0	0	NADH-cytochrome b5 reductase 3 (Fragment) OS=Mus musculus GN=Cyb5r3 PE=1 SV=6
Q9JHU9	0.451762183	0	3	2	0	2	0	0	0	Inositol-3-phosphate synthase 1 OS=Mus musculus GN=Isyna1 PE=1 SV=1
Q9CX56	0.92575085	6	4	0	0	0	0	3	5	26S proteasome non-ATPase regulatory subunit 8 OS=Mus musculus GN=Psmd8 PE=1 SV=2
Q9CPS5	0.92575085	6	4	0	0	0	0	3	5	26S proteasome non-ATPase regulatory subunit 8 OS=Mus musculus GN=Psmd8 PE=1 SV=2
Q9WTM5	0.181915707	48	33	40	6	29	24	20	28	RuvB-like 2 OS=Mus musculus GN=Ruvbl2 PE=1 SV=3
Q922D8	0.001070068	87	70	76	24	62	54	40	48	C-1-tetrahydrofolate synthase, cytoplasmic OS=Mus musculus GN=Mthfd1 PE=1 SV=4
Q9R118	0.079313958	46	49	48	21	42	33	27	28	Serine protease HTRA1 OS=Mus musculus GN=Htra1 PE=1 SV=2
Q8BG32	0.379279762	52	28	40	4	27	26	17	28	26S proteasome non-ATPase regulatory subunit 11 OS=Mus musculus GN=Psmd11 PE=1 SV=3
Q6P542	0.417606464	6	6	7	0	5	0	0	5	ATP-binding cassette sub-family F member 1 OS=Mus musculus GN=Abcf1 PE=1 SV=1
P00375	0.790757858	6	0	5	0	0	5	2	6	Dihydrofolate reductase OS=Mus musculus GN=Dhfr PE=1 SV=3
E9Q133	0.060584355	144	110	127	59	99	85	76	86	T-complex protein 1 subunit gamma OS=Mus musculus GN=Cct3 PE=1 SV=1
Q7TNQ1	0.821762615	7	7	7	0	5	7	5	5	Coatomer protein complex, subunit gamma OS=Mus musculus GN=Copg1 PE=1 SV=1
Q921F2	0.080535021	14	10	8	5	8	10	4	7	TAR DNA-binding protein 43 OS=Mus musculus GN=Tardbp PE=1 SV=1
Q9CWJ9	0.023065486	92	78	62	36	69	46	31	64	Bifunctional purine biosynthesis protein PURH OS=Mus musculus GN=Atic PE=1 SV=2
F6YJW4	0.836662636	9	8	6	0	5	7	6	6	60S ribosomal protein L18a OS=Mus musculus GN=Gm17541 PE=3 SV=1
A0A087WPE4	0.801737221	10	7	9	0	8	8	6	5	Protein Eloc (Fragment) OS=Mus musculus GN=Eloc PE=1 SV=1
J3QMM7	0.35318584	3	0	0	0	2	3	0	2	ATP-dependent (S)-NAD(P)H-hydrate dehydratase OS=Mus musculus GN=Naxd PE=1 SV=1
J3QN06	0.35318584	3	0	0	0	2	3	0	2	ATP-dependent (S)-NAD(P)H-hydrate dehydratase OS=Mus musculus GN=Naxd PE=1 SV=1
K3W4M4	0.35318584	3	0	0	0	2	3	0	2	ATP-dependent (S)-NAD(P)H-hydrate dehydratase OS=Mus musculus GN=Naxd PE=1 SV=1
Q9CZ42	0.35318584	3	0	0	0	2	3	0	2	ATP-dependent (S)-NAD(P)H-hydrate dehydratase OS=Mus musculus GN=Naxd PE=1 SV=1
A0A087WPH7	0.205622556	30	30	25	13	17	7	25	27	26S protease regulatory subunit 6A (Fragment) OS=Mus musculus GN=Psmc3 PE=1 SV=6
A0A1B0GRR3	0.027906461	26	17	24	4	18	13	12	12	40S ribosomal protein S11 OS=Mus musculus GN=Rps11 PE=1 SV=1
B7ZNL8	0.137122072	13	6	7	5	5	7	4	8	Protein Pus7 OS=Mus musculus GN=Pus7 PE=1 SV=1
Q91VU7	0.137122072	13	6	7	5	5	7	4	8	Protein Pus7 OS=Mus musculus GN=Pus7 PE=1 SV=2
G3UXL2	0.070775374	15	14	14	10	11	9	6	15	Protein Prps1l3 OS=Mus musculus GN=Prps1l3 PE=3 SV=1

Q9D7G0	0.070775374	15	14	14	10	11	9	6	15	Ribose-phosphate pyrophosphokinase 1 OS=Mus musculus GN=Prps1 PE=1 SV=4
A0A0A0MQM0	0.114770901	28	23	28	9	34	13	7	14	Eukaryotic translation initiation factor 5A (Fragment) OS=Mus musculus GN=Eif5a PE=1 SV=1
P97384	0.140384169	24	15	23	4	14	12	9	16	Annexin A11 OS=Mus musculus GN=Anxa11 PE=1 SV=2
P62869	0.558486439	5	6	0	0	5	3	3	6	Transcription elongation factor B polypeptide 2 OS=Mus musculus GN=Tceb2 PE=1 SV=1
P61164	0.207952856	46	25	25	5	27	16	10	25	Alpha-centractin OS=Mus musculus GN=Actr1a PE=1 SV=1
Q62095	0.355119395	21	11	23	2	11	10	10	13	ATP-dependent RNA helicase DDX3Y OS=Mus musculus GN=Ddx3y PE=1 SV=2
P39054	0.898026805	17	10	8	0	9	4	10	13	Dynamin-2 OS=Mus musculus GN=Dnm2 PE=1 SV=2
A0A087WRZ5	0.153523291	10	7	5	4	0	6	4	0	TAR DNA-binding protein 43 OS=Mus musculus GN=Tardbp PE=1 SV=1
H3BKN0	0.22684288	14	5	12	2	6	7	0	6	tRNA (cytosine(34)-C(5))-methyltransferase OS=Mus musculus GN=Nsun2 PE=1 SV=1
Q1HFZ0	0.22684288	14	5	12	2	6	7	0	6	tRNA (cytosine(34)-C(5))-methyltransferase OS=Mus musculus GN=Nsun2 PE=1 SV=2
P63330	0.935498292	37	30	36	0	42	22	10	31	Serine/threonine-protein phosphatase 2A catalytic subunit alpha isoform OS=Mus musculus GN=Ppp2ca PE=1 SV=1
A0A0N4SVB8	0.885078277	0	4	3	0	0	2	2	4	ADP-ribosylation factor-like protein 8B OS=Mus musculus GN=Arl8b PE=1 SV=1
Q9EQH3	0.000640955	50	45	46	15	31	30	23	35	Vacuolar protein sorting-associated protein 35 OS=Mus musculus GN=Vps35 PE=1 SV=1
E9PZS4	0.01823288	8	5	5	3	4	0	0	0	NHP2-like protein 1 OS=Mus musculus GN=Nhp2l1 PE=1 SV=1
Q9DBZ5	0.031550301	7	7	4	3	5	0	0	3	Eukaryotic translation initiation factor 3 subunit K OS=Mus musculus GN=Eif3k PE=1 SV=1
P60122	0.038885582	50	33	37	18	37	20	21	27	RuvB-like 1 OS=Mus musculus GN=Ruvbl1 PE=1 SV=1
Q9CZU6	0.013791653	31	23	21	8	16	15	10	22	Citrate synthase, mitochondrial OS=Mus musculus GN=Cs PE=1 SV=1
Q9DB05	0.134643719	13	5	7	4	5	6	4	7	Alpha-soluble NSF attachment protein OS=Mus musculus GN=Napa PE=1 SV=1
A2AFQ0	0.200119101	5	11	7	6	6	4	5	7	E3 ubiquitin-protein ligase HUWE1 OS=Mus musculus GN=Huwe1 PE=1 SV=1
Q7TMY8	0.200119101	5	11	7	6	6	4	5	7	E3 ubiquitin-protein ligase HUWE1 OS=Mus musculus GN=Huwe1 PE=1 SV=5
A0A0G2JFB4	0.084637619	17	9	18	9	8	10	0	12	Eukaryotic translation initiation factor 4E OS=Mus musculus GN=Eif4e PE=1 SV=1
Q9D8W5	0.143275264	28	18	23	4	15	11	12	17	26S proteasome non-ATPase regulatory subunit 12 OS=Mus musculus GN=Psmd12 PE=1 SV=4
Q9DAW6	0.000732709	32	29	28	8	23	21	14	15	U4/U6 small nuclear ribonucleoprotein Prp4 OS=Mus musculus GN=Prpf4 PE=1 SV=1
Q8CCK0	0.099766627	8	2	6	0	4	0	0	0	Core histone macro-H2A.2 OS=Mus musculus GN=H2afy2 PE=1 SV=3
Q9R0T7	0.277159512	7	5	4	0	0	0	0	4	MCG15085 OS=Mus musculus GN=Try4 PE=1 SV=1
Q9QUK9	0.277159512	7	5	4	0	0	0	0	4	MCG15083 OS=Mus musculus GN=Try5 PE=1 SV=1
Q8CIG8	0.003700727	16	15	12	5	8	11	4	13	Protein arginine N-methyltransferase 5 OS=Mus musculus GN=Prmt5 PE=1 SV=3
Q3THS6	0.066257647	13	10	13	4	13	7	3	7	S-adenosylmethionine synthase isoform type-2 OS=Mus musculus GN=Mat2a PE=1 SV=2
Q921F4	0.081456772	8	5	4	3	2	3	3	7	Heterogeneous nuclear ribonucleoprotein L-like OS=Mus musculus GN=Hnrnpll PE=1 SV=3
Q8C483	0.106711467	53	37	41	9	27	22	22	34	Putative uncharacterized protein OS=Mus musculus GN=Sars PE=1 SV=1
P26638	0.106711467	53	37	41	9	27	22	22	34	Serine--tRNA ligase, cytoplasmic OS=Mus musculus GN=Sars PE=1 SV=3
B1AT36	0.143218118	27	18	23	4	14	11	12	17	26S proteasome non-ATPase regulatory subunit 12 OS=Mus musculus GN=Psmd12 PE=1 SV=1
Q99L45	0.209538616	15	8	11	2	7	7	4	9	Eukaryotic translation initiation factor 2 subunit 2 OS=Mus musculus GN=Eif2s2 PE=1 SV=1
O88543	0.243754937	5	2	5	0	0	3	0	0	COP9 signalosome complex subunit 3 OS=Mus musculus GN=Cops3 PE=1 SV=3
Q99KI0	0.345793581	4	4	4	0	0	4	0	2	Aconitate hydratase, mitochondrial OS=Mus musculus GN=Aco2 PE=1 SV=1
Q8K4Z3	0.351719862	4	4	0	0	4	2	0	0	NAD(P)H-hydrate epimerase OS=Mus musculus GN=Naxe PE=1 SV=1
P97470	0.57210396	6	10	8	0	5	6	0	7	Serine/threonine-protein phosphatase 4 catalytic subunit OS=Mus musculus GN=Ppp4c PE=1 SV=2
Q9CQT1	0.698170742	5	4	3	0	0	5	2	2	Methylthioribose-1-phosphate isomerase OS=Mus musculus GN=Mri1 PE=1 SV=1
Q9Z2X2	0.739239157	0	4	0	0	3	0	0	0	26S proteasome non-ATPase regulatory subunit 10 OS=Mus musculus GN=Psmd10 PE=1 SV=3
E0CX62	0.787223017	0	4	0	0	0	0	0	3	Proteasome endopeptidase complex OS=Mus musculus GN=Psma3 PE=1 SV=2
Q99020	0.873623098	5	11	8	0	8	6	2	8	Heterogeneous nuclear ribonucleoprotein A/B OS=Mus musculus GN=Hnrnpab PE=1 SV=1
Q80XR6	0.873623098	5	11	8	0	8	6	2	8	Heterogeneous nuclear ribonucleoprotein A/B OS=Mus musculus GN=Hnrnpab PE=1 SV=1
Q20BD0	0.873623098	5	11	8	0	8	6	2	8	Heterogeneous nuclear ribonucleoprotein A/B OS=Mus musculus GN=Hnrnpab PE=1 SV=1
Q3TG45	0.897839464	4	4	0	0	0	0	3	3	26S proteasome non-ATPase regulatory subunit 8 OS=Mus musculus GN=Psmd8 PE=1 SV=1
Q3U1J4	0.942348784	10	6	11	0	8	4	7	8	DNA damage-binding protein 1 OS=Mus musculus GN=Ddb1 PE=1 SV=2
Q64487	0.966724591	6	2	0	0	0	3	3	0	Receptor-type tyrosine-protein phosphatase delta OS=Mus musculus GN=Ptprd PE=1 SV=3
F8WIV5	0.947821167	17	10	8	0	9	4	9	13	Dynamin-2 OS=Mus musculus GN=Dnm2 PE=1 SV=2
P21995	0.982212557	10	6	10	0	8	6	5	7	Embigin OS=Mus musculus GN=Emb PE=1 SV=2
Q91YH6	0.998157076	8	4	11	0	7	5	4	7	ATPase, H+ transporting, lysosomal V1 subunit B1 OS=Mus musculus GN=Atp6v1b1 PE=1 SV=1
E9Q1H7	0.852526194	10	8	2	0	0	3	6	6	tRNA (guanine-N(7)-)-methyltransferase non-catalytic subunit WDR4 OS=Mus musculus GN=Wdr4 PE=1 SV=1
P07607	0.196389933	2	2	4	0	2	0	0	0	Thymidylate synthase OS=Mus musculus GN=Tyms PE=1 SV=1
Q8BJU2	0.382762986	3	3	2	0	2	0	0	2	Tetraspanin-9 OS=Mus musculus GN=Tspan9 PE=1 SV=1
D3YXN7	0.382762986	3	3	2	0	2	0	0	2	Tetraspanin (Fragment) OS=Mus musculus GN=Tspan9 PE=1 SV=1
A0A0J9YTS4	0.382762986	3	3	2	0	2	0	0	2	Tetraspanin (Fragment) OS=Mus musculus GN=Tspan9 PE=1 SV=1
Q9CXW4	0.034279527	39	30	43	17	30	23	16	27	60S ribosomal protein L11 OS=Mus musculus GN=Rpl11 PE=1 SV=4
E9PYL9	0.034279527	39	30	43	17	30	23	16	27	Protein Gm10036 OS=Mus musculus GN=Gm10036 PE=3 SV=1
Q9DAY9	0.35536498	0	34	35	13	27	0	15	19	Nucleophosmin OS=Mus musculus GN=Npm1 PE=1 SV=1
P49718	0.114499001	25	26	17	6	14	14	14	13	DNA replication licensing factor MCM5 OS=Mus musculus GN=Mcm5 PE=1 SV=1
Q3TCR7	0.62264294	17	10	8	0	9	4	0	13	Dynamin-2 OS=Mus musculus GN=Dnm2 PE=1 SV=1
Q3T9X3	0.62264294	17	10	8	0	9	4	0	13	Dynamin-2 OS=Mus musculus GN=Dnm2 PE=1 SV=1
G3X9G4	0.62264294	17	10	8	0	9	4	0	13	Dynamin-2 OS=Mus musculus GN=Dnm2 PE=1 SV=1
Q02257	0.078619012	9	7	12	3	7	5	3	8	Junction plakoglobin OS=Mus musculus GN=Jup PE=1 SV=3
P70288	0.341645221	17	12	19	2	10	8	5	14	Histone deacetylase 2 OS=Mus musculus GN=Hdac2 PE=1 SV=1
Q5EG47	0.169545619	12	14	14	6	16	6	7	5	5'-AMP-activated protein kinase catalytic subunit alpha-1 OS=Mus musculus GN=Prkaa1 PE=1 SV=2
Q8BJY1	0.59541783	11	4	4	0	5	4	0	5	26S proteasome non-ATPase regulatory subunit 5 OS=Mus musculus GN=Psmd5 PE=1 SV=4
P17809	0.89259605	12	7	0	0	6	9	0	6	Solute carrier family 2, facilitated glucose transporter member 1 OS=Mus musculus GN=Slc2a1 PE=1 SV=4
P42932	0.001294	192	152	151	45	114	107	74	102	T-complex protein 1 subunit theta OS=Mus musculus GN=Cct8 PE=1 SV=3
Q52KC3	0.114179577	26	29	18	6	15	15	14	14	DNA helicase OS=Mus musculus GN=Mcm5 PE=1 SV=1
Q8R349	0.669501794	5	4	6	0	0	4	4	3	Cell division cycle protein 16 homolog OS=Mus musculus GN=Cdc16 PE=1 SV=1
Q8HWB2	0.185087462	16	12	15	2	11	7	5	10	Protein H2-Q4 OS=Mus musculus GN=H2-Q4 PE=1 SV=1
Q61151	0.027080591	12	7	8	4	8	0	4	5	Serine/threonine-protein phosphatase 2A 56 kDa regulatory subunit epsilon isoform OS=Mus musculus GN=Ppp2r5e PE=1 SV=3
A2ALV7	0.947813206	18	8	12	0	12	5	5	15	Protein AI314180 (Fragment) OS=Mus musculus GN=AI314180 PE=1 SV=1
Q61398	0.052621694	90	47	44	19	43	34	28	41	Procollagen C-endopeptidase enhancer 1 OS=Mus musculus GN=Pcolce PE=1 SV=2
G3XA10	0.017881492	45	36	52	14	37	23	19	28	Heterogeneous nuclear ribonucleoprotein U OS=Mus musculus GN=Hnrnpu PE=1 SV=1
Q8VEK3	0.017881492	45	36	52	14	37	23	19	28	Heterogeneous nuclear ribonucleoprotein U OS=Mus musculus GN=Hnrnpu PE=1 SV=1
Q9CQW2	0.707414558	4	4	3	0	0	2	2	4	ADP-ribosylation factor-like protein 8B OS=Mus musculus GN=Arl8b PE=1 SV=1
A0A0J9YV56	0.72314348	4	5	2	0	0	2	2	4	BRCA1-A complex subunit BRE (Fragment) OS=Mus musculus GN=Bre PE=1 SV=1
Q3TPM3	0.72314348	4	5	2	0	0	2	2	4	Cullin-1 (Fragment) OS=Mus musculus GN=Cul1 PE=1 SV=1
Q9WTX6	0.72314348	4	5	2	0	0	2	2	4	Cullin-1 OS=Mus musculus GN=Cul1 PE=1 SV=1
Q91VH2	0.74160568	0	2	9	0	6	3	2	5	Sorting nexin-9 OS=Mus musculus GN=Snx9 PE=1 SV=1
P83940	0.97713204	13	9	9	0	8	10	6	6	Transcription elongation factor B polypeptide 1 OS=Mus musculus GN=Tceb1 PE=1 SV=1
A0A087WNT1	0.97713204	13	9	9	0	8	10	6	6	Protein Eloc OS=Mus musculus GN=Eloc PE=1 SV=1
A0A087WQE6	0.97713204	13	9	9	0	8	10	6	6	Protein Eloc (Fragment) OS=Mus musculus GN=Eloc PE=1 SV=1
Q9D0T1	0.002438475	15	9	13	3	10	7	6	6	NHP2-like protein 1 OS=Mus musculus GN=Snu13 PE=1 SV=4
Q8VHK9	0.136730469	5	7	14	3	7	3	5	6	ATP-dependent RNA helicase DHX36 OS=Mus musculus GN=Dhx36 PE=1 SV=2
Q9R1Q7	0.259074891	11	9	9	0	0	0	0	7	Proteolipid protein 2 OS=Mus musculus GN=Plp2 PE=1 SV=1
G3UZZ3	0.592977537	14	8	7	0	7	4	0	10	Dynamin-2 (Fragment) OS=Mus musculus GN=Dnm2 PE=1 SV=1
Q8BJ71	0.940822932	10	8	11	0	8	6	6	8	Nuclear pore complex protein Nup93 OS=Mus musculus GN=Nup93 PE=1 SV=1
A0A1D5RLQ0	0.940822932	10	8	11	0	8	6	6	8	Nuclear pore complex protein Nup93 OS=Mus musculus GN=Nup93 PE=1 SV=1
P08207	0.945595908	109	98	79	0	39	28	41	167	Protein S100-A10 OS=Mus musculus GN=S100a10 PE=1 SV=2
F6Q2E3	0.983855042	28	25	22	0	15	6	25	26	26S protease regulatory subunit 6A (Fragment) OS=Mus musculus GN=Psmc3 PE=1 SV=1
Q8BTZ7	0.884902023	5	8	11	0	6	5	4	8	Mannose-1-phosphate guanyltransferase beta OS=Mus musculus GN=Gmppb PE=1 SV=1
P28658	0.946843524	20	10	15	0	13	12	8	10	Ataxin-10 OS=Mus musculus GN=Atxn10 PE=1 SV=2
Q9DBB4	0.826007903	8	7	7	0	8	5	3	5	N-alpha-acetyltransferase 16, NatA auxiliary subunit OS=Mus musculus GN=Naa16 PE=2 SV=1
P21550	0.207002607	27	25	26	24	31	17	7	18	Beta-enolase OS=Mus musculus GN=Eno3 PE=1 SV=3
G5E839	0.012840613	98	75	93	22	71	47	34	54	T-complex protein 1 subunit delta OS=Mus musculus GN=Cct4 PE=1 SV=1
P62960	0.030311055	40	29	27	9	21	15	16	23	Nuclease-sensitive element-binding protein 1 OS=Mus musculus GN=Ybx1 PE=1 SV=3
Reverse_tr|E9Q8N1|E9Q8N1_MOUSE	0.635216243	0	5	2	0	0	2	0	3	Titin OS=Mus musculus GN=Ttn PE=1 SV=1
P35282	0.644119534	0	5	9	0	5	5	0	0	Ras-related protein Rab-21 OS=Mus musculus GN=Rab21 PE=1 SV=4
Q6PDI5	0.910617154	18	8	13	0	12	5	5	15	Proteasome-associated protein ECM29 homolog OS=Mus musculus GN=Ecm29 PE=1 SV=3
P80315	0.010444303	105	83	101	25	80	50	37	56	T-complex protein 1 subunit delta OS=Mus musculus GN=Cct4 PE=1 SV=3
P80317	0.012996573	120	100	107	23	79	59	51	59	T-complex protein 1 subunit zeta OS=Mus musculus GN=Cct6a PE=1 SV=3
Q9WV32	0.053215098	42	33	38	7	34	19	12	20	Actin-related protein 2/3 complex subunit 1B OS=Mus musculus GN=Arpc1b PE=1 SV=4
Q91Z25	0.05840991	42	33	38	7	35	18	12	20	Actin-related protein 2/3 complex subunit 1B OS=Mus musculus GN=Arpc1b PE=1 SV=1
D3Z6E4	0.182634166	23	0	11	0	12	0	0	0	Gamma-enolase OS=Mus musculus GN=Eno2 PE=1 SV=1
Q99JY9	0.060894295	59	41	41	9	31	24	20	30	Actin-related protein 3 OS=Mus musculus GN=Actr3 PE=1 SV=3
H3BLE7	0.086723186	4	8	6	2	4	0	0	3	Serine/threonine-protein phosphatase 2A 65 kDa regulatory subunit A beta isoform OS=Mus musculus GN=Ppp2r1b PE=1 SV=1
P61358	0.01790292	23	13	16	4	18	6	5	10	60S ribosomal protein L27 OS=Mus musculus GN=Rpl27 PE=1 SV=2
F6XP90	0.152871366	5	9	4	5	4	0	3	5	E3 ubiquitin-protein ligase HUWE1 (Fragment) OS=Mus musculus GN=Huwe1 PE=1 SV=1
Q6PGB6	0.25203238	0	4	6	3	2	4	0	0	N-alpha-acetyltransferase 50 OS=Mus musculus GN=Naa50 PE=1 SV=1
Q921J2	0.389668572	4	5	4	0	0	2	0	4	GTP-binding protein Rheb OS=Mus musculus GN=Rheb PE=1 SV=1
P80313	0.02598507	92	78	77	18	63	38	32	48	T-complex protein 1 subunit eta OS=Mus musculus GN=Cct7 PE=1 SV=1
P51410	0.016286615	14	10	11	6	10	3	0	8	60S ribosomal protein L9 OS=Mus musculus GN=Rpl9 PE=2 SV=2
A0A0G2JES3	0.016286615	14	10	11	6	10	3	0	8	60S ribosomal protein L9 (Fragment) OS=Mus musculus GN=Rpl9 PE=1 SV=1
Q8K2V6	0.285818814	10	3	9	0	0	0	0	5	Importin-11 OS=Mus musculus GN=Ipo11 PE=1 SV=1
P62315	0.498399877	8	7	7	0	6	3	0	6	Small nuclear ribonucleoprotein Sm D1 OS=Mus musculus GN=Snrpd1 PE=1 SV=1
Q05D44	0.033250521	7	9	4	2	2	6	2	5	Eukaryotic translation initiation factor 5B OS=Mus musculus GN=Eif5b PE=1 SV=2
Q9D819	0.072585838	10	16	15	6	11	10	2	9	Inorganic pyrophosphatase OS=Mus musculus GN=Ppa1 PE=1 SV=1
P14131	0.009856113	45	30	37	16	27	21	14	25	40S ribosomal protein S16 OS=Mus musculus GN=Rps16 PE=1 SV=4
P28474	0.518986953	20	15	15	0	11	8	0	15	Alcohol dehydrogenase class-3 OS=Mus musculus GN=Adh5 PE=1 SV=3
G3X8Y3	0.043002755	72	61	65	31	43	33	39	40	N-alpha-acetyltransferase 15, NatA auxiliary subunit OS=Mus musculus GN=Naa15 PE=1 SV=1
P32921	0.251597325	48	33	30	3	29	12	14	22	Tryptophan--tRNA ligase, cytoplasmic OS=Mus musculus GN=Wars PE=1 SV=2
Q921M3	0.032053884	16	11	21	4	9	7	8	11	Splicing factor 3B subunit 3 OS=Mus musculus GN=Sf3b3 PE=1 SV=1
Q3U741	0.010510347	39	25	31	12	25	18	12	17	DEAD (Asp-Glu-Ala-Asp) box polypeptide 17, isoform CRA_a OS=Mus musculus GN=Ddx17 PE=1 SV=1
Q5XJF6	0.20563522	37	33	36	3	29	18	8	18	Ribosomal protein OS=Mus musculus GN=Rpl10a PE=1 SV=1
A2BH06	0.027780629	33	28	38	16	24	15	16	22	60S ribosomal protein L11 (Fragment) OS=Mus musculus GN=Rpl11 PE=1 SV=1
P84104	0.013529691	9	5	5	3	5	0	2	4	Serine/arginine-rich splicing factor 3 OS=Mus musculus GN=Srsf3 PE=1 SV=1
Q63932	0.903452897	7	4	0	0	0	4	3	4	Dual specificity mitogen-activated protein kinase kinase 2 OS=Mus musculus GN=Map2k2 PE=1 SV=2
Q91YS7	0.903452897	7	4	0	0	0	4	3	4	Dual-specificity mitogen-activated protein kinase kinase 2 OS=Mus musculus GN=Map2k2 PE=1 SV=1
E9PX23	0.000280604	8	4	4	2	3	0	0	3	Metastasis-associated protein MTA1 OS=Mus musculus GN=Mta1 PE=1 SV=1
Q8K4B0	0.000280604	8	4	4	2	3	0	0	3	Metastasis-associated protein MTA1 OS=Mus musculus GN=Mta1 PE=1 SV=1
F8WHY8	0.000280604	8	4	4	2	3	0	0	3	Metastasis-associated protein MTA1 OS=Mus musculus GN=Mta1 PE=1 SV=1
Q501J6	0.010174511	39	25	32	12	25	18	12	17	Probable ATP-dependent RNA helicase DDX17 OS=Mus musculus GN=Ddx17 PE=1 SV=1
P62274	0.036060011	6	9	6	3	4	0	0	0	40S ribosomal protein S29 OS=Mus musculus GN=Rps29 PE=3 SV=2
Q7TNC4	0.048376989	3	3	6	3	2	0	0	3	Putative RNA-binding protein Luc7-like 2 OS=Mus musculus GN=Luc7l2 PE=1 SV=1
E9QAK5	0.07367932	13	19	18	7	14	11	6	7	Kinesin-like protein (Fragment) OS=Mus musculus GN=Kif5b PE=1 SV=1
Q8R1Q8	0.079452368	13	8	7	2	0	3	0	7	Cytoplasmic dynein 1 light intermediate chain 1 OS=Mus musculus GN=Dync1li1 PE=1 SV=1
D3Z5I1	0.109558887	3	3	3	0	2	0	0	0	Zinc finger CCCH-type antiviral protein 1 OS=Mus musculus GN=Zc3hav1 PE=1 SV=1
Q3UPF5	0.109558887	3	3	3	0	2	0	0	0	Zinc finger CCCH-type antiviral protein 1 OS=Mus musculus GN=Zc3hav1 PE=1 SV=1
P55264	0.118026538	25	22	25	3	18	8	13	11	Adenosine kinase OS=Mus musculus GN=Adk PE=1 SV=2
P23198	0.139412118	5	6	7	0	4	0	0	0	Chromobox protein homolog 3 OS=Mus musculus GN=Cbx3 PE=1 SV=2
Q9DCC5	0.139412118	5	6	7	0	4	0	0	0	Cbx3 protein OS=Mus musculus GN=Cbx3 PE=1 SV=1
O08528	0.182717746	4	0	2	0	2	0	0	0	Hexokinase-2 OS=Mus musculus GN=Hk2 PE=1 SV=1
Q9ESU7	0.214838634	7	7	0	4	0	0	3	5	Amino acid transporter OS=Mus musculus GN=Slc1a5 PE=1 SV=1
A2ASS6	0.219850567	0	8	4	6	4	0	0	0	Titin OS=Mus musculus GN=Ttn PE=1 SV=1
P61514	0.223753639	0	3	3	0	0	2	0	0	60S ribosomal protein L37a OS=Mus musculus GN=Rpl37a PE=1 SV=2
P14873	0.258084869	0	0	3	0	2	2	2	0	Microtubule-associated protein 1B OS=Mus musculus GN=Map1b PE=1 SV=2
Q05CX5	0.268866728	3	3	0	0	2	0	0	0	Luc7l2 protein OS=Mus musculus GN=Luc7l2 PE=1 SV=1
O54825	0.475628384	2	4	0	0	0	0	0	2	Bystin OS=Mus musculus GN=Bysl PE=1 SV=3
Q8CCF0	0.526796547	4	0	2	0	0	2	0	0	U4/U6 small nuclear ribonucleoprotein Prp31 OS=Mus musculus GN=Prpf31 PE=1 SV=3
Reverse_tr|D3YUR7|D3YUR7_MOUSE	0.566966263	0	0	0	3	0	0	2	0	Lipoxygenase homology domain-containing protein 1 (Fragment) OS=Mus musculus GN=Loxhd1 PE=4 SV=1
G3X9X5	0.699888565	0	3	0	0	2	0	0	0	Zinc finger CCCH type, antiviral 1, isoform CRA_b OS=Mus musculus GN=Zc3hav1 PE=1 SV=1
G3X914	0.736399823	5	0	4	0	4	2	3	0	Cullin 5 OS=Mus musculus GN=Cul5 PE=1 SV=1
Q9D5V5	0.736399823	5	0	4	0	4	2	3	0	Cullin-5 OS=Mus musculus GN=Cul5 PE=1 SV=3
E9PV12	0.736399823	5	0	4	0	4	2	3	0	Cullin-5 OS=Mus musculus GN=Cul5 PE=1 SV=1
Q9WVE8	0.772026465	9	10	8	0	9	3	4	8	Protein kinase C and casein kinase substrate in neurons protein 2 OS=Mus musculus GN=Pacsin2 PE=1 SV=1
P16675	0.787283431	0	0	3	0	0	0	0	2	Lysosomal protective protein OS=Mus musculus GN=Ctsa PE=1 SV=1
G3X8T3	0.787283431	0	0	3	0	0	0	0	2	Carboxypeptidase OS=Mus musculus GN=Ctsa PE=1 SV=1
A0A0R4J093	0.787283431	0	0	3	0	0	0	0	2	UMP-CMP kinase OS=Mus musculus GN=Cmpk1 PE=1 SV=1
Q9DBP5	0.787283431	0	0	3	0	0	0	0	2	UMP-CMP kinase OS=Mus musculus GN=Cmpk1 PE=1 SV=1
Q9Z1D1	0.897810134	3	0	0	0	0	2	0	0	Eukaryotic translation initiation factor 3 subunit G OS=Mus musculus GN=Eif3g PE=1 SV=2
A0A1B0GQZ9	0.930745978	0	0	3	0	2	0	0	2	U6 snRNA-associated Sm-like protein LSm4 OS=Mus musculus GN=Lsm4 PE=1 SV=1
Q9CY46	0.930745978	0	0	3	0	2	0	0	2	LSM4 homolog, U6 small nuclear RNA associated (S. cerevisiae), isoform CRA_b OS=Mus musculus GN=Lsm4 PE=1 SV=1
D3YTP8	0.930745978	0	0	3	0	2	0	0	2	U6 snRNA-associated Sm-like protein LSm4 (Fragment) OS=Mus musculus GN=Lsm4 PE=1 SV=8
Q9QXA5	0.930745978	0	0	3	0	2	0	0	2	U6 snRNA-associated Sm-like protein LSm4 OS=Mus musculus GN=Lsm4 PE=1 SV=1

A0A1B0GRR9	0.930745978	0	0	3	0	2	0	0	2	U6 snRNA-associated Sm-like protein LSm4 OS=Mus musculus GN=Lsm4 PE=1 SV=1
B1AXN9	0.997662484	5	0	4	0	3	4	0	2	Ribosomal protein S6 kinase alpha-3 OS=Mus musculus GN=Rps6ka3 PE=1 SV=1
P18654	0.997662484	5	0	4	0	3	4	0	2	Ribosomal protein S6 kinase alpha-3 OS=Mus musculus GN=Rps6ka3 PE=1 SV=2
A0A0U1RPX4	0.01270274	27	17	18	6	14	12	0	8	Mitogen-activated protein kinase (Fragment) OS=Mus musculus GN=Mapk3 PE=1 SV=1
E0CYT5	0.169102411	9	18	10	7	0	5	11	6	Cullin-2 (Fragment) OS=Mus musculus GN=Cul2 PE=1 SV=1
Q8K1R7	0.922372567	4	3	0	0	2	3	0	2	Serine/threonine-protein kinase Nek9 OS=Mus musculus GN=Nek9 PE=1 SV=2
Q80UM3	0.05501893	69	61	61	31	41	31	38	37	N-alpha-acetyltransferase 15, NatA auxiliary subunit OS=Mus musculus GN=Naa15 PE=1 SV=1
A0A0U1RPZ0	0.121870507	18	10	13	6	8	9	9	5	Mitogen-activated protein kinase (Fragment) OS=Mus musculus GN=Mapk3 PE=1 SV=1
P26516	0.001020643	25	24	23	10	13	12	9	20	26S proteasome non-ATPase regulatory subunit 7 OS=Mus musculus GN=Psmd7 PE=1 SV=2
O35593	0.016754962	10	10	16	5	6	6	8	7	26S proteasome non-ATPase regulatory subunit 14 OS=Mus musculus GN=Psmd14 PE=1 SV=2
Q6P5F9	0.253914747	21	20	24	2	14	14	3	13	Exportin-1 OS=Mus musculus GN=Xpo1 PE=1 SV=1
P46460	0.340175461	5	7	4	0	4	0	0	3	Vesicle-fusing ATPase OS=Mus musculus GN=Nsf PE=1 SV=2
Q8BML9	0.181011248	22	12	27	3	17	8	6	11	Glutaminyl-tRNA synthetase OS=Mus musculus GN=Qars PE=1 SV=1
D3Z158	0.181011248	22	12	27	3	17	8	6	11	Protein Qars OS=Mus musculus GN=Qars PE=1 SV=2
P47964	0.409730496	6	0	7	0	3	4	6	4	60S ribosomal protein L36 OS=Mus musculus GN=Rpl36 PE=3 SV=2
P11983	0.000993688	158	128	147	48	99	79	55	81	T-complex protein 1 subunit alpha OS=Mus musculus GN=Tcp1 PE=1 SV=3
A0A0U1RNT6	0.598321889	13	10	0	0	13	7	3	7	S-adenosylmethionine synthase OS=Mus musculus GN=Mat2a PE=1 SV=1
Q9CQR2	0.228410662	5	0	0	0	3	2	3	5	40S ribosomal protein S21 OS=Mus musculus GN=Rps21 PE=1 SV=1
P60229	0.815446123	21	10	14	0	10	8	7	14	Eukaryotic translation initiation factor 3 subunit E OS=Mus musculus GN=Eif3e PE=1 SV=1
P63276	0.493146713	12	6	16	0	12	5	0	5	40S ribosomal protein S17 OS=Mus musculus GN=Rps17 PE=1 SV=2
Q8VEE4	0.010818426	13	15	14	6	3	8	8	12	Replication protein A 70 kDa DNA-binding subunit OS=Mus musculus GN=Rpa1 PE=1 SV=1
Q63844	0.009816458	28	17	19	6	15	12	10	8	Mitogen-activated protein kinase 3 OS=Mus musculus GN=Mapk3 PE=1 SV=5
Q6P2B1	0.025639357	9	9	6	4	6	0	0	3	Transportin-3 OS=Mus musculus GN=Tnpo3 PE=1 SV=1
F6SVV1	0.027828502	12	8	5	3	6	4	3	5	Protein Gm9493 OS=Mus musculus GN=Gm9493 PE=4 SV=1
Q61768	0.036797198	18	21	24	7	16	13	6	10	Kinesin-1 heavy chain OS=Mus musculus GN=Kif5b PE=1 SV=3
A2A4X6	0.302865824	6	3	5	0	3	0	0	3	MCG21910 OS=Mus musculus GN=Gm12355 PE=4 SV=1
Q8K3W0	0.617970488	5	6	3	0	0	2	2	5	BRCA1-A complex subunit BRE OS=Mus musculus GN=Bre PE=1 SV=2
Q8VDM4	0.003074222	89	59	70	25	38	39	30	49	26S proteasome non-ATPase regulatory subunit 2 OS=Mus musculus GN=Psmd2 PE=1 SV=1
Q3ULG5	0.031859845	55	41	42	10	26	23	16	30	DNA helicase OS=Mus musculus GN=Mcm6 PE=1 SV=1
P97311	0.031859845	55	41	42	10	26	23	16	30	DNA replication licensing factor MCM6 OS=Mus musculus GN=Mcm6 PE=1 SV=1
P63168	0.189808018	20	15	33	4	11	11	8	16	Dynein light chain 1, cytoplasmic OS=Mus musculus GN=Dynll1 PE=1 SV=1
Q99JI4	0.020299795	45	35	36	11	14	23	19	25	26S proteasome non-ATPase regulatory subunit 6 OS=Mus musculus GN=Psmd6 PE=1 SV=1
Q9D8N0	0.085866226	133	98	95	18	72	42	36	69	Elongation factor 1-gamma OS=Mus musculus GN=Eef1g PE=1 SV=3
Q8CIN4	0.565758381	0	5	6	0	5	2	0	0	Serine/threonine-protein kinase PAK 2 OS=Mus musculus GN=Pak2 PE=1 SV=1
P62334	0.068765688	46	43	40	8	23	19	18	27	26S protease regulatory subunit 10B OS=Mus musculus GN=Psmc6 PE=1 SV=1
Q3TUE1	0.459658252	5	3	3	0	3	0	2	2	Far upstream element-binding protein 1 OS=Mus musculus GN=Fubp1 PE=1 SV=1
Q3UUU2	0.459658252	5	3	3	0	3	0	2	2	Far upstream element-binding protein 1 OS=Mus musculus GN=Fubp1 PE=1 SV=1
A0A0G2JFY5	0.459658252	5	3	3	0	3	0	2	2	Far upstream element-binding protein 1 OS=Mus musculus GN=Fubp1 PE=1 SV=1
Q91WJ8	0.459658252	5	3	3	0	3	0	2	2	Far upstream element-binding protein 1 OS=Mus musculus GN=Fubp1 PE=1 SV=1
A0A0G2JGW9	0.459658252	5	3	3	0	3	0	2	2	Far upstream element-binding protein 1 OS=Mus musculus GN=Fubp1 PE=1 SV=1
Q9D0B6	0.096811362	9	7	3	2	0	5	2	3	Protein PBDC1 OS=Mus musculus GN=Pbdc1 PE=1 SV=1
B2RQS1	0.079117084	10	8	8	4	3	8	4	4	Striatin-3 OS=Mus musculus GN=Strn3 PE=1 SV=1
Q9ERG2	0.079117084	10	8	8	4	3	8	4	4	Striatin-3 OS=Mus musculus GN=Strn3 PE=1 SV=1
Q5SWU9	0.059991557	29	16	28	6	10	14	10	16	Acetyl-CoA carboxylase 1 OS=Mus musculus GN=Acaca PE=1 SV=1
P28271	0.535771739	7	4	8	0	4	2	0	6	Cytoplasmic aconitate hydratase OS=Mus musculus GN=Aco1 PE=1 SV=3
Q68FD5	0.004190833	416	294	302	95	189	175	130	204	Clathrin heavy chain 1 OS=Mus musculus GN=Cltc PE=1 SV=3
Q9Z1N5	0.00958943	34	28	33	13	24	14	11	19	Spliceosome RNA helicase Ddx39b OS=Mus musculus GN=Ddx39b PE=1 SV=1
D3Z3G6	0.014155292	28	17	19	6	13	12	0	8	Mitogen-activated protein kinase OS=Mus musculus GN=Mapk3 PE=1 SV=1
Q99K48	0.099055955	25	19	19	4	7	11	10	14	Non-POU domain-containing octamer-binding protein OS=Mus musculus GN=Nono PE=1 SV=3
Q5SWN2	0.011419175	13	15	14	6	3	8	8	11	Replication protein A 70 kDa DNA-binding subunit OS=Mus musculus GN=Rpa1 PE=1 SV=2
D3Z1S1	0.173907262	9	16	8	7	9	4	6	6	Septin-2 (Fragment) OS=Mus musculus GN=Sept2 PE=1 SV=1
A0A0G2JF23	0.20073407	6	0	0	0	4	3	4	4	Malate dehydrogenase (Fragment) OS=Mus musculus GN=Mdh2 PE=1 SV=1
Q8K1M6	0.598459988	14	12	16	0	13	11	4	7	Dynamin-1-like protein OS=Mus musculus GN=Dnm1l PE=1 SV=2
G3UYV7	0.972187024	0	0	4	0	3	0	0	2	40S ribosomal protein S28 (Fragment) OS=Mus musculus GN=Rps28 PE=1 SV=1
P62858	0.972187024	0	0	4	0	3	0	0	2	40S ribosomal protein S28 OS=Mus musculus GN=Rps28 PE=1 SV=1
P62849	0.985294829	9	0	6	0	6	0	4	4	40S ribosomal protein S24 OS=Mus musculus GN=Rps24 PE=1 SV=1
Q6P4T2	0.044322569	60	60	69	12	46	29	21	29	U5 small nuclear ribonucleoprotein 200 kDa helicase OS=Mus musculus GN=Snrnp200 PE=1 SV=1
H3BKH6	0.099216098	52	44	41	7	21	22	18	28	S-formylglutathione hydrolase OS=Mus musculus GN=Esd PE=1 SV=1
Q9R0P3	0.099216098	52	44	41	7	21	22	18	28	S-formylglutathione hydrolase OS=Mus musculus GN=Esd PE=1 SV=1
A0A1D5RMD8	0.000586401	9	7	7	3	0	0	0	4	CCR4-NOT transcription complex subunit 1 OS=Mus musculus GN=Cnot1 PE=1 SV=1
Q99LC2	0.038367273	4	3	4	2	0	0	2	0	Cleavage stimulation factor subunit 1 OS=Mus musculus GN=Cstf1 PE=1 SV=1
E9Q715	0.043193753	3	3	5	2	2	0	0	0	Putative RNA-binding protein Luc7-like 2 OS=Mus musculus GN=Luc7l2 PE=1 SV=1
P63085	0.082277381	33	19	20	6	9	16	12	11	Mitogen-activated protein kinase 1 OS=Mus musculus GN=Mapk1 PE=1 SV=3
O70194	0.032741316	22	16	17	2	15	8	7	5	Eukaryotic translation initiation factor 3 subunit D OS=Mus musculus GN=Eif3d PE=1 SV=2
H3BJL6	0.090321319	52	44	41	7	21	22	18	27	S-formylglutathione hydrolase OS=Mus musculus GN=Esd PE=1 SV=1
P52293	0.008257337	15	7	15	4	8	4	5	8	Importin subunit alpha-1 OS=Mus musculus GN=Kpna2 PE=1 SV=2
Q03145	0.07289422	18	8	16	4	0	7	7	7	Ephrin type-A receptor 2 OS=Mus musculus GN=Epha2 PE=1 SV=3
P54775	0.017087401	60	78	77	40	27	27	35	66	26S protease regulatory subunit 6B OS=Mus musculus GN=Psmc4 PE=1 SV=2
Q497W9	0.170890156	81	49	53	5	38	28	16	32	DEAH (Asp-Glu-Ala-His) box polypeptide 15 OS=Mus musculus GN=Dhx15 PE=1 SV=1
Q8JZZ5	0.89389702	6	0	5	0	4	3	0	3	Phosphatidylinositol transfer protein beta isoform OS=Mus musculus GN=Pitpnb PE=1 SV=1
P53811	0.89389702	6	0	5	0	4	3	0	3	Phosphatidylinositol transfer protein beta isoform OS=Mus musculus GN=Pitpnb PE=1 SV=2
Q8JZQ9	0.095685737	62	49	50	8	21	30	19	32	Eukaryotic translation initiation factor 3 subunit B OS=Mus musculus GN=Eif3b PE=1 SV=1
Q9ERF3	0.164683844	4	2	4	0	0	2	0	0	WD repeat-containing protein 61 OS=Mus musculus GN=Wdr61 PE=1 SV=1
D6RJ72	0.164683844	4	2	4	0	0	2	0	0	WD repeat-containing protein 61 (Fragment) OS=Mus musculus GN=Wdr61 PE=1 SV=1
Q8BVQ0	0.164683844	4	2	4	0	0	2	0	0	Putative uncharacterized protein OS=Mus musculus GN=Wdr61 PE=1 SV=1
D6RDC7	0.164683844	4	2	4	0	0	2	0	0	Putative uncharacterized protein OS=Mus musculus GN=Wdr61 PE=1 SV=2
Q6WKZ8	0.240188457	5	2	3	0	0	0	0	2	E3 ubiquitin-protein ligase UBR2 OS=Mus musculus GN=Ubr2 PE=1 SV=2
A2AN08	0.004229329	26	24	34	6	12	17	14	11	E3 ubiquitin-protein ligase UBR4 OS=Mus musculus GN=Ubr4 PE=1 SV=1
A0A0R4J008	0.015162576	17	12	19	2	10	0	5	0	Histone deacetylase OS=Mus musculus GN=Hdac2 PE=1 SV=1
Q3TTF6	0.024930486	4	8	6	2	0	0	0	3	Putative uncharacterized protein OS=Mus musculus GN=Ppp2r1b PE=1 SV=1
H3BKU1	0.024930486	4	8	6	2	0	0	0	3	Protein phosphatase 2 (Formerly 2A), regulatory subunit A (PR 65), beta isoform, isoform CRA_b OS=Mus musculus GN=Ppp2r1b PE=1 SV=1
G3UWS4	0.024930486	4	8	6	2	0	0	0	3	Serine/threonine-protein phosphatase 2A 65 kDa regulatory subunit A beta isoform OS=Mus musculus GN=Ppp2r1b PE=1 SV=1
H3BLQ0	0.024930486	4	8	6	2	0	0	0	3	Serine/threonine-protein phosphatase 2A 65 kDa regulatory subunit A beta isoform OS=Mus musculus GN=Ppp2r1b PE=1 SV=1
E9PVA8	0.075655815	18	11	22	4	9	9	5	10	eIF-2-alpha kinase activator GCN1 OS=Mus musculus GN=Gcn1 PE=1 SV=1
A0A0U1RQ71	0.438224859	5	0	5	0	0	0	0	3	40S ribosomal protein S13 OS=Mus musculus GN=Rps13 PE=1 SV=1
P60670	0.703915995	0	0	5	0	3	0	0	0	Nuclear protein localization protein 4 homolog OS=Mus musculus GN=Nploc4 PE=1 SV=3
G3UWN9	0.748345559	0	0	10	0	0	0	0	6	Proteasome activator complex subunit 1 (Fragment) OS=Mus musculus GN=Psme1 PE=1 SV=1
Q3TXS7	0.002910618	42	38	31	11	23	19	13	18	26S proteasome non-ATPase regulatory subunit 1 OS=Mus musculus GN=Psmd1 PE=1 SV=1
Q9D0R2	0.24624495	68	36	47	2	27	22	19	23	Threonine--tRNA ligase, cytoplasmic OS=Mus musculus GN=Tars PE=1 SV=2
Q9D0L8	0.651626178	13	5	11	0	7	5	5	6	mRNA cap guanine-N7 methyltransferase OS=Mus musculus GN=Rnmt PE=1 SV=1
E0CY23	0.777203558	0	4	5	0	2	3	0	3	Heat shock 70 kDa protein 4L (Fragment) OS=Mus musculus GN=Hspa4l PE=1 SV=1
P25206	0.038992609	17	13	26	5	11	11	9	5	DNA replication licensing factor MCM3 OS=Mus musculus GN=Mcm3 PE=1 SV=2
A0A0R4J049	0.002463412	18	15	13	5	7	8	4	11	Protein arginine N-methyltransferase 5 OS=Mus musculus GN=Prmt5 PE=1 SV=1
G3UXI6	0.11639109	17	18	15	13	10	10	7	10	Spliceosome RNA helicase Ddx39b (Fragment) OS=Mus musculus GN=Ddx39b PE=1 SV=1
P19253	0.635777941	5	6	12	0	6	6	2	4	60S ribosomal protein L13a OS=Mus musculus GN=Rpl13a PE=1 SV=4
Q8CGC7	0.004590521	164	134	131	39	69	68	51	85	Bifunctional glutamate/proline--tRNA ligase OS=Mus musculus GN=Eprs PE=1 SV=4
A0A0G2JG00	0.905365696	5	3	0	0	3	0	2	2	Far upstream element-binding protein 1 OS=Mus musculus GN=Fubp1 PE=1 SV=1
J3KMQ2	0.000750278	44	34	42	14	19	21	16	22	Protein Gm5422 OS=Mus musculus GN=Gm5422 PE=4 SV=1
Q99KP6	0.172164634	23	19	23	2	13	14	2	10	Pre-mRNA-processing factor 19 OS=Mus musculus GN=Prpf19 PE=1 SV=1
Q9R0E1	0.001179949	19	17	10	4	11	11	3	4	Procollagen-lysine,2-oxoglutarate 5-dioxygenase 3 OS=Mus musculus GN=Plod3 PE=1 SV=1
P54823	0.571570099	15	14	15	0	12	7	4	11	Probable ATP-dependent RNA helicase DDX6 OS=Mus musculus GN=Ddx6 PE=1 SV=1
Q5SW19	0.006960615	19	18	20	7	11	7	9	10	Clustered mitochondria protein homolog OS=Mus musculus GN=Cluh PE=1 SV=2
A0A0R4J140	0.006960615	19	18	20	7	11	7	9	10	Clustered mitochondria protein homolog OS=Mus musculus GN=Cluh PE=1 SV=1
P21278	0.0587275	8	9	11	2	4	5	0	4	Guanine nucleotide-binding protein subunit alpha-11 OS=Mus musculus GN=Gna11 PE=1 SV=1
Q8BJW6	0.397256345	5	4	5	0	2	3	0	3	Eukaryotic translation initiation factor 2A OS=Mus musculus GN=Eif2a PE=1 SV=2
A0A1B0GS70	0.47482706	4	6	4	0	3	0	2	3	Proteasome endopeptidase complex OS=Mus musculus GN=Psma1 PE=1 SV=1
O08759	0.002071619	8	6	5	2	3	0	0	0	Ubiquitin-protein ligase E3A OS=Mus musculus GN=Ube3a PE=1 SV=2
O08582	0.070748183	5	4	2	3	2	0	0	0	GTP-binding protein 1 OS=Mus musculus GN=Gtpbp1 PE=1 SV=2
Q3U4X8	0.202527389	6	3	12	0	4	0	0	0	DNA ligase OS=Mus musculus GN=Lig1 PE=1 SV=1
P37913	0.202527389	6	3	12	0	4	0	0	0	DNA ligase 1 OS=Mus musculus GN=Lig1 PE=1 SV=2
G3UY52	0.347352812	8	5	8	0	2	0	0	6	Probable ubiquitin carboxyl-terminal hydrolase FAF-X (Fragment) OS=Mus musculus GN=Usp9x PE=1 SV=1
Q99KE1	0.365531693	0	4	3	0	2	0	0	0	NAD-dependent malic enzyme, mitochondrial OS=Mus musculus GN=Me2 PE=1 SV=1
P51807	0.694813806	6	0	8	0	4	4	4	4	Dynein light chain Tctex-type 1 OS=Mus musculus GN=Dynlt1 PE=1 SV=1
Q80ZS7	0.475986993	14	12	25	0	0	7	8	14	MCG49550 OS=Mus musculus GN=BC048507 PE=1 SV=1
H3BJP2	0.003055521	52	44	40	7	21	22	18	0	S-formylglutathione hydrolase (Fragment) OS=Mus musculus GN=Esd PE=1 SV=1
Q5SQB0	0.004865949	59	33	35	12	27	18	15	19	Nucleophosmin OS=Mus musculus GN=Npm1 PE=1 SV=1
Q9D4H8	0.151455695	10	22	12	10	0	5	12	6	Cullin-2 OS=Mus musculus GN=Cul2 PE=1 SV=2
Q7TMB8	0.009509687	41	27	26	17	14	15	8	26	Cytoplasmic FMR1-interacting protein 1 OS=Mus musculus GN=Cyfip1 PE=1 SV=1
A0A140T8K2	0.887144665	4	0	11	0	5	6	2	4	Protein Rpl13a-ps1 OS=Mus musculus GN=Rpl13a-ps1 PE=3 SV=1
H3BLJ9	0.002298299	52	44	41	7	21	22	18	0	S-formylglutathione hydrolase OS=Mus musculus GN=Esd PE=1 SV=1
Q91VR5	0.356306863	7	9	7	0	3	5	0	5	ATP-dependent RNA helicase DDX1 OS=Mus musculus GN=Ddx1 PE=1 SV=1
E9PZX7	0.007064997	9	10	11	2	3	7	4	4	Cyclin-dependent kinase 4 (Fragment) OS=Mus musculus GN=Cdk4 PE=1 SV=1
O08529	0.015974083	24	17	12	4	12	8	2	10	Calpain-2 catalytic subunit OS=Mus musculus GN=Capn2 PE=1 SV=4
Q9JJ28	0.065240458	12	12	10	7	4	6	6	7	Protein flightless-1 homolog OS=Mus musculus GN=Flii PE=1 SV=1
A0A0N4SVP8	0.38592846	50	32	0	9	14	22	12	20	Protein Gm5580 OS=Mus musculus GN=Gm5580 PE=3 SV=1
Q61937	0.00429505	59	34	35	13	27	18	15	19	Nucleophosmin OS=Mus musculus GN=Npm1 PE=1 SV=1
G3UZ34	0.013697808	27	21	22	5	10	11	10	11	116 kDa U5 small nuclear ribonucleoprotein component OS=Mus musculus GN=Eftud2 PE=1 SV=1
Q5SQB5	0.003449063	58	34	35	13	27	17	15	19	Nucleophosmin OS=Mus musculus GN=Npm1 PE=1 SV=1
Z4YLI8	0.008810035	18	18	20	7	11	6	8	10	Clustered mitochondria protein homolog OS=Mus musculus GN=Cluh PE=1 SV=1
A0A0G2JDK2	0.363409644	0	0	7	2	3	0	0	2	Glucosylceramidase OS=Mus musculus GN=Gba PE=1 SV=1
E9Q156	0.588345299	12	11	4	0	0	3	6	6	tRNA (guanine-N(7)-)-methyltransferase non-catalytic subunit WDR4 OS=Mus musculus GN=Wdr4 PE=1 SV=1
Q9EP82	0.588345299	12	11	4	0	0	3	6	6	tRNA (guanine-N(7)-)-methyltransferase non-catalytic subunit WDR4 OS=Mus musculus GN=Wdr4 PE=1 SV=2
P14685	0.001717401	71	58	71	18	25	32	34	30	26S proteasome non-ATPase regulatory subunit 3 OS=Mus musculus GN=Psmd3 PE=1 SV=3
P50580	0.0130723	41	36	53	11	23	19	15	21	Proliferation-associated protein 2G4 OS=Mus musculus GN=Pa2g4 PE=1 SV=3
Q04736	0.372306369	5	4	10	0	2	0	0	5	Tyrosine-protein kinase Yes OS=Mus musculus GN=Yes1 PE=1 SV=3
O08810	0.019259189	28	22	23	5	10	11	10	12	116 kDa U5 small nuclear ribonucleoprotein component OS=Mus musculus GN=Eftud2 PE=1 SV=1
A2AH85	0.019259189	28	22	23	5	10	11	10	12	116 kDa U5 small nuclear ribonucleoprotein component OS=Mus musculus GN=Eftud2 PE=1 SV=1
Q8BVG4	0.231912342	2	4	5	0	0	0	0	2	Dipeptidyl peptidase 9 OS=Mus musculus GN=Dpp9 PE=1 SV=2
P25444	0.007287561	132	107	110	46	37	30	47	101	40S ribosomal protein S2 OS=Mus musculus GN=Rps2 PE=1 SV=3
B1AWE1	0.022323179	21	15	13	8	8	5	7	11	Clathrin light chain A OS=Mus musculus GN=Clta PE=1 SV=1
B1AWE0	0.0327684	21	15	13	8	7	5	8	11	Clathrin light chain A OS=Mus musculus GN=Clta PE=1 SV=1
B1AWD9	0.0327684	21	15	13	8	7	5	8	11	Clathrin light chain A OS=Mus musculus GN=Clta PE=1 SV=1
Q6PFA2	0.0327684	21	15	13	8	7	5	8	11	Clathrin light chain A OS=Mus musculus GN=Clta PE=1 SV=1
O08585	0.0327684	21	15	13	8	7	5	8	11	Clathrin light chain A OS=Mus musculus GN=Clta PE=1 SV=2
Q9WVJ2	0.180854868	33	25	31	3	6	17	9	18	26S proteasome non-ATPase regulatory subunit 13 OS=Mus musculus GN=Psmd13 PE=1 SV=1
Q99LC8	0.597543235	6	8	4	0	3	3	4	3	Translation initiation factor eIF-2B subunit alpha OS=Mus musculus GN=Eif2b1 PE=1 SV=1
Q8CIH9	0.585040415	14	4	14	0	8	6	3	6	Amidophosphoribosyltransferase OS=Mus musculus GN=Ppat PE=1 SV=1
O35286	0.110316427	96	60	67	8	39	31	16	38	Pre-mRNA-splicing factor ATP-dependent RNA helicase DHX15 OS=Mus musculus GN=Dhx15 PE=1 SV=2
D3Z061	0.143277166	17	12	10	2	3	6	5	8	Ubiquitin-like modifier-activating enzyme 6 OS=Mus musculus GN=Uba6 PE=1 SV=1
Q8C7R4	0.143277166	17	12	10	2	3	6	5	8	Ubiquitin-like modifier-activating enzyme 6 OS=Mus musculus GN=Uba6 PE=1 SV=1
O09106	0.012940096	27	24	31	15	11	14	6	21	Histone deacetylase 1 OS=Mus musculus GN=Hdac1 PE=1 SV=1
Q6PHN9	0.530264978	14	12	9	0	6	6	7	6	Ras-related protein Rab-35 OS=Mus musculus GN=Rab35 PE=1 SV=1
F6SJS2	0.08062176	11	3	4	2	2	3	0	3	Host cell factor 1 (Fragment) OS=Mus musculus GN=Hcfc1 PE=1 SV=1
Q9WTX5	0.339035275	4	4	7	0	0	4	2	2	S-phase kinase-associated protein 1 OS=Mus musculus GN=Skp1 PE=1 SV=3
Q6PI63	0.885028966	6	4	0	0	0	3	2	3	DNA-directed RNA polymerase II subunit RPB11 OS=Mus musculus GN=Polr2j PE=1 SV=1
P59999	0.72687304	20	5	6	0	4	6	4	8	Actin-related protein 2/3 complex subunit 4 OS=Mus musculus GN=Arpc4 PE=1 SV=3
D3YYI8	0.015919301	26	24	31	15	11	14	6	20	Histone deacetylase OS=Mus musculus GN=Gm10093 PE=3 SV=1
Q7TPV4	0.019451088	41	44	41	10	18	17	13	24	Myb-binding protein 1A OS=Mus musculus GN=Mybbp1a PE=1 SV=2
Q9QUJ7	0.011602103	6	8	3	2	2	3	0	0	Long-chain-fatty-acid--CoA ligase 4 OS=Mus musculus GN=Acsl4 PE=1 SV=2
Q9ES00	0.087876249	19	9	7	3	5	4	4	7	Ubiquitin conjugation factor E4 B OS=Mus musculus GN=Ube4b PE=1 SV=3
contaminant_GR78_SCHPO	0.308372448	8	6	6	0	0	0	4	3	owl|P36604| 78 KD GLUCOSE REGULATED PROTEIN HOMOLOG PRECURSOR (GRP 78) (IMMUNOGLOBULIN...
J3QMG5	0.010693977	127	102	101	46	27	25	46	98	Protein Gm5786 OS=Mus musculus GN=Gm5786 PE=3 SV=1
P47857	0.053648547	17	10	18	3	6	10	5	4	ATP-dependent 6-phosphofructokinase, muscle type OS=Mus musculus GN=Pfkm PE=1 SV=3
P12382	0.013744955	43	36	35	9	23	15	6	20	ATP-dependent 6-phosphofructokinase, liver type OS=Mus musculus GN=Pfkl PE=1 SV=4
Q9CZ30	0.081622886	30	20	23	4	16	7	2	15	Obg-like ATPase 1 OS=Mus musculus GN=Ola1 PE=1 SV=1

Q8C266	0.409685858	8	8	13	0	5	3	0	7 Putative uncharacterized protein OS=Mus musculus GN=Rab5c PE=1 SV=1
Q91VC3	0.036944523	53	33	43	9	14	24	12	21 Eukaryotic initiation factor 4A-III OS=Mus musculus GN=Eif4a3 PE=1 SV=3
Q9JM76	0.037036161	34	21	25	16	20	8	0	9 Actin-related protein 2/3 complex subunit 3 OS=Mus musculus GN=Arpc3 PE=1 SV=3
Q9JHR7	0.011073152	17	14	8	4	6	0	0	5 Insulin-degrading enzyme OS=Mus musculus GN=Ide PE=1 SV=1
E9Q5I9	0.145230243	31	24	30	3	5	15	9	16 26S proteasome non-ATPase regulatory subunit 13 OS=Mus musculus GN=Psmd13 PE=1 SV=1
D3Z124	0.407716427	9	9	7	0	5	4	5	3 DNA-(apurinic or apyrimidinic site) lyase (Fragment) OS=Mus musculus GN=Apex1 PE=1 SV=1
P49717	0.002361299	50	46	44	15	24	18	17	20 DNA replication licensing factor MCM4 OS=Mus musculus GN=Mcm4 PE=1 SV=1
A0A140T8L5	0.00529924	126	104	101	46	33	25	39	94 Protein Rps2-ps6 OS=Mus musculus GN=Rps2-ps6 PE=3 SV=1
E9QKR0	0.003968425	120	97	86	37	42	38	36	55 Guanine nucleotide-binding protein G(I)/G(S)/G(T) subunit beta-2 OS=Mus musculus GN=Gnb2 PE=1 SV=1
P62880	0.004031638	121	97	86	37	42	38	36	55 Guanine nucleotide-binding protein G(I)/G(S)/G(T) subunit beta-2 OS=Mus musculus GN=Gnb2 PE=1 SV=3
A0A087WQ46	0.326637035	9	8	11	0	6	3	0	5 Nucleolar protein 58 (Fragment) OS=Mus musculus GN=Nop58 PE=1 SV=1
F6VW30	0.003728583	89	82	76	41	22	26	29	67 14-3-3 protein theta (Fragment) OS=Mus musculus GN=Ywhaq PE=1 SV=1
F6YTZ4	0.00729791	125	104	103	46	26	25	43	95 Protein Gm6576 OS=Mus musculus GN=Gm6576 PE=3 SV=2
O88544	0.01581993	17	8	12	3	7	4	0	4 COP9 signalosome complex subunit 4 OS=Mus musculus GN=Cops4 PE=1 SV=1
A0A0G2JDF8	0.018823219	7	8	10	3	5	2	3	4 Serrate RNA effector molecule homolog (Fragment) OS=Mus musculus GN=Srrt PE=1 SV=1
E9PXY1	0.034983958	5	8	12	7	0	0	0	4 Cullin-4B OS=Mus musculus GN=Cul4b PE=1 SV=2
A2A432	0.034983958	5	8	12	7	0	0	0	4 Cullin-4B OS=Mus musculus GN=Cul4b PE=1 SV=1
Q8K1K2	0.046991785	19	20	16	3	11	8	2	8 26S protease regulatory subunit 8 OS=Mus musculus GN=Psmc5 PE=1 SV=1
A2AGT5	0.048739816	5	3	4	4	0	0	0	2 Cytoskeleton-associated protein 5 OS=Mus musculus GN=Ckap5 PE=1 SV=1
Z4YL78	0.048739816	5	3	4	4	0	0	0	2 Cytoskeleton-associated protein 5 OS=Mus musculus GN=Ckap5 PE=1 SV=1
K3W4R5	0.048739816	5	3	4	4	0	0	0	2 Cytoskeleton-associated protein 5 OS=Mus musculus GN=Ckap5 PE=1 SV=1
A0A0R4J0K2	0.048739816	5	3	4	4	0	0	0	2 Cytoskeleton-associated protein 5 OS=Mus musculus GN=Ckap5 PE=1 SV=1
Q61210	0.086995489	5	0	5	2	2	0	0	0 Rho guanine nucleotide exchange factor 1 OS=Mus musculus GN=Arhgef1 PE=1 SV=2
E9PUF7	0.086995489	5	0	5	2	2	0	0	0 Rho guanine nucleotide exchange factor 1 OS=Mus musculus GN=Arhgef1 PE=1 SV=1
F6ZN61	0.086995489	5	0	5	2	2	0	0	0 Rho guanine nucleotide exchange factor 1 (Fragment) OS=Mus musculus GN=Arhgef1 PE=1 SV=1
F6QKK2	0.17099986	5	4	3	0	0	2	2	0 ADP-ribosylation factor-like protein 8A (Fragment) OS=Mus musculus GN=Arl8a PE=1 SV=1
Q8VEH3	0.17099986	5	4	3	0	0	2	2	0 ADP-ribosylation factor-like protein 8A OS=Mus musculus GN=Arl8a PE=1 SV=1
P97823	0.199743087	6	5	4	0	3	0	0	2 Acyl-protein thioesterase 1 OS=Mus musculus GN=Lypla1 PE=1 SV=1
J3QP56	0.199743087	6	5	4	0	3	0	0	2 Acyl-protein thioesterase 1 OS=Mus musculus GN=Lypla1 PE=1 SV=1
Q924K8	0.213922502	8	5	5	0	3	0	0	3 Metastasis-associated protein MTA3 OS=Mus musculus GN=Mta3 PE=1 SV=1
Q3U3A7	0.213922502	8	5	5	0	3	0	0	3 Metastasis-associated protein MTA3 OS=Mus musculus GN=Mta3 PE=1 SV=1
Q3UII8	0.213922502	8	5	5	0	3	0	0	3 Metastasis-associated protein MTA3 OS=Mus musculus GN=Mta3 PE=1 SV=1
E9Q794	0.213922502	8	5	5	0	3	0	0	3 Metastasis-associated protein MTA3 OS=Mus musculus GN=Mta3 PE=1 SV=1
A2A8L1	0.21839457	3	3	6	0	0	0	0	2 Chromodomain-helicase-DNA-binding protein 5 OS=Mus musculus GN=Chd5 PE=1 SV=1
P30681	0.266412525	5	3	7	0	0	0	2	3 High mobility group protein B2 OS=Mus musculus GN=Hmgb2 PE=1 SV=3
Q8CBF3	0.274340091	5	0	7	0	3	0	0	0 Ephrin type-B receptor 1 OS=Mus musculus GN=Ephb1 PE=1 SV=1
Q8C0Z1	0.391002219	4	0	0	0	2	0	0	0 Protein FAM234A OS=Mus musculus GN=Fam234a PE=1 SV=1
A0A140T8T4	0.487379102	0	0	0	6	0	3	0	0 Protein Rpl9-ps6 OS=Mus musculus GN=Rpl9-ps6 PE=4 SV=1
E0CZ90	0.649650771	0	0	6	0	3	0	0	0 Proteasome activator complex subunit 2 (Fragment) OS=Mus musculus GN=Psme2 PE=1 SV=1
E9QM38	0.649650771	0	0	4	0	2	0	0	0 Solute carrier family 12 member 2 OS=Mus musculus GN=Slc12a2 PE=1 SV=1
P55012	0.649650771	0	0	4	0	2	0	0	0 Solute carrier family 12 member 2 OS=Mus musculus GN=Slc12a2 PE=1 SV=2
Q8CBY8	0.649650771	0	0	4	0	2	0	0	0 Dynactin subunit 4 OS=Mus musculus GN=Dctn4 PE=1 SV=1
A0A0A6YW28	0.653248269	0	4	0	0	0	0	0	2 Ubiquitin carboxyl-terminal hydrolase 4 OS=Mus musculus GN=Usp4 PE=1 SV=1
P35123	0.653248269	0	4	0	0	0	0	0	2 Ubiquitin carboxyl-terminal hydrolase 4 OS=Mus musculus GN=Usp4 PE=1 SV=3
Q9JKV1	0.743124526	4	0	0	0	0	0	0	2 Proteasomal ubiquitin receptor ADRM1 OS=Mus musculus GN=Adrm1 PE=1 SV=2
Q9JJA4	0.743124526	4	0	0	0	0	0	0	2 Ribosome biogenesis protein WDR12 OS=Mus musculus GN=Wdr12 PE=1 SV=1
Q6PE70	0.743124526	4	0	0	0	0	0	0	2 Integrin beta OS=Mus musculus GN=Itgb5 PE=1 SV=1
A0A0A6YVU8	0.743124526	4	0	0	0	0	0	0	2 MCG119397 OS=Mus musculus GN=Gm9774 PE=4 SV=1
O70309	0.743124526	4	0	0	0	0	0	0	2 Integrin beta-5 OS=Mus musculus GN=Itgb5 PE=1 SV=2
G5E8F8	0.743124526	4	0	0	0	0	0	0	2 Integrin beta OS=Mus musculus GN=Itgb5 PE=1 SV=1
Q921W0	0.774293415	0	4	0	0	0	0	2	0 Charged multivesicular body protein 1a OS=Mus musculus GN=Chmp1a PE=1 SV=1
O55201	0.859892987	0	5	0	0	3	0	0	2 Transcription elongation factor SPT5 OS=Mus musculus GN=Supt5h PE=1 SV=1
P68254	0.005192705	89	81	76	43	23	26	29	66 14-3-3 protein theta OS=Mus musculus GN=Ywhaq PE=1 SV=1
D3Z536	0.007893512	120	98	98	46	23	22	43	92 Protein Gm8225 OS=Mus musculus GN=Gm8225 PE=3 SV=1
A2AFK7	0.045423891	47	27	37	8	13	22	7	17 Eukaryotic initiation factor 4A-III (Fragment) OS=Mus musculus GN=Eif4a3 PE=1 SV=1
P62196	0.034697347	24	30	28	7	18	13	2	11 26S protease regulatory subunit 8 OS=Mus musculus GN=Psmc5 PE=1 SV=1
Q9WUA2	0.447235106	14	15	15	0	14	4	2	9 Phenylalanine--tRNA ligase beta subunit OS=Mus musculus GN=Farsb PE=1 SV=2
D3YVC1	0.004431373	123	103	109	46	24	30	47	86 40S ribosomal protein S2 (Fragment) OS=Mus musculus GN=Rps2 PE=1 SV=1
P28352	0.345037463	9	9	8	0	5	4	5	3 DNA-(apurinic or apyrimidinic site) lyase OS=Mus musculus GN=Apex1 PE=1 SV=2
F6QQQ6	0.42474004	9	9	8	0	3	5	4	5 Protein arginine N-methyltransferase 5 (Fragment) OS=Mus musculus GN=Prmt5 PE=1 SV=8
Q3UYV9	0.061157295	17	11	10	3	3	5	6	6 Nuclear cap-binding protein subunit 1 OS=Mus musculus GN=Ncbp1 PE=1 SV=2
G3UZD6	0.081495466	18	9	7	3	4	3	4	7 Ubiquitin conjugation factor E4 B OS=Mus musculus GN=Ube4b PE=1 SV=1
A0A0J9YV30	0.199370798	10	14	4	9	5	4	5	4 Paxillin OS=Mus musculus GN=Pxn PE=1 SV=1
Q8VI36	0.199370798	10	14	4	9	5	4	5	4 Paxillin OS=Mus musculus GN=Pxn PE=1 SV=1
F8VQ28	0.199370798	10	14	4	9	5	4	5	4 Paxillin OS=Mus musculus GN=Pxn PE=1 SV=1
H7BWZ3	0.546173821	25	15	0	0	12	8	0	9 Actin-related protein 2/3 complex subunit 3 OS=Mus musculus GN=Arpc3 PE=1 SV=1
A0A087WP81	0.114293821	12	6	7	0	0	4	0	0 Ubiquitin carboxyl-terminal hydrolase isozyme L5 (Fragment) OS=Mus musculus GN=Uchl5 PE=1 SV=1
D3YZX3	0.006586985	113	94	82	37	38	34	36	48 Guanine nucleotide-binding protein G(I)/G(S)/G(T) subunit beta-2 OS=Mus musculus GN=Gnb2 PE=1 SV=1
F6RPJ9	0.007630598	19	14	9	4	6	0	0	5 Insulin-degrading enzyme (Fragment) OS=Mus musculus GN=Ide PE=1 SV=1
A0A0U1RQ05	0.022058445	38	26	23	17	12	12	7	18 Cytoplasmic FMR1-interacting protein 1 (Fragment) OS=Mus musculus GN=Cyfip1 PE=1 SV=1
Q8VDM6	0.021104649	12	8	10	4	7	3	4	2 Heterogeneous nuclear ribonucleoprotein U-like protein 1 OS=Mus musculus GN=Hnrnpul1 PE=1 SV=1
Q9QUR6	0.018083317	70	48	63	14	32	17	10	32 Prolyl endopeptidase OS=Mus musculus GN=Prep PE=1 SV=1
Q99J09	0.000717869	13	11	12	5	4	4	4	7 Methylosome protein 50 OS=Mus musculus GN=Wdr77 PE=1 SV=1
Q9DC48	0.059002999	4	5	4	0	0	2	0	0 Pre-mRNA-processing factor 17 OS=Mus musculus GN=Cdc40 PE=2 SV=1
P35279	0.062979907	5	5	3	0	0	2	0	0 Ras-related protein Rab-6A OS=Mus musculus GN=Rab6a PE=1 SV=4
Q3UKV0	0.075688068	4	4	5	0	0	0	2	0 Protein Eif2b3 OS=Mus musculus GN=Eif2b3 PE=1 SV=1
B1AUN2	0.075688068	4	4	5	0	0	0	2	0 MCG14442 OS=Mus musculus GN=Eif2b3 PE=1 SV=1
B1AUN3	0.075688068	4	4	5	0	0	0	2	0 Protein Eif2b3 OS=Mus musculus GN=Eif2b3 PE=1 SV=1
P58242	0.138682635	3	4	6	0	2	0	0	0 Acid sphingomyelinase-like phosphodiesterase 3b OS=Mus musculus GN=Smpdl3b PE=1 SV=1
Q8BQM4	0.186822723	5	5	3	0	0	0	0	2 HEAT repeat-containing protein 3 OS=Mus musculus GN=Heatr3 PE=1 SV=1
D3Z2T9	0.027631466	46	31	42	5	18	13	14	12 Cyclin-dependent kinase 1 (Fragment) OS=Mus musculus GN=Cdk1 PE=1 SV=1
Q91ZW3	0.00999904	29	27	28	12	12	12	10	10 SWI/SNF-related matrix-associated actin-dependent regulator of chromatin subfamily A member 5 OS=Mus musculus GN=Smarca5 PE=1 SV=1
Q9D1C8	0.501207041	6	3	9	0	2	3	2	4 Vacuolar protein sorting-associated protein 28 homolog OS=Mus musculus GN=Vps28 PE=1 SV=1
P62855	0.449083163	23	13	10	0	8	5	5	10 40S ribosomal protein S26 OS=Mus musculus GN=Rps26 PE=1 SV=3
Q3TCH7	0.084106907	7	11	13	10	0	4	4	6 Cullin-4A OS=Mus musculus GN=Cul4a PE=1 SV=1
Q78ZA7	0.608673948	3	0	8	0	0	3	0	2 Nucleosome assembly protein 1-like 4 OS=Mus musculus GN=Nap1l4 PE=1 SV=1
Q8BU30	0.018119079	78	59	50	14	24	20	19	28 Isoleucine--tRNA ligase, cytoplasmic OS=Mus musculus GN=Iars PE=1 SV=2
F7D5L2	0.002094349	13	10	12	5	4	4	4	6 Methylosome protein 50 (Fragment) OS=Mus musculus GN=Wdr77 PE=1 SV=1
A0A1B0GS68	0.142644386	5	5	10	0	4	0	2	0 Fms-related tyrosine kinase 3 ligand OS=Mus musculus GN=Flt3l PE=3 SV=1
Q9CPN9	0.225157863	7	6	7	0	4	0	3	2 Protein 2210010C04Rik OS=Mus musculus GN=2210010C04Rik PE=1 SV=1
Q8VDW0	0.01163721	37	20	25	5	17	6	6	10 ATP-dependent RNA helicase DDX39A OS=Mus musculus GN=Ddx39a PE=1 SV=1
Q3THK7	0.001755439	40	33	33	15	17	11	8	18 GMP synthase [glutamine-hydrolyzing] OS=Mus musculus GN=Gmps PE=1 SV=2
Q9QZD9	0.051734362	41	35	38	5	16	13	8	16 Eukaryotic translation initiation factor 3 subunit I OS=Mus musculus GN=Eif3i PE=1 SV=1
Q9QXK3	0.399147424	5	3	10	0	3	2	0	3 Coatomer subunit gamma-2 OS=Mus musculus GN=Copg2 PE=1 SV=1
Q99KK7	0.351351021	5	4	0	0	0	0	0	2 Dipeptidyl peptidase 3 OS=Mus musculus GN=Dpp3 PE=1 SV=2
P36916	0.353036241	0	5	4	0	2	2	0	0 Guanine nucleotide-binding protein-like 1 OS=Mus musculus GN=Gnl1 PE=1 SV=4
P32233	0.176347054	4	5	8	0	3	2	0	0 Developmentally-regulated GTP-binding protein 1 OS=Mus musculus GN=Drg1 PE=1 SV=1
Q8BKC5	0.014985966	26	21	21	10	10	9	8	7 Importin-5 OS=Mus musculus GN=Ipo5 PE=1 SV=3
Q4FE56	0.006244174	47	46	41	13	16	13	11	24 Probable ubiquitin carboxyl-terminal hydrolase FAF-X OS=Mus musculus GN=Usp9x PE=1 SV=1
P70398	0.006244174	47	46	41	13	16	13	11	24 Probable ubiquitin carboxyl-terminal hydrolase FAF-X OS=Mus musculus GN=Usp9x PE=1 SV=2
Q8BY71	0.046494768	14	10	11	2	3	3	0	6 Histone acetyltransferase type B catalytic subunit OS=Mus musculus GN=Hat1 PE=1 SV=1
A2ATU9	0.046494768	14	10	11	2	3	3	0	6 Histone acetyltransferase type B catalytic subunit OS=Mus musculus GN=Hat1 PE=1 SV=1
P30285	0.000959084	12	13	15	4	4	7	4	4 Cyclin-dependent kinase 4 OS=Mus musculus GN=Cdk4 PE=1 SV=1
Q9WUP7	0.097915466	13	7	8	0	0	4	0	0 Ubiquitin carboxyl-terminal hydrolase isozyme L5 OS=Mus musculus GN=Uchl5 PE=1 SV=2
E9Q0U1	0.024974368	18	11	17	3	4	5	5	7 26S proteasome non-ATPase regulatory subunit 13 OS=Mus musculus GN=Psmd13 PE=1 SV=1
P54071	0.118459427	12	7	9	7	3	4	5	3 Isocitrate dehydrogenase [NADP], mitochondrial OS=Mus musculus GN=Idh2 PE=1 SV=3
G3X928	0.219620278	8	7	6	0	2	4	0	3 SEC23-interacting protein OS=Mus musculus GN=Sec23ip PE=1 SV=1
Q6NZC7	0.219620278	8	7	6	0	2	4	0	3 SEC23-interacting protein OS=Mus musculus GN=Sec23ip PE=1 SV=2
A2A4J3	0.344910652	8	6	0	0	0	0	0	3 Proteasome activator complex subunit 3 (Fragment) OS=Mus musculus GN=Psme3 PE=1 SV=1
O54950	0.06647788	4	5	5	0	0	2	0	0 5'-AMP-activated protein kinase subunit gamma-1 OS=Mus musculus GN=Prkag1 PE=1 SV=2
Q5SSW2	0.18117417	4	2	8	0	0	2	2	0 Proteasome activator complex subunit 4 OS=Mus musculus GN=Psme4 PE=1 SV=1
G3XA48	0.252653903	4	3	7	0	2	0	0	2 Isopentenyl-diphosphate Delta-isomerase 1 OS=Mus musculus GN=Idi1 PE=1 SV=1
P58044	0.252653903	4	3	7	0	2	0	0	2 Isopentenyl-diphosphate Delta-isomerase 1 OS=Mus musculus GN=Idi1 PE=1 SV=1
P46471	0.007441821	61	56	49	21	18	21	21	20 26S protease regulatory subunit 7 OS=Mus musculus GN=Psmc2 PE=1 SV=5
Q8BVQ9	0.007255756	61	55	49	21	17	21	21	20 26S protease regulatory subunit 7 OS=Mus musculus GN=Psmc2 PE=1 SV=1
Q6DFW4	0.044909028	16	14	18	2	8	4	4	5 Nucleolar protein 58 OS=Mus musculus GN=Nop58 PE=1 SV=1
Q9CQC6	0.914818976	6	0	0	0	0	2	3	0 Basic leucine zipper and W2 domain-containing protein 1 OS=Mus musculus GN=Bzw1 PE=1 SV=1
A0A087WQS2	0.914818976	6	0	0	0	0	2	3	0 Basic leucine zipper and W2 domain-containing protein 1 OS=Mus musculus GN=Bzw1 PE=1 SV=1
Q8BFY9	0.000639064	32	22	24	11	10	7	6	14 Transportin-1 OS=Mus musculus GN=Tnpo1 PE=1 SV=2
D3Z5N9	0.000474157	10	8	8	3	0	0	0	3 MCG49198 OS=Mus musculus GN=Gm5449 PE=4 SV=1
Q6P8X1	0.193986355	10	10	9	0	4	0	0	4 Sorting nexin-6 OS=Mus musculus GN=Snx6 PE=1 SV=2
P11440	0.059279497	78	49	57	5	26	19	15	17 Cyclin-dependent kinase 1 OS=Mus musculus GN=Cdk1 PE=1 SV=3
Q9Z1F9	0.002094517	47	35	43	13	19	18	11	8 SUMO-activating enzyme subunit 2 OS=Mus musculus GN=Uba2 PE=1 SV=1
Q9QYI3	0.261406353	9	5	12	0	0	3	0	4 DnaJ homolog subfamily C member 7 OS=Mus musculus GN=Dnajc7 PE=1 SV=2
F7BTP8	0.261406353	9	5	12	0	0	3	0	4 DnaJ homolog subfamily C member 7 (Fragment) OS=Mus musculus GN=Dnajc7 PE=1 SV=1
P14094	0.000814801	18	20	22	7	5	8	7	7 Sodium/potassium-transporting ATPase subunit beta-1 OS=Mus musculus GN=Atp1b1 PE=1 SV=1
Q9JLJ2	0.002155254	32	22	21	7	10	7	5	11 4-trimethylaminobutyraldehyde dehydrogenase OS=Mus musculus GN=Aldh9a1 PE=1 SV=1
Q3U367	0.002155254	32	22	21	7	10	7	5	11 4-trimethylaminobutyraldehyde dehydrogenase OS=Mus musculus GN=Aldh9a1 PE=1 SV=1
Q62470	0.006048741	90	51	69	19	20	24	22	26 Integrin alpha-3 OS=Mus musculus GN=Itga3 PE=1 SV=1
P62082	0.005157787	17	14	10	4	6	4	3	5 40S ribosomal protein S7 OS=Mus musculus GN=Rps7 PE=2 SV=1
A0A0A0MQ76	0.034141922	16	14	18	2	8	4	4	4 Nucleolar protein 58 OS=Mus musculus GN=Nop58 PE=1 SV=1
Q9WUA3	0.053465777	17	18	22	3	6	5	0	7 ATP-dependent 6-phosphofructokinase, platelet type OS=Mus musculus GN=Pfkp PE=1 SV=1
Q8C605	0.053465777	17	18	22	3	6	5	0	7 ATP-dependent 6-phosphofructokinase OS=Mus musculus GN=Pfkp PE=1 SV=1
P31266	0.70219432	5	0	0	0	0	2	0	0 Recombining binding protein suppressor of hairless OS=Mus musculus GN=Rbpj PE=1 SV=1
Q3U6F1	0.70219432	5	0	0	0	0	2	0	0 Putative uncharacterized protein OS=Mus musculus GN=Rbpj PE=1 SV=1
E9Q7W0	0.70219432	5	0	0	0	0	2	0	0 Recombining-binding protein suppressor of hairless OS=Mus musculus GN=Rbpj PE=1 SV=1
A0A0J9YTV5	0.70219432	5	0	0	0	0	2	0	0 Recombining binding protein suppressor of hairless (Drosophila), isoform CRA_b OS=Mus musculus GN=Rbpj PE=1 SV=1
A0A1B0GSQ6	0.798107232	5	0	0	0	0	0	2	0 60S ribosomal protein L13a (Fragment) OS=Mus musculus GN=Rpl13a PE=3 SV=1
A0A1B0GTA1	0.798107232	5	0	0	0	0	0	2	0 60S ribosomal protein L13a (Fragment) OS=Mus musculus GN=Rpl13a PE=3 SV=1
E9Q1T9	0.000258219	73	50	56	17	24	19	13	22 Exportin-2 OS=Mus musculus GN=Cse1l PE=1 SV=1
P47962	0.00119576	140	124	127	57	45	23	30	79 60S ribosomal protein L5 OS=Mus musculus GN=Rpl5 PE=1 SV=3
A2AR07	0.241157919	7	7	14	0	3	0	4	4 Lupus La protein homolog (Fragment) OS=Mus musculus GN=Ssb PE=1 SV=1
Q9D0C4	0.832741415	0	0	6	0	2	3	2	0 tRNA (guanine(37)-N1)-methyltransferase OS=Mus musculus GN=Trmt5 PE=1 SV=1
Q61881	0.018008192	66	63	64	32	33	18	15	21 DNA replication licensing factor MCM7 OS=Mus musculus GN=Mcm7 PE=1 SV=1
O70133	0.012982991	148	84	90	23	38	34	22	39 ATP-dependent RNA helicase A OS=Mus musculus GN=Dhx9 PE=1 SV=2
E9PV04	0.058565224	50	0	43	9	14	0	12	0 Protein Gm8994 OS=Mus musculus GN=Gm8994 PE=3 SV=2
E9QNN1	0.008196457	148	87	91	25	39	34	22	39 ATP-dependent RNA helicase A OS=Mus musculus GN=Dhx9 PE=1 SV=1
A0A087WPL5	0.008196457	148	87	91	25	39	34	22	39 ATP-dependent RNA helicase A OS=Mus musculus GN=Dhx9 PE=1 SV=1
Q8K2Z4	0.001231506	6	8	5	2	0	2	0	0 Condensin complex subunit 1 OS=Mus musculus GN=Ncapd2 PE=1 SV=2
A0A0R4J0H7	0.001231506	6	8	5	2	0	2	0	0 Condensin complex subunit 1 OS=Mus musculus GN=Ncapd2 PE=1 SV=1
E9Q634	0.004863241	6	5	8	2	0	0	0	2 Unconventional myosin-Ie OS=Mus musculus GN=Myo1e PE=1 SV=1
B7ZWL1	0.010401758	14	10	15	3	0	0	0	4 CCR4-NOT transcription complex subunit 1 OS=Mus musculus GN=Cnot1 PE=1 SV=1
Q6ZQ08	0.010401758	14	10	15	3	0	0	0	4 CCR4-NOT transcription complex subunit 1 OS=Mus musculus GN=Cnot1 PE=1 SV=2
Q62318	0.020296598	16	18	22	7	11	4	3	6 Transcription intermediary factor 1-beta OS=Mus musculus GN=Trim28 PE=1 SV=3
Q9ERK4	0.000545432	77	53	58	17	24	19	13	22 Exportin-2 OS=Mus musculus GN=Cse1l PE=1 SV=1
Q3UE37	0.071280645	6	4	6	0	2	0	0	0 Ubiquitin-conjugating enzyme E2 Z OS=Mus musculus GN=Ube2z PE=1 SV=2
Q5SSI6	0.148979952	6	4	6	0	0	0	0	2 U3 small nucleolar RNA-associated protein 18 homolog OS=Mus musculus GN=Utp18 PE=1 SV=1
P40336	0.158600842	11	5	8	0	0	0	0	3 Vacuolar protein sorting-associated protein 26A OS=Mus musculus GN=Vps26a PE=1 SV=1
P52479	0.175824801	12	4	8	0	0	0	0	3 Ubiquitin carboxyl-terminal hydrolase 10 OS=Mus musculus GN=Usp10 PE=1 SV=3
A2A4J1	0.321031022	9	7	0	0	0	0	0	3 Proteasome activator complex subunit 3 (Fragment) OS=Mus musculus GN=Psme3 PE=1 SV=1
P61290	0.321031022	9	7	0	0	0	0	0	3 Proteasome activator complex subunit 3 OS=Mus musculus GN=Psme3 PE=1 SV=1
Q62376	0.065898091	10	6	4	0	3	2	0	0 U1 small nuclear ribonucleoprotein 70 kDa OS=Mus musculus GN=Snrnp70 PE=1 SV=2
Q04750	0.03243367	15	20	22	10	8	8	4	5 DNA topoisomerase 1 OS=Mus musculus GN=Top1 PE=1 SV=2
Q8CFI7	0.051872042	30	25	27	4	12	6	2	12 DNA-directed RNA polymerase II subunit RPB2 OS=Mus musculus GN=Polr2b PE=1 SV=2
A0A0N4SVL9	0.306365382	8	12	23	0	5	6	0	5 Serine/threonine-protein phosphatase OS=Mus musculus GN=Ppp6c PE=1 SV=1

Q9CQR6	0.306365382	8	12	23	0	5	6	0	5 Serine/threonine-protein phosphatase 6 catalytic subunit OS=Mus musculus GN=Ppp6c PE=1 SV=1
Q99MR6	0.019436289	10	9	16	3	5	2	3	4 Serrate RNA effector molecule homolog OS=Mus musculus GN=Srrt PE=1 SV=1
O55142	0.075253108	20	13	21	3	3	4	3	11 60S ribosomal protein L35a OS=Mus musculus GN=Rpl35a PE=1 SV=2
P09405	0.096950615	12	10	5	2	2	0	4	2 Nucleolin OS=Mus musculus GN=Ncl PE=1 SV=2
P32067	0.266853412	8	8	15	0	5	2	4	4 Lupus La protein homolog OS=Mus musculus GN=Ssb PE=1 SV=1
D3Z2F7	0.876962937	23	0	0	0	10	8	0	7 Actin-related protein 2/3 complex subunit 3 OS=Mus musculus GN=Arpc3 PE=1 SV=1
A0A1B0GQW6	0.729234395	0	0	9	0	3	5	2	3 60S ribosomal protein L13a (Fragment) OS=Mus musculus GN=Rpl13a PE=4 SV=1
Q9JKB1	0.409319681	0	6	8	0	3	0	0	2 Ubiquitin carboxyl-terminal hydrolase isozyme L3 OS=Mus musculus GN=Uchl3 PE=1 SV=2
F6QTS1	0.001498412	15	6	10	3	3	0	0	0 COP9 signalosome complex subunit 4 (Fragment) OS=Mus musculus GN=Cops4 PE=1 SV=1
Q61207	0.068796628	6	5	6	0	0	2	0	0 Prosaposin OS=Mus musculus GN=Psap PE=1 SV=2
E9PZ00	0.068796628	6	5	6	0	0	2	0	0 Prosaposin OS=Mus musculus GN=Psap PE=1 SV=1
Q8BFQ1	0.068796628	6	5	6	0	0	2	0	0 Prosaposin OS=Mus musculus GN=Psap PE=1 SV=1
J3QPG5	0.068796628	6	5	6	0	0	2	0	0 Prosaposin OS=Mus musculus GN=Psap PE=1 SV=1
K3W4L3	0.068796628	6	5	6	0	0	2	0	0 Prosaposin OS=Mus musculus GN=Psap PE=1 SV=1
D3Z780	0.097574647	6	7	4	0	2	0	0	0 Translation initiation factor eIF-2B subunit delta OS=Mus musculus GN=Eif2b4 PE=1 SV=1
Q61749	0.097574647	6	7	4	0	2	0	0	0 Translation initiation factor eIF-2B subunit delta OS=Mus musculus GN=Eif2b4 PE=1 SV=2
Q924C1	0.016776926	14	13	11	2	0	4	0	3 Exportin-5 OS=Mus musculus GN=Xpo5 PE=1 SV=1
P62317	0.003005497	10	8	8	3	0	0	2	3 Small nuclear ribonucleoprotein Sm D2 OS=Mus musculus GN=Snrpd2 PE=1 SV=1
Q920B9	0.007792413	12	6	12	5	3	0	0	3 FACT complex subunit SPT16 OS=Mus musculus GN=Supt16h PE=1 SV=2
G3X956	0.007792413	12	6	12	5	3	0	0	3 FACT complex subunit SPT16 OS=Mus musculus GN=Supt16 PE=1 SV=1
Q99L27	0.008129763	19	10	12	6	0	4	0	4 GMP reductase 2 OS=Mus musculus GN=Gmpr2 PE=1 SV=2
P13439	0.070919551	41	24	33	3	9	9	5	11 Uridine 5'-monophosphate synthase OS=Mus musculus GN=Umps PE=1 SV=3
Q922B2	0.002549983	112	68	91	31	29	31	18	23 Aspartate--tRNA ligase, cytoplasmic OS=Mus musculus GN=Dars PE=1 SV=2
Q8CG48	0.016730601	13	6	3	2	2	0	0	0 Structural maintenance of chromosomes protein 2 OS=Mus musculus GN=Smc2 PE=1 SV=2
Q9JKY0	0.10212985	5	5	8	0	2	0	0	0 Cell differentiation protein RCD1 homolog OS=Mus musculus GN=Rqcd1 PE=1 SV=1
Q6PDQ2	0.152860787	6	4	8	0	0	0	0	2 Chromodomain-helicase-DNA-binding protein 4 OS=Mus musculus GN=Chd4 PE=1 SV=1
E9QAS5	0.152860787	6	4	8	0	0	0	0	2 Chromodomain-helicase-DNA-binding protein 4 OS=Mus musculus GN=Chd4 PE=1 SV=1
E9QAS4	0.152860787	6	4	8	0	0	0	0	2 Chromodomain-helicase-DNA-binding protein 4 OS=Mus musculus GN=Chd4 PE=1 SV=1
O55222	0.237766181	10	7	10	0	3	3	2	4 Integrin-linked protein kinase OS=Mus musculus GN=Ilk PE=1 SV=2
J3QNW0	0.008512109	57	46	40	12	8	13	11	19 DNA (cytosine-5)-methyltransferase OS=Mus musculus GN=Dnmt1 PE=1 SV=1
P13864	0.008512109	57	46	40	12	8	13	11	19 DNA (cytosine-5)-methyltransferase 1 OS=Mus musculus GN=Dnmt1 PE=1 SV=5
Q99PV0	0.073956804	25	10	18	2	0	6	0	3 Pre-mRNA-processing-splicing factor 8 OS=Mus musculus GN=Prpf8 PE=1 SV=2
P27048	0.217277225	13	14	10	0	4	0	5	3 Small nuclear ribonucleoprotein-associated protein B OS=Mus musculus GN=Snrpb PE=1 SV=1
P63163	0.217277225	13	14	10	0	4	0	5	3 Small nuclear ribonucleoprotein-associated protein N OS=Mus musculus GN=Snrpn PE=1 SV=1
Q9R190	0.020434511	24	14	10	5	3	4	3	7 Metastasis-associated protein MTA2 OS=Mus musculus GN=Mta2 PE=1 SV=1
Q9WV60	0.451027464	0	11	8	0	3	2	0	4 Glycogen synthase kinase-3 beta OS=Mus musculus GN=Gsk3b PE=1 SV=2
E9QAQ5	0.451027464	0	11	8	0	3	2	0	4 Glycogen synthase kinase-3 beta OS=Mus musculus GN=Gsk3b PE=1 SV=1
Q9WTL7	0.272491409	0	7	6	0	2	0	0	0 Acyl-protein thioesterase 2 OS=Mus musculus GN=Lypla2 PE=1 SV=1
D6RI87	0.698960769	0	0	13	0	0	0	4	4 Lupus La protein homolog OS=Mus musculus GN=Ssb PE=1 SV=1
Q9EPL8	0.002798021	18	16	18	8	0	5	0	4 Importin-7 OS=Mus musculus GN=Ipo7 PE=1 SV=2
Q9EQU5	0.161092723	19	12	14	0	6	3	5	4 Protein SET OS=Mus musculus GN=Set PE=1 SV=1
A2BE93	0.161092723	19	12	14	0	6	3	5	4 Protein SET (Fragment) OS=Mus musculus GN=Set PE=1 SV=1
P01901	0.188575138	22	18	14	0	5	3	0	8 H-2 class I histocompatibility antigen, K-B alpha chain OS=Mus musculus GN=H2-K1 PE=1 SV=1
P97760	0.012552643	10	8	14	3	2	0	0	3 DNA-directed RNA polymerase II subunit RPB3 OS=Mus musculus GN=Polr2c PE=1 SV=2
A0A1D5RML8	0.012552643	10	8	14	3	2	0	0	3 DNA-directed RNA polymerase II subunit RPB3 OS=Mus musculus GN=Polr2c PE=1 SV=1
E9PWG6	0.036139122	9	8	6	5	0	2	2	2 Protein Ncapg OS=Mus musculus GN=Ncapg PE=1 SV=1
Q4FJZ2	0.199489274	7	11	3	0	0	0	0	2 Importin subunit alpha OS=Mus musculus GN=Kpna6 PE=1 SV=1
D3YYL7	0.274974961	5	9	0	0	2	0	0	0 Protein Gm10126 OS=Mus musculus GN=Gm10126 PE=4 SV=2
S4R1I6	0.005649855	44	33	42	9	12	8	0	7 MCG2872, isoform CRA_b OS=Mus musculus GN=Ddx5 PE=1 SV=1
Q8BTS0	0.002597577	52	37	45	11	13	9	0	8 DEAD (Asp-Glu-Ala-Asp) box polypeptide 5 OS=Mus musculus GN=Ddx5 PE=1 SV=1
O35345	0.189023507	8	11	3	0	0	0	0	2 Importin subunit alpha-7 OS=Mus musculus GN=Kpna6 PE=1 SV=2
Q61191	0.007289029	19	9	12	4	2	3	0	4 Host cell factor 1 OS=Mus musculus GN=Hcfc1 PE=1 SV=2
Q8R010	0.028208623	43	39	42	30	11	0	0	10 Aminoacyl tRNA synthase complex-interacting multifunctional protein 2 OS=Mus musculus GN=Aimp2 PE=1 SV=2
B1AUX2	0.007905807	19	9	13	4	2	3	0	4 Host cell factor 1 OS=Mus musculus GN=Hcfc1 PE=1 SV=1
P43247	0.025519975	14	10	3	3	0	0	0	2 DNA mismatch repair protein Msh2 OS=Mus musculus GN=Msh2 PE=1 SV=1
Q68FL6	0.003944719	56	33	46	11	11	10	5	12 Methionine--tRNA ligase, cytoplasmic OS=Mus musculus GN=Mars PE=1 SV=1
E9QB02	0.003944719	56	33	46	11	11	10	5	12 Methionine--tRNA ligase, cytoplasmic OS=Mus musculus GN=Mars PE=1 SV=1
Q9D1A2	0.006544286	18	10	15	4	0	3	0	3 Cytosolic non-specific dipeptidase OS=Mus musculus GN=Cndp2 PE=1 SV=1
Q8K4P0	0.109934488	23	20	19	0	0	0	0	5 pre-mRNA 3' end processing protein WDR33 OS=Mus musculus GN=Wdr33 PE=1 SV=1
Q61656	0.000690645	52	37	45	11	13	9	5	8 Probable ATP-dependent RNA helicase DDX5 OS=Mus musculus GN=Ddx5 PE=1 SV=2
P46061	0.003964936	24	20	13	6	2	2	0	7 Ran GTPase-activating protein 1 OS=Mus musculus GN=Rangap1 PE=1 SV=2
P20664	0.150296354	11	7	15	0	0	3	0	2 DNA primase small subunit OS=Mus musculus GN=Prim1 PE=1 SV=1
J3QN19	0.150296354	11	7	15	0	0	3	0	2 DNA primase OS=Mus musculus GN=Prim1 PE=1 SV=1
Q9D6Z1	0.10667036	20	14	19	0	0	0	0	4 Nucleolar protein 56 OS=Mus musculus GN=Nop56 PE=1 SV=2
E9Q2X6	0.008593869	12	16	20	8	0	3	0	3 Structural maintenance of chromosomes protein OS=Mus musculus GN=Smc4 PE=1 SV=1
Q8CG47	0.008593869	12	16	20	8	0	3	0	3 Structural maintenance of chromosomes protein 4 OS=Mus musculus GN=Smc4 PE=1 SV=1
P62267	0.013122411	44	34	33	6	9	3	6	7 40S ribosomal protein S23 OS=Mus musculus GN=Rps23 PE=1 SV=3
Q9D7B2	0.07697724	8	9	12	0	2	0	2	0 LIM and senescent cell antigen-like-containing domain protein OS=Mus musculus GN=Lims1 PE=1 SV=2
P11214	0.005178179	14	9	10	6	0	0	0	2 Tissue-type plasminogen activator OS=Mus musculus GN=Plat PE=1 SV=3
P08775	0.000138968	34	31	29	10	6	6	2	7 DNA-directed RNA polymerase II subunit RPB1 OS=Mus musculus GN=Polr2a PE=1 SV=3
A0A0R4J0V5	0.000138968	34	31	29	10	6	6	2	7 DNA-directed RNA polymerase subunit OS=Mus musculus GN=Polr2a PE=1 SV=1
P07742	0.027021016	116	75	78	11	10	14	0	18 Ribonucleoside-diphosphate reductase large subunit OS=Mus musculus GN=Rrm1 PE=1 SV=2
Q99JW4	0.06981691	9	9	12	0	2	0	2	0 LIM and senescent cell antigen-like-containing domain protein 1 OS=Mus musculus GN=Lims1 PE=1 SV=3
E9QP62	0.06981691	9	9	12	0	2	0	2	0 LIM and senescent cell antigen-like-containing domain protein OS=Mus musculus GN=Lims1 PE=1 SV=1
A0A0R4J005	0.06981691	9	9	12	0	2	0	2	0 LIM and senescent cell antigen-like-containing domain protein OS=Mus musculus GN=Lims1 PE=1 SV=1
P46664	0.007528771	18	15	21	8	2	5	3	2 Adenylosuccinate synthetase isozyme 2 OS=Mus musculus GN=Adss PE=1 SV=2
Q9CZU3	0.001495783	16	16	21	7	0	3	3	2 Superkiller viralicidic activity 2-like 2 OS=Mus musculus GN=Skiv2l2 PE=1 SV=1
P35550	0.018548013	19	21	11	7	0	0	3	2 rRNA 2'-O-methyltransferase fibrillarin OS=Mus musculus GN=Fbl PE=1 SV=2
F6TVP2	0.125122415	30	28	0	3	2	3	0	4 Deoxynucleoside triphosphate triphosphohydrolase SAMHD1 (Fragment) OS=Mus musculus GN=Samhd1 PE=1 SV=1
Q9R0B9	0.014968399	29	20	29	4	0	3	0	3 Procollagen-lysine,2-oxoglutarate 5-dioxygenase 2 OS=Mus musculus GN=Plod2 PE=1 SV=2
E9Q718	0.014968399	29	20	29	4	0	3	0	3 Procollagen-lysine,2-oxoglutarate 5-dioxygenase 2 OS=Mus musculus GN=Plod2 PE=1 SV=1
E9Q0K6	0.026470023	30	28	25	3	2	3	0	4 Deoxynucleoside triphosphate triphosphohydrolase SAMHD1 OS=Mus musculus GN=Samhd1 PE=1 SV=1
Q60710	0.026988023	30	28	27	3	2	3	0	4 Deoxynucleoside triphosphate triphosphohydrolase SAMHD1 OS=Mus musculus GN=Samhd1 PE=1 SV=2
F8WJE0	0.026988023	30	28	27	3	2	3	0	4 Deoxynucleoside triphosphate triphosphohydrolase SAMHD1 OS=Mus musculus GN=Samhd1 PE=1 SV=1
Q07133	0.061147267	16	14	14	0	2	0	0	0 Histone H1t OS=Mus musculus GN=Hist1h1t PE=1 SV=4
Q9EPK7	0.064804651	17	17	16	0	0	0	2	0 Exportin-7 OS=Mus musculus GN=Xpo7 PE=1 SV=3
E9PUW7	0.064804651	17	17	16	0	0	0	2	0 Exportin-7 OS=Mus musculus GN=Xpo7 PE=1 SV=1
Q8C1A5	0.002128014	67	37	57	13	5	3	0	2 Thimet oligopeptidase OS=Mus musculus GN=Thop1 PE=1 SV=1
A0A0R4IZY0	0.002128014	67	37	57	13	5	3	0	2 Thimet oligopeptidase OS=Mus musculus GN=Thop1 PE=1 SV=1
P43277	0.002618916	36	24	33	13	2	2	0	0 Histone H1.3 OS=Mus musculus GN=Hist1h1d PE=1 SV=2
P15864	0.002618916	36	24	33	13	2	2	0	0 Histone H1.2 OS=Mus musculus GN=Hist1h1c PE=1 SV=2
P43274	0.000555097	42	25	30	11	2	2	0	0 Histone H1.4 OS=Mus musculus GN=Hist1h1e PE=1 SV=2
Q9CSH3	0.001427414	40	33	49	12	0	0	0	2 Exosome complex exonuclease RRP44 OS=Mus musculus GN=Dis3 PE=1 SV=4
Q05DV1	0.000204161	24	19	25	7	0	0	0	0 NADPH--cytochrome P450 reductase OS=Mus musculus GN=Por PE=1 SV=1
A0A1B0GR11	0.000311532	24	23	22	7	0	0	0	0 Transaldolase OS=Mus musculus GN=Taldo1 PE=1 SV=1
Q93092	0.000311532	24	23	22	7	0	0	0	0 Transaldolase OS=Mus musculus GN=Taldo1 PE=1 SV=2
Q9CYN9	0.000418234	17	15	18	6	0	0	0	0 Renin receptor OS=Mus musculus GN=Atp6ap2 PE=1 SV=2
Q80W65	0.000798488	15	10	13	5	0	0	0	0 Proprotein convertase subtilisin/kexin type 9 OS=Mus musculus GN=Pcsk9 PE=1 SV=2
P37040	0.000921255	24	19	28	7	0	0	0	0 NADPH--cytochrome P450 reductase OS=Mus musculus GN=Por PE=1 SV=2
Q01320	0.001023873	63	49	49	22	0	0	0	0 DNA topoisomerase 2-alpha OS=Mus musculus GN=Top2a PE=1 SV=2
Q99P88	0.001046718	8	7	7	3	0	0	0	0 Nuclear pore complex protein Nup155 OS=Mus musculus GN=Nup155 PE=1 SV=1
Q9Z2V5	0.001224913	5	5	6	2	0	0	0	0 Histone deacetylase 6 OS=Mus musculus GN=Hdac6 PE=1 SV=3
A0A1B0GX25	0.001224913	5	5	6	2	0	0	0	0 Histone deacetylase 6 (Fragment) OS=Mus musculus GN=Hdac6 PE=1 SV=1
Q810B6	0.001268145	5	5	5	2	0	0	0	0 Rabankyrin-5 OS=Mus musculus GN=Ankfy1 PE=1 SV=2
Q9Z1T1	0.001433046	9	7	11	3	0	0	0	0 AP-3 complex subunit beta-1 OS=Mus musculus GN=Ap3b1 PE=1 SV=2
P19001	0.001570232	16	10	17	4	0	0	0	0 Keratin, type I cytoskeletal 19 OS=Mus musculus GN=Krt19 PE=1 SV=1
F8VPK5	0.001956166	11	9	8	4	0	0	0	0 Rho-associated protein kinase OS=Mus musculus GN=Rock2 PE=1 SV=1
Q61001	0.002109615	56	33	39	18	0	0	0	0 Laminin subunit alpha-5 OS=Mus musculus GN=Lama5 PE=1 SV=4
O35566	0.00294824	44	44	61	13	0	0	0	0 CD151 antigen OS=Mus musculus GN=Cd151 PE=1 SV=2
A0A1B0GRG3	0.003121584	41	42	59	13	0	0	0	0 CD151 antigen (Fragment) OS=Mus musculus GN=Cd151 PE=1 SV=1
Q8R480	0.003317181	7	4	8	2	0	0	0	0 Nuclear pore complex protein Nup85 OS=Mus musculus GN=Nup85 PE=1 SV=1
A2AW05	0.003781076	6	3	5	2	0	0	0	0 FACT complex subunit SSRP1 (Fragment) OS=Mus musculus GN=Ssrp1 PE=1 SV=1
Q08943	0.003781076	6	3	5	2	0	0	0	0 FACT complex subunit SSRP1 OS=Mus musculus GN=Ssrp1 PE=1 SV=2
P58854	0.004033403	4	5	6	2	0	0	0	0 Gamma-tubulin complex component 3 OS=Mus musculus GN=Tubgcp3 PE=1 SV=2
Q6P5D8	0.004175839	17	9	17	6	0	0	0	0 Structural maintenance of chromosomes flexible hinge domain-containing protein 1 OS=Mus musculus GN=Smchd1 PE=1 SV=2
Q9JHK4	0.004441765	10	8	7	4	0	0	0	0 Geranylgeranyl transferase type-2 subunit alpha OS=Mus musculus GN=Rabggta PE=1 SV=1
A0A0R4J233	0.005084388	8	8	13	3	0	0	0	0 Septin-10 OS=Mus musculus GN=Sept10 PE=1 SV=1
Q8C650	0.005084388	8	8	13	3	0	0	0	0 Septin-10 OS=Mus musculus GN=Sept10 PE=1 SV=1
Q9WUU7	0.005388301	11	6	8	4	0	0	0	0 Cathepsin Z OS=Mus musculus GN=Ctsz PE=1 SV=1
Q9WTZ2	0.005894615	5	3	4	2	0	0	0	0 Membrane-bound transcription factor site-1 protease OS=Mus musculus GN=Mbtps1 PE=1 SV=1
E9Q3L1	0.006679375	6	9	8	2	0	0	0	0 Unconventional myosin-VI OS=Mus musculus GN=Myo6 PE=1 SV=1
V9GXM4	0.006679375	6	9	8	2	0	0	0	0 Unconventional myosin-VI OS=Mus musculus GN=Myo6 PE=1 SV=1
V9GX76	0.006679375	6	9	8	2	0	0	0	0 Unconventional myosin-VI OS=Mus musculus GN=Myo6 PE=1 SV=1
E9PVU0	0.006679375	6	9	8	2	0	0	0	0 Unconventional myosin-VI OS=Mus musculus GN=Myo6 PE=1 SV=1
E9Q175	0.006679375	6	9	8	2	0	0	0	0 Unconventional myosin-VI OS=Mus musculus GN=Myo6 PE=1 SV=1
E9Q174	0.006679375	6	9	8	2	0	0	0	0 Unconventional myosin-VI OS=Mus musculus GN=Myo6 PE=1 SV=1
P98063	0.007098455	54	55	52	28	0	0	0	0 Bone morphogenetic protein 1 OS=Mus musculus GN=Bmp1 PE=1 SV=2
Q920Q8	0.007213972	7	4	3	2	0	0	0	0 Influenza virus NS1A-binding protein homolog OS=Mus musculus GN=Ivns1abp PE=1 SV=2
P43346	0.008144487	16	12	6	4	0	0	0	0 Deoxycytidine kinase OS=Mus musculus GN=Dck PE=1 SV=1
Q6P5B0	0.009106029	8	11	8	2	0	0	0	0 RRP12-like protein OS=Mus musculus GN=Rrp12 PE=1 SV=1
P11370	0.009215352	13	10	6	2	0	0	0	0 Retrovirus-related Env polyprotein from Fv-4 locus OS=Mus musculus GN=Fv4 PE=1 SV=2
Q8VC28	0.00990262	7	8	7	4	0	0	0	0 Aldo-keto reductase family 1 member C13 OS=Mus musculus GN=Akr1c13 PE=1 SV=2
G3X934	0.009949113	13	15	16	8	0	0	0	0 MCG115964 OS=Mus musculus GN=Wdr70 PE=1 SV=1
Q571I9	0.010633929	6	4	8	3	0	0	0	0 Aldehyde dehydrogenase family 16 member A1 OS=Mus musculus GN=Aldh16a1 PE=1 SV=2
A0A1B0GSU0	0.010633929	6	4	8	3	0	0	0	0 Aldehyde dehydrogenase family 16 member A1 OS=Mus musculus GN=Aldh16a1 PE=1 SV=1
D3Z0B9	0.010633929	6	4	8	3	0	0	0	0 Aldehyde dehydrogenase family 16 member A1 OS=Mus musculus GN=Aldh16a1 PE=1 SV=3
P43276	0.010645841	11	8	18	5	0	0	0	0 Histone H1.5 OS=Mus musculus GN=Hist1h1b PE=1 SV=2
E9PXY8	0.01072589	15	23	14	5	0	0	0	0 Ubiquitin carboxyl-terminal hydrolase 7 OS=Mus musculus GN=Usp7 PE=1 SV=1
F8VPX1	0.01072589	15	23	14	5	0	0	0	0 Ubiquitin carboxyl-terminal hydrolase 7 OS=Mus musculus GN=Usp7 PE=1 SV=1
Q6A4J8	0.01072589	15	23	14	5	0	0	0	0 Ubiquitin carboxyl-terminal hydrolase 7 OS=Mus musculus GN=Usp7 PE=1 SV=1
Q3TWF6	0.010824746	13	15	15	8	0	0	0	0 WD repeat-containing protein 70 OS=Mus musculus GN=Wdr70 PE=1 SV=1
Q6ZWQ0	0.012629768	6	7	8	4	0	0	0	0 Nesprin-2 OS=Mus musculus GN=Syne2 PE=1 SV=2
E9QP46	0.012629768	6	7	8	4	0	0	0	0 Nesprin-2 OS=Mus musculus GN=Syne2 PE=1 SV=1
Q9CXF4	0.018087725	11	8	3	2	0	0	0	0 TBC1 domain family member 15 OS=Mus musculus GN=Tbc1d15 PE=1 SV=1
O88796	0.01816149	5	4	5	3	0	0	0	0 Ribonuclease P protein subunit p30 OS=Mus musculus GN=Rpp30 PE=1 SV=1
Q923B1	0.018774733	7	10	4	3	0	0	0	0 Lariat debranching enzyme OS=Mus musculus GN=Dbr1 PE=1 SV=2
Q8C7V3	0.021181052	4	4	2	2	0	0	0	0 U3 small nucleolar RNA-associated protein 15 homolog OS=Mus musculus GN=Utp15 PE=1 SV=1
Q6PAR5	0.021295486	8	9	4	4	0	0	0	0 GTPase-activating protein and VPS9 domain-containing protein 1 OS=Mus musculus GN=Gapvd1 PE=1 SV=2
Q8JZK9	0.022526936	24	14	24	2	0	0	0	0 Hydroxymethylglutaryl-CoA synthase, cytoplasmic OS=Mus musculus GN=Hmgcs1 PE=1 SV=1
A6PWY4	0.023585826	12	11	19	9	0	0	0	0 WD repeat-containing protein 76 OS=Mus musculus GN=Wdr76 PE=1 SV=1
P10404	0.023627748	20	9	13	9	0	0	0	0 MLV-related proviral Env polyprotein OS=Mus musculus PE=1 SV=3
Q549C9	0.023862496	25	24	20	2	0	0	0	0 Cellular tumor antigen p53 OS=Mus musculus GN=Trp53 PE=1 SV=1
P02340	0.023862496	25	24	20	2	0	0	0	0 Cellular tumor antigen p53 OS=Mus musculus GN=Tp53 PE=1 SV=3
Q80ZA1	0.023862496	25	24	20	2	0	0	0	0 Cellular tumor antigen p53 OS=Mus musculus GN=Trp53 PE=1 SV=1
I7HIK9	0.023862496	25	24	20	2	0	0	0	0 Cellular tumor antigen p53 OS=Mus musculus GN=Trp53 PE=1 SV=1
Q9D1M4	0.023879296	9	7	6	5	0	0	0	0 Eukaryotic translation elongation factor 1 epsilon-1 OS=Mus musculus GN=Eef1e1 PE=1 SV=1
Q91W50	0.024268697	7	4	7	4	0	0	0	0 Cold shock domain-containing protein E1 OS=Mus musculus GN=Csde1 PE=1 SV=1
Q3TY56	0.02835207	5	5	3	3	0	0	0	0 Eukaryotic translation initiation factor 3 subunit K OS=Mus musculus GN=Eif3k PE=1 SV=1
Q4VA53	0.03394996	18	4	14	2	0	0	0	0 Sister chromatid cohesion protein PDS5 homolog B OS=Mus musculus GN=Pds5b PE=1 SV=1
F8WHU5	0.03394996	18	4	14	2	0	0	0	0 Sister chromatid cohesion protein PDS5 homolog B OS=Mus musculus GN=Pds5b PE=1 SV=1
Q99JX4	0.034797772	7	9	2	3	0	0	0	0 Eukaryotic translation initiation factor 3 subunit M OS=Mus musculus GN=Eif3m PE=1 SV=1
O35130	0.040393757	3	3	7	3	0	0	0	0 Ribosomal RNA small subunit methyltransferase NEP1 OS=Mus musculus GN=Emg1 PE=1 SV=1
P70336	0.058329407	9	7	7	0	0	0	0	0 Rho-associated protein kinase 2 OS=Mus musculus GN=Rock2 PE=1 SV=1
Q9ERU9	0.058426329	6	4	5	0	0	0	0	0 E3 SUMO-protein ligase RanBP2 OS=Mus musculus GN=Ranbp2 PE=1 SV=2
F6SDD7	0.058501493	4	3	3	0	0	0	0	0 DNA topoisomerase (Fragment) OS=Mus musculus GN=Top3b PE=1 SV=1

O88792	0.058501493	4	3	3	0	0	0	0	0	Junctional adhesion molecule A OS=Mus musculus GN=F11r PE=1 SV=2
A2RTH5	0.058501493	4	3	3	0	0	0	0	0	Leucine carboxyl methyltransferase 1 OS=Mus musculus GN=Lcmt1 PE=1 SV=1
A0A0U1RNF2	0.058501493	4	3	3	0	0	0	0	0	Protein Lcmt1 OS=Mus musculus GN=Lcmt1 PE=1 SV=1
Q6PAV2	0.059002466	7	5	5	0	0	0	0	0	Probable E3 ubiquitin-protein ligase HERC4 OS=Mus musculus GN=Herc4 PE=1 SV=2
P17563	0.059095581	15	13	15	0	0	0	0	0	Selenium-binding protein 1 OS=Mus musculus GN=Selenbp1 PE=1 SV=2
Q921D0	0.059504973	4	3	4	0	0	0	0	0	Annexin OS=Mus musculus GN=Anxa8 PE=1 SV=1
Q9Z321	0.059504973	4	3	4	0	0	0	0	0	DNA topoisomerase 3-beta-1 OS=Mus musculus GN=Top3b PE=1 SV=1
Q8BFV2	0.060063403	5	3	4	0	0	0	0	0	PCI domain-containing protein 2 OS=Mus musculus GN=Pcid2 PE=1 SV=1
Q80Y81	0.060063403	5	3	4	0	0	0	0	0	Zinc phosphodiesterase ELAC protein 2 OS=Mus musculus GN=Elac2 PE=1 SV=1
B1ATP7	0.060063403	5	3	4	0	0	0	0	0	Zinc phosphodiesterase ELAC protein 2 OS=Mus musculus GN=Elac2 PE=1 SV=1
Q3UKJ7	0.060118731	9	7	0	2	0	0	0	0	WD40 repeat-containing protein SMU1 OS=Mus musculus GN=Smu1 PE=2 SV=2
G3UWR8	0.060491654	2	13	7	5	0	0	0	0	Ubiquitin carboxyl-terminal hydrolase 7 (Fragment) OS=Mus musculus GN=Usp7 PE=1 SV=1
Q63836	0.060537538	14	13	15	0	0	0	0	0	Selenium-binding protein 2 OS=Mus musculus GN=Selenbp2 PE=1 SV=2
Q64511	0.060601494	10	9	8	0	0	0	0	0	DNA topoisomerase 2-beta OS=Mus musculus GN=Top2b PE=1 SV=2
P39061	0.060871826	15	9	13	0	0	0	0	0	Collagen alpha-1(XVIII) chain OS=Mus musculus GN=Col18a1 PE=1 SV=4
E9QPX1	0.061071315	12	7	10	0	0	0	0	0	Collagen alpha-1(XVIII) chain OS=Mus musculus GN=Col18a1 PE=1 SV=1
Q9CXK8	0.061273139	7	4	5	0	0	0	0	0	60S ribosome subunit biogenesis protein NIP7 homolog OS=Mus musculus GN=Nip7 PE=1 SV=1
D3YU58	0.061273139	7	4	5	0	0	0	0	0	Anaphase-promoting complex subunit 7 OS=Mus musculus GN=Anapc7 PE=1 SV=1
Q9WVM3	0.061273139	7	4	5	0	0	0	0	0	Anaphase-promoting complex subunit 7 OS=Mus musculus GN=Anapc7 PE=1 SV=3
O08663	0.061340757	5	5	5	0	0	0	0	0	Methionine aminopeptidase 2 OS=Mus musculus GN=Metap2 PE=1 SV=1
H9KV04	0.061340757	9	9	9	0	0	0	0	0	Mini-chromosome maintenance complex-binding protein OS=Mus musculus GN=Mcmbp PE=1 SV=1
Q3UI33	0.061340757	5	5	5	0	0	0	0	0	Methionine aminopeptidase 2 OS=Mus musculus GN=Metap2 PE=1 SV=1
Q3TMN1	0.061340757	5	5	5	0	0	0	0	0	Methionine aminopeptidase 2 OS=Mus musculus GN=Metap2 PE=1 SV=1
O54692	0.061340757	3	3	3	0	0	0	0	0	Centromere/kinetochore protein zw10 homolog OS=Mus musculus GN=Zw10 PE=1 SV=3
Q8VDP4	0.061340757	3	3	3	0	0	0	0	0	Cell cycle and apoptosis regulator protein 2 OS=Mus musculus GN=Ccar2 PE=1 SV=2
Q80YV3	0.061340757	2	2	2	0	0	0	0	0	Transformation/transcription domain-associated protein OS=Mus musculus GN=Trrap PE=1 SV=2
Q8CGY8	0.061340757	8	8	8	0	0	0	0	0	UDP-N-acetylglucosamine--peptide N-acetylglucosaminyltransferase 110 kDa subunit OS=Mus musculus GN=Ogt PE=1 SV=2
Q9D2U9	0.061488652	180	155	203	0	0	0	0	0	Histone H2B type 3-A OS=Mus musculus GN=Hist3h2ba PE=1 SV=3
Q8BM72	0.06157035	6	4	6	0	0	0	0	0	Heat shock 70 kDa protein 13 OS=Mus musculus GN=Hspa13 PE=1 SV=1
Q8R3C0	0.061984728	9	9	10	0	0	0	0	0	Mini-chromosome maintenance complex-binding protein OS=Mus musculus GN=Mcmbp PE=1 SV=1
A0A0R4J190	0.062049877	8	5	5	0	0	0	0	0	ADP-ribosyl cyclase/cyclic ADP-ribose hydrolase 2 OS=Mus musculus GN=Bst1 PE=1 SV=1
Q3TH01	0.063884307	22	18	14	0	0	0	0	0	H-2 class I histocompatibility antigen, K-K alpha chain OS=Mus musculus GN=H2-K1 PE=1 SV=1
P33610	0.064497923	4	4	5	0	0	0	0	0	DNA primase large subunit OS=Mus musculus GN=Prim2 PE=1 SV=1
E9Q5E2	0.064539319	5	3	5	0	0	0	0	0	Protein BC005561 OS=Mus musculus GN=BC005561 PE=1 SV=1
Q61738	0.06460263	5	4	6	0	0	0	0	0	Integrin alpha-7 OS=Mus musculus GN=Itga7 PE=1 SV=3
P33609	0.065027744	4	2	3	0	0	0	0	0	DNA polymerase alpha catalytic subunit OS=Mus musculus GN=Pola1 PE=1 SV=2
P97377	0.065122296	10	5	7	0	0	0	0	0	Cyclin-dependent kinase 2 OS=Mus musculus GN=Cdk2 PE=1 SV=2
Q8R5F3	0.065209612	8	9	0	3	0	0	0	0	O-acetyl-ADP-ribose deacetylase 1 OS=Mus musculus GN=Oard1 PE=1 SV=2
A0A0U1RPE7	0.065348584	5	4	3	0	0	0	0	0	Hsp70-binding protein 1 (Fragment) OS=Mus musculus GN=Hspbp1 PE=1 SV=1
A0A0U1RPF2	0.065348584	5	4	3	0	0	0	0	0	Hsp70-binding protein 1 (Fragment) OS=Mus musculus GN=Hspbp1 PE=1 SV=1
Q99P31	0.065348584	5	4	3	0	0	0	0	0	Hsp70-binding protein 1 OS=Mus musculus GN=Hspbp1 PE=1 SV=1
O35640	0.065540635	6	3	4	0	0	0	0	0	Annexin A8 OS=Mus musculus GN=Anxa8 PE=1 SV=2
Q810D6	0.066088509	6	3	5	0	0	0	0	0	Glutamate-rich WD repeat-containing protein 1 OS=Mus musculus GN=Grwd1 PE=1 SV=2
E9Q906	0.066824035	0	5	4	2	0	0	0	0	Catenin delta-1 OS=Mus musculus GN=Ctnnd1 PE=1 SV=1
Q8K2B3	0.067282364	5	6	6	0	0	0	0	0	Succinate dehydrogenase [ubiquinone] flavoprotein subunit, mitochondrial OS=Mus musculus GN=Sdha PE=1 SV=1
Q8BGZ4	0.067758005	7	7	5	0	0	0	0	0	Cell division cycle protein 23 homolog OS=Mus musculus GN=Cdc23 PE=1 SV=2
A0A0R4J1W7	0.067758005	7	7	5	0	0	0	0	0	CDC23 (Cell division cycle 23, yeast, homolog), isoform CRA_c OS=Mus musculus GN=Cdc23 PE=1 SV=1
G3X8W7	0.067758005	7	7	5	0	0	0	0	0	CDC23 (Cell division cycle 23, yeast, homolog), isoform CRA_a OS=Mus musculus GN=Cdc23 PE=1 SV=1
Q9CRT8	0.068235596	13	6	10	0	0	0	0	0	Exportin-T OS=Mus musculus GN=Xpot PE=1 SV=3
Q2NL51	0.068476125	5	6	5	0	0	0	0	0	Glycogen synthase kinase-3 alpha OS=Mus musculus GN=Gsk3a PE=1 SV=2
A0A0R4J006	0.068778285	0	4	4	2	0	0	0	0	Na(+)/H(+) exchange regulatory cofactor NHE-RF2 OS=Mus musculus GN=Slc9a3r2 PE=1 SV=1
P29351	0.068943432	4	5	5	0	0	0	0	0	Tyrosine-protein phosphatase non-receptor type 6 OS=Mus musculus GN=Ptpn6 PE=1 SV=2
Q99JN2	0.069541267	8	5	4	0	0	0	0	0	Kelch-like protein 22 OS=Mus musculus GN=Klhl22 PE=1 SV=1
H9KV05	0.069541267	8	5	4	0	0	0	0	0	Kelch-like protein 22 OS=Mus musculus GN=Klhl22 PE=1 SV=1
A0A1B0GSX7	0.070340683	3	3	2	0	0	0	0	0	Nuclear pore complex protein Nup98-Nup96 OS=Mus musculus GN=Nup98 PE=1 SV=1
Q64378	0.070765139	4	5	4	0	0	0	0	0	Peptidyl-prolyl cis-trans isomerase FKBP5 OS=Mus musculus GN=Fkbp5 PE=1 SV=1
A0A1B0GR08	0.071671625	4	2	4	0	0	0	0	0	Gamma-tubulin complex component (Fragment) OS=Mus musculus GN=Tubgcp2 PE=1 SV=1
Q921G8	0.071671625	4	2	4	0	0	0	0	0	Gamma-tubulin complex component 2 OS=Mus musculus GN=Tubgcp2 PE=1 SV=2
A0A087WNS9	0.07199381	6	8	7	0	0	0	0	0	Lymphocyte antigen 6E (Fragment) OS=Mus musculus GN=Ly6e PE=1 SV=6
P14602	0.07222639	18	14	9	0	0	0	0	0	Heat shock protein beta-1 OS=Mus musculus GN=Hspb1 PE=1 SV=3
contaminant_TRYPSIN	0.072429565	4	4	6	0	0	0	0	0	no description
Q505N6	0.072671706	9	4	8	0	0	0	0	0	Ribosomal protein S6 kinase OS=Mus musculus GN=Rps6ka1 PE=1 SV=1
F6Q8A4	0.072671706	9	4	8	0	0	0	0	0	Ribosomal protein S6 kinase alpha-1 (Fragment) OS=Mus musculus GN=Rps6ka1 PE=1 SV=1
E9PWV3	0.072671706	9	4	8	0	0	0	0	0	Ribosomal protein S6 kinase OS=Mus musculus GN=Rps6ka1 PE=1 SV=1
F6RQA2	0.072671706	9	4	8	0	0	0	0	0	Ribosomal protein S6 kinase OS=Mus musculus GN=Rps6ka1 PE=1 SV=1
P18653	0.072671706	9	4	8	0	0	0	0	0	Ribosomal protein S6 kinase alpha-1 OS=Mus musculus GN=Rps6ka1 PE=1 SV=1
G3UZI3	0.072671706	9	4	8	0	0	0	0	0	Ribosomal protein S6 kinase alpha-1 OS=Mus musculus GN=Rps6ka1 PE=1 SV=1
O09159	0.073037536	4	2	2	0	0	0	0	0	Lysosomal alpha-mannosidase OS=Mus musculus GN=Man2b1 PE=1 SV=4
Q9WV54	0.073037536	4	2	2	0	0	0	0	0	Acid ceramidase OS=Mus musculus GN=Asah1 PE=1 SV=1
G3X9Q1	0.073226064	5	4	7	0	0	0	0	0	Integrin alpha 7 OS=Mus musculus GN=Itga7 PE=1 SV=1
Q8R2U0	0.073375642	7	3	6	0	0	0	0	0	Nucleoporin SEH1 OS=Mus musculus GN=Seh1l PE=2 SV=1
B1AZI6	0.073639414	5	3	6	0	0	0	0	0	THO complex subunit 2 OS=Mus musculus GN=Thoc2 PE=1 SV=1
Q91YP2	0.074507005	6	5	3	0	0	0	0	0	Neurolysin, mitochondrial OS=Mus musculus GN=Nln PE=1 SV=1
P83887	0.07475414	12	5	7	0	0	0	0	0	Tubulin gamma-1 chain OS=Mus musculus GN=Tubg1 PE=1 SV=1
E9PWH6	0.074889414	5	5	3	0	0	0	0	0	HEAT repeat-containing protein 3 OS=Mus musculus GN=Heatr3 PE=1 SV=1
Q9JIK5	0.074907086	6	8	6	0	0	0	0	0	Nucleolar RNA helicase 2 OS=Mus musculus GN=Ddx21 PE=1 SV=3
Q9WVM1	0.074907086	3	4	3	0	0	0	0	0	Rac GTPase-activating protein 1 OS=Mus musculus GN=Racgap1 PE=1 SV=1
Q99LB6	0.074907086	6	8	6	0	0	0	0	0	Methionine adenosyltransferase 2 subunit beta OS=Mus musculus GN=Mat2b PE=1 SV=1
Q8C4B4	0.075959594	5	2	3	0	0	0	0	0	Protein unc-119 homolog B OS=Mus musculus GN=Unc119b PE=1 SV=1
Q3UW53	0.07638058	10	14	16	0	0	0	0	0	Protein Niban OS=Mus musculus GN=Fam129a PE=1 SV=2
Q91XR9	0.0764554	3	2	4	0	0	0	0	0	Phospholipid hydroperoxide glutathione peroxidase, nuclear OS=Mus musculus GN=Gpx4 PE=1 SV=3
Q9Z204	0.0764554	3	2	4	0	0	0	0	0	Heterogeneous nuclear ribonucleoproteins C1/C2 OS=Mus musculus GN=Hnrnpc PE=1 SV=1
Q9D0I8	0.077041604	5	3	0	2	0	0	0	0	mRNA turnover protein 4 homolog OS=Mus musculus GN=Mrto4 PE=1 SV=1
A2AMV1	0.077041604	5	3	0	2	0	0	0	0	RIKEN cDNA 2610012O22, isoform CRA_a OS=Mus musculus GN=Mrto4 PE=1 SV=1
Q5SU71	0.077782126	7	5	3	0	0	0	0	0	E3 ubiquitin/ISG15 ligase TRIM25 OS=Mus musculus GN=Trim25 PE=1 SV=1
Q61510	0.077782126	7	5	3	0	0	0	0	0	E3 ubiquitin/ISG15 ligase TRIM25 OS=Mus musculus GN=Trim25 PE=1 SV=2
Q8K298	0.078460951	5	7	5	0	0	0	0	0	Anillin OS=Mus musculus GN=Anln PE=1 SV=2
P62307	0.079333295	3	3	5	0	0	0	0	0	Small nuclear ribonucleoprotein F OS=Mus musculus GN=Snrpf PE=1 SV=1
Q99JA5	0.079374068	6	9	7	0	0	0	0	0	Ly6e protein OS=Mus musculus GN=Ly6e PE=1 SV=1
A0A087WRZ2	0.079374068	6	9	7	0	0	0	0	0	Lymphocyte antigen 6E (Fragment) OS=Mus musculus GN=Ly6e PE=1 SV=1
Q64253	0.079374068	6	9	7	0	0	0	0	0	Lymphocyte antigen 6E OS=Mus musculus GN=Ly6e PE=1 SV=2
A0A087WNT2	0.079374068	6	9	7	0	0	0	0	0	Lymphocyte antigen 6E (Fragment) OS=Mus musculus GN=Ly6e PE=1 SV=6
A0A087WPY4	0.079374068	6	9	7	0	0	0	0	0	Lymphocyte antigen 6E (Fragment) OS=Mus musculus GN=Ly6e PE=1 SV=1
A0A087WQ65	0.079374068	6	9	7	0	0	0	0	0	Lymphocyte antigen 6E (Fragment) OS=Mus musculus GN=Ly6e PE=1 SV=1
Q99JF5	0.080797769	4	3	6	0	0	0	0	0	Diphosphomevalonate decarboxylase OS=Mus musculus GN=Mvd PE=1 SV=2
Q91W96	0.081193273	4	4	3	4	0	0	0	0	Anaphase-promoting complex subunit 4 OS=Mus musculus GN=Anapc4 PE=1 SV=1
P54276	0.082748624	5	0	3	2	0	0	0	0	DNA mismatch repair protein Msh6 OS=Mus musculus GN=Msh6 PE=1 SV=3
Q6A026	0.083679833	12	4	8	0	0	0	0	0	Sister chromatid cohesion protein PDS5 homolog A OS=Mus musculus GN=Pds5a PE=1 SV=3
E9QPI5	0.083679833	12	4	8	0	0	0	0	0	Sister chromatid cohesion protein PDS5 homolog A OS=Mus musculus GN=Pds5a PE=1 SV=1
P59328	0.084095285	2	3	2	0	0	0	0	0	WD repeat and HMG-box DNA-binding protein 1 OS=Mus musculus GN=Wdhd1 PE=1 SV=2
A0A0R4J1F8	0.084095285	2	3	2	0	0	0	0	0	WD repeat and HMG-box DNA-binding protein 1 OS=Mus musculus GN=Wdhd1 PE=1 SV=1
E9QPB3	0.084095285	2	3	2	0	0	0	0	0	WD repeat and HMG-box DNA-binding protein 1 OS=Mus musculus GN=Wdhd1 PE=1 SV=1
E9Q9M5	0.085057279	7	3	3	0	0	0	0	0	Ubiquitin carboxyl-terminal hydrolase 19 OS=Mus musculus GN=Usp19 PE=1 SV=1
J3KMM1	0.085057279	7	3	3	0	0	0	0	0	Ubiquitin carboxyl-terminal hydrolase 19 OS=Mus musculus GN=Usp19 PE=1 SV=1
A0A0A6YWN9	0.085057279	7	3	3	0	0	0	0	0	Ubiquitin carboxyl-terminal hydrolase 19 (Fragment) OS=Mus musculus GN=Usp19 PE=1 SV=1
A0A0A6YWX1	0.085057279	7	3	3	0	0	0	0	0	Ubiquitin carboxyl-terminal hydrolase 19 OS=Mus musculus GN=Usp19 PE=1 SV=1
Q3UJD6	0.085057279	7	3	3	0	0	0	0	0	Ubiquitin carboxyl-terminal hydrolase 19 OS=Mus musculus GN=Usp19 PE=1 SV=1
V9GX06	0.086113781	2	3	4	0	0	0	0	0	Protein Gm11214 (Fragment) OS=Mus musculus GN=Gm11214 PE=1 SV=1
Q99LE6	0.086172937	6	4	9	0	0	0	0	0	ATP-binding cassette sub-family F member 2 OS=Mus musculus GN=Abcf2 PE=1 SV=1
Q8VCT3	0.086247576	11	5	13	0	0	0	0	0	Aminopeptidase B OS=Mus musculus GN=Rnpep PE=1 SV=2
E9PYF1	0.086247576	11	5	13	0	0	0	0	0	Aminopeptidase B OS=Mus musculus GN=Rnpep PE=1 SV=1
Q5SYD0	0.088877496	5	2	4	4	0	0	0	0	Unconventional myosin-Id OS=Mus musculus GN=Myo1d PE=1 SV=1
Q61805	0.092769043	6	2	6	0	0	0	0	0	Lipopolysaccharide-binding protein OS=Mus musculus GN=Lbp PE=1 SV=2
Q64324	0.092972088	2	4	4	0	0	0	0	0	Syntaxin-binding protein 2 OS=Mus musculus GN=Stxbp2 PE=1 SV=1
F8WGM5	0.092972088	2	4	4	0	0	0	0	0	Syntaxin-binding protein 2 (Fragment) OS=Mus musculus GN=Stxbp2 PE=1 SV=1
Q07832	0.09313315	6	3	2	0	0	0	0	0	Serine/threonine-protein kinase PLK1 OS=Mus musculus GN=Plk1 PE=1 SV=2
Q9WVG5	0.093509353	3	6	5	0	0	0	0	0	Endothelial lipase OS=Mus musculus GN=Lipg PE=2 SV=3
G3X8X0	0.093582308	9	7	3	0	0	0	0	0	DEAH (Asp-Glu-Ala-His) box polypeptide 16, isoform CRA_a OS=Mus musculus GN=Dhx16 PE=1 SV=1
Q06890	0.094230641	14	16	7	0	0	0	0	0	Clusterin OS=Mus musculus GN=Clu PE=1 SV=1
O70325	0.094533848	5	2	6	0	0	0	0	0	Phospholipid hydroperoxide glutathione peroxidase, mitochondrial OS=Mus musculus GN=Gpx4 PE=1 SV=4
F7A8H6	0.094533848	5	2	6	0	0	0	0	0	Glutathione peroxidase OS=Mus musculus GN=Gpx4 PE=1 SV=1
S4R1E5	0.094533848	5	2	6	0	0	0	0	0	Glutathione peroxidase OS=Mus musculus GN=Gpx4 PE=1 SV=1
Q9Z0S1	0.095176456	2	2	4	0	0	0	0	0	3'(2'),5'-bisphosphate nucleotidase 1 OS=Mus musculus GN=Bpnt1 PE=1 SV=2
Q3UW83	0.095176456	2	2	4	0	0	0	0	0	40S ribosomal protein S10 OS=Mus musculus GN=Rps10 PE=1 SV=1
Q6A0A9	0.095176456	3	3	6	0	0	0	0	0	Constitutive coactivator of PPAR-gamma-like protein 1 OS=Mus musculus GN=FAM120A PE=1 SV=2
A0A1B0GRV0	0.095176456	2	2	4	0	0	0	0	0	3'(2'),5'-bisphosphate nucleotidase 1 OS=Mus musculus GN=Bpnt1 PE=1 SV=1
P63325	0.095176456	2	2	4	0	0	0	0	0	40S ribosomal protein S10 OS=Mus musculus GN=Rps10 PE=1 SV=1
E9Q5G3	0.095795094	2	4	3	0	0	0	0	0	Kinesin-like protein KIF23 OS=Mus musculus GN=Kif23 PE=1 SV=1
Q99KD5	0.095795094	2	4	3	0	0	0	0	0	Protein unc-45 homolog A OS=Mus musculus GN=Unc45a PE=1 SV=2
O89023	0.099193118	7	4	2	0	0	0	0	0	Tripeptidyl-peptidase 1 OS=Mus musculus GN=Tpp1 PE=1 SV=2
Q9D554	0.099812647	3	6	4	0	0	0	0	0	Splicing factor 3A subunit 3 OS=Mus musculus GN=Sf3a3 PE=1 SV=2
Q8K2Q7	0.100361789	0	2	4	2	0	0	0	0	BRO1 domain-containing protein BROX OS=Mus musculus GN=Brox PE=1 SV=1
P97792	0.10202611	4	2	6	0	0	0	0	0	Coxsackievirus and adenovirus receptor homolog OS=Mus musculus GN=Cxadr PE=1 SV=1
P70261	0.102623212	4	5	0	3	0	0	0	0	Paladin OS=Mus musculus GN=Pald1 PE=1 SV=1
A0A0R4J007	0.102623212	4	5	0	3	0	0	0	0	Paladin OS=Mus musculus GN=Pald1 PE=1 SV=1
Q9R0M4	0.102982561	8	2	7	0	0	0	0	0	Podocalyxin OS=Mus musculus GN=Podxl PE=1 SV=2
Q9JHL1	0.103492551	0	4	4	3	0	0	0	0	Na(+)/H(+) exchange regulatory cofactor NHE-RF2 OS=Mus musculus GN=Slc9a3r2 PE=1 SV=2
A0A0R4IZX2	0.103492551	0	4	4	3	0	0	0	0	Na(+)/H(+) exchange regulatory cofactor NHE-RF OS=Mus musculus GN=Slc9a3r2 PE=1 SV=1
Q80TP3	0.103910301	0	4	2	2	0	0	0	0	E3 ubiquitin-protein ligase UBR5 OS=Mus musculus GN=Ubr5 PE=1 SV=2
E9Q2H1	0.103910301	0	4	2	2	0	0	0	0	E3 ubiquitin-protein ligase UBR5 OS=Mus musculus GN=Ubr5 PE=1 SV=1
M9MMK0	0.110354948	4	3	8	0	0	0	0	0	Semaphorin-3B OS=Mus musculus GN=Sema3b PE=1 SV=1
Q62177	0.110354948	4	3	8	0	0	0	0	0	Semaphorin-3B OS=Mus musculus GN=Sema3b PE=2 SV=2
P43275	0.11392901	10	2	6	0	0	0	0	0	Histone H1.1 OS=Mus musculus GN=Hist1h1a PE=1 SV=2
G3UXW9	0.11449028	2	4	2	0	0	0	0	0	COP9 signalosome complex subunit 1 OS=Mus musculus GN=Gps1 PE=1 SV=1
F7CGG2	0.11449028	2	4	2	0	0	0	0	0	Transformation/transcription domain-associated protein (Fragment) OS=Mus musculus GN=Trrap PE=1 SV=1
E9QLK7	0.11449028	2	4	2	0	0	0	0	0	Transformation/transcription domain-associated protein OS=Mus musculus GN=Trrap PE=1 SV=1
E9PZA7	0.11449028	2	4	2	0	0	0	0	0	Transformation/transcription domain-associated protein OS=Mus musculus GN=Trrap PE=1 SV=1
A0A1D5RLL4	0.11449028	2	4	2	0	0	0	0	0	Transformation/transcription domain-associated protein OS=Mus musculus GN=Trrap PE=1 SV=1
E9PWT1	0.11449028	2	4	2	0	0	0	0	0	Transformation/transcription domain-associated protein OS=Mus musculus GN=Trrap PE=1 SV=1
A0A140LJB7	0.11449028	2	4	2	0	0	0	0	0	COP9 signalosome complex subunit 1 OS=Mus musculus GN=Gps1 PE=1 SV=1
Q99LD4	0.11449028	2	4	2	0	0	0	0	0	COP9 signalosome complex subunit 1 OS=Mus musculus GN=Gps1 PE=1 SV=1
B1ATU4	0.11449028	2	4	2	0	0	0	0	0	COP9 signalosome complex subunit 1 OS=Mus musculus GN=Gps1 PE=1 SV=1
Q3MIA8	0.11449028	2	4	2	0	0	0	0	0	COP9 signalosome complex subunit 1 OS=Mus musculus GN=Gps1 PE=1 SV=1
E9PWK1	0.115370201	5	11	6	0	0	0	0	0	Epoxide hydrolase OS=Mus musculus GN=Ephx1 PE=1 SV=1
Q9D379	0.115370201	5	11	6	0	0	0	0	0	Epoxide hydrolase 1 OS=Mus musculus GN=Ephx1 PE=1 SV=2
P23927	0.116277245	6	18	15	0	0	0	0	0	Alpha-crystallin B chain OS=Mus musculus GN=Cryab PE=1 SV=2
Q9CRA5	0.119352591	4	2	7	0	0	0	0	0	Golgi phosphoprotein 3 OS=Mus musculus GN=Golph3 PE=1 SV=1
Q9DCE5	0.125054635	6	3	11	0	0	0	0	0	p21-activated protein kinase-interacting protein 1 OS=Mus musculus GN=Pak1ip1 PE=1 SV=2
P57784	0.137349757	9	3	2	0	0	0	0	0	U2 small nuclear ribonucleoprotein A' OS=Mus musculus GN=Snrpa1 PE=1 SV=2
Q8R0G9	0.137835159	3	2	7	0	0	0	0	0	Nuclear pore complex protein Nup133 OS=Mus musculus GN=Nup133 PE=1 SV=2
E9Q8N1	0.145809642	0	8	4	6	0	0	0	0	Titin OS=Mus musculus GN=Ttn PE=1 SV=1
A3KG93	0.151173659	2	2	0	2	0	0	0	0	Lysine-specific histone demethylase 1A OS=Mus musculus GN=Kdm1a PE=1 SV=1
Q6ZQ88	0.151173659	2	2	0	2	0	0	0	0	Lysine-specific histone demethylase 1A OS=Mus musculus GN=Kdm1a PE=1 SV=2
Q05A75	0.181690482	4	3	0	0	0	0	0	0	H-2 class I histocompatibility antigen, TLA(B) alpha chain OS=Mus musculus GN=H2-T3 PE=1 SV=1
P14432	0.181690482	4	3	0	0	0	0	0	0	H-2 class I histocompatibility antigen, TLA(B) alpha chain OS=Mus musculus GN=H2-T3 PE=1 SV=2

Q6IFZ8	0.181704484	7	0	6	0	0	0	0	0	MCG1050941 OS=Mus musculus GN=Gm5414 PE=1 SV=1
A0A0R4J135	0.181740479	0	13	15	0	0	0	0	0	Selenium-binding protein 2 OS=Mus musculus GN=Selenbp2 PE=1 SV=1
D6RJ62	0.181887376	9	0	8	0	0	0	0	0	Uridine 5'-monophosphate synthase OS=Mus musculus GN=Umps PE=1 SV=1
D3Z132	0.181890642	0	0	6	2	0	0	0	0	THO complex subunit 6 homolog OS=Mus musculus GN=Thoc6 PE=1 SV=1
D3Z131	0.181890642	0	0	6	2	0	0	0	0	THO complex subunit 6 homolog OS=Mus musculus GN=Thoc6 PE=1 SV=1
Q5U4D9	0.181890642	0	0	6	2	0	0	0	0	THO complex subunit 6 homolog OS=Mus musculus GN=Thoc6 PE=1 SV=1
P24860	0.181896468	7	5	0	0	0	0	0	0	G2/mitotic-specific cyclin-B1 OS=Mus musculus GN=Ccnb1 PE=1 SV=3
P35822	0.181999486	5	4	0	0	0	0	0	0	Receptor-type tyrosine-protein phosphatase kappa OS=Mus musculus GN=Ptprk PE=1 SV=1
O55106	0.182019553	0	5	6	0	0	0	0	0	Striatin OS=Mus musculus GN=Strn PE=1 SV=2
F8WH41	0.182019553	0	5	6	0	0	0	0	0	Striatin OS=Mus musculus GN=Strn PE=1 SV=1
Q8R5H1	0.18253515	6	5	0	0	0	0	0	0	Ubiquitin carboxyl-terminal hydrolase 15 OS=Mus musculus GN=Usp15 PE=1 SV=1
Q8C547	0.18253515	6	5	0	0	0	0	0	0	HEAT repeat-containing protein 5B OS=Mus musculus GN=Heatr5b PE=1 SV=3
Q9CPX6	0.182568009	0	4	5	0	0	0	0	0	Ubiquitin-like-conjugating enzyme ATG3 OS=Mus musculus GN=Atg3 PE=1 SV=1
Q78JE5	0.182792568	0	2	2	0	0	0	0	0	F-box only protein 22 OS=Mus musculus GN=Fbxo22 PE=1 SV=2
Q9D753	0.182792568	0	2	2	0	0	0	0	0	Exosome complex component RRP43 OS=Mus musculus GN=Exosc8 PE=1 SV=1
D3YYN3	0.182792568	0	2	2	0	0	0	0	0	Exosome complex component RRP43 (Fragment) OS=Mus musculus GN=Exosc8 PE=1 SV=1
E9PWW8	0.182792568	0	2	2	0	0	0	0	0	Histone demethylase UTY OS=Mus musculus GN=Uty PE=4 SV=1
P79457	0.182792568	0	2	2	0	0	0	0	0	Histone demethylase UTY OS=Mus musculus GN=Uty PE=1 SV=2
F6SGT4	0.182792568	0	2	2	0	0	0	0	0	Exosome complex component RRP43 (Fragment) OS=Mus musculus GN=Exosc8 PE=1 SV=1
G3X9F2	0.182792568	0	2	2	0	0	0	0	0	Histone demethylase UTY OS=Mus musculus GN=Uty PE=4 SV=1
Q61468	0.182830001	3	2	0	0	0	0	0	0	Mesothelin OS=Mus musculus GN=Msln PE=1 SV=1
P56546	0.182830001	3	2	0	0	0	0	0	0	C-terminal-binding protein 2 OS=Mus musculus GN=Ctbp2 PE=1 SV=2
Q91YZ2	0.182830001	3	2	0	0	0	0	0	0	C-terminal binding protein 2, isoform CRA_b OS=Mus musculus GN=Ctbp2 PE=1 SV=1
A2A864	0.182830001	3	2	0	0	0	0	0	0	Integrin beta OS=Mus musculus GN=Itgb4 PE=1 SV=1
E0CYH4	0.182830001	3	2	0	0	0	0	0	0	WD repeat-containing protein 26 OS=Mus musculus GN=Wdr26 PE=1 SV=1
E9PWQ9	0.182830001	3	2	0	0	0	0	0	0	C-terminal-binding protein 2 (Fragment) OS=Mus musculus GN=Ctbp2 PE=1 SV=1
E9Q0T4	0.182830001	3	2	0	0	0	0	0	0	C-terminal-binding protein 2 (Fragment) OS=Mus musculus GN=Ctbp2 PE=1 SV=8
Q8R323	0.182830001	3	2	0	0	0	0	0	0	Replication factor C subunit 3 OS=Mus musculus GN=Rfc3 PE=1 SV=1
Q8C6G8	0.182830001	3	2	0	0	0	0	0	0	WD repeat-containing protein 26 OS=Mus musculus GN=Wdr26 PE=1 SV=3
Q80WQ2	0.182830001	3	2	0	0	0	0	0	0	Protein VAC14 homolog OS=Mus musculus GN=Vac14 PE=1 SV=1
Q3TIU4	0.182832992	4	0	3	0	0	0	0	0	2',5'-phosphodiesterase 12 OS=Mus musculus GN=Pde12 PE=1 SV=2
Q922E4	0.182832992	4	0	3	0	0	0	0	0	Ethanolamine-phosphate cytidylyltransferase OS=Mus musculus GN=Pcyt2 PE=1 SV=1
Q6PD03	0.182832992	4	0	3	0	0	0	0	0	Serine/threonine-protein phosphatase 2A 56 kDa regulatory subunit alpha isoform OS=Mus musculus GN=Ppp2r5a PE=1 SV=1
A2ABY3	0.182832992	4	0	3	0	0	0	0	0	Ethanolamine-phosphate cytidylyltransferase OS=Mus musculus GN=Pcyt2 PE=1 SV=1
Q3U7R1	0.182832992	4	0	3	0	0	0	0	0	Extended synaptotagmin-1 OS=Mus musculus GN=Esyt1 PE=1 SV=2
Q80X41	0.182832992	4	0	3	0	0	0	0	0	Serine/threonine-protein kinase VRK1 OS=Mus musculus GN=Vrk1 PE=1 SV=2
Q8CEC6	0.183906964	3	0	3	0	0	0	0	0	Peptidylprolyl isomerase domain and WD repeat-containing protein 1 OS=Mus musculus GN=Ppwd1 PE=1 SV=2
D6RFB8	0.183906964	3	0	3	0	0	0	0	0	DNA polymerase OS=Mus musculus GN=Pold1 PE=1 SV=1
D3Z667	0.183906964	2	0	2	0	0	0	0	0	Dynein heavy chain 2, axonemal OS=Mus musculus GN=Dnah2 PE=1 SV=1
Q8BZQ7	0.183906964	3	0	3	0	0	0	0	0	Anaphase-promoting complex subunit 2 OS=Mus musculus GN=Anapc2 PE=1 SV=2
O70492	0.183906964	2	0	2	0	0	0	0	0	Sorting nexin-3 OS=Mus musculus GN=Snx3 PE=1 SV=3
Q69ZX6	0.183906964	3	0	3	0	0	0	0	0	MORC family CW-type zinc finger protein 2A OS=Mus musculus GN=Morc2a PE=1 SV=2
P0C6F1	0.183906964	2	0	2	0	0	0	0	0	Dynein heavy chain 2, axonemal OS=Mus musculus GN=Dnah2 PE=1 SV=1
Q78ZM0	0.183906964	2	0	2	0	0	0	0	0	Putative uncharacterized protein OS=Mus musculus GN=Snx3 PE=1 SV=1
D3Z7E5	0.183906964	5	0	5	0	0	0	0	0	Glycogen synthase kinase-3 alpha OS=Mus musculus GN=Gsk3a PE=1 SV=1
D3Z6Z0	0.183906964	2	0	2	0	0	0	0	0	Sorting nexin-3 OS=Mus musculus GN=Snx3 PE=1 SV=1
A0A0G2JFJ6	0.183906964	5	0	5	0	0	0	0	0	Integrator complex subunit 3 OS=Mus musculus GN=Ints3 PE=1 SV=1
D3YY73	0.183906964	8	0	8	0	0	0	0	0	Histone deacetylase complex subunit SAP18 OS=Mus musculus GN=Sap18 PE=1 SV=1
Q7TPD0	0.183906964	5	0	5	0	0	0	0	0	Integrator complex subunit 3 OS=Mus musculus GN=Ints3 PE=1 SV=2
P52431	0.183906964	3	0	3	0	0	0	0	0	DNA polymerase delta catalytic subunit OS=Mus musculus GN=Pold1 PE=1 SV=2
P63044	0.183906964	2	0	2	0	0	0	0	0	Vesicle-associated membrane protein 2 OS=Mus musculus GN=Vamp2 PE=1 SV=2
P63024	0.183906964	2	0	2	0	0	0	0	0	Vesicle-associated membrane protein 3 OS=Mus musculus GN=Vamp3 PE=1 SV=1
B0QZN5	0.183906964	2	0	2	0	0	0	0	0	Vesicle-associated membrane protein 2 OS=Mus musculus GN=Vamp2 PE=1 SV=1
Q3UHD6	0.183906964	3	0	3	0	0	0	0	0	Sorting nexin-27 OS=Mus musculus GN=Snx27 PE=1 SV=2
P28659	0.183963778	0	3	4	0	0	0	0	0	CUGBP Elav-like family member 1 OS=Mus musculus GN=Celf1 PE=1 SV=2
A2ASC6	0.183963778	0	3	4	0	0	0	0	0	mRNA export factor (Fragment) OS=Mus musculus GN=Rae1 PE=1 SV=1
Q91XL3	0.183963778	0	3	4	0	0	0	0	0	UDP-glucuronic acid decarboxylase 1 OS=Mus musculus GN=Uxs1 PE=1 SV=1
A0A0R4J0T5	0.183963778	0	3	4	0	0	0	0	0	CUG triplet repeat, RNA binding protein 1, isoform CRA_b OS=Mus musculus GN=Celf1 PE=1 SV=1
Q6NTA4	0.185670288	5	3	0	0	0	0	0	0	Ras-related GTP-binding protein B OS=Mus musculus GN=Rragb PE=1 SV=1
Q80X95	0.185670288	5	3	0	0	0	0	0	0	Ras-related GTP-binding protein A OS=Mus musculus GN=Rraga PE=1 SV=1
Q8N7N5	0.186104718	0	0	8	2	0	0	0	0	DDB1- and CUL4-associated factor 8 OS=Mus musculus GN=Dcaf8 PE=1 SV=1
H3BJW3	0.186119761	3	0	2	0	0	0	0	0	Cleavage and polyadenylation-specificity factor subunit 6 OS=Mus musculus GN=Cpsf6 PE=1 SV=1
A2A5K2	0.186119761	3	0	2	0	0	0	0	0	Phospholipid transfer protein OS=Mus musculus GN=Pltp PE=1 SV=1
H3BJ30	0.186119761	3	0	2	0	0	0	0	0	Cleavage and polyadenylation-specificity factor subunit 6 OS=Mus musculus GN=Cpsf6 PE=1 SV=1
P17095	0.186119761	3	0	2	0	0	0	0	0	High mobility group protein HMG-I/HMG-Y OS=Mus musculus GN=Hmga1 PE=1 SV=4
P55065	0.186119761	3	0	2	0	0	0	0	0	Phospholipid transfer protein OS=Mus musculus GN=Pltp PE=1 SV=1
Q8C2D3	0.186119761	3	0	2	0	0	0	0	0	Putative uncharacterized protein OS=Mus musculus GN=Tank PE=1 SV=1
Q6NVF9	0.186119761	3	0	2	0	0	0	0	0	Cleavage and polyadenylation specificity factor subunit 6 OS=Mus musculus GN=Cpsf6 PE=1 SV=1
B1AQD9	0.186119761	3	0	2	0	0	0	0	0	Protein unc-119 homolog A OS=Mus musculus GN=Unc119 PE=1 SV=1
O08688	0.186119761	3	0	2	0	0	0	0	0	Calpain-5 OS=Mus musculus GN=Capn5 PE=1 SV=1
A2AR78	0.186119761	3	0	2	0	0	0	0	0	TRAF family member-associated NF-kappa-B activator OS=Mus musculus GN=Tank PE=1 SV=1
A2AR76	0.186119761	3	0	2	0	0	0	0	0	TRAF family member-associated NF-kappa-B activator OS=Mus musculus GN=Tank PE=1 SV=1
Q9Z2R6	0.186119761	3	0	2	0	0	0	0	0	Protein unc-119 homolog A OS=Mus musculus GN=Unc119 PE=1 SV=1
A0A0G2JF85	0.186119761	3	0	2	0	0	0	0	0	Sorting nexin-27 OS=Mus musculus GN=Snx27 PE=1 SV=1
P70347	0.186119761	3	0	2	0	0	0	0	0	TRAF family member-associated NF-kappa-B activator OS=Mus musculus GN=Tank PE=1 SV=1
Q3TCJ1	0.187539731	7	4	0	0	0	0	0	0	BRISC complex subunit Abro1 OS=Mus musculus GN=Fam175b PE=1 SV=1
D3Z4D8	0.187539731	7	4	0	0	0	0	0	0	BRISC complex subunit Abro1 (Fragment) OS=Mus musculus GN=Fam175b PE=1 SV=2
Z4YJY0	0.187539731	7	4	0	0	0	0	0	0	BRISC complex subunit Abro1 OS=Mus musculus GN=Fam175b PE=1 SV=1
O55128	0.188019961	8	0	9	0	0	0	0	0	Histone deacetylase complex subunit SAP18 OS=Mus musculus GN=Sap18 PE=1 SV=1
E9Q317	0.188019961	8	0	9	0	0	0	0	0	Histone deacetylase complex subunit SAP18 OS=Mus musculus GN=Sap18b PE=1 SV=1
Q5SUQ9	0.188026807	2	2	0	0	0	0	0	0	CST complex subunit CTC1 OS=Mus musculus GN=Ctc1 PE=1 SV=2
Q8BHB4	0.188026807	2	2	0	0	0	0	0	0	WD repeat-containing protein 3 OS=Mus musculus GN=Wdr3 PE=1 SV=1
B8ZXI1	0.188026807	3	3	0	0	0	0	0	0	Queuine tRNA-ribosyltransferase accessory subunit 2 OS=Mus musculus GN=Qtrt2 PE=1 SV=2
Q921N6	0.188026807	2	2	0	0	0	0	0	0	Probable ATP-dependent RNA helicase DDX27 OS=Mus musculus GN=Ddx27 PE=1 SV=3
Q80YQ2	0.188026807	2	2	0	0	0	0	0	0	Mediator of RNA polymerase II transcription subunit 23 OS=Mus musculus GN=Med23 PE=1 SV=2
P11157	0.188026807	2	2	0	0	0	0	0	0	Ribonucleoside-diphosphate reductase subunit M2 OS=Mus musculus GN=Rrm2 PE=1 SV=1
Q8C4J7	0.188113512	0	2	3	0	0	0	0	0	Transducin beta-like protein 3 OS=Mus musculus GN=Tbl3 PE=2 SV=1
Q6PAC3	0.188113512	0	2	3	0	0	0	0	0	DDB1- and CUL4-associated factor 13 OS=Mus musculus GN=Dcaf13 PE=2 SV=2
Q9Z1G4	0.188680299	0	6	5	0	0	0	0	0	V-type proton ATPase 116 kDa subunit a isoform 1 OS=Mus musculus GN=Atp6v0a1 PE=1 SV=3
K3W4T3	0.188680299	0	6	5	0	0	0	0	0	V-type proton ATPase subunit a OS=Mus musculus GN=Atp6v0a1 PE=1 SV=1
G5E895	0.189476345	0	11	17	0	0	0	0	0	MCG142264, isoform CRA_b OS=Mus musculus GN=Akr1b10 PE=1 SV=1
Q8C570	0.190643167	0	5	4	0	0	0	0	0	mRNA export factor OS=Mus musculus GN=Rae1 PE=1 SV=1
E9Q455	0.192105147	23	25	0	0	0	0	0	0	Tropomyosin alpha-1 chain OS=Mus musculus GN=Tpm1 PE=1 SV=1
E9Q453	0.192105147	23	25	0	0	0	0	0	0	Tropomyosin alpha-1 chain OS=Mus musculus GN=Tpm1 PE=1 SV=1
E9Q456	0.192105147	23	25	0	0	0	0	0	0	Tropomyosin alpha-1 chain OS=Mus musculus GN=Tpm1 PE=1 SV=1
G5E8R0	0.192105147	23	25	0	0	0	0	0	0	Tropomyosin 1, alpha, isoform CRA_i OS=Mus musculus GN=Tpm1 PE=1 SV=1
G5E8R2	0.192105147	23	25	0	0	0	0	0	0	Tropomyosin 1, alpha, isoform CRA_k OS=Mus musculus GN=Tpm1 PE=1 SV=1
G5E8R1	0.192105147	23	25	0	0	0	0	0	0	Tropomyosin 1, alpha, isoform CRA_j OS=Mus musculus GN=Tpm1 PE=1 SV=1
A2AFQ9	0.193319142	4	0	5	0	0	0	0	0	Gem-associated protein 5 OS=Mus musculus GN=Gemin5 PE=1 SV=1
Q8BX17	0.193319142	4	0	5	0	0	0	0	0	Gem-associated protein 5 OS=Mus musculus GN=Gemin5 PE=1 SV=2
E9PUU4	0.193319142	4	0	5	0	0	0	0	0	Gem-associated protein 5 OS=Mus musculus GN=Gemin5 PE=1 SV=1
Q6PGC1	0.193319142	4	0	5	0	0	0	0	0	ATP-dependent RNA helicase DHX29 OS=Mus musculus GN=Dhx29 PE=1 SV=1
Q6P5D3	0.193441996	0	3	5	0	0	0	0	0	Putative ATP-dependent RNA helicase DHX57 OS=Mus musculus GN=Dhx57 PE=1 SV=2
O89112	0.194180622	0	4	3	0	0	0	0	0	LanC-like protein 1 OS=Mus musculus GN=Lancl1 PE=1 SV=1
Q8R3N1	0.194180622	0	4	3	0	0	0	0	0	Nucleolar protein 14 OS=Mus musculus GN=Nop14 PE=1 SV=2
G5E846	0.194180622	0	4	3	0	0	0	0	0	Peripherin OS=Mus musculus GN=Prph PE=1 SV=1
G3X981	0.194180622	0	4	3	0	0	0	0	0	Peripherin OS=Mus musculus GN=Prph PE=1 SV=1
Q3TTW5	0.194297975	6	3	0	0	0	0	0	0	Pumilio homolog 1 OS=Mus musculus GN=Pum1 PE=1 SV=1
Q80U78	0.194297975	6	3	0	0	0	0	0	0	Pumilio homolog 1 OS=Mus musculus GN=Pum1 PE=1 SV=2
A2A863	0.194297975	4	2	0	0	0	0	0	0	Integrin beta-4 OS=Mus musculus GN=Itgb4 PE=1 SV=1
Q8VI94	0.194297975	4	2	0	0	0	0	0	0	2'-5'-oligoadenylate synthase-like protein 1 OS=Mus musculus GN=Oasl1 PE=2 SV=1
Q62193	0.194297975	4	2	0	0	0	0	0	0	Replication protein A 32 kDa subunit OS=Mus musculus GN=Rpa2 PE=1 SV=1
Q3TE40	0.194297975	4	2	0	0	0	0	0	0	Putative uncharacterized protein OS=Mus musculus GN=Rpa2 PE=1 SV=1
E9Q6M7	0.194297975	6	3	0	0	0	0	0	0	Pumilio homolog 1 OS=Mus musculus GN=Pum1 PE=1 SV=1
F7BX26	0.197238517	3	0	4	0	0	0	0	0	Serine/threonine-protein phosphatase (Fragment) OS=Mus musculus GN=Ppp5c PE=1 SV=1
Q60676	0.197238517	3	0	4	0	0	0	0	0	Serine/threonine-protein phosphatase 5 OS=Mus musculus GN=Ppp5c PE=1 SV=3
F8WHR6	0.197238517	3	0	4	0	0	0	0	0	Probable ATP-dependent RNA helicase DDX46 OS=Mus musculus GN=Ddx46 PE=1 SV=1
P62320	0.198188024	0	5	9	0	0	0	0	0	Small nuclear ribonucleoprotein Sm D3 OS=Mus musculus GN=Snrpd3 PE=1 SV=1
A0A0N4SUX5	0.199064205	23	0	12	0	0	0	0	0	Gamma-enolase OS=Mus musculus GN=Eno2 PE=1 SV=1
O08739	0.200504347	5	0	7	0	0	0	0	0	AMP deaminase 3 OS=Mus musculus GN=Ampd3 PE=1 SV=2
P59325	0.200825625	4	5	0	0	0	0	0	0	Eukaryotic translation initiation factor 5 OS=Mus musculus GN=Eif5 PE=1 SV=1
A0A0G2JFK2	0.201865695	0	3	2	0	0	0	0	0	Far upstream element-binding protein 1 (Fragment) OS=Mus musculus GN=Fubp1 PE=1 SV=1
Q62074	0.201865695	0	3	2	0	0	0	0	0	Protein kinase C iota type OS=Mus musculus GN=Prkci PE=1 SV=3
Q3UVG3	0.202019349	4	0	2	0	0	0	0	0	Protein FAM91A1 OS=Mus musculus GN=Fam91a1 PE=1 SV=1
E9Q5B5	0.202019349	4	0	2	0	0	0	0	0	Hexokinase-2 OS=Mus musculus GN=Hk2 PE=1 SV=1
Q8BMA6	0.202019349	6	0	3	0	0	0	0	0	Signal recognition particle subunit SRP68 OS=Mus musculus GN=Srp68 PE=1 SV=2
Q9WUK4	0.202019349	4	0	2	0	0	0	0	0	Replication factor C subunit 2 OS=Mus musculus GN=Rfc2 PE=1 SV=1
P97452	0.202019349	4	0	2	0	0	0	0	0	Ribosome biogenesis protein BOP1 OS=Mus musculus GN=Bop1 PE=1 SV=1
Q8VCA8	0.205518834	4	0	6	0	0	0	0	0	Secernin-2 OS=Mus musculus GN=Scrn2 PE=1 SV=1
Q91YR7	0.205518834	2	0	3	0	0	0	0	0	Pre-mRNA-processing factor 6 OS=Mus musculus GN=Prpf6 PE=1 SV=1
Q62383	0.205518834	2	0	3	0	0	0	0	0	Transcription elongation factor SPT6 OS=Mus musculus GN=Supt6h PE=1 SV=2
contaminant_UBIQUITIN10	0.205682074	0	4	8	0	0	0	0	0	no description
Q9Z277	0.209598203	0	3	0	2	0	0	0	0	Tyrosine-protein kinase BAZ1B OS=Mus musculus GN=Baz1b PE=1 SV=2
Q3UM45	0.211041973	9	0	4	0	0	0	0	0	Protein phosphatase 1 regulatory subunit 7 OS=Mus musculus GN=Ppp1r7 PE=1 SV=2
Q3TIX9	0.213932305	3	0	5	0	0	0	0	0	U4/U6.U5 tri-snRNP-associated protein 2 OS=Mus musculus GN=Usp39 PE=1 SV=2
Q569Z5	0.213932305	3	0	5	0	0	0	0	0	Probable ATP-dependent RNA helicase DDX46 OS=Mus musculus GN=Ddx46 PE=1 SV=2
Q99JX7	0.214040447	7	0	3	0	0	0	0	0	Nuclear RNA export factor 1 OS=Mus musculus GN=Nxf1 PE=1 SV=3
Q6PDM2	0.214988347	4	6	0	0	0	0	0	0	Serine/arginine-rich splicing factor 1 OS=Mus musculus GN=Srsf1 PE=1 SV=3
H7BX95	0.214988347	4	6	0	0	0	0	0	0	Serine/arginine-rich splicing factor 1 OS=Mus musculus GN=Srsf1 PE=1 SV=1
E9QNV2	0.214988347	2	3	0	0	0	0	0	0	Mediator of RNA polymerase II transcription subunit 23 OS=Mus musculus GN=Med23 PE=1 SV=1
Reverse_tr|B1AXN3|B1AXN3_MOUSE	0.219482345	0	0	3	2	0	0	0	0	MCG56945 OS=Mus musculus GN=Gm382 PE=4 SV=1
Q3TJ91	0.219948484	5	0	2	0	0	0	0	0	Lethal(2) giant larvae protein homolog 2 OS=Mus musculus GN=Llgl2 PE=1 SV=2
J3QJU5	0.219948484	5	0	2	0	0	0	0	0	Lethal(2) giant larvae protein homolog 2 OS=Mus musculus GN=Llgl2 PE=1 SV=1
H3BKT5	0.221016497	0	12	0	2	0	0	0	0	Adenosylhomocysteinase OS=Mus musculus GN=Ahcyl2 PE=1 SV=1
F8WGT1	0.221016497	0	12	0	2	0	0	0	0	Adenosylhomocysteinase OS=Mus musculus GN=Ahcyl2 PE=1 SV=1
F8WI65	0.221016497	0	12	0	2	0	0	0	0	Adenosylhomocysteinase OS=Mus musculus GN=Ahcyl2 PE=1 SV=1
Q91YN5	0.224168476	3	5	0	0	0	0	0	0	UDP-N-acetylhexosamine pyrophosphorylase OS=Mus musculus GN=Uap1 PE=1 SV=1
Q3UHZ7	0.224168476	3	5	0	0	0	0	0	0	Putative uncharacterized protein OS=Mus musculus GN=Uap1 PE=1 SV=1
A0A0R4J1F6	0.224168476	3	5	0	0	0	0	0	0	UDP-N-acetylhexosamine pyrophosphorylase OS=Mus musculus GN=Uap1 PE=1 SV=1
A0A0R4J085	0.224168476	3	5	0	0	0	0	0	0	UDP-N-acetylhexosamine pyrophosphorylase OS=Mus musculus GN=Uap1 PE=1 SV=1
Q99J83	0.22434713	0	2	5	0	0	0	0	0	Autophagy protein 5 OS=Mus musculus GN=Atg5 PE=1 SV=1
Q91X52	0.22434713	0	2	5	0	0	0	0	0	L-xylulose reductase OS=Mus musculus GN=Dcxr PE=1 SV=2
B2KGR2	0.225492113	0	4	2	0	0	0	0	0	LanC-like protein 1 (Fragment) OS=Mus musculus GN=Lancl1 PE=1 SV=1
Q8K363	0.240964741	2	4	0	0	0	0	0	0	ATP-dependent RNA helicase DDX18 OS=Mus musculus GN=Ddx18 PE=1 SV=1
Q80VJ3	0.245864241	0	2	0	2	0	0	0	0	2'-deoxynucleoside 5'-phosphate N-hydrolase 1 OS=Mus musculus GN=Dnph1 PE=1 SV=2
G8JL40	0.245864241	0	2	0	2	0	0	0	0	Lysine-specific histone demethylase 1A (Fragment) OS=Mus musculus GN=Kdm1a PE=1 SV=1
W4VSN7	0.245864241	0	4	0	4	0	0	0	0	MCG123916 OS=Mus musculus GN=Gm3550 PE=4 SV=1
D3Z1N9	0.245864241	0	4	0	4	0	0	0	0	MCG9889 OS=Mus musculus GN=Gm10709 PE=4 SV=1
F6QL70	0.245864241	0	4	0	4	0	0	0	0	Protein Gm17669 OS=Mus musculus GN=Gm17669 PE=4 SV=1
P47915	0.245864241	0	4	0	4	0	0	0	0	60S ribosomal protein L29 OS=Mus musculus GN=Rpl29 PE=2 SV=2
B1AR17	0.250836144	2	0	5	0	0	0	0	0	Protein Chd3 OS=Mus musculus GN=Chd3 PE=1 SV=1
Q9D1H7	0.250836144	2	0	5	0	0	0	0	0	Golgi to ER traffic protein 4 homolog OS=Mus musculus GN=Get4 PE=1 SV=2
Q8BL97	0.250836144	2	0	5	0	0	0	0	0	Serine/arginine-rich splicing factor 7 OS=Mus musculus GN=Srsf7 PE=1 SV=1
B1ARD6	0.25689963	0	0	2	2	0	0	0	0	Protein Slfn9 OS=Mus musculus GN=Slfn9 PE=1 SV=1
O35218	0.263153266	0	6	2	0	0	0	0	0	Cleavage and polyadenylation specificity factor subunit 2 OS=Mus musculus GN=Cpsf2 PE=1 SV=1

D3Z729	0.391002219	0	0	4	0	0	0	0	0	Eukaryotic translation initiation factor 4E type 2 OS=Mus musculus GN=Eif4e2 PE=1 SV=1
Reverse_tr|S4R1P5|S4R1P5_MOUSE	0.391002219	0	0	2	0	0	0	0	0	Dystonin OS=Mus musculus GN=Dst PE=1 SV=1
D3Z730	0.391002219	0	0	4	0	0	0	0	0	Eukaryotic translation initiation factor 4E type 2 OS=Mus musculus GN=Eif4e2 PE=1 SV=1
P42859	0.391002219	2	0	0	0	0	0	0	0	Huntingtin OS=Mus musculus GN=Htt PE=1 SV=2
Q9Z130	0.391002219	4	0	0	0	0	0	0	0	Heterogeneous nuclear ribonucleoprotein D-like OS=Mus musculus GN=Hnrnpdl PE=1 SV=1
A0A0J9YUF9	0.391002219	0	0	0	2	0	0	0	0	Bifunctional polynucleotide phosphatase/kinase (Fragment) OS=Mus musculus GN=Pnkp PE=1 SV=1
H3BKA7	0.391002219	0	3	0	0	0	0	0	0	Neuroplastin OS=Mus musculus GN=Nptn PE=1 SV=1
Q9JKX6	0.391002219	2	0	0	0	0	0	0	0	ADP-sugar pyrophosphatase OS=Mus musculus GN=Nudt5 PE=1 SV=1
Q9DB77	0.391002219	0	4	0	0	0	0	0	0	Cytochrome b-c1 complex subunit 2, mitochondrial OS=Mus musculus GN=Uqcrc2 PE=1 SV=1
Q9DAV6	0.391002219	2	0	0	0	0	0	0	0	Protein Serpinb9b OS=Mus musculus GN=Serpinb9b PE=1 SV=1
D3YUV1	0.391002219	3	0	0	0	0	0	0	0	Nuclear receptor-binding protein OS=Mus musculus GN=Nrbp1 PE=1 SV=1
D3YUV9	0.391002219	0	0	4	0	0	0	0	0	Eukaryotic translation initiation factor 4E type 2 OS=Mus musculus GN=Eif4e2 PE=1 SV=1
Q8C3Y4	0.391002219	0	0	2	0	0	0	0	0	Kinetochore-associated protein 1 OS=Mus musculus GN=Kntc1 PE=1 SV=2
D3Z6W2	0.391002219	0	2	0	0	0	0	0	0	Tyrosine-protein phosphatase non-receptor type OS=Mus musculus GN=Ptpn2 PE=1 SV=1
E9Q405	0.391002219	3	0	0	0	0	0	0	0	Unconventional myosin-XVIIIa OS=Mus musculus GN=Myo18a PE=1 SV=1
Q8CE96	0.391002219	0	0	2	0	0	0	0	0	tRNA (adenine(58)-N(1))-methyltransferase non-catalytic subunit TRM6 OS=Mus musculus GN=Trmt6 PE=1 SV=1
A2A4P0	0.391002219	2	0	0	0	0	0	0	0	ATP-dependent RNA helicase DHX8 OS=Mus musculus GN=Dhx8 PE=2 SV=1
Reverse_sp|Q62209|SYCP1_MOUSE	0.391002219	2	0	0	0	0	0	0	0	Synaptonemal complex protein 1 OS=Mus musculus GN=Sycp1 PE=1 SV=2
Q9CWX9	0.391002219	2	0	0	0	0	0	0	0	Probable ATP-dependent RNA helicase DDX47 OS=Mus musculus GN=Ddx47 PE=2 SV=2
Q6PFD9	0.391002219	0	0	2	0	0	0	0	0	Nuclear pore complex protein Nup98-Nup96 OS=Mus musculus GN=Nup98 PE=1 SV=2
E9PZR3	0.391002219	3	0	0	0	0	0	0	0	Poly(A) polymerase alpha OS=Mus musculus GN=Papola PE=1 SV=1
Q8CDM1	0.391002219	0	0	0	2	0	0	0	0	ATPase family AAA domain-containing protein 2 OS=Mus musculus GN=Atad2 PE=1 SV=1
A2A4N9	0.391002219	2	0	0	0	0	0	0	0	ATP-dependent RNA helicase DHX8 (Fragment) OS=Mus musculus GN=Dhx8 PE=1 SV=8
Q6PFA1	0.391002219	0	0	3	0	0	0	0	0	Geranylgeranyl transferase type-2 subunit beta OS=Mus musculus GN=Rabggtb PE=1 SV=1
G3XA09	0.391002219	0	0	2	0	0	0	0	0	Apoptotic protease-activating factor 1 OS=Mus musculus GN=Apaf1 PE=1 SV=1
Reverse_sp|Q9JMH9|MY18A_MOUSE	0.391002219	0	0	2	0	0	0	0	0	Unconventional myosin-XVIIIa OS=Mus musculus GN=Myo18a PE=1 SV=2
B2RRE2	0.391002219	3	0	0	0	0	0	0	0	Myo18a protein OS=Mus musculus GN=Myo18a PE=1 SV=1
Q9CWU9	0.391002219	0	0	4	0	0	0	0	0	Nucleoporin Nup37 OS=Mus musculus GN=Nup37 PE=1 SV=2
A0A0J9YV48	0.391002219	0	0	0	2	0	0	0	0	Bifunctional polynucleotide phosphatase/kinase (Fragment) OS=Mus musculus GN=Pnkp PE=1 SV=1
Q64471	0.391002219	0	2	0	0	0	0	0	0	Glutathione S-transferase theta-1 OS=Mus musculus GN=Gstt1 PE=1 SV=4
A0A0J9YUR5	0.391002219	0	2	0	0	0	0	0	0	C-terminal-binding protein 1 (Fragment) OS=Mus musculus GN=Ctbp1 PE=1 SV=1
Q6IRU5	0.391002219	0	0	4	0	0	0	0	0	Clathrin light chain B OS=Mus musculus GN=Cltb PE=1 SV=1
A2A4J8	0.391002219	2	0	0	0	0	0	0	0	Vacuolar protein-sorting-associated protein 25 OS=Mus musculus GN=Vps25 PE=1 SV=1
Q8VCV2	0.391002219	0	2	0	0	0	0	0	0	Ndrg3 protein OS=Mus musculus GN=Ndrg3 PE=1 SV=1
Q9DAK9	0.391002219	4	0	0	0	0	0	0	0	14 kDa phosphohistidine phosphatase OS=Mus musculus GN=Phpt1 PE=1 SV=1
Q9Z0G2	0.391002219	0	2	0	0	0	0	0	0	SRSF protein kinase 3 OS=Mus musculus GN=Srpk3 PE=1 SV=1
Reverse_tr|H7BX05|H7BX05_MOUSE	0.391002219	0	0	0	2	0	0	0	0	Obscurin OS=Mus musculus GN=Obscn PE=1 SV=2
E9Q3Y1	0.391002219	0	0	0	2	0	0	0	0	Serpin B6 (Fragment) OS=Mus musculus GN=Serpinb6a PE=1 SV=1
Q6NZH9	0.391002219	0	0	2	0	0	0	0	0	Protein Rasgrp3 OS=Mus musculus GN=Rasgrp3 PE=1 SV=1
E9Q3Y4	0.391002219	0	0	4	0	0	0	0	0	Lipopolysaccharide-responsive and beige-like anchor protein OS=Mus musculus GN=Lrba PE=1 SV=1
Q80WC7	0.391002219	0	0	2	0	0	0	0	0	Arf-GAP domain and FG repeat-containing protein 2 OS=Mus musculus GN=Agfg2 PE=1 SV=1
P55288	0.391002219	0	0	5	0	0	0	0	0	Cadherin-11 OS=Mus musculus GN=Cdh11 PE=1 SV=1
Reverse_tr|A2AD39|A2AD39_MOUSE	0.391002219	0	2	0	0	0	0	0	0	SCL-interrupting locus protein homolog OS=Mus musculus GN=Stil PE=4 SV=1
Q80WC1	0.391002219	0	0	0	2	0	0	0	0	Ubinuclein-2 OS=Mus musculus GN=Ubn2 PE=1 SV=2
Q8BK58	0.391002219	5	0	0	0	0	0	0	0	HSPB1-associated protein 1 OS=Mus musculus GN=Hspbap1 PE=1 SV=2
Q9JLC8	0.391002219	3	0	0	0	0	0	0	0	Sacsin OS=Mus musculus GN=Sacs PE=1 SV=2
A0A087WRU0	0.391002219	0	0	0	2	0	0	0	0	Protein Tns1 (Fragment) OS=Mus musculus GN=Tns1 PE=1 SV=1
Q99JR8	0.391002219	0	0	3	0	0	0	0	0	SWI/SNF-related matrix-associated actin-dependent regulator of chromatin subfamily D member 2 OS=Mus musculus GN=Smarcd2 PE=1 SV=2
Q9CXW7	0.391002219	0	0	0	5	0	0	0	0	40S ribosomal protein S9 OS=Mus musculus GN=Rps9 PE=1 SV=1
Q7TMF2	0.391002219	0	0	2	0	0	0	0	0	3'-5' exoribonuclease 1 OS=Mus musculus GN=Eri1 PE=1 SV=2
O88895	0.391002219	4	0	0	0	0	0	0	0	Histone deacetylase 3 OS=Mus musculus GN=Hdac3 PE=1 SV=1
Q99JX3	0.391002219	0	0	4	0	0	0	0	0	Golgi reassembly-stacking protein 2 OS=Mus musculus GN=Gorasp2 PE=1 SV=3
A0A0J9YUY8	0.391002219	0	0	5	0	0	0	0	0	Ras GTPase-activating protein-binding protein 2 (Fragment) OS=Mus musculus GN=G3bp2 PE=1 SV=1
Reverse_tr|E9QPE7|E9QPE7_MOUSE	0.391002219	2	0	0	0	0	0	0	0	Myosin-11 OS=Mus musculus GN=Myh11 PE=1 SV=1
O88879	0.391002219	0	0	2	0	0	0	0	0	Apoptotic protease-activating factor 1 OS=Mus musculus GN=Apaf1 PE=1 SV=3
E9QMR2	0.391002219	2	0	0	0	0	0	0	0	Dedicator of cytokinesis protein 9 OS=Mus musculus GN=Dock9 PE=1 SV=1
D3YUG9	0.391002219	0	0	2	0	0	0	0	0	Quinone oxidoreductase (Fragment) OS=Mus musculus GN=Cryz PE=1 SV=1
Q9CY52	0.391002219	0	0	4	0	0	0	0	0	Probable tRNA(His) guanylyltransferase OS=Mus musculus GN=Thg1l PE=1 SV=1
Q99K74	0.391002219	0	2	0	0	0	0	0	0	Mediator of RNA polymerase II transcription subunit 24 OS=Mus musculus GN=Med24 PE=1 SV=1
A2AFF6	0.391002219	0	0	3	0	0	0	0	0	Cohesin subunit SA-2 OS=Mus musculus GN=Stag2 PE=1 SV=1
Q7TMC8	0.391002219	0	2	0	0	0	0	0	0	L-fucose kinase OS=Mus musculus GN=Fuk PE=1 SV=1
D3Z5W6	0.391002219	2	0	0	0	0	0	0	0	Fatty acyl-CoA reductase (Fragment) OS=Mus musculus GN=Far1 PE=1 SV=1
P18242	0.391002219	3	0	0	0	0	0	0	0	Cathepsin D OS=Mus musculus GN=Ctsd PE=1 SV=1
Reverse_sp|Q9JKK8|ATR_MOUSE	0.391002219	0	2	0	0	0	0	0	0	Serine/threonine-protein kinase ATR OS=Mus musculus GN=Atr PE=1 SV=2
Q5SVF9	0.391002219	0	0	2	0	0	0	0	0	THO complex subunit 5 homolog OS=Mus musculus GN=Thoc5 PE=1 SV=1
Q56A07	0.391002219	5	0	0	0	0	0	0	0	Sodium channel subunit beta-2 OS=Mus musculus GN=Scn2b PE=1 SV=1
F6Y6L6	0.391002219	3	0	0	0	0	0	0	0	Uncharacterized protein (Fragment) OS=Mus musculus GN=Ctsd PE=1 SV=1
Q60963	0.391002219	0	0	2	0	0	0	0	0	Platelet-activating factor acetylhydrolase OS=Mus musculus GN=Pla2g7 PE=2 SV=2
Q9R1T2	0.391002219	0	0	5	0	0	0	0	0	SUMO-activating enzyme subunit 1 OS=Mus musculus GN=Sae1 PE=1 SV=1
E9QME5	0.391002219	0	2	0	0	0	0	0	0	E3 ubiquitin-protein ligase TRIM33 OS=Mus musculus GN=Trim33 PE=1 SV=1
Q9QYF9	0.391002219	0	2	0	0	0	0	0	0	Protein NDRG3 OS=Mus musculus GN=Ndrg3 PE=1 SV=1
A2AWA9	0.391002219	0	0	2	0	0	0	0	0	Rab GTPase-activating protein 1 OS=Mus musculus GN=Rabgap1 PE=1 SV=1
A2AWA7	0.391002219	0	0	2	0	0	0	0	0	Rab GTPase-activating protein 1 (Fragment) OS=Mus musculus GN=Rabgap1 PE=1 SV=1
G3X926	0.391002219	0	2	0	0	0	0	0	0	MCG1407, isoform CRA_a OS=Mus musculus GN=Rpf2 PE=1 SV=1
G3X920	0.391002219	2	0	0	0	0	0	0	0	Armadillo repeat containing 8, isoform CRA_b OS=Mus musculus GN=Armc8 PE=1 SV=1
Reverse_sp|Q924A2|CIC_MOUSE	0.391002219	0	2	0	0	0	0	0	0	Protein capicua homolog OS=Mus musculus GN=Cic PE=1 SV=2
A2AWB0	0.391002219	0	0	2	0	0	0	0	0	Rab GTPase-activating protein 1 (Fragment) OS=Mus musculus GN=Rabgap1 PE=1 SV=8
Q6PFV6	0.391002219	0	0	3	0	0	0	0	0	Epha5 protein OS=Mus musculus GN=Epha5 PE=1 SV=1
A0A0J9YVI3	0.391002219	0	2	0	0	0	0	0	0	C-terminal-binding protein 1 (Fragment) OS=Mus musculus GN=Ctbp1 PE=1 SV=1
Q3TJZ6	0.391002219	0	0	2	0	0	0	0	0	Protein FAM98A OS=Mus musculus GN=Fam98a PE=1 SV=1
D3YTQ3	0.391002219	4	0	0	0	0	0	0	0	Heterogeneous nuclear ribonucleoprotein D-like OS=Mus musculus GN=Hnrnpdl PE=1 SV=1
P28667	0.391002219	3	0	0	0	0	0	0	0	MARCKS-related protein OS=Mus musculus GN=Marcksl1 PE=1 SV=2
Q8CD92	0.391002219	0	0	3	0	0	0	0	0	Tetratricopeptide repeat protein 27 OS=Mus musculus GN=Ttc27 PE=1 SV=2
P53612	0.391002219	0	0	3	0	0	0	0	0	Geranylgeranyl transferase type-2 subunit beta OS=Mus musculus GN=Rabggtb PE=1 SV=2
G3UXC0	0.391002219	0	0	3	0	0	0	0	0	SWI/SNF-related matrix-associated actin-dependent regulator of chromatin subfamily D member 2 (Fragment) OS=Mus musculus GN=Smarcd2 PE=1 SV=1
Reverse_sp|A2CG49|KALRN_MOUSE	0.391002219	2	0	0	0	0	0	0	0	Kalirin OS=Mus musculus GN=Kalrn PE=1 SV=1
Q8BHY2	0.391002219	0	0	5	0	0	0	0	0	Nucleolar complex protein 4 homolog OS=Mus musculus GN=Noc4l PE=2 SV=1
Reverse_sp|Q9WTI7|MYO1C_MOUSE	0.391002219	0	0	2	0	0	0	0	0	Unconventional myosin-Ic OS=Mus musculus GN=Myo1c PE=1 SV=2
O55143	0.391002219	0	0	2	0	0	0	0	0	Sarcoplasmic/endoplasmic reticulum calcium ATPase 2 OS=Mus musculus GN=Atp2a2 PE=1 SV=2
G3UXE9	0.391002219	0	0	4	0	0	0	0	0	Protein Gm8909 OS=Mus musculus GN=Gm8909 PE=1 SV=1
Q8CD15	0.391002219	0	0	3	0	0	0	0	0	Bifunctional lysine-specific demethylase and histidyl-hydroxylase MINA OS=Mus musculus GN=Mina PE=1 SV=2
Q3UVL4	0.391002219	0	3	0	0	0	0	0	0	Vacuolar protein sorting-associated protein 51 homolog OS=Mus musculus GN=Vps51 PE=1 SV=2
Q80UJ7	0.391002219	2	0	0	0	0	0	0	0	Rab3 GTPase-activating protein catalytic subunit OS=Mus musculus GN=Rab3gap1 PE=1 SV=4
Q99J45	0.391002219	3	0	0	0	0	0	0	0	Nuclear receptor-binding protein OS=Mus musculus GN=Nrbp1 PE=1 SV=1
Q9DAA6	0.391002219	0	2	0	0	0	0	0	0	Exosome complex component CSL4 OS=Mus musculus GN=Exosc1 PE=1 SV=1
Reverse_tr|B2RRE2|B2RRE2_MOUSE	0.391002219	0	0	2	0	0	0	0	0	Myo18a protein OS=Mus musculus GN=Myo18a PE=1 SV=1
O54781	0.391002219	0	2	0	0	0	0	0	0	SRSF protein kinase 2 OS=Mus musculus GN=Srpk2 PE=1 SV=2
Reverse_tr|D6RHQ6|D6RHQ6_MOUSE	0.391002219	0	2	0	0	0	0	0	0	Ubiquitinyl hydrolase 1 OS=Mus musculus GN=Usp16 PE=1 SV=1
Q8BIQ5	0.391002219	3	0	0	0	0	0	0	0	Cleavage stimulation factor subunit 2 OS=Mus musculus GN=Cstf2 PE=1 SV=2
Reverse_tr|F6VZH1|F6VZH1_MOUSE	0.391002219	0	2	0	0	0	0	0	0	Small G protein-signaling modulator 3 (Fragment) OS=Mus musculus GN=Sgsm3 PE=1 SV=1
Reverse_sp|P97378|I12R2_MOUSE	0.391002219	3	0	0	0	0	0	0	0	Interleukin-12 receptor subunit beta-2 OS=Mus musculus GN=Il12rb2 PE=1 SV=1
Q99J79	0.391002219	0	2	0	0	0	0	0	0	DNA damage-binding protein 2 OS=Mus musculus GN=Ddb2 PE=1 SV=1
F6T941	0.391002219	0	0	0	2	0	0	0	0	Transcription factor E2-alpha (Fragment) OS=Mus musculus GN=Tcf3 PE=1 SV=1
Q8BRF7	0.391002219	2	0	0	0	0	0	0	0	Sec1 family domain-containing protein 1 OS=Mus musculus GN=Scfd1 PE=1 SV=1
E9Q5C9	0.391002219	0	3	0	0	0	0	0	0	Protein Nolc1 OS=Mus musculus GN=Nolc1 PE=1 SV=1
Q8VCD5	0.391002219	0	2	0	0	0	0	0	0	Mediator of RNA polymerase II transcription subunit 17 OS=Mus musculus GN=Med17 PE=1 SV=1
P10400	0.391002219	2	0	0	0	0	0	0	0	Retrovirus-related Pol polyprotein (Fragment) OS=Mus musculus GN=Pol PE=2 SV=1
Q80UW8	0.391002219	3	0	0	0	0	0	0	0	DNA-directed RNA polymerases I, II, and III subunit RPABC1 OS=Mus musculus GN=Polr2e PE=1 SV=1
F7CJS8	0.391002219	0	0	0	5	0	0	0	0	40S ribosomal protein S9 (Fragment) OS=Mus musculus GN=Rps9 PE=1 SV=1
A2AP29	0.391002219	0	2	0	0	0	0	0	0	Probable ATP-dependent RNA helicase DDX58 (Fragment) OS=Mus musculus GN=Ddx58 PE=1 SV=1
Q6Q899	0.391002219	0	2	0	0	0	0	0	0	Probable ATP-dependent RNA helicase DDX58 OS=Mus musculus GN=Ddx58 PE=1 SV=2
Q5SUF2	0.391002219	0	0	2	0	0	0	0	0	Luc7-like protein 3 OS=Mus musculus GN=Luc7l3 PE=1 SV=1
Q8VCF1	0.391002219	0	0	3	0	0	0	0	0	Soluble calcium-activated nucleotidase 1 OS=Mus musculus GN=Cant1 PE=2 SV=1
Q6P4S8	0.391002219	2	0	0	0	0	0	0	0	Integrator complex subunit 1 OS=Mus musculus GN=Ints1 PE=1 SV=2
Q6P549	0.391002219	3	0	0	0	0	0	0	0	Phosphatidylinositol 3,4,5-trisphosphate 5-phosphatase 2 OS=Mus musculus GN=Inppl1 PE=1 SV=1
Q922J9	0.391002219	2	0	0	0	0	0	0	0	Fatty acyl-CoA reductase 1 OS=Mus musculus GN=Far1 PE=1 SV=1
Q99PP7	0.391002219	0	2	0	0	0	0	0	0	E3 ubiquitin-protein ligase TRIM33 OS=Mus musculus GN=Trim33 PE=1 SV=2
Q8R5K4	0.391002219	0	0	2	0	0	0	0	0	Nucleolar protein 6 OS=Mus musculus GN=Nol6 PE=2 SV=2
E9PXW3	0.391002219	0	0	0	3	0	0	0	0	Tetratricopeptide repeat protein 14 OS=Mus musculus GN=Ttc14 PE=1 SV=3
Q64261	0.391002219	0	0	3	0	0	0	0	0	Cyclin-dependent kinase 6 OS=Mus musculus GN=Cdk6 PE=1 SV=2
A2CES4	0.391002219	3	0	0	0	0	0	0	0	U2 small nuclear ribonucleoprotein B'' (Fragment) OS=Mus musculus GN=Snrpb2 PE=1 SV=1
Q80U44	0.391002219	0	0	0	2	0	0	0	0	Zinc finger FYVE domain-containing protein 16 OS=Mus musculus GN=Zfyve16 PE=1 SV=2
Q6A009	0.391002219	6	0	0	0	0	0	0	0	E3 ubiquitin-protein ligase listerin OS=Mus musculus GN=Ltn1 PE=1 SV=3
Reverse_sp|Q8CG73|FTM_MOUSE	0.391002219	2	0	0	0	0	0	0	0	Protein fantom OS=Mus musculus GN=Rpgrip1l PE=1 SV=2
Reverse_tr|Q8BXX3|Q8BXX3_MOUSE	0.391002219	2	0	0	0	0	0	0	0	GPI-anchor transamidase OS=Mus musculus GN=Pigk PE=1 SV=1
Reverse_sp|P11369|LORF2_MOUSE	0.391002219	0	2	0	0	0	0	0	0	LINE-1 retrotransposable element ORF2 protein OS=Mus musculus GN=Pol PE=1 SV=2
B1AQ75	0.391002219	0	5	0	0	0	0	0	0	Keratin, type I cuticular Ha6 OS=Mus musculus GN=Krt36 PE=1 SV=1
B1AQ77	0.391002219	0	0	18	0	0	0	0	0	Keratin 15, isoform CRA_a OS=Mus musculus GN=Krt15 PE=1 SV=1
Reverse_tr|E9QPK4|E9QPK4_MOUSE	0.391002219	0	2	0	0	0	0	0	0	Serine/threonine-protein kinase ATR OS=Mus musculus GN=Atr PE=1 SV=1
Q3UUT0	0.391002219	0	0	4	0	0	0	0	0	Eukaryotic translation initiation factor 4E type 2 OS=Mus musculus GN=Eif4e2 PE=1 SV=1
Q922K9	0.391002219	0	0	0	2	0	0	0	0	Tyrosine-protein kinase FRK OS=Mus musculus GN=Frk PE=1 SV=3
Q8K0Y2	0.391002219	0	24	0	0	0	0	0	0	Keratin, type I cuticular Ha3-I OS=Mus musculus GN=Krt33a PE=1 SV=1
Reverse_sp|Q99LG0|UBP16_MOUSE	0.391002219	0	2	0	0	0	0	0	0	Ubiquitin carboxyl-terminal hydrolase 16 OS=Mus musculus GN=Usp16 PE=1 SV=2
Q9ESJ0	0.391002219	0	0	4	0	0	0	0	0	Exportin-4 OS=Mus musculus GN=Xpo4 PE=1 SV=2
E9Q9M1	0.391002219	3	0	0	0	0	0	0	0	Cytosolic purine 5'-nucleotidase OS=Mus musculus GN=Nt5c2 PE=1 SV=1
Q8BH76	0.391002219	0	0	0	2	0	0	0	0	DNA polymerase delta subunit 3 OS=Mus musculus GN=Pold3 PE=1 SV=1
Q9JIF6	0.391002219	0	0	3	0	0	0	0	0	Geranylgeranyl transferase type-2 subunit beta OS=Mus musculus GN=Rabggtb PE=1 SV=1
A0A1B0GT66	0.391002219	3	0	0	0	0	0	0	0	Uncharacterized protein (Fragment) OS=Mus musculus PE=1 SV=1
Q8BPM2	0.391002219	0	0	3	0	0	0	0	0	Mitogen-activated protein kinase kinase kinase kinase 5 OS=Mus musculus GN=Map4k5 PE=1 SV=2
O88597	0.391002219	0	0	2	0	0	0	0	0	Beclin-1 OS=Mus musculus GN=Becn1 PE=1 SV=3
Q91Z50	0.391002219	5	0	0	0	0	0	0	0	Flap endonuclease 1 OS=Mus musculus GN=Fen1 PE=1 SV=1
Q9ESL4	0.391002219	4	0	0	0	0	0	0	0	Mitogen-activated protein kinase kinase kinase MLT OS=Mus musculus GN=Zak PE=1 SV=1
Q61183	0.391002219	3	0	0	0	0	0	0	0	Poly(A) polymerase alpha OS=Mus musculus GN=Papola PE=1 SV=4
Q69ZR2	0.391002219	3	0	0	0	0	0	0	0	E3 ubiquitin-protein ligase HECTD1 OS=Mus musculus GN=Hectd1 PE=1 SV=2
A2A709	0.391002219	4	0	0	0	0	0	0	0	Secernin-2 (Fragment) OS=Mus musculus GN=Scrn2 PE=1 SV=1
O88587	0.391002219	0	0	2	0	0	0	0	0	Catechol O-methyltransferase OS=Mus musculus GN=Comt PE=1 SV=2
Q6EDY6	0.391002219	4	0	0	0	0	0	0	0	F-actin-uncapping protein LRRC16A OS=Mus musculus GN=Carmil1 PE=1 SV=2
E0CXE7	0.391002219	2	0	0	0	0	0	0	0	CST complex subunit CTC1 OS=Mus musculus GN=Ctc1 PE=1 SV=1
Q8BI06	0.391002219	0	2	0	0	0	0	0	0	Cell migration-inducing and hyaluronan-binding protein OS=Mus musculus GN=Cemip PE=1 SV=4
Reverse_contaminant_IGA0_HAEIN	0.391002219	2	0	0	0	0	0	0	0	owl|P44969| IMMUNOGLOBULIN A1 PROTEASE PRECURSOR (EC 3.4.21.72) (IGA1 PROTEASE)....
Q9JJF3	0.391002219	0	2	0	0	0	0	0	0	Bifunctional lysine-specific demethylase and histidyl-hydroxylase NO66 OS=Mus musculus GN=No66 PE=1 SV=2
E9PYJ6	0.391002219	0	0	3	0	0	0	0	0	Focadhesin OS=Mus musculus GN=Focad PE=1 SV=1
A0A1B0GSK8	0.391002219	0	0	0	13	0	0	0	0	Tetraspanin (Fragment) OS=Mus musculus GN=Cd151 PE=1 SV=1
Q9D832	0.391002219	0	3	0	0	0	0	0	0	DnaJ homolog subfamily B member 4 OS=Mus musculus GN=Dnajb4 PE=1 SV=1
Reverse_contaminant_IGA2_HAEIN	0.391002219	2	0	0	0	0	0	0	0	owl|P45384| IMMUNOGLOBULIN A1 PROTEASE PRECURSOR (EC 3.4.21.72) (IGA1 PROTEASE)....
Reverse_tr|E9Q202|E9Q202_MOUSE	0.391002219	0	0	2	0	0	0	0	0	Low-density lipoprotein receptor-related protein 1B OS=Mus musculus GN=Lrp1b PE=1 SV=2
V9GXY8	0.391002219	0	0	2	0	0	0	0	0	Quinone oxidoreductase OS=Mus musculus GN=Cryz PE=1 SV=1
O88632	0.391002219	2	0	0	0	0	0	0	0	Semaphorin-3F OS=Mus musculus GN=Sema3f PE=2 SV=2
P41241	0.391002219	2	0	0	0	0	0	0	0	Tyrosine-protein kinase CSK OS=Mus musculus GN=Csk PE=1 SV=2
Q9JJ80	0.391002219	0	2	0	0	0	0	0	0	Ribosome production factor 2 homolog OS=Mus musculus GN=Rpf2 PE=2 SV=2
Q99PW4	0.391002219	2	0	0	0	0	0	0	0	TP53-regulating kinase OS=Mus musculus GN=Tp53rk PE=2 SV=1
A8XY17	0.391002219	2	0	0	0	0	0	0	0	Vacuolar protein-sorting-associated protein 25 OS=Mus musculus GN=Vps25 PE=1 SV=1
Q9D7J7	0.391002219	0	0	2	0	0	0	0	0	Calpain small subunit 2 OS=Mus musculus GN=Capns2 PE=2 SV=1
Reverse_sp|P08553|NFM_MOUSE	0.391002219	0	0	0	3	0	0	0	0	Neurofilament medium polypeptide OS=Mus musculus GN=Nefm PE=1 SV=4
J3QMW5	0.391002219	0	0	0	2	0	0	0	0	Protein Gm2075 OS=Mus musculus GN=Gm2075 PE=3 SV=1

Q8BHL4	0.391002219	3	0	0	0	0	0	0	0	Retinoic acid-induced protein 3 OS=Mus musculus GN=Gprc5a PE=1 SV=1
D3Z030	0.391002219	4	0	0	0	0	0	0	0	F-actin-uncapping protein LRRC16A OS=Mus musculus GN=Carmil1 PE=1 SV=2
E9Q1T3	0.391002219	0	0	3	0	0	0	0	0	Mitogen-activated protein kinase kinase kinase kinase OS=Mus musculus GN=Map4k5 PE=1 SV=1
E0CY49	0.391002219	2	0	0	0	0	0	0	0	Argininosuccinate lyase (Fragment) OS=Mus musculus GN=Asl PE=1 SV=1
Reverse_tr|A0A0U1RPD8|A0A0U1RPD8_MOUSE	0.391002219	2	0	0	0	0	0	0	0	General transcription factor 3C polypeptide 1 OS=Mus musculus GN=Gtf3c1 PE=1 SV=1
E9PYX7	0.391002219	0	0	2	0	0	0	0	0	Afadin OS=Mus musculus GN=Afdn PE=1 SV=1
Reverse_tr|Q6A099|Q6A099_MOUSE	0.391002219	0	0	5	0	0	0	0	0	MKIAA0248 protein (Fragment) OS=Mus musculus GN=Gbf1 PE=1 SV=1
Q91XV3	0.391002219	0	5	0	0	0	0	0	0	Brain acid soluble protein 1 OS=Mus musculus GN=Basp1 PE=1 SV=3
Q8C156	0.391002219	0	2	0	0	0	0	0	0	Condensin complex subunit 2 OS=Mus musculus GN=Ncaph PE=1 SV=1
Q3U2K8	0.391002219	0	0	2	0	0	0	0	0	Arf-GAP domain and FG repeat-containing protein 2 OS=Mus musculus GN=Agfg2 PE=1 SV=1
E0CY11	0.391002219	0	0	0	2	0	0	0	0	Neurexin-1 OS=Mus musculus GN=Nrxn1 PE=1 SV=2
O70551	0.391002219	0	2	0	0	0	0	0	0	SRSF protein kinase 1 OS=Mus musculus GN=Srpk1 PE=1 SV=2
Q91XZ2	0.391002219	0	0	2	0	0	0	0	0	Protocadherin beta-8 OS=Mus musculus GN=Pcdhb8 PE=1 SV=1
Q0P688	0.391002219	0	0	4	0	0	0	0	0	Eukaryotic translation initiation factor 4E member 2 OS=Mus musculus GN=Eif4e2 PE=1 SV=1
P54729	0.391002219	3	0	0	0	0	0	0	0	NEDD8 ultimate buster 1 OS=Mus musculus GN=Nub1 PE=1 SV=2
Q8C129	0.391002219	2	0	0	0	0	0	0	0	Leucyl-cystinyl aminopeptidase OS=Mus musculus GN=Lnpep PE=1 SV=1
P97861	0.391002219	0	60	0	0	0	0	0	0	Keratin, type II cuticular Hb6 OS=Mus musculus GN=Krt86 PE=2 SV=2
Q9D646	0.391002219	0	30	0	0	0	0	0	0	Keratin, type I cuticular Ha4 OS=Mus musculus GN=Krt34 PE=2 SV=1
Q61033	0.391002219	0	0	0	2	0	0	0	0	Lamina-associated polypeptide 2, isoforms alpha/zeta OS=Mus musculus GN=Tmpo PE=1 SV=4
F6SMY7	0.391002219	0	2	0	0	0	0	0	0	E3 ubiquitin-protein ligase MYCBP2 (Fragment) OS=Mus musculus GN=Mycbp2 PE=1 SV=1
Q8C0D5	0.391002219	2	0	0	0	0	0	0	0	Elongation factor-like GTPase 1 OS=Mus musculus GN=Efl1 PE=1 SV=1
A2AQE4	0.391002219	0	2	0	0	0	0	0	0	COP9 signalosome complex subunit 2 OS=Mus musculus GN=Cops2 PE=1 SV=1
D3Z7Q3	0.391002219	3	0	0	0	0	0	0	0	Guanine nucleotide-binding protein subunit gamma OS=Mus musculus GN=Gng5 PE=1 SV=1
Q8BWY9	0.391002219	2	0	0	0	0	0	0	0	Protein CIP2A OS=Mus musculus GN=Kiaa1524 PE=1 SV=3
F6WYM0	0.391002219	0	0	0	7	0	0	0	0	Septin-2 (Fragment) OS=Mus musculus GN=Sept2 PE=1 SV=2
Q91YI0	0.391002219	2	0	0	0	0	0	0	0	Argininosuccinate lyase OS=Mus musculus GN=Asl PE=1 SV=1
Q62418	0.391002219	0	0	0	2	0	0	0	0	Drebrin-like protein OS=Mus musculus GN=Dbnl PE=1 SV=2
E9Q397	0.391002219	0	0	0	2	0	0	0	0	Spectrin beta chain, erythrocytic OS=Mus musculus GN=Sptb PE=1 SV=1
Reverse_sp|Q91ZU6|DYST_MOUSE	0.391002219	0	0	2	0	0	0	0	0	Dystonin OS=Mus musculus GN=Dst PE=1 SV=2
V9GX38	0.391002219	0	0	4	0	0	0	0	0	Protein Gm17190 OS=Mus musculus GN=Gm17190 PE=1 SV=1
Q80SZ7	0.391002219	3	0	0	0	0	0	0	0	Guanine nucleotide-binding protein G(I)/G(S)/G(O) subunit gamma-5 OS=Mus musculus GN=Gng5 PE=1 SV=2
Q8BH57	0.391002219	2	0	0	0	0	0	0	0	WD repeat-containing protein 48 OS=Mus musculus GN=Wdr48 PE=1 SV=1
B7ZNL2	0.391002219	0	0	8	0	0	0	0	0	Nap1l4 protein OS=Mus musculus GN=Nap1l4 PE=1 SV=1
Reverse_sp|F7A4A7|OTOGL_MOUSE	0.391002219	0	3	0	0	0	0	0	0	Otogelin-like protein OS=Mus musculus GN=Otogl PE=1 SV=1
Reverse_sp|Q8VCZ6|SGSM3_MOUSE	0.391002219	0	2	0	0	0	0	0	0	Small G protein signaling modulator 3 OS=Mus musculus GN=Sgsm3 PE=1 SV=1
Q9ESE1	0.391002219	0	0	4	0	0	0	0	0	Lipopolysaccharide-responsive and beige-like anchor protein OS=Mus musculus GN=Lrba PE=1 SV=1
Q3UM33	0.391002219	4	0	0	0	0	0	0	0	Histone deacetylase OS=Mus musculus GN=Hdac3 PE=1 SV=1
D3YVI6	0.391002219	0	0	2	0	0	0	0	0	COP9 signalosome complex subunit 7a (Fragment) OS=Mus musculus GN=Cops7a PE=1 SV=1
V9GX97	0.391002219	0	5	0	0	0	0	0	0	Zinc finger protein 280D (Fragment) OS=Mus musculus GN=Zfp280d PE=1 SV=1
D3YVJ6	0.391002219	0	0	2	0	0	0	0	0	Protein DEK (Fragment) OS=Mus musculus GN=Dek PE=1 SV=1
P70700	0.391002219	0	0	2	0	0	0	0	0	DNA-directed RNA polymerase I subunit RPA2 OS=Mus musculus GN=Polr1b PE=1 SV=2
Q60629	0.391002219	0	0	3	0	0	0	0	0	Ephrin type-A receptor 5 OS=Mus musculus GN=Epha5 PE=1 SV=2
J3QNU1	0.391002219	0	0	4	0	0	0	0	0	Arginyl-tRNA--protein transferase 1 OS=Mus musculus GN=Ate1 PE=1 SV=1
J3QNU6	0.391002219	4	0	0	0	0	0	0	0	Beta-arrestin-1 OS=Mus musculus GN=Arrb1 PE=1 SV=1
Q9JI58	0.391002219	0	2	0	0	0	0	0	0	Retinoic acid early-inducible protein 1-delta OS=Mus musculus GN=Raet1d PE=1 SV=1
Q9ES74	0.391002219	3	0	0	0	0	0	0	0	Serine/threonine-protein kinase Nek7 OS=Mus musculus GN=Nek7 PE=1 SV=1
Reverse_tr|E9QQ96|E9QQ96_MOUSE	0.391002219	0	0	0	2	0	0	0	0	Obscurin OS=Mus musculus GN=Obscn PE=1 SV=2
Reverse_tr|F8WIA4|F8WIA4_MOUSE	0.391002219	0	2	0	0	0	0	0	0	Small G protein-signaling modulator 3 OS=Mus musculus GN=Sgsm3 PE=1 SV=1
Q3UCV8	0.391002219	0	0	4	0	0	0	0	0	Ubiquitin thioesterase otulin OS=Mus musculus GN=Otulin PE=1 SV=1
P11531	0.391002219	0	2	0	0	0	0	0	0	Dystrophin OS=Mus musculus GN=Dmd PE=1 SV=3
Q8R4U0	0.391002219	2	0	0	0	0	0	0	0	Stabilin-2 OS=Mus musculus GN=Stab2 PE=1 SV=1
G3UW85	0.391002219	2	0	0	0	0	0	0	0	Enhancer of rudimentary homolog OS=Mus musculus GN=Erh PE=1 SV=1
Q9JHW4	0.391002219	0	0	4	0	0	0	0	0	Selenocysteine-specific elongation factor OS=Mus musculus GN=Eefsec PE=1 SV=2
D3Z7B5	0.391002219	2	0	0	0	0	0	0	0	Protein C330027C09Rik OS=Mus musculus GN=C330027C09Rik PE=1 SV=1
A0A1D5RM23	0.391002219	4	0	0	0	0	0	0	0	Cleavage and polyadenylation-specificity factor subunit 5 OS=Mus musculus GN=Nudt21 PE=1 SV=1
Q6P9P6	0.391002219	0	2	0	0	0	0	0	0	Kinesin-like protein KIF11 OS=Mus musculus GN=Kif11 PE=1 SV=1
Q64012	0.391002219	0	0	2	0	0	0	0	0	RNA-binding protein Raly OS=Mus musculus GN=Raly PE=1 SV=3
Q80ZX0	0.391002219	0	2	0	0	0	0	0	0	Protein Sec24b OS=Mus musculus GN=Sec24b PE=1 SV=1
A0A1D5RLM8	0.391002219	0	0	0	2	0	0	0	0	Protein Gm11639 OS=Mus musculus GN=Gm11639 PE=4 SV=1
C8YR32	0.391002219	0	7	0	0	0	0	0	0	Lipoxygenase homology domain-containing protein 1 OS=Mus musculus GN=Loxhd1 PE=2 SV=1
E9PVV2	0.391002219	0	0	0	2	0	0	0	0	Transcription factor E2-alpha OS=Mus musculus GN=Tcf3 PE=1 SV=1
E9PVV1	0.391002219	0	0	0	2	0	0	0	0	Transcription factor E2-alpha OS=Mus musculus GN=Tcf3 PE=1 SV=1
D3Z2N9	0.391002219	8	0	0	0	0	0	0	0	Histone deacetylase complex subunit SAP18 OS=Mus musculus GN=Sap18 PE=1 SV=1
F7C528	0.391002219	0	0	5	0	0	0	0	0	Protein Chd3 (Fragment) OS=Mus musculus GN=Chd3 PE=1 SV=1
A0A0G2JG79	0.391002219	0	0	3	0	0	0	0	0	Geranylgeranyl transferase type-2 subunit beta OS=Mus musculus GN=Rabggtb PE=1 SV=1
P53995	0.391002219	0	0	2	0	0	0	0	0	Anaphase-promoting complex subunit 1 OS=Mus musculus GN=Anapc1 PE=1 SV=2
Q8CHW4	0.391002219	3	0	0	0	0	0	0	0	Translation initiation factor eIF-2B subunit epsilon OS=Mus musculus GN=Eif2b5 PE=1 SV=1
A0A0A6YW67	0.391002219	0	0	0	46	0	0	0	0	MCG23377, isoform CRA_b OS=Mus musculus GN=Gm8797 PE=4 SV=1
A0A1D5RLY2	0.391002219	0	2	0	0	0	0	0	0	Protein VAC14 homolog OS=Mus musculus GN=Vac14 PE=1 SV=1
Q9CSP9	0.391002219	0	0	0	3	0	0	0	0	Tetratricopeptide repeat protein 14 OS=Mus musculus GN=Ttc14 PE=1 SV=2
Q91WM3	0.391002219	0	0	2	0	0	0	0	0	U3 small nucleolar RNA-interacting protein 2 OS=Mus musculus GN=Rrp9 PE=1 SV=1
A0A1D5RM88	0.391002219	2	0	0	0	0	0	0	0	Dedicator of cytokinesis protein 9 (Fragment) OS=Mus musculus GN=Dock9 PE=1 SV=1
Q6PB90	0.391002219	0	0	2	0	0	0	0	0	Protocadherin beta-14 OS=Mus musculus GN=Pcdhb14 PE=1 SV=2
Reverse_sp|Q9QYC1|PCX1_MOUSE	0.391002219	2	0	0	0	0	0	0	0	Pecanex-like protein 1 OS=Mus musculus GN=Pcnx1 PE=1 SV=3
E9Q7L7	0.391002219	4	0	0	0	0	0	0	0	Transient receptor potential cation channel subfamily V member 4 OS=Mus musculus GN=Trpv4 PE=1 SV=1
Q06180	0.391002219	0	2	0	0	0	0	0	0	Tyrosine-protein phosphatase non-receptor type 2 OS=Mus musculus GN=Ptpn2 PE=1 SV=2
Q920A5	0.391002219	0	3	0	0	0	0	0	0	Retinoid-inducible serine carboxypeptidase OS=Mus musculus GN=Scpep1 PE=1 SV=2
A0A1D5RLS2	0.391002219	4	0	0	0	0	0	0	0	Cleavage and polyadenylation-specificity factor subunit 5 (Fragment) OS=Mus musculus GN=Nudt21 PE=1 SV=1
A0A1D5RM59	0.391002219	0	0	0	2	0	0	0	0	Protein Tns1 OS=Mus musculus GN=Tns1 PE=1 SV=1
E9PWC8	0.391002219	3	0	0	0	0	0	0	0	Poly(A) polymerase alpha OS=Mus musculus GN=Papola PE=1 SV=1
A6PW47	0.391002219	0	2	0	0	0	0	0	0	Mediator of RNA polymerase II transcription subunit 24 OS=Mus musculus GN=Med24 PE=1 SV=1
E9PWE4	0.391002219	0	0	0	2	0	0	0	0	Transcription factor E2-alpha OS=Mus musculus GN=Tcf3 PE=1 SV=1
E9PWE3	0.391002219	0	0	0	2	0	0	0	0	Transcription factor E2-alpha OS=Mus musculus GN=Tcf3 PE=1 SV=1
E9QA74	0.391002219	3	0	0	0	0	0	0	0	Unconventional myosin-XVIIIa OS=Mus musculus GN=Myo18a PE=1 SV=1
F8WIR1	0.391002219	3	0	0	0	0	0	0	0	Cathepsin D OS=Mus musculus GN=Ctsd PE=1 SV=2
Q3TYK3	0.391002219	0	0	5	0	0	0	0	0	Nuclear factor 1 OS=Mus musculus GN=Nfix PE=1 SV=1
Q8VHZ7	0.391002219	0	3	0	0	0	0	0	0	U3 small nucleolar ribonucleoprotein protein IMP4 OS=Mus musculus GN=Imp4 PE=2 SV=1
D3YZ00	0.391002219	0	0	5	0	0	0	0	0	Nuclear factor 1 (Fragment) OS=Mus musculus GN=Nfix PE=1 SV=1
Q8BG15	0.391002219	0	2	0	0	0	0	0	0	CTD small phosphatase-like protein 2 OS=Mus musculus GN=Ctdspl2 PE=1 SV=1
Q7TQK1	0.391002219	0	0	3	0	0	0	0	0	Integrator complex subunit 7 OS=Mus musculus GN=Ints7 PE=1 SV=1
F6RG68	0.391002219	0	0	0	2	0	0	0	0	F-BAR domain only protein 2 OS=Mus musculus GN=Fcho2 PE=1 SV=2
Q8BWG8	0.391002219	4	0	0	0	0	0	0	0	Beta-arrestin-1 OS=Mus musculus GN=Arrb1 PE=1 SV=1
Q9WVL3	0.391002219	0	0	3	0	0	0	0	0	Solute carrier family 12 member 7 OS=Mus musculus GN=Slc12a7 PE=1 SV=1
F8WJB0	0.391002219	2	0	0	0	0	0	0	0	Mediator of RNA polymerase II transcription subunit 23 OS=Mus musculus GN=Med23 PE=1 SV=1
A0A1D5RLG3	0.391002219	2	0	0	0	0	0	0	0	Rab3 GTPase-activating protein catalytic subunit OS=Mus musculus GN=Rab3gap1 PE=1 SV=1
A2ARZ3	0.391002219	0	0	2	0	0	0	0	0	Fibrous sheath-interacting protein 2 OS=Mus musculus GN=Fsip2 PE=1 SV=3
Reverse_sp|A2AAJ9|OBSCN_MOUSE	0.391002219	0	0	0	2	0	0	0	0	Obscurin OS=Mus musculus GN=Obscn PE=1 SV=2
Q3TQ29	0.391002219	0	0	5	0	0	0	0	0	Pumilio homolog 2 OS=Mus musculus GN=Pum2 PE=1 SV=1
P46737	0.391002219	3	0	0	0	0	0	0	0	Lys-63-specific deubiquitinase BRCC36 OS=Mus musculus GN=Brcc3 PE=1 SV=1
Q920Q4	0.391002219	0	0	2	0	0	0	0	0	Vacuolar protein sorting-associated protein 16 homolog OS=Mus musculus GN=Vps16 PE=1 SV=3
Q9WVJ3	0.391002219	4	0	0	0	0	0	0	0	Carboxypeptidase Q OS=Mus musculus GN=Cpq PE=1 SV=1
A0A0N4SUV6	0.391002219	0	0	4	0	0	0	0	0	Selenocysteine-specific elongation factor OS=Mus musculus GN=Eefsec PE=1 SV=1
Q9CS84	0.391002219	0	0	0	2	0	0	0	0	Neurexin-1 OS=Mus musculus GN=Nrxn1 PE=1 SV=3
Reverse_sp|A6PWY4|WDR76_MOUSE	0.391002219	0	0	2	0	0	0	0	0	WD repeat-containing protein 76 OS=Mus musculus GN=Wdr76 PE=1 SV=1
Reverse_sp|Q5SS00|ZDBF2_MOUSE	0.391002219	6	0	0	0	0	0	0	0	DBF4-type zinc finger-containing protein 2 homolog OS=Mus musculus GN=Zdbf2 PE=2 SV=1
Q91W53	0.391002219	2	0	0	0	0	0	0	0	Golgin subfamily A member 7 OS=Mus musculus GN=Golga7 PE=1 SV=1
D3Z1H7	0.391002219	4	0	0	0	0	0	0	0	Transient receptor potential cation channel subfamily V member 4 OS=Mus musculus GN=Trpv4 PE=1 SV=1
D3Z1H6	0.391002219	4	0	0	0	0	0	0	0	Transient receptor potential cation channel subfamily V member 4 OS=Mus musculus GN=Trpv4 PE=1 SV=1
Q9EPK8	0.391002219	4	0	0	0	0	0	0	0	Transient receptor potential cation channel subfamily V member 4 OS=Mus musculus GN=Trpv4 PE=1 SV=1
Q9D3U0	0.391002219	0	0	2	0	0	0	0	0	Putative tRNA pseudouridine synthase Pus10 OS=Mus musculus GN=Pus10 PE=1 SV=1
Q6PIP5	0.391002219	0	0	2	0	0	0	0	0	NudC domain-containing protein 1 OS=Mus musculus GN=Nudcd1 PE=1 SV=2
O35134	0.391002219	0	2	0	0	0	0	0	0	DNA-directed RNA polymerase I subunit RPA1 OS=Mus musculus GN=Polr1a PE=1 SV=2
A0A0A6YWK7	0.391002219	0	5	0	0	0	0	0	0	Immunoglobulin superfamily member 8 (Fragment) OS=Mus musculus GN=Igsf8 PE=1 SV=1
Q9EQ28	0.391002219	0	0	0	2	0	0	0	0	DNA polymerase delta subunit 3 OS=Mus musculus GN=Pold3 PE=1 SV=2
Q8BMP6	0.391002219	0	0	5	0	0	0	0	0	Golgi resident protein GCP60 OS=Mus musculus GN=Acbd3 PE=1 SV=3
P0CG50	0.391002219	0	0	0	53	0	0	0	0	Polyubiquitin-C OS=Mus musculus GN=Ubc PE=1 SV=2
A2AT37	0.391002219	0	0	3	0	0	0	0	0	Protein Upf2 OS=Mus musculus GN=Upf2 PE=1 SV=1
E9Q0G4	0.391002219	0	0	4	0	0	0	0	0	Protein Gm8909 OS=Mus musculus GN=Gm8909 PE=1 SV=1
G5E884	0.391002219	0	5	0	0	0	0	0	0	Serine/threonine-protein kinase PAK 1 OS=Mus musculus GN=Pak1 PE=1 SV=1
Q91W86	0.391002219	0	2	0	0	0	0	0	0	Vacuolar protein sorting-associated protein 11 homolog OS=Mus musculus GN=Vps11 PE=1 SV=3
E9Q8Y1	0.391002219	0	0	2	0	0	0	0	0	Protein DEK (Fragment) OS=Mus musculus GN=Dek PE=1 SV=1
A0A1D5RMM1	0.391002219	2	0	0	0	0	0	0	0	Dedicator of cytokinesis protein 9 OS=Mus musculus GN=Dock9 PE=1 SV=1
Q91W61	0.391002219	0	0	2	0	0	0	0	0	F-box/LRR-repeat protein 15 OS=Mus musculus GN=Fbxl15 PE=1 SV=2
P60330	0.391002219	0	0	3	0	0	0	0	0	Separin OS=Mus musculus GN=Espl1 PE=1 SV=1
A0A0R4J244	0.391002219	3	0	0	0	0	0	0	0	Poly(A) polymerase alpha OS=Mus musculus GN=Papola PE=1 SV=1
Q8C754	0.391002219	0	0	3	0	0	0	0	0	Vacuolar protein sorting-associated protein 52 homolog OS=Mus musculus GN=Vps52 PE=1 SV=1
Q8BMB3	0.391002219	0	0	4	0	0	0	0	0	Eukaryotic translation initiation factor 4E type 2 OS=Mus musculus GN=Eif4e2 PE=1 SV=1
A0A0N4SUL6	0.391002219	0	3	0	0	0	0	0	0	Ribosomal RNA small subunit methyltransferase NEP1 (Fragment) OS=Mus musculus GN=Emg1 PE=1 SV=1
Q8CGS6	0.391002219	0	2	0	0	0	0	0	0	DNA polymerase theta OS=Mus musculus GN=Polq PE=1 SV=2
Q8BMA3	0.391002219	0	2	0	0	0	0	0	0	Connector enhancer of kinase suppressor of ras 3 OS=Mus musculus GN=Cnksr3 PE=1 SV=1
Q8BUR4	0.391002219	0	3	0	0	0	0	0	0	Dedicator of cytokinesis protein 1 OS=Mus musculus GN=Dock1 PE=1 SV=3
Reverse_sp|Q8K284|TF3C1_MOUSE	0.391002219	2	0	0	0	0	0	0	0	General transcription factor 3C polypeptide 1 OS=Mus musculus GN=Gtf3c1 PE=1 SV=2
Q6IME9	0.391002219	0	0	5	0	0	0	0	0	Keratin, type II cytoskeletal 72 OS=Mus musculus GN=Krt72 PE=3 SV=1
P14430	0.391002219	7	0	0	0	0	0	0	0	H-2 class I histocompatibility antigen, Q8 alpha chain OS=Mus musculus GN=H2-Q8 PE=3 SV=1
A0A0R4J254	0.391002219	0	0	4	0	0	0	0	0	Exportin-4 OS=Mus musculus GN=Xpo4 PE=1 SV=1
Q62168	0.391002219	0	2	0	0	0	0	0	0	Keratin, type I cuticular Ha2 OS=Mus musculus GN=Krt32 PE=1 SV=2
A0A0N4SUH4	0.391002219	2	0	0	0	0	0	0	0	Pumilio homolog 3 OS=Mus musculus GN=Pum3 PE=1 SV=1
Q99ME2	0.391002219	0	3	0	0	0	0	0	0	WD repeat-containing protein 6 OS=Mus musculus GN=Wdr6 PE=1 SV=1
G5E8C3	0.391002219	3	0	0	0	0	0	0	0	G protein-coupled receptor, family C, group 5, member A OS=Mus musculus GN=Gprc5a PE=1 SV=1
P26443	0.391002219	2	0	0	0	0	0	0	0	Glutamate dehydrogenase 1, mitochondrial OS=Mus musculus GN=Glud1 PE=1 SV=1
F6TFF2	0.391002219	0	0	8	0	0	0	0	0	Pituitary tumor-transforming gene 1 protein-interacting protein (Fragment) OS=Mus musculus GN=Pttg1ip PE=1 SV=1
Q91VM5	0.391002219	0	2	0	0	0	0	0	0	RNA binding motif protein, X-linked-like-1 OS=Mus musculus GN=Rbmxl1 PE=1 SV=1
P39749	0.391002219	5	0	0	0	0	0	0	0	Flap endonuclease 1 OS=Mus musculus GN=Fen1 PE=1 SV=1
A0A0R4J1P2	0.391002219	0	0	0	13	0	0	0	0	Tropomyosin alpha-3 chain OS=Mus musculus GN=Tpm3 PE=1 SV=1
P62984	0.391002219	0	0	0	46	0	0	0	0	Ubiquitin-60S ribosomal protein L40 OS=Mus musculus GN=Uba52 PE=1 SV=2
E9PX30	0.391002219	0	0	3	0	0	0	0	0	Mitogen-activated protein kinase kinase kinase kinase OS=Mus musculus GN=Map4k5 PE=1 SV=1
E9Q0S6	0.391002219	0	0	0	2	0	0	0	0	Protein Tns1 OS=Mus musculus GN=Tns1 PE=1 SV=1
Q7TPH6	0.391002219	0	2	0	0	0	0	0	0	E3 ubiquitin-protein ligase MYCBP2 OS=Mus musculus GN=Mycbp2 PE=1 SV=2
Q9WV55	0.391002219	0	0	2	0	0	0	0	0	Vesicle-associated membrane protein-associated protein A OS=Mus musculus GN=Vapa PE=1 SV=2
E9QAX2	0.391002219	3	0	0	0	0	0	0	0	Unconventional myosin-XVIIIa OS=Mus musculus GN=Myo18a PE=1 SV=1
Q8CI43	0.391002219	0	4	0	0	0	0	0	0	Myosin light chain 6B OS=Mus musculus GN=Myl6b PE=1 SV=1
A6H6N9	0.391002219	0	0	0	3	0	0	0	0	MCG123425, isoform CRA_e OS=Mus musculus GN=Ttc14 PE=1 SV=1
Q8BVQ5	0.391002219	0	0	3	0	0	0	0	0	Protein phosphatase methylesterase 1 OS=Mus musculus GN=Ppme1 PE=1 SV=5
E9Q9J0	0.391002219	0	0	0	46	0	0	0	0	Ubiquitin-60S ribosomal protein L40 (Fragment) OS=Mus musculus GN=Uba52 PE=1 SV=1
A2TJV2	0.391002219	0	2	0	0	0	0	0	0	Paralemmin-3 OS=Mus musculus GN=Palm3 PE=1 SV=1
Reverse_tr|E9Q9X1|E9Q9X1_MOUSE	0.391002219	0	0	2	0	0	0	0	0	Dystonin OS=Mus musculus GN=Dst PE=1 SV=2
F8VPN7	0.391002219	2	0	0	0	0	0	0	0	Dedicator of cytokinesis protein 9 OS=Mus musculus GN=Dock9 PE=1 SV=1
Reverse_tr|E9Q421|E9Q421_MOUSE	0.391002219	2	0	0	0	0	0	0	0	GPI-anchor transamidase OS=Mus musculus GN=Pigk PE=1 SV=1
M0QWD1	0.391002219	0	0	4	0	0	0	0	0	Eukaryotic translation initiation factor 4E type 2 (Fragment) OS=Mus musculus GN=Eif4e2 PE=1 SV=1
A0A1D5RMI8	0.391002219	0	2	0	0	0	0	0	0	Alstrom syndrome protein 1 homolog OS=Mus musculus GN=Alms1 PE=1 SV=1
E9QAN2	0.391002219	3	0	0	0	0	0	0	0	Poly(A) polymerase alpha OS=Mus musculus GN=Papola PE=1 SV=1
Q3V3P1	0.391002219	0	2	0	0	0	0	0	0	DNA topoisomerase OS=Mus musculus GN=Top3a PE=1 SV=1
Q6DFV1	0.391002219	0	0	0	2	0	0	0	0	Condensin-2 complex subunit G2 OS=Mus musculus GN=Ncapg2 PE=1 SV=2
A0A0G2JGU4	0.391002219	0	0	3	0	0	0	0	0	Geranylgeranyl transferase type-2 subunit beta (Fragment) OS=Mus musculus GN=Rabggtb PE=1 SV=1
Reverse_tr|A2API5|A2API5_MOUSE	0.391002219	0	0	2	0	0	0	0	0	Low-density lipoprotein receptor-related protein 1B OS=Mus musculus GN=Lrp1b PE=1 SV=2

E9Q108	0.391002219	0	0	0	2	0	0	0	0	Serpin B6 (Fragment) OS=Mus musculus GN=Serpinb6a PE=1 SV=8
A2AKG8	0.391002219	0	0	3	0	0	0	0	0	Focadhesin OS=Mus musculus GN=Focad PE=1 SV=1
A0A0G2JH17	0.391002219	2	0	0	0	0	0	0	0	Integrator complex subunit 1 OS=Mus musculus GN=Ints1 PE=1 SV=1
G5E8S8	0.391002219	0	2	0	0	0	0	0	0	MAGUK p55 subfamily member 7 OS=Mus musculus GN=Mpp7 PE=1 SV=1
E9Q9C3	0.391002219	0	0	2	0	0	0	0	0	Afadin OS=Mus musculus GN=Afdn PE=1 SV=1
A0A0G2JGQ4	0.391002219	3	0	0	0	0	0	0	0	NEDD8 ultimate buster 1 OS=Mus musculus GN=Nub1 PE=1 SV=1
P52432	0.391002219	0	0	2	0	0	0	0	0	DNA-directed RNA polymerases I and III subunit RPAC1 OS=Mus musculus GN=Polr1c PE=1 SV=3
Q9Z2T6	0.391002219	0	74	0	0	0	0	0	0	Keratin, type II cuticular Hb5 OS=Mus musculus GN=Krt85 PE=1 SV=2
Reverse_sp|Q61468|MSLN_MOUSE	0.391002219	5	0	0	0	0	0	0	0	Mesothelin OS=Mus musculus GN=Msln PE=1 SV=1
Q8BTZ4	0.391002219	0	0	2	0	0	0	0	0	Anaphase-promoting complex subunit 5 OS=Mus musculus GN=Anapc5 PE=1 SV=1
A0A0G2JE97	0.391002219	0	0	0	3	0	0	0	0	MCG123425, isoform CRA_a OS=Mus musculus GN=Ttc14 PE=1 SV=1
Reverse_tr|D3YVU1|D3YVU1_MOUSE	0.391002219	0	2	0	0	0	0	0	0	Ubiquitinyl hydrolase 1 OS=Mus musculus GN=Usp16 PE=1 SV=1
Q9CQT0	0.391002219	0	0	4	0	0	0	0	0	tRNA(His) guanylyltransferase OS=Mus musculus GN=Thg1l PE=1 SV=1
Q5U452	0.391002219	2	0	0	0	0	0	0	0	MCG14616 OS=Mus musculus GN=2810408M09Rik PE=1 SV=1
D3Z4N9	0.391002219	2	0	0	0	0	0	0	0	Fatty acyl-CoA reductase (Fragment) OS=Mus musculus GN=Far1 PE=1 SV=8
A2AU62	0.391002219	0	0	2	0	0	0	0	0	RNA-binding protein Raly (Fragment) OS=Mus musculus GN=Raly PE=1 SV=1
Q8BTW3	0.391002219	0	0	2	0	0	0	0	0	Exosome complex component MTR3 OS=Mus musculus GN=Exosc6 PE=1 SV=1
E9Q614	0.391002219	0	0	5	0	0	0	0	0	Protein Chd3 OS=Mus musculus GN=Chd3 PE=1 SV=1
Q5DTJ9	0.391002219	0	2	0	0	0	0	0	0	Myopalladin OS=Mus musculus GN=Mypn PE=1 SV=2
Reverse_sp|Q03517|SCG2_MOUSE	0.391002219	0	2	0	0	0	0	0	0	Secretogranin-2 OS=Mus musculus GN=Scg2 PE=1 SV=1
O35685	0.391002219	0	0	4	0	0	0	0	0	Nuclear migration protein nudC OS=Mus musculus GN=Nudc PE=1 SV=1
Q8BLN5	0.391002219	0	0	4	0	0	0	0	0	Lanosterol synthase OS=Mus musculus GN=Lss PE=1 SV=2
A2ATQ5	0.391002219	0	0	2	0	0	0	0	0	Anaphase-promoting complex subunit 1 OS=Mus musculus GN=Anapc1 PE=1 SV=1
F8WHB1	0.391002219	0	4	0	0	0	0	0	0	Calcium-transporting ATPase OS=Mus musculus GN=Atp2b2 PE=1 SV=1
Reverse_sp|Q9CXY9|GPI8_MOUSE	0.391002219	2	0	0	0	0	0	0	0	GPI-anchor transamidase OS=Mus musculus GN=Pigk PE=1 SV=2
Q7TNV0	0.391002219	0	0	2	0	0	0	0	0	Protein DEK OS=Mus musculus GN=Dek PE=1 SV=1
A0A0G2JEB3	0.391002219	0	0	2	0	0	0	0	0	Farnesyl pyrophosphate synthase (Fragment) OS=Mus musculus GN=Fdps PE=4 SV=4
A0A0R4J124	0.391002219	0	2	0	0	0	0	0	0	SRSF protein kinase 2 OS=Mus musculus GN=Srpk2 PE=1 SV=1
P70257	0.391002219	0	0	5	0	0	0	0	0	Nuclear factor 1 X-type OS=Mus musculus GN=Nfix PE=1 SV=2
O35638	0.391002219	0	0	3	0	0	0	0	0	Cohesin subunit SA-2 OS=Mus musculus GN=Stag2 PE=1 SV=3
F6YAR3	0.391002219	0	2	0	0	0	0	0	0	Transducin beta-like protein 3 (Fragment) OS=Mus musculus GN=Tbl3 PE=1 SV=1
E9Q5F6	0.391002219	0	0	0	47	0	0	0	0	Polyubiquitin-C (Fragment) OS=Mus musculus GN=Ubc PE=4 SV=1
A0A0G2JEA5	0.391002219	0	0	2	0	0	0	0	0	Protein Gm43738 OS=Mus musculus GN=Gm43738 PE=4 SV=1
P70275	0.391002219	0	0	2	0	0	0	0	0	Semaphorin-3E OS=Mus musculus GN=Sema3e PE=1 SV=3
Q543M9	0.391002219	2	0	0	0	0	0	0	0	MCG14605, isoform CRA_c OS=Mus musculus GN=Trp53rkb PE=1 SV=1
E9QNY8	0.391002219	3	0	0	0	0	0	0	0	Sacsin OS=Mus musculus GN=Sacs PE=1 SV=1
A0A0H2UH27	0.391002219	0	2	0	0	0	0	0	0	Fragile X mental retardation syndrome-related protein 1 OS=Mus musculus GN=Fxr1 PE=1 SV=1
Q9CQI6	0.391002219	3	0	0	0	0	0	0	0	Coactosin-like protein OS=Mus musculus GN=Cotl1 PE=1 SV=3
Q9CQI7	0.391002219	3	0	0	0	0	0	0	0	U2 small nuclear ribonucleoprotein B'' OS=Mus musculus GN=Snrpb2 PE=1 SV=1
Q9D312	0.391002219	0	0	2	0	0	0	0	0	Keratin, type I cytoskeletal 20 OS=Mus musculus GN=Krt20 PE=1 SV=1
A0A140LIW6	0.391002219	0	0	0	2	0	0	0	0	DNA polymerase delta subunit 3 (Fragment) OS=Mus musculus GN=Pold3 PE=1 SV=1
A2ATI8	0.391002219	0	0	4	0	0	0	0	0	Golgi reassembly-stacking protein 2 OS=Mus musculus GN=Gorasp2 PE=1 SV=1
A2ATI6	0.391002219	0	0	4	0	0	0	0	0	Golgi reassembly stacking protein 2, isoform CRA_c OS=Mus musculus GN=Gorasp2 PE=1 SV=1
A2ATI9	0.391002219	0	0	4	0	0	0	0	0	Golgi reassembly stacking protein 2, isoform CRA_d OS=Mus musculus GN=Gorasp2 PE=1 SV=1
Reverse_sp|O08638|MYH11_MOUSE	0.391002219	2	0	0	0	0	0	0	0	Myosin-11 OS=Mus musculus GN=Myh11 PE=1 SV=1
Reverse_tr|F7ABZ6|F7ABZ6_MOUSE	0.391002219	0	0	2	0	0	0	0	0	Protein Dnah10 OS=Mus musculus GN=Dnah10 PE=1 SV=2
E9QP19	0.391002219	0	2	0	0	0	0	0	0	E3 ubiquitin-protein ligase TRIM33 OS=Mus musculus GN=Trim33 PE=1 SV=1
Q61686	0.391002219	2	0	0	0	0	0	0	0	Chromobox protein homolog 5 OS=Mus musculus GN=Cbx5 PE=1 SV=1
B1ATJ5	0.391002219	0	2	0	0	0	0	0	0	Keratin, type I cuticular Ha2 OS=Mus musculus GN=Krt32 PE=1 SV=1
Q3TWF7	0.391002219	0	0	2	0	0	0	0	0	Anaphase-promoting complex subunit 5 OS=Mus musculus GN=Anapc5 PE=1 SV=1
Q3UR91	0.391002219	0	0	5	0	0	0	0	0	Pumilio homolog 2 OS=Mus musculus GN=Pum2 PE=1 SV=1
Q99M74	0.391002219	0	3	0	0	0	0	0	0	Keratin, type II cuticular Hb2 OS=Mus musculus GN=Krt82 PE=1 SV=2
Q8CH18	0.391002219	0	0	4	0	0	0	0	0	Cell division cycle and apoptosis regulator protein 1 OS=Mus musculus GN=Ccar1 PE=1 SV=1
P99026	0.391002219	2	0	0	0	0	0	0	0	Proteasome subunit beta type-4 OS=Mus musculus GN=Psmb4 PE=1 SV=1
Q9CZX7	0.391002219	0	3	0	0	0	0	0	0	Type 2 phosphatidylinositol 4,5-bisphosphate 4-phosphatase OS=Mus musculus GN=Tmem55a PE=1 SV=1
D3Z440	0.391002219	0	0	2	0	0	0	0	0	COP9 signalosome complex subunit 7a (Fragment) OS=Mus musculus GN=Cops7a PE=1 SV=8
F6VQH5	0.391002219	4	0	0	0	0	0	0	0	Heterogeneous nuclear ribonucleoprotein D-like (Fragment) OS=Mus musculus GN=Hnrnpdl PE=1 SV=1
Reverse_sp|Q9JI18|LRP1B_MOUSE	0.391002219	0	0	2	0	0	0	0	0	Low-density lipoprotein receptor-related protein 1B OS=Mus musculus GN=Lrp1b PE=1 SV=1
Q3UQN2	0.391002219	0	0	0	2	0	0	0	0	F-BAR domain only protein 2 OS=Mus musculus GN=Fcho2 PE=1 SV=1
A0A0G2JE03	0.391002219	0	0	2	0	0	0	0	0	Anaphase-promoting complex subunit 5 OS=Mus musculus GN=Anapc5 PE=1 SV=1
A0A0G2JDM7	0.391002219	0	0	2	0	0	0	0	0	Anaphase-promoting complex subunit 5 OS=Mus musculus GN=Anapc5 PE=1 SV=1
S4R1Y5	0.391002219	0	2	0	0	0	0	0	0	Ribosome production factor 2 homolog (Fragment) OS=Mus musculus GN=Rpf2 PE=1 SV=1
Q3UA06	0.391002219	0	0	4	0	0	0	0	0	Pachytene checkpoint protein 2 homolog OS=Mus musculus GN=Trip13 PE=1 SV=1
Q9CZQ6	0.391002219	0	2	0	0	0	0	0	0	Retinoic acid early-inducible protein 1-epsilon OS=Mus musculus GN=Raet1e PE=1 SV=1
Q6NSU3	0.391002219	0	0	3	0	0	0	0	0	Glycosyltransferase 8 domain-containing protein 1 OS=Mus musculus GN=Glt8d1 PE=1 SV=1
A0A0G2JDK3	0.391002219	0	0	3	0	0	0	0	0	Geranylgeranyl transferase type-2 subunit beta (Fragment) OS=Mus musculus GN=Rabggtb PE=1 SV=1
Reverse_tr|F6YMV1|F6YMV1_MOUSE	0.391002219	3	0	0	0	0	0	0	0	Angiomotin (Fragment) OS=Mus musculus GN=Amot PE=1 SV=1
Q9DBT6	0.391002219	0	0	0	2	0	0	0	0	Protein Tns1 OS=Mus musculus GN=Tns1 PE=1 SV=1
Q497I4	0.391002219	0	29	0	0	0	0	0	0	Keratin, type I cuticular Ha5 OS=Mus musculus GN=Krt35 PE=1 SV=1
Q9DBT5	0.391002219	0	2	0	0	0	0	0	0	AMP deaminase 2 OS=Mus musculus GN=Ampd2 PE=1 SV=1
Reverse_tr|Q4W8U9|Q4W8U9_MOUSE	0.391002219	0	2	0	0	0	0	0	0	Scg2 protein OS=Mus musculus GN=Scg2 PE=1 SV=1
F6VJC5	0.391002219	0	2	0	0	0	0	0	0	Protein Sec24b (Fragment) OS=Mus musculus GN=Sec24b PE=1 SV=1
F8WIE5	0.391002219	3	0	0	0	0	0	0	0	E3 ubiquitin-protein ligase HECTD1 OS=Mus musculus GN=Hectd1 PE=1 SV=1
A0A140LHY7	0.391002219	0	0	0	2	0	0	0	0	DNA polymerase delta subunit 3 OS=Mus musculus GN=Pold3 PE=1 SV=1
G3X963	0.391002219	0	0	0	2	0	0	0	0	ATPase family AAA domain-containing protein 2 OS=Mus musculus GN=Atad2 PE=1 SV=1
A0A0R4J079	0.391002219	0	0	5	0	0	0	0	0	Acyl-Coenzyme A binding domain containing 3, isoform CRA_b OS=Mus musculus GN=Acbd3 PE=1 SV=1
K3W4P2	0.391002219	2	0	0	0	0	0	0	0	Integrator complex subunit 1 OS=Mus musculus GN=Ints1 PE=1 SV=2
Q9DBR3	0.391002219	2	0	0	0	0	0	0	0	Armadillo repeat-containing protein 8 OS=Mus musculus GN=Armc8 PE=1 SV=2
Q9QYX7	0.391002219	0	2	0	0	0	0	0	0	Protein piccolo OS=Mus musculus GN=Pclo PE=1 SV=4
Reverse_sp|A2ARZ3|FSIP2_MOUSE	0.391002219	0	2	0	0	0	0	0	0	Fibrous sheath-interacting protein 2 OS=Mus musculus GN=Fsip2 PE=1 SV=3
A8C756	0.391002219	0	0	0	2	0	0	0	0	Thyroid adenoma-associated protein homolog OS=Mus musculus GN=Thada PE=1 SV=1
G3X8X7	0.391002219	0	0	2	0	0	0	0	0	Vacuolar protein sorting 16 (Yeast) OS=Mus musculus GN=Vps16 PE=1 SV=1
Q9CQ80	0.391002219	2	0	0	0	0	0	0	0	Vacuolar protein-sorting-associated protein 25 OS=Mus musculus GN=Vps25 PE=1 SV=1
Q9JLZ6	0.391002219	4	0	0	0	0	0	0	0	Hypermethylated in cancer 2 protein OS=Mus musculus GN=Hic2 PE=2 SV=4
A0A0G2JF90	0.391002219	0	0	0	3	0	0	0	0	Tetratricopeptide repeat protein 14 (Fragment) OS=Mus musculus GN=Ttc14 PE=1 SV=1
A0A0G2JEX0	0.391002219	0	0	0	3	0	0	0	0	MCG123425, isoform CRA_f OS=Mus musculus GN=Ttc14 PE=1 SV=1
Q3UGX2	0.391002219	0	0	0	2	0	0	0	0	Putative uncharacterized protein OS=Mus musculus GN=Sptb PE=1 SV=1
K3W4L0	0.391002219	3	0	0	0	0	0	0	0	Unconventional myosin-XVIIIa OS=Mus musculus GN=Myo18a PE=1 SV=1
Q99KH8	0.391002219	0	2	0	0	0	0	0	0	Serine/threonine-protein kinase 24 OS=Mus musculus GN=Stk24 PE=1 SV=1
Reverse_tr|E9QP01|E9QP01_MOUSE	0.391002219	0	2	0	0	0	0	0	0	Protein capicua homolog OS=Mus musculus GN=Cic PE=1 SV=2
Reverse_sp|Q8BKN5|GCP5_MOUSE	0.391002219	0	0	2	0	0	0	0	0	Gamma-tubulin complex component 5 OS=Mus musculus GN=Tubgcp5 PE=2 SV=2
Q99KG5	0.391002219	3	0	0	0	0	0	0	0	Lipolysis-stimulated lipoprotein receptor OS=Mus musculus GN=Lsr PE=1 SV=1
D3Z3C0	0.391002219	0	0	0	7	0	0	0	0	Septin-2 (Fragment) OS=Mus musculus GN=Sept2 PE=1 SV=1
Reverse_tr|Q8BL63|Q8BL63_MOUSE	0.391002219	2	0	0	0	0	0	0	0	GPI-anchor transamidase OS=Mus musculus GN=Pigk PE=1 SV=1
P70372	0.391002219	0	0	4	0	0	0	0	0	ELAV-like protein 1 OS=Mus musculus GN=Elavl1 PE=1 SV=2
A2AE27	0.391002219	0	2	0	0	0	0	0	0	AMP deaminase 2 OS=Mus musculus GN=Ampd2 PE=1 SV=1
A0A087WQ94	0.391002219	0	0	0	2	0	0	0	0	Protein Tns1 (Fragment) OS=Mus musculus GN=Tns1 PE=1 SV=1
P15508	0.391002219	0	0	0	2	0	0	0	0	Spectrin beta chain, erythrocytic OS=Mus musculus GN=Sptb PE=1 SV=4
E9PUH7	0.391002219	0	0	5	0	0	0	0	0	Nuclear factor 1 OS=Mus musculus GN=Nfix PE=1 SV=1
A0A0R4J011	0.391002219	0	0	0	2	0	0	0	0	Transcription factor E2-alpha OS=Mus musculus GN=Tcf3 PE=1 SV=1
E9PUJ6	0.391002219	0	2	0	0	0	0	0	0	E3 ubiquitin-protein ligase MYCBP2 OS=Mus musculus GN=Mycbp2 PE=1 SV=1
A0A087WPT7	0.391002219	3	0	0	0	0	0	0	0	Phosphatidylinositol 3,4,5-trisphosphate 5-phosphatase 2 (Fragment) OS=Mus musculus GN=Inppl1 PE=1 SV=1
P62748	0.391002219	5	0	0	0	0	0	0	0	Hippocalcin-like protein 1 OS=Mus musculus GN=Hpcal1 PE=1 SV=2
Q9JMH9	0.391002219	3	0	0	0	0	0	0	0	Unconventional myosin-XVIIIa OS=Mus musculus GN=Myo18a PE=1 SV=2
Q8BKX6	0.391002219	0	0	2	0	0	0	0	0	Serine/threonine-protein kinase SMG1 OS=Mus musculus GN=Smg1 PE=1 SV=3
Reverse_tr|Q6DFZ1|Q6DFZ1_MOUSE	0.391002219	0	0	5	0	0	0	0	0	Golgi-specific brefeldin A-resistance factor 1 OS=Mus musculus GN=Gbf1 PE=1 SV=1
A0A0J9YU66	0.391002219	0	2	0	0	0	0	0	0	C-terminal binding protein 1, isoform CRA_a OS=Mus musculus GN=Ctbp1 PE=1 SV=1
A2AEJ8	0.391002219	3	0	0	0	0	0	0	0	Cleavage stimulation factor subunit 2 (Fragment) OS=Mus musculus GN=Cstf2 PE=1 SV=1
F6QKD2	0.391002219	2	0	0	0	0	0	0	0	Probable ATP-dependent RNA helicase DDX47 (Fragment) OS=Mus musculus GN=Ddx47 PE=1 SV=1
Q9CQC8	0.391002219	0	3	0	0	0	0	0	0	Maspardin OS=Mus musculus GN=Spg21 PE=1 SV=1
A2AEK1	0.391002219	3	0	0	0	0	0	0	0	AlphaCstF-64 variant 4 OS=Mus musculus GN=Cstf2 PE=1 SV=1
Q9CQC9	0.391002219	0	0	6	0	0	0	0	0	GTP-binding protein SAR1b OS=Mus musculus GN=Sar1b PE=1 SV=1
Reverse_tr|Z4YJE4|Z4YJE4_MOUSE	0.391002219	0	0	0	2	0	0	0	0	Obscurin OS=Mus musculus GN=Obscn PE=1 SV=1
G3X9H5	0.391002219	2	0	0	0	0	0	0	0	Huntingtin OS=Mus musculus GN=Htt PE=1 SV=1
G3X9H1	0.391002219	0	0	4	0	0	0	0	0	Eukaryotic translation initiation factor 4E member 2, isoform CRA_a OS=Mus musculus GN=Eif4e2 PE=1 SV=1
A0A087WQS0	0.391002219	0	0	0	2	0	0	0	0	Protein Tns1 OS=Mus musculus GN=Tns1 PE=1 SV=1
A0A0N4SVT1	0.391002219	2	0	0	0	0	0	0	0	Probable ATP-dependent RNA helicase DDX47 (Fragment) OS=Mus musculus GN=Ddx47 PE=1 SV=1
A0A0R4J0J6	0.391002219	0	0	2	0	0	0	0	0	THO complex subunit 5 homolog OS=Mus musculus GN=Thoc5 PE=1 SV=1
Q9QZQ1	0.391002219	0	0	2	0	0	0	0	0	Afadin OS=Mus musculus GN=Afdn PE=1 SV=3
G3X9J6	0.391002219	3	0	0	0	0	0	0	0	5'-nucleotidase, cytosolic II, isoform CRA_b OS=Mus musculus GN=Nt5c2 PE=1 SV=1
A0A0R4J0E4	0.391002219	0	0	3	0	0	0	0	0	Integrator complex subunit 7 OS=Mus musculus GN=Ints7 PE=1 SV=1
Reverse_tr|D3YYQ8|D3YYQ8_MOUSE	0.391002219	0	0	2	0	0	0	0	0	Protein Dnah10 OS=Mus musculus GN=Dnah10 PE=1 SV=2
E9PVD3	0.391002219	0	2	0	0	0	0	0	0	Protocadherin-16 OS=Mus musculus GN=Dchs1 PE=1 SV=1
Q3UHH0	0.391002219	0	4	0	0	0	0	0	0	Calcium-transporting ATPase OS=Mus musculus GN=Atp2b2 PE=1 SV=1
Q3TVF4	0.391002219	0	0	3	0	0	0	0	0	Geranylgeranyl transferase type-2 subunit beta OS=Mus musculus GN=Rabggtb PE=1 SV=1
A0A087WQM0	0.391002219	0	0	0	2	0	0	0	0	Protein Tns1 OS=Mus musculus GN=Tns1 PE=1 SV=1
P11103	0.391002219	0	0	0	2	0	0	0	0	Poly [ADP-ribose] polymerase 1 OS=Mus musculus GN=Parp1 PE=1 SV=3
Q9CQF3	0.391002219	4	0	0	0	0	0	0	0	Cleavage and polyadenylation specificity factor subunit 5 OS=Mus musculus GN=Nudt21 PE=1 SV=1
P79568	0.391002219	7	0	0	0	0	0	0	0	Class Ib MHC antigen Qa-2 OS=Mus musculus GN=H2-Q6 PE=3 SV=1
Q61584	0.391002219	0	2	0	0	0	0	0	0	Fragile X mental retardation syndrome-related protein 1 OS=Mus musculus GN=Fxr1 PE=1 SV=2
A0A1C7ZN10	0.391002219	3	0	0	0	0	0	0	0	Unconventional myosin-XVIIIa OS=Mus musculus GN=Myo18a PE=1 SV=1
Q99KN2	0.391002219	0	0	2	0	0	0	0	0	Probable cytosolic iron-sulfur protein assembly protein CIAO1 OS=Mus musculus GN=Ciao1 PE=1 SV=1
Q99KN1	0.391002219	3	0	0	0	0	0	0	0	Arrestin domain-containing protein 1 OS=Mus musculus GN=Arrdc1 PE=1 SV=2
E9Q6X2	0.391002219	0	0	0	2	0	0	0	0	Serpin B6 (Fragment) OS=Mus musculus GN=Serpinb6a PE=1 SV=1
A0A0R4J0C8	0.391002219	0	0	2	0	0	0	0	0	3'-5' exoribonuclease 1 OS=Mus musculus GN=Eri1 PE=1 SV=1
A0A0G2JEP0	0.391002219	0	2	0	0	0	0	0	0	Fragile X mental retardation syndrome-related protein 1 OS=Mus musculus GN=Fxr1 PE=1 SV=1
A0A0R4J0B4	0.391002219	0	3	0	0	0	0	0	0	Cytidine monophospho-N-acetylneuraminic acid synthetase OS=Mus musculus GN=Cmas PE=1 SV=1
Q3V1L4	0.391002219	3	0	0	0	0	0	0	0	Cytosolic purine 5'-nucleotidase OS=Mus musculus GN=Nt5c2 PE=1 SV=2
A0A0G2JF29	0.391002219	0	0	3	0	0	0	0	0	Geranylgeranyl transferase type-2 subunit beta OS=Mus musculus GN=Rabggtb PE=1 SV=1
Q99KK2	0.391002219	0	3	0	0	0	0	0	0	N-acylneuraminate cytidylyltransferase OS=Mus musculus GN=Cmas PE=1 SV=2
F8WI95	0.391002219	5	0	0	0	0	0	0	0	Methylthioribose-1-phosphate isomerase (Fragment) OS=Mus musculus GN=Mri1 PE=1 SV=1
Q8BKT7	0.391002219	0	0	2	0	0	0	0	0	THO complex subunit 5 homolog OS=Mus musculus GN=Thoc5 PE=1 SV=2
Q9CZ04	0.391002219	0	0	2	0	0	0	0	0	COP9 signalosome complex subunit 7a OS=Mus musculus GN=Cops7a PE=1 SV=2
Q8BKS9	0.391002219	2	0	0	0	0	0	0	0	Pumilio homolog 3 OS=Mus musculus GN=Pum3 PE=1 SV=2
G3X9T5	0.391002219	0	0	0	3	0	0	0	0	MCG123425, isoform CRA_b OS=Mus musculus GN=Ttc14 PE=1 SV=3
P84089	0.391002219	2	0	0	0	0	0	0	0	Enhancer of rudimentary homolog OS=Mus musculus GN=Erh PE=1 SV=1
Q8VEJ4	0.391002219	0	0	0	2	0	0	0	0	Notchless protein homolog 1 OS=Mus musculus GN=Nle1 PE=1 SV=4
Q8CFE6	0.391002219	0	0	2	0	0	0	0	0	Sodium-coupled neutral amino acid transporter 2 OS=Mus musculus GN=Slc38a2 PE=1 SV=1
P84075	0.391002219	5	0	0	0	0	0	0	0	Neuron-specific calcium-binding protein hippocalcin OS=Mus musculus GN=Hpca PE=1 SV=2

Table S2 Click here to access/download;Table;Table S2.pdf

Table S2. Only 748 proteins fractions with significance P-value ≤ 0.05 are further analyzed.

Accession	T-test p-value	Spectral count for ciliated EV (WT)				Spectral count for non-ciliated EV (KO)				Description
		Sample1	Sample2	Sample3	Sample4	Sample1	Sample2	Sample3	Sample4	
A2ASQ1	1.2973E-05	40	30	42	5	91	71	63	61	Agrin OS=Mus musculus GN=Agrn PE=1 SV=1
Q61490	1.9445E-05	0	0	0	0	29	24	17	27	CD166 antigen OS=Mus musculus GN=Alcam PE=1 SV=3
E9Q3Q6	1.9445E-05	0	0	0	0	29	24	17	27	CD166 antigen OS=Mus musculus GN=Alcam PE=1 SV=1
E9Q4G8	1.9445E-05	0	0	0	0	29	24	17	27	CD166 antigen OS=Mus musculus GN=Alcam PE=1 SV=1
Q8VHX6	3.9976E-05	33	28	33	10	199	155	116	162	Filamin-C OS=Mus musculus GN=Flnc PE=1 SV=3
Q5SUW3	7.1174E-05	0	0	0	0	6	5	4	5	Growth factor receptor-bound protein 10 OS=Mus musculus GN=Grb10 PE=1 SV=1
Q60760	7.1174E-05	0	0	0	0	6	5	4	5	Growth factor receptor-bound protein 10 OS=Mus musculus GN=Grb10 PE=1 SV=2
P26618	7.3818E-05	0	0	0	0	13	10	7	10	Platelet-derived growth factor receptor alpha OS=Mus musculus GN=Pdgfra PE=1 SV=3
O35295	9.8879E-05	0	0	0	0	7	6	5	7	Transcriptional activator protein Pur-beta OS=Mus musculus GN=Purb PE=1 SV=3
O35474	1.0574E-04	0	0	0	0	15	11	10	13	EGF-like repeat and discoidin I-like domain-containing protein 3 OS=Mus musculus GN=Edil3 PE=1 SV=2
Q8C4U8	1.0574E-04	0	0	0	0	15	11	10	13	EGF-like repeat and discoidin I-like domain-containing protein 3 OS=Mus musculus GN=Edil3 PE=1 SV=1
Q80YX1	1.2499E-04	0	0	0	0	71	62	49	75	Tenascin OS=Mus musculus GN=Tnc PE=1 SV=1
P08775	1.3897E-04	34	31	29	10	6	6	2	7	DNA-directed RNA polymerase II subunit RPB1 OS=Mus musculus GN=Polr2a PE=1 SV=3
A0A0R4J0V5	1.3897E-04	34	31	29	10	6	6	2	7	DNA-directed RNA polymerase subunit OS=Mus musculus GN=Polr2a PE=1 SV=1
D3YUQ9	1.6781E-04	2	0	5	0	12	7	9	9	Elongation factor 1-delta (Fragment) OS=Mus musculus GN=Eef1d PE=1 SV=8
P13020	1.9109E-04	21	15	18	5	46	38	29	43	Gelsolin OS=Mus musculus GN=Gsn PE=1 SV=3
Q9QXS1	2.0287E-04	21	16	21	5	192	148	109	138	Plectin OS=Mus musculus GN=Plec PE=1 SV=3
E9Q3W4	2.0309E-04	21	15	20	5	191	148	108	138	Plectin OS=Mus musculus GN=Plec PE=1 SV=1
Q05DV1	2.0416E-04	24	19	25	7	0	0	0	0	NADPH--cytochrome P450 reductase OS=Mus musculus GN=Por PE=1 SV=1
Q04857	2.0775E-04	0	0	0	0	23	19	14	17	Collagen alpha-1(VI) chain OS=Mus musculus GN=Col6a1 PE=1 SV=1
Q6P1J1	2.5750E-04	0	0	0	0	19	18	12	16	Crmp1 protein OS=Mus musculus GN=Crmp1 PE=1 SV=1
E9Q1T9	2.5822E-04	73	50	56	17	24	19	13	22	Exportin-2 OS=Mus musculus GN=Cse1l PE=1 SV=1
E9PX23	2.8060E-04	8	4	4	2	3	0	0	3	Metastasis-associated protein MTA1 OS=Mus musculus GN=Mta1 PE=1 SV=1
Q8K4B0	2.8060E-04	8	4	4	2	3	0	0	3	Metastasis-associated protein MTA1 OS=Mus musculus GN=Mta1 PE=1 SV=1
F8WHY8	2.8060E-04	8	4	4	2	3	0	0	3	Metastasis-associated protein MTA1 OS=Mus musculus GN=Mta1 PE=1 SV=1
Q9WV92	2.8565E-04	0	4	0	0	48	51	29	48	Band 4.1-like protein 3 OS=Mus musculus GN=Epb41l3 PE=1 SV=1
Q62351	2.9072E-04	106	72	82	24	5	5	4	10	Transferrin receptor protein 1 OS=Mus musculus GN=Tfrc PE=1 SV=1
M0QWP1	3.0775E-04	44	32	44	5	98	72	66	69	Agrin OS=Mus musculus GN=Agrn PE=1 SV=1
A0A1B0GR11	3.1153E-04	24	23	22	7	0	0	0	0	Transaldolase OS=Mus musculus GN=Taldo1 PE=1 SV=1
Q93092	3.1153E-04	24	23	22	7	0	0	0	0	Transaldolase OS=Mus musculus GN=Taldo1 PE=1 SV=2
P23116	3.3446E-04	69	48	55	19	55	36	31	49	Eukaryotic translation initiation factor 3 subunit A OS=Mus musculus GN=Eif3a PE=1 SV=5
P51655	3.9230E-04	0	0	0	0	35	22	20	25	Glypican-4 OS=Mus musculus GN=Gpc4 PE=1 SV=2
Q9CYN9	4.1823E-04	17	15	18	6	0	0	0	0	Renin receptor OS=Mus musculus GN=Atp6ap2 PE=1 SV=2
D3Z5N9	4.7416E-04	10	8	8	3	0	0	0	3	MCG49198 OS=Mus musculus GN=Gm5449 PE=4 SV=1
D3Z1J5	5.0864E-04	0	0	0	0	4	3	3	4	Palladin (Fragment) OS=Mus musculus GN=Palld PE=1 SV=1
Q9ERK4	5.4543E-04	77	53	58	17	24	19	13	22	Exportin-2 OS=Mus musculus GN=Cse1l PE=1 SV=1
P62897	5.4606E-04	0	0	0	0	17	13	8	12	Cytochrome c, somatic OS=Mus musculus GN=Cycs PE=1 SV=2
P43274	5.5510E-04	42	25	30	11	2	2	0	0	Histone H1.4 OS=Mus musculus GN=Hist1h1e PE=1 SV=2
P28653	5.7413E-04	0	0	0	0	35	21	19	31	Biglycan OS=Mus musculus GN=Bgn PE=1 SV=1
A0A1D5RMD8	5.8640E-04	9	7	7	3	0	0	0	4	CCR4-NOT transcription complex subunit 1 OS=Mus musculus GN=Cnot1 PE=1 SV=1
Q9WTK5	6.3792E-04	0	0	0	0	4	3	2	4	Nuclear factor NF-kappa-B p100 subunit OS=Mus musculus GN=Nfkb2 PE=1 SV=1
Q8BFY9	6.3906E-04	32	22	24	11	10	7	6	14	Transportin-1 OS=Mus musculus GN=Tnpo1 PE=1 SV=2
Q9EQH3	6.4096E-04	50	45	46	15	31	30	23	35	Vacuolar protein sorting-associated protein 35 OS=Mus musculus GN=Vps35 PE=1 SV=1
Q62009	6.4439E-04	0	0	0	0	37	24	17	25	Periostin OS=Mus musculus GN=Postn PE=1 SV=2
P97927	6.5484E-04	0	0	0	0	34	29	17	34	Laminin subunit alpha-4 OS=Mus musculus GN=Lama4 PE=1 SV=2
Q61656	6.9064E-04	52	37	45	11	13	9	5	8	Probable ATP-dependent RNA helicase DDX5 OS=Mus musculus GN=Ddx5 PE=1 SV=2
Q99J09	7.1787E-04	13	11	12	5	4	4	4	7	Methylosome protein 50 OS=Mus musculus GN=Wdr77 PE=1 SV=1
Q9DAW6	7.3271E-04	32	29	28	8	23	21	14	15	U4/U6 small nuclear ribonucleoprotein Prp4 OS=Mus musculus GN=Prpf4 PE=1 SV=1
E9QA16	7.4994E-04	0	0	0	0	29	24	22	23	Protein Cald1 OS=Mus musculus GN=Cald1 PE=1 SV=1
J3KMQ2	7.5028E-04	44	34	42	14	19	21	16	22	Protein Gm5422 OS=Mus musculus GN=Gm5422 PE=4 SV=1
P14824	7.8030E-04	123	76	95	31	212	169	122	199	Annexin A6 OS=Mus musculus GN=Anxa6 PE=1 SV=3
Q80W65	7.9849E-04	15	10	13	5	0	0	0	0	Proprotein convertase subtilisin/kexin type 9 OS=Mus musculus GN=Pcsk9 PE=1 SV=2
Q8VCQ8	8.0820E-04	0	0	0	0	29	23	22	23	Caldesmon 1 OS=Mus musculus GN=Cald1 PE=1 SV=1
P14094	8.1480E-04	18	20	22	7	5	8	7	7	Sodium/potassium-transporting ATPase subunit beta-1 OS=Mus musculus GN=Atp1b1 PE=1 SV=1
A0A0G2JGD2	8.4487E-04	0	0	0	0	5	5	3	4	Protein S100-A4 (Fragment) OS=Mus musculus GN=S100a4 PE=1 SV=1
P07091	8.4487E-04	0	0	0	0	5	5	3	4	Protein S100-A4 OS=Mus musculus GN=S100a4 PE=1 SV=1
P46935	8.8205E-04	7	4	4	2	36	23	23	35	E3 ubiquitin-protein ligase NEDD4 OS=Mus musculus GN=Nedd4 PE=1 SV=3
O70318	9.0375E-04	7	6	3	2	41	37	27	49	Band 4.1-like protein 2 OS=Mus musculus GN=Epb41l2 PE=1 SV=2
P37040	9.2125E-04	24	19	28	7	0	0	0	0	NADPH--cytochrome P450 reductase OS=Mus musculus GN=Por PE=1 SV=2
P37889	9.4862E-04	0	0	0	0	86	61	36	62	Fibulin-2 OS=Mus musculus GN=Fbln2 PE=1 SV=2
P30285	9.5908E-04	12	13	15	4	4	7	4	4	Cyclin-dependent kinase 4 OS=Mus musculus GN=Cdk4 PE=1 SV=1
P11983	9.9369E-04	158	128	147	48	99	79	55	81	T-complex protein 1 subunit alpha OS=Mus musculus GN=Tcp1 PE=1 SV=3
P26516	1.0206E-03	25	24	23	10	13	12	9	20	26S proteasome non-ATPase regulatory subunit 7 OS=Mus musculus GN=Psmd7 PE=1 SV=2
Q01320	1.0239E-03	63	49	49	22	0	0	0	0	DNA topoisomerase 2-alpha OS=Mus musculus GN=Top2a PE=1 SV=2
P60766	1.0399E-03	26	22	22	7	82	63	48	57	Cell division control protein 42 homolog OS=Mus musculus GN=Cdc42 PE=1 SV=2
Q99P88	1.0467E-03	8	7	7	3	0	0	0	0	Nuclear pore complex protein Nup155 OS=Mus musculus GN=Nup155 PE=1 SV=1
Q922D8	1.0701E-03	87	70	76	24	62	54	40	48	C-1-tetrahydrofolate synthase, cytoplasmic OS=Mus musculus GN=Mthfd1 PE=1 SV=4
A0A0J9YUE9	1.1716E-03	0	0	0	0	10	6	6	10	Dynamin-1 OS=Mus musculus GN=Dnm1 PE=1 SV=1
P54728	1.1768E-03	0	0	0	0	8	7	7	8	UV excision repair protein RAD23 homolog B OS=Mus musculus GN=Rad23b PE=1 SV=2
Q9R0E1	1.1799E-03	19	17	10	4	11	11	3	4	Procollagen-lysine,2-oxoglutarate 5-dioxygenase 3 OS=Mus musculus GN=Plod3 PE=1 SV=1
P47962	1.1958E-03	140	124	127	57	45	23	30	79	60S ribosomal protein L5 OS=Mus musculus GN=Rpl5 PE=1 SV=3
Q9Z2V5	1.2249E-03	5	5	6	2	0	0	0	0	Histone deacetylase 6 OS=Mus musculus GN=Hdac6 PE=1 SV=3
A0A1B0GX25	1.2249E-03	5	5	6	2	0	0	0	0	Histone deacetylase 6 (Fragment) OS=Mus musculus GN=Hdac6 PE=1 SV=1
Q8K2Z4	1.2315E-03	6	8	5	2	0	2	0	0	Condensin complex subunit 1 OS=Mus musculus GN=Ncapd2 PE=1 SV=2
A0A0R4J0H7	1.2315E-03	6	8	5	2	0	2	0	0	Condensin complex subunit 1 OS=Mus musculus GN=Ncapd2 PE=1 SV=1
Q80X90	1.2448E-03	51	26	45	7	163	151	101	135	Filamin-B OS=Mus musculus GN=Flnb PE=1 SV=3
Q810B6	1.2681E-03	5	5	5	2	0	0	0	0	Rabankyrin-5 OS=Mus musculus GN=Ankfy1 PE=1 SV=2
P42932	1.2940E-03	192	152	151	45	114	107	74	102	T-complex protein 1 subunit theta OS=Mus musculus GN=Cct8 PE=1 SV=3
G3XA35	1.3870E-03	0	0	0	0	12	9	6	7	MCG116562, isoform CRA_a OS=Mus musculus GN=Vcan PE=1 SV=1
E9PYH0	1.3870E-03	0	0	0	0	12	9	6	7	Versican core protein OS=Mus musculus GN=Vcan PE=1 SV=1
Q61739	1.3996E-03	36	26	26	9	6	6	7	5	Integrin alpha-6 OS=Mus musculus GN=Itga6 PE=1 SV=3
P10518	1.4062E-03	0	0	0	0	8	9	7	8	Delta-aminolevulinic acid dehydratase OS=Mus musculus GN=Alad PE=1 SV=1
P10107	1.4120E-03	90	73	70	26	229	152	129	205	Annexin A1 OS=Mus musculus GN=Anxa1 PE=1 SV=2
B7ZCU0	1.4258E-03	0	2	4	0	4	7	6	4	Abl interactor 1 OS=Mus musculus GN=Abi1 PE=1 SV=1
B7ZCU2	1.4258E-03	0	2	4	0	4	7	6	4	Abl interactor 1 OS=Mus musculus GN=Abi1 PE=1 SV=1
B7ZCU3	1.4258E-03	0	2	4	0	4	7	6	4	Abl interactor 1 OS=Mus musculus GN=Abi1 PE=1 SV=1
B7ZCU4	1.4258E-03	0	2	4	0	4	7	6	4	Abl interactor 1 OS=Mus musculus GN=Abi1 PE=1 SV=1
B7ZCU5	1.4258E-03	0	2	4	0	4	7	6	4	Abl interactor 1 OS=Mus musculus GN=Abi1 PE=1 SV=1
Q8CBW3	1.4258E-03	0	2	4	0	4	7	6	4	Abl interactor 1 OS=Mus musculus GN=Abi1 PE=1 SV=3
J3QNK8	1.4258E-03	0	2	4	0	4	7	6	4	Abl interactor 1 OS=Mus musculus GN=Abi1 PE=1 SV=1
Q9CSH3	1.4274E-03	40	33	49	12	0	0	0	2	Exosome complex exonuclease RRP44 OS=Mus musculus GN=Dis3 PE=1 SV=4
Q6P069	1.4303E-03	0	0	0	0	12	11	8	8	Sorcin OS=Mus musculus GN=Sri PE=1 SV=1
Q9Z1T1	1.4330E-03	9	7	11	3	0	0	0	0	AP-3 complex subunit beta-1 OS=Mus musculus GN=Ap3b1 PE=1 SV=2
P18872	1.4805E-03	0	0	0	0	26	22	13	29	Guanine nucleotide-binding protein G(o) subunit alpha OS=Mus musculus GN=Gnao1 PE=1 SV=3
Q9CZU3	1.4958E-03	16	16	21	7	0	3	3	2	Superkiller viralicidic activity 2-like 2 OS=Mus musculus GN=Skiv2l2 PE=1 SV=1
F6QTS1	1.4984E-03	15	6	10	3	3	0	0	0	COP9 signalosome complex subunit 4 (Fragment) OS=Mus musculus GN=Cops4 PE=1 SV=1
Q02248	1.5575E-03	12	11	13	4	50	35	25	38	Catenin beta-1 OS=Mus musculus GN=Ctnnb1 PE=1 SV=1
P19001	1.5702E-03	16	10	17	4	0	0	0	0	Keratin, type I cytoskeletal 19 OS=Mus musculus GN=Krt19 PE=1 SV=1
P80314	1.5821E-03	217	145	230	79	76	70	68	120	T-complex protein 1 subunit beta OS=Mus musculus GN=Cct2 PE=1 SV=4
Q8CG14	1.6038E-03	0	0	0	0	12	12	6	10	Complement C1s-A subcomponent OS=Mus musculus GN=C1sa PE=2 SV=2
E9Q6C2	1.6038E-03	0	0	0	0	12	12	6	10	Protein C1s1 OS=Mus musculus GN=C1s1 PE=1 SV=1
D3Z2M7	1.6145E-03	0	0	0	0	26	19	11	25	Guanine nucleotide-binding protein G(o) subunit alpha (Fragment) OS=Mus musculus GN=Gnao1 PE=1 SV=1
O88322	1.7128E-03	0	0	0	0	92	66	35	71	Nidogen-2 OS=Mus musculus GN=Nid2 PE=1 SV=2
P14685	1.7174E-03	71	58	71	18	25	32	34	30	26S proteasome non-ATPase regulatory subunit 3 OS=Mus musculus GN=Psmd3 PE=1 SV=3
Q3THK7	1.7554E-03	40	33	33	15	17	11	8	18	GMP synthase [glutamine-hydrolyzing] OS=Mus musculus GN=Gmps PE=1 SV=2
O70326	1.7876E-03	0	0	0	0	5	3	3	3	Gremlin-1 OS=Mus musculus GN=Grem1 PE=2 SV=1
F8VPK5	1.9562E-03	11	9	8	4	0	0	0	0	Rho-associated protein kinase OS=Mus musculus GN=Rock2 PE=1 SV=1
O08759	2.0716E-03	8	6	5	2	3	0	0	0	Ubiquitin-protein ligase E3A OS=Mus musculus GN=Ube3a PE=1 SV=2
Q62523	2.0850E-03	0	0	0	0	6	5	3	7	Zyxin OS=Mus musculus GN=Zyx PE=1 SV=2
Q7TQE2	2.0850E-03	0	0	0	0	6	5	3	7	Zyx protein OS=Mus musculus GN=Zyx PE=1 SV=1
F7D5L2	2.0943E-03	13	10	12	5	4	4	4	6	Methylosome protein 50 (Fragment) OS=Mus musculus GN=Wdr77 PE=1 SV=1
Q9Z1F9	2.0945E-03	47	35	43	13	19	18	11	8	SUMO-activating enzyme subunit 2 OS=Mus musculus GN=Uba2 PE=1 SV=1
Q91ZX7	2.0947E-03	0	3	0	2	50	35	23	34	Prolow-density lipoprotein receptor-related protein 1 OS=Mus musculus GN=Lrp1 PE=1 SV=1
A0A0R4J0I9	2.0947E-03	0	3	0	2	50	35	23	34	Low density lipoprotein receptor-related protein 1 OS=Mus musculus GN=Lrp1 PE=1 SV=1
F8VQJ3	2.1025E-03	36	33	36	9	112	105	61	84	Laminin subunit gamma-1 OS=Mus musculus GN=Lamc1 PE=1 SV=1
Q3UH59	2.1095E-03	133	96	111	31	128	92	74	96	Myosin-10 OS=Mus musculus GN=Myh10 PE=1 SV=1
Q61001	2.1096E-03	56	33	39	18	0	0	0	0	Laminin subunit alpha-5 OS=Mus musculus GN=Lama5 PE=1 SV=4
Q01149	2.1203E-03	0	0	0	0	42	39	25	26	Collagen alpha-2(I) chain OS=Mus musculus GN=Col1a2 PE=1 SV=2
Q8C1A5	2.1280E-03	67	37	57	13	5	3	0	2	Thimet oligopeptidase OS=Mus musculus GN=Thop1 PE=1 SV=1
A0A0R4IZY0	2.1280E-03	67	37	57	13	5	3	0	2	Thimet oligopeptidase OS=Mus musculus GN=Thop1 PE=1 SV=1
Q9JLJ2	2.1553E-03	32	22	21	7	10	7	5	11	4-trimethylaminobutyraldehyde dehydrogenase OS=Mus musculus GN=Aldh9a1 PE=1 SV=1
Q3U367	2.1553E-03	32	22	21	7	10	7	5	11	4-trimethylaminobutyraldehyde dehydrogenase OS=Mus musculus GN=Aldh9a1 PE=1 SV=1
H3BL26	2.2492E-03	0	0	0	0	6	5	4	8	Tetraspanin-6 (Fragment) OS=Mus musculus GN=Tspan6 PE=1 SV=8
O70401	2.2492E-03	0	0	0	0	6	5	4	8	Tetraspanin-6 OS=Mus musculus GN=Tspan6 PE=1 SV=1
Q99L96	2.2492E-03	0	0	0	0	6	5	4	8	Tetraspanin OS=Mus musculus GN=Tspan6 PE=1 SV=1
H3BLJ9	2.2983E-03	52	44	41	7	21	22	18	0	S-formylglutathione hydrolase OS=Mus musculus GN=Esd PE=1 SV=1
P10639	2.3325E-03	0	8	11	6	16	25	18	42	Thioredoxin OS=Mus musculus GN=Txn PE=1 SV=3
P49717	2.3613E-03	50	46	44	15	24	18	17	20	DNA replication licensing factor MCM4 OS=Mus musculus GN=Mcm4 PE=1 SV=1
P28654	2.3675E-03	0	0	0	0	77	101	72	91	Decorin OS=Mus musculus GN=Dcn PE=1 SV=1
Q62000	2.4045E-03	0	0	0	0	14	9	5	10	Mimecan OS=Mus musculus GN=Ogn PE=1 SV=1
Q9D0T1	2.4385E-03	15	9	13	3	10	7	6	6	NHP2-like protein 1 OS=Mus musculus GN=Snu13 PE=1 SV=4
A0A0R4J049	2.4634E-03	18	15	13	5	7	8	4	11	Protein arginine N-methyltransferase 5 OS=Mus musculus GN=Prmt5 PE=1 SV=1
Q922B2	2.5500E-03	112	68	91	31	29	31	18	23	Aspartate--tRNA ligase, cytoplasmic OS=Mus musculus GN=Dars PE=1 SV=2
Q8BTS0	2.5976E-03	52	37	45	11	13	9	0	8	DEAD (Asp-Glu-Ala-Asp) box polypeptide 5 OS=Mus musculus GN=Ddx5 PE=1 SV=1

P43277	2.6189E-03	36	24	33	13	2	2	0	0	Histone H1.3 OS=Mus musculus GN=Hist1h1d PE=1 SV=2
P15864	2.6189E-03	36	24	33	13	2	2	0	0	Histone H1.2 OS=Mus musculus GN=Hist1h1c PE=1 SV=2
D3Z2H2	2.6231E-03	0	5	4	2	27	18	17	26	Catenin delta-1 OS=Mus musculus GN=Ctnnd1 PE=1 SV=1
E9Q8Z8	2.6231E-03	0	5	4	2	27	18	17	26	Catenin delta-1 OS=Mus musculus GN=Ctnnd1 PE=1 SV=1
Q7TPR4	2.7395E-03	28	26	25	10	94	79	42	84	Alpha-actinin-1 OS=Mus musculus GN=Actn1 PE=1 SV=1
Q61553	2.7538E-03	0	0	0	0	85	107	89	111	Fascin OS=Mus musculus GN=Fscn1 PE=1 SV=4
A2A7S7	2.7550E-03	3	0	4	0	11	9	10	12	Tyrosine--tRNA ligase OS=Mus musculus GN=Yars PE=1 SV=1
Q91WQ3	2.7550E-03	3	0	4	0	11	9	10	12	Tyrosine--tRNA ligase, cytoplasmic OS=Mus musculus GN=Yars PE=1 SV=3
Q9EPL8	2.7980E-03	18	16	18	8	0	5	0	4	Importin-7 OS=Mus musculus GN=Ipo7 PE=1 SV=2
G3UXG7	2.8867E-03	0	0	0	0	5	5	5	5	Casein kinase II subunit beta OS=Mus musculus GN=Csnk2b PE=1 SV=1
P67871	2.8867E-03	0	0	0	0	5	5	5	5	Casein kinase II subunit beta OS=Mus musculus GN=Csnk2b PE=1 SV=1
Q3TXS7	2.9106E-03	42	38	31	11	23	19	13	18	26S proteasome non-ATPase regulatory subunit 1 OS=Mus musculus GN=Psmd1 PE=1 SV=1
P02468	2.9437E-03	35	32	33	9	103	96	53	77	Laminin subunit gamma-1 OS=Mus musculus GN=Lamc1 PE=1 SV=2
O35566	2.9482E-03	44	44	61	13	0	0	0	0	CD151 antigen OS=Mus musculus GN=Cd151 PE=1 SV=2
P62317	3.0055E-03	10	8	8	3	0	0	2	3	Small nuclear ribonucleoprotein Sm D2 OS=Mus musculus GN=Snrpd2 PE=1 SV=1
H3BJP2	3.0555E-03	52	44	40	7	21	22	18	0	S-formylglutathione hydrolase (Fragment) OS=Mus musculus GN=Esd PE=1 SV=1
Q8VDM4	3.0742E-03	89	59	70	25	38	39	30	49	26S proteasome non-ATPase regulatory subunit 2 OS=Mus musculus GN=Psmd2 PE=1 SV=1
A0A1B0GRG3	3.1216E-03	41	42	59	13	0	0	0	0	CD151 antigen (Fragment) OS=Mus musculus GN=Cd151 PE=1 SV=1
F6YY69	3.1822E-03	89	81	76	40	23	26	29	67	14-3-3 protein theta (Fragment) OS=Mus musculus GN=Ywhaq PE=1 SV=1
F8WIT2	3.2005E-03	132	80	104	32	219	180	123	210	Annexin OS=Mus musculus GN=Anxa6 PE=1 SV=1
P27601	3.2321E-03	2	2	0	0	8	10	5	9	Guanine nucleotide-binding protein subunit alpha-13 OS=Mus musculus GN=Gna13 PE=1 SV=1
E9Q6R7	3.2437E-03	0	0	2	0	6	3	5	6	Protein Utrn OS=Mus musculus GN=Utrn PE=1 SV=1
D3Z4A4	3.2694E-03	0	0	0	0	27	18	9	20	Peroxiredoxin-2 (Fragment) OS=Mus musculus GN=Prdx2 PE=1 SV=8
Q8R480	3.3172E-03	7	4	8	2	0	0	0	0	Nuclear pore complex protein Nup85 OS=Mus musculus GN=Nup85 PE=1 SV=1
D3YX76	3.3397E-03	0	0	0	0	4	5	3	6	Glutathione S-transferase OS=Mus musculus GN=Gstm2 PE=1 SV=1
P15626	3.3397E-03	0	0	0	0	4	5	3	6	Glutathione S-transferase Mu 2 OS=Mus musculus GN=Gstm2 PE=1 SV=2
Q5SXR6	3.3435E-03	414	294	302	96	189	174	129	202	Clathrin heavy chain OS=Mus musculus GN=Cltc PE=1 SV=1
O88477	3.3787E-03	5	2	3	0	15	10	7	5	Insulin-like growth factor 2 mRNA-binding protein 1 OS=Mus musculus GN=Igf2bp1 PE=1 SV=1
Q5SQB5	3.4491E-03	58	34	35	13	27	17	15	19	Nucleophosmin OS=Mus musculus GN=Npm1 PE=1 SV=1
P16125	3.4578E-03	0	0	0	0	11	13	12	13	L-lactate dehydrogenase B chain OS=Mus musculus GN=Ldhb PE=1 SV=2
A0A087WS16	3.5044E-03	0	0	0	0	25	18	9	15	Protein Col6a3 OS=Mus musculus GN=Col6a3 PE=1 SV=1
Q9DCC4	3.5735E-03	0	0	0	0	5	3	4	4	Pyrroline-5-carboxylate reductase 3 OS=Mus musculus GN=Pycrl PE=1 SV=2
Q5SS83	3.6049E-03	0	0	0	0	8	4	5	5	Flotillin 2, isoform CRA_a OS=Mus musculus GN=Flot2 PE=1 SV=1
Q60634	3.6049E-03	0	0	0	0	8	4	5	5	Flotillin-2 OS=Mus musculus GN=Flot2 PE=1 SV=2
Q8CIG8	3.7007E-03	16	15	12	5	8	11	4	13	Protein arginine N-methyltransferase 5 OS=Mus musculus GN=Prmt5 PE=1 SV=3
P18760	3.7241E-03	67	60	60	17	134	96	86	108	Cofilin-1 OS=Mus musculus GN=Cfl1 PE=1 SV=3
F6VW30	3.7286E-03	89	82	76	41	22	26	29	67	14-3-3 protein theta (Fragment) OS=Mus musculus GN=Ywhaq PE=1 SV=1
P30999	3.7519E-03	0	5	4	2	27	17	17	27	Catenin delta-1 OS=Mus musculus GN=Ctnnd1 PE=1 SV=2
E9Q905	3.7519E-03	0	5	4	2	27	17	17	27	Catenin delta-1 OS=Mus musculus GN=Ctnnd1 PE=1 SV=1
A0A067XG53	3.7689E-03	0	0	0	0	8	8	6	5	Peripheral plasma membrane protein CASK (Fragment) OS=Mus musculus GN=Cask PE=1 SV=1
O70589	3.7689E-03	0	0	0	0	8	8	6	5	Peripheral plasma membrane protein CASK OS=Mus musculus GN=Cask PE=1 SV=2
A2AW05	3.7811E-03	6	3	5	2	0	0	0	0	FACT complex subunit SSRP1 (Fragment) OS=Mus musculus GN=Ssrp1 PE=1 SV=1
Q08943	3.7811E-03	6	3	5	2	0	0	0	0	FACT complex subunit SSRP1 OS=Mus musculus GN=Ssrp1 PE=1 SV=2
E9Q904	3.8046E-03	0	5	4	2	27	17	17	26	Catenin delta-1 OS=Mus musculus GN=Ctnnd1 PE=1 SV=1
F8WGL3	3.8699E-03	67	60	61	17	132	95	86	108	Cofilin-1 OS=Mus musculus GN=Cfl1 PE=1 SV=1
Q68FL6	3.9447E-03	56	33	46	11	11	10	5	12	Methionine--tRNA ligase, cytoplasmic OS=Mus musculus GN=Mars PE=1 SV=1
E9QB02	3.9447E-03	56	33	46	11	11	10	5	12	Methionine--tRNA ligase, cytoplasmic OS=Mus musculus GN=Mars PE=1 SV=1
P46061	3.9649E-03	24	20	13	6	2	2	0	7	Ran GTPase-activating protein 1 OS=Mus musculus GN=Rangap1 PE=1 SV=2
E9QKR0	3.9684E-03	120	97	86	37	42	38	36	55	Guanine nucleotide-binding protein G(I)/G(S)/G(T) subunit beta-2 OS=Mus musculus GN=Gnb2 PE=1 SV=1
P11152	4.0176E-03	0	0	0	0	19	8	10	13	Lipoprotein lipase OS=Mus musculus GN=Lpl PE=1 SV=3
P62880	4.0316E-03	121	97	86	37	42	38	36	55	Guanine nucleotide-binding protein G(I)/G(S)/G(T) subunit beta-2 OS=Mus musculus GN=Gnb2 PE=1 SV=3
P58854	4.0334E-03	4	5	6	2	0	0	0	0	Gamma-tubulin complex component 3 OS=Mus musculus GN=Tubgcp3 PE=1 SV=2
A0A0N4SVS6	4.0425E-03	4	4	0	0	26	19	14	11	Cellular nucleic acid-binding protein OS=Mus musculus GN=Cnbp PE=1 SV=1
P68037	4.1297E-03	2	3	0	0	8	8	5	4	Ubiquitin-conjugating enzyme E2 L3 OS=Mus musculus GN=Ube2l3 PE=1 SV=1
Q6P5D8	4.1758E-03	17	9	17	6	0	0	0	0	Structural maintenance of chromosomes flexible hinge domain-containing protein 1 OS=Mus musculus GN=Smchd1 PE=1 SV=2
Q68FD5	4.1908E-03	416	294	302	95	189	175	130	204	Clathrin heavy chain 1 OS=Mus musculus GN=Cltc PE=1 SV=3
A2AN08	4.2293E-03	26	24	34	6	12	17	14	11	E3 ubiquitin-protein ligase UBR4 OS=Mus musculus GN=Ubr4 PE=1 SV=1
A1BN54	4.2464E-03	28	26	24	10	89	72	37	78	Alpha actinin 1a OS=Mus musculus GN=Actn1 PE=1 SV=1
Q61879	4.2633E-03	132	97	111	31	128	93	76	97	Myosin-10 OS=Mus musculus GN=Myh10 PE=1 SV=2
B1B1E2	4.2921E-03	0	0	0	0	4	6	4	5	Latent-transforming growth factor beta-binding protein 1 (Fragment) OS=Mus musculus GN=Ltbp1 PE=1 SV=8
Q8CG19	4.2921E-03	0	0	0	0	4	6	4	5	Latent-transforming growth factor beta-binding protein 1 OS=Mus musculus GN=Ltbp1 PE=1 SV=2
Q61937	4.2950E-03	59	34	35	13	27	18	15	19	Nucleophosmin OS=Mus musculus GN=Npm1 PE=1 SV=1
P58252	4.3118E-03	399	264	287	89	315	191	161	247	Elongation factor 2 OS=Mus musculus GN=Eef2 PE=1 SV=2
Q9ET54	4.3760E-03	0	0	0	0	6	8	7	8	Palladin OS=Mus musculus GN=Palld PE=1 SV=2
E9QN70	4.3834E-03	10	9	10	4	45	26	22	33	Laminin subunit beta-1 OS=Mus musculus GN=Lamb1 PE=1 SV=1
P02469	4.3834E-03	10	9	10	4	45	26	22	33	Laminin subunit beta-1 OS=Mus musculus GN=Lamb1 PE=1 SV=3
D3YVC1	4.4314E-03	123	103	109	46	24	30	47	86	40S ribosomal protein S2 (Fragment) OS=Mus musculus GN=Rps2 PE=1 SV=1
Q9JHK4	4.4418E-03	10	8	7	4	0	0	0	0	Geranylgeranyl transferase type-2 subunit alpha OS=Mus musculus GN=Rabggta PE=1 SV=1
B7FAV1	4.4552E-03	99	73	99	20	303	195	138	211	Filamin, alpha (Fragment) OS=Mus musculus GN=Flna PE=1 SV=1
B7ZNU9	4.4711E-03	4	0	0	0	10	6	5	4	Beta-2-syntrophin OS=Mus musculus GN=Sntb2 PE=2 SV=1
P48428	4.5249E-03	0	0	0	0	11	5	5	6	Tubulin-specific chaperone A OS=Mus musculus GN=Tbca PE=1 SV=3
B7FAU9	4.5721E-03	99	73	99	20	304	195	138	212	Filamin, alpha OS=Mus musculus GN=Flna PE=1 SV=1
Q8BTM8	4.5721E-03	99	73	99	20	304	195	138	212	Filamin-A OS=Mus musculus GN=Flna PE=1 SV=5
Q8CGC7	4.5905E-03	164	134	131	39	69	68	51	85	Bifunctional glutamate/proline--tRNA ligase OS=Mus musculus GN=Eprs PE=1 SV=4
E9Q2A0	4.6097E-03	0	0	0	0	8	7	5	4	EGF-containing fibulin-like extracellular matrix protein 2 (Fragment) OS=Mus musculus GN=Efemp2 PE=1 SV=1
P12815	4.6137E-03	20	7	12	6	18	6	8	18	Programmed cell death protein 6 OS=Mus musculus GN=Pdcd6 PE=1 SV=2
P20152	4.7288E-03	46	37	39	14	157	96	66	127	Vimentin OS=Mus musculus GN=Vim PE=1 SV=3
Q9D2R0	4.7819E-03	8	2	5	0	16	12	12	13	Acetoacetyl-CoA synthetase OS=Mus musculus GN=Aacs PE=1 SV=1
E9Q634	4.8632E-03	6	5	8	2	0	0	0	2	Unconventional myosin-Ie OS=Mus musculus GN=Myo1e PE=1 SV=1
Q5SQB0	4.8659E-03	59	33	35	12	27	18	15	19	Nucleophosmin OS=Mus musculus GN=Npm1 PE=1 SV=1
A0A0R4J233	5.0844E-03	8	8	13	3	0	0	0	0	Septin-10 OS=Mus musculus GN=Sept10 PE=1 SV=1
Q8C650	5.0844E-03	8	8	13	3	0	0	0	0	Septin-10 OS=Mus musculus GN=Sept10 PE=1 SV=1
Q99K41	5.1092E-03	0	0	0	0	26	32	15	21	EMILIN-1 OS=Mus musculus GN=Emilin1 PE=1 SV=1
E9PXM6	5.1282E-03	0	6	8	0	8	10	10	7	Equilibrative nucleoside transporter 1 OS=Mus musculus GN=Slc29a1 PE=1 SV=1
E9PWQ3	5.1488E-03	0	0	0	0	29	22	11	15	Protein Col6a3 OS=Mus musculus GN=Col6a3 PE=1 SV=2
P62082	5.1578E-03	17	14	10	4	6	4	3	5	40S ribosomal protein S7 OS=Mus musculus GN=Rps7 PE=2 SV=1
P11214	5.1782E-03	14	9	10	6	0	0	0	2	Tissue-type plasminogen activator OS=Mus musculus GN=Plat PE=1 SV=3
P68254	5.1927E-03	89	81	76	43	23	26	29	66	14-3-3 protein theta OS=Mus musculus GN=Ywhaq PE=1 SV=1
P26041	5.1949E-03	111	85	83	28	266	206	188	192	Moesin OS=Mus musculus GN=Msn PE=1 SV=3
Q9DBJ1	5.2862E-03	50	40	30	14	88	78	49	105	Phosphoglycerate mutase 1 OS=Mus musculus GN=Pgam1 PE=1 SV=3
A0A140T8L5	5.2992E-03	126	104	101	46	33	25	39	94	Protein Rps2-ps6 OS=Mus musculus GN=Rps2-ps6 PE=3 SV=1
D3Z7H6	5.3130E-03	0	5	4	2	27	16	17	27	Catenin delta-1 OS=Mus musculus GN=Ctnnd1 PE=1 SV=1
E9Q8Z4	5.3130E-03	0	5	4	2	27	16	17	27	Catenin delta-1 OS=Mus musculus GN=Ctnnd1 PE=1 SV=1
O70433	5.3616E-03	0	0	0	0	5	7	5	9	Four and a half LIM domains protein 2 OS=Mus musculus GN=Fhl2 PE=1 SV=1
Q9WUU7	5.3883E-03	11	6	8	4	0	0	0	0	Cathepsin Z OS=Mus musculus GN=Ctsz PE=1 SV=1
Q9DBC7	5.4406E-03	4	3	5	0	16	22	17	20	cAMP-dependent protein kinase type I-alpha regulatory subunit OS=Mus musculus GN=Prkar1a PE=1 SV=3
E9PYB0	5.6377E-03	0	0	0	0	7	4	6	6	Protein Ahnak2 (Fragment) OS=Mus musculus GN=Ahnak2 PE=1 SV=8
Q61753	5.6400E-03	87	66	69	18	81	57	42	43	D-3-phosphoglycerate dehydrogenase OS=Mus musculus GN=Phgdh PE=1 SV=3
S4R1I6	5.6499E-03	44	33	42	9	12	8	0	7	MCG2872, isoform CRA_b OS=Mus musculus GN=Ddx5 PE=1 SV=1
B1B0C7	5.7747E-03	470	369	367	154	1090	802	577	780	Basement membrane-specific heparan sulfate proteoglycan core protein OS=Mus musculus GN=Hspg2 PE=1 SV=1
Q9WVJ9	5.8766E-03	0	0	0	0	12	10	5	6	EGF-containing fibulin-like extracellular matrix protein 2 OS=Mus musculus GN=Efemp2 PE=1 SV=1
G5E8D6	5.8766E-03	0	0	0	0	12	10	5	6	EGF-containing fibulin-like extracellular matrix protein 2 OS=Mus musculus GN=Efemp2 PE=1 SV=1
Q9WTZ2	5.8946E-03	5	3	4	2	0	0	0	0	Membrane-bound transcription factor site-1 protease OS=Mus musculus GN=Mbtps1 PE=1 SV=1
Q6IRU2	5.9680E-03	17	18	18	9	51	44	38	48	Tropomyosin alpha-4 chain OS=Mus musculus GN=Tpm4 PE=1 SV=3
P10493	5.9969E-03	0	0	0	0	126	87	36	89	Nidogen-1 OS=Mus musculus GN=Nid1 PE=1 SV=2
Q62470	6.0487E-03	90	51	69	19	20	24	22	26	Integrin alpha-3 OS=Mus musculus GN=Itga3 PE=1 SV=1
Q9JJU8	6.0895E-03	0	0	0	0	8	8	9	8	SH3 domain-binding glutamic acid-rich-like protein OS=Mus musculus GN=Sh3bgrl PE=1 SV=1
E9PZ16	6.1781E-03	480	373	373	159	1100	808	579	792	Basement membrane-specific heparan sulfate proteoglycan core protein OS=Mus musculus GN=Hspg2 PE=1 SV=1
Q9CQV8	6.2301E-03	73	54	58	34	29	21	24	58	14-3-3 protein beta/alpha OS=Mus musculus GN=Ywhab PE=1 SV=3
Q4FE56	6.2442E-03	47	46	41	13	16	13	11	24	Probable ubiquitin carboxyl-terminal hydrolase FAF-X OS=Mus musculus GN=Usp9x PE=1 SV=1
P70398	6.2442E-03	47	46	41	13	16	13	11	24	Probable ubiquitin carboxyl-terminal hydrolase FAF-X OS=Mus musculus GN=Usp9x PE=1 SV=2
Q62059	6.3155E-03	0	0	0	0	11	9	5	5	Versican core protein OS=Mus musculus GN=Vcan PE=1 SV=2
Q61081	6.3335E-03	0	0	0	0	5	2	2	3	Hsp90 co-chaperone Cdc37 OS=Mus musculus GN=Cdc37 PE=1 SV=1
Q9D1A2	6.5443E-03	18	10	15	4	0	3	0	3	Cytosolic non-specific dipeptidase OS=Mus musculus GN=Cndp2 PE=1 SV=1
P40142	6.5846E-03	63	52	60	16	38	39	17	23	Transketolase OS=Mus musculus GN=Tkt PE=1 SV=1
D3YZX3	6.5870E-03	113	94	82	37	38	34	36	48	Guanine nucleotide-binding protein G(I)/G(S)/G(T) subunit beta-2 OS=Mus musculus GN=Gnb2 PE=1 SV=1
O08603	6.6067E-03	0	0	0	0	8	7	6	4	Retinoic acid early-inducible protein 1-beta OS=Mus musculus GN=Raet1b PE=1 SV=1
E9Q3L1	6.6794E-03	6	9	8	2	0	0	0	0	Unconventional myosin-VI OS=Mus musculus GN=Myo6 PE=1 SV=1
V9GXM4	6.6794E-03	6	9	8	2	0	0	0	0	Unconventional myosin-VI OS=Mus musculus GN=Myo6 PE=1 SV=1
V9GX76	6.6794E-03	6	9	8	2	0	0	0	0	Unconventional myosin-VI OS=Mus musculus GN=Myo6 PE=1 SV=1
E9PVU0	6.6794E-03	6	9	8	2	0	0	0	0	Unconventional myosin-VI OS=Mus musculus GN=Myo6 PE=1 SV=1
E9Q175	6.6794E-03	6	9	8	2	0	0	0	0	Unconventional myosin-VI OS=Mus musculus GN=Myo6 PE=1 SV=1
E9Q174	6.6794E-03	6	9	8	2	0	0	0	0	Unconventional myosin-VI OS=Mus musculus GN=Myo6 PE=1 SV=1
P53996	6.8278E-03	0	0	0	0	22	13	12	9	Cellular nucleic acid-binding protein OS=Mus musculus GN=Cnbp PE=1 SV=2
P08113	6.8871E-03	29	23	26	9	25	20	14	27	Endoplasmin OS=Mus musculus GN=Hsp90b1 PE=1 SV=2
Q5SW19	6.9606E-03	19	18	20	7	11	7	9	10	Clustered mitochondria protein homolog OS=Mus musculus GN=Cluh PE=1 SV=2
A0A0R4J140	6.9606E-03	19	18	20	7	11	7	9	10	Clustered mitochondria protein homolog OS=Mus musculus GN=Cluh PE=1 SV=1
Q60866	6.9640E-03	0	0	0	0	3	4	4	5	Phosphotriesterase-related protein OS=Mus musculus GN=Pter PE=1 SV=1
A2AUR3	6.9640E-03	0	0	0	0	3	4	4	5	Phosphotriesterase-related protein (Fragment) OS=Mus musculus GN=Pter PE=1 SV=2
P35441	6.9997E-03	0	0	0	0	4	7	4	6	Thrombospondin-1 OS=Mus musculus GN=Thbs1 PE=1 SV=1
Q80YQ1	6.9997E-03	0	0	0	0	4	7	4	6	Thrombospondin 1 OS=Mus musculus GN=Thbs1 PE=1 SV=1
E9PZX7	7.0650E-03	9	10	11	2	3	7	4	4	Cyclin-dependent kinase 4 (Fragment) OS=Mus musculus GN=Cdk4 PE=1 SV=1
P98063	7.0985E-03	54	55	52	28	0	0	0	0	Bone morphogenetic protein 1 OS=Mus musculus GN=Bmp1 PE=1 SV=2
Q5F2E8	7.1030E-03	2	0	0	0	8	7	7	6	Serine/threonine-protein kinase TAO1 OS=Mus musculus GN=Taok1 PE=1 SV=1
P06745	7.1050E-03	6	8	7	0	19	21	18	22	Glucose-6-phosphate isomerase OS=Mus musculus GN=Gpi PE=1 SV=4
P15532	7.1118E-03	23	26	22	4	68	54	56	52	Nucleoside diphosphate kinase A OS=Mus musculus GN=Nme1 PE=1 SV=1
D3YTP0	7.1305E-03	0	0	0	0	5	7	4	9	Metalloreductase STEAP3 (Fragment) OS=Mus musculus GN=Steap3 PE=1 SV=8
E9QN92	7.1305E-03	0	0	0	0	5	7	4	9	Metalloreductase STEAP3 OS=Mus musculus GN=Steap3 PE=1 SV=1
Q8CI59	7.1305E-03	0	0	0	0	5	7	4	9	Metalloreductase STEAP3 OS=Mus musculus GN=Steap3 PE=1 SV=1

A0A0R4J1G9	7.1305E-03	0	0	0	0	5	7	4	9	Metalloreductase STEAP3 OS=Mus musculus GN=Steap3 PE=1 SV=1
Q920Q8	7.2140E-03	7	4	3	2	0	0	0	0	Influenza virus NS1A-binding protein homolog OS=Mus musculus GN=Ivns1abp PE=1 SV=2
Q9CWF2	7.2181E-03	307	196	238	46	397	297	216	263	Tubulin beta-2B chain OS=Mus musculus GN=Tubb2b PE=1 SV=1
Q8BVQ9	7.2558E-03	61	55	49	21	17	21	21	20	26S protease regulatory subunit 7 OS=Mus musculus GN=Psmc2 PE=1 SV=1
Q9CWS0	7.2764E-03	0	0	0	0	5	3	4	3	N(G),N(G)-dimethylarginine dimethylaminohydrolase 1 OS=Mus musculus GN=Ddah1 PE=1 SV=3
P25444	7.2876E-03	132	107	110	46	37	30	47	101	40S ribosomal protein S2 OS=Mus musculus GN=Rps2 PE=1 SV=3
Q61191	7.2890E-03	19	9	12	4	2	3	0	4	Host cell factor 1 OS=Mus musculus GN=Hcfc1 PE=1 SV=2
F6YTZ4	7.2979E-03	125	104	103	46	26	25	43	95	Protein Gm6576 OS=Mus musculus GN=Gm6576 PE=3 SV=2
Q64314	7.3258E-03	0	0	0	0	8	10	5	13	Hematopoietic progenitor cell antigen CD34 OS=Mus musculus GN=Cd34 PE=1 SV=1
O88342	7.3340E-03	27	20	21	5	39	30	20	30	WD repeat-containing protein 1 OS=Mus musculus GN=Wdr1 PE=1 SV=3
P46471	7.4418E-03	61	56	49	21	18	21	21	20	26S protease regulatory subunit 7 OS=Mus musculus GN=Psmc2 PE=1 SV=5
P01027	7.5220E-03	0	0	0	0	12	17	11	10	Complement C3 OS=Mus musculus GN=C3 PE=1 SV=3
P46664	7.5288E-03	18	15	21	8	2	5	3	2	Adenylosuccinate synthetase isozyme 2 OS=Mus musculus GN=Adss PE=1 SV=2
A0A087WS56	7.5463E-03	48	34	45	13	518	384	169	326	Fibronectin OS=Mus musculus GN=Fn1 PE=1 SV=1
Q9CQW9	7.5996E-03	0	5	8	0	16	13	11	12	Interferon-induced transmembrane protein 3 OS=Mus musculus GN=Ifitm3 PE=1 SV=1
Q3U687	7.6210E-03	0	0	0	0	5	8	6	6	Protein Ifit1bl2 OS=Mus musculus GN=Ifit1bl2 PE=1 SV=1
F6RPJ9	7.6306E-03	19	14	9	4	6	0	0	5	Insulin-degrading enzyme (Fragment) OS=Mus musculus GN=Ide PE=1 SV=1
Q5NC80	7.7732E-03	22	26	22	4	65	54	56	51	Nucleoside diphosphate kinase (Fragment) OS=Mus musculus GN=Nme1 PE=1 SV=1
Q920B9	7.7924E-03	12	6	12	5	3	0	0	3	FACT complex subunit SPT16 OS=Mus musculus GN=Supt16h PE=1 SV=2
G3X956	7.7924E-03	12	6	12	5	3	0	0	3	FACT complex subunit SPT16 OS=Mus musculus GN=Supt16 PE=1 SV=1
D3Z536	7.8935E-03	120	98	98	46	23	22	43	92	Protein Gm8225 OS=Mus musculus GN=Gm8225 PE=3 SV=1
B1AUX2	7.9058E-03	19	9	13	4	2	3	0	4	Host cell factor 1 OS=Mus musculus GN=Hcfc1 PE=1 SV=1
P62874	7.9091E-03	108	89	75	36	45	32	32	44	Guanine nucleotide-binding protein G(I)/G(S)/G(T) subunit beta-1 OS=Mus musculus GN=Gnb1 PE=1 SV=3
P68372	7.9313E-03	311	205	249	46	416	310	228	281	Tubulin beta-4B chain OS=Mus musculus GN=Tubb4b PE=1 SV=1
Q8BMJ2	7.9445E-03	88	67	77	23	67	51	32	61	Leucine--tRNA ligase, cytoplasmic OS=Mus musculus GN=Lars PE=1 SV=2
Q99L27	8.1298E-03	19	10	12	6	0	4	0	4	GMP reductase 2 OS=Mus musculus GN=Gmpr2 PE=1 SV=2
P43346	8.1445E-03	16	12	6	4	0	0	0	0	Deoxycytidine kinase OS=Mus musculus GN=Dck PE=1 SV=1
E9QNN1	8.1965E-03	148	87	91	25	39	34	22	39	ATP-dependent RNA helicase A OS=Mus musculus GN=Dhx9 PE=1 SV=1
A0A087WPL5	8.1965E-03	148	87	91	25	39	34	22	39	ATP-dependent RNA helicase A OS=Mus musculus GN=Dhx9 PE=1 SV=1
Q3UHL6	8.2428E-03	49	35	47	14	533	385	169	334	Fibronectin OS=Mus musculus GN=Fn1 PE=1 SV=1
P52293	8.2573E-03	15	7	15	4	8	4	5	8	Importin subunit alpha-1 OS=Mus musculus GN=Kpna2 PE=1 SV=2
Q7TMM9	8.2655E-03	304	194	239	44	402	298	216	263	Tubulin beta-2A chain OS=Mus musculus GN=Tubb2a PE=1 SV=1
Q8K354	8.3182E-03	4	0	0	0	15	14	15	16	Carbonyl reductase [NADPH] 3 OS=Mus musculus GN=Cbr3 PE=1 SV=1
J3QNW0	8.5121E-03	57	46	40	12	8	13	11	19	DNA (cytosine-5)-methyltransferase OS=Mus musculus GN=Dnmt1 PE=1 SV=1
P13864	8.5121E-03	57	46	40	12	8	13	11	19	DNA (cytosine-5)-methyltransferase 1 OS=Mus musculus GN=Dnmt1 PE=1 SV=5
A0A087WR50	8.5145E-03	49	35	47	14	537	393	169	338	Fibronectin OS=Mus musculus GN=Fn1 PE=1 SV=1
Q920E5	8.5396E-03	0	0	2	0	8	5	6	3	Farnesyl pyrophosphate synthase OS=Mus musculus GN=Fdps PE=1 SV=1
D3Z2H7	8.5584E-03	0	5	4	2	25	14	14	25	Catenin delta-1 OS=Mus musculus GN=Ctnnd1 PE=1 SV=1
P11276	8.5928E-03	49	35	47	14	537	394	169	337	Fibronectin OS=Mus musculus GN=Fn1 PE=1 SV=4
E9Q2X6	8.5939E-03	12	16	20	8	0	3	0	3	Structural maintenance of chromosomes protein OS=Mus musculus GN=Smc4 PE=1 SV=1
Q8CG47	8.5939E-03	12	16	20	8	0	3	0	3	Structural maintenance of chromosomes protein 4 OS=Mus musculus GN=Smc4 PE=1 SV=1
contaminant_UBIQUITIN02	8.7200E-03	50	42	42	10	107	68	50	70	no description
P62908	8.8084E-03	121	77	102	25	81	60	55	69	40S ribosomal protein S3 OS=Mus musculus GN=Rps3 PE=1 SV=1
Z4YLI8	8.8100E-03	18	18	20	7	11	6	8	10	Clustered mitochondria protein homolog OS=Mus musculus GN=Cluh PE=1 SV=1
P14069	8.9384E-03	31	24	30	6	56	41	40	32	Protein S100-A6 OS=Mus musculus GN=S100a6 PE=1 SV=3
Q05793	9.0058E-03	400	325	327	143	983	731	531	695	Basement membrane-specific heparan sulfate proteoglycan core protein OS=Mus musculus GN=Hspg2 PE=1 SV=1
Q6P5B0	9.1060E-03	8	11	8	2	0	0	0	0	RRP12-like protein OS=Mus musculus GN=Rrp12 PE=1 SV=1
P70290	9.1634E-03	0	0	0	0	13	18	7	12	55 kDa erythrocyte membrane protein OS=Mus musculus GN=Mpp1 PE=1 SV=1
A2AN84	9.1634E-03	0	0	0	0	13	18	7	12	55 kDa erythrocyte membrane protein OS=Mus musculus GN=Mpp1 PE=1 SV=1
P97427	9.1695E-03	8	3	8	0	19	18	12	16	Dihydropyrimidinase-related protein 1 OS=Mus musculus GN=Crmp1 PE=1 SV=1
P11370	9.2154E-03	13	10	6	2	0	0	0	0	Retrovirus-related Env polyprotein from Fv-4 locus OS=Mus musculus GN=Fv4 PE=1 SV=2
Q03350	9.2283E-03	0	0	0	0	14	12	6	6	Thrombospondin-2 OS=Mus musculus GN=Thbs2 PE=1 SV=2
A0A087WSN6	9.2657E-03	47	35	47	14	505	370	157	318	Fibronectin OS=Mus musculus GN=Fn1 PE=1 SV=1
P62259	9.2766E-03	34	34	35	14	68	54	35	71	14-3-3 protein epsilon OS=Mus musculus GN=Ywhae PE=1 SV=1
P17918	9.4833E-03	54	39	37	16	42	29	21	33	Proliferating cell nuclear antigen OS=Mus musculus GN=Pcna PE=1 SV=2
Q7TMB8	9.5097E-03	41	27	26	17	14	15	8	26	Cytoplasmic FMR1-interacting protein 1 OS=Mus musculus GN=Cyfip1 PE=1 SV=1
Q62159	9.5305E-03	23	14	15	6	49	29	19	32	Rho-related GTP-binding protein RhoC OS=Mus musculus GN=Rhoc PE=1 SV=2
Q64449	9.5641E-03	0	0	0	0	7	9	5	13	C-type mannose receptor 2 OS=Mus musculus GN=Mrc2 PE=1 SV=3
Q9Z1N5	9.5894E-03	34	28	33	13	24	14	11	19	Spliceosome RNA helicase Ddx39b OS=Mus musculus GN=Ddx39b PE=1 SV=1
P14428	9.6101E-03	0	0	0	0	6	5	4	10	H-2 class I histocompatibility antigen, K-Q alpha chain (Fragment) OS=Mus musculus GN=H2-K1 PE=1 SV=1
Q6PHC1	9.6643E-03	147	127	136	34	108	84	64	90	Alpha-enolase OS=Mus musculus GN=Eno1 PE=1 SV=1
Q9D6X6	9.6692E-03	5	4	4	0	29	18	10	23	Serine protease 23 OS=Mus musculus GN=Prss23 PE=2 SV=2
Q63844	9.8165E-03	28	17	19	6	15	12	10	8	Mitogen-activated protein kinase 3 OS=Mus musculus GN=Mapk3 PE=1 SV=5
P14131	9.8561E-03	45	30	37	16	27	21	14	25	40S ribosomal protein S16 OS=Mus musculus GN=Rps16 PE=1 SV=4
Q8VC28	9.9026E-03	7	8	7	4	0	0	0	0	Aldo-keto reductase family 1 member C13 OS=Mus musculus GN=Akr1c13 PE=1 SV=2
G3X934	9.9491E-03	13	15	16	8	0	0	0	0	MCG115964 OS=Mus musculus GN=Wdr70 PE=1 SV=1
Q91ZW3	9.9990E-03	29	27	28	12	12	12	10	10	SWI/SNF-related matrix-associated actin-dependent regulator of chromatin subfamily A member 5 OS=Mus musculus GN=Smarca5 PE=1 SV=1
Q9JJ00	1.0001E-02	0	0	2	0	3	4	4	6	Phospholipid scramblase 1 OS=Mus musculus GN=Plscr1 PE=1 SV=1
Q8BKG3	1.0103E-02	6	2	0	2	23	13	16	19	Inactive tyrosine-protein kinase 7 OS=Mus musculus GN=Ptk7 PE=1 SV=1
Q501J6	1.0175E-02	39	25	32	12	25	18	12	17	Probable ATP-dependent RNA helicase DDX17 OS=Mus musculus GN=Ddx17 PE=1 SV=1
Z4YJS5	1.0354E-02	31	23	27	5	57	46	46	35	Agrin OS=Mus musculus GN=Agrn PE=1 SV=1
Q9CR57	1.0400E-02	9	5	6	0	15	13	11	12	60S ribosomal protein L14 OS=Mus musculus GN=Rpl14 PE=1 SV=3
B7ZWL1	1.0402E-02	14	10	15	3	0	0	0	4	CCR4-NOT transcription complex subunit 1 OS=Mus musculus GN=Cnot1 PE=1 SV=1
Q6ZQ08	1.0402E-02	14	10	15	3	0	0	0	4	CCR4-NOT transcription complex subunit 1 OS=Mus musculus GN=Cnot1 PE=1 SV=2
P80315	1.0444E-02	105	83	101	25	80	50	37	56	T-complex protein 1 subunit delta OS=Mus musculus GN=Cct4 PE=1 SV=3
P70168	1.0504E-02	51	38	49	14	63	52	40	58	Importin subunit beta-1 OS=Mus musculus GN=Kpnb1 PE=1 SV=2
Q3U741	1.0510E-02	39	25	31	12	25	18	12	17	DEAD (Asp-Glu-Ala-Asp) box polypeptide 17, isoform CRA_a OS=Mus musculus GN=Ddx17 PE=1 SV=1
G3UXU2	1.0594E-02	0	0	0	0	3	3	4	5	Casein kinase II subunit beta (Fragment) OS=Mus musculus GN=Csnk2b PE=1 SV=2
P61622	1.0594E-02	0	0	0	0	3	3	4	5	Integrin alpha-11 OS=Mus musculus GN=Itga11 PE=1 SV=1
A0A0B4J1F0	1.0594E-02	0	0	0	0	3	3	4	5	Integrin alpha-11 OS=Mus musculus GN=Itga11 PE=1 SV=1
Q8K2A1	1.0610E-02	0	0	0	0	6	4	3	2	PTB domain-containing engulfment adapter protein 1 OS=Mus musculus GN=Gulp1 PE=1 SV=1
E0CXC5	1.0610E-02	0	0	0	0	6	4	3	2	PTB domain-containing engulfment adapter protein 1 OS=Mus musculus GN=Gulp1 PE=1 SV=1
Q571I9	1.0634E-02	6	4	8	3	0	0	0	0	Aldehyde dehydrogenase family 16 member A1 OS=Mus musculus GN=Aldh16a1 PE=1 SV=2
A0A1B0GSU0	1.0634E-02	6	4	8	3	0	0	0	0	Aldehyde dehydrogenase family 16 member A1 OS=Mus musculus GN=Aldh16a1 PE=1 SV=1
D3Z0B9	1.0634E-02	6	4	8	3	0	0	0	0	Aldehyde dehydrogenase family 16 member A1 OS=Mus musculus GN=Aldh16a1 PE=1 SV=3
P43276	1.0646E-02	11	8	18	5	0	0	0	0	Histone H1.5 OS=Mus musculus GN=Hist1h1b PE=1 SV=2
J3QMG5	1.0694E-02	127	102	101	46	27	25	46	98	Protein Gm5786 OS=Mus musculus GN=Gm5786 PE=3 SV=1
O55131	1.0714E-02	28	22	34	7	63	39	36	41	Septin-7 OS=Mus musculus GN=Sept7 PE=1 SV=1
E9Q1G8	1.0714E-02	28	22	34	7	63	39	36	41	Septin-7 OS=Mus musculus GN=Sept7 PE=1 SV=2
E9Q9F5	1.0714E-02	28	22	34	7	63	39	36	41	Septin-7 OS=Mus musculus GN=Sept7 PE=1 SV=2
E9PXY8	1.0726E-02	15	23	14	5	0	0	0	0	Ubiquitin carboxyl-terminal hydrolase 7 OS=Mus musculus GN=Usp7 PE=1 SV=1
F8VPX1	1.0726E-02	15	23	14	5	0	0	0	0	Ubiquitin carboxyl-terminal hydrolase 7 OS=Mus musculus GN=Usp7 PE=1 SV=1
Q6A4J8	1.0726E-02	15	23	14	5	0	0	0	0	Ubiquitin carboxyl-terminal hydrolase 7 OS=Mus musculus GN=Usp7 PE=1 SV=1
Q8VEE4	1.0818E-02	13	15	14	6	3	8	8	12	Replication protein A 70 kDa DNA-binding subunit OS=Mus musculus GN=Rpa1 PE=1 SV=1
Q3TWF6	1.0825E-02	13	15	15	8	0	0	0	0	WD repeat-containing protein 70 OS=Mus musculus GN=Wdr70 PE=1 SV=1
O35646	1.0936E-02	0	0	0	0	3	5	4	7	Calpain-6 OS=Mus musculus GN=Capn6 PE=1 SV=2
P15379	1.1007E-02	5	5	6	0	14	11	8	11	CD44 antigen OS=Mus musculus GN=Cd44 PE=1 SV=3
Q9JHR7	1.1073E-02	17	14	8	4	6	0	0	5	Insulin-degrading enzyme OS=Mus musculus GN=Ide PE=1 SV=1
Q9D0F9	1.1235E-02	0	0	0	0	8	6	5	13	Phosphoglucomutase-1 OS=Mus musculus GN=Pgm1 PE=1 SV=4
P19324	1.1397E-02	13	6	10	0	33	30	16	28	Serpin H1 OS=Mus musculus GN=Serpinh1 PE=1 SV=3
Q5SWN2	1.1419E-02	13	15	14	6	3	8	8	11	Replication protein A 70 kDa DNA-binding subunit OS=Mus musculus GN=Rpa1 PE=1 SV=2
P97310	1.1443E-02	88	81	92	22	64	67	40	57	DNA replication licensing factor MCM2 OS=Mus musculus GN=Mcm2 PE=1 SV=3
E9QN12	1.1496E-02	0	0	0	0	8	3	6	6	Platelet-derived growth factor receptor beta OS=Mus musculus GN=Pdgfrb PE=1 SV=1
P05622	1.1496E-02	0	0	0	0	8	3	6	6	Platelet-derived growth factor receptor beta OS=Mus musculus GN=Pdgfrb PE=1 SV=1
E9QPE2	1.1496E-02	0	0	0	0	8	3	6	6	Platelet-derived growth factor receptor beta OS=Mus musculus GN=Pdgfrb PE=1 SV=1
Q9QUJ7	1.1602E-02	6	8	3	2	2	3	0	0	Long-chain-fatty-acid--CoA ligase 4 OS=Mus musculus GN=Acsl4 PE=1 SV=2
Q8VDW0	1.1637E-02	37	20	25	5	17	6	6	10	ATP-dependent RNA helicase DDX39A OS=Mus musculus GN=Ddx39a PE=1 SV=1
Q6GQT1	1.1733E-02	0	0	0	0	4	8	4	6	Alpha-2-macroglobulin-P OS=Mus musculus GN=A2m PE=2 SV=2
P26231	1.1905E-02	11	9	11	0	45	31	18	36	Catenin alpha-1 OS=Mus musculus GN=Ctnna1 PE=1 SV=1
B2RUE8	1.1944E-02	0	0	0	0	3	5	5	6	Map4k4 protein OS=Mus musculus GN=Map4k4 PE=1 SV=1
A0A0A6YWM8	1.1944E-02	0	0	0	0	3	5	5	6	Mitogen-activated protein kinase kinase kinase kinase 4 OS=Mus musculus GN=Map4k4 PE=1 SV=1
P97820	1.1961E-02	0	0	0	0	3	4	4	3	Mitogen-activated protein kinase kinase kinase kinase 4 OS=Mus musculus GN=Map4k4 PE=1 SV=1
F7DBB3	1.1974E-02	0	0	0	0	7	4	7	6	Protein Ahnak2 (Fragment) OS=Mus musculus GN=Ahnak2 PE=1 SV=1
Q8VDJ3	1.2054E-02	3	4	3	0	13	9	10	13	Vigilin OS=Mus musculus GN=Hdlbp PE=1 SV=1
P24549	1.2080E-02	0	0	0	0	2	4	2	4	Retinal dehydrogenase 1 OS=Mus musculus GN=Aldh1a1 PE=1 SV=5
Q61235	1.2083E-02	8	0	0	0	12	5	5	4	Beta-2-syntrophin OS=Mus musculus GN=Sntb2 PE=1 SV=2
P10126	1.2181E-02	291	199	255	75	449	342	250	315	Elongation factor 1-alpha 1 OS=Mus musculus GN=Eef1a1 PE=1 SV=3
E9Q8Z5	1.2336E-02	0	5	4	2	26	13	15	24	Catenin delta-1 OS=Mus musculus GN=Ctnnd1 PE=1 SV=1
P97760	1.2553E-02	10	8	14	3	2	0	0	3	DNA-directed RNA polymerase II subunit RPB3 OS=Mus musculus GN=Polr2c PE=1 SV=2
A0A1D5RML8	1.2553E-02	10	8	14	3	2	0	0	3	DNA-directed RNA polymerase II subunit RPB3 OS=Mus musculus GN=Polr2c PE=1 SV=1
Q6ZWQ0	1.2630E-02	6	7	8	4	0	0	0	0	Nesprin-2 OS=Mus musculus GN=Syne2 PE=1 SV=2
E9QP46	1.2630E-02	6	7	8	4	0	0	0	0	Nesprin-2 OS=Mus musculus GN=Syne2 PE=1 SV=1
A0A0U1RPX4	1.2703E-02	27	17	18	6	14	12	0	8	Mitogen-activated protein kinase (Fragment) OS=Mus musculus GN=Mapk3 PE=1 SV=1
P48036	1.2725E-02	73	41	42	16	95	67	59	84	Annexin A5 OS=Mus musculus GN=Anxa5 PE=1 SV=1
P48678	1.2733E-02	20	12	18	7	52	46	33	37	Prelamin-A/C OS=Mus musculus GN=Lmna PE=1 SV=2
A0A140T8V5	1.2813E-02	47	36	38	16	36	26	19	29	Proliferating cell nuclear antigen OS=Mus musculus GN=Pcna-ps2 PE=3 SV=1
G5E839	1.2841E-02	98	75	93	22	71	47	34	54	T-complex protein 1 subunit delta OS=Mus musculus GN=Cct4 PE=1 SV=1
O09106	1.2940E-02	27	24	31	15	11	14	6	21	Histone deacetylase 1 OS=Mus musculus GN=Hdac1 PE=1 SV=1
O70133	1.2983E-02	148	84	90	23	38	34	22	39	ATP-dependent RNA helicase A OS=Mus musculus GN=Dhx9 PE=1 SV=2
P80317	1.2997E-02	120	100	107	23	79	59	51	59	T-complex protein 1 subunit zeta OS=Mus musculus GN=Cct6a PE=1 SV=3
P50580	1.3072E-02	41	36	53	11	23	19	15	21	Proliferation-associated protein 2G4 OS=Mus musculus GN=Pa2g4 PE=1 SV=3
A2AE89	1.3079E-02	5	0	0	0	9	8	4	7	Glutathione S-transferase Mu 1 OS=Mus musculus GN=Gstm1 PE=1 SV=2
P10649	1.3079E-02	5	0	0	0	9	8	4	7	Glutathione S-transferase Mu 1 OS=Mus musculus GN=Gstm1 PE=1 SV=2
P62267	1.3122E-02	44	34	33	6	9	3	6	7	40S ribosomal protein S23 OS=Mus musculus GN=Rps23 PE=1 SV=3
A0A087WP83	1.3247E-02	3	4	3	0	12	9	9	13	Vigilin OS=Mus musculus GN=Hdlbp PE=1 SV=1
P84104	1.3530E-02	9	5	5	3	5	0	2	4	Serine/arginine-rich splicing factor 3 OS=Mus musculus GN=Srsf3 PE=1 SV=1
P62965	1.3542E-02	0	0	0	0	4	9	5	7	Cellular retinoic acid-binding protein 1 OS=Mus musculus GN=Crabp1 PE=1 SV=2
Q99PT1	1.3659E-02	19	11	8	0	42	46	40	53	Rho GDP-dissociation inhibitor 1 OS=Mus musculus GN=Arhgdia PE=1 SV=3
G3UZ34	1.3698E-02	27	21	22	5	10	11	10	11	116 kDa U5 small nuclear ribonucleoprotein component OS=Mus musculus GN=Eftud2 PE=1 SV=1

P38647	1.3733E-02	0	0	0	0	7	5	2	3	Stress-70 protein, mitochondrial OS=Mus musculus GN=Hspa9 PE=1 SV=3
P12382	1.3745E-02	43	36	35	9	23	15	6	20	ATP-dependent 6-phosphofructokinase, liver type OS=Mus musculus GN=Pfkl PE=1 SV=4
Q9CZU6	1.3792E-02	31	23	21	8	16	15	10	22	Citrate synthase, mitochondrial OS=Mus musculus GN=Cs PE=1 SV=1
E9PWE8	1.3936E-02	0	0	0	0	39	29	8	29	Dihydropyrimidinase-related protein 3 OS=Mus musculus GN=Dpysl3 PE=1 SV=1
D3Z3G6	1.4155E-02	28	17	19	6	13	12	0	8	Mitogen-activated protein kinase OS=Mus musculus GN=Mapk3 PE=1 SV=1
D3YV80	1.4229E-02	0	2	2	0	3	5	3	5	Unconventional myosin-Ib (Fragment) OS=Mus musculus GN=Myo1b PE=1 SV=1
Q07113	1.4478E-02	4	0	0	0	11	15	11	18	Cation-independent mannose-6-phosphate receptor OS=Mus musculus GN=Igf2r PE=1 SV=1
Q01768	1.4502E-02	22	20	22	2	48	38	39	49	Nucleoside diphosphate kinase B OS=Mus musculus GN=Nme2 PE=1 SV=1
Q6P5F7	1.4585E-02	2	3	0	0	7	6	5	7	Protein tweety homolog 3 OS=Mus musculus GN=Ttyh3 PE=1 SV=1
A0A0J9YUL3	1.4623E-02	18	18	17	5	39	26	26	31	Septin 11, isoform CRA_b OS=Mus musculus GN=Sept11 PE=1 SV=1
Q8C1B7	1.4623E-02	18	18	17	5	39	26	26	31	Septin-11 OS=Mus musculus GN=Sept11 PE=1 SV=4
A0A0J9YTY0	1.4623E-02	18	18	17	5	39	26	26	31	Septin-11 OS=Mus musculus GN=Sept11 PE=1 SV=1
Q60864	1.4640E-02	22	16	25	7	43	40	28	34	Stress-induced-phosphoprotein 1 OS=Mus musculus GN=Stip1 PE=1 SV=1
Q5SUR0	1.4697E-02	24	19	22	5	18	15	13	15	Phosphoribosylformylglycinamidine synthase OS=Mus musculus GN=Pfas PE=1 SV=1
P26043	1.4775E-02	51	41	38	10	86	78	74	72	Radixin OS=Mus musculus GN=Rdx PE=1 SV=3
E9Q616	1.4784E-02	29	25	21	14	54	53	51	81	Protein Ahnak OS=Mus musculus GN=Ahnak PE=1 SV=1
Q9D281	1.4866E-02	0	0	0	0	8	10	5	4	Protein Noxp20 OS=Mus musculus GN=Fam114a1 PE=1 SV=1
Q9R0B9	1.4968E-02	29	20	29	4	0	3	0	3	Procollagen-lysine,2-oxoglutarate 5-dioxygenase 2 OS=Mus musculus GN=Plod2 PE=1 SV=2
E9Q718	1.4968E-02	29	20	29	4	0	3	0	3	Procollagen-lysine,2-oxoglutarate 5-dioxygenase 2 OS=Mus musculus GN=Plod2 PE=1 SV=1
Q8BKC5	1.4986E-02	26	21	21	10	10	9	8	7	Importin-5 OS=Mus musculus GN=Ipo5 PE=1 SV=3
A0A0R4J008	1.5163E-02	17	12	19	2	10	0	5	0	Histone deacetylase OS=Mus musculus GN=Hdac2 PE=1 SV=1
P11087	1.5241E-02	0	0	0	0	43	36	13	19	Collagen alpha-1(I) chain OS=Mus musculus GN=Col1a1 PE=1 SV=4
Q08093	1.5428E-02	0	0	0	0	7	5	6	3	Calponin-2 OS=Mus musculus GN=Cnn2 PE=1 SV=1
P07356	1.5573E-02	123	89	75	15	208	135	97	156	Annexin A2 OS=Mus musculus GN=Anxa2 PE=1 SV=2
Q99LD8	1.5789E-02	0	5	4	0	24	15	9	17	N(G),N(G)-dimethylarginine dimethylaminohydrolase 2 OS=Mus musculus GN=Ddah2 PE=1 SV=1
O88544	1.5820E-02	17	8	12	3	7	4	0	4	COP9 signalosome complex subunit 4 OS=Mus musculus GN=Cops4 PE=1 SV=1
D3YYI8	1.5919E-02	26	24	31	15	11	14	6	20	Histone deacetylase OS=Mus musculus GN=Gm10093 PE=3 SV=1
O08529	1.5974E-02	24	17	12	4	12	8	2	10	Calpain-2 catalytic subunit OS=Mus musculus GN=Capn2 PE=1 SV=4
P16045	1.6282E-02	62	21	51	5	100	84	83	111	Galectin-1 OS=Mus musculus GN=Lgals1 PE=1 SV=3
P51410	1.6287E-02	14	10	11	6	10	3	0	8	60S ribosomal protein L9 OS=Mus musculus GN=Rpl9 PE=2 SV=2
A0A0G2JES3	1.6287E-02	14	10	11	6	10	3	0	8	60S ribosomal protein L9 (Fragment) OS=Mus musculus GN=Rpl9 PE=1 SV=1
D3Z795	1.6341E-02	13	6	7	3	6	0	0	8	Proteasome assembly chaperone 1 OS=Mus musculus GN=Psmg1 PE=1 SV=1
Q9JK23	1.6341E-02	13	6	7	3	6	0	0	8	Proteasome assembly chaperone 1 OS=Mus musculus GN=Psmg1 PE=1 SV=1
Q8BZY3	1.6354E-02	6	4	6	0	9	8	9	4	Protein Ddx19b OS=Mus musculus GN=Ddx19b PE=1 SV=1
Q8CG48	1.6731E-02	13	6	3	2	2	0	0	0	Structural maintenance of chromosomes protein 2 OS=Mus musculus GN=Smc2 PE=1 SV=2
O35593	1.6755E-02	10	10	16	5	6	6	8	7	26S proteasome non-ATPase regulatory subunit 14 OS=Mus musculus GN=Psmd14 PE=1 SV=2
Q924C1	1.6777E-02	14	13	11	2	0	4	0	3	Exportin-5 OS=Mus musculus GN=Xpo5 PE=1 SV=1
Q99L47	1.6949E-02	12	14	6	3	23	19	13	21	Hsc70-interacting protein OS=Mus musculus GN=St13 PE=1 SV=1
P54775	1.7087E-02	60	78	77	40	27	27	35	66	26S protease regulatory subunit 6B OS=Mus musculus GN=Psmc4 PE=1 SV=2
Q3U6K9	1.7100E-02	8	2	6	0	24	33	16	28	Phosphoserine aminotransferase OS=Mus musculus GN=Psat1 PE=1 SV=1
A0A0J9YUD8	1.7142E-02	5	7	5	0	9	15	11	10	High mobility group protein B1 OS=Mus musculus GN=Hmgb1 PE=1 SV=1
Q61598	1.7222E-02	18	14	14	2	55	29	21	39	Rab GDP dissociation inhibitor beta OS=Mus musculus GN=Gdi2 PE=1 SV=1
Q62188	1.7362E-02	0	0	0	0	39	29	7	29	Dihydropyrimidinase-related protein 3 OS=Mus musculus GN=Dpysl3 PE=1 SV=1
P26039	1.7463E-02	27	23	28	7	67	41	30	50	Talin-1 OS=Mus musculus GN=Tln1 PE=1 SV=2
Q9JIM1	1.7471E-02	0	7	9	0	8	10	10	7	Equilibrative nucleoside transporter 1 OS=Mus musculus GN=Slc29a1 PE=1 SV=3
P47757	1.7695E-02	26	23	26	9	46	51	29	43	F-actin-capping protein subunit beta OS=Mus musculus GN=Capzb PE=1 SV=3
G3X9V0	1.7823E-02	0	0	6	0	5	6	6	6	MCG22048, isoform CRA_a OS=Mus musculus GN=Psme2 PE=1 SV=1
Q8BY89	1.7839E-02	11	8	8	2	18	20	17	25	Choline transporter-like protein 2 OS=Mus musculus GN=Slc44a2 PE=1 SV=2
G3XA10	1.7881E-02	45	36	52	14	37	23	19	28	Heterogeneous nuclear ribonucleoprotein U OS=Mus musculus GN=Hnrnpu PE=1 SV=1
Q8VEK3	1.7881E-02	45	36	52	14	37	23	19	28	Heterogeneous nuclear ribonucleoprotein U OS=Mus musculus GN=Hnrnpu PE=1 SV=1
P61358	1.7903E-02	23	13	16	4	18	6	5	10	60S ribosomal protein L27 OS=Mus musculus GN=Rpl27 PE=1 SV=2
Q61881	1.8008E-02	66	63	64	32	33	18	15	21	DNA replication licensing factor MCM7 OS=Mus musculus GN=Mcm7 PE=1 SV=1
Q9QUR6	1.8083E-02	70	48	63	14	32	17	10	32	Prolyl endopeptidase OS=Mus musculus GN=Prep PE=1 SV=1
Q9CXF4	1.8088E-02	11	8	3	2	0	0	0	0	TBC1 domain family member 15 OS=Mus musculus GN=Tbc1d15 PE=1 SV=1
Q8BU30	1.8119E-02	78	59	50	14	24	20	19	28	Isoleucine--tRNA ligase, cytoplasmic OS=Mus musculus GN=Iars PE=1 SV=2
O88796	1.8161E-02	5	4	5	3	0	0	0	0	Ribonuclease P protein subunit p30 OS=Mus musculus GN=Rpp30 PE=1 SV=1
E9PZS4	1.8233E-02	8	5	5	3	4	0	0	0	NHP2-like protein 1 OS=Mus musculus GN=Nhp2l1 PE=1 SV=1
Q99K51	1.8264E-02	0	0	0	0	3	7	6	6	Plastin-3 OS=Mus musculus GN=Pls3 PE=1 SV=3
B1AX58	1.8264E-02	0	0	0	0	3	7	6	6	Plastin-3 OS=Mus musculus GN=Pls3 PE=1 SV=1
A0A1C7CYV0	1.8264E-02	0	0	0	0	3	7	6	6	Plastin-3 (Fragment) OS=Mus musculus GN=Pls3 PE=1 SV=1
Q99K85	1.8362E-02	8	2	8	0	30	41	19	38	Phosphoserine aminotransferase OS=Mus musculus GN=Psat1 PE=1 SV=1
P62962	1.8547E-02	53	57	56	18	73	104	70	89	Profilin-1 OS=Mus musculus GN=Pfn1 PE=1 SV=2
P35550	1.8548E-02	19	21	11	7	0	0	3	2	rRNA 2'-O-methyltransferase fibrillarin OS=Mus musculus GN=Fbl PE=1 SV=2
P27773	1.8679E-02	6	5	8	2	14	14	8	13	Protein disulfide-isomerase A3 OS=Mus musculus GN=Pdia3 PE=1 SV=2
Q923B1	1.8775E-02	7	10	4	3	0	0	0	0	Lariat debranching enzyme OS=Mus musculus GN=Dbr1 PE=1 SV=2
A0A0G2JDF8	1.8823E-02	7	8	10	3	5	2	3	4	Serrate RNA effector molecule homolog (Fragment) OS=Mus musculus GN=Srrt PE=1 SV=1
Q64727	1.9078E-02	23	14	17	7	85	46	28	48	Vinculin OS=Mus musculus GN=Vcl PE=1 SV=4
P99024	1.9142E-02	327	210	262	46	440	317	232	304	Tubulin beta-5 chain OS=Mus musculus GN=Tubb5 PE=1 SV=1
O08810	1.9259E-02	28	22	23	5	10	11	10	12	116 kDa U5 small nuclear ribonucleoprotein component OS=Mus musculus GN=Eftud2 PE=1 SV=1
A2AH85	1.9259E-02	28	22	23	5	10	11	10	12	116 kDa U5 small nuclear ribonucleoprotein component OS=Mus musculus GN=Eftud2 PE=1 SV=1
Q99MR6	1.9436E-02	10	9	16	3	5	2	3	4	Serrate RNA effector molecule homolog OS=Mus musculus GN=Srrt PE=1 SV=1
Q7TPV4	1.9451E-02	41	44	41	10	18	17	13	24	Myb-binding protein 1A OS=Mus musculus GN=Mybbp1a PE=1 SV=2
Q8R2Q8	1.9477E-02	6	0	8	0	12	11	10	7	Bone marrow stromal antigen 2 OS=Mus musculus GN=Bst2 PE=1 SV=1
D3Z7D5	1.9536E-02	0	0	0	0	8	5	2	3	Collagen alpha-2(VI) chain OS=Mus musculus GN=Col6a2 PE=1 SV=1
P63101	1.9830E-02	78	75	78	35	54	31	42	70	14-3-3 protein zeta/delta OS=Mus musculus GN=Ywhaz PE=1 SV=1
Q922F4	1.9995E-02	209	150	180	38	311	233	152	186	Tubulin beta-6 chain OS=Mus musculus GN=Tubb6 PE=1 SV=1
Q9CZN7	2.0166E-02	0	3	5	0	19	14	7	15	Serine hydroxymethyltransferase OS=Mus musculus GN=Shmt2 PE=1 SV=1
Q62318	2.0297E-02	16	18	22	7	11	4	3	6	Transcription intermediary factor 1-beta OS=Mus musculus GN=Trim28 PE=1 SV=3
Q99JI4	2.0300E-02	45	35	36	11	14	23	19	25	26S proteasome non-ATPase regulatory subunit 6 OS=Mus musculus GN=Psmd6 PE=1 SV=1
F8WJK8	2.0318E-02	12	14	6	4	23	19	13	21	Hsc70-interacting protein OS=Mus musculus GN=St13 PE=1 SV=1
Q9R190	2.0435E-02	24	14	10	5	3	4	3	7	Metastasis-associated protein MTA2 OS=Mus musculus GN=Mta2 PE=1 SV=1
P09103	2.0891E-02	3	0	4	0	14	20	9	16	Protein disulfide-isomerase OS=Mus musculus GN=P4hb PE=1 SV=2
P05201	2.1068E-02	2	0	0	0	3	2	2	2	Aspartate aminotransferase, cytoplasmic OS=Mus musculus GN=Got1 PE=1 SV=3
Q8VDM6	2.1105E-02	12	8	10	4	7	3	4	2	Heterogeneous nuclear ribonucleoprotein U-like protein 1 OS=Mus musculus GN=Hnrnpul1 PE=1 SV=1
Q8C8U0	2.1148E-02	0	0	0	0	22	10	4	14	Liprin-beta-1 OS=Mus musculus GN=Ppfibp1 PE=1 SV=3
D3Z6N3	2.1164E-02	25	20	23	8	17	14	15	15	DNA replication licensing factor MCM7 OS=Mus musculus GN=Mcm7 PE=1 SV=1
Q8C7V3	2.1181E-02	4	4	2	2	0	0	0	0	U3 small nucleolar RNA-associated protein 15 homolog OS=Mus musculus GN=Utp15 PE=1 SV=1
Q6PAR5	2.1295E-02	8	9	4	4	0	0	0	0	GTPase-activating protein and VPS9 domain-containing protein 1 OS=Mus musculus GN=Gapvd1 PE=1 SV=2
P17182	2.1348E-02	172	144	148	38	127	103	72	115	Alpha-enolase OS=Mus musculus GN=Eno1 PE=1 SV=3
A2AMW0	2.1410E-02	26	23	26	9	49	55	29	45	Capping protein (Actin filament) muscle Z-line, beta, isoform CRA_a OS=Mus musculus GN=Capzb PE=1 SV=1
D3YWF6	2.1469E-02	14	12	12	6	9	10	8	18	Ubiquitin thioesterase OTUB1 OS=Mus musculus GN=Otub1 PE=1 SV=1
Q7TQI3	2.1469E-02	14	12	12	6	9	10	8	18	Ubiquitin thioesterase OTUB1 OS=Mus musculus GN=Otub1 PE=1 SV=2
A0A0G2JGZ5	2.1633E-02	0	0	0	0	12	6	2	8	PDZ and LIM domain protein 5 (Fragment) OS=Mus musculus GN=Pdlim5 PE=1 SV=1
Q8C052	2.1685E-02	12	12	6	4	16	16	9	17	Microtubule-associated protein 1S OS=Mus musculus GN=Map1s PE=1 SV=2
P16546	2.1823E-02	21	12	18	3	29	26	19	27	Spectrin alpha chain, non-erythrocytic 1 OS=Mus musculus GN=Sptan1 PE=1 SV=4
A0A0U1RQ05	2.2058E-02	38	26	23	17	12	12	7	18	Cytoplasmic FMR1-interacting protein 1 (Fragment) OS=Mus musculus GN=Cyfip1 PE=1 SV=1
E9PZF0	2.2299E-02	22	25	23	3	52	40	44	55	Nucleoside diphosphate kinase OS=Mus musculus GN=Gm20390 PE=3 SV=1
B1AWE1	2.2323E-02	21	15	13	8	8	5	7	11	Clathrin light chain A OS=Mus musculus GN=Clta PE=1 SV=1
B1AZ46	2.2450E-02	3	2	0	0	10	5	4	10	Brain-specific angiogenesis inhibitor 1-associated protein 2 OS=Mus musculus GN=Baiap2 PE=1 SV=1
Q8BKX1	2.2450E-02	3	2	0	0	10	5	4	10	Brain-specific angiogenesis inhibitor 1-associated protein 2 OS=Mus musculus GN=Baiap2 PE=1 SV=2
Q8JZK9	2.2527E-02	24	14	24	2	0	0	0	0	Hydroxymethylglutaryl-CoA synthase, cytoplasmic OS=Mus musculus GN=Hmgcs1 PE=1 SV=1
Q9R0P5	2.2736E-02	14	11	10	3	29	23	18	14	Destrin OS=Mus musculus GN=Dstn PE=1 SV=3
A2APM1	2.2903E-02	5	5	7	0	14	10	8	11	CD44 antigen OS=Mus musculus GN=Cd44 PE=1 SV=1
Q9CWJ9	2.3065E-02	92	78	62	36	69	46	31	64	Bifunctional purine biosynthesis protein PURH OS=Mus musculus GN=Atic PE=1 SV=2
A2APM5	2.3080E-02	5	5	7	0	14	11	8	12	CD44 antigen OS=Mus musculus GN=Cd44 PE=1 SV=1
Q80X37	2.3080E-02	5	5	7	0	14	11	8	12	CD44 antigen OS=Mus musculus GN=Cd44 PE=1 SV=1
A6PWY4	2.3586E-02	12	11	19	9	0	0	0	0	WD repeat-containing protein 76 OS=Mus musculus GN=Wdr76 PE=1 SV=1
P10404	2.3628E-02	20	9	13	9	0	0	0	0	MLV-related proviral Env polyprotein OS=Mus musculus PE=1 SV=3
Q549C9	2.3862E-02	25	24	20	2	0	0	0	0	Cellular tumor antigen p53 OS=Mus musculus GN=Trp53 PE=1 SV=1
P02340	2.3862E-02	25	24	20	2	0	0	0	0	Cellular tumor antigen p53 OS=Mus musculus GN=Tp53 PE=1 SV=3
Q80ZA1	2.3862E-02	25	24	20	2	0	0	0	0	Cellular tumor antigen p53 OS=Mus musculus GN=Trp53 PE=1 SV=1
I7HIK9	2.3862E-02	25	24	20	2	0	0	0	0	Cellular tumor antigen p53 OS=Mus musculus GN=Trp53 PE=1 SV=1
Q5SF07	2.3864E-02	6	2	4	0	10	12	7	9	Insulin-like growth factor 2 mRNA-binding protein 2 OS=Mus musculus GN=Igf2bp2 PE=1 SV=1
Q9D1M4	2.3879E-02	9	7	6	5	0	0	0	0	Eukaryotic translation elongation factor 1 epsilon-1 OS=Mus musculus GN=Eef1e1 PE=1 SV=1
Q91W50	2.4269E-02	7	4	7	4	0	0	0	0	Cold shock domain-containing protein E1 OS=Mus musculus GN=Csde1 PE=1 SV=1
A6X8Z3	2.4269E-02	6	2	4	0	10	12	7	7	Insulin-like growth factor 2 mRNA-binding protein 2 OS=Mus musculus GN=Igf2bp2 PE=1 SV=1
Q8BH35	2.4283E-02	0	0	0	0	4	5	7	5	Complement component C8 beta chain OS=Mus musculus GN=C8b PE=1 SV=1
Q3TTF6	2.4930E-02	4	8	6	2	0	0	0	3	Putative uncharacterized protein OS=Mus musculus GN=Ppp2r1b PE=1 SV=1
H3BKU1	2.4930E-02	4	8	6	2	0	0	0	3	Protein phosphatase 2 (Formerly 2A), regulatory subunit A (PR 65), beta isoform, isoform CRA_b OS=Mus musculus GN=Ppp2r1b PE=1 SV=1
G3UWS4	2.4930E-02	4	8	6	2	0	0	0	3	Serine/threonine-protein phosphatase 2A 65 kDa regulatory subunit A beta isoform OS=Mus musculus GN=Ppp2r1b PE=1 SV=1
H3BLQ0	2.4930E-02	4	8	6	2	0	0	0	3	Serine/threonine-protein phosphatase 2A 65 kDa regulatory subunit A beta isoform OS=Mus musculus GN=Ppp2r1b PE=1 SV=1
E9Q0U1	2.4974E-02	18	11	17	3	4	5	5	7	26S proteasome non-ATPase regulatory subunit 13 OS=Mus musculus GN=Psmd13 PE=1 SV=1
P43247	2.5520E-02	14	10	3	3	0	0	0	2	DNA mismatch repair protein Msh2 OS=Mus musculus GN=Msh2 PE=1 SV=1
V9GXB6	2.5604E-02	3	3	4	2	0	2	0	5	Heterogeneous nuclear ribonucleoprotein L-like OS=Mus musculus GN=Hnrnpll PE=1 SV=1
Q6P2B1	2.5639E-02	9	9	6	4	6	0	0	3	Transportin-3 OS=Mus musculus GN=Tnpo3 PE=1 SV=1
Q07797	2.5674E-02	14	15	14	0	42	38	17	37	Galectin-3-binding protein OS=Mus musculus GN=Lgals3bp PE=1 SV=1
A6ZI44	2.5873E-02	66	75	60	26	133	113	84	106	Fructose-bisphosphate aldolase OS=Mus musculus GN=Aldoa PE=1 SV=1
E9QNH6	2.5888E-02	0	2	2	0	4	6	3	7	Unconventional myosin-Ib OS=Mus musculus GN=Myo1b PE=1 SV=1
E9Q580	2.5888E-02	0	2	2	0	4	6	3	7	Unconventional myosin-Ib OS=Mus musculus GN=Myo1b PE=1 SV=1
P46735	2.5888E-02	0	2	2	0	4	6	3	7	Unconventional myosin-Ib OS=Mus musculus GN=Myo1b PE=1 SV=3
Q7TQD7	2.5888E-02	0	2	2	0	4	6	3	7	Myo1b protein OS=Mus musculus GN=Myo1b PE=1 SV=1
P80313	2.5985E-02	92	78	77	18	63	38	32	48	T-complex protein 1 subunit eta OS=Mus musculus GN=Cct7 PE=1 SV=1
D9J2Z9	2.6009E-02	0	0	0	0	13	6	2	8	ENH isoform 1b OS=Mus musculus GN=Pdlim5 PE=1 SV=1
Q8CI51	2.6009E-02	0	0	0	0	13	6	2	8	PDZ and LIM domain protein 5 OS=Mus musculus GN=Pdlim5 PE=1 SV=4
D9J301	2.6009E-02	0	0	0	0	13	6	2	8	ENH isoform 1d OS=Mus musculus GN=Pdlim5 PE=1 SV=1
D9J300	2.6009E-02	0	0	0	0	13	6	2	8	ENH isoform 1c OS=Mus musculus GN=Pdlim5 PE=1 SV=1
D9J302	2.6009E-02	0	0	0	0	13	6	2	8	ENH isoform 1e OS=Mus musculus GN=Pdlim5 PE=1 SV=1
E9Q1S3	2.6048E-02	0	0	0	0	5	12	4	9	Protein transport protein Sec23A OS=Mus musculus GN=Sec23a PE=1 SV=1
Q01405	2.6048E-02	0	0	0	0	5	12	4	9	Protein transport protein Sec23A OS=Mus musculus GN=Sec23a PE=1 SV=2

G3X9K9	2.6066E-02	0	0	9	0	5	12	11	8	Proteasome activator complex subunit 1 OS=Mus musculus GN=Psme1 PE=1 SV=1
P62071	2.6161E-02	9	0	3	0	13	13	12	13	Ras-related protein R-Ras2 OS=Mus musculus GN=Rras2 PE=1 SV=1
Q6ZWV7	2.6250E-02	0	0	4	0	6	6	4	5	60S ribosomal protein L35 OS=Mus musculus GN=Rpl35 PE=1 SV=1
E9Q0K6	2.6470E-02	30	28	25	3	2	3	0	4	Deoxynucleoside triphosphate triphosphohydrolase SAMHD1 OS=Mus musculus GN=Samhd1 PE=1 SV=1
Q60710	2.6988E-02	30	28	27	3	2	3	0	4	Deoxynucleoside triphosphate triphosphohydrolase SAMHD1 OS=Mus musculus GN=Samhd1 PE=1 SV=2
F8WJE0	2.6988E-02	30	28	27	3	2	3	0	4	Deoxynucleoside triphosphate triphosphohydrolase SAMHD1 OS=Mus musculus GN=Samhd1 PE=1 SV=1
P07742	2.7021E-02	116	75	78	11	10	14	0	18	Ribonucleoside-diphosphate reductase large subunit OS=Mus musculus GN=Rrm1 PE=1 SV=2
Q61151	2.7081E-02	12	7	8	4	8	0	4	5	Serine/threonine-protein phosphatase 2A 56 kDa regulatory subunit epsilon isoform OS=Mus musculus GN=Ppp2r5e PE=1 SV=3
P50396	2.7088E-02	8	5	4	2	18	19	19	16	Rab GDP dissociation inhibitor alpha OS=Mus musculus GN=Gdi1 PE=1 SV=3
A0A0A6YY91	2.7603E-02	0	4	0	0	9	6	6	8	Neural cell adhesion molecule 1 (Fragment) OS=Mus musculus GN=Ncam1 PE=1 SV=1
D3Z2T9	2.7631E-02	46	31	42	5	18	13	14	12	Cyclin-dependent kinase 1 (Fragment) OS=Mus musculus GN=Cdk1 PE=1 SV=1
P59708	2.7736E-02	0	6	6	0	6	8	7	6	Splicing factor 3B subunit 6 OS=Mus musculus GN=Sf3b6 PE=1 SV=1
A2BH06	2.7781E-02	33	28	38	16	24	15	16	22	60S ribosomal protein L11 (Fragment) OS=Mus musculus GN=Rpl11 PE=1 SV=1
F6SVV1	2.7829E-02	12	8	5	3	6	4	3	5	Protein Gm9493 OS=Mus musculus GN=Gm9493 PE=4 SV=1
A0A1B0GRR3	2.7906E-02	26	17	24	4	18	13	12	12	40S ribosomal protein S11 OS=Mus musculus GN=Rps11 PE=1 SV=1
P70349	2.7930E-02	4	6	8	0	22	12	10	10	Histidine triad nucleotide-binding protein 1 OS=Mus musculus GN=Hint1 PE=1 SV=3
P11688	2.8182E-02	5	0	3	0	37	24	6	27	Integrin alpha-5 OS=Mus musculus GN=Itga5 PE=1 SV=3
Q8R010	2.8209E-02	43	39	42	30	11	0	0	10	Aminoacyl tRNA synthase complex-interacting multifunctional protein 2 OS=Mus musculus GN=Aimp2 PE=1 SV=2
P05064	2.8302E-02	65	75	60	26	133	113	84	105	Fructose-bisphosphate aldolase A OS=Mus musculus GN=Aldoa PE=1 SV=2
Q3TY56	2.8352E-02	5	5	3	3	0	0	0	0	Eukaryotic translation initiation factor 3 subunit K OS=Mus musculus GN=Eif3k PE=1 SV=1
P62631	2.8516E-02	144	109	133	38	193	198	158	162	Elongation factor 1-alpha 2 OS=Mus musculus GN=Eef1a2 PE=1 SV=1
Q8JZV7	2.8676E-02	2	0	4	0	7	6	4	5	N-acetylglucosamine-6-phosphate deacetylase OS=Mus musculus GN=Amdhd2 PE=1 SV=1
P01942	2.8798E-02	0	0	0	0	3	8	4	4	Hemoglobin subunit alpha OS=Mus musculus GN=Hba PE=1 SV=2
Q91VB8	2.8798E-02	0	0	0	0	3	8	4	4	Alpha globin 1 OS=Mus musculus GN=haemaglobin alpha 2 PE=1 SV=1
E9Q390	2.8964E-02	104	81	83	20	149	139	110	184	Myoferlin OS=Mus musculus GN=Myof PE=1 SV=2
A2APM3	2.9386E-02	5	5	7	0	14	10	8	12	CD44 antigen OS=Mus musculus GN=Cd44 PE=1 SV=1
A2APM4	2.9386E-02	5	5	7	0	14	10	8	12	CD44 antigen OS=Mus musculus GN=Cd44 PE=1 SV=1
A2APM2	2.9386E-02	5	5	7	0	14	10	8	12	CD44 antigen OS=Mus musculus GN=Cd44 PE=1 SV=1
E9QKM8	2.9386E-02	5	5	7	0	14	10	8	12	CD44 antigen OS=Mus musculus GN=Cd44 PE=1 SV=1
Q3U8S1	2.9386E-02	5	5	7	0	14	10	8	12	CD44 antigen OS=Mus musculus GN=Cd44 PE=1 SV=1
P54754	2.9759E-02	0	0	5	0	5	7	5	6	Ephrin type-B receptor 3 OS=Mus musculus GN=Ephb3 PE=1 SV=2
contaminant_KERATIN06	2.9775E-02	12	6	13	0	19	19	13	8	no description
Q69ZN7	2.9905E-02	105	81	83	20	149	139	110	184	Myoferlin OS=Mus musculus GN=Myof PE=1 SV=2
P62960	3.0311E-02	40	29	27	9	21	15	16	23	Nuclease-sensitive element-binding protein 1 OS=Mus musculus GN=Ybx1 PE=1 SV=3
P17426	3.0555E-02	8	5	4	0	13	10	5	9	AP-2 complex subunit alpha-1 OS=Mus musculus GN=Ap2a1 PE=1 SV=1
P13595	3.0679E-02	0	4	0	0	10	6	6	10	Neural cell adhesion molecule 1 OS=Mus musculus GN=Ncam1 PE=1 SV=3
E9QB01	3.0679E-02	0	4	0	0	10	6	6	10	Neural cell adhesion molecule 1 OS=Mus musculus GN=Ncam1 PE=1 SV=2
A0A0A6YY47	3.0679E-02	0	4	0	0	10	6	6	10	Neural cell adhesion molecule 1 OS=Mus musculus GN=Ncam1 PE=1 SV=1
Q05816	3.0910E-02	5	4	4	0	48	34	8	25	Fatty acid-binding protein, epidermal OS=Mus musculus GN=Fabp5 PE=1 SV=3
Q64282	3.1493E-02	2	0	0	0	8	10	13	9	Interferon-induced protein with tetratricopeptide repeats 1 OS=Mus musculus GN=Ifit1 PE=1 SV=2
Q9DBZ5	3.1550E-02	7	7	4	3	5	0	0	3	Eukaryotic translation initiation factor 3 subunit K OS=Mus musculus GN=Eif3k PE=1 SV=1
Q3ULG5	3.1860E-02	55	41	42	10	26	23	16	30	DNA helicase OS=Mus musculus GN=Mcm6 PE=1 SV=1
P97311	3.1860E-02	55	41	42	10	26	23	16	30	DNA replication licensing factor MCM6 OS=Mus musculus GN=Mcm6 PE=1 SV=1
Q9ESX5	3.2023E-02	11	10	11	0	22	15	18	18	H/ACA ribonucleoprotein complex subunit 4 OS=Mus musculus GN=Dkc1 PE=1 SV=4
Q921M3	3.2054E-02	16	11	21	4	9	7	8	11	Splicing factor 3B subunit 3 OS=Mus musculus GN=Sf3b3 PE=1 SV=1
Q04750	3.2434E-02	15	20	22	10	8	8	4	5	DNA topoisomerase 1 OS=Mus musculus GN=Top1 PE=1 SV=2
Q61205	3.2543E-02	0	0	0	0	2	7	4	4	Platelet-activating factor acetylhydrolase IB subunit gamma OS=Mus musculus GN=Pafah1b3 PE=1 SV=1
D3Z7E6	3.2543E-02	0	0	0	0	2	7	4	4	Platelet-activating factor acetylhydrolase IB subunit gamma OS=Mus musculus GN=Pafah1b3 PE=1 SV=1
O70194	3.2741E-02	22	16	17	2	15	8	7	5	Eukaryotic translation initiation factor 3 subunit D OS=Mus musculus GN=Eif3d PE=1 SV=2
B1AWE0	3.2768E-02	21	15	13	8	7	5	8	11	Clathrin light chain A OS=Mus musculus GN=Clta PE=1 SV=1
B1AWD9	3.2768E-02	21	15	13	8	7	5	8	11	Clathrin light chain A OS=Mus musculus GN=Clta PE=1 SV=1
Q6PFA2	3.2768E-02	21	15	13	8	7	5	8	11	Clathrin light chain A OS=Mus musculus GN=Clta PE=1 SV=1
O08585	3.2768E-02	21	15	13	8	7	5	8	11	Clathrin light chain A OS=Mus musculus GN=Clta PE=1 SV=2
P97371	3.2811E-02	0	0	10	0	5	12	11	8	Proteasome activator complex subunit 1 OS=Mus musculus GN=Psme1 PE=1 SV=2
Q05D44	3.3251E-02	7	9	4	2	2	6	2	5	Eukaryotic translation initiation factor 5B OS=Mus musculus GN=Eif5b PE=1 SV=2
Q7TSG6	3.3599E-02	50	39	38	0	69	72	65	62	Radixin OS=Mus musculus GN=Rdx PE=1 SV=1
P05132	3.3724E-02	3	2	5	0	9	13	6	8	cAMP-dependent protein kinase catalytic subunit alpha OS=Mus musculus GN=Prkaca PE=1 SV=3
Q8VDD5	3.3863E-02	272	183	227	83	409	303	230	305	Myosin-9 OS=Mus musculus GN=Myh9 PE=1 SV=4
Q4VA53	3.3950E-02	18	4	14	2	0	0	0	0	Sister chromatid cohesion protein PDS5 homolog B OS=Mus musculus GN=Pds5b PE=1 SV=1
F8WHU5	3.3950E-02	18	4	14	2	0	0	0	0	Sister chromatid cohesion protein PDS5 homolog B OS=Mus musculus GN=Pds5b PE=1 SV=1
A0A0A0MQ76	3.4142E-02	16	14	18	2	8	4	4	4	Nucleolar protein 58 OS=Mus musculus GN=Nop58 PE=1 SV=1
Q9CXW4	3.4280E-02	39	30	43	17	30	23	16	27	60S ribosomal protein L11 OS=Mus musculus GN=Rpl11 PE=1 SV=4
E9PYL9	3.4280E-02	39	30	43	17	30	23	16	27	Protein Gm10036 OS=Mus musculus GN=Gm10036 PE=3 SV=1
Q8R1F1	3.4374E-02	22	11	13	4	27	24	19	33	Niban-like protein 1 OS=Mus musculus GN=Fam129b PE=1 SV=2
Q01853	3.4475E-02	45	26	44	11	120	98	42	89	Transitional endoplasmic reticulum ATPase OS=Mus musculus GN=Vcp PE=1 SV=4
P54761	3.4542E-02	3	0	5	0	4	3	5	3	Ephrin type-B receptor 4 OS=Mus musculus GN=Ephb4 PE=1 SV=2
Q8C8K1	3.4542E-02	3	0	5	0	4	3	5	3	Ephrin type-B receptor 4 OS=Mus musculus GN=Ephb4 PE=1 SV=1
E9PWK7	3.4542E-02	3	0	5	0	4	3	5	3	Ephrin type-B receptor 4 OS=Mus musculus GN=Ephb4 PE=1 SV=1
E9PX48	3.4680E-02	6	2	5	0	8	9	8	9	Dedicator of cytokinesis protein 7 OS=Mus musculus GN=Dock7 PE=1 SV=2
P62196	3.4697E-02	24	30	28	7	18	13	2	11	26S protease regulatory subunit 8 OS=Mus musculus GN=Psmc5 PE=1 SV=1
Q640N1	3.4719E-02	15	11	16	2	59	60	18	44	Adipocyte enhancer-binding protein 1 OS=Mus musculus GN=Aebp1 PE=1 SV=1
H7BX99	3.4770E-02	4	2	3	0	11	16	7	19	Prothrombin OS=Mus musculus GN=F2 PE=1 SV=1
P19221	3.4770E-02	4	2	3	0	11	16	7	19	Prothrombin OS=Mus musculus GN=F2 PE=1 SV=1
Q99JX4	3.4798E-02	7	9	2	3	0	0	0	0	Eukaryotic translation initiation factor 3 subunit M OS=Mus musculus GN=Eif3m PE=1 SV=1
E9PXY1	3.4984E-02	5	8	12	7	0	0	0	4	Cullin-4B OS=Mus musculus GN=Cul4b PE=1 SV=2
A2A432	3.4984E-02	5	8	12	7	0	0	0	4	Cullin-4B OS=Mus musculus GN=Cul4b PE=1 SV=1
Q8VIJ6	3.5440E-02	11	7	12	2	13	15	15	14	Splicing factor, proline- and glutamine-rich OS=Mus musculus GN=Sfpq PE=1 SV=1
Q9CPN8	3.5556E-02	0	0	0	0	13	14	3	6	Insulin-like growth factor 2 mRNA-binding protein 3 OS=Mus musculus GN=Igf2bp3 PE=1 SV=1
Q61171	3.5908E-02	7	2	8	0	32	28	9	28	Peroxiredoxin-2 OS=Mus musculus GN=Prdx2 PE=1 SV=3
P62274	3.6060E-02	6	9	6	3	4	0	0	0	40S ribosomal protein S29 OS=Mus musculus GN=Rps29 PE=3 SV=2
E9PWG6	3.6139E-02	9	8	6	5	0	2	2	2	Protein Ncapg OS=Mus musculus GN=Ncapg PE=1 SV=1
A0A0G2JEJ0	3.6260E-02	0	0	0	0	12	4	2	6	PDZ and LIM domain protein 5 (Fragment) OS=Mus musculus GN=Pdlim5 PE=1 SV=1
Q91WK2	3.6437E-02	6	6	5	2	3	5	0	5	Eukaryotic translation initiation factor 3 subunit H OS=Mus musculus GN=Eif3h PE=1 SV=1
P33434	3.6570E-02	0	0	0	0	4	9	3	4	72 kDa type IV collagenase OS=Mus musculus GN=Mmp2 PE=1 SV=1
A2AKI5	3.6733E-02	4	2	0	3	28	19	20	15	Integrin alpha-V OS=Mus musculus GN=Itgav PE=1 SV=1
P43406	3.6733E-02	4	2	0	3	28	19	20	15	Integrin alpha-V OS=Mus musculus GN=Itgav PE=1 SV=2
Q61768	3.6797E-02	18	21	24	7	16	13	6	10	Kinesin-1 heavy chain OS=Mus musculus GN=Kif5b PE=1 SV=3
Q91VC3	3.6945E-02	53	33	43	9	14	24	12	21	Eukaryotic initiation factor 4A-III OS=Mus musculus GN=Eif4a3 PE=1 SV=3
Q9JM76	3.7036E-02	34	21	25	16	20	8	0	9	Actin-related protein 2/3 complex subunit 3 OS=Mus musculus GN=Arpc3 PE=1 SV=3
E9QN08	3.8124E-02	2	5	5	0	15	7	10	12	Elongation factor 1-delta (Fragment) OS=Mus musculus GN=Eef1d PE=1 SV=1
Q9CR16	3.8303E-02	12	8	8	6	31	16	14	24	Peptidyl-prolyl cis-trans isomerase D OS=Mus musculus GN=Ppid PE=1 SV=3
Q99LC2	3.8367E-02	4	3	4	2	0	0	2	0	Cleavage stimulation factor subunit 1 OS=Mus musculus GN=Cstf1 PE=1 SV=1
P60122	3.8886E-02	50	33	37	18	37	20	21	27	RuvB-like 1 OS=Mus musculus GN=Ruvbl1 PE=1 SV=1
P25206	3.8993E-02	17	13	26	5	11	11	9	5	DNA replication licensing factor MCM3 OS=Mus musculus GN=Mcm3 PE=1 SV=2
Q80T06	3.9441E-02	2	5	5	0	16	7	10	12	Elongation factor 1-delta OS=Mus musculus GN=Eef1d PE=1 SV=1
P57776	3.9441E-02	2	5	5	0	16	7	10	12	Elongation factor 1-delta OS=Mus musculus GN=Eef1d PE=1 SV=3
A0A0R4J1E2	3.9441E-02	2	5	5	0	16	7	10	12	Elongation factor 1-delta OS=Mus musculus GN=Eef1d PE=1 SV=1
P80318	3.9641E-02	161	123	144	68	99	88	82	103	T-complex protein 1 subunit gamma OS=Mus musculus GN=Cct3 PE=1 SV=1
A2A9M4	3.9855E-02	6	2	5	0	8	9	8	10	Dedicator of cytokinesis protein 7 OS=Mus musculus GN=Dock7 PE=1 SV=2
A2A9M5	3.9855E-02	6	2	5	0	8	9	8	10	Dedicator of cytokinesis protein 7 OS=Mus musculus GN=Dock7 PE=1 SV=1
A0A0U1RNK7	3.9855E-02	6	2	5	0	8	9	8	10	Dedicator of cytokinesis protein 7 OS=Mus musculus GN=Dock7 PE=1 SV=1
Q8R1A4	3.9855E-02	6	2	5	0	8	9	8	10	Dedicator of cytokinesis protein 7 OS=Mus musculus GN=Dock7 PE=1 SV=3
Q99LX0	3.9922E-02	2	0	0	0	4	8	7	13	Protein deglycase DJ-1 OS=Mus musculus GN=Park7 PE=1 SV=1
Q9CQ60	4.0113E-02	0	0	0	0	14	9	2	5	6-phosphogluconolactonase OS=Mus musculus GN=Pgls PE=1 SV=1
Q61990	4.0220E-02	24	17	16	4	18	12	8	13	Poly(rC)-binding protein 2 OS=Mus musculus GN=Pcbp2 PE=1 SV=1
O35130	4.0394E-02	3	3	7	3	0	0	0	0	Ribosomal RNA small subunit methyltransferase NEP1 OS=Mus musculus GN=Emg1 PE=1 SV=1
Q8R1B4	4.0790E-02	41	27	38	9	30	26	21	28	Eukaryotic translation initiation factor 3 subunit C OS=Mus musculus GN=Eif3c PE=1 SV=1
Q6PE01	4.0997E-02	20	22	24	11	17	16	10	19	U5 small nuclear ribonucleoprotein 40 kDa protein OS=Mus musculus GN=Snrnp40 PE=1 SV=1
Q9WU78	4.1530E-02	88	65	57	17	107	83	68	95	Programmed cell death 6-interacting protein OS=Mus musculus GN=Pdcd6ip PE=1 SV=3
Q6R0H7	4.2890E-02	30	19	25	7	20	17	10	21	Guanine nucleotide-binding protein G(s) subunit alpha isoforms XLas OS=Mus musculus GN=Gnas PE=1 SV=1
P63094	4.2890E-02	30	19	25	7	20	17	10	21	Guanine nucleotide-binding protein G(s) subunit alpha isoforms short OS=Mus musculus GN=Gnas PE=1 SV=1
G3X8Y3	4.3003E-02	72	61	65	31	43	33	39	40	N-alpha-acetyltransferase 15, NatA auxiliary subunit OS=Mus musculus GN=Naa15 PE=1 SV=1
P57780	4.3193E-02	46	42	31	7	76	53	40	66	Alpha-actinin-4 OS=Mus musculus GN=Actn4 PE=1 SV=1
E9Q715	4.3194E-02	3	3	5	2	2	0	0	0	Putative RNA-binding protein Luc7-like 2 OS=Mus musculus GN=Luc7l2 PE=1 SV=1
F6WHQ7	4.3269E-02	5	0	0	0	6	6	4	7	Glutathione S-transferase Mu 1 (Fragment) OS=Mus musculus GN=Gstm1 PE=1 SV=1
A0A1B0GSX0	4.3670E-02	28	20	20	14	52	36	31	51	L-lactate dehydrogenase OS=Mus musculus GN=Ldha PE=1 SV=1
Q564E2	4.3670E-02	28	20	20	14	52	36	31	51	L-lactate dehydrogenase OS=Mus musculus GN=Ldha PE=1 SV=1
A0A0U1RNK6	4.3734E-02	13	10	9	3	13	7	3	7	S-adenosylmethionine synthase OS=Mus musculus GN=Mat2a PE=1 SV=1
A0A1B0GSE8	4.3916E-02	22	14	20	5	17	15	10	11	40S ribosomal protein S11 (Fragment) OS=Mus musculus GN=Rps11 PE=1 SV=1
A2A813	4.4255E-02	2	0	0	0	4	6	7	12	Protein deglycase DJ-1 OS=Mus musculus GN=Park7 PE=1 SV=1
Q6P4T2	4.4323E-02	60	60	69	12	46	29	21	29	U5 small nuclear ribonucleoprotein 200 kDa helicase OS=Mus musculus GN=Snrnp200 PE=1 SV=1
O08553	4.4363E-02	45	34	36	4	91	81	34	83	Dihydropyrimidinase-related protein 2 OS=Mus musculus GN=Dpysl2 PE=1 SV=2
A2A5N1	4.4372E-02	21	8	17	5	25	18	16	22	14-3-3 protein beta/alpha (Fragment) OS=Mus musculus GN=Ywhab PE=1 SV=1
Q61072	4.4523E-02	0	3	3	0	5	7	12	12	Disintegrin and metalloproteinase domain-containing protein 9 OS=Mus musculus GN=Adam9 PE=1 SV=2
E9Q638	4.4523E-02	0	3	3	0	5	7	12	12	Disintegrin and metalloproteinase domain-containing protein 9 OS=Mus musculus GN=Adam9 PE=1 SV=1
A0A140LHU0	4.4523E-02	0	3	3	0	5	7	12	12	Disintegrin and metalloproteinase domain-containing protein 9 OS=Mus musculus GN=Adam9 PE=1 SV=1
P40124	4.4783E-02	28	20	21	9	38	46	44	50	Adenylyl cyclase-associated protein 1 OS=Mus musculus GN=Cap1 PE=1 SV=4
Q6Q477	4.4867E-02	3	0	4	0	5	4	4	5	Plasma membrane calcium-transporting ATPase 4 OS=Mus musculus GN=Atp2b4 PE=1 SV=1
Q6DFW4	4.4909E-02	16	14	18	2	8	4	4	5	Nucleolar protein 58 OS=Mus musculus GN=Nop58 PE=1 SV=1
Q61024	4.5048E-02	54	48	50	8	82	79	59	100	Asparagine synthetase [glutamine-hydrolyzing] OS=Mus musculus GN=Asns PE=1 SV=3
A2AFK7	4.5424E-02	47	27	37	8	13	22	7	17	Eukaryotic initiation factor 4A-III (Fragment) OS=Mus musculus GN=Eif4a3 PE=1 SV=1
P06151	4.5821E-02	28	20	19	14	51	36	31	51	L-lactate dehydrogenase A chain OS=Mus musculus GN=Ldha PE=1 SV=3
Q8BY71	4.6495E-02	14	10	11	2	3	3	0	6	Histone acetyltransferase type B catalytic subunit OS=Mus musculus GN=Hat1 PE=1 SV=1
A2ATU9	4.6495E-02	14	10	11	2	3	3	0	6	Histone acetyltransferase type B catalytic subunit OS=Mus musculus GN=Hat1 PE=1 SV=1
P32883	4.6516E-02	9	0	7	0	11	7	9	10	GTPase KRas OS=Mus musculus GN=Kras PE=1 SV=1
P14211	4.6848E-02	9	7	10	4	18	13	10	15	Calreticulin OS=Mus musculus GN=Calr PE=1 SV=1
Q8K1K2	4.6992E-02	19	20	16	3	11	8	2	8	26S protease regulatory subunit 8 OS=Mus musculus GN=Psmc5 PE=1 SV=1
A0A1B0GSR9	4.8281E-02	28	20	18	14	51	36	31	51	L-lactate dehydrogenase OS=Mus musculus GN=Ldha PE=1 SV=1
Q7TNC4	4.8377E-02	3	3	6	3	2	0	0	3	Putative RNA-binding protein Luc7-like 2 OS=Mus musculus GN=Luc7l2 PE=1 SV=1

A2AGT5	4.8740E-02	5	3	4	4	0	0	0	2	Cytoskeleton-associated protein 5 OS=Mus musculus GN=Ckap5 PE=1 SV=1
Z4YL78	4.8740E-02	5	3	4	4	0	0	0	2	Cytoskeleton-associated protein 5 OS=Mus musculus GN=Ckap5 PE=1 SV=1
K3W4R5	4.8740E-02	5	3	4	4	0	0	0	2	Cytoskeleton-associated protein 5 OS=Mus musculus GN=Ckap5 PE=1 SV=1
A0A0R4J0K2	4.8740E-02	5	3	4	4	0	0	0	2	Cytoskeleton-associated protein 5 OS=Mus musculus GN=Ckap5 PE=1 SV=1
P62281	4.9547E-02	29	21	28	5	22	18	12	15	40S ribosomal protein S11 OS=Mus musculus GN=Rps11 PE=1 SV=3
Q9WUM3	5.0923E-02	13	6	10	0	16	27	18	24	Coronin-1B OS=Mus musculus GN=Coro1b PE=1 SV=1
Q9Z2U0	5.0970E-02	7	0	5	0	10	7	6	10	Proteasome subunit alpha type-7 OS=Mus musculus GN=Psma7 PE=1 SV=1
Q9QZD9	5.1734E-02	41	35	38	5	16	13	8	16	Eukaryotic translation initiation factor 3 subunit I OS=Mus musculus GN=Eif3i PE=1 SV=1
Q8CFI7	5.1872E-02	30	25	27	4	12	6	2	12	DNA-directed RNA polymerase II subunit RPB2 OS=Mus musculus GN=Polr2b PE=1 SV=2
Q61398	5.2622E-02	90	47	44	19	43	34	28	41	Procollagen C-endopeptidase enhancer 1 OS=Mus musculus GN=Pcolce PE=1 SV=2
Q9WV32	5.3215E-02	42	33	38	7	34	19	12	20	Actin-related protein 2/3 complex subunit 1B OS=Mus musculus GN=Arpc1b PE=1 SV=4
Q9WUA3	5.3466E-02	17	18	22	3	6	5	0	7	ATP-dependent 6-phosphofructokinase, platelet type OS=Mus musculus GN=Pfkp PE=1 SV=1
Q8C605	5.3466E-02	17	18	22	3	6	5	0	7	ATP-dependent 6-phosphofructokinase OS=Mus musculus GN=Pfkp PE=1 SV=1
P47857	5.3649E-02	17	10	18	3	6	10	5	4	ATP-dependent 6-phosphofructokinase, muscle type OS=Mus musculus GN=Pfkm PE=1 SV=3
P17742	5.3917E-02	52	27	25	0	77	59	30	62	Peptidyl-prolyl cis-trans isomerase A OS=Mus musculus GN=Ppia PE=1 SV=2
Q922I7	5.4312E-02	8	8	5	0	13	10	9	9	MCG13402, isoform CRA_c OS=Mus musculus GN=Ptbp1 PE=1 SV=1
Q8BGJ5	5.4782E-02	8	8	5	0	13	10	8	9	MCG13402, isoform CRA_a OS=Mus musculus GN=Ptbp1 PE=1 SV=1
Q80UM3	5.5019E-02	69	61	61	31	41	31	38	37	N-alpha-acetyltransferase 15, NatA auxiliary subunit OS=Mus musculus GN=Naa15 PE=1 SV=1
F7CAZ6	5.5925E-02	21	20	25	9	40	45	24	37	F-actin-capping protein subunit beta (Fragment) OS=Mus musculus GN=Capzb PE=1 SV=1
Q9D0I9	5.6114E-02	50	39	37	10	33	27	19	34	Arginine--tRNA ligase, cytoplasmic OS=Mus musculus GN=Rars PE=1 SV=2
G3UYZ1	5.6359E-02	0	5	0	0	14	6	11	12	Immunoglobulin superfamily member 8 OS=Mus musculus GN=Igsf8 PE=1 SV=1
Q8R366	5.6359E-02	0	5	0	0	14	6	11	12	Immunoglobulin superfamily member 8 OS=Mus musculus GN=Igsf8 PE=1 SV=2
A0A0R4J117	5.6359E-02	0	5	0	0	14	6	11	12	Immunoglobulin superfamily member 8 OS=Mus musculus GN=Igsf8 PE=1 SV=1
O55023	5.6507E-02	4	3	5	0	13	8	4	7	Inositol monophosphatase 1 OS=Mus musculus GN=Impa1 PE=1 SV=1
Q9CQ65	5.7506E-02	26	17	24	3	43	37	19	33	S-methyl-5'-thioadenosine phosphorylase OS=Mus musculus GN=Mtap PE=1 SV=1
Q8C253	5.7683E-02	26	13	17	3	32	41	21	33	Galectin OS=Mus musculus GN=Lgals3 PE=1 SV=1
O08604	5.8169E-02	0	0	0	0	7	5	4	0	Retinoic acid early-inducible protein 1-gamma OS=Mus musculus GN=Raet1c PE=1 SV=1
P70336	5.8329E-02	9	7	7	0	0	0	0	0	Rho-associated protein kinase 2 OS=Mus musculus GN=Rock2 PE=1 SV=1
P19157	5.8350E-02	20	19	16	8	19	12	0	19	Glutathione S-transferase P 1 OS=Mus musculus GN=Gstp1 PE=1 SV=2
Q91Z25	5.8410E-02	42	33	38	7	35	18	12	20	Actin-related protein 2/3 complex subunit 1B OS=Mus musculus GN=Arpc1b PE=1 SV=1
Q9ERU9	5.8426E-02	6	4	5	0	0	0	0	0	E3 SUMO-protein ligase RanBP2 OS=Mus musculus GN=Ranbp2 PE=1 SV=2
Q91V35	5.8492E-02	0	0	0	0	3	0	2	3	Receptor-type tyrosine-protein phosphatase OS=Mus musculus GN=Ptpra PE=1 SV=1
F6SDD7	5.8501E-02	4	3	3	0	0	0	0	0	DNA topoisomerase (Fragment) OS=Mus musculus GN=Top3b PE=1 SV=1
O88792	5.8501E-02	4	3	3	0	0	0	0	0	Junctional adhesion molecule A OS=Mus musculus GN=F11r PE=1 SV=2
A2RTH5	5.8501E-02	4	3	3	0	0	0	0	0	Leucine carboxyl methyltransferase 1 OS=Mus musculus GN=Lcmt1 PE=1 SV=1
A0A0U1RNF2	5.8501E-02	4	3	3	0	0	0	0	0	Protein Lcmt1 OS=Mus musculus GN=Lcmt1 PE=1 SV=1
E9PV04	5.8565E-02	50	0	43	9	14	0	12	0	Protein Gm8994 OS=Mus musculus GN=Gm8994 PE=3 SV=2
P21278	5.8728E-02	8	9	11	2	4	5	0	4	Guanine nucleotide-binding protein subunit alpha-11 OS=Mus musculus GN=Gna11 PE=1 SV=1
Q9D8U8	5.8802E-02	0	0	0	0	4	3	0	3	Sorting nexin-5 OS=Mus musculus GN=Snx5 PE=1 SV=1
Q3TT92	5.8920E-02	0	0	0	0	39	29	0	29	Dihydropyrimidinase-related protein 3 OS=Mus musculus GN=Dpysl3 PE=1 SV=1
D3Z2H9	5.8939E-02	27	26	25	13	57	53	39	45	Protein Tpm3-rs7 OS=Mus musculus GN=Tpm3-rs7 PE=3 SV=1
Q76MZ3	5.8950E-02	80	46	74	25	69	48	34	59	Serine/threonine-protein phosphatase 2A 65 kDa regulatory subunit A alpha isoform OS=Mus musculus GN=Ppp2r1a PE=1 SV=3
A0A0J9YUZ4	5.8986E-02	8	8	8	0	9	15	12	11	High mobility group protein B1 (Fragment) OS=Mus musculus GN=Hmgb1 PE=1 SV=1
P63158	5.8986E-02	8	8	8	0	9	15	12	11	High mobility group protein B1 OS=Mus musculus GN=Hmgb1 PE=1 SV=2
Q6PAV2	5.9002E-02	7	5	5	0	0	0	0	0	Probable E3 ubiquitin-protein ligase HERC4 OS=Mus musculus GN=Herc4 PE=1 SV=2
Q9DC48	5.9003E-02	4	5	4	0	0	2	0	0	Pre-mRNA-processing factor 17 OS=Mus musculus GN=Cdc40 PE=2 SV=1
P17563	5.9096E-02	15	13	15	0	0	0	0	0	Selenium-binding protein 1 OS=Mus musculus GN=Selenbp1 PE=1 SV=2
F8WID5	5.9205E-02	27	28	0	0	49	37	32	33	Tropomyosin alpha-1 chain OS=Mus musculus GN=Tpm1 PE=1 SV=1
P11440	5.9279E-02	78	49	57	5	26	19	15	17	Cyclin-dependent kinase 1 OS=Mus musculus GN=Cdk1 PE=1 SV=3
Q60716	5.9372E-02	0	0	0	0	4	3	2	0	Prolyl 4-hydroxylase subunit alpha-2 OS=Mus musculus GN=P4ha2 PE=1 SV=1
Q5SX75	5.9372E-02	0	0	0	0	4	3	2	0	Procollagen-proline, 2-oxoglutarate 4-dioxygenase (Proline 4-hydroxylase), alpha II polypeptide, isoform CRA_f OS=Mus musculus GN=P4ha2 PE=1 SV=1
Q61391	5.9404E-02	0	0	0	0	4	3	0	4	Neprilysin OS=Mus musculus GN=Mme PE=1 SV=3
A0A0G2JH04	5.9457E-02	17	9	18	9	0	10	0	13	Eukaryotic translation initiation factor 4E OS=Mus musculus GN=Eif4e PE=1 SV=1
Q921D0	5.9505E-02	4	3	4	0	0	0	0	0	Annexin OS=Mus musculus GN=Anxa8 PE=1 SV=1
Q9Z321	5.9505E-02	4	3	4	0	0	0	0	0	DNA topoisomerase 3-beta-1 OS=Mus musculus GN=Top3b PE=1 SV=1
F6X8L5	5.9606E-02	0	0	0	0	7	5	0	5	6-phosphogluconolactonase (Fragment) OS=Mus musculus GN=Pgls PE=1 SV=1
A2AW41	5.9822E-02	5	0	5	0	10	5	4	7	Protein Hnrnpr (Fragment) OS=Mus musculus GN=Hnrnpr PE=1 SV=1
Q5SWU9	5.9992E-02	29	16	28	6	10	14	10	16	Acetyl-CoA carboxylase 1 OS=Mus musculus GN=Acaca PE=1 SV=1

Table S3

Click here to access/download;Table;Table S3.pdf

Table S3. Shown here are 79 protein fractions expressed exclusively in isolated EVs from WT (ciliated cells) after inducing the release of ciliary vesicle by fluid-shear flow.

		Spectral count for ciliated EV (WT)				Spectral count for non-ciliated EV (KO)				
**Accession**	**T-test p-value**	**Sample1**	**Sample2**	**Sample3**	**Sample4**	**Sample1**	**Sample2**	**Sample3**	**Sample4**	**Description**
Q05DV1	2.042E-04	18.99	20.01	23.4	20.67	0	0	0	0	NADPH--cytochrome P450 reductase OS=Mus musculus GN=Por PE=1 SV=1
A0A1B0GR11	3.115E-04	18.99	24.22	20.59	20.67	0	0	0	0	Transaldolase OS=Mus musculus GN=Taldo1 PE=1 SV=1
Q93092	3.115E-04	18.99	24.22	20.59	20.67	0	0	0	0	Transaldolase OS=Mus musculus GN=Taldo1 PE=1 SV=2
Q9CYN9	4.182E-04	13.45	15.8	16.85	17.71	0	0	0	0	Renin receptor OS=Mus musculus GN=Atp6ap2 PE=1 SV=2
Q80W65	7.985E-04	11.87	10.53	12.17	14.76	0	0	0	0	Proprotein convertase subtilisin/kexin type 9 OS=Mus musculus GN=Pcsk9 PE=1 SV=2
P37040	9.213E-04	18.99	20.01	26.2	20.67	0	0	0	0	NADPH--cytochrome P450 reductase OS=Mus musculus GN=Por PE=1 SV=2
Q01320	1.024E-03	49.86	51.6	45.86	64.95	0	0	0	0	DNA topoisomerase 2-alpha OS=Mus musculus GN=Top2a PE=1 SV=2
Q99P88	1.047E-03	6.332	7.371	6.551	8.857	0	0	0	0	Nuclear pore complex protein Nup155 OS=Mus musculus GN=Nup155 PE=1 SV=1
Q9Z2V5	1.225E-03	3.957	5.265	5.615	5.904	0	0	0	0	Histone deacetylase 6 OS=Mus musculus GN=Hdac6 PE=1 SV=3
A0A1B0GX25	1.225E-03	3.957	5.265	5.615	5.904	0	0	0	0	Histone deacetylase 6 (Fragment) OS=Mus musculus GN=Hdac6 PE=1 SV=1
Q810B6	1.268E-03	3.957	5.265	4.679	5.904	0	0	0	0	Rabankyrin-5 OS=Mus musculus GN=Ankfy1 PE=1 SV=2
Q9Z1T1	1.433E-03	7.123	7.371	10.29	8.857	0	0	0	0	AP-3 complex subunit beta-1 OS=Mus musculus GN=Ap3b1 PE=1 SV=2
P19001	1.570E-03	12.66	10.53	15.91	11.81	0	0	0	0	Keratin, type I cytoskeletal 19 OS=Mus musculus GN=Krt19 PE=1 SV=1
F8VPK5	1.956E-03	8.706	9.477	7.487	11.81	0	0	0	0	Rho-associated protein kinase OS=Mus musculus GN=Rock2 PE=1 SV=1
Q61001	2.110E-03	44.32	34.75	36.5	53.14	0	0	0	0	Laminin subunit alpha-5 OS=Mus musculus GN=Lama5 PE=1 SV=4
O35566	2.948E-03	34.82	46.33	57.09	38.38	0	0	0	0	CD151 antigen OS=Mus musculus GN=Cd151 PE=1 SV=2
A0A1B0GRG3	3.122E-03	32.45	44.23	55.22	38.38	0	0	0	0	CD151 antigen (Fragment) OS=Mus musculus GN=Cd151 PE=1 SV=1
Q8R480	3.317E-03	5.54	4.212	7.487	5.904	0	0	0	0	Nuclear pore complex protein Nup85 OS=Mus musculus GN=Nup85 PE=1 SV=1
A2AW05	3.781E-03	4.749	3.159	4.679	5.904	0	0	0	0	FACT complex subunit SSRP1 (Fragment) OS=Mus musculus GN=Ssrp1 PE=1 SV=1
Q08943	3.781E-03	4.749	3.159	4.679	5.904	0	0	0	0	FACT complex subunit SSRP1 OS=Mus musculus GN=Ssrp1 PE=1 SV=2
P58854	4.033E-03	3.166	5.265	5.615	5.904	0	0	0	0	Gamma-tubulin complex component 3 OS=Mus musculus GN=Tubgcp3 PE=1 SV=2
Q6P5D8	4.176E-03	13.45	9.477	15.91	17.71	0	0	0	0	Structural maintenance of chromosomes flexible hinge domain-containing protein 1 OS=Mus musculus GN=Smchd1 PE=1 SV=2
Q9JHK4	4.442E-03	7.915	8.424	6.551	11.81	0	0	0	0	Geranylgeranyl transferase type-2 subunit alpha OS=Mus musculus GN=Rabggta PE=1 SV=1
A0A0R4J233	5.084E-03	6.332	8.424	12.17	8.857	0	0	0	0	Septin-10 OS=Mus musculus GN=Sept10 PE=1 SV=1
Q8C650	5.084E-03	6.332	8.424	12.17	8.857	0	0	0	0	Septin-10 OS=Mus musculus GN=Sept10 PE=1 SV=1
Q9WUU7	5.388E-03	8.706	6.318	7.487	11.81	0	0	0	0	Cathepsin Z OS=Mus musculus GN=Ctsz PE=1 SV=1
Q9WTZ2	5.895E-03	3.957	3.159	3.743	5.904	0	0	0	0	Membrane-bound transcription factor site-1 protease OS=Mus musculus GN=Mbtps1 PE=1 SV=1
E9Q3L1	6.679E-03	4.749	9.477	7.487	5.904	0	0	0	0	Unconventional myosin-VI OS=Mus musculus GN=Myo6 PE=1 SV=1
V9GXM4	6.679E-03	4.749	9.477	7.487	5.904	0	0	0	0	Unconventional myosin-VI OS=Mus musculus GN=Myo6 PE=1 SV=1
V9GX76	6.679E-03	4.749	9.477	7.487	5.904	0	0	0	0	Unconventional myosin-VI OS=Mus musculus GN=Myo6 PE=1 SV=1
E9PVU0	6.679E-03	4.749	9.477	7.487	5.904	0	0	0	0	Unconventional myosin-VI OS=Mus musculus GN=Myo6 PE=1 SV=1
E9Q175	6.679E-03	4.749	9.477	7.487	5.904	0	0	0	0	Unconventional myosin-VI OS=Mus musculus GN=Myo6 PE=1 SV=1
E9Q174	6.679E-03	4.749	9.477	7.487	5.904	0	0	0	0	Unconventional myosin-VI OS=Mus musculus GN=Myo6 PE=1 SV=1
P98063	7.098E-03	42.74	57.92	48.66	82.66	0	0	0	0	Bone morphogenetic protein 1 OS=Mus musculus GN=Bmp1 PE=1 SV=2
Q920Q8	7.214E-03	5.54	4.212	2.808	5.904	0	0	0	0	Influenza virus NS1A-binding protein homolog OS=Mus musculus GN=Ivns1abp PE=1 SV=2
P43346	8.144E-03	12.66	12.64	5.615	11.81	0	0	0	0	Deoxycytidine kinase OS=Mus musculus GN=Dck PE=1 SV=1
Q6P5B0	9.106E-03	6.332	11.58	7.487	5.904	0	0	0	0	RRP12-like protein OS=Mus musculus GN=Rrp12 PE=1 SV=1
P11370	9.215E-03	10.29	10.53	5.615	5.904	0	0	0	0	Retrovirus-related Env polyprotein from Fv-4 locus OS=Mus musculus GN=Fv4 PE=1 SV=2
Q8VC28	9.903E-03	5.54	8.424	6.551	11.81	0	0	0	0	Aldo-keto reductase family 1 member C13 OS=Mus musculus GN=Akr1c13 PE=1 SV=2
G3X934	9.949E-03	10.29	15.8	14.97	23.62	0	0	0	0	MCG115964 OS=Mus musculus GN=Wdr70 PE=1 SV=1
Q571I9	1.063E-02	4.749	4.212	7.487	8.857	0	0	0	0	Aldehyde dehydrogenase family 16 member A1 OS=Mus musculus GN=Aldh16a1 PE=1 SV=2
A0A1B0GSU0	1.063E-02	4.749	4.212	7.487	8.857	0	0	0	0	Aldehyde dehydrogenase family 16 member A1 OS=Mus musculus GN=Aldh16a1 PE=1 SV=1
D3Z0B9	1.063E-02	4.749	4.212	7.487	8.857	0	0	0	0	Aldehyde dehydrogenase family 16 member A1 OS=Mus musculus GN=Aldh16a1 PE=1 SV=3
P43276	1.065E-02	8.706	8.424	16.85	14.76	0	0	0	0	Histone H1.5 OS=Mus musculus GN=Hist1h1b PE=1 SV=2
E9PXY8	1.073E-02	11.87	24.22	13.1	14.76	0	0	0	0	Ubiquitin carboxyl-terminal hydrolase 7 OS=Mus musculus GN=Usp7 PE=1 SV=1
F8VPX1	1.073E-02	11.87	24.22	13.1	14.76	0	0	0	0	Ubiquitin carboxyl-terminal hydrolase 7 OS=Mus musculus GN=Usp7 PE=1 SV=1
Q6A4J8	1.073E-02	11.87	24.22	13.1	14.76	0	0	0	0	Ubiquitin carboxyl-terminal hydrolase 7 OS=Mus musculus GN=Usp7 PE=1 SV=1
Q3TWF6	1.082E-02	10.29	15.8	14.04	23.62	0	0	0	0	WD repeat-containing protein 70 OS=Mus musculus GN=Wdr70 PE=1 SV=1
Q6ZWQ0	1.263E-02	4.749	7.371	7.487	11.81	0	0	0	0	Nesprin-2 OS=Mus musculus GN=Syne2 PE=1 SV=2
E9QP46	1.263E-02	4.749	7.371	7.487	11.81	0	0	0	0	Nesprin-2 OS=Mus musculus GN=Syne2 PE=1 SV=1
Q9CXF4	1.809E-02	8.706	8.424	2.808	5.904	0	0	0	0	TBC1 domain family member 15 OS=Mus musculus GN=Tbc1d15 PE=1 SV=1
O88796	1.816E-02	3.957	4.212	4.679	8.857	0	0	0	0	Ribonuclease P protein subunit p30 OS=Mus musculus GN=Rpp30 PE=1 SV=1
Q923B1	1.877E-02	5.54	10.53	3.743	8.857	0	0	0	0	Lariat debranching enzyme OS=Mus musculus GN=Dbr1 PE=1 SV=2
Q8C7V3	2.118E-02	3.166	4.212	1.872	5.904	0	0	0	0	U3 small nucleolar RNA-associated protein 15 homolog OS=Mus musculus GN=Utp15 PE=1 SV=1
Q6PAR5	2.130E-02	6.332	9.477	3.743	11.81	0	0	0	0	GTPase-activating protein and VPS9 domain-containing protein 1 OS=Mus musculus GN=Gapvd1 PE=1 SV=2
Q8JZK9	2.253E-02	18.99	14.74	22.46	5.904	0	0	0	0	Hydroxymethylglutaryl-CoA synthase, cytoplasmic OS=Mus musculus GN=Hmgcs1 PE=1 SV=1
A6PWY4	2.359E-02	9.497	11.58	17.78	26.57	0	0	0	0	WD repeat-containing protein 76 OS=Mus musculus GN=Wdr76 PE=1 SV=1
P10404	2.363E-02	15.83	9.477	12.17	26.57	0	0	0	0	MLV-related proviral Env polyprotein OS=Mus musculus PE=1 SV=3
Q549C9	2.386E-02	19.79	25.27	18.72	5.904	0	0	0	0	Cellular tumor antigen p53 OS=Mus musculus GN=Trp53 PE=1 SV=1
P02340	2.386E-02	19.79	25.27	18.72	5.904	0	0	0	0	Cellular tumor antigen p53 OS=Mus musculus GN=Tp53 PE=1 SV=3
Q80ZA1	2.386E-02	19.79	25.27	18.72	5.904	0	0	0	0	Cellular tumor antigen p53 OS=Mus musculus GN=Trp53 PE=1 SV=1
I7HIK9	2.386E-02	19.79	25.27	18.72	5.904	0	0	0	0	Cellular tumor antigen p53 OS=Mus musculus GN=Trp53 PE=1 SV=1
Q9D1M4	2.388E-02	7.123	7.371	5.615	14.76	0	0	0	0	Eukaryotic translation elongation factor 1 epsilon-1 OS=Mus musculus GN=Eef1e1 PE=1 SV=1
Q91W50	2.427E-02	5.54	4.212	6.551	11.81	0	0	0	0	Cold shock domain-containing protein E1 OS=Mus musculus GN=Csde1 PE=1 SW=1
Q3TY56	2.835E-02	3.957	5.265	2.808	8.857	0	0	0	0	Eukaryotic translation initiation factor 3 subunit K OS=Mus musculus GN=Eif3k PE=1 SV=1
Q4VA53	3.395E-02	14.25	4.212	13.1	5.904	0	0	0	0	Sister chromatid cohesion protein PDS5 homolog B OS=Mus musculus GN=Pds5b PE=1 SV=1
F8WHU5	3.395E-02	14.25	4.212	13.1	5.904	0	0	0	0	Sister chromatid cohesion protein PDS5 homolog B OS=Mus musculus GN=Pds5b PE=1 SV=1
Q99JX4	3.480E-02	5.54	9.477	1.872	8.857	0	0	0	0	Eukaryotic translation initiation factor 3 subunit M OS=Mus musculus GN=Eif3m PE=1 SV=1
O35130	4.039E-02	2.374	3.159	6.551	8.857	0	0	0	0	Ribosomal RNA small subunit methyltransferase NEP1 OS=Mus musculus GN=Emg1 PE=1 SV=1
P70336	5.833E-02	7.123	7.371	6.551	0	0	0	0	0	Rho-associated protein kinase 2 OS=Mus musculus GN=Rock2 PE=1 SV=1
Q9ERU9	5.843E-02	4.749	4.212	4.679	0	0	0	0	0	E3 SUMO-protein ligase RanBP2 OS=Mus musculus GN=Ranbp2 PE=1 SV=2
F6SDD7	5.850E-02	3.166	3.159	2.808	0	0	0	0	0	DNA topoisomerase (Fragment) OS=Mus musculus GN=Top3b PE=1 SV=1
O88792	5.850E-02	3.166	3.159	2.808	0	0	0	0	0	Junctional adhesion molecule A OS=Mus musculus GN=F11r PE=1 SV=2
A2RTH5	5.850E-02	3.166	3.159	2.808	0	0	0	0	0	Leucine carboxyl methyltransferase 1 OS=Mus musculus GN=Lcmt1 PE=1 SV=1
A0A0U1RNF2	5.850E-02	3.166	3.159	2.808	0	0	0	0	0	Protein Lcmt1 OS=Mus musculus GN=Lcmt1 PE=1 SV=1
Q6PAV2	5.900E-02	5.54	5.265	4.679	0	0	0	0	0	Probable E3 ubiquitin-protein ligase HERC4 OS=Mus musculus GN=Herc4 PE=1 SV=2
P17563	5.910E-02	11.87	13.69	14.04	0	0	0	0	0	Selenium-binding protein 1 OS=Mus musculus GN=Selenbp1 PE=1 SV=2
Q921D0	5.950E-02	3.166	3.159	3.743	0	0	0	0	0	Annexin OS=Mus musculus GN=Anxa8 PE=1 SV=1
Q9Z321	5.950E-02	3.166	3.159	3.743	0	0	0	0	0	DNA topoisomerase 3-beta-1 OS=Mus musculus GN=Top3b PE=1 SV=1

Table S4

Click here to access/download;Table;Table S4.pdf

Table S4. Shown here are 145 protein fractions expressed exclusively in isolated EVs from KO (non-ciliated cells) after inducing the release of ciliary vesicle by fluid-shear flow.

Accession	T-test p-value	Spectral count for ciliated EV (WT)				Spectral count for non-ciliated EV (KO)				Description
		Sample1	Sample2	Sample3	Sample4	Sample1	Sample2	Sample3	Sample4	
Q61490	1.944E-05	0	0	0	0	21.69	22.64	21.01	23.02	CD166 antigen OS=Mus musculus GN=Alcam PE=1 SV=3
E9Q3Q6	1.944E-05	0	0	0	0	21.69	22.64	21.01	23.02	CD166 antigen OS=Mus musculus GN=Alcam PE=1 SV=1
E9Q4G8	1.944E-05	0	0	0	0	21.69	22.64	21.01	23.02	CD166 antigen OS=Mus musculus GN=Alcam PE=1 SV=1
Q5SUW3	7.117E-05	0	0	0	0	4.487	4.718	4.943	4.262	Growth factor receptor-bound protein 10 OS=Mus musculus GN=Grb10 PE=1 SV=1
Q60760	7.117E-05	0	0	0	0	4.487	4.718	4.943	4.262	Growth factor receptor-bound protein 10 OS=Mus musculus GN=Grb10 PE=1 SV=2
P26618	7.382E-05	0	0	0	0	9.721	9.435	8.65	8.524	Platelet-derived growth factor receptor alpha OS=Mus musculus GN=Pdgfra PE=1 SV=3
O35295	9.888E-05	0	0	0	0	5.234	5.661	6.178	5.967	Transcriptional activator protein Pur-beta OS=Mus musculus GN=Purb PE=1 SV=3
O35474	1.057E-04	0	0	0	0	11.22	10.38	12.36	11.08	EGF-like repeat and discoidin I-like domain-containing protein 3 OS=Mus musculus GN=Edil3 PE=1 SV=2
Q8C4U8	1.057E-04	0	0	0	0	11.22	10.38	12.36	11.08	EGF-like repeat and discoidin I-like domain-containing protein 3 OS=Mus musculus GN=Edil3 PE=1 SV=1
Q80YX1	1.250E-04	0	0	0	0	53.09	58.5	60.55	63.93	Tenascin OS=Mus musculus GN=Tnc PE=1 SV=1
Q04857	2.077E-04	0	0	0	0	17.2	17.93	17.3	14.49	Collagen alpha-1(VI) chain OS=Mus musculus GN=Col6a1 PE=1 SV=1
Q6P1J1	2.575E-04	0	0	0	0	14.21	16.98	14.83	13.64	Crmp1 protein OS=Mus musculus GN=Crmp1 PE=1 SV=1
P51655	3.923E-04	0	0	0	0	26.17	20.76	24.71	21.31	Glypican-4 OS=Mus musculus GN=Gpc4 PE=1 SV=2
D3Z1J5	5.086E-04	0	0	0	0	2.991	2.831	3.707	3.41	Palladin (Fragment) OS=Mus musculus GN=Palld PE=1 SV=1
P62897	5.461E-04	0	0	0	0	12.71	12.27	9.885	10.23	Cytochrome c, somatic OS=Mus musculus GN=Cycs PE=1 SV=2
P28653	5.741E-04	0	0	0	0	26.17	19.81	23.48	26.43	Biglycan OS=Mus musculus GN=Bgn PE=1 SV=1
Q9WTK5	6.379E-04	0	0	0	0	2.991	2.831	2.471	3.41	Nuclear factor NF-kappa-B p100 subunit OS=Mus musculus GN=Nfkb2 PE=1 SV=1
Q62009	6.444E-04	0	0	0	0	27.67	22.64	21.01	21.31	Periostin OS=Mus musculus GN=Postn PE=1 SV=2
P97927	6.548E-04	0	0	0	0	25.42	27.36	21.01	28.98	Laminin subunit alpha-4 OS=Mus musculus GN=Lama4 PE=1 SV=2
E9QA16	7.499E-04	0	0	0	0	21.69	22.64	27.19	19.61	Protein Cald1 OS=Mus musculus GN=Cald1 PE=1 SV=1
Q8VCQ8	8.082E-04	0	0	0	0	21.69	21.7	27.19	19.61	Caldesmon 1 OS=Mus musculus GN=Cald1 PE=1 SV=1
A0A0G2JGD2	8.449E-04	0	0	0	0	3.739	4.718	3.707	3.41	Protein S100-A4 (Fragment) OS=Mus musculus GN=S100a4 PE=1 SV=1
P07091	8.449E-04	0	0	0	0	3.739	4.718	3.707	3.41	Protein S100-A4 OS=Mus musculus GN=S100a4 PE=1 SV=1
P37889	9.486E-04	0	0	0	0	64.31	57.55	44.48	52.85	Fibulin-2 OS=Mus musculus GN=Fbln2 PE=1 SV=2
A0A0J9YUE9	1.172E-03	0	0	0	0	7.478	5.661	7.414	8.524	Dynamin-1 OS=Mus musculus GN=Dnm1 PE=1 SV=1
P54728	1.177E-03	0	0	0	0	5.982	6.605	8.65	6.819	UV excision repair protein RAD23 homolog B OS=Mus musculus GN=Rad23b PE=1 SV=2
G3XA35	1.387E-03	0	0	0	0	8.973	8.492	7.414	5.967	MCG116562, isoform CRA_a OS=Mus musculus GN=Vcan PE=1 SV=1
E9PYH0	1.387E-03	0	0	0	0	8.973	8.492	7.414	5.967	Versican core protein OS=Mus musculus GN=Vcan PE=1 SV=1
P10518	1.406E-03	0	0	0	0	5.982	8.492	8.65	6.819	Delta-aminolevulinic acid dehydratase OS=Mus musculus GN=Alad PE=1 SV=1
Q6P069	1.430E-03	0	0	0	0	8.973	10.38	9.885	6.819	Sorcin OS=Mus musculus GN=Sri PE=1 SV=1
P18872	1.481E-03	0	0	0	0	19.44	20.76	16.06	24.72	Guanine nucleotide-binding protein G(o) subunit alpha OS=Mus musculus GN=Gnao1 PE=1 SV=3
Q8CG14	1.604E-03	0	0	0	0	8.973	11.32	7.414	8.524	Complement C1s-A subcomponent OS=Mus musculus GN=C1sa PE=2 SV=2
E9Q6C2	1.604E-03	0	0	0	0	8.973	11.32	7.414	8.524	Protein C1s1 OS=Mus musculus GN=C1s1 PE=1 SV=1
D3Z2M7	1.615E-03	0	0	0	0	19.44	17.93	13.59	21.31	Guanine nucleotide-binding protein G(o) subunit alpha (Fragment) OS=Mus musculus GN=Gnao1 PE=1 SV=1
O88322	1.713E-03	0	0	0	0	68.79	62.27	43.25	60.52	Nidogen-2 OS=Mus musculus GN=Nid2 PE=1 SV=2
O70326	1.788E-03	0	0	0	0	3.739	2.831	3.707	2.557	Gremlin-1 OS=Mus musculus GN=Grem1 PE=2 SV=1
Q62523	2.085E-03	0	0	0	0	4.487	4.718	3.707	5.967	Zyxin OS=Mus musculus GN=Zyx PE=1 SV=2
Q7TQE2	2.085E-03	0	0	0	0	4.487	4.718	3.707	5.967	Zyx protein OS=Mus musculus GN=Zyx PE=1 SV=1
Q01149	2.120E-03	0	0	0	0	31.41	36.8	30.89	22.16	Collagen alpha-2(I) chain OS=Mus musculus GN=Col1a2 PE=1 SV=2
H3BL26	2.249E-03	0	0	0	0	4.487	4.718	4.943	6.819	Tetraspanin-6 (Fragment) OS=Mus musculus GN=Tspan6 PE=1 SV=8
O70401	2.249E-03	0	0	0	0	4.487	4.718	4.943	6.819	Tetraspanin-6 OS=Mus musculus GN=Tspan6 PE=1 SV=1
Q99L96	2.249E-03	0	0	0	0	4.487	4.718	4.943	6.819	Tetraspanin OS=Mus musculus GN=Tspan6 PE=1 SV=1
P28654	2.368E-03	0	0	0	0	57.58	95.29	88.97	77.57	Decorin OS=Mus musculus GN=Dcn PE=1 SV=1
Q62000	2.405E-03	0	0	0	0	10.47	8.492	6.178	8.524	Mimecan OS=Mus musculus GN=Ogn PE=1 SV=1
Q61553	2.754E-03	0	0	0	0	63.56	101	110	94.62	Fascin OS=Mus musculus GN=Fscn1 PE=1 SV=4
G3UXG7	2.887E-03	0	0	0	0	3.739	4.718	6.178	4.262	Casein kinase II subunit beta OS=Mus musculus GN=Csnk2b PE=1 SV=1
P67871	2.887E-03	0	0	0	0	3.739	4.718	6.178	4.262	Casein kinase II subunit beta OS=Mus musculus GN=Csnk2b PE=1 SV=1
D3Z4A4	3.269E-03	0	0	0	0	20.19	16.98	11.12	17.05	Peroxiredoxin-2 (Fragment) OS=Mus musculus GN=Prdx2 PE=1 SV=8
D3YX76	3.340E-03	0	0	0	0	2.991	4.718	3.707	5.115	Glutathione S-transferase OS=Mus musculus GN=Gstm2 PE=1 SV=1
P15626	3.340E-03	0	0	0	0	2.991	4.718	3.707	5.115	Glutathione S-transferase Mu 2 OS=Mus musculus GN=Gstm2 PE=1 SV=2
P16125	3.458E-03	0	0	0	0	8.225	12.27	14.83	11.08	L-lactate dehydrogenase B chain OS=Mus musculus GN=Ldhb PE=1 SV=2
A0A087WS16	3.504E-03	0	0	0	0	18.69	16.98	11.12	12.79	Protein Col6a3 OS=Mus musculus GN=Col6a3 PE=1 SV=1
Q9DCC4	3.573E-03	0	0	0	0	3.739	2.831	4.943	3.41	Pyrroline-5-carboxylate reductase 3 OS=Mus musculus GN=Pycrl PE=1 SV=2
Q5SS83	3.605E-03	0	0	0	0	5.982	3.774	6.178	4.262	Flotillin 2, isoform CRA_a OS=Mus musculus GN=Flot2 PE=1 SV=1
Q60634	3.605E-03	0	0	0	0	5.982	3.774	6.178	4.262	Flotillin-2 OS=Mus musculus GN=Flot2 PE=1 SV=2
A0A067XG53	3.769E-03	0	0	0	0	5.982	7.548	7.414	4.262	Peripheral plasma membrane protein CASK (Fragment) OS=Mus musculus GN=Cask PE=1 SV=1
O70589	3.769E-03	0	0	0	0	5.982	7.548	7.414	4.262	Peripheral plasma membrane protein CASK OS=Mus musculus GN=Cask PE=1 SV=2
P11152	4.018E-03	0	0	0	0	14.21	7.548	12.36	11.08	Lipoprotein lipase OS=Mus musculus GN=Lpl PE=1 SV=3
B1B1E2	4.292E-03	0	0	0	0	2.991	5.661	4.943	4.262	Latent-transforming growth factor beta-binding protein 1 (Fragment) OS=Mus musculus GN=Ltbp1 PE=1 SV=8
Q8CG19	4.292E-03	0	0	0	0	2.991	5.661	4.943	4.262	Latent-transforming growth factor beta-binding protein 1 OS=Mus musculus GN=Ltbp1 PE=1 SV=2
Q9ET54	4.376E-03	0	0	0	0	4.487	7.548	8.65	6.819	Palladin OS=Mus musculus GN=Palld PE=1 SV=2
P48428	4.525E-03	0	0	0	0	8.225	4.718	6.178	5.115	Tubulin-specific chaperone A OS=Mus musculus GN=Tbca PE=1 SV=3
E9Q2A0	4.610E-03	0	0	0	0	5.982	6.605	6.178	3.41	EGF-containing fibulin-like extracellular matrix protein 2 (Fragment) OS=Mus musculus GN=Efemp2 PE=1 SV=1
Q99K41	5.109E-03	0	0	0	0	19.44	30.19	18.54	17.9	EMILIN-1 OS=Mus musculus GN=Emilin1 PE=1 SV=1
E9PWQ3	5.149E-03	0	0	0	0	21.69	20.76	13.59	12.79	Protein Col6a3 OS=Mus musculus GN=Col6a3 PE=1 SV=2
O70433	5.362E-03	0	0	0	0	3.739	6.605	6.178	7.672	Four and a half LIM domains protein 2 OS=Mus musculus GN=Fhl2 PE=1 SV=1
E9PYB0	5.638E-03	0	0	0	0	5.234	3.774	7.414	5.115	Protein Ahnak2 (Fragment) OS=Mus musculus GN=Ahnak2 PE=1 SV=8
Q9WVJ9	5.877E-03	0	0	0	0	8.973	9.435	6.178	5.115	EGF-containing fibulin-like extracellular matrix protein 2 OS=Mus musculus GN=Efemp2 PE=1 SV=1
G5E8D6	5.877E-03	0	0	0	0	8.973	9.435	6.178	5.115	EGF-containing fibulin-like extracellular matrix protein 2 OS=Mus musculus GN=Efemp2 PE=1 SV=1
P10493	5.997E-03	0	0	0	0	94.22	82.08	44.48	75.87	Nidogen-1 OS=Mus musculus GN=Nid1 PE=1 SV=2
Q9JJU8	6.089E-03	0	0	0	0	5.982	7.548	11.12	6.819	SH3 domain-binding glutamic acid-rich-like protein OS=Mus musculus GN=Sh3bgrl PE=1 SV=1
Q62059	6.316E-03	0	0	0	0	8.225	8.492	6.178	4.262	Versican core protein OS=Mus musculus GN=Vcan PE=1 SV=2
Q61081	6.334E-03	0	0	0	0	3.739	1.887	2.471	2.557	Hsp90 co-chaperone Cdc37 OS=Mus musculus GN=Cdc37 PE=1 SV=1
O08603	6.607E-03	0	0	0	0	5.982	6.605	7.414	3.41	Retinoic acid early-inducible protein 1-beta OS=Mus musculus GN=Raet1b PE=1 SV=1
P53996	6.828E-03	0	0	0	0	16.45	12.27	14.83	7.672	Cellular nucleic acid-binding protein OS=Mus musculus GN=Cnbp PE=1 SV=2
Q60866	6.964E-03	0	0	0	0	2.243	3.774	4.943	4.262	Phosphotriesterase-related protein OS=Mus musculus GN=Pter PE=1 SV=1
A2AUR3	6.964E-03	0	0	0	0	2.243	3.774	4.943	4.262	Phosphotriesterase-related protein (Fragment) OS=Mus musculus GN=Pter PE=1 SV=2
P35441	7.000E-03	0	0	0	0	2.991	6.605	4.943	5.115	Thrombospondin-1 OS=Mus musculus GN=Thbs1 PE=1 SV=1
Q80YQ1	7.000E-03	0	0	0	0	2.991	6.605	4.943	5.115	Thrombospondin 1 OS=Mus musculus GN=Thbs1 PE=1 SV=1
D3YTP0	7.130E-03	0	0	0	0	3.739	6.605	4.943	7.672	Metalloreductase STEAP3 (Fragment) OS=Mus musculus GN=Steap3 PE=1 SV=8
E9QN92	7.130E-03	0	0	0	0	3.739	6.605	4.943	7.672	Metalloreductase STEAP3 OS=Mus musculus GN=Steap3 PE=1 SV=1
Q8CI59	7.130E-03	0	0	0	0	3.739	6.605	4.943	7.672	Metalloreductase STEAP3 OS=Mus musculus GN=Steap3 PE=1 SV=1
A0A0R4J1G9	7.130E-03	0	0	0	0	3.739	6.605	4.943	7.672	Metalloreductase STEAP3 OS=Mus musculus GN=Steap3 PE=1 SV=1
Q9CWS0	7.276E-03	0	0	0	0	3.739	2.831	4.943	2.557	N(G),N(G)-dimethylarginine dimethylaminohydrolase 1 OS=Mus musculus GN=Ddah1 PE=1 SV=3
Q64314	7.326E-03	0	0	0	0	5.982	9.435	6.178	11.08	Hematopoietic progenitor cell antigen CD34 OS=Mus musculus GN=Cd34 PE=1 SV=1
P01027	7.522E-03	0	0	0	0	8.973	16.04	13.59	8.524	Complement C3 OS=Mus musculus GN=C3 PE=1 SV=3
Q3U687	7.621E-03	0	0	0	0	3.739	7.548	7.414	5.115	Protein Ifit1bl2 OS=Mus musculus GN=Ifit1bl2 PE=1 SV=1
P70290	9.163E-03	0	0	0	0	9.721	16.98	8.65	10.23	55 kDa erythrocyte membrane protein OS=Mus musculus GN=Mpp1 PE=1 SV=1
A2AN84	9.163E-03	0	0	0	0	9.721	16.98	8.65	10.23	55 kDa erythrocyte membrane protein OS=Mus musculus GN=Mpp1 PE=1 SV=1
Q03350	9.228E-03	0	0	0	0	10.47	11.32	7.414	5.115	Thrombospondin-2 OS=Mus musculus GN=Thbs2 PE=1 SV=2
Q64449	9.564E-03	0	0	0	0	5.234	8.492	6.178	11.08	C-type mannose receptor 2 OS=Mus musculus GN=Mrc2 PE=1 SV=3
P14428	9.610E-03	0	0	0	0	4.487	4.718	4.943	8.524	H-2 class I histocompatibility antigen, K-Q alpha chain (Fragment) OS=Mus musculus GN=H2-K1 PE=1 SV=1
G3UXU2	1.059E-02	0	0	0	0	2.243	2.831	4.943	4.262	Casein kinase II subunit beta (Fragment) OS=Mus musculus GN=Csnk2b PE=1 SV=2
P61622	1.059E-02	0	0	0	0	2.243	2.831	4.943	4.262	Integrin alpha-11 OS=Mus musculus GN=Itga11 PE=1 SV=1
A0A0B4J1F0	1.059E-02	0	0	0	0	2.243	2.831	4.943	4.262	Integrin alpha-11 OS=Mus musculus GN=Itga11 PE=1 SV=1
Q8K2A1	1.061E-02	0	0	0	0	4.487	3.774	3.707	1.705	PTB domain-containing engulfment adapter protein 1 OS=Mus musculus GN=Gulp1 PE=1 SV=1
E0CXC5	1.061E-02	0	0	0	0	4.487	3.774	3.707	1.705	PTB domain-containing engulfment adapter protein 1 OS=Mus musculus GN=Gulp1 PE=1 SV=1
O35646	1.094E-02	0	0	0	0	2.243	4.718	4.943	5.967	Calpain-6 OS=Mus musculus GN=Capn6 PE=1 SV=2
Q9D0F9	1.123E-02	0	0	0	0	5.982	5.661	6.178	11.08	Phosphoglucomutase-1 OS=Mus musculus GN=Pgm1 PE=1 SV=4
E9QN12	1.150E-02	0	0	0	0	5.982	2.831	7.414	5.115	Platelet-derived growth factor receptor beta OS=Mus musculus GN=Pdgfrb PE=1 SV=1
P05622	1.150E-02	0	0	0	0	5.982	2.831	7.414	5.115	Platelet-derived growth factor receptor beta OS=Mus musculus GN=Pdgfrb PE=1 SV=1
E9QPE2	1.150E-02	0	0	0	0	5.982	2.831	7.414	5.115	Platelet-derived growth factor receptor beta OS=Mus musculus GN=Pdgfrb PE=1 SV=1
Q6GQT1	1.173E-02	0	0	0	0	2.991	7.548	4.943	5.115	Alpha-2-macroglobulin-P OS=Mus musculus GN=A2m PE=2 SV=2
B2RUE8	1.194E-02	0	0	0	0	2.243	4.718	6.178	5.115	Map4k4 protein OS=Mus musculus GN=Map4k4 PE=1 SV=1
A0A0A6YWM8	1.194E-02	0	0	0	0	2.243	4.718	6.178	5.115	Mitogen-activated protein kinase kinase kinase kinase 4 OS=Mus musculus GN=Map4k4 PE=1 SV=1
P97820	1.196E-02	0	0	0	0	2.243	3.774	4.943	2.557	Mitogen-activated protein kinase kinase kinase kinase 4 OS=Mus musculus GN=Map4k4 PE=1 SV=1
F7DBB3	1.197E-02	0	0	0	0	5.234	3.774	8.65	5.115	Protein Ahnak2 (Fragment) OS=Mus musculus GN=Ahnak2 PE=1 SV=1
P24549	1.208E-02	0	0	0	0	1.496	3.774	2.471	3.41	Retinal dehydrogenase 1 OS=Mus musculus GN=Aldh1a1 PE=1 SV=5
P62965	1.354E-02	0	0	0	0	2.991	8.492	6.178	5.967	Cellular retinoic acid-binding protein 1 OS=Mus musculus GN=Crabp1 PE=1 SV=2
P38647	1.373E-02	0	0	0	0	5.234	4.718	2.471	2.557	Stress-70 protein, mitochondrial OS=Mus musculus GN=Hspa9 PE=1 SV=3
E9PWE8	1.394E-02	0	0	0	0	29.16	27.36	9.885	24.72	Dihydropyrimidinase-related protein 3 OS=Mus musculus GN=Dpysl3 PE=1 SV=1
Q9D281	1.487E-02	0	0	0	0	5.982	9.435	6.178	3.41	Protein Noxp20 OS=Mus musculus GN=Fam114a1 PE=1 SV=1
P11087	1.524E-02	0	0	0	0	32.15	33.97	16.06	16.2	Collagen alpha-1(I) chain OS=Mus musculus GN=Col1a1 PE=1 SV=4
Q08093	1.543E-02	0	0	0	0	5.234	4.718	7.414	2.557	Calponin-2 OS=Mus musculus GN=Cnn2 PE=1 SV=1
Q62188	1.736E-02	0	0	0	0	29.16	27.36	8.65	24.72	Dihydropyrimidinase-related protein 3 OS=Mus musculus GN=Dpysl3 PE=1 SV=1
Q99K51	1.826E-02	0	0	0	0	2.243	6.605	7.414	5.115	Plastin-3 OS=Mus musculus GN=Pls3 PE=1 SV=3
B1AX58	1.826E-02	0	0	0	0	2.243	6.605	7.414	5.115	Plastin-3 OS=Mus musculus GN=Pls3 PE=1 SV=1
A0A1C7CYV0	1.826E-02	0	0	0	0	2.243	6.605	7.414	5.115	Plastin-3 (Fragment) OS=Mus musculus GN=Pls3 PE=1 SV=1
D3Z7D5	1.954E-02	0	0	0	0	5.982	4.718	2.471	2.557	Collagen alpha-2(VI) chain OS=Mus musculus GN=Col6a2 PE=1 SV=1
Q8C8U0	2.115E-02	0	0	0	0	16.45	9.435	4.943	11.93	Liprin-beta-1 OS=Mus musculus GN=Ppfibp1 PE=1 SV=3
A0A0G2JGZ5	2.163E-02	0	0	0	0	8.973	5.661	2.471	6.819	PDZ and LIM domain protein 5 (Fragment) OS=Mus musculus GN=Pdlim5 PE=1 SV=1

Q8BH35	2.428E-02	0	0	0	0	2.991	4.718	8.65	4.262	Complement component C8 beta chain OS=Mus musculus GN=C8b PE=1 SV=1
D9J2Z9	2.601E-02	0	0	0	0	9.721	5.661	2.471	6.819	ENH isoform 1b OS=Mus musculus GN=Pdlim5 PE=1 SV=1
Q8CI51	2.601E-02	0	0	0	0	9.721	5.661	2.471	6.819	PDZ and LIM domain protein 5 OS=Mus musculus GN=Pdlim5 PE=1 SV=4
D9J301	2.601E-02	0	0	0	0	9.721	5.661	2.471	6.819	ENH isoform 1d OS=Mus musculus GN=Pdlim5 PE=1 SV=1
D9J300	2.601E-02	0	0	0	0	9.721	5.661	2.471	6.819	ENH isoform 1c OS=Mus musculus GN=Pdlim5 PE=1 SV=1
D9J302	2.601E-02	0	0	0	0	9.721	5.661	2.471	6.819	ENH isoform 1e OS=Mus musculus GN=Pdlim5 PE=1 SV=1
E9Q1S3	2.605E-02	0	0	0	0	3.739	11.32	4.943	7.672	Protein transport protein Sec23A OS=Mus musculus GN=Sec23a PE=1 SV=1
Q01405	2.605E-02	0	0	0	0	3.739	11.32	4.943	7.672	Protein transport protein Sec23A OS=Mus musculus GN=Sec23a PE=1 SV=2
P01942	2.880E-02	0	0	0	0	2.243	7.548	4.943	3.41	Hemoglobin subunit alpha OS=Mus musculus GN=Hba PE=1 SV=2
Q91VB8	2.880E-02	0	0	0	0	2.243	7.548	4.943	3.41	Alpha globin 1 OS=Mus musculus GN=haemaglobin alpha 2 PE=1 SV=1
Q61205	3.254E-02	0	0	0	0	1.496	6.605	4.943	3.41	Platelet-activating factor acetylhydrolase IB subunit gamma OS=Mus musculus GN=Pafah1b3 PE=1 SV=1
D3Z7E6	3.254E-02	0	0	0	0	1.496	6.605	4.943	3.41	Platelet-activating factor acetylhydrolase IB subunit gamma OS=Mus musculus GN=Pafah1b3 PE=1 SV=1
Q9CPN8	3.556E-02	0	0	0	0	9.721	13.21	3.707	5.115	Insulin-like growth factor 2 mRNA-binding protein 3 OS=Mus musculus GN=Igf2bp3 PE=1 SV=1
A0A0G2JEJ0	3.626E-02	0	0	0	0	8.973	3.774	2.471	5.115	PDZ and LIM domain protein 5 (Fragment) OS=Mus musculus GN=Pdlim5 PE=1 SV=1
P33434	3.657E-02	0	0	0	0	2.991	8.492	3.707	3.41	72 kDa type IV collagenase OS=Mus musculus GN=Mmp2 PE=1 SV=1
Q9CQ60	4.011E-02	0	0	0	0	10.47	8.492	2.471	4.262	6-phosphogluconolactonase OS=Mus musculus GN=Pgls PE=1 SV=1
O08604	5.817E-02	0	0	0	0	5.234	4.718	4.943	0	Retinoic acid early-inducible protein 1-gamma OS=Mus musculus GN=Raet1c PE=1 SV=1
Q91V35	5.849E-02	0	0	0	0	2.243	0	2.471	2.557	Receptor-type tyrosine-protein phosphatase OS=Mus musculus GN=Ptpra PE=1 SV=1
Q9D8U8	5.880E-02	0	0	0	0	2.991	2.831	0	2.557	Sorting nexin-5 OS=Mus musculus GN=Snx5 PE=1 SV=1
Q3TT92	5.892E-02	0	0	0	0	29.16	27.36	0	24.72	Dihydropyrimidinase-related protein 3 OS=Mus musculus GN=Dpysl3 PE=1 SV=1
Q60716	5.937E-02	0	0	0	0	2.991	2.831	2.471	0	Prolyl 4-hydroxylase subunit alpha-2 OS=Mus musculus GN=P4ha2 PE=1 SV=1
Q5SX75	5.937E-02	0	0	0	0	2.991	2.831	2.471	0	Procollagen-proline, 2-oxoglutarate 4-dioxygenase (Proline 4-hydroxylase), alpha II polypeptide, isoform CRA_f OS=Mus musculus GN=P4ha2 PE=1 SV=1
Q61391	5.940E-02	0	0	0	0	2.991	2.831	0	3.41	Neprilysin OS=Mus musculus GN=Mme PE=1 SV=3
F6X8L5	5.961E-02	0	0	0	0	5.234	4.718	0	4.262	6-phosphogluconolactonase (Fragment) OS=Mus musculus GN=Pgls PE=1 SV=1

Table S5 Click here to access/download;Table;Table S5.pdf

Table S5. Shown here are 524 proteins expressed in both ciliated WT and non-ciliated KO cell-derived vesicles.

		Spectral count for ciliated EV (WT)				Spectral count for non-ciliated (KO)				
Accession	T-test p-value	Sample1	Sample2	Sample3	Sample4	Sample1	Sample2	Sample3	Sample4	Description
Q8VHX6	3.9977E-05	26.12	29.48	30.88	29.52	148.8	146.2	143.3	138.1	Filamin-C OS=Mus musculus GN=Flnc PE=1 SV=3
P08775	1.3897E-04	26.91	32.64	27.14	29.52	4.487	5.661	2.471	5.967	DNA-directed RNA polymerase II subunit RPB1 OS=Mus musculus GN=Polr2a PE=1 SV=3
A0A0R4J0V5	1.3897E-04	26.91	32.64	27.14	29.52	4.487	5.661	2.471	5.967	DNA-directed RNA polymerase subunit OS=Mus musculus GN=Polr2a PE=1 SV=1
D3YUQ9	1.6781E-04	1.583	0	4.679	0	8.973	6.605	11.12	7.672	Elongation factor 1-delta (Fragment) OS=Mus musculus GN=Eef1d PE=1 SV=8
P13020	1.9109E-04	16.62	15.8	16.85	14.76	34.4	35.85	35.83	36.65	Gelsolin OS=Mus musculus GN=Gsn PE=1 SV=3
E9Q3W4	2.0309E-04	16.62	15.8	18.72	14.76	142.8	139.6	133.5	117.6	Plectin OS=Mus musculus GN=Plec PE=1 SV=1
Q9QXS1	2.0287E-04	16.62	16.85	19.65	14.76	143.6	139.6	134.7	117.6	Plectin OS=Mus musculus GN=Plec PE=1 SV=3
E9Q1T9	2.5822E-04	57.78	52.65	52.41	50.19	17.95	17.93	16.06	18.75	Exportin-2 OS=Mus musculus GN=Cse1l PE=1 SV=1
E9PX23	2.8060E-04	6.332	4.212	3.743	5.904	2.243	0	0	2.557	Metastasis-associated protein MTA1 OS=Mus musculus GN=Mta1 PE=1 SV=1
Q8K4B0	2.8060E-04	6.332	4.212	3.743	5.904	2.243	0	0	2.557	Metastasis-associated protein MTA1 OS=Mus musculus GN=Mta1 PE=1 SV=1
F8WHY8	2.8060E-04	6.332	4.212	3.743	5.904	2.243	0	0	2.557	Metastasis-associated protein MTA1 OS=Mus musculus GN=Mta1 PE=1 SV=1
Q9WV92	2.8565E-04	0	4.212	0	0	35.89	48.12	35.83	40.92	Band 4.1-like protein 3 OS=Mus musculus GN=Epb41l3 PE=1 SV=1
Q62351	2.9072E-04	83.89	75.82	76.74	70.85	3.739	4.718	4.943	8.524	Transferrin receptor protein 1 OS=Mus musculus GN=Tfrc PE=1 SV=1
M0QWP1	3.0775E-04	34.82	33.7	41.18	14.76	73.28	67.93	81.56	58.82	Agrin OS=Mus musculus GN=Agrn PE=1 SV=1
P23116	3.3446E-04	54.61	50.54	51.47	56.09	41.13	33.97	38.31	41.77	Eukaryotic translation initiation factor 3 subunit A OS=Mus musculus GN=Eif3a PE=1 SV=5
D3Z5N9	4.7416E-04	7.915	8.424	7.487	8.857	0	0	0	2.557	MCG49198 OS=Mus musculus GN=Gm5449 PE=4 SV=1
Q9ERK4	5.4543E-04	60.94	55.81	54.28	50.19	17.95	17.93	16.06	18.75	Exportin-2 OS=Mus musculus GN=Cse1l PE=1 SV=1
P43274	5.5510E-04	33.24	26.33	28.08	32.47	1.496	1.887	0	0	Histone H1.4 OS=Mus musculus GN=Hist1h1e PE=1 SV=2
A0A1D5RMD8	5.8640E-04	7.123	7.371	6.551	8.857	0	0	0	3.41	CCR4-NOT transcription complex subunit 1 OS=Mus musculus GN=Cnot1 PE=1 SV=1
Q8BFY9	6.3906E-04	25.33	23.17	22.46	32.47	7.478	6.605	7.414	11.93	Transportin-1 OS=Mus musculus GN=Tnpo1 PE=1 SV=2
Q9EQH3	6.4096E-04	39.57	47.39	43.05	44.28	23.18	28.31	28.42	29.84	Vacuolar protein sorting-associated protein 35 OS=Mus musculus GN=Vps35 PE=1 SV=1
Q61656	6.9064E-04	41.16	38.96	42.11	32.47	9.721	8.492	6.178	6.819	Probable ATP-dependent RNA helicase DDX5 OS=Mus musculus GN=Ddx5 PE=1 SV=2
Q99J09	7.1787E-04	10.29	11.58	11.23	14.76	2.991	3.774	4.943	5.967	Methylosome protein 50 OS=Mus musculus GN=Wdr77 PE=1 SV=1
Q9DAW6	7.3271E-04	25.33	30.54	26.2	23.62	17.2	19.81	17.3	12.79	U4/U6 small nuclear ribonucleoprotein Prp4 OS=Mus musculus GN=Prpf4 PE=1 SV=1
J3KMQ2	7.5028E-04	34.82	35.8	39.31	41.33	14.21	19.81	19.77	18.75	Protein Gm5422 OS=Mus musculus GN=Gm5422 PE=4 SV=1
P14824	7.8030E-04	97.35	80.03	88.91	91.52	158.5	159.5	150.8	169.6	Annexin A6 OS=Mus musculus GN=Anxa6 PE=1 SV=3
P14094	8.1480E-04	14.25	21.06	20.59	20.67	3.739	7.548	8.65	5.967	Sodium/potassium-transporting ATPase subunit beta-1 OS=Mus musculus GN=Atp1b1 PE=1 SV=1
P46935	8.8205E-04	5.54	4.212	3.743	5.904	26.92	21.7	28.42	29.84	E3 ubiquitin-protein ligase NEDD4 OS=Mus musculus GN=Nedd4 PE=1 SV=3
O70318	9.0375E-04	5.54	6.318	2.808	5.904	30.66	34.91	33.36	41.77	Band 4.1-like protein 2 OS=Mus musculus GN=Epb41l2 PE=1 SV=2
P30285	9.5908E-04	9.497	13.69	14.04	11.81	2.991	6.605	4.943	3.41	Cyclin-dependent kinase 4 OS=Mus musculus GN=Cdk4 PE=1 SV=1
P11983	9.9369E-04	125.1	134.8	137.6	141.7	74.03	74.54	67.96	69.05	T-complex protein 1 subunit alpha OS=Mus musculus GN=Tcp1 PE=1 SV=3
P26516	1.0206E-03	19.79	25.27	21.52	29.52	9.721	11.32	11.12	17.05	26S proteasome non-ATPase regulatory subunit 7 OS=Mus musculus GN=Psmd7 PE=1 SV=2
P60766	1.0399E-03	20.58	23.17	20.59	20.67	61.32	59.44	59.31	48.59	Cell division control protein 42 homolog OS=Mus musculus GN=Cdc42 PE=1 SV=2
Q922D8	1.0701E-03	68.86	73.71	71.13	70.85	46.36	50.95	49.43	40.92	C-1-tetrahydrofolate synthase, cytoplasmic OS=Mus musculus GN=Mthfd1 PE=1 SV=4
Q9R0E1	1.1799E-03	15.04	17.9	9.359	11.81	8.225	10.38	3.707	3.41	Procollagen-lysine,2-oxoglutarate 5-dioxygenase 3 OS=Mus musculus GN=Plod3 PE=1 SV=1
P47962	1.1958E-03	110.8	130.6	118.9	168.3	33.65	21.7	37.07	67.34	60S ribosomal protein L5 OS=Mus musculus GN=Rpl5 PE=1 SV=3
Q8K2Z4	1.2315E-03	4.749	8.424	4.679	5.904	0	1.887	0	0	Condensin complex subunit 1 OS=Mus musculus GN=Ncapd2 PE=1 SV=2
A0A0R4J0H7	1.2315E-03	4.749	8.424	4.679	5.904	0	1.887	0	0	Condensin complex subunit 1 OS=Mus musculus GN=Ncapd2 PE=1 SV=1
Q80X90	1.2448E-03	40.36	27.38	42.11	20.67	121.9	142.5	124.8	115.1	Filamin-B OS=Mus musculus GN=Flnb PE=1 SV=3
P42932	1.2940E-03	152	160.1	141.3	132.8	85.25	101	91.44	86.95	T-complex protein 1 subunit theta OS=Mus musculus GN=Cct8 PE=1 SV=3
Q61739	1.3996E-03	28.49	27.38	24.33	26.57	4.487	5.661	8.65	4.262	Integrin alpha-6 OS=Mus musculus GN=Itga6 PE=1 SV=3
P10107	1.4120E-03	71.23	76.87	65.51	76.76	171.2	143.4	159.4	174.7	Annexin A1 OS=Mus musculus GN=Anxa1 PE=1 SV=2
B7ZCU0	1.4258E-03	0	2.106	3.743	0	2.991	6.605	7.414	3.41	Abl interactor 1 OS=Mus musculus GN=Abi1 PE=1 SV=1
B7ZCU2	1.4258E-03	0	2.106	3.743	0	2.991	6.605	7.414	3.41	Abl interactor 1 OS=Mus musculus GN=Abi1 PE=1 SV=1
B7ZCU3	1.4258E-03	0	2.106	3.743	0	2.991	6.605	7.414	3.41	Abl interactor 1 OS=Mus musculus GN=Abi1 PE=1 SV=1
B7ZCU4	1.4258E-03	0	2.106	3.743	0	2.991	6.605	7.414	3.41	Abl interactor 1 OS=Mus musculus GN=Abi1 PE=1 SV=1
B7ZCU5	1.4258E-03	0	2.106	3.743	0	2.991	6.605	7.414	3.41	Abl interactor 1 OS=Mus musculus GN=Abi1 PE=1 SV=1
Q8CBW3	1.4258E-03	0	2.106	3.743	0	2.991	6.605	7.414	3.41	Abl interactor 1 OS=Mus musculus GN=Abi1 PE=1 SV=3
J3QNK8	1.4258E-03	0	2.106	3.743	0	2.991	6.605	7.414	3.41	Abl interactor 1 OS=Mus musculus GN=Abi1 PE=1 SV=1
Q9CSH3	1.4274E-03	31.66	34.75	45.86	35.43	0	0	0	1.705	Exosome complex exonuclease RRP44 OS=Mus musculus GN=Dis3 PE=1 SV=4
F6QTS1	1.4984E-03	11.87	6.318	9.359	8.857	2.243	0	0	0	COP9 signalosome complex subunit 4 (Fragment) OS=Mus musculus GN=Cops4 PE=1 SV=1
Q9CZU3	1.4958E-03	12.66	16.85	19.65	20.67	0	2.831	3.707	1.705	Superkiller viralicidic activity 2-like 2 OS=Mus musculus GN=Skiv2l2 PE=1 SV=1
Q02248	1.5575E-03	9.497	11.58	12.17	11.81	37.39	33.02	30.89	32.39	Catenin beta-1 OS=Mus musculus GN=Ctnnb1 PE=1 SV=1
P80314	1.5821E-03	171.7	152.7	215.2	233.2	56.83	66.05	84.03	102.3	T-complex protein 1 subunit beta OS=Mus musculus GN=Cct2 PE=1 SV=4
P14685	1.7174E-03	56.19	61.07	66.45	53.14	18.69	30.19	42.01	25.57	26S proteasome non-ATPase regulatory subunit 3 OS=Mus musculus GN=Psmd3 PE=1 SV=3
Q3THK7	1.7554E-03	31.66	34.75	30.88	44.28	12.71	10.38	9.885	15.34	GMP synthase [glutamine-hydrolyzing] OS=Mus musculus GN=Gmps PE=1 SV=2
O08759	2.0716E-03	6.332	6.318	4.679	5.904	2.243	0	0	0	Ubiquitin-protein ligase E3A OS=Mus musculus GN=Ube3a PE=1 SV=2
Q3UH59	2.1095E-03	105.3	101.1	103.9	91.52	95.71	86.8	91.44	81.83	Myosin-10 OS=Mus musculus GN=Myh10 PE=1 SV=1
Q9Z1F9	2.0945E-03	37.2	36.86	40.24	38.38	14.21	16.98	13.59	6.819	SUMO-activating enzyme subunit 2 OS=Mus musculus GN=Uba2 PE=1 SV=1
F7D5L2	2.0943E-03	10.29	10.53	11.23	14.76	2.991	3.774	4.943	5.115	Methylosome protein 50 (Fragment) OS=Mus musculus GN=Wdr77 PE=1 SV=1
Q91ZX7	2.0947E-03	0	3.159	0	5.904	37.39	33.02	28.42	28.98	Prolow-density lipoprotein receptor-related protein 1 OS=Mus musculus GN=Lrp1 PE=1 SV=1
A0A0R4J0I9	2.0947E-03	0	3.159	0	5.904	37.39	33.02	28.42	28.98	Low density lipoprotein receptor-related protein 1 OS=Mus musculus GN=Lrp1 PE=1 SV=1
F8VQJ3	2.1025E-03	28.49	34.75	33.69	26.57	83.75	99.07	75.38	71.6	Laminin subunit gamma-1 OS=Mus musculus GN=Lamc1 PE=1 SV=1
Q8C1A5	2.1280E-03	53.03	38.96	53.34	38.38	3.739	2.831	0	1.705	Thimet oligopeptidase OS=Mus musculus GN=Thop1 PE=1 SV=1
A0A0R4IZY0	2.1280E-03	53.03	38.96	53.34	38.38	3.739	2.831	0	1.705	Thimet oligopeptidase OS=Mus musculus GN=Thop1 PE=1 SV=1
Q9JLJ2	2.1553E-03	25.33	23.17	19.65	20.67	7.478	6.605	6.178	9.377	4-trimethylaminobutyraldehyde dehydrogenase OS=Mus musculus GN=Aldh9a1 PE=1 SV=1
Q3U367	2.1553E-03	25.33	23.17	19.65	20.67	7.478	6.605	6.178	9.377	4-trimethylaminobutyraldehyde dehydrogenase OS=Mus musculus GN=Aldh9a1 PE=1 SV=1
H3BLJ9	2.2983E-03	41.16	46.33	38.37	20.67	15.7	20.76	22.24	0	S-formylglutathione hydrolase OS=Mus musculus GN=Esd PE=1 SV=1
P10639	2.3325E-03	0	8.424	10.29	17.71	11.96	23.59	22.24	35.8	Thioredoxin OS=Mus musculus GN=Txn PE=1 SV=3
P49717	2.3613E-03	39.57	48.44	41.18	44.28	17.95	16.98	21.01	17.05	DNA replication licensing factor MCM4 OS=Mus musculus GN=Mcm4 PE=1 SV=1
Q9D0T1	2.4385E-03	11.87	9.477	12.17	8.857	7.478	6.605	7.414	5.115	NHP2-like protein 1 OS=Mus musculus GN=Snu13 PE=1 SV=4
A0A0R4J049	2.4634E-03	14.25	15.8	12.17	14.76	5.234	7.548	4.943	9.377	Protein arginine N-methyltransferase 5 OS=Mus musculus GN=Prmt5 PE=1 SV=1
Q922B2	2.5500E-03	88.64	71.6	85.16	91.52	21.69	29.25	22.24	19.61	Aspartate--tRNA ligase, cytoplasmic OS=Mus musculus GN=Dars PE=1 SV=2
A2ASQ1	1.2973E-05	31.66	31.59	39.31	14.76	68.05	66.99	77.85	52	Agrin OS=Mus musculus GN=Agrn PE=1 SV=1
Q8BTS0	2.5976E-03	41.16	38.96	42.11	32.47	9.721	8.492	0	6.819	DEAD (Asp-Glu-Ala-Asp) box polypeptide 5 OS=Mus musculus GN=Ddx5 PE=1 SV=1
P43277	2.6189E-03	28.49	25.27	30.88	38.38	1.496	1.887	0	0	Histone H1.3 OS=Mus musculus GN=Hist1h1d PE=1 SV=2
P15864	2.6189E-03	28.49	25.27	30.88	38.38	1.496	1.887	0	0	Histone H1.2 OS=Mus musculus GN=Hist1h1c PE=1 SV=2
D3Z2H2	2.6231E-03	0	5.265	3.743	5.904	20.19	16.98	21.01	22.16	Catenin delta-1 OS=Mus musculus GN=Ctnnd1 PE=1 SV=1
E9Q8Z8	2.6231E-03	0	5.265	3.743	5.904	20.19	16.98	21.01	22.16	Catenin delta-1 OS=Mus musculus GN=Ctnnd1 PE=1 SV=1
Q7TPR4	2.7395E-03	22.16	27.38	23.4	29.52	70.29	74.54	51.9	71.6	Alpha-actinin-1 OS=Mus musculus GN=Actn1 PE=1 SV=1
A2A7S7	2.7550E-03	2.374	0	3.743	0	8.225	8.492	12.36	10.23	Tyrosine--tRNA ligase OS=Mus musculus GN=Yars PE=1 SV=1
Q91WQ3	2.7550E-03	2.374	0	3.743	0	8.225	8.492	12.36	10.23	Tyrosine--tRNA ligase, cytoplasmic OS=Mus musculus GN=Yars PE=1 SV=3
Q9EPL8	2.7980E-03	14.25	16.85	16.85	23.62	0	4.718	0	3.41	Importin-7 OS=Mus musculus GN=Ipo7 PE=1 SV=2
Q3TXS7	2.9106E-03	33.24	40.01	29.01	32.47	17.2	17.93	16.06	15.34	26S proteasome non-ATPase regulatory subunit 1 OS=Mus musculus GN=Psmd1 PE=1 SV=1
P02468	2.9437E-03	27.7	33.7	30.88	26.57	77.02	90.58	65.49	65.64	Laminin subunit gamma-1 OS=Mus musculus GN=Lamc1 PE=1 SV=2
P62317	3.0055E-03	7.915	8.424	7.487	8.857	0	0	2.471	2.557	Small nuclear ribonucleoprotein Sm D2 OS=Mus musculus GN=Snrpd2 PE=1 SV=1
H3BJP2	3.0555E-03	41.16	46.33	37.43	20.67	15.7	20.76	22.24	0	S-formylglutathione hydrolase (Fragment) OS=Mus musculus GN=Esd PE=1 SV=1
Q8VDM4	3.0742E-03	70.44	62.13	65.51	73.8	28.42	36.8	37.07	41.77	26S proteasome non-ATPase regulatory subunit 2 OS=Mus musculus GN=Psmd2 PE=1 SV=1
F6YY69	3.1822E-03	70.44	85.29	71.13	118.1	17.2	24.53	35.83	57.11	14-3-3 protein theta (Fragment) OS=Mus musculus GN=Ywhaq PE=1 SV=1
F8WIT2	3.2005E-03	104.5	84.24	97.33	94.47	163.8	169.8	152	179	Annexin OS=Mus musculus GN=Anxa6 PE=1 SV=1
P27601	3.2321E-03	1.583	2.106	0	0	5.982	9.435	6.178	7.672	Guanine nucleotide-binding protein subunit alpha-13 OS=Mus musculus GN=Gna13 PE=1 SV=1
E9Q6R7	3.2437E-03	0	0	1.872	0	4.487	2.831	6.178	5.115	Protein Utrn OS=Mus musculus GN=Utrn PE=1 SV=1
Q5SXR6	3.3435E-03	327.7	309.6	282.6	283.4	141.3	164.2	159.4	172.2	Clathrin heavy chain OS=Mus musculus GN=Cltc PE=1 SV=1
O88477	3.3787E-03	3.957	2.106	2.808	0	11.22	9.435	8.65	4.262	Insulin-like growth factor 2 mRNA-binding protein 1 OS=Mus musculus GN=Igf2bp1 PE=1 SV=1
Q5SQB5	3.4491E-03	45.9	35.8	32.76	38.38	20.19	16.04	18.54	16.2	Nucleophosmin OS=Mus musculus GN=Npm1 PE=1 SV=1
Q8CIG8	3.7007E-03	12.66	15.8	11.23	14.76	5.982	10.38	4.943	11.08	Protein arginine N-methyltransferase 5 OS=Mus musculus GN=Prmt5 PE=1 SV=3
F6VW30	3.7286E-03	70.44	86.35	71.13	121	16.45	24.53	35.83	57.11	14-3-3 protein theta (Fragment) OS=Mus musculus GN=Ywhaq PE=1 SV=1
P18760	3.7241E-03	53.03	63.18	56.15	50.19	100.2	90.58	106.3	92.06	Cofilin-1 OS=Mus musculus GN=Cfl1 PE=1 SV=3
P30999	3.7519E-03	0	5.265	3.743	5.904	20.19	16.04	21.01	23.02	Catenin delta-1 OS=Mus musculus GN=Ctnnd1 PE=1 SV=2
E9Q905	3.7519E-03	0	5.265	3.743	5.904	20.19	16.04	21.01	23.02	Catenin delta-1 OS=Mus musculus GN=Ctnnd1 PE=1 SV=1
E9Q904	3.8046E-03	0	5.265	3.743	5.904	20.19	16.04	21.01	22.16	Catenin delta-1 OS=Mus musculus GN=Ctnnd1 PE=1 SV=1
F8WGL3	3.8699E-03	53.03	63.18	57.09	50.19	98.7	89.63	106.3	92.06	Cofilin-1 OS=Mus musculus GN=Cfl1 PE=1 SV=1
Q68FL6	3.9447E-03	44.32	34.75	43.05	32.47	8.225	9.435	6.178	10.23	Methionine--tRNA ligase, cytoplasmic OS=Mus musculus GN=Mars PE=1 SV=1
E9QB02	3.9447E-03	44.32	34.75	43.05	32.47	8.225	9.435	6.178	10.23	Methionine--tRNA ligase, cytoplasmic OS=Mus musculus GN=Mars PE=1 SV=1
E9QKR0	3.9684E-03	94.97	102.1	80.48	109.2	31.41	35.85	44.48	46.88	Guanine nucleotide-binding protein G(I)/G(S)/G(T) subunit beta-2 OS=Mus musculus GN=Gnb2 PE=1 SV=1
P46061	3.9649E-03	18.99	21.06	12.17	17.71	1.496	1.887	0	5.967	Ran GTPase-activating protein 1 OS=Mus musculus GN=Rangap1 PE=1 SV=2
P62880	4.0316E-03	95.77	102.1	80.48	109.2	31.41	35.85	44.48	46.88	Guanine nucleotide-binding protein G(I)/G(S)/G(T) subunit beta-2 OS=Mus musculus GN=Gnb2 PE=1 SV=3
A0A0N4SVS6	4.0425E-03	3.166	4.212	0	0	19.44	17.93	17.3	9.377	Cellular nucleic acid-binding protein OS=Mus musculus GN=Cnbp PE=1 SV=1
Q61879	4.2633E-03	104.5	102.1	103.9	91.52	95.71	87.75	93.91	82.69	Myosin-10 OS=Mus musculus GN=Myh10 PE=1 SV=2
P68037	4.1297E-03	1.583	3.159	0	0	5.982	7.548	6.178	3.41	Ubiquitin-conjugating enzyme E2 L3 OS=Mus musculus GN=Ube2l3 PE=1 SV=1
Q68FD5	4.1908E-03	329.2	309.6	282.6	280.5	141.3	165.1	160.6	173.9	Clathrin heavy chain 1 OS=Mus musculus GN=Cltc PE=1 SV=3
A2AN08	4.2293E-03	20.58	25.27	31.82	17.71	8.973	16.04	17.3	9.377	E3 ubiquitin-protein ligase UBR4 OS=Mus musculus GN=Ubr4 PE=1 SV=1
A1BN54	4.2464E-03	22.16	27.38	22.46	29.52	66.55	67.93	45.72	66.49	Alpha actinin 1a OS=Mus musculus GN=Actn1 PE=1 SV=1
Q61937	4.2950E-03	46.7	35.8	32.76	38.38	20.19	16.98	18.54	16.2	Nucleophosmin OS=Mus musculus GN=Npm1 PE=1 SV=1
P58252	4.3118E-03	315.8	278	268.6	262.7	235.5	180.2	198.9	210.6	Elongation factor 2 OS=Mus musculus GN=Eef2 PE=1 SV=2
E9QN70	4.3834E-03	7.915	9.477	9.359	11.81	33.65	24.53	27.19	28.13	Laminin subunit beta-1 OS=Mus musculus GN=Lamb1 PE=1 SV=1
P02469	4.3834E-03	7.915	9.477	9.359	11.81	33.65	24.53	27.19	28.13	Laminin subunit beta-1 OS=Mus musculus GN=Lamb1 PE=1 SV=3
D3YVC1	4.4314E-03	97.35	108.5	102	135.8	17.95	28.31	58.08	73.31	40S ribosomal protein S2 (Fragment) OS=Mus musculus GN=Rps2 PE=1 SV=1
B7FAV1	4.4552E-03	78.35	76.87	92.65	59.04	226.6	184	170.5	179.9	Filamin, alpha (Fragment) OS=Mus musculus GN=Flna PE=1 SV=1
B7ZNU9	4.4711E-03	3.166	0	0	0	7.478	5.661	6.178	3.41	Beta-2-syntrophin OS=Mus musculus GN=Sntb2 PE=2 SV=1
B7FAU9	4.5721E-03	78.35	76.87	92.65	59.04	227.3	184	170.5	180.7	Filamin, alpha OS=Mus musculus GN=Flna PE=1 SV=1
Q8BTM8	4.5721E-03	78.35	76.87	92.65	59.04	227.3	184	170.5	180.7	Filamin-A OS=Mus musculus GN=Flna PE=1 SV=5
Q8CGC7	4.5905E-03	129.8	141.1	122.6	115.1	51.6	64.16	63.02	72.46	Bifunctional glutamate/proline--tRNA ligase OS=Mus musculus GN=Eprs PE=1 SV=4
P12815	4.6137E-03	15.83	7.371	11.23	17.71	13.46	5.661	9.885	15.34	Programmed cell death protein 6 OS=Mus musculus GN=Pdcd6 PE=1 SV=2
P20152	4.7288E-03	36.41	38.96	36.5	41.33	117.4	90.58	81.56	108.3	Vimentin OS=Mus musculus GN=Vim PE=1 SV=3
Q9D2R0	4.7819E-03	6.332	2.106	4.679	0	11.96	11.32	14.83	11.08	Acetoacetyl-CoA synthetase OS=Mus musculus GN=Aacs PE=1 SV=1
E9Q634	4.8632E-03	4.749	5.265	7.487	5.904	0	0	0	1.705	Unconventional myosin-Ie OS=Mus musculus GN=Myo1e PE=1 SV=1
Q5SQB0	4.8659E-03	46.7	34.75	32.76	35.43	20.19	16.98	18.54	16.2	Nucleophosmin OS=Mus musculus GN=Npm1 PE=1 SV=1
E9PXM6	5.1282E-03	0	6.318	7.487	0	5.982	9.435	12.36	5.967	Equilibrative nucleoside transporter 1 OS=Mus musculus GN=Slc29a1 PE=1 SV=1
P62082	5.1578E-03	13.45	14.74	9.359	11.81	4.487	3.774	3.707	4.262	40S ribosomal protein S7 OS=Mus musculus GN=Rps7 PE=2 SV=1
P11214	5.1782E-03	11.08	9.477	9.359	17.71	0	0	0	1.705	Tissue-type plasminogen activator OS=Mus musculus GN=Plat PE=1 SV=3
P68254	5.1927E-03	70.44	85.29	71.13	126.9	17.2	24.53	35.83	56.26	14-3-3 protein theta OS=Mus musculus GN=Ywhaq PE=1 SV=1
P26041	5.1949E-03	87.85	89.51	77.68	82.66	198.9	194.4	232.3	163.7	Moesin OS=Mus musculus GN=Msn PE=1 SV=3
Q9DBJ1	5.2862E-03	39.57	42.12	28.08	41.33	65.8	73.59	60.55	89.51	Phosphoglycerate mutase 1 OS=Mus musculus GN=Pgam1 PE=1 SV=3
A0A140T8L5	5.2992E-03	99.72	109.5	94.52	135.8	24.68	23.59	48.19	80.13	Protein Rps2-ps6 OS=Mus musculus GN=Rps2-ps6 PE=3 SV=1
D3Z7H6	5.3130E-03	0	5.265	3.743	5.904	20.19	15.1	21.01	23.02	Catenin delta-1 OS=Mus musculus GN=Ctnnd1 PE=1 SV=1
E9Q8Z4	5.3130E-03	0	5.265	3.743	5.904	20.19	15.1	21.01	23.02	Catenin delta-1 OS=Mus musculus GN=Ctnnd1 PE=1 SV=1
Q9DBC7	5.4406E-03	3.166	3.159	4.679	0	11.96	20.76	21.01	17.05	cAMP-dependent protein kinase type I-alpha regulatory subunit OS=Mus musculus GN=Prkar1a PE=1 SV=3
Q61753	5.6400E-03	68.86	69.5	64.57	53.14	60.57	53.78	51.9	36.65	D-3-phosphoglycerate dehydrogenase OS=Mus musculus GN=Phgdh PE=1 SV=3
S4R1I6	5.6499E-03	34.82	34.75	39.31	26.57	8.973	7.548	0	5.967	MCG2872, isoform CRA_b OS=Mus musculus GN=Ddx5 PE=1 SV=1
B1B0C7	5.7747E-03	372	388.6	343.5	454.6	815.1	756.7	713	664.9	Basement membrane-specific heparan sulfate proteoglycan core protein OS=Mus musculus GN=Hspg2 PE=1 SV=1
Q6IRU2	5.9680E-03	13.45	18.95	16.85	26.57	38.14	41.51	46.96	40.92	Tropomyosin alpha-4 chain OS=Mus musculus GN=Tpm4 PE=1 SV=3
Q62470	6.0487E-03	71.23	53.7	64.57	56.09	14.96	22.64	27.19	22.16	Integrin alpha-3 OS=Mus musculus GN=Itga3 PE=1 SV=1
E9PZ16	6.1781E-03	379.9	392.8	349.1	469.4	822.5	762.4	715.5	675.1	Basement membrane-specific heparan sulfate proteoglycan core protein OS=Mus musculus GN=Hspg2 PE=1 SV=1
Q4FE56	6.2442E-03	37.2	48.44	38.37	38.38	11.96	12.27	13.59	20.46	Probable ubiquitin carboxyl-terminal hydrolase FAF-X OS=Mus musculus GN=Usp9x PE=1 SV=1
P70398	6.2442E-03	37.2	48.44	38.37	38.38	11.96	12.27	13.59	20.46	Probable ubiquitin carboxyl-terminal hydrolase FAF-X OS=Mus musculus GN=Usp9x PE=1 SV=2
Q9CQV8	6.2301E-03	57.78	56.86	54.28	100.4	21.69	19.81	29.66	49.44	14-3-3 protein beta/alpha OS=Mus musculus GN=Ywhab PE=1 SV=3
Q9D1A2	6.5443E-03	14.25	10.53	14.04	11.81	0	2.831	0	2.557	Cytosolic non-specific dipeptidase OS=Mus musculus GN=Cndp2 PE=1 SV=1
D3YZX3	6.5870E-03	89.43	98.98	76.74	109.2	28.42	32.08	44.48	40.92	Guanine nucleotide-binding protein G(I)/G(S)/G(T) subunit beta-2 OS=Mus musculus GN=Gnb2 PE=1 SV=1
P40142	6.5846E-03	49.86	54.76	56.15	47.23	28.42	36.8	21.01	19.61	Transketolase OS=Mus musculus GN=Tkt PE=1 SV=1
P08113	6.8871E-03	22.95	24.22	24.33	26.57	18.69	18.87	17.3	23.02	Endoplasmin OS=Mus musculus GN=Hsp90b1 PE=1 SV=2
Q5SW19	6.9606E-03	15.04	18.95	18.72	20.67	8.225	6.605	11.12	8.524	Clustered mitochondria protein homolog OS=Mus musculus GN=Cluh PE=1 SV=2

A0A0R4J140	6.9606E-03	15.04	18.95	18.72	20.67	8.225	6.605	11.12	8.524	Clustered mitochondria protein homolog OS=Mus musculus GN=Cluh PE=1 SV=1
E9PZX7	7.0650E-03	7.123	10.53	10.29	5.904	2.243	6.605	4.943	3.41	Cyclin-dependent kinase 4 (Fragment) OS=Mus musculus GN=Cdk4 PE=1 SV=1
P06745	7.1050E-03	4.749	8.424	6.551	0	14.21	19.81	22.24	18.75	Glucose-6-phosphate isomerase OS=Mus musculus GN=Gpi PE=1 SV=4
Q5F2E8	7.1030E-03	1.583	0	0	0	5.982	6.605	8.65	5.115	Serine/threonine-protein kinase TAO1 OS=Mus musculus GN=Taok1 PE=1 SV=1
P15532	7.1118E-03	18.2	27.38	20.59	11.81	50.85	50.95	69.2	44.33	Nucleoside diphosphate kinase A OS=Mus musculus GN=Nme1 PE=1 SV=1
Q9CWF2	7.2181E-03	243	206.4	222.7	135.8	296.9	280.2	266.9	224.2	Tubulin beta-2B chain OS=Mus musculus GN=Tubb2b PE=1 SV=1
Q8BVQ9	7.2558E-03	48.28	57.92	45.86	62	12.71	19.81	25.95	17.05	26S protease regulatory subunit 7 OS=Mus musculus GN=Psmc2 PE=1 SV=1
Q61191	7.2890E-03	15.04	9.477	11.23	11.81	1.496	2.831	0	3.41	Host cell factor 1 OS=Mus musculus GN=Hcfc1 PE=1 SV=2
F6YTZ4	7.2979E-03	98.93	109.5	96.39	135.8	19.44	23.59	53.13	80.98	Protein Gm6576 OS=Mus musculus GN=Gm6576 PE=3 SV=2
P25444	7.2876E-03	104.5	112.7	102.9	135.8	27.67	28.31	58.08	86.1	40S ribosomal protein S2 OS=Mus musculus GN=Rps2 PE=1 SV=3
O88342	7.3340E-03	21.37	21.06	19.65	14.76	29.16	28.31	24.71	25.57	WD repeat-containing protein 1 OS=Mus musculus GN=Wdr1 PE=1 SV=3
P46471	7.4418E-03	48.28	58.97	45.86	62	13.46	19.81	25.95	17.05	26S protease regulatory subunit 7 OS=Mus musculus GN=Psmc2 PE=1 SV=5
P46664	7.5288E-03	14.25	15.8	19.65	23.62	1.496	4.718	3.707	1.705	Adenylosuccinate synthetase isozyme 2 OS=Mus musculus GN=Adss PE=1 SV=2
A0A087WS56	7.5463E-03	37.99	35.8	42.11	38.38	387.3	362.3	208.8	277.9	Fibronectin OS=Mus musculus GN=Fn1 PE=1 SV=1
Q9CQW9	7.5996E-03	0	5.265	7.487	0	11.96	12.27	13.59	10.23	Interferon-induced transmembrane protein 3 OS=Mus musculus GN=Ifitm3 PE=1 SV=1
F6RPJ9	7.6306E-03	15.04	14.74	8.423	11.81	4.487	0	0	4.262	Insulin-degrading enzyme (Fragment) OS=Mus musculus GN=Ide PE=1 SV=1
Q5NC80	7.7732E-03	17.41	27.38	20.59	11.81	48.6	50.95	69.2	43.47	Nucleoside diphosphate kinase (Fragment) OS=Mus musculus GN=Nme1 PE=1 SV=1
Q920B9	7.7924E-03	9.497	6.318	11.23	14.76	2.243	0	0	2.557	FACT complex subunit SPT16 OS=Mus musculus GN=Supt16h PE=1 SV=2
G3X956	7.7924E-03	9.497	6.318	11.23	14.76	2.243	0	0	2.557	FACT complex subunit SPT16 OS=Mus musculus GN=Supt16 PE=1 SV=1
D3Z536	7.8935E-03	94.97	103.2	91.71	135.8	17.2	20.76	53.13	78.42	Protein Gm8225 OS=Mus musculus GN=Gm8225 PE=3 SV=1
P68372	7.9313E-03	246.1	215.9	233	135.8	311.1	292.5	281.7	239.5	Tubulin beta-4B chain OS=Mus musculus GN=Tubb4b PE=1 SV=1
B1AUX2	7.9058E-03	15.04	9.477	12.17	11.81	1.496	2.831	0	3.41	Host cell factor 1 OS=Mus musculus GN=Hcfc1 PE=1 SV=1
P62874	7.9091E-03	85.48	93.72	70.19	106.3	33.65	30.19	39.54	37.51	Guanine nucleotide-binding protein G(I)/G(S)/G(T) subunit beta-1 OS=Mus musculus GN=Gnb1 PE=1 SV=3
Q8BMJ2	7.9445E-03	69.65	70.55	72.06	67.9	50.1	48.12	39.54	52	Leucine--tRNA ligase, cytoplasmic OS=Mus musculus GN=Lars PE=1 SV=2
Q99L27	8.1298E-03	15.04	10.53	11.23	17.71	0	3.774	0	3.41	GMP reductase 2 OS=Mus musculus GN=Gmpr2 PE=1 SV=2
E9QNN1	8.1965E-03	117.1	91.61	85.16	73.8	29.16	32.08	27.19	33.24	ATP-dependent RNA helicase A OS=Mus musculus GN=Dhx9 PE=1 SV=1
A0A087WPL5	8.1965E-03	117.1	91.61	85.16	73.8	29.16	32.08	27.19	33.24	ATP-dependent RNA helicase A OS=Mus musculus GN=Dhx9 PE=1 SV=1
Q3UHL6	8.2428E-03	38.78	36.86	43.99	41.33	398.6	363.2	208.8	284.7	Fibronectin OS=Mus musculus GN=Fn1 PE=1 SV=1
P52293	8.2573E-03	11.87	7.371	14.04	11.81	5.982	3.774	6.178	6.819	Importin subunit alpha-1 OS=Mus musculus GN=Kpna2 PE=1 SV=2
Q7TMM9	8.2655E-03	240.6	204.3	223.7	129.9	300.6	281.2	266.9	224.2	Tubulin beta-2A chain OS=Mus musculus GN=Tubb2a PE=1 SV=1
Q8K354	8.3182E-03	3.166	0	0	0	11.22	13.21	18.54	13.64	Carbonyl reductase [NADPH] 3 OS=Mus musculus GN=Cbr3 PE=1 SV=1
J3QNW0	8.5121E-03	45.11	48.44	37.43	35.43	5.982	12.27	13.59	16.2	DNA (cytosine-5)-methyltransferase OS=Mus musculus GN=Dnmt1 PE=1 SV=1
P13864	8.5121E-03	45.11	48.44	37.43	35.43	5.982	12.27	13.59	16.2	DNA (cytosine-5)-methyltransferase 1 OS=Mus musculus GN=Dnmt1 PE=1 SV=5
A0A087WR50	8.5145E-03	38.78	36.86	43.99	41.33	401.5	370.8	208.8	288.1	Fibronectin OS=Mus musculus GN=Fn1 PE=1 SV=1
Q920E5	8.5396E-03	0	0	1.872	0	5.982	4.718	7.414	2.557	Farnesyl pyrophosphate synthase OS=Mus musculus GN=Fdps PE=1 SV=1
D3Z2H7	8.5584E-03	0	5.265	3.743	5.904	18.69	13.21	17.3	21.31	Catenin delta-1 OS=Mus musculus GN=Ctnnd1 PE=1 SV=1
P11276	8.5928E-03	38.78	36.86	43.99	41.33	401.5	371.7	208.8	287.3	Fibronectin OS=Mus musculus GN=Fn1 PE=1 SV=4
E9Q2X6	8.5939E-03	9.497	16.85	18.72	23.62	0	2.831	0	2.557	Structural maintenance of chromosomes protein OS=Mus musculus GN=Smc4 PE=1 SV=1
Q8CG47	8.5939E-03	9.497	16.85	18.72	23.62	0	2.831	0	2.557	Structural maintenance of chromosomes protein 4 OS=Mus musculus GN=Smc4 PE=1 SV=1
contaminant_UBIQUITIN02	8.7200E-03	39.57	44.23	39.31	29.52	80.01	64.16	61.78	59.67	no description
Z4YLI8	8.8100E-03	14.25	18.95	18.72	20.67	8.225	5.661	9.885	8.524	Clustered mitochondria protein homolog OS=Mus musculus GN=Cluh PE=1 SV=1
P62908	8.8084E-03	95.77	81.08	95.46	73.8	60.57	56.61	67.96	58.82	40S ribosomal protein S3 OS=Mus musculus GN=Rps3 PE=1 SV=1
P14069	8.9384E-03	24.54	25.27	28.08	17.71	41.87	38.68	49.43	27.28	Protein S100-A6 OS=Mus musculus GN=S100a6 PE=1 SV=3
Q05793	9.0058E-03	316.6	342.2	306	422.2	735.1	689.7	656.1	592.4	Basement membrane-specific heparan sulfate proteoglycan core protein OS=Mus musculus GN=Hspg2 PE=1 SV=1
P97427	9.1695E-03	6.332	3.159	7.487	0	14.21	16.98	14.83	13.64	Dihydropyrimidinase-related protein 1 OS=Mus musculus GN=Crmp1 PE=1 SV=1
A0A087WSN6	9.2657E-03	37.2	36.86	43.99	41.33	377.6	349.1	194	271.1	Fibronectin OS=Mus musculus GN=Fn1 PE=1 SV=1
P62259	9.2766E-03	26.91	35.8	32.76	41.33	50.85	50.95	43.25	60.52	14-3-3 protein epsilon OS=Mus musculus GN=Ywhae PE=1 SV=1
P17918	9.4833E-03	42.74	41.07	34.63	47.23	31.41	27.36	25.95	28.13	Proliferating cell nuclear antigen OS=Mus musculus GN=Pcna PE=1 SV=2
Q7TMB8	9.5097E-03	32.45	28.43	24.33	50.19	10.47	14.15	9.885	22.16	Cytoplasmic FMR1-interacting protein 1 OS=Mus musculus GN=Cyfip1 PE=1 SV=1
Q62159	9.5305E-03	18.2	14.74	14.04	17.71	36.64	27.36	23.48	27.28	Rho-related GTP-binding protein RhoC OS=Mus musculus GN=Rhoc PE=1 SV=2
Q9Z1N5	9.5894E-03	26.91	29.48	30.88	38.38	17.95	13.21	13.59	16.2	Spliceosome RNA helicase Ddx39b OS=Mus musculus GN=Ddx39b PE=1 SV=1
Q6PHC1	9.6643E-03	116.3	133.7	127.3	100.4	80.76	79.25	79.08	76.72	Alpha-enolase OS=Mus musculus GN=Eno1 PE=1 SV=1
Q9D6X6	9.6692E-03	3.957	4.212	3.743	0	21.69	16.98	12.36	19.61	Serine protease 23 OS=Mus musculus GN=Prss23 PE=2 SV=2
Q63844	9.8165E-03	22.16	17.9	17.78	17.71	11.22	11.32	12.36	6.819	Mitogen-activated protein kinase 3 OS=Mus musculus GN=Mapk3 PE=1 SV=5
P14131	9.8561E-03	35.62	31.59	34.63	47.23	20.19	19.81	17.3	21.31	40S ribosomal protein S16 OS=Mus musculus GN=Rps16 PE=1 SV=4
Q9JJ00	1.0001E-02	0	0	1.872	0	2.243	3.774	4.943	5.115	Phospholipid scramblase 1 OS=Mus musculus GN=Plscr1 PE=1 SV=1
Q91ZW3	9.9990E-03	22.95	28.43	26.2	35.43	8.973	11.32	12.36	8.524	SWI/SNF-related matrix-associated actin-dependent regulator of chromatin subfamily A member 5 OS=Mus musculus GN=Smarca5 PE=1 SV=1
Q8BKG3	1.0103E-02	4.749	2.106	0	5.904	17.2	12.27	19.77	16.2	Inactive tyrosine-protein kinase 7 OS=Mus musculus GN=Ptk7 PE=1 SV=1
Q501J6	1.0175E-02	30.87	26.33	29.95	35.43	18.69	16.98	14.83	14.49	Probable ATP-dependent RNA helicase DDX17 OS=Mus musculus GN=Ddx17 PE=1 SV=1
Z4YJS5	1.0354E-02	24.54	24.22	25.27	14.76	42.62	43.4	56.84	29.84	Agrin OS=Mus musculus GN=Agrn PE=1 SV=1
Q9CR57	1.0400E-02	7.123	5.265	5.615	0	11.22	12.27	13.59	10.23	60S ribosomal protein L14 OS=Mus musculus GN=Rpl14 PE=1 SV=3
B7ZWL1	1.0402E-02	11.08	10.53	14.04	8.857	0	0	0	3.41	CCR4-NOT transcription complex subunit 1 OS=Mus musculus GN=Cnot1 PE=1 SV=1
Q6ZQ08	1.0402E-02	11.08	10.53	14.04	8.857	0	0	0	3.41	CCR4-NOT transcription complex subunit 1 OS=Mus musculus GN=Cnot1 PE=1 SV=2
P80315	1.0444E-02	83.1	87.4	94.52	73.8	59.82	47.18	45.72	47.74	T-complex protein 1 subunit delta OS=Mus musculus GN=Cct4 PE=1 SV=3
Q3U741	1.0510E-02	30.87	26.33	29.01	35.43	18.69	16.98	14.83	14.49	DEAD (Asp-Glu-Ala-Asp) box polypeptide 17, isoform CRA_a OS=Mus musculus GN=Ddx17 PE=1 SV=1
P70168	1.0504E-02	40.36	40.01	45.86	41.33	47.11	49.06	49.43	49.44	Importin subunit beta-1 OS=Mus musculus GN=Kpnb1 PE=1 SV=2
J3QMG5	1.0694E-02	100.5	107.4	94.52	135.8	20.19	23.59	56.84	83.54	Protein Gm5786 OS=Mus musculus GN=Gm5786 PE=3 SV=1
O55131	1.0714E-02	22.16	23.17	31.82	20.67	47.11	36.8	44.48	34.95	Septin-7 OS=Mus musculus GN=Sept7 PE=1 SV=1
E9Q1G8	1.0714E-02	22.16	23.17	31.82	20.67	47.11	36.8	44.48	34.95	Septin-7 OS=Mus musculus GN=Sept7 PE=1 SV=2
E9Q9F5	1.0714E-02	22.16	23.17	31.82	20.67	47.11	36.8	44.48	34.95	Septin-7 OS=Mus musculus GN=Sept7 PE=1 SV=2
Q8VEE4	1.0818E-02	10.29	15.8	13.1	17.71	2.243	7.548	9.885	10.23	Replication protein A 70 kDa DNA-binding subunit OS=Mus musculus GN=Rpa1 PE=1 SV=1
P15379	1.1007E-02	3.957	5.265	5.615	0	10.47	10.38	9.885	9.377	CD44 antigen OS=Mus musculus GN=Cd44 PE=1 SV=3
Q9JHR7	1.1073E-02	13.45	14.74	7.487	11.81	4.487	0	0	4.262	Insulin-degrading enzyme OS=Mus musculus GN=Ide PE=1 SV=1
P19324	1.1397E-02	10.29	6.318	9.359	0	24.68	28.31	19.77	23.87	Serpin H1 OS=Mus musculus GN=Serpinh1 PE=1 SV=3
Q5SWN2	1.1419E-02	10.29	15.8	13.1	17.71	2.243	7.548	9.885	9.377	Replication protein A 70 kDa DNA-binding subunit OS=Mus musculus GN=Rpa1 PE=1 SV=2
P97310	1.1443E-02	69.65	85.29	86.1	64.95	47.86	63.21	49.43	48.59	DNA replication licensing factor MCM2 OS=Mus musculus GN=Mcm2 PE=1 SV=3
Q9QUJ7	1.1602E-02	4.749	8.424	2.808	5.904	1.496	2.831	0	0	Long-chain-fatty-acid--CoA ligase 4 OS=Mus musculus GN=Acsl4 PE=1 SV=2
Q8VDW0	1.1637E-02	29.28	21.06	23.4	14.76	12.71	5.661	7.414	8.524	ATP-dependent RNA helicase DDX39A OS=Mus musculus GN=Ddx39a PE=1 SV=1
P26231	1.1905E-02	8.706	9.477	10.29	0	33.65	29.25	22.24	30.69	Catenin alpha-1 OS=Mus musculus GN=Ctnna1 PE=1 SV=1
Q8VDJ3	1.2054E-02	2.374	4.212	2.808	0	9.721	8.492	12.36	11.08	Vigilin OS=Mus musculus GN=Hdlbp PE=1 SV=1
Q61235	1.2083E-02	6.332	0	0	0	8.973	4.718	6.178	3.41	Beta-2-syntrophin OS=Mus musculus GN=Sntb2 PE=1 SV=2
P10126	1.2181E-02	230.3	209.5	238.6	221.4	335.7	322.7	308.9	268.5	Elongation factor 1-alpha 1 OS=Mus musculus GN=Eef1a1 PE=1 SV=3
E9Q8Z5	1.2336E-02	0	5.265	3.743	5.904	19.44	12.27	18.54	20.46	Catenin delta-1 OS=Mus musculus GN=Ctnnd1 PE=1 SV=1
P97760	1.2553E-02	7.915	8.424	13.1	8.857	1.496	0	0	2.557	DNA-directed RNA polymerase II subunit RPB3 OS=Mus musculus GN=Polr2c PE=1 SV=2
A0A1D5RML8	1.2553E-02	7.915	8.424	13.1	8.857	1.496	0	0	2.557	DNA-directed RNA polymerase II subunit RPB3 OS=Mus musculus GN=Polr2c PE=1 SV=1
A0A0U1RPX4	1.2703E-02	21.37	17.9	16.85	17.71	10.47	11.32	0	6.819	Mitogen-activated protein kinase (Fragment) OS=Mus musculus GN=Mapk3 PE=1 SV=1
P48036	1.2725E-02	57.78	43.17	39.31	47.23	71.04	63.21	72.91	71.6	Annexin A5 OS=Mus musculus GN=Anxa5 PE=1 SV=1
P48678	1.2733E-02	15.83	12.64	16.85	20.67	38.88	43.4	40.78	31.54	Prelamin-A/C OS=Mus musculus GN=Lmna PE=1 SV=2
A0A140T8V5	1.2813E-02	37.2	37.91	35.56	47.23	26.92	24.53	23.48	24.72	Proliferating cell nuclear antigen OS=Mus musculus GN=Pcna-ps2 PE=3 SV=1
G5E839	1.2841E-02	77.56	78.98	87.04	64.95	53.09	44.34	42.01	46.03	T-complex protein 1 subunit delta OS=Mus musculus GN=Cct4 PE=1 SV=1
O09106	1.2940E-02	21.37	25.27	29.01	44.28	8.225	13.21	7.414	17.9	Histone deacetylase 1 OS=Mus musculus GN=Hdac1 PE=1 SV=1
O70133	1.2983E-02	117.1	88.45	84.23	67.9	28.42	32.08	27.19	33.24	ATP-dependent RNA helicase A OS=Mus musculus GN=Dhx9 PE=1 SV=2
P80317	1.2997E-02	94.97	105.3	100.1	67.9	59.07	55.67	63.02	50.29	T-complex protein 1 subunit zeta OS=Mus musculus GN=Cct6a PE=1 SV=3
P50580	1.3072E-02	32.45	37.91	49.6	32.47	17.2	17.93	18.54	17.9	Proliferation-associated protein 2G4 OS=Mus musculus GN=Pa2g4 PE=1 SV=3
A2AE89	1.3079E-02	3.957	0	0	0	6.73	7.548	4.943	5.967	Glutathione S-transferase Mu 1 OS=Mus musculus GN=Gstm1 PE=1 SV=2
P10649	1.3079E-02	3.957	0	0	0	6.73	7.548	4.943	5.967	Glutathione S-transferase Mu 1 OS=Mus musculus GN=Gstm1 PE=1 SV=2
P62267	1.3122E-02	34.82	35.8	30.88	17.71	6.73	2.831	7.414	5.967	40S ribosomal protein S23 OS=Mus musculus GN=Rps23 PE=1 SV=3
A0A087WP83	1.3247E-02	2.374	4.212	2.808	0	8.973	8.492	11.12	11.08	Vigilin OS=Mus musculus GN=Hdlbp PE=1 SV=1
P84104	1.3530E-02	7.123	5.265	4.679	8.857	3.739	0	2.471	3.41	Serine/arginine-rich splicing factor 3 OS=Mus musculus GN=Srsf3 PE=1 SV=1
Q99PT1	1.3659E-02	15.04	11.58	7.487	0	31.41	43.4	49.43	45.18	Rho GDP-dissociation inhibitor 1 OS=Mus musculus GN=Arhgdia PE=1 SV=3
G3UZ34	1.3698E-02	21.37	22.11	20.59	14.76	7.478	10.38	12.36	9.377	116 kDa U5 small nuclear ribonucleoprotein component OS=Mus musculus GN=Eftud2 PE=1 SV=1
P12382	1.3745E-02	34.03	37.91	32.76	26.57	17.2	14.15	7.414	17.05	ATP-dependent 6-phosphofructokinase, liver type OS=Mus musculus GN=Pfkl PE=1 SV=4
Q9CZU6	1.3792E-02	24.54	24.22	19.65	23.62	11.96	14.15	12.36	18.75	Citrate synthase, mitochondrial OS=Mus musculus GN=Cs PE=1 SV=1
D3Z3G6	1.4155E-02	22.16	17.9	17.78	17.71	9.721	11.32	0	6.819	Mitogen-activated protein kinase OS=Mus musculus GN=Mapk3 PE=1 SV=1
D3YV80	1.4229E-02	0	2.106	1.872	0	2.243	4.718	3.707	4.262	Unconventional myosin-Ib (Fragment) OS=Mus musculus GN=Myo1b PE=1 SV=1
Q07113	1.4478E-02	3.166	0	0	0	8.225	14.15	13.59	15.34	Cation-independent mannose-6-phosphate receptor OS=Mus musculus GN=Igf2r PE=1 SV=1
Q01768	1.4502E-02	17.41	21.06	20.59	5.904	35.89	35.85	48.19	41.77	Nucleoside diphosphate kinase B OS=Mus musculus GN=Nme2 PE=1 SV=1
Q6P5F7	1.4585E-02	1.583	3.159	0	0	5.234	5.661	6.178	5.967	Protein tweety homolog 3 OS=Mus musculus GN=Ttyh3 PE=1 SV=1
A0A0J9YUL3	1.4623E-02	14.25	18.95	15.91	14.76	29.16	24.53	32.13	26.43	Septin 11, isoform CRA_b OS=Mus musculus GN=Sept11 PE=1 SV=1
Q8C1B7	1.4623E-02	14.25	18.95	15.91	14.76	29.16	24.53	32.13	26.43	Septin-11 OS=Mus musculus GN=Sept11 PE=1 SV=4
A0A0J9YTY0	1.4623E-02	14.25	18.95	15.91	14.76	29.16	24.53	32.13	26.43	Septin-11 OS=Mus musculus GN=Sept11 PE=1 SV=1
Q60864	1.4640E-02	17.41	16.85	23.4	20.67	32.15	37.74	34.6	28.98	Stress-induced-phosphoprotein 1 OS=Mus musculus GN=Stip1 PE=1 SV=1
Q5SUR0	1.4697E-02	18.99	20.01	20.59	14.76	13.46	14.15	16.06	12.79	Phosphoribosylformylglycinamidine synthase OS=Mus musculus GN=Pfas PE=1 SV=1
P26043	1.4775E-02	40.36	43.17	35.56	29.52	64.31	73.59	91.44	61.37	Radixin OS=Mus musculus GN=Rdx PE=1 SV=3
E9Q616	1.4784E-02	22.95	26.33	19.65	41.33	40.38	50.01	63.02	69.05	Protein Ahnak OS=Mus musculus GN=Ahnak PE=1 SV=1
Q9R0B9	1.4968E-02	22.95	21.06	27.14	11.81	0	2.831	0	2.557	Procollagen-lysine,2-oxoglutarate 5-dioxygenase 2 OS=Mus musculus GN=Plod2 PE=1 SV=2
E9Q718	1.4968E-02	22.95	21.06	27.14	11.81	0	2.831	0	2.557	Procollagen-lysine,2-oxoglutarate 5-dioxygenase 2 OS=Mus musculus GN=Plod2 PE=1 SV=1
Q8BKC5	1.4986E-02	20.58	22.11	19.65	29.52	7.478	8.492	9.885	5.967	Importin-5 OS=Mus musculus GN=Ipo5 PE=1 SV=3
A0A0R4J008	1.5163E-02	13.45	12.64	17.78	5.904	7.478	0	6.178	0	Histone deacetylase OS=Mus musculus GN=Hdac2 PE=1 SV=1
P07356	1.5573E-02	97.35	93.72	70.19	44.28	155.5	127.4	119.9	133	Annexin A2 OS=Mus musculus GN=Anxa2 PE=1 SV=2
Q99LD8	1.5789E-02	0	5.265	3.743	0	17.95	14.15	11.12	14.49	N(G),N(G)-dimethylarginine dimethylaminohydrolase 2 OS=Mus musculus GN=Ddah2 PE=1 SV=1
O88544	1.5820E-02	13.45	8.424	11.23	8.857	5.234	3.774	0	3.41	COP9 signalosome complex subunit 4 OS=Mus musculus GN=Cops4 PE=1 SV=1
D3YYI8	1.5919E-02	20.58	25.27	29.01	44.28	8.225	13.21	7.414	17.05	Histone deacetylase OS=Mus musculus GN=Gm10093 PE=3 SV=1
O08529	1.5974E-02	18.99	17.9	11.23	11.81	8.973	7.548	2.471	8.524	Calpain-2 catalytic subunit OS=Mus musculus GN=Capn2 PE=1 SV=4
P16045	1.6282E-02	49.07	22.11	47.73	14.76	74.78	79.25	102.6	94.62	Galectin-1 OS=Mus musculus GN=Lgals1 PE=1 SV=3
P51410	1.6287E-02	11.08	10.53	10.29	17.71	7.478	2.831	0	6.819	60S ribosomal protein L9 OS=Mus musculus GN=Rpl9 PE=2 SV=2
A0A0G2JES3	1.6287E-02	11.08	10.53	10.29	17.71	7.478	2.831	0	6.819	60S ribosomal protein L9 (Fragment) OS=Mus musculus GN=Rpl9 PE=1 SV=1
D3Z795	1.6341E-02	10.29	6.318	6.551	8.857	4.487	0	0	6.819	Proteasome assembly chaperone 1 OS=Mus musculus GN=Psmg1 PE=1 SV=1
Q9JK23	1.6341E-02	10.29	6.318	6.551	8.857	4.487	0	0	6.819	Proteasome assembly chaperone 1 OS=Mus musculus GN=Psmg1 PE=1 SV=1
Q8BZY3	1.6354E-02	4.749	4.212	5.615	0	6.73	7.548	11.12	3.41	Protein Ddx19b OS=Mus musculus GN=Ddx19b PE=1 SV=1
Q8CG48	1.6731E-02	10.29	6.318	2.808	5.904	1.496	0	0	0	Structural maintenance of chromosomes protein 2 OS=Mus musculus GN=Smc2 PE=1 SV=2
O35593	1.6755E-02	7.915	10.53	14.97	14.76	4.487	5.661	9.885	5.967	26S proteasome non-ATPase regulatory subunit 14 OS=Mus musculus GN=Psmd14 PE=1 SV=2
Q924C1	1.6777E-02	11.08	13.69	10.29	5.904	0	3.774	0	2.557	Exportin-5 OS=Mus musculus GN=Xpo5 PE=1 SV=1
Q99L47	1.6949E-02	9.497	14.74	5.615	8.857	17.2	17.93	16.06	17.9	Hsc70-interacting protein OS=Mus musculus GN=St13 PE=1 SV=1
P54775	1.7087E-02	47.49	82.13	72.06	118.1	20.19	25.47	43.25	56.26	26S protease regulatory subunit 6B OS=Mus musculus GN=Psmc4 PE=1 SV=2
Q3U6K9	1.7100E-02	6.332	2.106	5.615	0	17.95	31.14	19.77	23.87	Phosphoserine aminotransferase OS=Mus musculus GN=Psat1 PE=1 SV=1
A0A0J9YUD8	1.7142E-02	3.957	7.371	4.679	0	6.73	14.15	13.59	8.524	High mobility group protein B1 OS=Mus musculus GN=Hmgb1 PE=1 SV=1
Q61598	1.7222E-02	14.25	14.74	13.1	5.904	41.13	27.36	25.95	33.24	Rab GDP dissociation inhibitor beta OS=Mus musculus GN=Gdi2 PE=1 SV=1
Q9JIM1	1.7471E-02	0	7.371	8.423	0	5.982	9.435	12.36	5.967	Equilibrative nucleoside transporter 1 OS=Mus musculus GN=Slc29a1 PE=1 SV=3
P26039	1.7463E-02	21.37	24.22	26.2	20.67	50.1	38.68	37.07	42.62	Talin-1 OS=Mus musculus GN=Tln1 PE=1 SV=2
P47757	1.7695E-02	20.58	24.22	24.33	26.57	34.4	48.12	35.83	36.65	F-actin-capping protein subunit beta OS=Mus musculus GN=Capzb PE=1 SV=3
G3X9V0	1.7823E-02	0	0	5.615	0	3.739	5.661	7.414	5.115	MCG22048, isoform CRA_a OS=Mus musculus GN=Psme2 PE=1 SV=1
Q8BY89	1.7839E-02	8.706	8.424	7.487	5.904	13.46	18.87	21.01	21.31	Choline transporter-like protein 2 OS=Mus musculus GN=Slc44a2 PE=1 SV=2
G3XA10	1.7881E-02	35.62	37.91	48.66	41.33	27.67	21.7	23.48	23.87	Heterogeneous nuclear ribonucleoprotein U OS=Mus musculus GN=Hnrnpu PE=1 SV=1
Q8VEK3	1.7881E-02	35.62	37.91	48.66	41.33	27.67	21.7	23.48	23.87	Heterogeneous nuclear ribonucleoprotein U OS=Mus musculus GN=Hnrnpu PE=1 SV=1
P61358	1.7903E-02	18.2	13.69	14.97	11.81	13.46	5.661	6.178	8.524	60S ribosomal protein L27 OS=Mus musculus GN=Rpl27 PE=1 SV=2
Q61881	1.8008E-02	52.24	66.34	59.9	94.47	24.68	16.98	18.54	17.9	DNA replication licensing factor MCM7 OS=Mus musculus GN=Mcm7 PE=1 SV=1
Q9QUR6	1.8083E-02	55.4	50.54	58.96	41.33	23.93	16.04	12.36	27.28	Prolyl endopeptidase OS=Mus musculus GN=Prep PE=1 SV=1
Q8BU30	1.8119E-02	61.73	62.13	46.79	41.33	17.95	18.87	23.48	23.87	Isoleucine--tRNA ligase, cytoplasmic OS=Mus musculus GN=Iars PE=1 SV=2
E9PZS4	1.8233E-02	6.332	5.265	4.679	8.857	2.991	0	0	0	NHP2-like protein 1 OS=Mus musculus GN=Nhp2l1 PE=1 SV=1
Q99K85	1.8362E-02	6.332	2.106	7.487	0	22.43	38.68	23.48	32.39	Phosphoserine aminotransferase OS=Mus musculus GN=Psat1 PE=1 SV=1
P62962	1.8547E-02	41.95	60.02	52.41	53.14	54.59	98.12	86.5	75.87	Profilin-1 OS=Mus musculus GN=Pfn1 PE=1 SV=2
P35550	1.8548E-02	15.04	22.11	10.29	20.67	0	0	3.707	1.705	rRNA 2'-O-methyltransferase fibrillarin OS=Mus musculus GN=Fbl PE=1 SV=2
P27773	1.8679E-02	4.749	5.265	7.487	5.904	10.47	13.21	9.885	11.08	Protein disulfide-isomerase A3 OS=Mus musculus GN=Pdia3 PE=1 SV=2
A0A0G2JDF8	1.8823E-02	5.54	8.424	9.359	8.857	3.739	1.887	3.707	3.41	Serrate RNA effector molecule homolog (Fragment) OS=Mus musculus GN=Srrt PE=1 SV=1
Q64727	1.9078E-02	18.2	14.74	15.91	20.67	63.56	43.4	34.6	40.92	Vinculin OS=Mus musculus GN=Vcl PE=1 SV=4
P99024	1.9142E-02	258.8	221.1	245.2	135.8	329	299.1	286.7	259.1	Tubulin beta-5 chain OS=Mus musculus GN=Tubb5 PE=1 SV=1

O08810	1.9259E-02	22.16	23.17	21.52	14.76	7.478	10.38	12.36	10.23	116 kDa U5 small nuclear ribonucleoprotein component OS=Mus musculus GN=Eftud2 PE=1 SV=1
A2AH85	1.9259E-02	22.16	23.17	21.52	14.76	7.478	10.38	12.36	10.23	116 kDa U5 small nuclear ribonucleoprotein component OS=Mus musculus GN=Eftud2 PE=1 SV=1
Q99MR6	1.9436E-02	7.915	9.477	14.97	8.857	3.739	1.887	3.707	3.41	Serrate RNA effector molecule homolog OS=Mus musculus GN=Srrt PE=1 SV=1
Q7TPV4	1.9451E-02	32.45	46.33	38.37	29.52	13.46	16.04	16.06	20.46	Myb-binding protein 1A OS=Mus musculus GN=Mybbp1a PE=1 SV=2
Q8R2Q8	1.9477E-02	4.749	0	7.487	0	8.973	10.38	12.36	5.967	Bone marrow stromal antigen 2 OS=Mus musculus GN=Bst2 PE=1 SV=1
P63101	1.9830E-02	61.73	78.98	73	103.3	40.38	29.25	51.9	59.67	14-3-3 protein zeta/delta OS=Mus musculus GN=Ywhaz PE=1 SV=1
Q922F4	1.9995E-02	165.4	158	168.5	112.2	232.6	219.8	187.8	158.6	Tubulin beta-6 chain OS=Mus musculus GN=Tubb6 PE=1 SV=1
Q9CZN7	2.0166E-02	0	3.159	4.679	0	14.21	13.21	8.65	12.79	Serine hydroxymethyltransferase OS=Mus musculus GN=Shmt2 PE=1 SV=1
F8WJK8	2.0318E-02	9.497	14.74	5.615	11.81	17.2	17.93	16.06	17.9	Hsc70-interacting protein OS=Mus musculus GN=St13 PE=1 SV=1
Q62318	2.0297E-02	12.66	18.95	20.59	20.67	8.225	3.774	3.707	5.115	Transcription intermediary factor 1-beta OS=Mus musculus GN=Trim28 PE=1 SV=3
Q99JI4	2.0300E-02	35.62	36.86	33.69	32.47	10.47	21.7	23.48	21.31	26S proteasome non-ATPase regulatory subunit 6 OS=Mus musculus GN=Psmd6 PE=1 SV=1
Q9R190	2.0435E-02	18.99	14.74	9.359	14.76	2.243	3.774	3.707	5.967	Metastasis-associated protein MTA2 OS=Mus musculus GN=Mta2 PE=1 SV=1
P09103	2.0891E-02	2.374	0	3.743	0	10.47	18.87	11.12	13.64	Protein disulfide-isomerase OS=Mus musculus GN=P4hb PE=1 SV=2
P05201	2.1068E-02	1.583	0	0	0	2.243	1.887	2.471	1.705	Aspartate aminotransferase, cytoplasmic OS=Mus musculus GN=Got1 PE=1 SV=3
Q8VDM6	2.1105E-02	9.497	8.424	9.359	11.81	5.234	2.831	4.943	1.705	Heterogeneous nuclear ribonucleoprotein U-like protein 1 OS=Mus musculus GN=Hnrnpul1 PE=1 SV=1
D3Z6N3	2.1164E-02	19.79	21.06	21.52	23.62	12.71	13.21	18.54	12.79	DNA replication licensing factor MCM7 OS=Mus musculus GN=Mcm7 PE=1 SV=1
P17182	2.1348E-02	136.1	151.6	138.5	112.2	94.97	97.18	88.97	98.03	Alpha-enolase OS=Mus musculus GN=Eno1 PE=1 SV=3
A2AMW0	2.1410E-02	20.58	24.22	24.33	26.57	36.64	51.89	35.83	38.36	Capping protein (Actin filament) muscle Z-line, beta, isoform CRA_a OS=Mus musculus GN=Capzb PE=1 SV=1
D3YWF6	2.1469E-02	11.08	12.64	11.23	17.71	6.73	9.435	9.885	15.34	Ubiquitin thioesterase OTUB1 OS=Mus musculus GN=Otub1 PE=1 SV=1
Q7TQI3	2.1469E-02	11.08	12.64	11.23	17.71	6.73	9.435	9.885	15.34	Ubiquitin thioesterase OTUB1 OS=Mus musculus GN=Otub1 PE=1 SV=2
Q8C052	2.1685E-02	9.497	12.64	5.615	11.81	11.96	15.1	11.12	14.49	Microtubule-associated protein 1S OS=Mus musculus GN=Map1s PE=1 SV=2
P16546	2.1823E-02	16.62	12.64	16.85	8.857	21.69	24.53	23.48	23.02	Spectrin alpha chain, non-erythrocytic 1 OS=Mus musculus GN=Sptan1 PE=1 SV=4
A0A0U1RQ05	2.2058E-02	30.08	27.38	21.52	50.19	8.973	11.32	8.65	15.34	Cytoplasmic FMR1-interacting protein 1 (Fragment) OS=Mus musculus GN=Cyfip1 PE=1 SV=1
B1AWE1	2.2323E-02	16.62	15.8	12.17	23.62	5.982	4.718	8.65	9.377	Clathrin light chain A OS=Mus musculus GN=Clta PE=1 SV=1
E9PZF0	2.2299E-02	17.41	26.33	21.52	8.857	38.88	37.74	54.37	46.88	Nucleoside diphosphate kinase OS=Mus musculus GN=Gm20390 PE=3 SV=1
B1AZ46	2.2450E-02	2.374	2.106	0	0	7.478	4.718	4.943	8.524	Brain-specific angiogenesis inhibitor 1-associated protein 2 OS=Mus musculus GN=Baiap2 PE=1 SV=1
Q8BKX1	2.2450E-02	2.374	2.106	0	0	7.478	4.718	4.943	8.524	Brain-specific angiogenesis inhibitor 1-associated protein 2 OS=Mus musculus GN=Baiap2 PE=1 SV=2
Q9R0P5	2.2736E-02	11.08	11.58	9.359	8.857	21.69	21.7	22.24	11.93	Destrin OS=Mus musculus GN=Dstn PE=1 SV=3
A2APM1	2.2903E-02	3.957	5.265	6.551	0	10.47	9.435	9.885	9.377	CD44 antigen OS=Mus musculus GN=Cd44 PE=1 SV=1
A2APM5	2.3080E-02	3.957	5.265	6.551	0	10.47	10.38	9.885	10.23	CD44 antigen OS=Mus musculus GN=Cd44 PE=1 SV=1
Q80X37	2.3080E-02	3.957	5.265	6.551	0	10.47	10.38	9.885	10.23	CD44 antigen OS=Mus musculus GN=Cd44 PE=1 SV=1
Q9CWJ9	2.3065E-02	72.81	82.13	58.02	106.3	51.6	43.4	38.31	54.56	Bifunctional purine biosynthesis protein PURH OS=Mus musculus GN=Atic PE=1 SV=2
Q5SF07	2.3864E-02	4.749	2.106	3.743	0	7.478	11.32	8.65	7.672	Insulin-like growth factor 2 mRNA-binding protein 2 OS=Mus musculus GN=Igf2bp2 PE=1 SV=1
A6X8Z3	2.4269E-02	4.749	2.106	3.743	0	7.478	11.32	8.65	5.967	Insulin-like growth factor 2 mRNA-binding protein 2 OS=Mus musculus GN=Igf2bp2 PE=1 SV=1
Q3TTF6	2.4930E-02	3.166	8.424	5.615	5.904	0	0	0	2.557	Putative uncharacterized protein OS=Mus musculus GN=Ppp2r1b PE=1 SV=1
H3BKU1	2.4930E-02	3.166	8.424	5.615	5.904	0	0	0	2.557	Protein phosphatase 2 (Formerly 2A), regulatory subunit A (PR 65), beta isoform, isoform CRA_b OS=Mus musculus GN=Ppp2r1b PE=1 SV=1
G3UWS4	2.4930E-02	3.166	8.424	5.615	5.904	0	0	0	2.557	Serine/threonine-protein phosphatase 2A 65 kDa regulatory subunit A beta isoform OS=Mus musculus GN=Ppp2r1b PE=1 SV=1
H3BLQ0	2.4930E-02	3.166	8.424	5.615	5.904	0	0	0	2.557	Serine/threonine-protein phosphatase 2A 65 kDa regulatory subunit A beta isoform OS=Mus musculus GN=Ppp2r1b PE=1 SV=1
E9Q0U1	2.4974E-02	14.25	11.58	15.91	8.857	2.991	4.718	6.178	5.967	26S proteasome non-ATPase regulatory subunit 13 OS=Mus musculus GN=Psmd13 PE=1 SV=1
P43247	2.5520E-02	11.08	10.53	2.808	8.857	0	0	0	1.705	DNA mismatch repair protein Msh2 OS=Mus musculus GN=Msh2 PE=1 SV=1
V9GXB6	2.5604E-02	2.374	3.159	3.743	5.904	0	1.887	0	4.262	Heterogeneous nuclear ribonucleoprotein L-like OS=Mus musculus GN=Hnrnpll PE=1 SV=1
Q6P2B1	2.5639E-02	7.123	9.477	5.615	11.81	4.487	0	0	2.557	Transportin-3 OS=Mus musculus GN=Tnpo3 PE=1 SV=1
Q07797	2.5674E-02	11.08	15.8	13.1	0	31.41	35.85	21.01	31.54	Galectin-3-binding protein OS=Mus musculus GN=Lgals3bp PE=1 SV=1
E9QNH6	2.5888E-02	0	2.106	1.872	0	2.991	5.661	3.707	5.967	Unconventional myosin-Ib OS=Mus musculus GN=Myo1b PE=1 SV=1
E9Q580	2.5888E-02	0	2.106	1.872	0	2.991	5.661	3.707	5.967	Unconventional myosin-Ib OS=Mus musculus GN=Myo1b PE=1 SV=1
P46735	2.5888E-02	0	2.106	1.872	0	2.991	5.661	3.707	5.967	Unconventional myosin-Ib OS=Mus musculus GN=Myo1b PE=1 SV=3
Q7TQD7	2.5888E-02	0	2.106	1.872	0	2.991	5.661	3.707	5.967	Myo1b protein OS=Mus musculus GN=Myo1b PE=1 SV=1
A6ZI44	2.5873E-02	52.24	78.98	56.15	76.76	99.45	106.6	103.8	90.36	Fructose-bisphosphate aldolase OS=Mus musculus GN=Aldoa PE=1 SV=1
P80313	2.5985E-02	72.81	82.13	72.06	53.14	47.11	35.85	39.54	40.92	T-complex protein 1 subunit eta OS=Mus musculus GN=Cct7 PE=1 SV=1
G3X9K9	2.6066E-02	0	0	8.423	0	3.739	11.32	13.59	6.819	Proteasome activator complex subunit 1 OS=Mus musculus GN=Psme1 PE=1 SV=1
P62071	2.6161E-02	7.123	0	2.808	0	9.721	12.27	14.83	11.08	Ras-related protein R-Ras2 OS=Mus musculus GN=Rras2 PE=1 SV=1
Q6ZWV7	2.6250E-02	0	0	3.743	0	4.487	5.661	4.943	4.262	60S ribosomal protein L35 OS=Mus musculus GN=Rpl35 PE=1 SV=1
E9Q0K6	2.6470E-02	23.74	29.48	23.4	8.857	1.496	2.831	0	3.41	Deoxynucleoside triphosphate triphosphohydrolase SAMHD1 OS=Mus musculus GN=Samhd1 PE=1 SV=1
Q60710	2.6988E-02	23.74	29.48	25.27	8.857	1.496	2.831	0	3.41	Deoxynucleoside triphosphate triphosphohydrolase SAMHD1 OS=Mus musculus GN=Samhd1 PE=1 SV=2
F8WJE0	2.6988E-02	23.74	29.48	25.27	8.857	1.496	2.831	0	3.41	Deoxynucleoside triphosphate triphosphohydrolase SAMHD1 OS=Mus musculus GN=Samhd1 PE=1 SV=1
P07742	2.7021E-02	91.81	78.98	73	32.47	7.478	13.21	0	15.34	Ribonucleoside-diphosphate reductase large subunit OS=Mus musculus GN=Rrm1 PE=1 SV=2
P50396	2.7088E-02	6.332	5.265	3.743	5.904	13.46	17.93	23.48	13.64	Rab GDP dissociation inhibitor alpha OS=Mus musculus GN=Gdi1 PE=1 SV=3
Q61151	2.7081E-02	9.497	7.371	7.487	11.81	5.982	0	4.943	4.262	Serine/threonine-protein phosphatase 2A 56 kDa regulatory subunit epsilon isoform OS=Mus musculus GN=Ppp2r5e PE=1 SV=3
A0A0A6YY91	2.7603E-02	0	4.212	0	0	6.73	5.661	7.414	6.819	Neural cell adhesion molecule 1 (Fragment) OS=Mus musculus GN=Ncam1 PE=1 SV=1
D3Z2T9	2.7631E-02	36.41	32.64	39.31	14.76	13.46	12.27	17.3	10.23	Cyclin-dependent kinase 1 (Fragment) OS=Mus musculus GN=Cdk1 PE=1 SV=1
P59708	2.7736E-02	0	6.318	5.615	0	4.487	7.548	8.65	5.115	Splicing factor 3B subunit 6 OS=Mus musculus GN=Sf3b6 PE=1 SV=1
A2BH06	2.7781E-02	26.12	29.48	35.56	47.23	17.95	14.15	19.77	18.75	60S ribosomal protein L11 (Fragment) OS=Mus musculus GN=Rpl11 PE=1 SV=1
F6SVV1	2.7829E-02	9.497	8.424	4.679	8.857	4.487	3.774	3.707	4.262	Protein Gm9493 OS=Mus musculus GN=Gm9493 PE=4 SV=1
A0A1B0GRR3	2.7906E-02	20.58	17.9	22.46	11.81	13.46	12.27	14.83	10.23	40S ribosomal protein S11 OS=Mus musculus GN=Rps11 PE=1 SV=1
P70349	2.7930E-02	3.166	6.318	7.487	0	16.45	11.32	12.36	8.524	Histidine triad nucleotide-binding protein 1 OS=Mus musculus GN=Hint1 PE=1 SV=3
P11688	2.8182E-02	3.957	0	2.808	0	27.67	22.64	7.414	23.02	Integrin alpha-5 OS=Mus musculus GN=Itga5 PE=1 SV=3
Q8R010	2.8209E-02	34.03	41.07	39.31	88.57	8.225	0	0	8.524	Aminoacyl tRNA synthase complex-interacting multifunctional protein 2 OS=Mus musculus GN=Aimp2 PE=1 SV=2
P05064	2.8302E-02	51.44	78.98	56.15	76.76	99.45	106.6	103.8	89.51	Fructose-bisphosphate aldolase A OS=Mus musculus GN=Aldoa PE=1 SV=2
P62631	2.8516E-02	114	114.8	124.5	112.2	144.3	186.8	195.2	138.1	Elongation factor 1-alpha 2 OS=Mus musculus GN=Eef1a2 PE=1 SV=1
Q8JZV7	2.8676E-02	1.583	0	3.743	0	5.234	5.661	4.943	4.262	N-acetylglucosamine-6-phosphate deacetylase OS=Mus musculus GN=Amdhd2 PE=1 SV=1
E9Q390	2.8964E-02	82.31	85.29	77.68	59.04	111.4	131.1	135.9	156.8	Myoferlin OS=Mus musculus GN=Myof PE=1 SV=2
A2APM3	2.9386E-02	3.957	5.265	6.551	0	10.47	9.435	9.885	10.23	CD44 antigen OS=Mus musculus GN=Cd44 PE=1 SV=1
A2APM4	2.9386E-02	3.957	5.265	6.551	0	10.47	9.435	9.885	10.23	CD44 antigen OS=Mus musculus GN=Cd44 PE=1 SV=1
A2APM2	2.9386E-02	3.957	5.265	6.551	0	10.47	9.435	9.885	10.23	CD44 antigen OS=Mus musculus GN=Cd44 PE=1 SV=1
E9QKM8	2.9386E-02	3.957	5.265	6.551	0	10.47	9.435	9.885	10.23	CD44 antigen OS=Mus musculus GN=Cd44 PE=1 SV=1
Q3U8S1	2.9386E-02	3.957	5.265	6.551	0	10.47	9.435	9.885	10.23	CD44 antigen OS=Mus musculus GN=Cd44 PE=1 SV=1
P54754	2.9759E-02	0	0	4.679	0	3.739	6.605	6.178	5.115	Ephrin type-B receptor 3 OS=Mus musculus GN=Ephb3 PE=1 SV=2
contaminant_KERATIN06	2.9775E-02	9.497	6.318	12.17	0	14.21	17.93	16.06	6.819	no description
Q69ZN7	2.9905E-02	83.1	85.29	77.68	59.04	111.4	131.1	135.9	156.8	Myoferlin OS=Mus musculus GN=Myof PE=1 SV=2
P62960	3.0311E-02	31.66	30.54	25.27	26.57	15.7	14.15	19.77	19.61	Nuclease-sensitive element-binding protein 1 OS=Mus musculus GN=Ybx1 PE=1 SV=3
P17426	3.0555E-02	6.332	5.265	3.743	0	9.721	9.435	6.178	7.672	AP-2 complex subunit alpha-1 OS=Mus musculus GN=Ap2a1 PE=1 SV=1
P13595	3.0679E-02	0	4.212	0	0	7.478	5.661	7.414	8.524	Neural cell adhesion molecule 1 OS=Mus musculus GN=Ncam1 PE=1 SV=3
E9QB01	3.0679E-02	0	4.212	0	0	7.478	5.661	7.414	8.524	Neural cell adhesion molecule 1 OS=Mus musculus GN=Ncam1 PE=1 SV=2
A0A0A6YY47	3.0679E-02	0	4.212	0	0	7.478	5.661	7.414	8.524	Neural cell adhesion molecule 1 OS=Mus musculus GN=Ncam1 PE=1 SV=1
Q05816	3.0910E-02	3.957	4.212	3.743	0	35.89	32.08	9.885	21.31	Fatty acid-binding protein, epidermal OS=Mus musculus GN=Fabp5 PE=1 SV=3
Q64282	3.1493E-02	1.583	0	0	0	5.982	9.435	16.06	7.672	Interferon-induced protein with tetratricopeptide repeats 1 OS=Mus musculus GN=Ifit1 PE=1 SV=2
Q9DBZ5	3.1550E-02	5.54	7.371	3.743	8.857	3.739	0	0	2.557	Eukaryotic translation initiation factor 3 subunit K OS=Mus musculus GN=Eif3k PE=1 SV=1
Q3ULG5	3.1860E-02	43.53	43.17	39.31	29.52	19.44	21.7	19.77	25.57	DNA helicase OS=Mus musculus GN=Mcm6 PE=1 SV=1
P97311	3.1860E-02	43.53	43.17	39.31	29.52	19.44	21.7	19.77	25.57	DNA replication licensing factor MCM6 OS=Mus musculus GN=Mcm6 PE=1 SV=1
Q9ESX5	3.2023E-02	8.706	10.53	10.29	0	16.45	14.15	22.24	15.34	H/ACA ribonucleoprotein complex subunit 4 OS=Mus musculus GN=Dkc1 PE=1 SV=4
Q921M3	3.2054E-02	12.66	11.58	19.65	11.81	6.73	6.605	9.885	9.377	Splicing factor 3B subunit 3 OS=Mus musculus GN=Sf3b3 PE=1 SV=1
Q04750	3.2434E-02	11.87	21.06	20.59	29.52	5.982	7.548	4.943	4.262	DNA topoisomerase 1 OS=Mus musculus GN=Top1 PE=1 SV=2
B1AWE0	3.2768E-02	16.62	15.8	12.17	23.62	5.234	4.718	9.885	9.377	Clathrin light chain A OS=Mus musculus GN=Clta PE=1 SV=1
B1AWD9	3.2768E-02	16.62	15.8	12.17	23.62	5.234	4.718	9.885	9.377	Clathrin light chain A OS=Mus musculus GN=Clta PE=1 SV=1
Q6PFA2	3.2768E-02	16.62	15.8	12.17	23.62	5.234	4.718	9.885	9.377	Clathrin light chain A OS=Mus musculus GN=Clta PE=1 SV=1
O08585	3.2768E-02	16.62	15.8	12.17	23.62	5.234	4.718	9.885	9.377	Clathrin light chain A OS=Mus musculus GN=Clta PE=1 SV=2
O70194	3.2741E-02	17.41	16.85	15.91	5.904	11.22	7.548	8.65	4.262	Eukaryotic translation initiation factor 3 subunit D OS=Mus musculus GN=Eif3d PE=1 SV=2
P97371	3.2811E-02	0	0	9.359	0	3.739	11.32	13.59	6.819	Proteasome activator complex subunit 1 OS=Mus musculus GN=Psme1 PE=1 SV=2
Q05D44	3.3251E-02	5.54	9.477	3.743	5.904	1.496	5.661	2.471	4.262	Eukaryotic translation initiation factor 5B OS=Mus musculus GN=Eif5b PE=1 SV=2
Q7TSG6	3.3599E-02	39.57	41.07	35.56	0	51.6	67.93	80.32	52.85	Radixin OS=Mus musculus GN=Rdx PE=1 SV=1
P05132	3.3724E-02	2.374	2.106	4.679	0	6.73	12.27	7.414	6.819	cAMP-dependent protein kinase catalytic subunit alpha OS=Mus musculus GN=Prkaca PE=1 SV=3
Q8VDD5	3.3863E-02	215.3	192.7	212.4	245	305.8	285.9	284.2	260	Myosin-9 OS=Mus musculus GN=Myh9 PE=1 SV=4
A0A0A0MQ76	3.4142E-02	12.66	14.74	16.85	5.904	5.982	3.774	4.943	3.41	Nucleolar protein 58 OS=Mus musculus GN=Nop58 PE=1 SV=1
Q9CXW4	3.4280E-02	30.87	31.59	40.24	50.19	22.43	21.7	19.77	23.02	60S ribosomal protein L11 OS=Mus musculus GN=Rpl11 PE=1 SV=4
E9PYL9	3.4280E-02	30.87	31.59	40.24	50.19	22.43	21.7	19.77	23.02	Protein Gm10036 OS=Mus musculus GN=Gm10036 PE=3 SV=1
Q8R1F1	3.4374E-02	17.41	11.58	12.17	11.81	20.19	22.64	23.48	28.13	Niban-like protein 1 OS=Mus musculus GN=Fam129b PE=1 SV=2
Q01853	3.4475E-02	35.62	27.38	41.18	32.47	89.73	92.46	51.9	75.87	Transitional endoplasmic reticulum ATPase OS=Mus musculus GN=Vcp PE=1 SV=4
P54761	3.4542E-02	2.374	0	4.679	0	2.991	2.831	6.178	2.557	Ephrin type-B receptor 4 OS=Mus musculus GN=Ephb4 PE=1 SV=2
Q8C8K1	3.4542E-02	2.374	0	4.679	0	2.991	2.831	6.178	2.557	Ephrin type-B receptor 4 OS=Mus musculus GN=Ephb4 PE=1 SV=1
E9PWK7	3.4542E-02	2.374	0	4.679	0	2.991	2.831	6.178	2.557	Ephrin type-B receptor 4 OS=Mus musculus GN=Ephb4 PE=1 SV=1
E9PX48	3.4680E-02	4.749	2.106	4.679	0	5.982	8.492	9.885	7.672	Dedicator of cytokinesis protein 7 OS=Mus musculus GN=Dock7 PE=1 SV=2
P62196	3.4697E-02	18.99	31.59	26.2	20.67	13.46	12.27	2.471	9.377	26S protease regulatory subunit 8 OS=Mus musculus GN=Psmc5 PE=1 SV=1
Q640N1	3.4719E-02	11.87	11.58	14.97	5.904	44.12	56.61	22.24	37.51	Adipocyte enhancer-binding protein 1 OS=Mus musculus GN=Aebp1 PE=1 SV=1
H7BX99	3.4770E-02	3.166	2.106	2.808	0	8.225	15.1	8.65	16.2	Prothrombin OS=Mus musculus GN=F2 PE=1 SV=1
P19221	3.4770E-02	3.166	2.106	2.808	0	8.225	15.1	8.65	16.2	Prothrombin OS=Mus musculus GN=F2 PE=1 SV=1
E9PXY1	3.4984E-02	3.957	8.424	11.23	20.67	0	0	0	3.41	Cullin-4B OS=Mus musculus GN=Cul4b PE=1 SV=2
A2A432	3.4984E-02	3.957	8.424	11.23	20.67	0	0	0	3.41	Cullin-4B OS=Mus musculus GN=Cul4b PE=1 SV=1
Q8VIJ6	3.5440E-02	8.706	7.371	11.23	5.904	9.721	14.15	18.54	11.93	Splicing factor, proline- and glutamine-rich OS=Mus musculus GN=Sfpq PE=1 SV=1
Q61171	3.5908E-02	5.54	2.106	7.487	0	23.93	26.42	11.12	23.87	Peroxiredoxin-2 OS=Mus musculus GN=Prdx2 PE=1 SV=3
P62274	3.6060E-02	4.749	9.477	5.615	8.857	2.991	0	0	0	40S ribosomal protein S29 OS=Mus musculus GN=Rps29 PE=3 SV=2
E9PWG6	3.6139E-02	7.123	8.424	5.615	14.76	0	1.887	2.471	1.705	Protein Ncapg OS=Mus musculus GN=Ncapg PE=1 SV=1
Q91WK2	3.6437E-02	4.749	6.318	4.679	5.904	2.243	4.718	0	4.262	Eukaryotic translation initiation factor 3 subunit H OS=Mus musculus GN=Eif3h PE=1 SV=1
A2AKI5	3.6733E-02	3.166	2.106	0	8.857	20.94	17.93	24.71	12.79	Integrin alpha-V OS=Mus musculus GN=Itgav PE=1 SV=1
P43406	3.6733E-02	3.166	2.106	0	8.857	20.94	17.93	24.71	12.79	Integrin alpha-V OS=Mus musculus GN=Itgav PE=1 SV=2
Q61768	3.6797E-02	14.25	22.11	22.46	20.67	11.96	12.27	7.414	8.524	Kinesin-1 heavy chain OS=Mus musculus GN=Kif5b PE=1 SV=3
Q91VC3	3.6945E-02	41.95	34.75	40.24	26.57	10.47	22.64	14.83	17.9	Eukaryotic initiation factor 4A-III OS=Mus musculus GN=Eif4a3 PE=1 SV=3
Q9JM76	3.7036E-02	26.91	22.11	23.4	47.23	14.96	7.548	0	7.672	Actin-related protein 2/3 complex subunit 3 OS=Mus musculus GN=Arpc3 PE=1 SV=3
E9QN08	3.8124E-02	1.583	5.265	4.679	0	11.22	6.605	12.36	10.23	Elongation factor 1-delta (Fragment) OS=Mus musculus GN=Eef1d PE=1 SV=1
Q9CR16	3.8303E-02	9.497	8.424	7.487	17.71	23.18	15.1	17.3	20.46	Peptidyl-prolyl cis-trans isomerase D OS=Mus musculus GN=Ppid PE=1 SV=3
Q99LC2	3.8367E-02	3.166	3.159	3.743	5.904	0	0	2.471	0	Cleavage stimulation factor subunit 1 OS=Mus musculus GN=Cstf1 PE=1 SV=1
P60122	3.8886E-02	39.57	34.75	34.63	53.14	27.67	18.87	25.95	23.02	RuvB-like 1 OS=Mus musculus GN=Ruvbl1 PE=1 SV=1
P25206	3.8893E-02	13.45	13.69	24.33	14.76	8.225	10.38	11.12	4.262	DNA replication licensing factor MCM3 OS=Mus musculus GN=Mcm3 PE=1 SV=2
Q80T06	3.9441E-02	1.583	5.265	4.679	0	11.96	6.605	12.36	10.23	Elongation factor 1-delta OS=Mus musculus GN=Eef1d PE=1 SV=1
P57776	3.9441E-02	1.583	5.265	4.679	0	11.96	6.605	12.36	10.23	Elongation factor 1-delta OS=Mus musculus GN=Eef1d PE=1 SV=3
A0A0R4J1E2	3.9441E-02	1.583	5.265	4.679	0	11.96	6.605	12.36	10.23	Elongation factor 1-delta OS=Mus musculus GN=Eef1d PE=1 SV=1
P80318	3.9641E-02	127.4	129.5	134.8	200.7	74.03	83.03	101.3	87.8	T-complex protein 1 subunit gamma OS=Mus musculus GN=Cct3 PE=1 SV=1
A2A9M4	3.9855E-02	4.749	2.106	4.679	0	5.982	8.492	9.885	8.524	Dedicator of cytokinesis protein 7 OS=Mus musculus GN=Dock7 PE=1 SV=2
A2A9M5	3.9855E-02	4.749	2.106	4.679	0	5.982	8.492	9.885	8.524	Dedicator of cytokinesis protein 7 OS=Mus musculus GN=Dock7 PE=1 SV=1
A0A0U1RNK7	3.9855E-02	4.749	2.106	4.679	0	5.982	8.492	9.885	8.524	Dedicator of cytokinesis protein 7 OS=Mus musculus GN=Dock7 PE=1 SV=1
Q8R1A4	3.9855E-02	4.749	2.106	4.679	0	5.982	8.492	9.885	8.524	Dedicator of cytokinesis protein 7 OS=Mus musculus GN=Dock7 PE=1 SV=3
Q99LX0	3.9922E-02	1.583	0	0	0	2.991	7.548	8.65	11.08	Protein deglycase DJ-1 OS=Mus musculus GN=Park7 PE=1 SV=1
Q61990	4.0220E-02	18.99	17.9	14.97	11.81	13.46	11.32	9.885	11.08	Poly(rC)-binding protein 2 OS=Mus musculus GN=Pcbp2 PE=1 SV=1
Q8R1B4	4.0790E-02	32.45	28.43	35.56	26.57	22.43	24.53	25.95	23.87	Eukaryotic translation initiation factor 3 subunit C OS=Mus musculus GN=Eif3c PE=1 SV=1
Q6PE01	4.0997E-02	15.83	23.17	22.46	32.47	12.71	15.1	12.36	16.2	U5 small nuclear ribonucleoprotein 40 kDa protein OS=Mus musculus GN=Snrnp40 PE=1 SV=1
Q9WU78	4.1530E-02	69.65	68.45	53.34	50.19	80.01	78.31	84.03	80.98	Programmed cell death 6-interacting protein OS=Mus musculus GN=Pdcd6ip PE=1 SV=3
Q6R0H7	4.2890E-02	23.74	20.01	23.4	20.67	14.96	16.04	12.36	17.9	Guanine nucleotide-binding protein G(s) subunit alpha isoforms XLas OS=Mus musculus GN=Gnas PE=1 SV=1
P63094	4.2890E-02	23.74	20.01	23.4	20.67	14.96	16.04	12.36	17.9	Guanine nucleotide-binding protein G(s) subunit alpha isoforms short OS=Mus musculus GN=Gnas PE=1 SV=1
G3X8Y3	4.3003E-02	56.98	64.23	60.83	91.52	32.15	31.14	48.19	34.1	N-alpha-acetyltransferase 15, NatA auxiliary subunit OS=Mus musculus GN=Naa15 PE=1 SV=1
P57780	4.3193E-02	36.41	44.23	29.01	20.67	56.83	50.01	49.43	56.26	Alpha-actinin-4 OS=Mus musculus GN=Actn4 PE=1 SV=1
E9Q715	4.3194E-02	2.374	3.159	4.679	5.904	1.496	0	0	0	Putative RNA-binding protein Luc7-like 2 OS=Mus musculus GN=Luc7l2 PE=1 SV=1
F6WHQ7	4.3269E-02	3.957	0	0	0	4.487	5.661	4.943	5.967	Glutathione S-transferase Mu 1 (Fragment) OS=Mus musculus GN=Gstm1 PE=1 SV=1
A0A1B0GSX0	4.3670E-02	22.16	21.06	18.72	41.33	38.88	33.97	38.31	43.47	L-lactate dehydrogenase OS=Mus musculus GN=Ldha PE=1 SV=1
Q564E2	4.3670E-02	22.16	21.06	18.72	41.33	38.88	33.97	38.31	43.47	L-lactate dehydrogenase OS=Mus musculus GN=Ldha PE=1 SV=1
A0A0U1RNK6	4.3734E-02	10.29	10.53	8.423	8.857	9.721	6.605	3.707	5.967	S-adenosylmethionine synthase OS=Mus musculus GN=Mat2a PE=1 SV=1
A0A1B0GSE8	4.3916E-02	17.41	14.74	18.72	14.76	12.71	14.15	12.36	9.377	40S ribosomal protein S11 (Fragment) OS=Mus musculus GN=Rps11 PE=1 SV=1
A2A813	4.4255E-02	1.583	0	0	0	2.991	5.661	8.65	10.23	Protein deglycase DJ-1 OS=Mus musculus GN=Park7 PE=1 SV=1
Q6P4T2	4.4323E-02	47.49	63.18	64.57	35.43	34.4	27.36	25.95	24.72	U5 small nuclear ribonucleoprotein 200 kDa helicase OS=Mus musculus GN=Snrnp200 PE=1 SV=1

O08553	4.4363E-02	35.62	35.8	33.69	11.81	68.05	76.42	42.01	70.75	Dihydropyrimidinase-related protein 2 OS=Mus musculus GN=Dpysl2 PE=1 SV=2
A2A5N1	4.4372E-02	16.62	8.424	15.91	14.76	18.69	16.98	19.77	18.75	14-3-3 protein beta/alpha (Fragment) OS=Mus musculus GN=Ywhab PE=1 SV=1
Q61072	4.4523E-02	0	3.159	2.808	0	3.739	6.605	14.83	10.23	Disintegrin and metalloproteinase domain-containing protein 9 OS=Mus musculus GN=Adam9 PE=1 SV=2
E9Q638	4.4523E-02	0	3.159	2.808	0	3.739	6.605	14.83	10.23	Disintegrin and metalloproteinase domain-containing protein 9 OS=Mus musculus GN=Adam9 PE=1 SV=1
A0A140LHU0	4.4523E-02	0	3.159	2.808	0	3.739	6.605	14.83	10.23	Disintegrin and metalloproteinase domain-containing protein 9 OS=Mus musculus GN=Adam9 PE=1 SV=1
P40124	4.4783E-02	22.16	21.06	19.65	26.57	28.42	43.4	54.37	42.62	Adenylyl cyclase-associated protein 1 OS=Mus musculus GN=Cap1 PE=1 SV=4
Q6Q477	4.4867E-02	2.374	0	3.743	0	3.739	3.774	4.943	4.262	Plasma membrane calcium-transporting ATPase 4 OS=Mus musculus GN=Atp2b4 PE=1 SV=1
Q6DFW4	4.4909E-02	12.66	14.74	16.85	5.904	5.982	3.774	4.943	4.262	Nucleolar protein 58 OS=Mus musculus GN=Nop58 PE=1 SV=1
Q61024	4.5048E-02	42.74	50.54	46.79	23.62	61.32	74.54	72.91	85.24	Asparagine synthetase [glutamine-hydrolyzing] OS=Mus musculus GN=Asns PE=1 SV=3
A2AFK7	4.5424E-02	37.2	28.43	34.63	23.62	9.721	20.76	8.65	14.49	Eukaryotic initiation factor 4A-III (Fragment) OS=Mus musculus GN=Eif4a3 PE=1 SV=1
P06151	4.5821E-02	22.16	21.06	17.78	41.33	38.14	33.97	38.31	43.47	L-lactate dehydrogenase A chain OS=Mus musculus GN=Ldha PE=1 SV=3
Q8BY71	4.6495E-02	11.08	10.53	10.29	5.904	2.243	2.831	0	5.115	Histone acetyltransferase type B catalytic subunit OS=Mus musculus GN=Hat1 PE=1 SV=1
A2ATU9	4.6495E-02	11.08	10.53	10.29	5.904	2.243	2.831	0	5.115	Histone acetyltransferase type B catalytic subunit OS=Mus musculus GN=Hat1 PE=1 SV=1
P32883	4.6516E-02	7.123	0	6.551	0	8.225	6.605	11.12	8.524	GTPase KRas OS=Mus musculus GN=Kras PE=1 SV=1
P14211	4.6848E-02	7.123	7.371	9.359	11.81	13.46	12.27	12.36	12.79	Calreticulin OS=Mus musculus GN=Calr PE=1 SV=1
Q8K1K2	4.6992E-02	15.04	21.06	14.97	8.857	8.225	7.548	2.471	6.819	26S protease regulatory subunit 8 OS=Mus musculus GN=Psmc5 PE=1 SV=1
A0A1B0GSR9	4.8281E-02	22.16	21.06	16.85	41.33	38.14	33.97	38.31	43.47	L-lactate dehydrogenase OS=Mus musculus GN=Ldha PE=1 SV=1
Q7TNC4	4.8377E-02	2.374	3.159	5.615	8.857	1.496	0	0	2.557	Putative RNA-binding protein Luc7-like 2 OS=Mus musculus GN=Luc7l2 PE=1 SV=1
A2AGT5	4.8740E-02	3.957	3.159	3.743	11.81	0	0	0	1.705	Cytoskeleton-associated protein 5 OS=Mus musculus GN=Ckap5 PE=1 SV=1
Z4YL78	4.8740E-02	3.957	3.159	3.743	11.81	0	0	0	1.705	Cytoskeleton-associated protein 5 OS=Mus musculus GN=Ckap5 PE=1 SV=1
K3W4R5	4.8740E-02	3.957	3.159	3.743	11.81	0	0	0	1.705	Cytoskeleton-associated protein 5 OS=Mus musculus GN=Ckap5 PE=1 SV=1
A0A0R4J0K2	4.8740E-02	3.957	3.159	3.743	11.81	0	0	0	1.705	Cytoskeleton-associated protein 5 OS=Mus musculus GN=Ckap5 PE=1 SV=1
P62281	4.9547E-02	22.95	22.11	26.2	14.76	16.45	16.98	14.83	12.79	40S ribosomal protein S11 OS=Mus musculus GN=Rps11 PE=1 SV=3
Q9Z2U0	5.0970E-02	5.54	0	4.679	0	7.478	6.605	7.414	8.524	Proteasome subunit alpha type-7 OS=Mus musculus GN=Psma7 PE=1 SV=1
Q9WUM3	5.0923E-02	10.29	6.318	9.359	0	11.96	25.47	22.24	20.46	Coronin-1B OS=Mus musculus GN=Coro1b PE=1 SV=1
Q9QZD9	5.1734E-02	32.45	36.86	35.56	14.76	11.96	12.27	9.885	13.64	Eukaryotic translation initiation factor 3 subunit I OS=Mus musculus GN=Eif3i PE=1 SV=1
Q8CFI7	5.1872E-02	23.74	26.33	25.27	11.81	8.973	5.661	2.471	10.23	DNA-directed RNA polymerase II subunit RPB2 OS=Mus musculus GN=Polr2b PE=1 SV=2
Q61398	5.2622E-02	71.23	49.49	41.18	56.09	32.15	32.08	34.6	34.95	Procollagen C-endopeptidase enhancer 1 OS=Mus musculus GN=Pcolce PE=1 SV=2
Q9WV32	5.3215E-02	33.24	34.75	35.56	20.67	25.42	17.93	14.83	17.05	Actin-related protein 2/3 complex subunit 1B OS=Mus musculus GN=Arpc1b PE=1 SV=4
Q9WUA3	5.3466E-02	13.45	18.95	20.59	8.857	4.487	4.718	0	5.967	ATP-dependent 6-phosphofructokinase, platelet type OS=Mus musculus GN=Pfkp PE=1 SV=1
Q8C605	5.3466E-02	13.45	18.95	20.59	8.857	4.487	4.718	0	5.967	ATP-dependent 6-phosphofructokinase OS=Mus musculus GN=Pfkp PE=1 SV=1
P47857	5.3649E-02	13.45	10.53	16.85	8.857	4.487	9.435	6.178	3.41	ATP-dependent 6-phosphofructokinase, muscle type OS=Mus musculus GN=Pfkm PE=1 SV=3
P17742	5.3917E-02	41.16	28.43	23.4	0	57.58	55.67	37.07	52.85	Peptidyl-prolyl cis-trans isomerase A OS=Mus musculus GN=Ppia PE=1 SV=2
Q922I7	5.4312E-02	6.332	8.424	4.679	0	9.721	9.435	11.12	7.672	MCG13402, isoform CRA_c OS=Mus musculus GN=Ptbp1 PE=1 SV=1
Q8BGJ5	5.4782E-02	6.332	8.424	4.679	0	9.721	9.435	9.885	7.672	MCG13402, isoform CRA_a OS=Mus musculus GN=Ptbp1 PE=1 SV=1
Q80UM3	5.5019E-02	54.61	64.23	57.09	91.52	30.66	29.25	46.96	31.54	N-alpha-acetyltransferase 15, NatA auxiliary subunit OS=Mus musculus GN=Naa15 PE=1 SV=1
F7CAZ6	5.5925E-02	16.62	21.06	23.4	26.57	29.91	42.46	29.66	31.54	F-actin-capping protein subunit beta (Fragment) OS=Mus musculus GN=Capzb PE=1 SV=1
Q9D0I9	5.6114E-02	39.57	41.07	34.63	29.52	24.68	25.47	23.48	28.98	Arginine--tRNA ligase, cytoplasmic OS=Mus musculus GN=Rars PE=1 SV=2
G3UYZ1	5.6359E-02	0	5.265	0	0	10.47	5.661	13.59	10.23	Immunoglobulin superfamily member 8 OS=Mus musculus GN=Igsf8 PE=1 SV=1
Q8R366	5.6359E-02	0	5.265	0	0	10.47	5.661	13.59	10.23	Immunoglobulin superfamily member 8 OS=Mus musculus GN=Igsf8 PE=1 SV=2
A0A0R4J117	5.6359E-02	0	5.265	0	0	10.47	5.661	13.59	10.23	Immunoglobulin superfamily member 8 OS=Mus musculus GN=Igsf8 PE=1 SV=1
O55023	5.6507E-02	3.166	3.159	4.679	0	9.721	7.548	4.943	5.967	Inositol monophosphatase 1 OS=Mus musculus GN=Impa1 PE=1 SV=1
Q9CQ65	5.7506E-02	20.58	17.9	22.46	8.857	32.15	34.91	23.48	28.13	S-methyl-5'-thioadenosine phosphorylase OS=Mus musculus GN=Mtap PE=1 SV=1
Q8C253	5.7683E-02	20.58	13.69	15.91	8.857	23.93	38.68	25.95	28.13	Galectin OS=Mus musculus GN=Lgals3 PE=1 SV=1
P19157	5.8350E-02	15.83	20.01	14.97	23.62	14.21	11.32	0	16.2	Glutathione S-transferase P 1 OS=Mus musculus GN=Gstp1 PE=1 SV=2
Q91Z25	5.8410E-02	33.24	34.75	35.56	20.67	26.17	16.98	14.83	17.05	Actin-related protein 2/3 complex subunit 1B OS=Mus musculus GN=Arpc1b PE=1 SV=1
E9PV04	5.8565E-02	39.57	0	40.24	26.57	10.47	0	14.83	0	Protein Gm8994 OS=Mus musculus GN=Gm8994 PE=3 SV=2
P21278	5.8728E-02	6.332	9.477	10.29	5.904	2.991	4.718	0	3.41	Guanine nucleotide-binding protein subunit alpha-11 OS=Mus musculus GN=Gna11 PE=1 SV=1
D3Z2H9	5.8939E-02	21.37	27.38	23.4	38.38	42.62	50.01	48.19	38.36	Protein Tpm3-rs7 OS=Mus musculus GN=Tpm3-rs7 PE=3 SV=1
Q76MZ3	5.8950E-02	63.32	48.44	69.25	73.8	51.6	45.29	42.01	50.29	Serine/threonine-protein phosphatase 2A 65 kDa regulatory subunit A alpha isoform OS=Mus musculus GN=Ppp2r1a PE=1 SV=3
Q9DC48	5.9003E-02	3.166	5.265	3.743	0	0	1.887	0	0	Pre-mRNA-processing factor 17 OS=Mus musculus GN=Cdc40 PE=2 SV=1
A0A0J9YUZ4	5.8986E-02	6.332	8.424	7.487	0	6.73	14.15	14.83	9.377	High mobility group protein B1 (Fragment) OS=Mus musculus GN=Hmgb1 PE=1 SV=1
P63158	5.8986E-02	6.332	8.424	7.487	0	6.73	14.15	14.83	9.377	High mobility group protein B1 OS=Mus musculus GN=Hmgb1 PE=1 SV=2
F8WID5	5.9205E-02	21.37	29.48	0	0	36.64	34.91	39.54	28.13	Tropomyosin alpha-1 chain OS=Mus musculus GN=Tpm1 PE=1 SV=1
P11440	5.9279E-02	61.73	51.6	53.34	14.76	19.44	17.93	18.54	14.49	Cyclin-dependent kinase 1 OS=Mus musculus GN=Cdk1 PE=1 SV=3
A0A0G2JH04	5.9457E-02	13.45	9.477	16.85	26.57	0	9.435	0	11.08	Eukaryotic translation initiation factor 4E OS=Mus musculus GN=Eif4e PE=1 SV=1
A2AW41	5.9822E-02	3.957	0	4.679	0	7.478	4.718	4.943	5.967	Protein Hnrnpr (Fragment) OS=Mus musculus GN=Hnrnpr PE=1 SV=1
Q5SWU9	5.9992E-02	22.95	16.85	26.2	17.71	7.478	13.21	12.36	13.64	Acetyl-CoA carboxylase 1 OS=Mus musculus GN=Acaca PE=1 SV=1

Table S6 Click here to access/download;Table;Table S6.pdf

Table S6. Shown here are top 100 extracellular vesicle biomarkers matched to the proteomic dataset (Figure 2 e,f).

Accession	T-test p-value	Spectral count for ciliated EV (WT)				Spectral count for non-ciliated (KO)				Description	References (Pubmed ID)
		Sample1	Sample2	Sample3	Sample4	Sample1	Sample2	Sample3	Sample4		
**Extracellular vesicle biomarkers exclusively expressed in KO cell-derived vesicles (3 protein fractions)**											
P16125	3.458E-03	0	0	0	0	11	13	12	13	L-lactate dehydrogenase B chain	25857718
P35441	7.000E-03	0	0	0	0	4	7	4	6	Thrombospondin-1	18494037, 20224111
Q6GQT1	1.173E-02	0	0	0	0	4	8	4	6	Alpha-2-macroglobulin-P	26027894
**Extracellular vesicle biomarkers expressed in both WT and KO cell-derived vesicles (33 protein fractions)**											
Q62351	2.907E-04	106	72	82	24	5	5	4	10	Transferrin receptor protein 1	20424270, 15908444
P60766	1.040E-03	26	22	22	7	82	63	48	57	Cell division control protein 42 homolog	23260141, 25944692
Q61739	1.400E-03	36	26	26	9	6	6	7	5	Integrin alpha-6	18494037, 20224111
P10107	1.412E-03	90	73	70	26	229	152	129	205	Annexin A1	11390481, 21362503
P80314	1.582E-03	217	145	230	79	76	70	68	120	T-complex protein 1 subunit beta	23260141, 19415654
F6YY69	3.182E-03	89	81	76	40	23	26	29	67	14-3-3 protein theta	25857718
F8WIT2	3.201E-03	132	80	104	32	219	180	123	210	Annexin	15210972, 25332113
Q5SXR6	3.344E-03	414	294	302	96	189	174	129	202	Clathrin heavy chain	21136642, 25890246
P18760	3.724E-03	67	60	60	17	134	96	86	108	Cofilin-1	16081791, 22635005
B7FAV1	4.455E-03	99	73	99	20	303	195	138	211	Filamin, alpha	19351151
P26041	5.195E-03	111	85	83	28	266	206	188	192	Moesin	18494037, 12519789
Q9CQV8	6.230E-03	73	54	58	34	29	21	24	58	14-3-3 protein beta/alpha	25944692
P40142	6.585E-03	63	52	60	16	38	39	17	23	Transketolase	21136642, 19190083
P62874	7.909E-03	108	89	75	36	45	32	32	44	Guanine nucleotide-binding protein G(I)/G(S)/G(T) subunit beta-1	20424270, 21595033
P62259	9.277E-03	34	34	35	14	68	54	35	71	14-3-3 protein epsilon	15210972, 15326289
Q6PHC1	9.664E-03	147	127	136	34	108	84	64	90	Alpha-enolase	19351151, 17956143
P70168	1.050E-02	51	38	49	14	63	52	40	58	Importin subunit beta-1	23260141, 19056867
P48036	1.273E-02	73	41	42	16	95	67	59	84	Annexin A5	11390481
P07356	1.557E-02	123	89	75	15	208	135	97	156	Annexin A2	23260141, 15210972
Q61598	1.722E-02	18	14	14	2	55	29	21	39	Rab GDP dissociation inhibitor beta	19351151
P62962	1.855E-02	53	57	56	18	73	104	70	89	Profilin-1	17486113
P63101	1.983E-02	78	75	78	35	54	31	42	70	14-3-3 protein zeta/delta	18494037, 20124223
Q07797	2.567E-02	14	15	14	0	42	38	17	37	Galectin-3-binding protein	19028452
A6ZI44	2.587E-02	66	75	60	26	133	113	84	106	Fructose-bisphosphate aldolase	17486113, 26054723
Q8VDD5	3.386E-02	272	183	227	83	409	303	230	305	Myosin-9	23260141
Q01853	3.447E-02	45	26	44	11	120	98	42	89	Transitional endoplasmic reticulum ATPase	21136642
Q61171	3.591E-02	7	2	8	0	32	28	9	28	Peroxiredoxin-2	19351151, 15210972
P80318	3.964E-02	161	123	144	68	99	88	82	103	T-complex protein 1 subunit gamma	23260141, 11390481
Q9WU78	4.153E-02	88	65	57	17	107	83	68	95	Programmed cell death 6-interacting protein	23658846
Q6R0H7	4.289E-02	30	19	25	7	20	17	10	21	Guanine nucleotide-binding protein G(s) subunit alpha isoforms Xlas	25890246, 22635005
P57780	4.319E-02	46	42	31	7	76	53	40	66	Alpha-actinin-4	23260141
A0A1B0GSX0	4.367E-02	28	20	20	14	52	36	31	51	L-lactate dehydrogenase	19351151
P17742	5.392E-02	52	27	25	0	77	59	30	62	Peptidyl-prolyl cis-trans isomerase A	17956143
**Extracellular vesicle biomarkers expressed in both WT and KO cell-derived vesicles, but were not significant (≥ 0.05 p-value) (64 protein fractions)**											
A0A0U1RQ49	6.535E-02	5	4	3	0	0	0	0	0	Hsp70-binding protein 1	20224111, 22635005
P84078	6.609E-02	20	12	9	4	22	18	13	25	ADP-ribosylation factor 1	17486113
P62821	7.497E-02	0	0	0	0	2	3	0	3	Ras-related protein Rab-1A	19351151, 23658846
Q9WV91	7.938E-02	34	29	28	6	38	43	30	47	Prostaglandin F2 receptor negative regulator	20424270, 23260141
P54116	8.166E-02	3	9	10	2	5	11	8	7	Erythrocyte band 7 integral membrane protein	23260141
P07901	8.858E-02	233	174	225	50	194	159	124	177	Heat shock protein HSP 90-alpha	18494037, 20224111
E9Q0C3	1.011E-01	94	93	104	43	98	82	59	88	Heat shock protein HSP 90-beta	23260141, 23333927
P63001	1.021E-01	49	28	42	10	47	53	58	60	Ras-related C3 botulinum toxin substrate 1	18494037
Q9QZF2	1.036E-01	13	15	11	0	3	3	0	2	Glypican-1	23260141, 19199708
P53986	1.070E-01	16	12	16	10	8	7	8	6	Monocarboxylate transporter 1	16081791
Q8VDN2	1.141E-01	125	108	113	23	98	74	50	88	Sodium/potassium-transporting ATPase subunit alpha-1	21136642
Q02053	1.204E-01	120	86	102	22	169	118	72	116	Ubiquitin-like modifier-activating enzyme 1	19351151, 26054723
P41731	1.214E-01	0	0	3	0	3	6	4	0	CD63 antigen	20424270, 16081791
Q7TT50	1.229E-01	6	3	0	0	6	6	8	11	Serine/threonine-protein kinase MRCK beta	15210972, 26054723
Q01279	1.276E-01	3	4	0	0	4	5	5	6	Epidermal growth factor receptor	19109410, 21059916
Q9D091	1.406E-01	10	4	0	0	10	7	10	11	GTPase NRas	23658846, 15326289
P09055	1.447E-01	94	85	90	29	89	73	71	78	Integrin beta-1	18494037, 26054723
Q99JI6	1.464E-01	21	14	16	5	23	20	18	18	Ras-related protein Rap-1b	11390481
O08992	1.727E-01	17	14	14	4	24	16	12	28	Syntenin-1	16081791, 17641064
O08917	1.893E-01	0	0	0	0	5	0	0	6	Flotillin-1	15210972, 21362503
P35700	1.907E-01	53	33	51	7	96	50	31	60	Peroxiredoxin-1	21136642
Q61696	1.991E-01	87	76	81	39	0	0	95	0	Heat shock 70 kDa protein 1A	19190083
P40240	2.076E-01	4	4	0	6	0	0	0	0	CD9 antigen	17700640, 19109410
P62806	2.261E-01	288	226	234	72	327	315	284	232	Histone H4	25242146
P68510	2.310E-01	22	18	26	14	16	18	20	26	14-3-3 protein eta	19351151, 19056867
Q9Z1Q5	2.663E-01	47	32	37	8	43	31	27	30	Chloride intracellular channel protein 1	23260141, 26054723
P55258	2.675E-01	7	0	7	0	4	5	7	7	Ras-related protein Rab-8A	20458337, 25944692
P52480	2.675E-01	301	216	239	80	488	246	166	352	Pyruvate kinase PKM	19109410, 19199708
F2Z483	2.734E-01	30	17	23	7	13	20	17	19	T-complex protein 1 subunit alpha	15210972, 11487543
P10852	3.021E-01	35	26	19	4	33	27	25	38	4F2 cell-surface antigen heavy chain	23260141, 20224111
P63260	3.054E-01	1196	955	993	449	1055	887	881	1094	Actin, cytoplasmic 2	18494037, 16302729
P21956	3.158E-01	230	171	193	80	218	198	145	187	Lactadherin	23260141, 10545503
J3QP71	3.411E-01	22	20	17	5	12	17	15	17	Basigin	23260141
P09411	3.450E-01	184	141	179	76	210	223	191	223	Phosphoglycerate kinase 1	19109410, 11487543
D3Z3I8	3.624E-01	225	178	220	75	367	0	0	244	Elongation factor 1-alpha 1	17486113, 15326289
P20029	3.750E-01	34	20	26	8	31	28	8	23	78 kDa glucose-regulated protein	17486113, 20224111
H3BKG0	3.910E-01	0	0	0	0	0	0	0	4	Caveolin-1	26054723
Q9WVF5	3.910E-01	0	4	0	0	0	0	0	0	Epidermal growth factor receptor	19109410, 21059916
Reverse_sp|P5825	3.910E-01	0	2	0	0	0	0	0	0	Elongation factor 2	23260141, 22723089
P68373	3.937E-01	220	149	149	34	186	135	77	160	Tubulin alpha-1C chain	18494037, 26054723
P35278	4.097E-01	8	8	13	0	5	3	0	7	Ras-related protein Rab-5C	20424270
P08752	4.204E-01	56	39	40	11	52	33	25	47	Guanine nucleotide-binding protein G(i) subunit alpha-2	20424270, 12519789
O35598	4.305E-01	4	5	7	0	8	7	3	5	Disintegrin and metalloproteinase domain-containing protein 10	23658846, 21601258
P68369	4.586E-01	223	150	153	34	188	143	77	165	Tubulin alpha-1A chain	15210972, 22723089
P05213	4.608E-01	225	151	152	34	190	143	76	166	Tubulin alpha-1B chain	23260141, 19837982
P97429	4.743E-01	68	43	52	14	76	42	43	57	Annexin A4	23658846, 25950383
P63017	5.043E-01	348	311	313	148	418	338	237	324	Heat shock cognate 71 kDa protein	12626558, 19351151
A0A0A0MQF6	5.087E-01	137	109	132	59	182	134	74	112	Glyceraldehyde-3-phosphate dehydrogenase	23658846, 25242146
E9Q1F2	5.280E-01	473	343	350	95	543	327	269	465	Actin, cytoplasmic 1	19190083, 10545503
A0A0A6YXF6	5.796E-01	86	71	58	21	52	67	83	119	Transforming protein RhoA	20424270, 26027894
P26040	5.990E-01	70	61	65	18	63	68	64	66	Ezrin	16081791
Q3V117	6.037E-01	77	46	67	26	112	66	40	85	ATP-citrate synthase	23260141, 25265333
Q61187	6.043E-01	4	4	0	0	0	0	0	4	Tumor susceptibility gene 101 protein	19367702, 25265333
Q9EQP2	6.230E-01	30	13	18	2	18	10	8	20	EH domain-containing protein 4	17486113, 17641064
D3Z1M1	7.025E-01	59	43	49	9	35	38	36	52	Guanine nucleotide-binding protein G(I)/G(S)/G(T) subunit beta-2	18494037, 19190083
A0A140LJL0	7.170E-01	7	9	7	0	12	8	0	13	CD81 antigen (Fragment) OS=Mus musculus GN=Cd81 PE=1 SV=1	18494037
P17047	7.225E-01	9	0	4	0	7	5	0	5	Lysosome-associated membrane glycoprotein 2	15210972, 15908444
E9Q3X0	7.352E-01	13	9	15	2	8	15	8	17	Major vault protein	19109410, 19056867
P80316	7.618E-01	129	77	99	22	115	85	80	81	T-complex protein 1 subunit epsilon	23260141
P07724	7.855E-01	0	4	4	13	7	8	11	11	Serum albumin	23260141, 26054723
Q91V41	7.917E-01	16	11	9	0	11	9	5	8	Ras-related protein Rab-14	26027894
A0A0U1RNJ1	8.329E-01	304	204	263	79	315	246	178	280	Fatty acid synthase	23260141, 25265333
P17751	9.189E-01	122	96	106	20	84	92	98	108	Triosephosphate isomerase	21136642, 19530224
P51150	9.417E-01	32	19	18	0	16	14	10	26	Ras-related protein Rab-7a	25944692, 11390481

Table S7. A comparative analysis with primary cilia targeted vesicle study (exocyst-containing vesicles) shows the overlap with an extended list of our significantly expressed EV proteome.

Accession	Mohieldin et. al Current study Description	Xiaofeng Zuo et. al 2019 Description
G3UZ34	116 kDa U5 small nuclear ribonucleoprotein component	116 kDa U5 small nuclear ribonucleoprotein component
Q9CQV8	14-3-3 protein beta/alpha	14-3-3 protein beta/alpha
P68254	14-3-3 protein theta	14-3-3 protein theta
P63101	14-3-3 protein zeta/delta	14-3-3 protein zeta/delta
Q3TXS7	26S proteasome non-ATPase regulatory subunit 1	26S proteasome non-ATPase regulatory subunit 1
Q8VDM4	26S proteasome non-ATPase regulatory subunit 2	26S proteasome non-ATPase regulatory subunit 2
P14685	26S proteasome non-ATPase regulatory subunit 3	26S proteasome non-ATPase regulatory subunit 3
P26516	26S proteasome non-ATPase regulatory subunit 7	26S proteasome non-ATPase regulatory subunit 7
P62281	40S ribosomal protein S11	40S ribosomal protein S11
P14131	40S ribosomal protein S16	40S ribosomal protein S16
P62908	40S ribosomal protein S3	40S ribosomal protein S3
Q9CQ60	6-phosphogluconolactonase	6-phosphogluconolactonase
Q9CR57	60S ribosomal protein L14	60S ribosomal protein L14
P51410	60S ribosomal protein L9	60S ribosomal protein L9
Q91Z25	actin-related protein 2/3 complex subunit 1B	actin-related protein 2/3 complex subunit 1B
P10107	annexin A1	annexin A1
P07356	annexin A2	annexin A2
Q9D0I9	Arginine--tRNA ligase, cytoplasmic	Arginine--tRNA ligase, cytoplasmic
Q9CWJ9	bifunctional purine biosynthesis protein PURH	bifunctional purine biosynthesis protein PURH
Q8R2Q8	bone marrow stromal antigen 2	bone marrow stromal antigen 2
Q8BKX1	brain-specific angiogenesis inhibitor 1-associated protein 2	brain-specific angiogenesis inhibitor 1-associated protein 2
O08529	calpain-2 catalytic subunit	calpain-2 catalytic subunit
P14211	calreticulin	calreticulin
P05132	cAMP-dependent protein kinase catalytic subunit alpha	cAMP-dependent protein kinase catalytic subunit alpha
Q9DBC7	cAMP-dependent protein kinase type I-alpha regulatory subunit	cAMP-dependent protein kinase type I-alpha regulatory subunit
P26231	catenin alpha-1	catenin alpha-1
Q02248	catenin beta-1	catenin beta-1
O35566	CD151 antigen	CD151 antigen
F8WGL3	cofilin-1	cofilin-1
Q04857	collagen alpha-1(VI) chain	collagen alpha-1(VI) chain
P01027	complement C3	complement C3
Q8BH35	complement component C8 beta chain	complement component C8 beta chain
Q9WUM3	Coronin-1B	Coronin-1B
Q7TMB8	Cytoplasmic FMR1-interacting protein 1	Cytoplasmic FMR1-interacting protein 1
Q9D1A2	cytosolic non-specific dipeptidase	cytosolic non-specific dipeptidase
Q04750	DNA topoisomerase 1	DNA topoisomerase 1
P10126	elongation factor 1-alpha 1	elongation factor 1-alpha 1
P58252	elongation factor 2	elongation factor 2
Q8R1B4	eukaryotic translation initiation factor 3 subunit C	eukaryotic translation initiation factor 3 subunit C
Q8BTM8	filamin-A	filamin-A
O70433	four and a half LIM domains protein 2	four and a half LIM domains protein 2
P05064	fructose-bisphosphate aldolase A	fructose-bisphosphate aldolase A
P16045	Galectin-1	Galectin-1
P13020	gelsolin	gelsolin
P06745	glucose-6-phosphate isomerase	glucose-6-phosphate isomerase
P62874	guanine nucleotide-binding protein G(I)/G(S)/G(T) subunit beta-1	guanine nucleotide-binding protein G(I)/G(S)/G(T) subunit beta-1
D3YZX3	guanine nucleotide-binding protein G(I)/G(S)/G(T) subunit beta-2	guanine nucleotide-binding protein G(I)/G(S)/G(T) subunit beta-2
P63158	high mobility group protein B1	high mobility group protein B1
P70349	Histidine triad nucleotide-binding protein 1	Histidine triad nucleotide-binding protein 1
P15864	histone H1.2	histone H1.2
P43274	histone H1.4	histone H1.4
F8WJK8	Hsc70-interacting protein	Hsc70-interacting protein
Q61081	hsp90 co-chaperone Cdc37	hsp90 co-chaperone Cdc37
P70168	importin subunit beta-1	importin subunit beta-1
Q8BKC5	importin-5	importin-5
Q9EPL8	importin-7	importin-7
Q62470	integrin alpha-3	integrin alpha-3

P43406	integrin alpha-V	integrin alpha-V
O88792	junctional adhesion molecule A	junctional adhesion molecule A
P19001	keratin, type I cytoskeletal 19	keratin, type I cytoskeletal 19
Q61768	Kinesin-1 heavy chain	Kinesin-1 heavy chain
P16125	L-lactate dehydrogenase B chain	L-lactate dehydrogenase B chain
P02469	laminin subunit beta-1	laminin subunit beta-1
P02468	laminin subunit gamma-1	laminin subunit gamma-1
Q8VDD5	myosin-9	myosin-9
Q8R1F1	niban-like protein 1	niban-like protein 1
Q5SQB0	nucleophosmin	nucleophosmin
P15532	nucleoside diphosphate kinase A	nucleoside diphosphate kinase A
Q01768	nucleoside diphosphate kinase B	nucleoside diphosphate kinase B
P17742	peptidyl-prolyl cis-trans isomerase A	peptidyl-prolyl cis-trans isomerase A
Q9CR16	Peptidyl-prolyl cis-trans isomerase D	Peptidyl-prolyl cis-trans isomerase D
Q9DBJ1	phosphoglycerate mutase 1	phosphoglycerate mutase 1
B1AX58	plastin-3	plastin-3
P70398	probable ubiquitin carboxyl-terminal hydrolase FAF-X	probable ubiquitin carboxyl-terminal hydrolase FAF-X
P62962	profilin-1	profilin-1
Q9QUR6	prolyl endopeptidase	prolyl endopeptidase
P97371	proteasome activator complex subunit 1	proteasome activator complex subunit 1
Q9Z2U0	proteasome subunit alpha type-7	proteasome subunit alpha type-7
P27773	protein disulfide-isomerase A3	protein disulfide-isomerase A3
P14069	Protein S100-A6	Protein S100-A6
P19221	prothrombin	prothrombin
Q61598	rab GDP dissociation inhibitor beta	rab GDP dissociation inhibitor beta
P24549	retinal dehydrogenase 1	retinal dehydrogenase 1
Q99PT1	rho GDP-dissociation inhibitor 1	rho GDP-dissociation inhibitor 1
Q6P5B0	RRP12-like protein	RRP12-like protein
P60122	ruvB-like 1	ruvB-like 1
H3BLJ9	S-formylglutathione hydrolase	S-formylglutathione hydrolase
P17563	Selenium-binding protein 1	Selenium-binding protein 1
P84104	serine/arginine-rich splicing factor 3	serine/arginine-rich splicing factor 3
P14094	sodium/potassium-transporting ATPase subunit beta-1	sodium/potassium-transporting ATPase subunit beta-1
Q60864	Stress-induced-phosphoprotein 1	Stress-induced-phosphoprotein 1
Q8CG48	structural maintenance of chromosomes protein 2	structural maintenance of chromosomes protein 2
G5E839	T-complex protein 1 subunit delta	T-complex protein 1 subunit delta
P80313	T-complex protein 1 subunit eta	T-complex protein 1 subunit eta
P35441	thrombospondin-1	thrombospondin-1
Q93092	Transaldolase	Transaldolase
Q62351	transferrin receptor protein 1	transferrin receptor protein 1
P40142	transketolase	transketolase
Q8BFY9	transportin-1	transportin-1
Q9CWF2	tubulin beta-2B chain	tubulin beta-2B chain
P48428	tubulin-specific chaperone A	tubulin-specific chaperone A
E9Q634	unconventional myosin-I	unconventional myosin-I
P20152	vimentin	vimentin
Q64727	vinculin	vinculin
O88342	WD repeat-containing protein 1	WD repeat-containing protein 1
Q62523	Zyxin	Zyxin

Table S8. Shown here are the list of 30 exclusively expressed EV proteins isolated from ciliary WT (Table S3) that matched with known cilia specific markers.

Accession	Mohieldin et. al Current study	David U. Mick *et. al* 2015
	**Cilia Specific Protein Descriptions**	**Cilia Specific Protein Descriptions**
Q571I9	Aldehyde dehydrogenase family 16 member A1	Aldehyde dehydrogenase family 16 member A1
O35566	CD151 antigen	CD151 antigen
Q549C9	Cellular tumor antigen p53	Cellular tumor antigen p53
Q91W50	Cold shock domain-containing protein E1	Cold shock domain-containing protein E1
Q01320	DNA topoisomerase 2-alpha	DNA topoisomerase 2-alpha
Q9ERU9	E3 SUM	E3 SUM
Q99JX4	Eukaryotic translation initiation factor 3 subunit M	Eukaryotic translation initiation factor 3 subunit M
Q08943	FACT complex subunit SSRP1	FACT complex subunit SSRP1
P58854	Gamma-tubulin complex component 3	Gamma-tubulin complex component 3
Q9JHK4	Geranylgeranyl transferase type-2 subunit alpha	Geranylgeranyl transferase type-2 subunit alpha
Q9Z2V5	Histone deacetylase 6	Histone deacetylase 6
P43276	Histone H1.5	Histone H1.5
Q8JZK9	Hydroxymethylglutaryl-CoA synthase, cytoplasmic	Hydroxymethylglutaryl-CoA synthase, cytoplasmic
P19001	Keratin, type I cytoskeletal 19	Keratin, type I cytoskeletal 19
Q61001	Laminin subunit alpha-5	Laminin subunit alpha-5
P10404	MLV-related proviral Env polyprotein	MLV-related proviral Env polyprotein
Q6ZWQ0	Nesprin-2	Nesprin-2
Q99P88	Nuclear pore complex protein Nup155	Nuclear pore complex protein Nup155
Q8R480	Nuclear pore complex protein Nup85	Nuclear pore complex protein Nup85
Q6PAV2	Probable E3 ubiquitin-protein ligase HERC4	Probable E3 ubiquitin-protein ligase HERC4
Q810B6	Rabankyrin-5	Rabankyrin-5
P11370	Retrovirus-related Env polyprotein from Fv-4 locus	Retrovirus-related Env polyprotein from Fv-4 locus
F8VPK5	Rho-associated protein kinase	Rho-associated protein kinase
O88796	Ribonuclease P protein subunit p30	Ribonuclease P protein subunit p30
O35130	Ribosomal RNA small subunit methyltransferase NEP1	Ribosomal RNA small subunit methyltransferase NEP1
A0A0R4J23	Septin-10	Septin-10
Q6P5D8	Structural maintenance of chromosomes flexible hinge domain-containing protein 1	Structural maintenance of chromosomes flexible hinge domain-containing protein 1
Q9CXF4	TBC1 domain family member 15	TBC1 domain family member 15
E9PXY8	Ubiquitin carboxyl-terminal hydrolase 7	Ubiquitin carboxyl-terminal hydrolase 7
E9Q3L1	Unconventional myosin-VI	Unconventional myosin-VI
